# Supplementary figures and images for: Male-biased Cyp17a2 orchestrates antiviral sexual dimorphism in fish via STING stabilization and viral protein degradation (part 1 of 5)
Source: eLife. 2026 Feb 18;14:RP108048. doi: 10.7554/eLife.108048 (PMC12916102; doi:10.7554/eLife.108048)

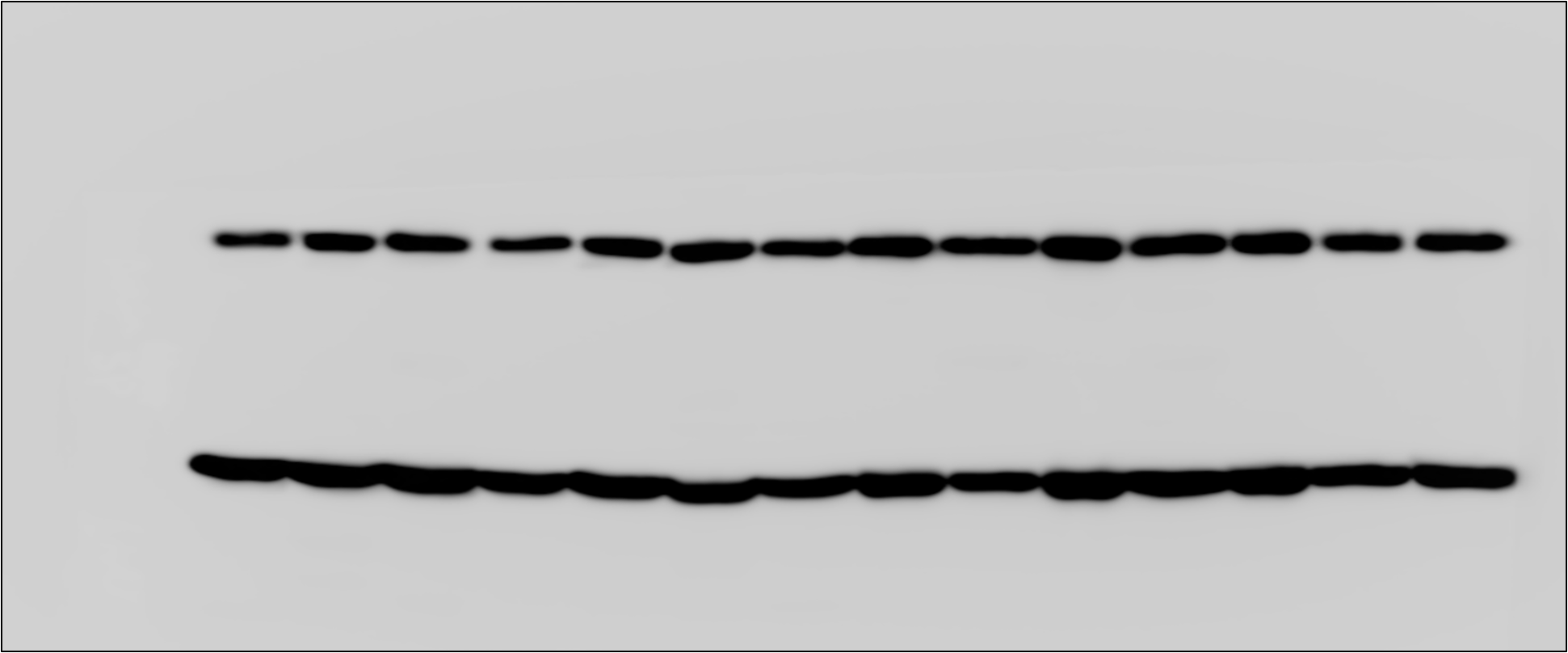

Supplement: Figure 1—source data 2. [file elife-108048-fig1-data2.zip › Figure 1/Figure 1F-Actin-Liver.tif]

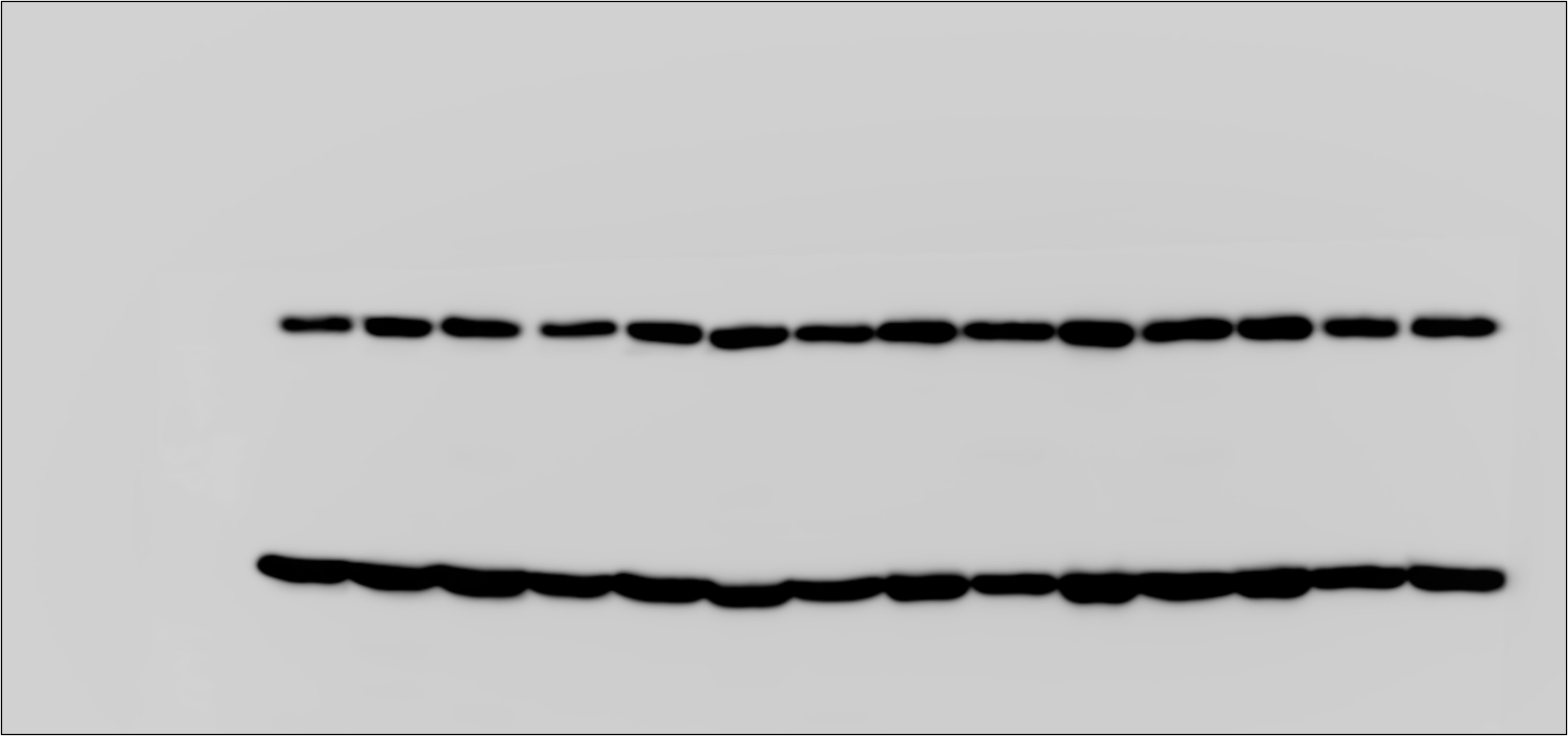

Supplement: Figure 1—source data 2. [file elife-108048-fig1-data2.zip › Figure 1/Figure 1F-Actin-Spleen.tif]

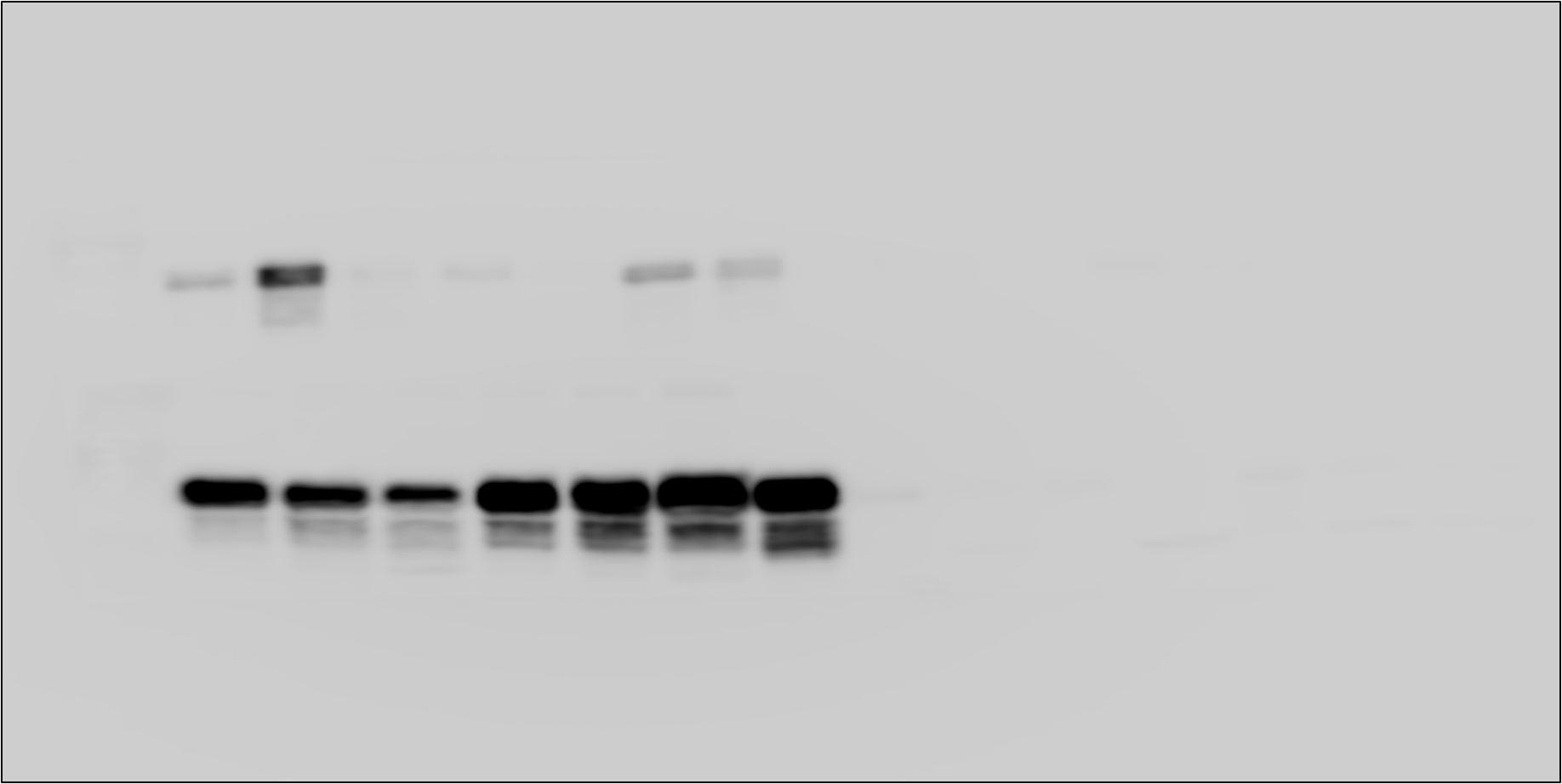

Supplement: Figure 1—source data 2. [file elife-108048-fig1-data2.zip › Figure 1/Figure 1F-G-Liver.tif]

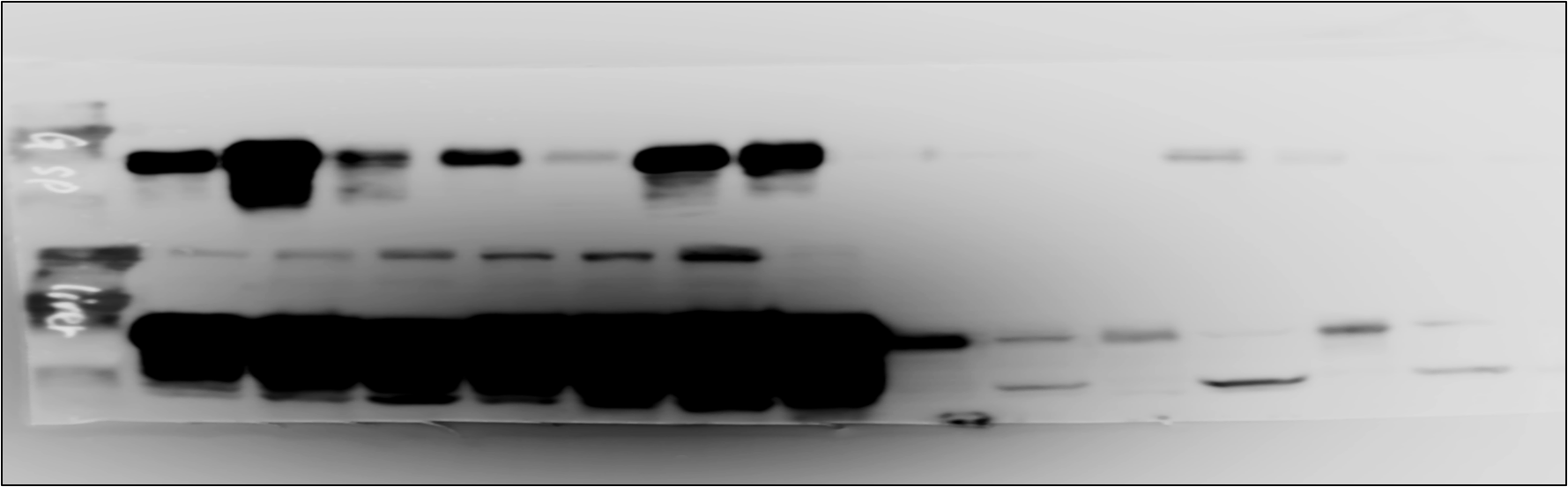

Supplement: Figure 1—source data 2. [file elife-108048-fig1-data2.zip › Figure 1/Figure 1F-G-Spleen.tif]

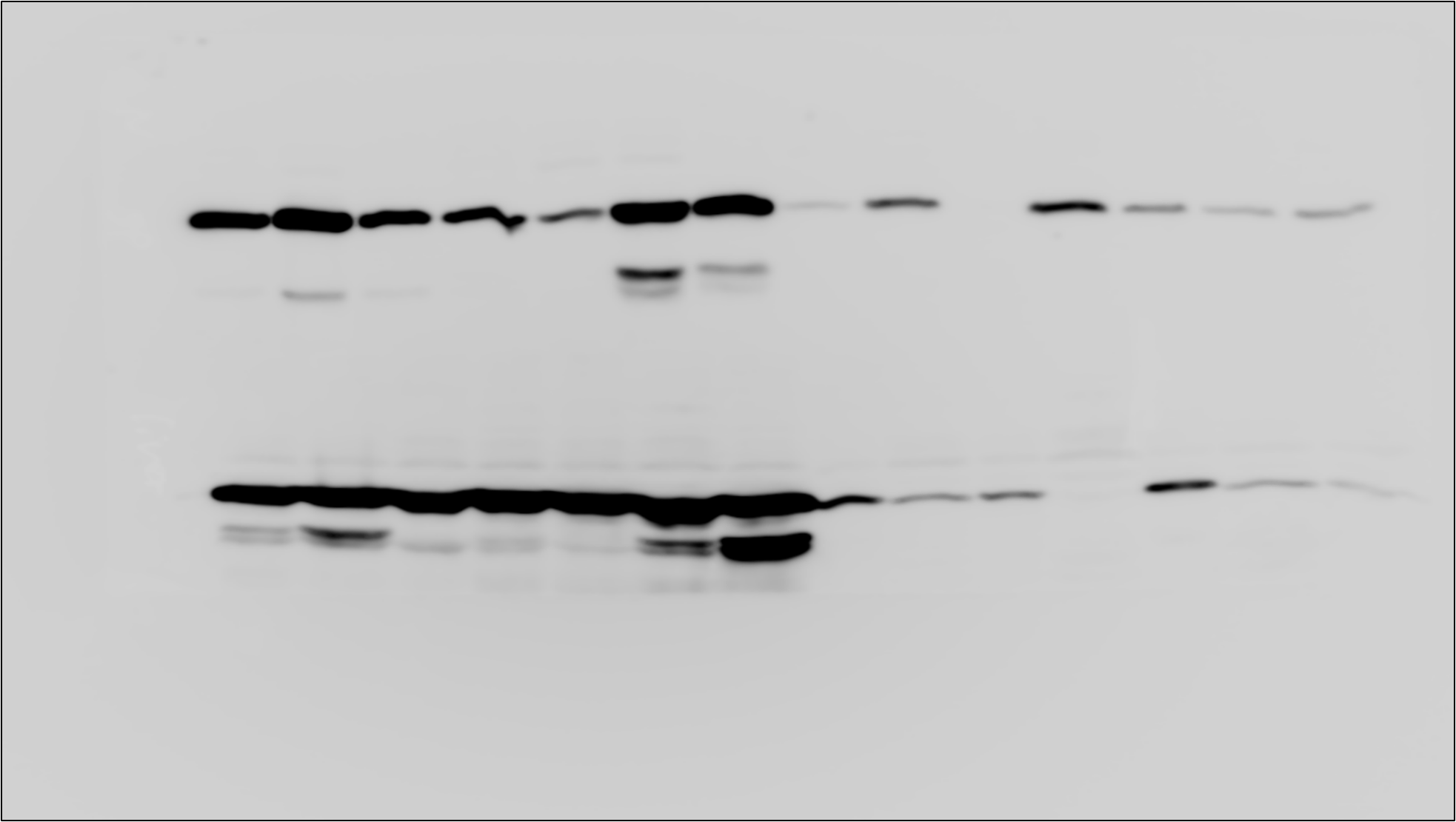

Supplement: Figure 1—source data 2. [file elife-108048-fig1-data2.zip › Figure 1/Figure 1F-N-Liver.tif]

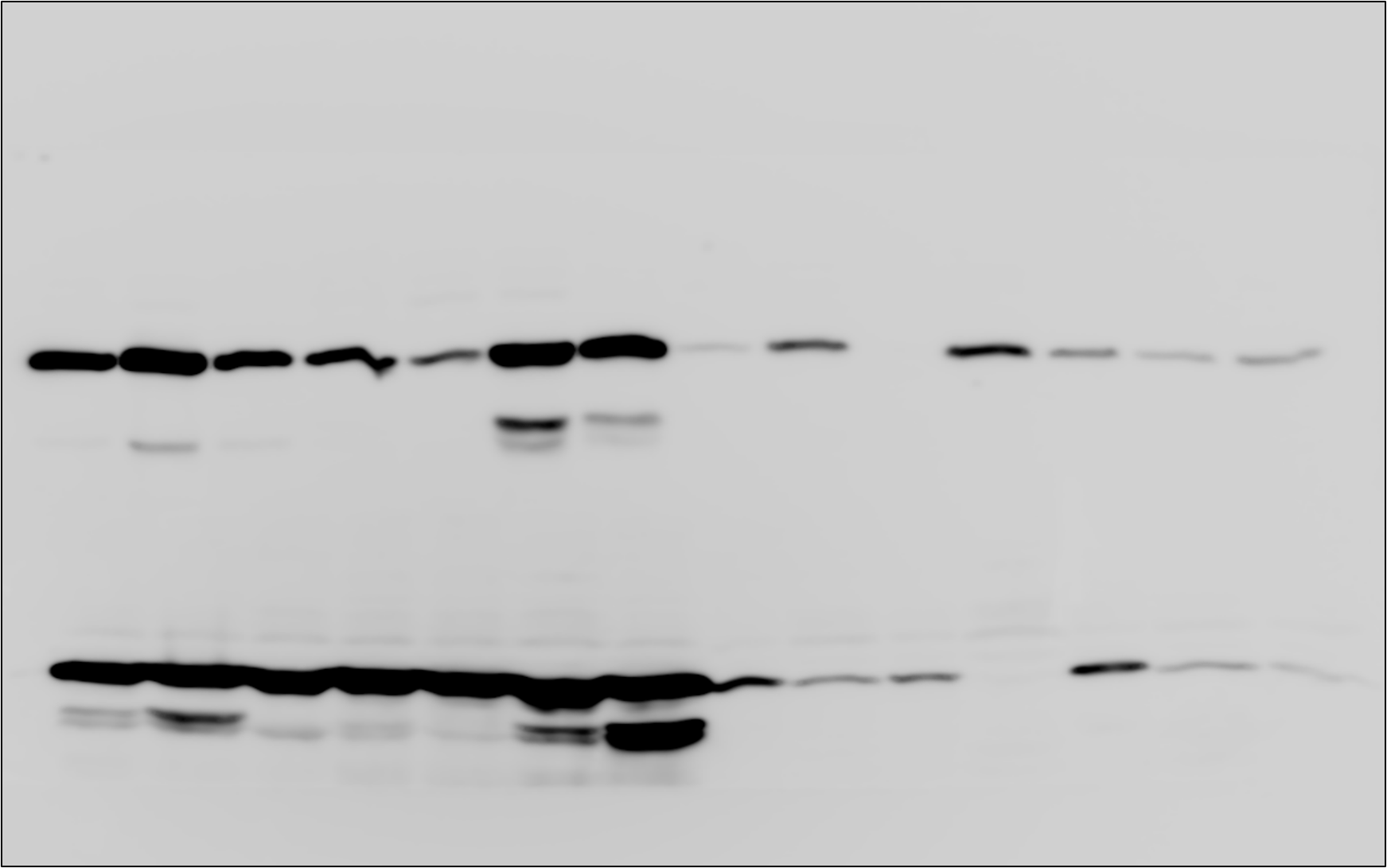

Supplement: Figure 1—source data 2. [file elife-108048-fig1-data2.zip › Figure 1/Figure 1F-N-Spleen.tif]

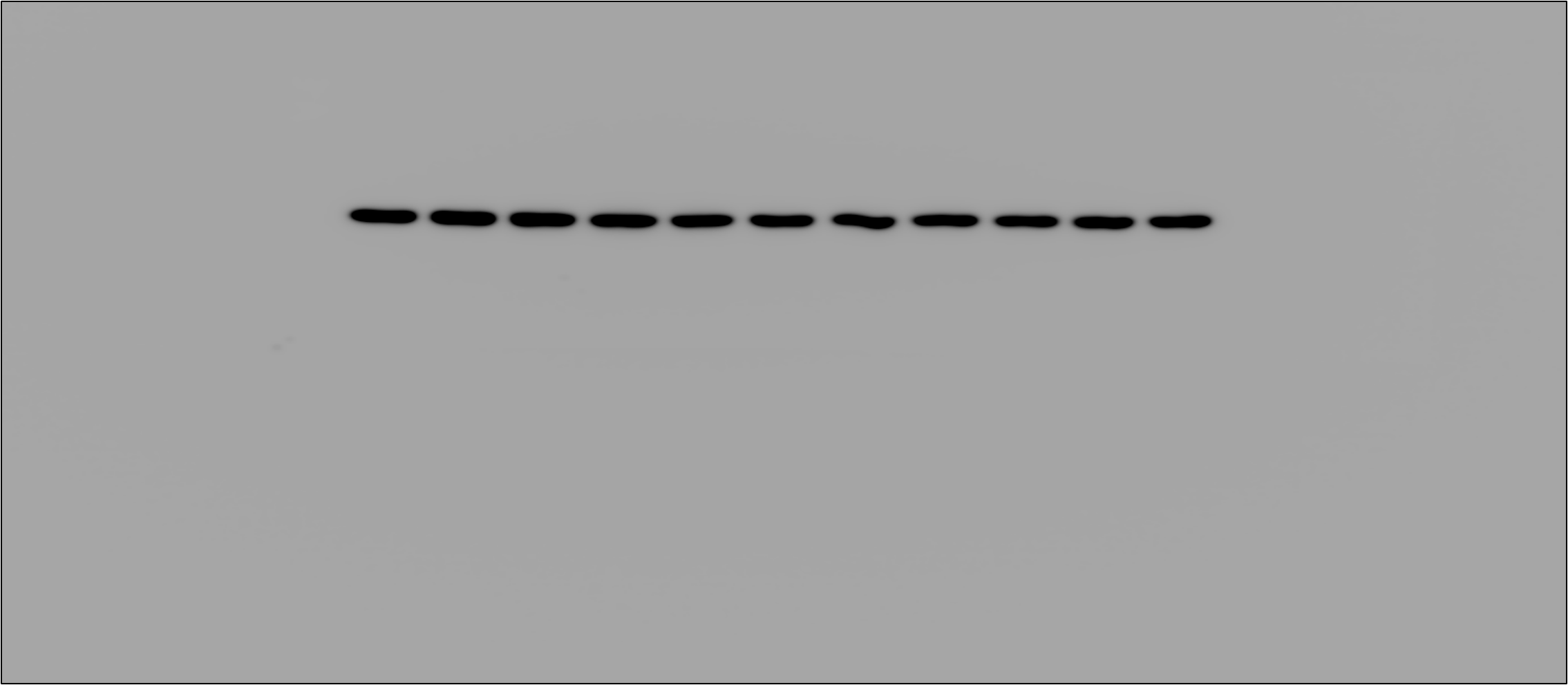

Supplement: Figure 2—source data 2. [file elife-108048-fig2-data2.zip › Figure 2/Figure 2 D-Body-kidney-Actin.tif]

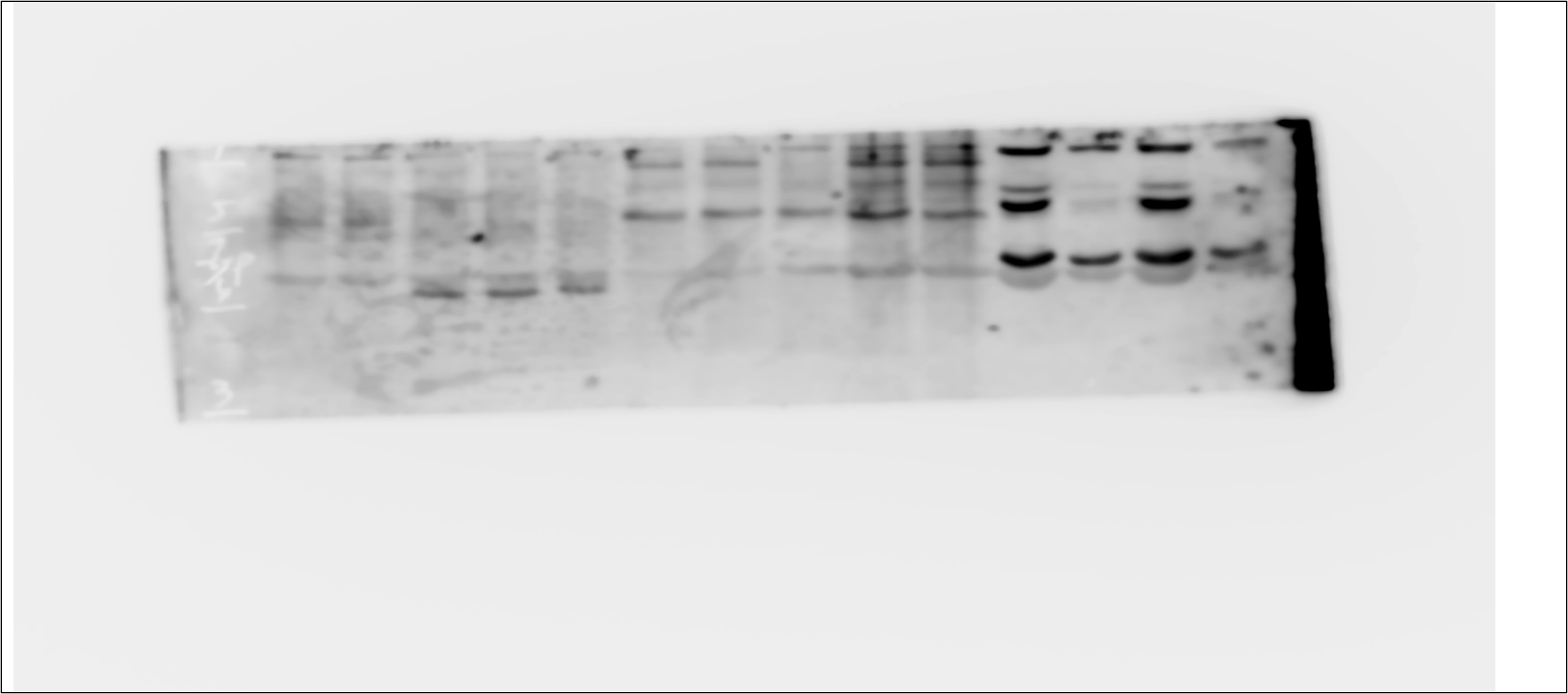

Supplement: Figure 2—source data 2. [file elife-108048-fig2-data2.zip › Figure 2/Figure 2 D-Body-kidney-cyp17a2.tif]

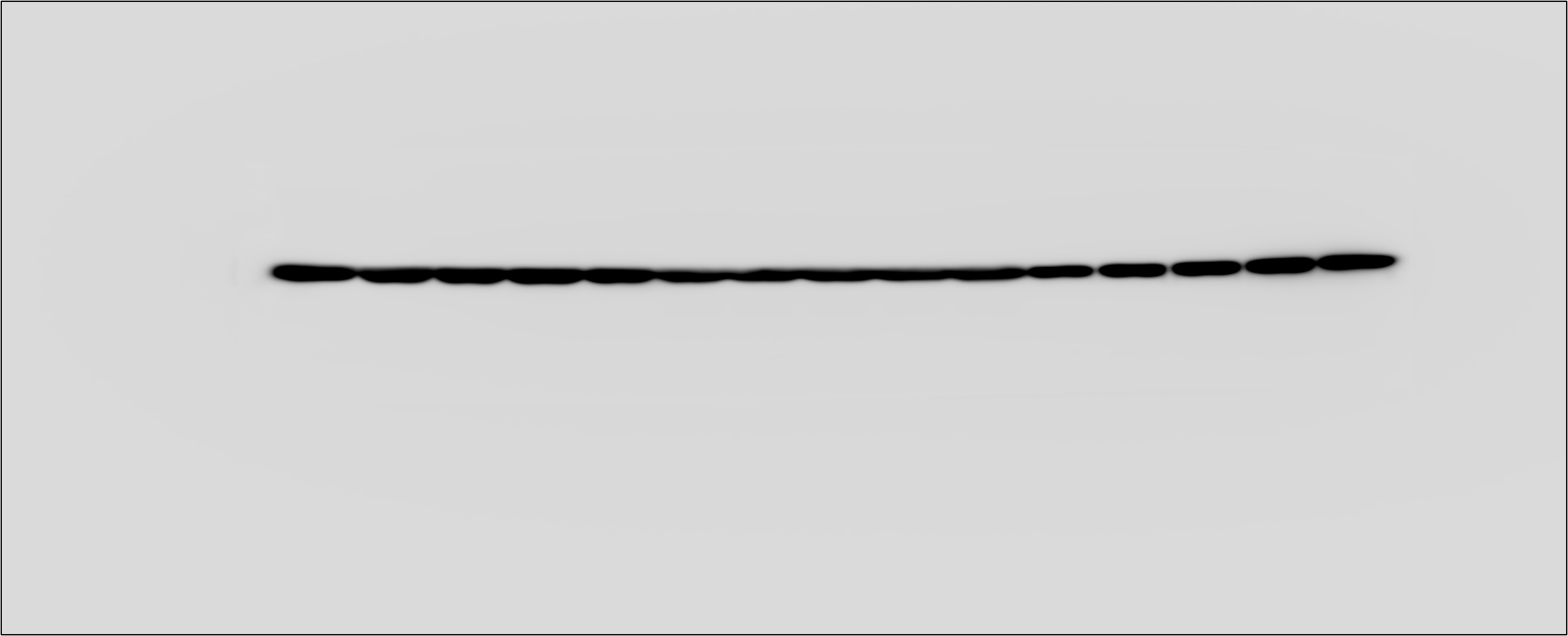

Supplement: Figure 2—source data 2. [file elife-108048-fig2-data2.zip › Figure 2/Figure 2 D-Gill-Actin.tif]

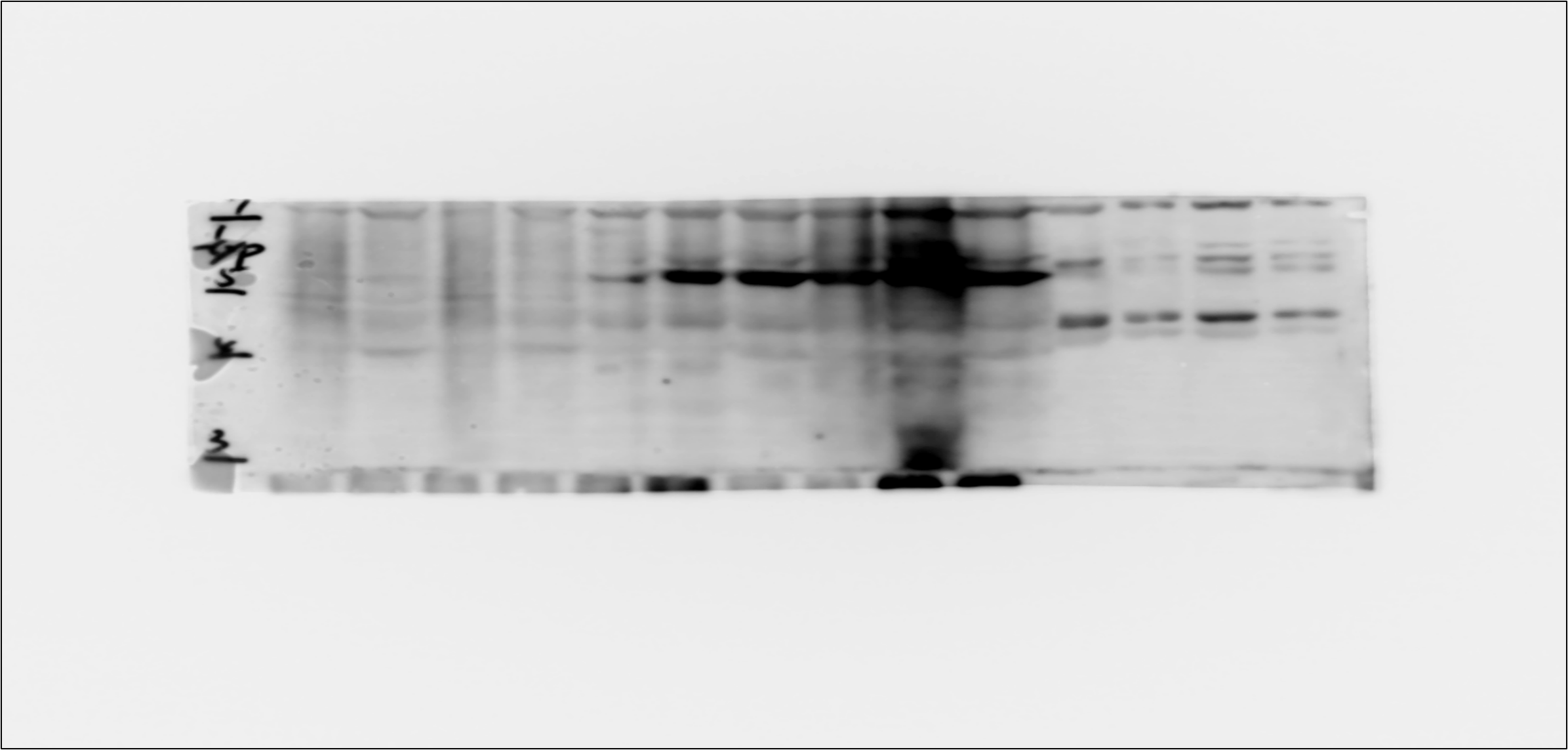

Supplement: Figure 2—source data 2. [file elife-108048-fig2-data2.zip › Figure 2/Figure 2 D-Gill-cyp17a2.tif]

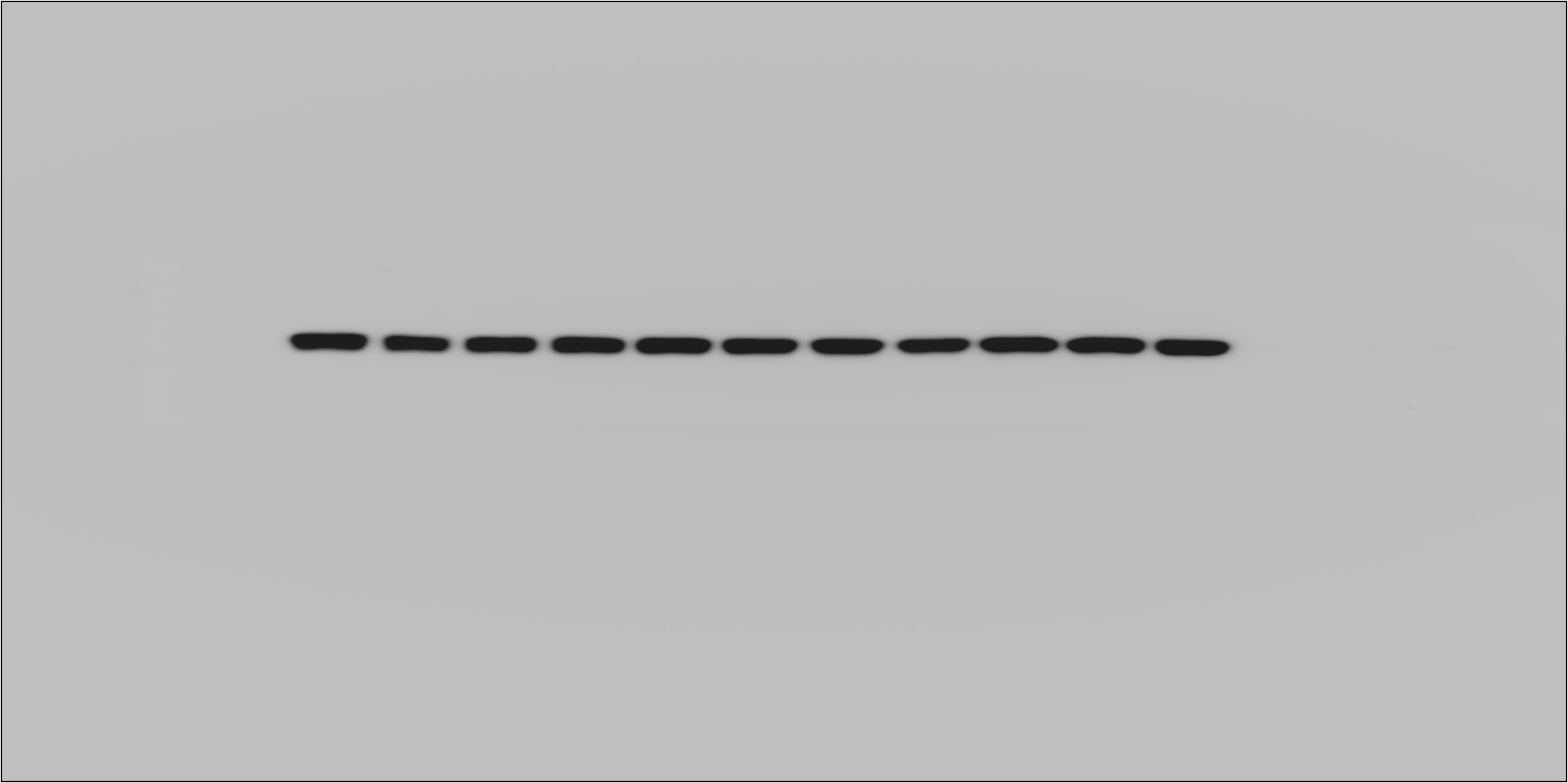

Supplement: Figure 2—source data 2. [file elife-108048-fig2-data2.zip › Figure 2/Figure 2 D-Gonad-Actin.tif]

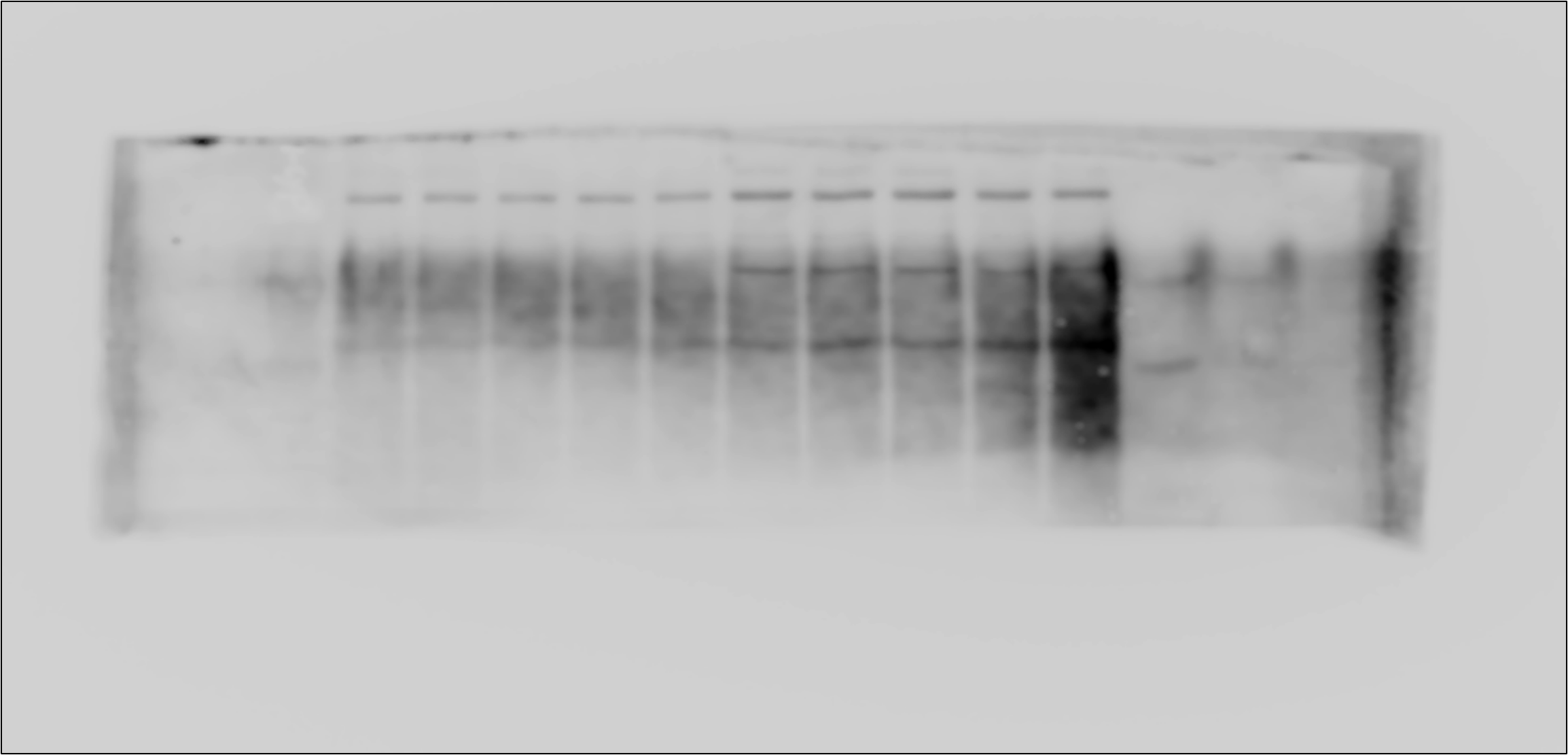

Supplement: Figure 2—source data 2. [file elife-108048-fig2-data2.zip › Figure 2/Figure 2 D-Gonad-cyp17a2.tif]

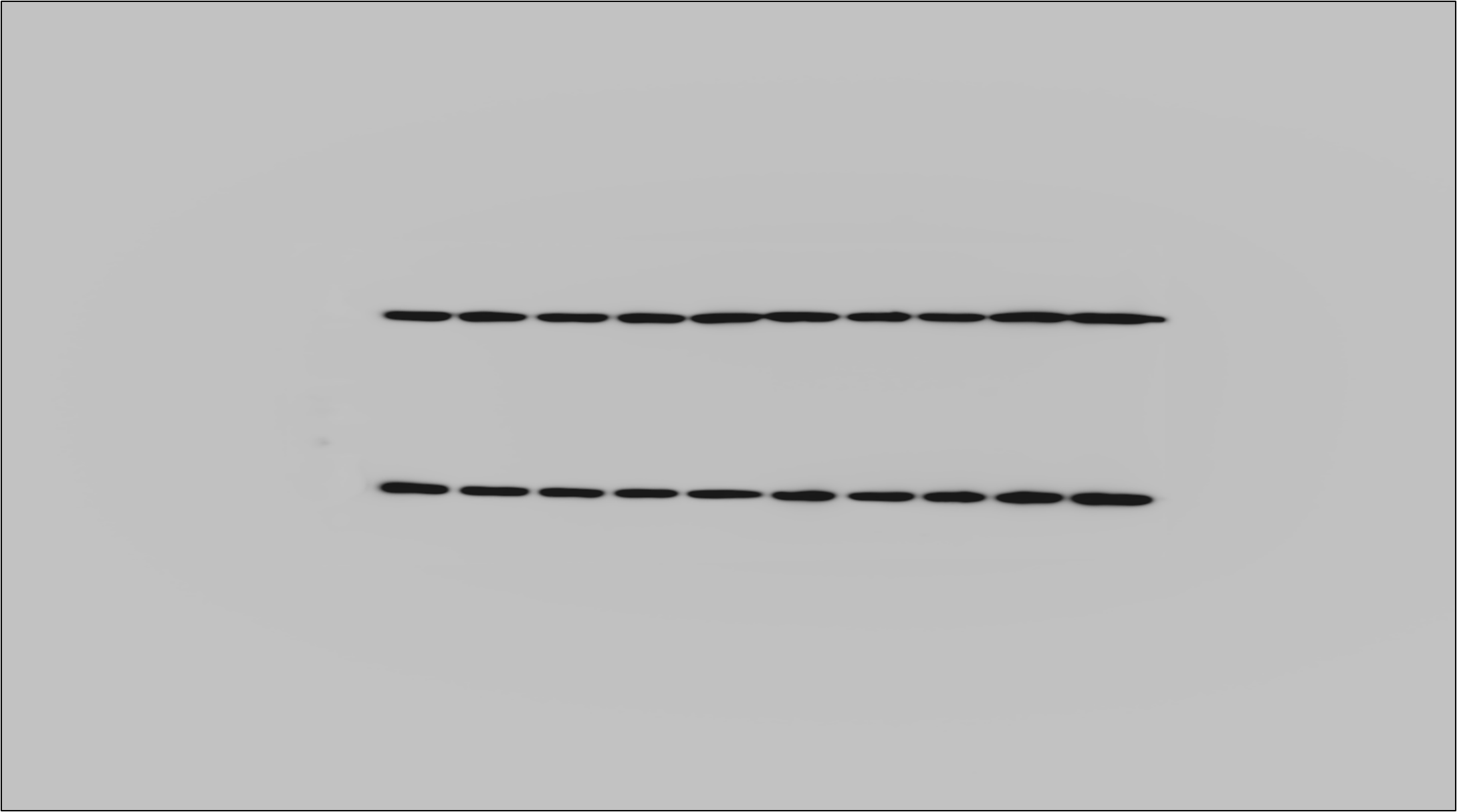

Supplement: Figure 2—source data 2. [file elife-108048-fig2-data2.zip › Figure 2/Figure 2 D-Head-kidney-Actin.tif]

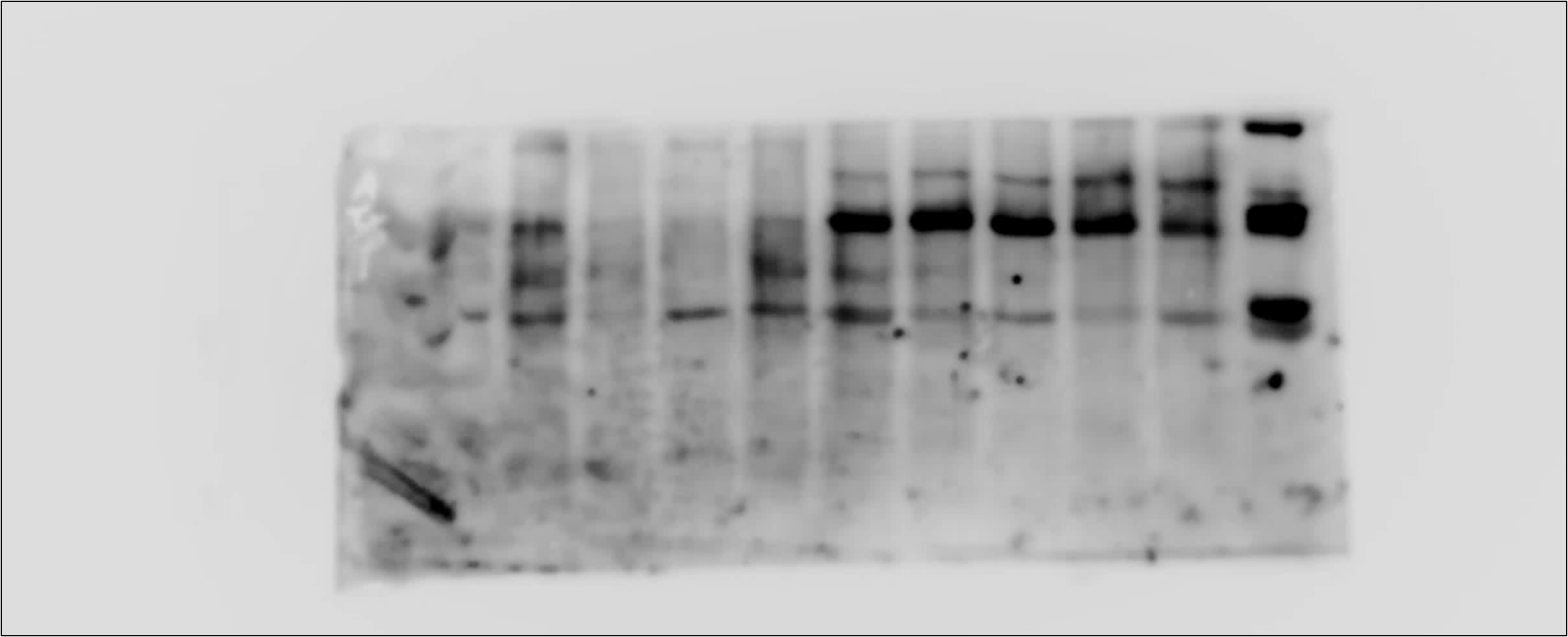

Supplement: Figure 2—source data 2. [file elife-108048-fig2-data2.zip › Figure 2/Figure 2 D-Head-kidney-cyp17a2.tif]

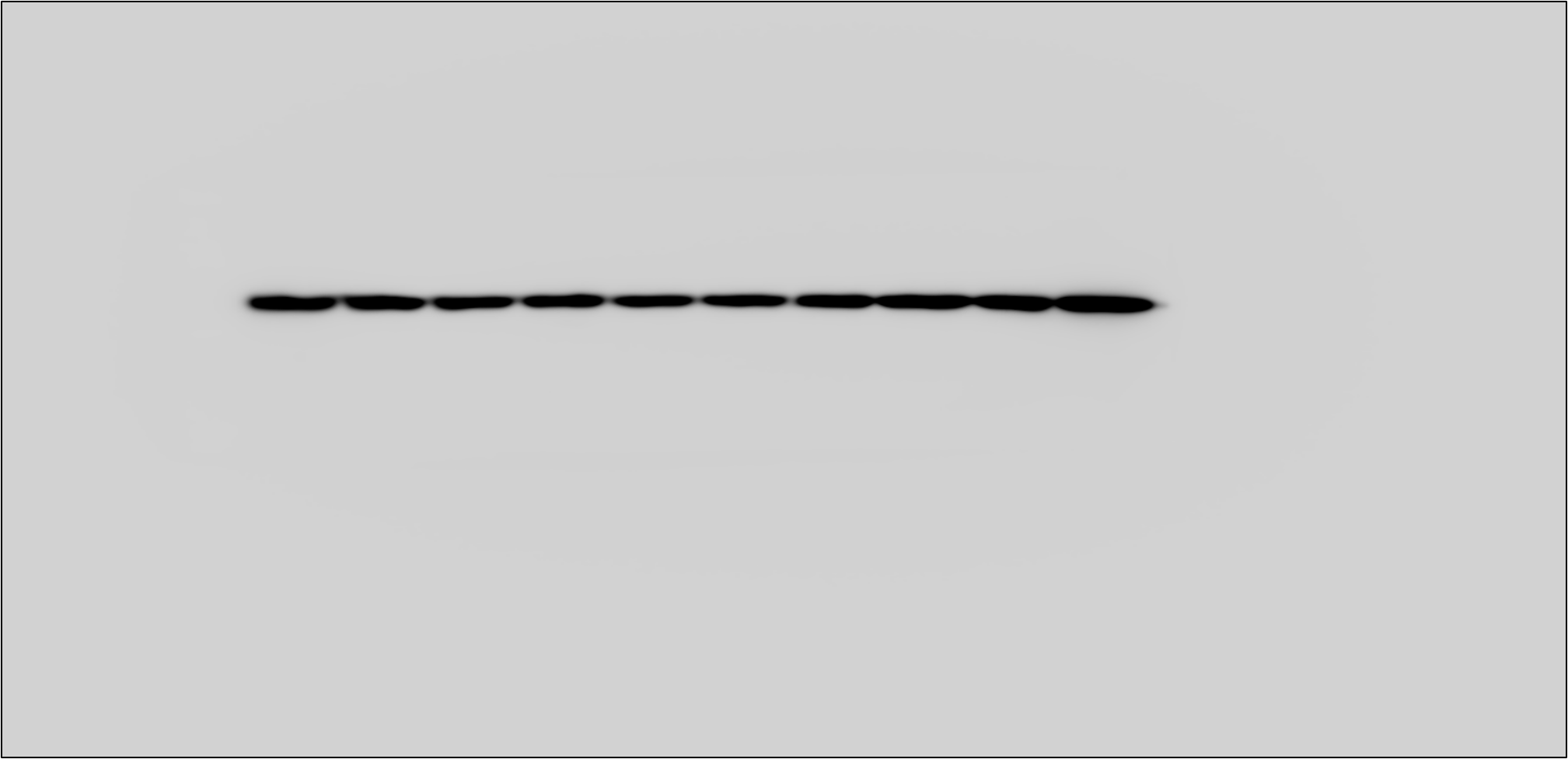

Supplement: Figure 2—source data 2. [file elife-108048-fig2-data2.zip › Figure 2/Figure 2 D-Heart-Actin.tif]

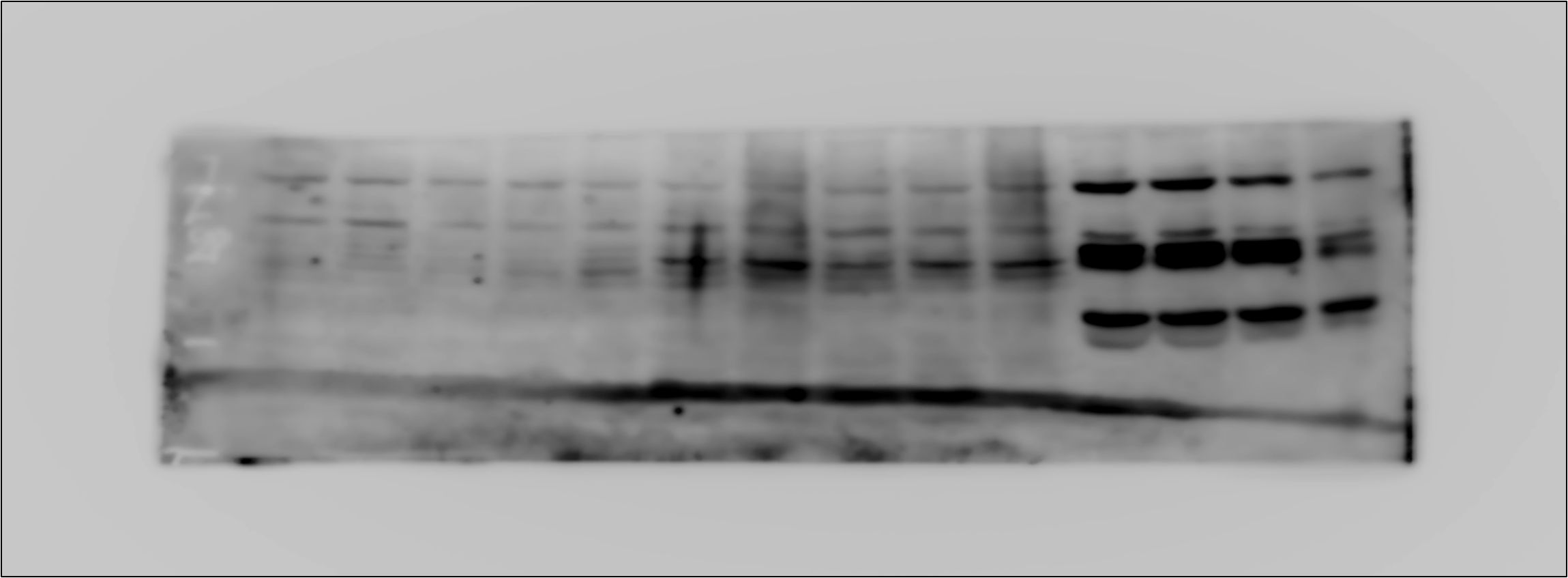

Supplement: Figure 2—source data 2. [file elife-108048-fig2-data2.zip › Figure 2/Figure 2 D-Heart-cyp17a2.tif]

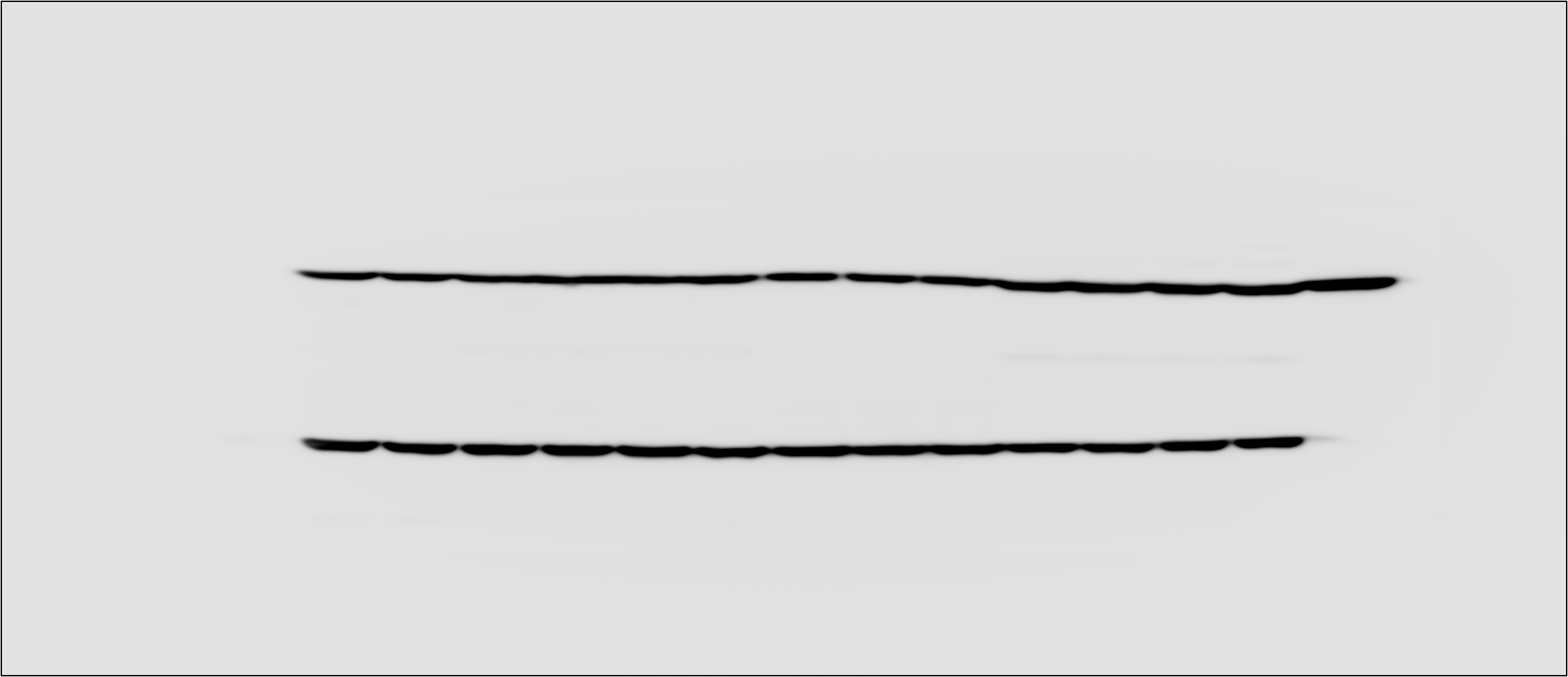

Supplement: Figure 2—source data 2. [file elife-108048-fig2-data2.zip › Figure 2/Figure 2 D-Liver-Actin.tif]

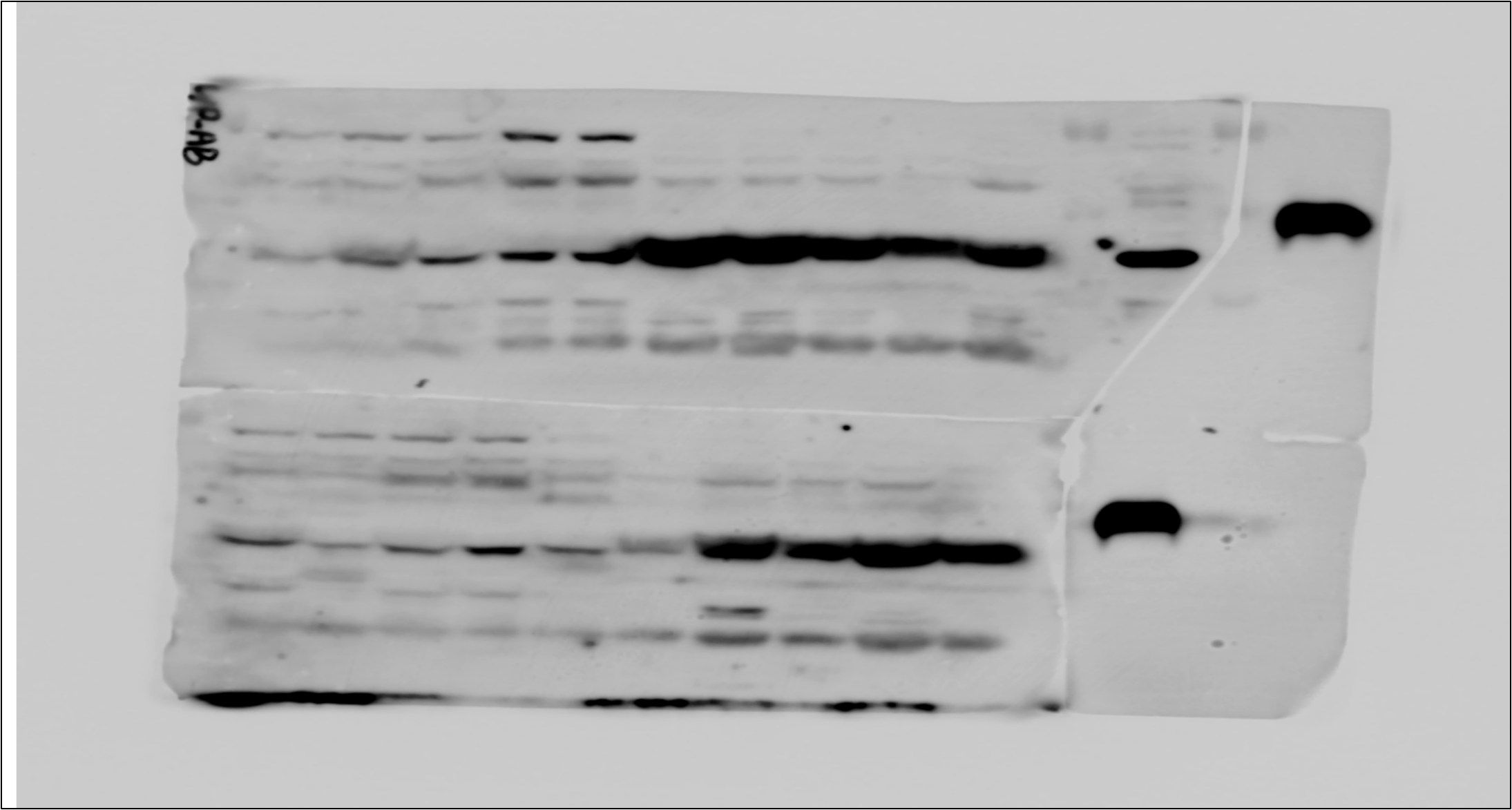

Supplement: Figure 2—source data 2. [file elife-108048-fig2-data2.zip › Figure 2/Figure 2 D-Liver-cyp17a2.tif]

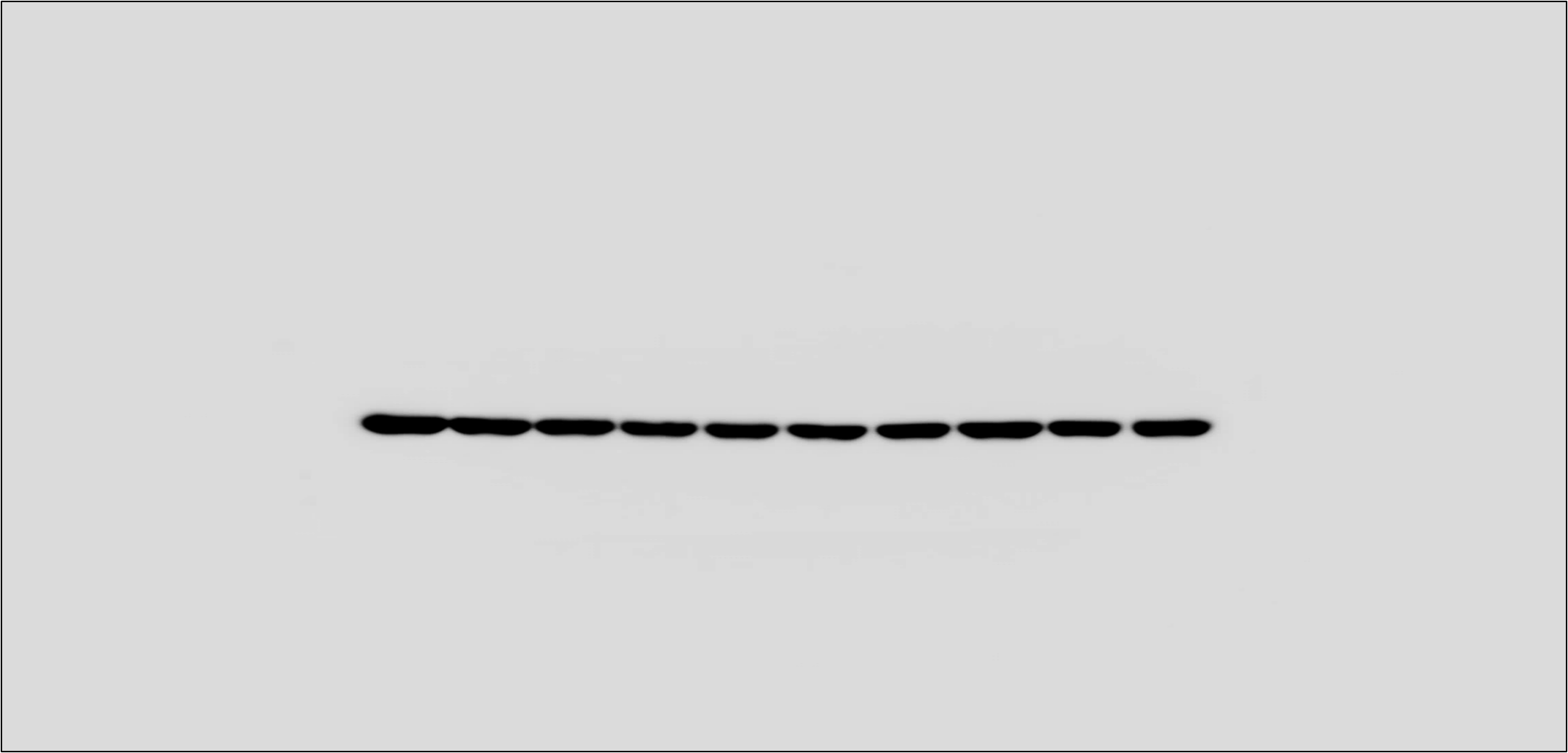

Supplement: Figure 2—source data 2. [file elife-108048-fig2-data2.zip › Figure 2/Figure 2 D-Skin-Actin.tif]

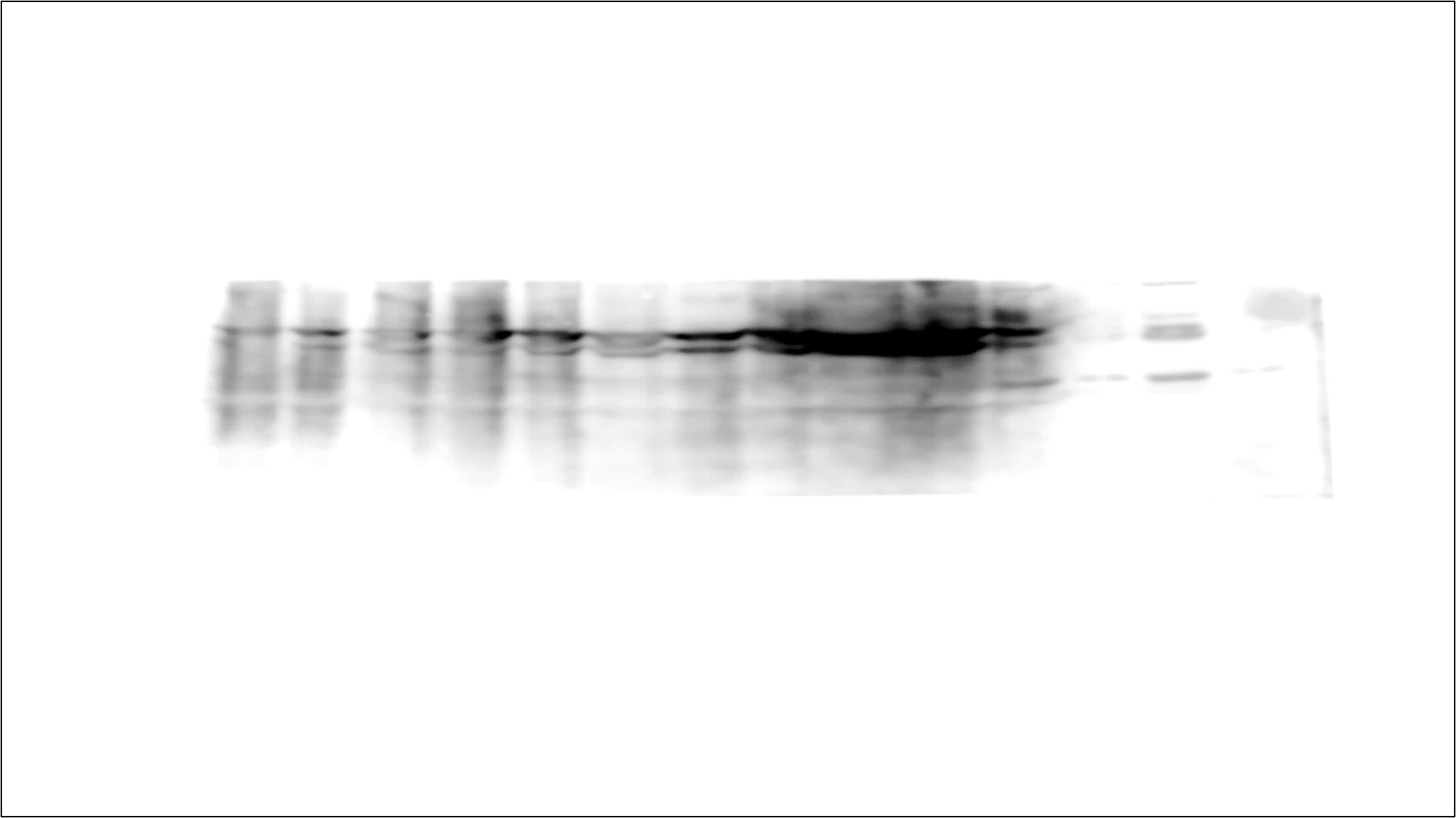

Supplement: Figure 2—source data 2. [file elife-108048-fig2-data2.zip › Figure 2/Figure 2 D-Skin-cyp17a2.tif]

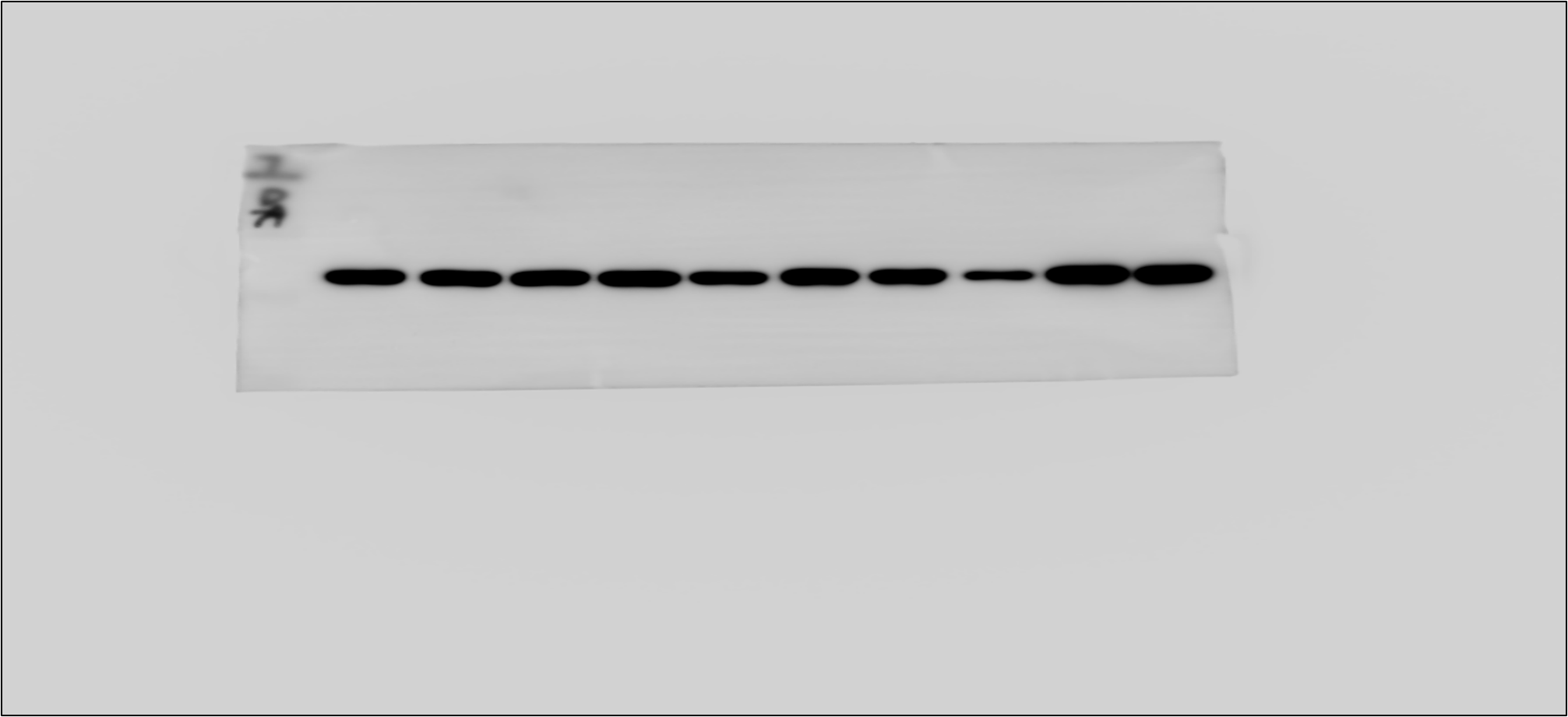

Supplement: Figure 2—source data 2. [file elife-108048-fig2-data2.zip › Figure 2/Figure 2 D-Spleen-Actin.tif]

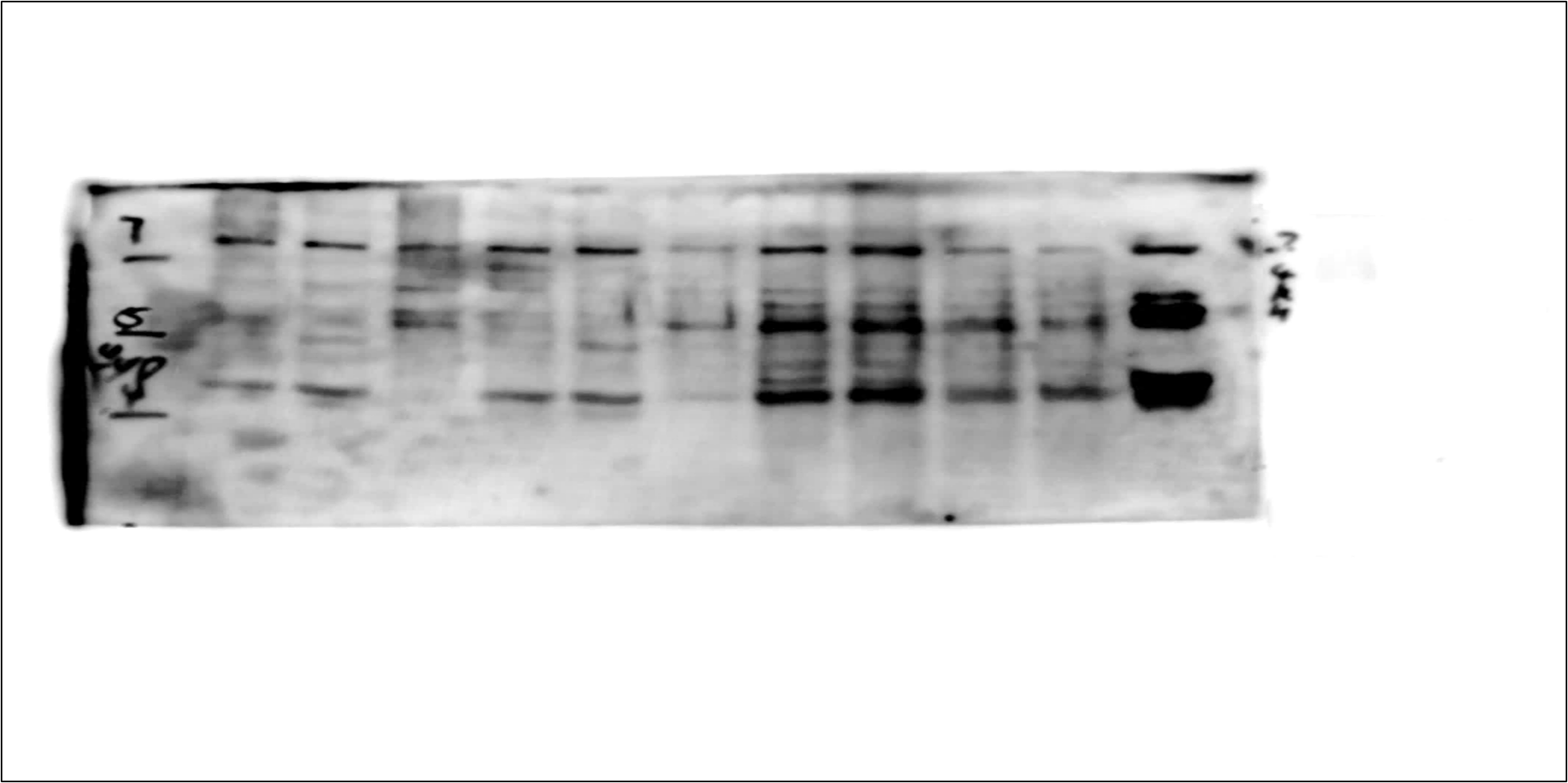

Supplement: Figure 2—source data 2. [file elife-108048-fig2-data2.zip › Figure 2/Figure 2 D-Spleen-cyp17a2.tif]

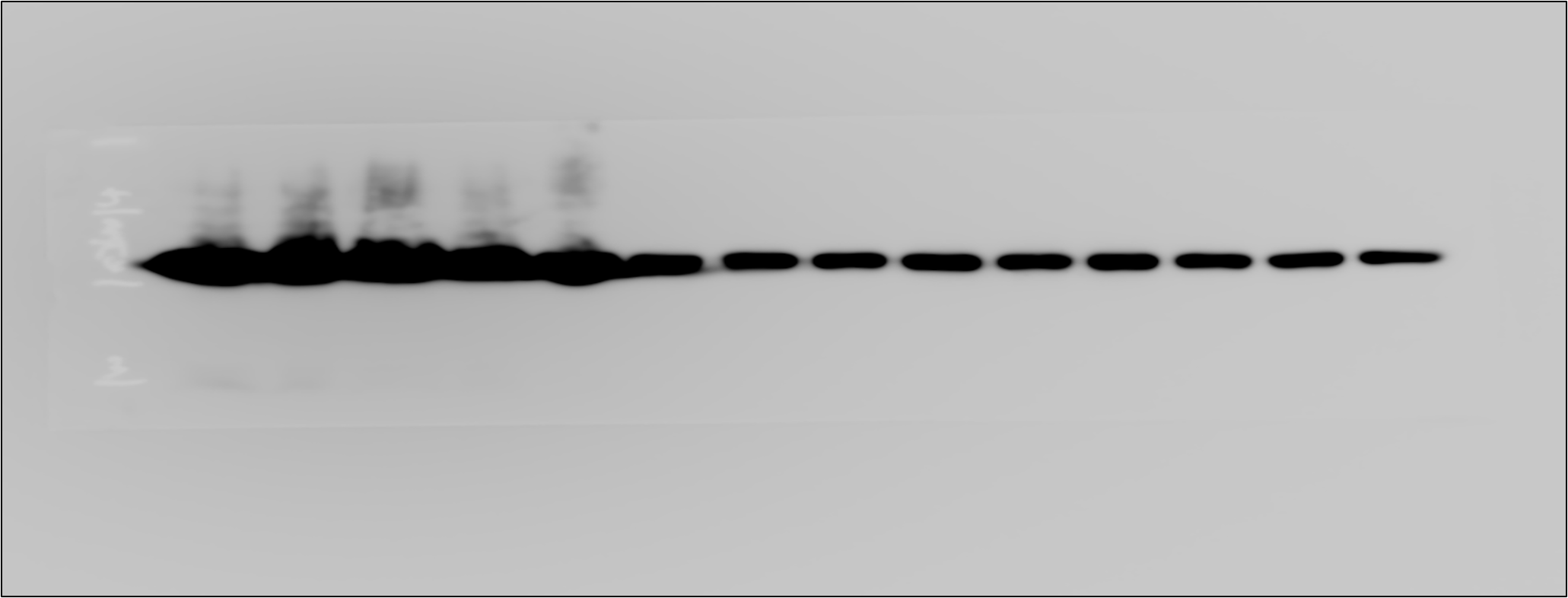

Supplement: Figure 2—figure supplement 1—source data 2. [file elife-108048-fig2-figsupp1-data2.zip › Figure 2-figure supplement 1/Figure S1C-EPC-Actin.tif]

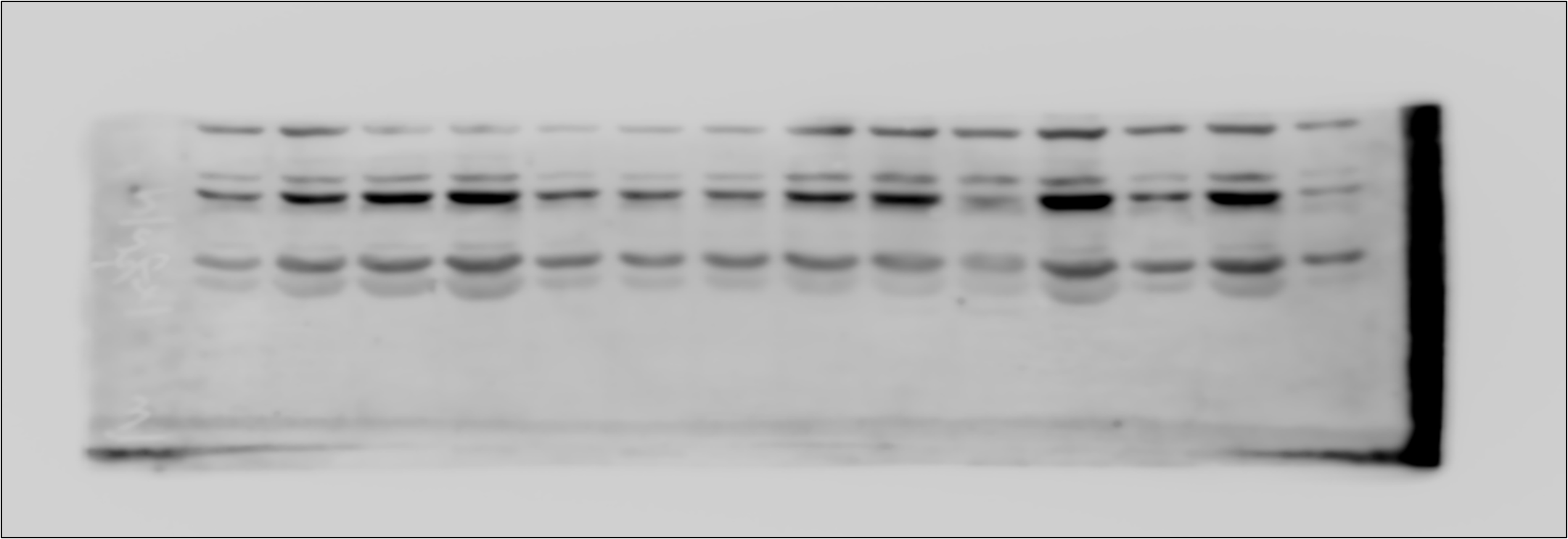

Supplement: Figure 2—figure supplement 1—source data 2. [file elife-108048-fig2-figsupp1-data2.zip › Figure 2-figure supplement 1/Figure S1C-EPC-cyp17a2.tif]

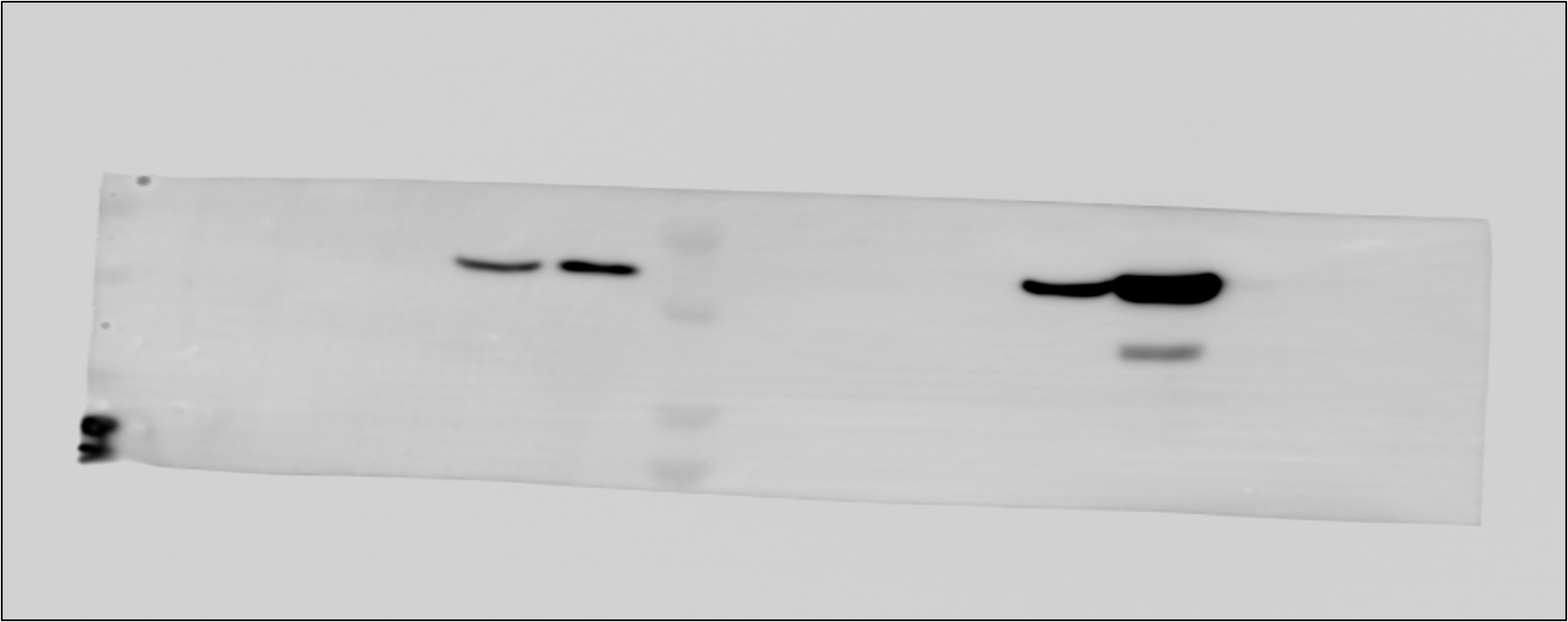

Supplement: Figure 2—figure supplement 1—source data 2. [file elife-108048-fig2-figsupp1-data2.zip › Figure 2-figure supplement 1/Figure S1C-EPC-N.tif]

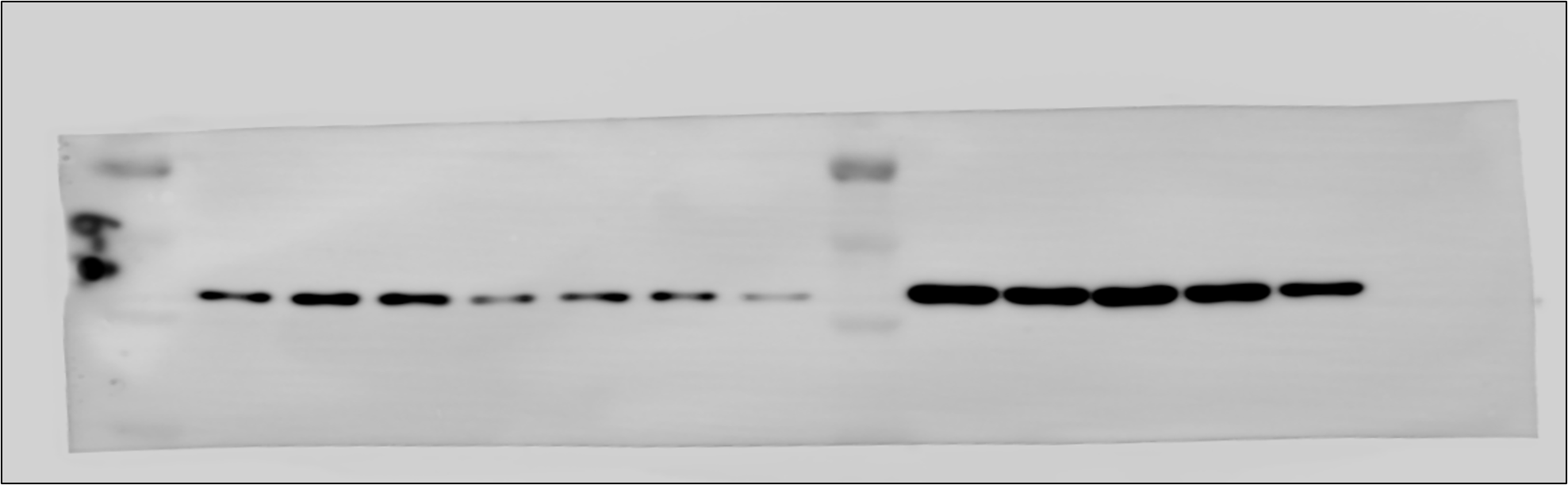

Supplement: Figure 2—figure supplement 1—source data 2. [file elife-108048-fig2-figsupp1-data2.zip › Figure 2-figure supplement 1/Figure S1C-ZF4-Actin.tif]

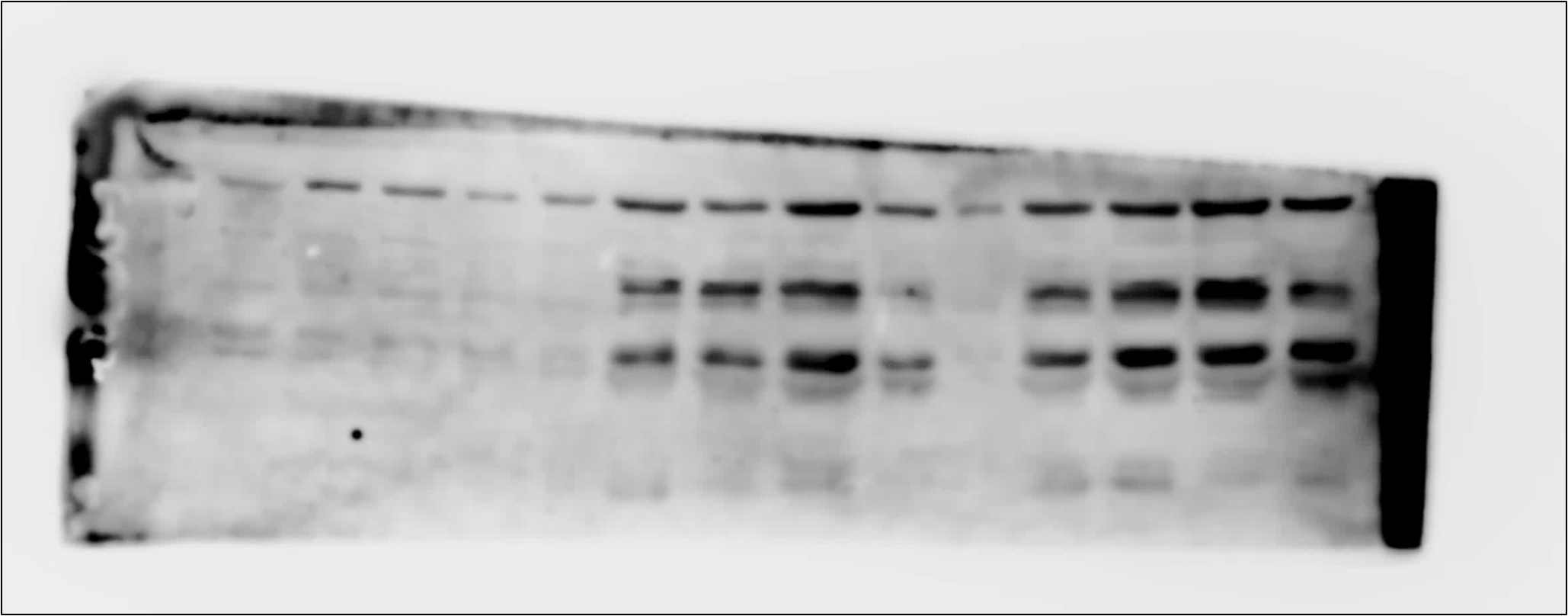

Supplement: Figure 2—figure supplement 1—source data 2. [file elife-108048-fig2-figsupp1-data2.zip › Figure 2-figure supplement 1/Figure S1C-ZF4-cyp17a2.tif]

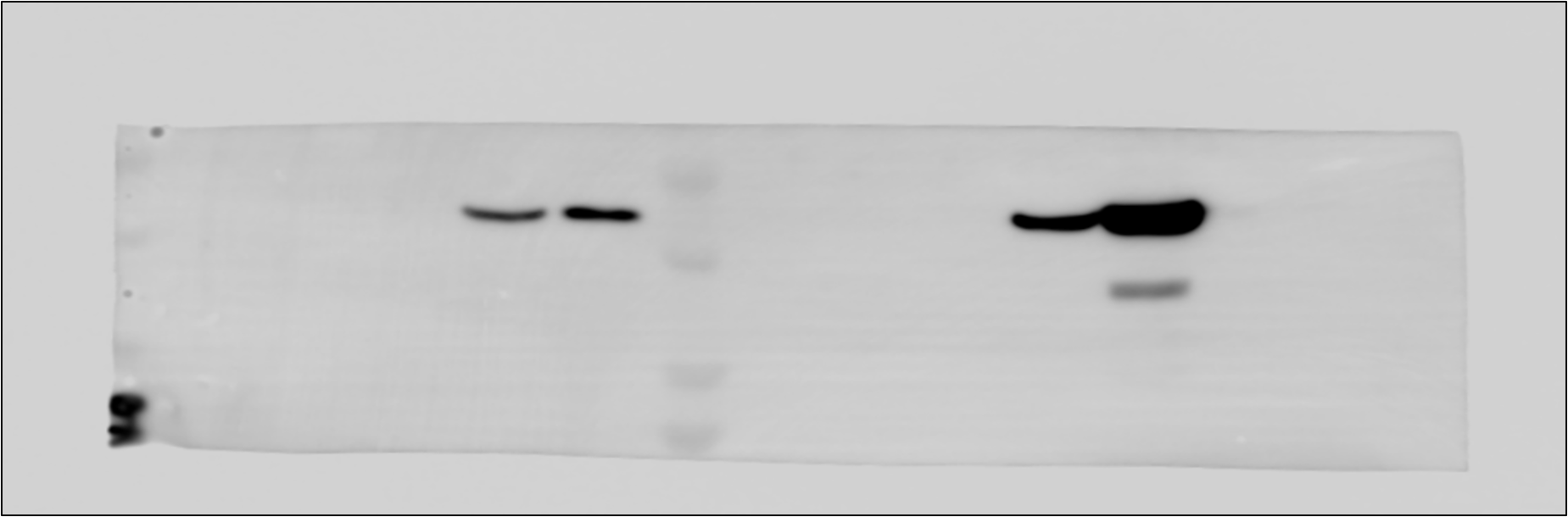

Supplement: Figure 2—figure supplement 1—source data 2. [file elife-108048-fig2-figsupp1-data2.zip › Figure 2-figure supplement 1/Figure S1C-ZF4-N.tif]

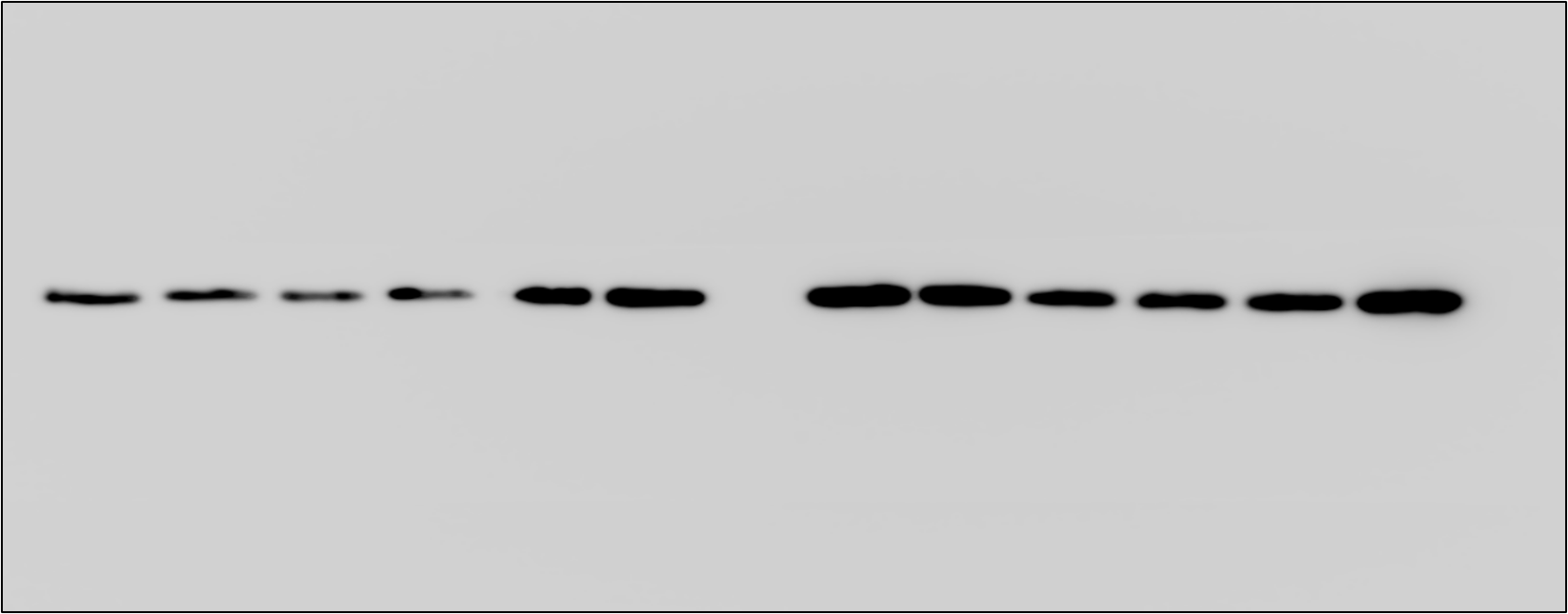

Supplement: Figure 2—figure supplement 1—source data 2. [file elife-108048-fig2-figsupp1-data2.zip › Figure 2-figure supplement 1/Figure S1G-Liver-Actin.tif]

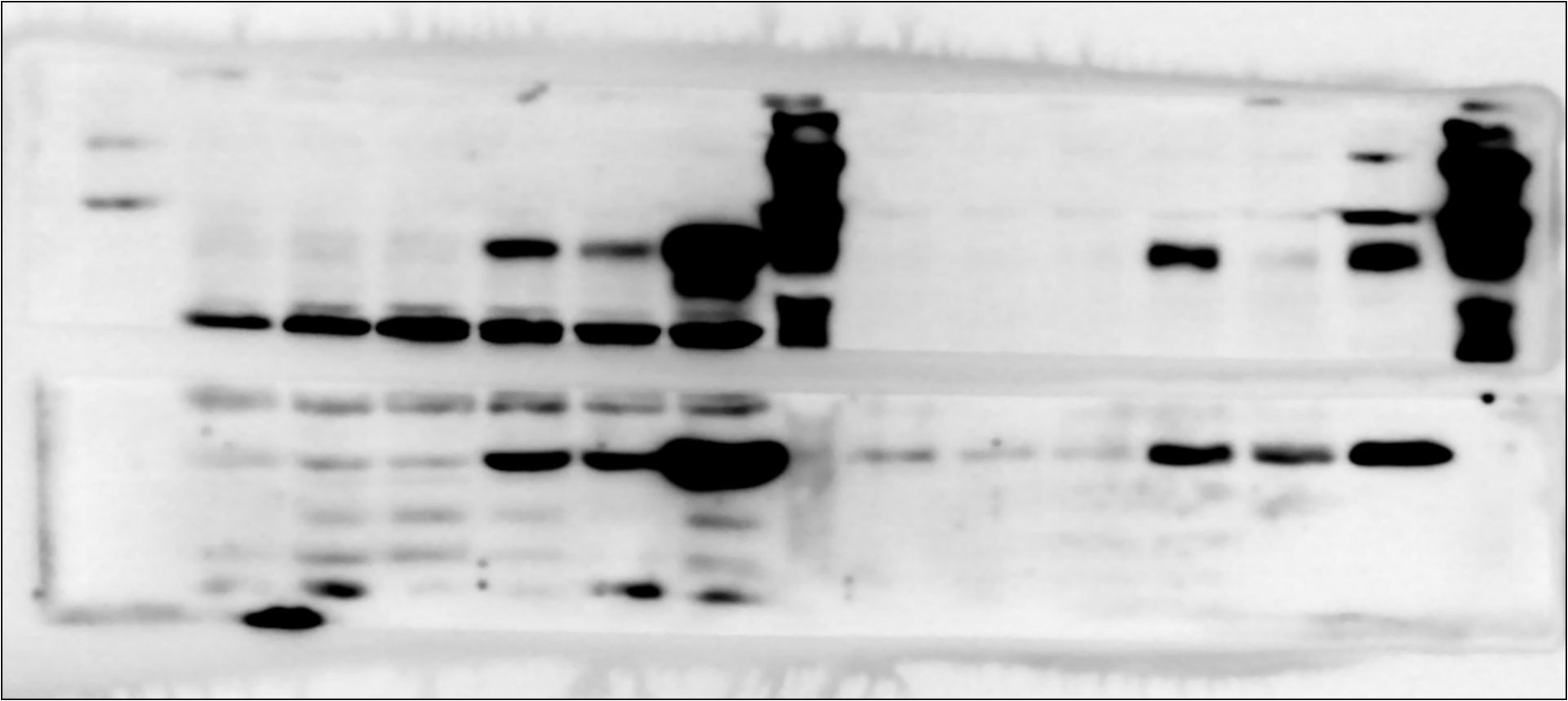

Supplement: Figure 2—figure supplement 1—source data 2. [file elife-108048-fig2-figsupp1-data2.zip › Figure 2-figure supplement 1/Figure S1G-liver-G.tif]

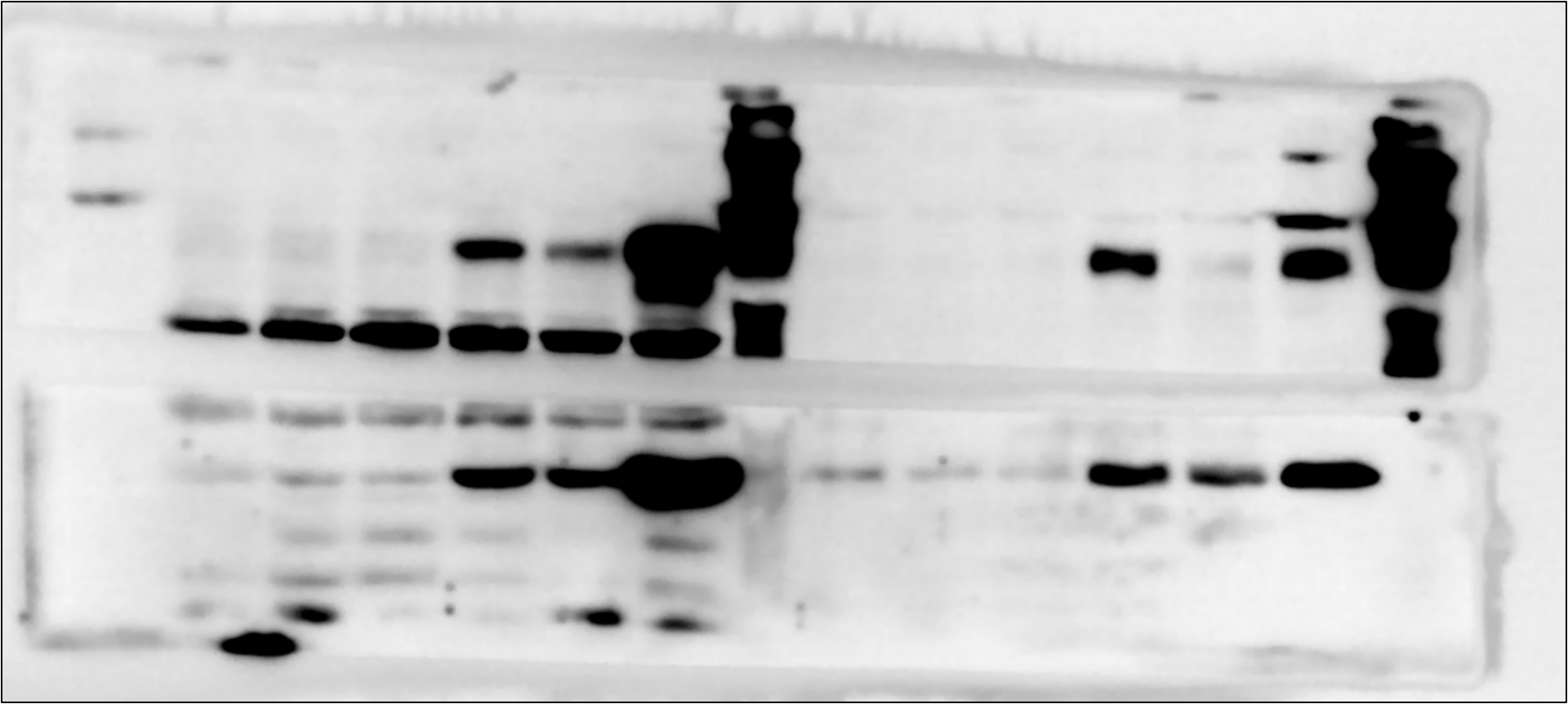

Supplement: Figure 2—figure supplement 1—source data 2. [file elife-108048-fig2-figsupp1-data2.zip › Figure 2-figure supplement 1/Figure S1G-liver-N.tif]

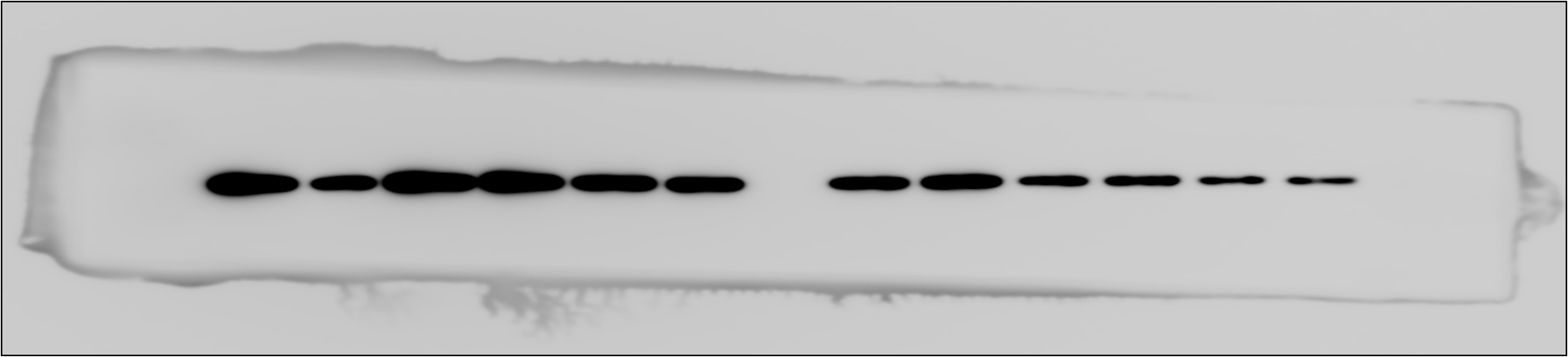

Supplement: Figure 2—figure supplement 1—source data 2. [file elife-108048-fig2-figsupp1-data2.zip › Figure 2-figure supplement 1/Figure S1G-spleen-Actin.tif]

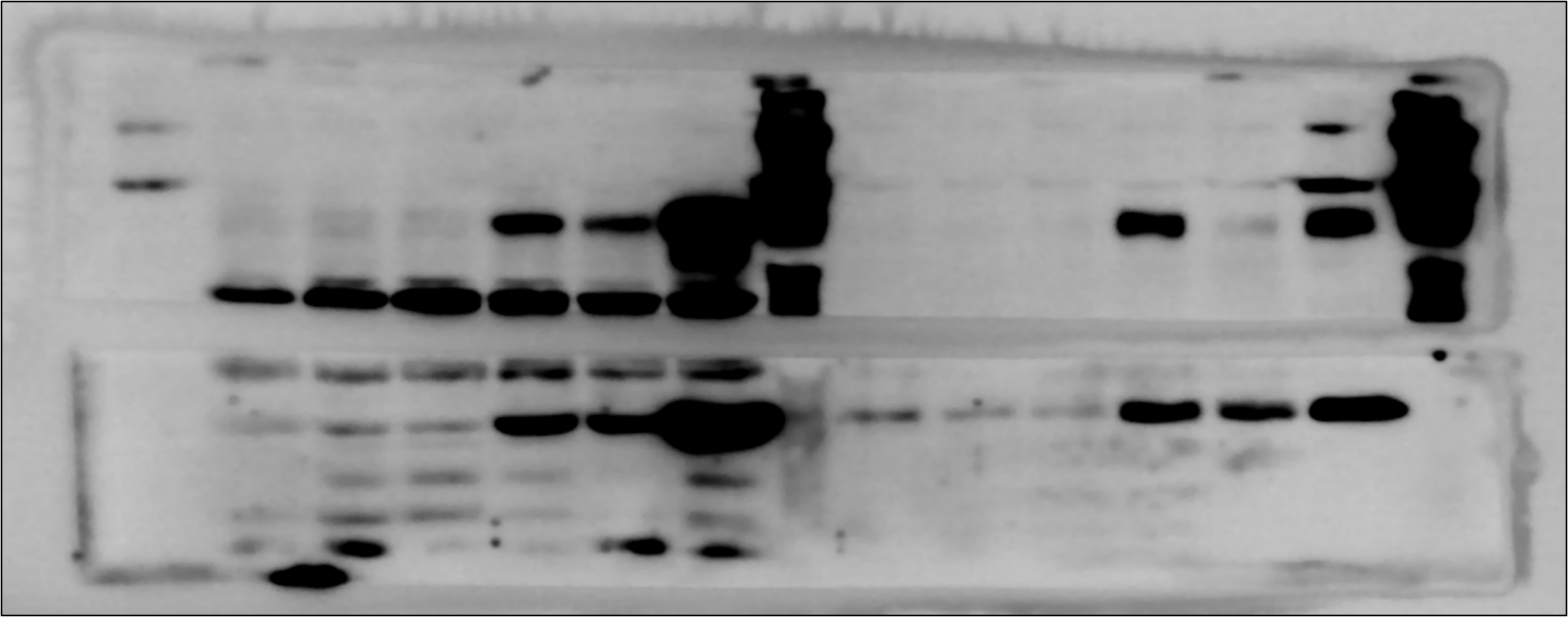

Supplement: Figure 2—figure supplement 1—source data 2. [file elife-108048-fig2-figsupp1-data2.zip › Figure 2-figure supplement 1/Figure S1G-spleen-G.tif]

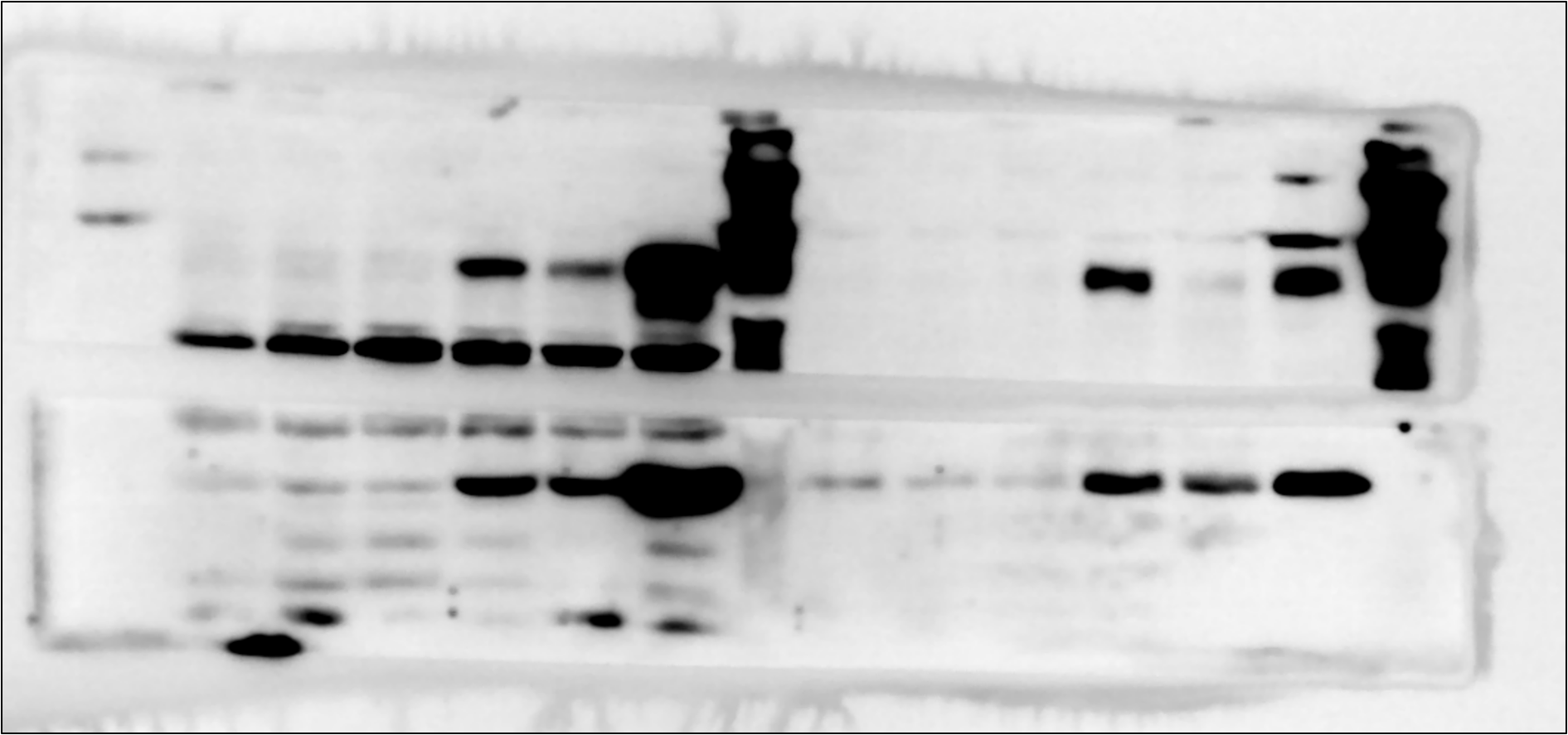

Supplement: Figure 2—figure supplement 1—source data 2. [file elife-108048-fig2-figsupp1-data2.zip › Figure 2-figure supplement 1/Figure S1G-spleen-N.tif]

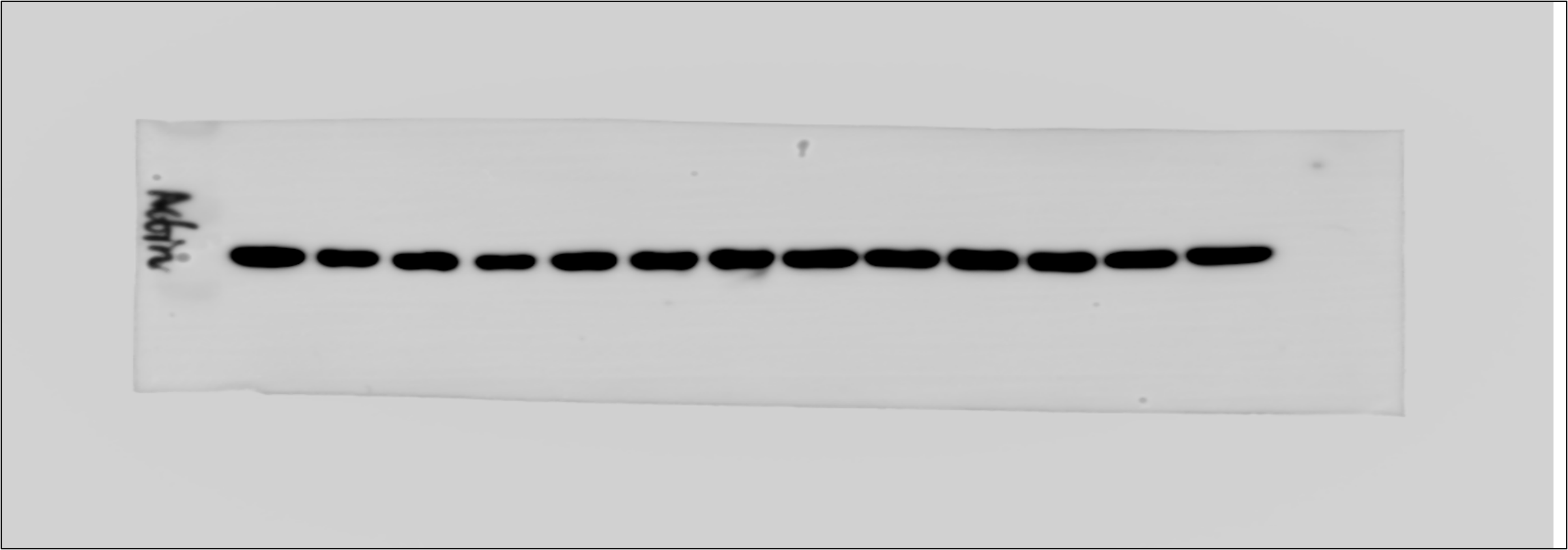

Supplement: Figure 3—source data 2. [file elife-108048-fig3-data2.zip › Figure 3/Figure 3C-Actin.tif]

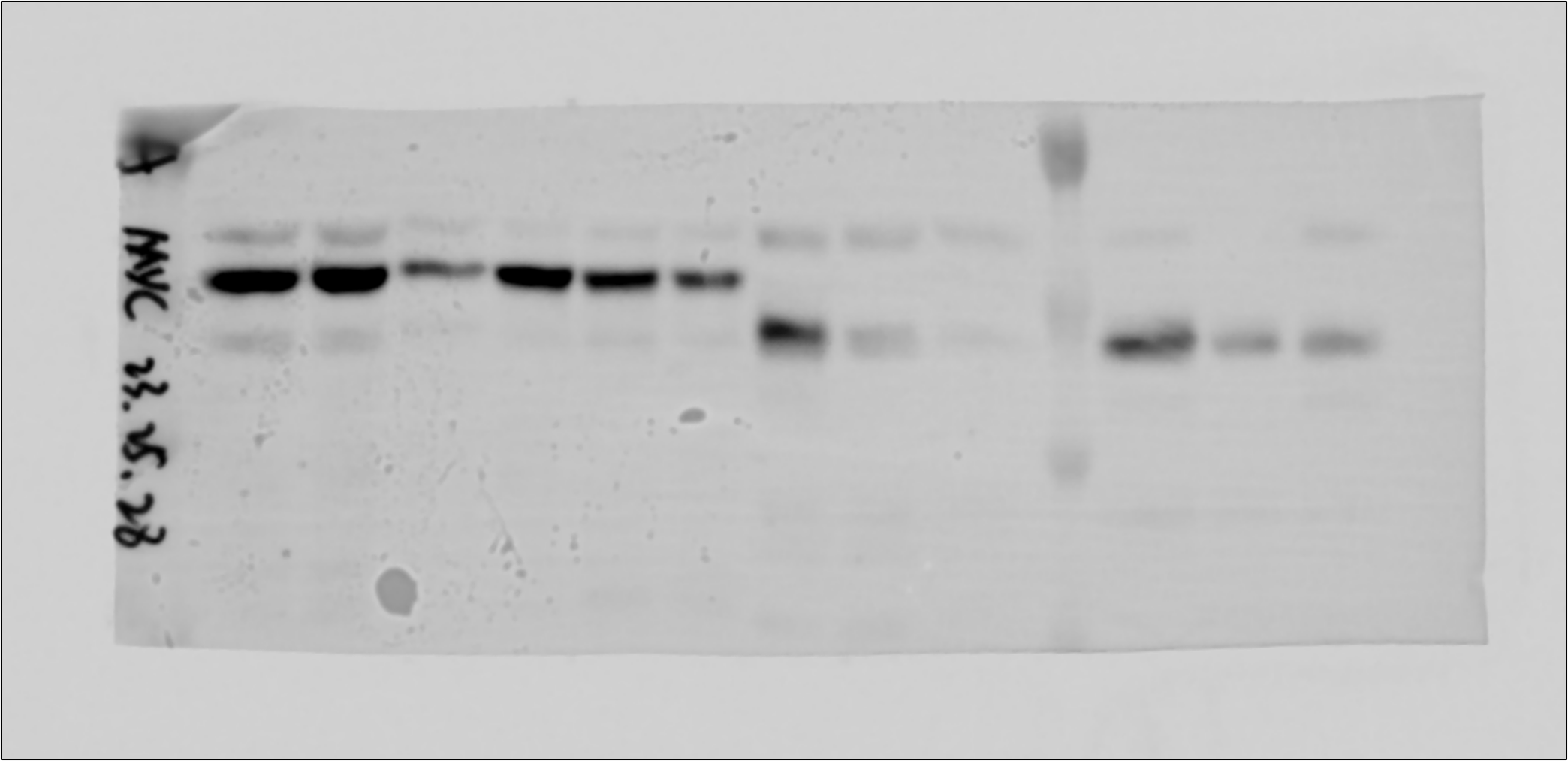

Supplement: Figure 3—source data 2. [file elife-108048-fig3-data2.zip › Figure 3/Figure 3C-Myc.tif]

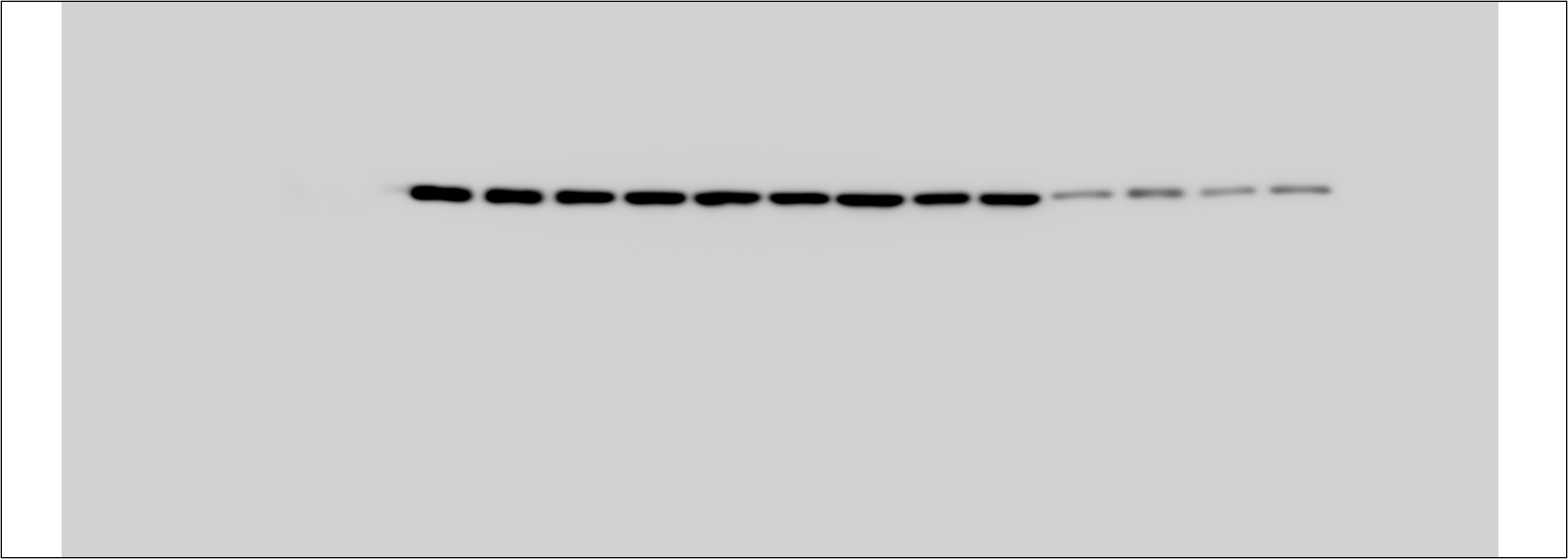

Supplement: Figure 3—source data 2. [file elife-108048-fig3-data2.zip › Figure 3/Figure 3D-Actin.tif]

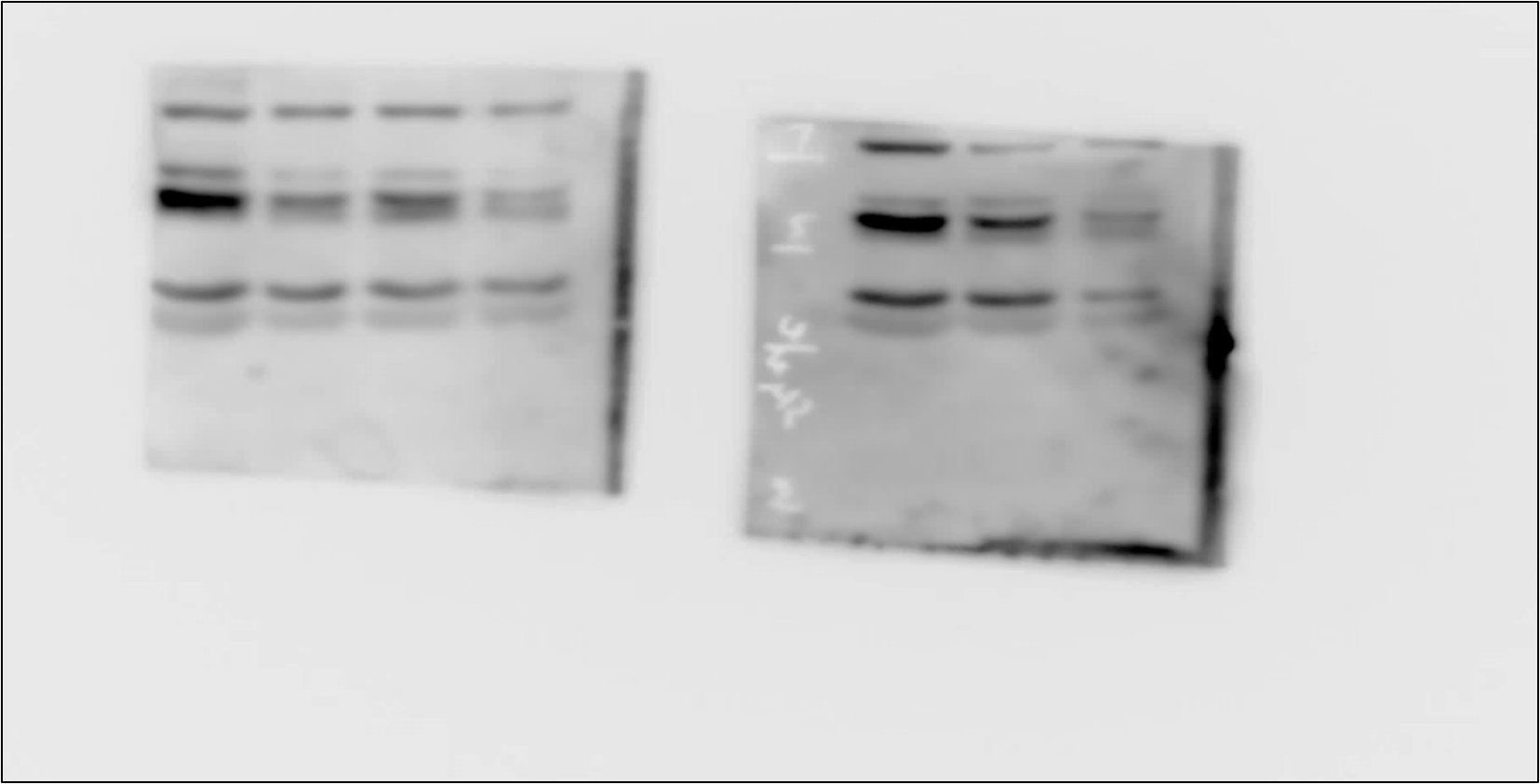

Supplement: Figure 3—source data 2. [file elife-108048-fig3-data2.zip › Figure 3/Figure 3D-Myc.tif]

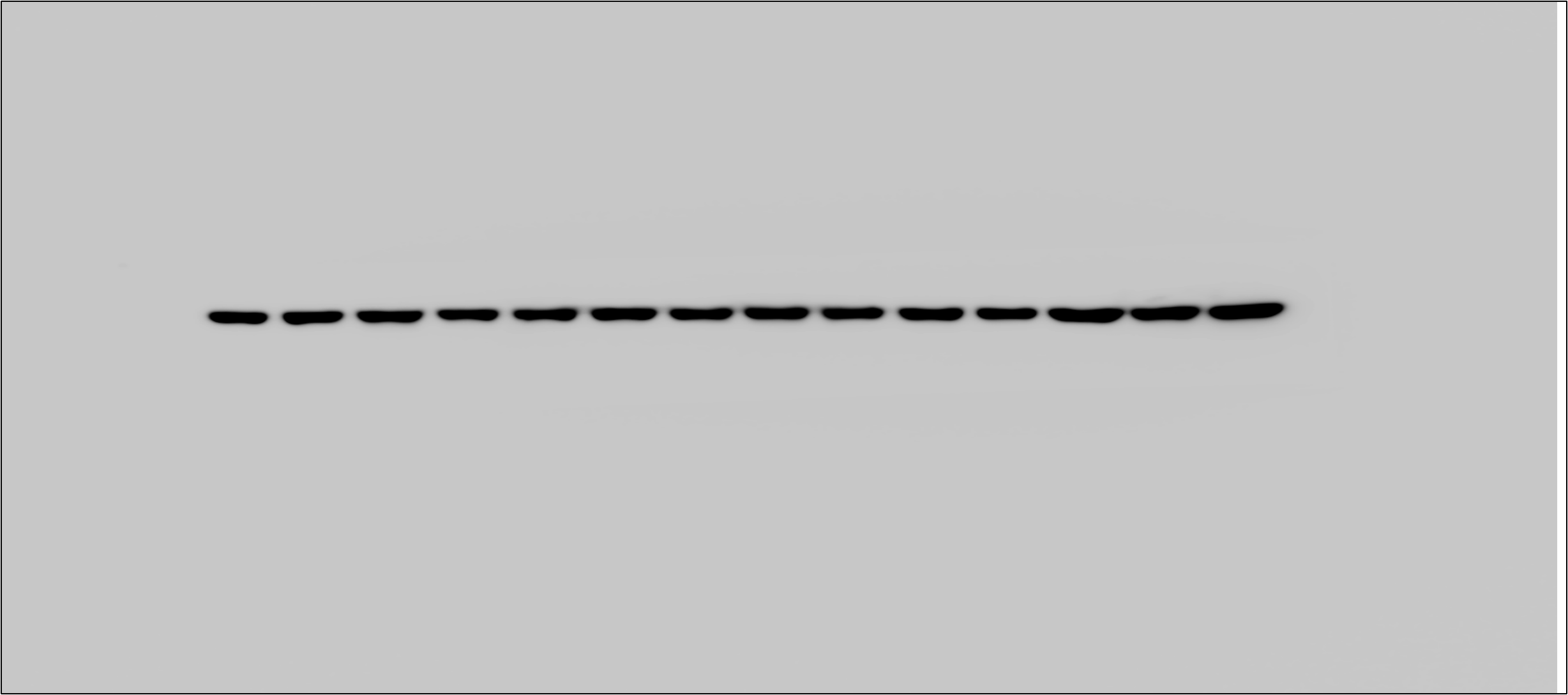

Supplement: Figure 3—source data 2. [file elife-108048-fig3-data2.zip › Figure 3/Figure 3J-Actin.tif]

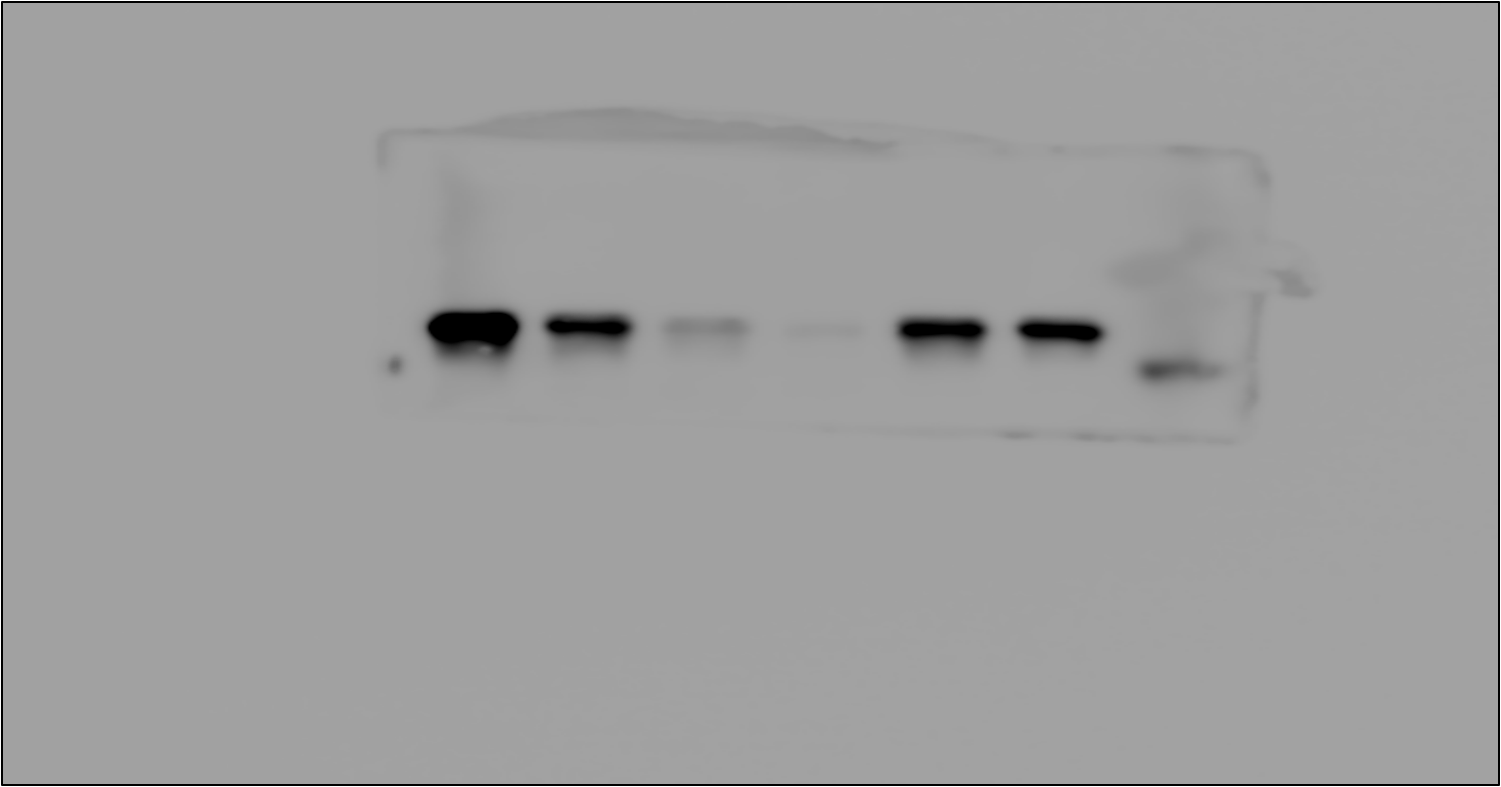

Supplement: Figure 3—source data 2. [file elife-108048-fig3-data2.zip › Figure 3/Figure 3J-G.tif]

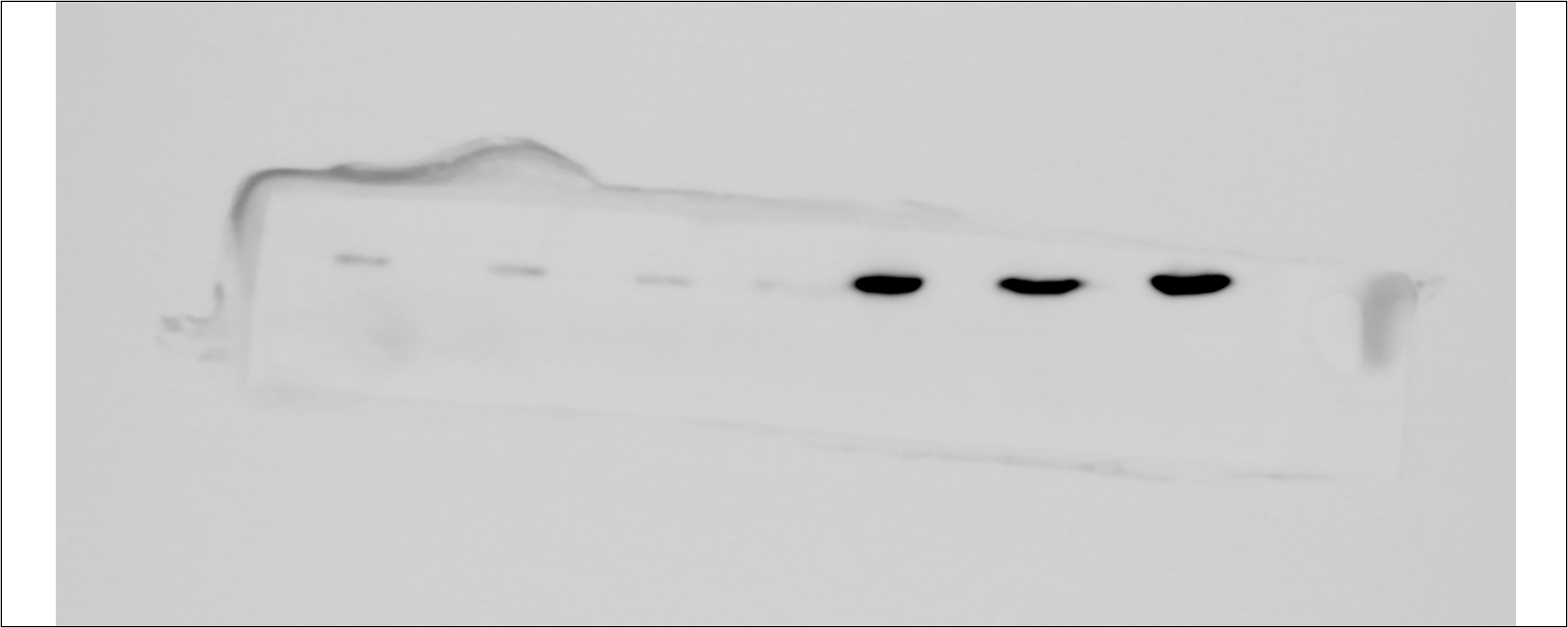

Supplement: Figure 3—source data 2. [file elife-108048-fig3-data2.zip › Figure 3/Figure 3J-HA.tif]

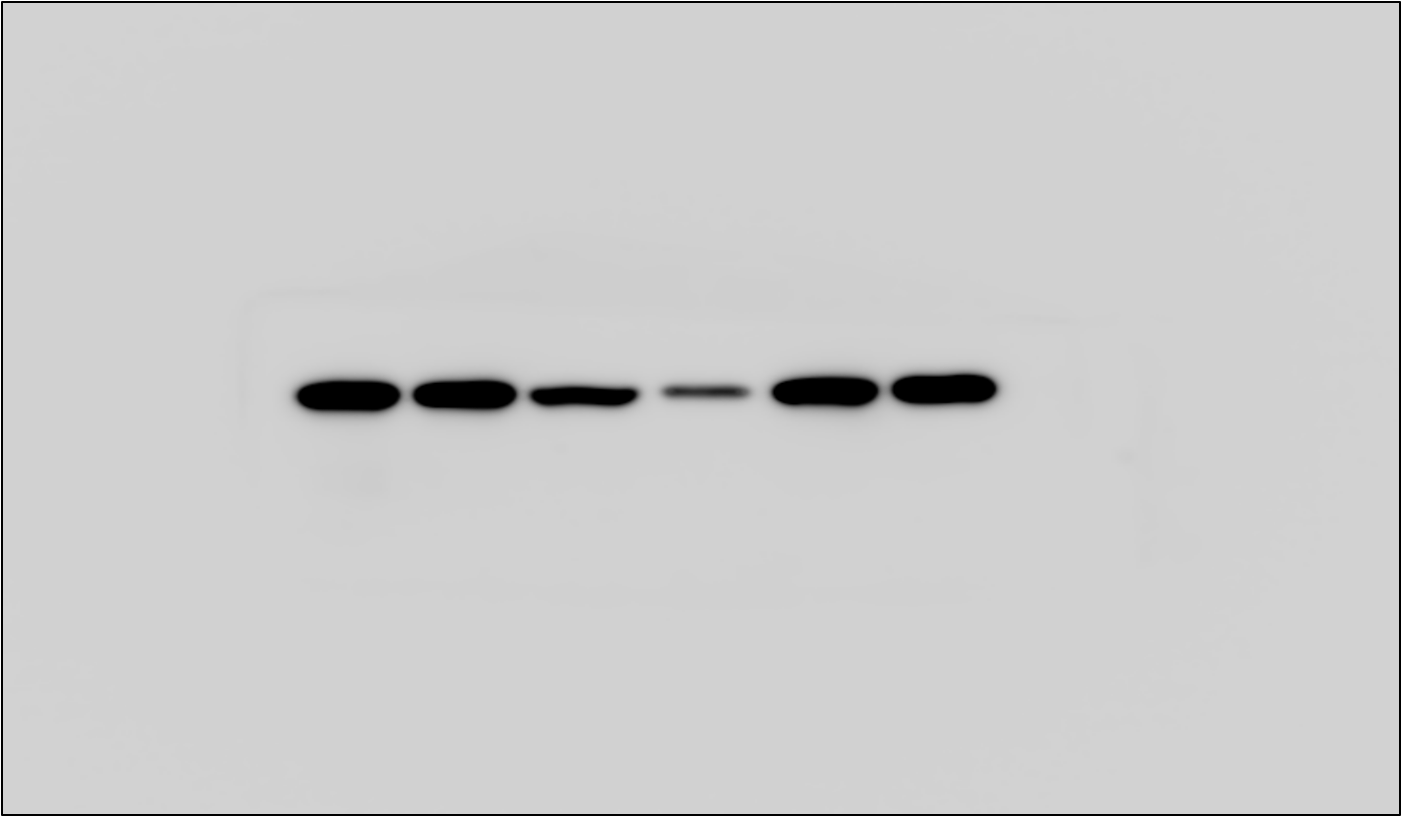

Supplement: Figure 3—source data 2. [file elife-108048-fig3-data2.zip › Figure 3/Figure 3J-N.tif]

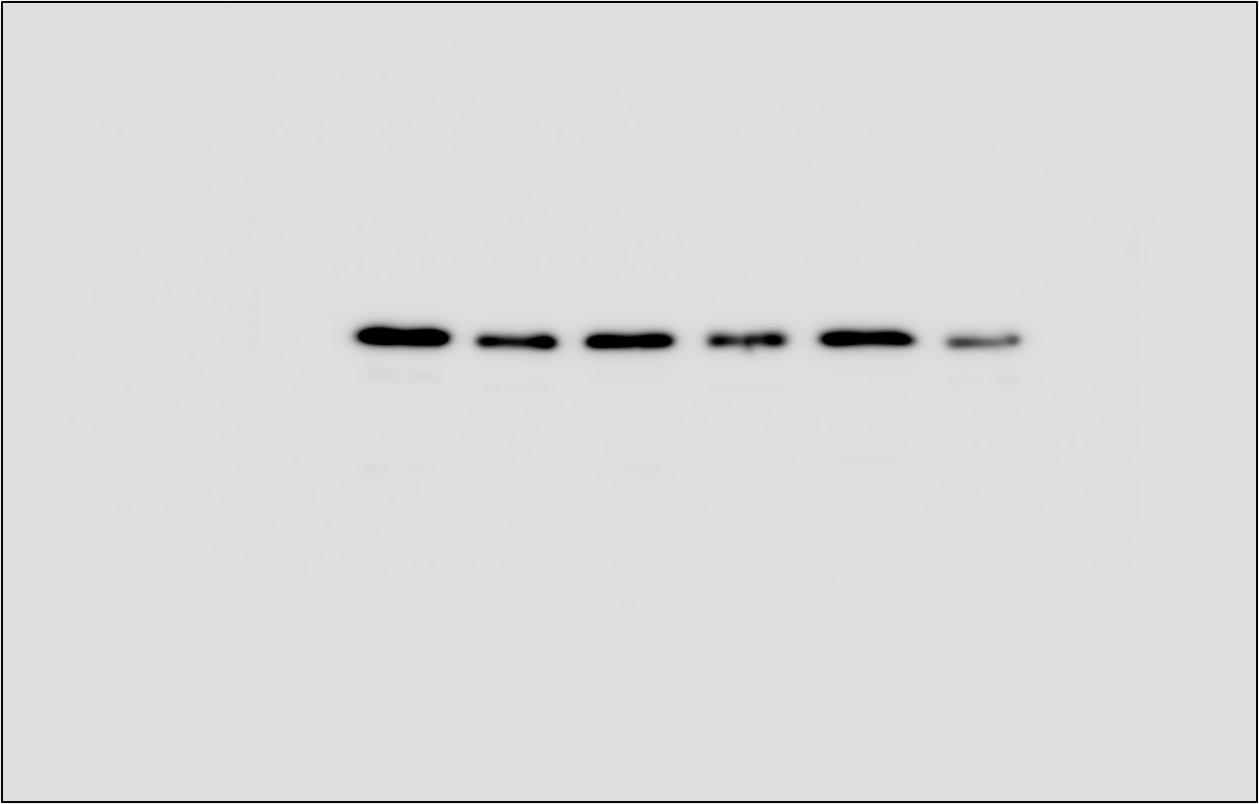

Supplement: Figure 3—source data 2. [file elife-108048-fig3-data2.zip › Figure 3/Figure 3J-P.tif]

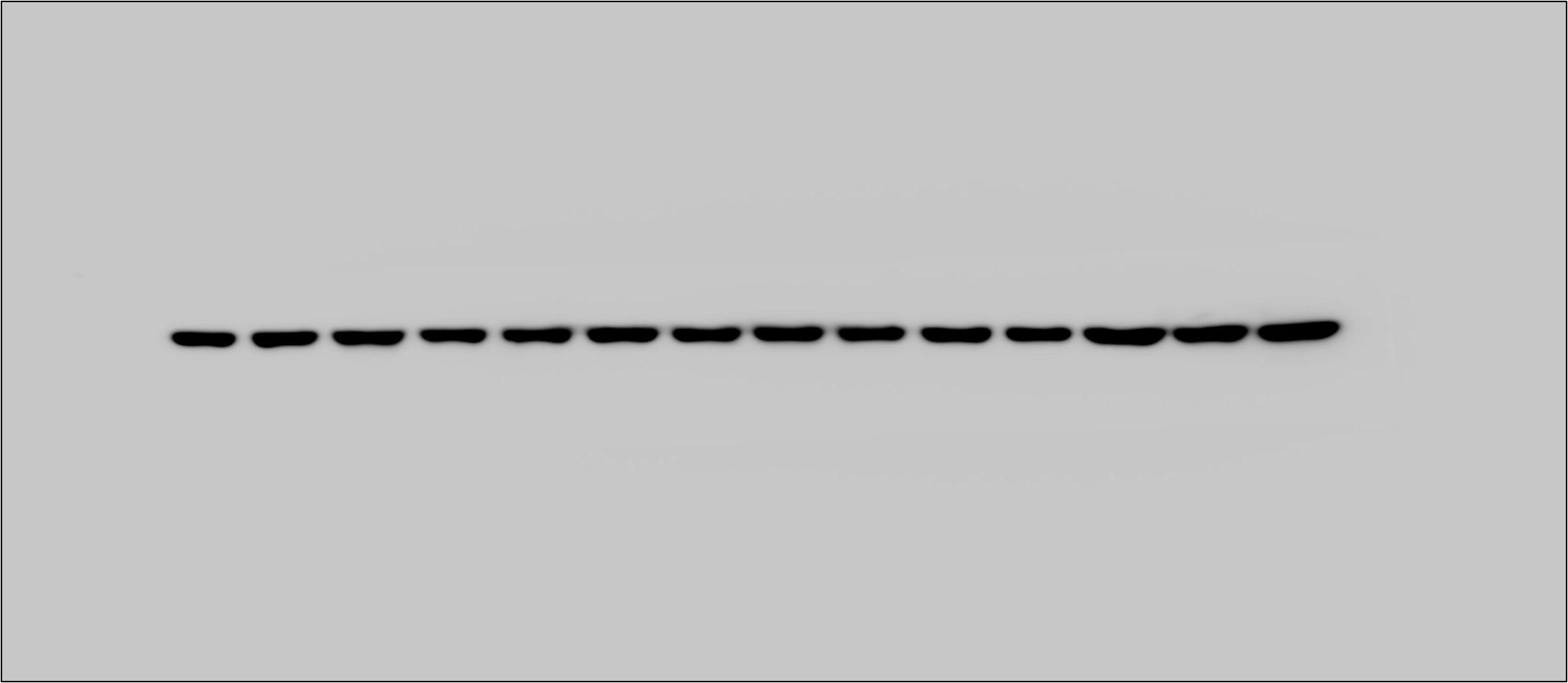

Supplement: Figure 3—figure supplement 1—source data 2. [file elife-108048-fig3-figsupp1-data2.zip › Figure 3-figure supplement 1/Figure S3 H-Actin.tif]

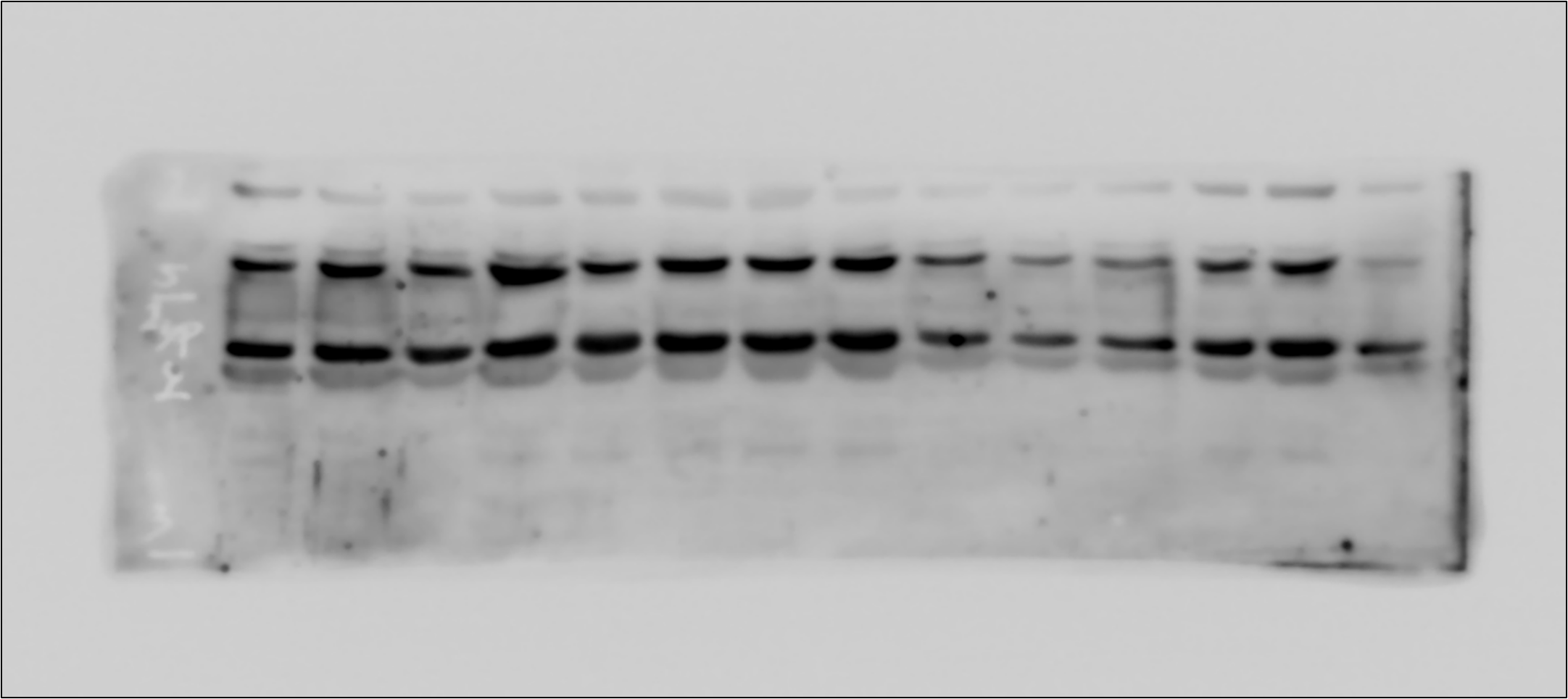

Supplement: Figure 3—figure supplement 1—source data 2. [file elife-108048-fig3-figsupp1-data2.zip › Figure 3-figure supplement 1/Figure S3 H-cyp17a2.tif]

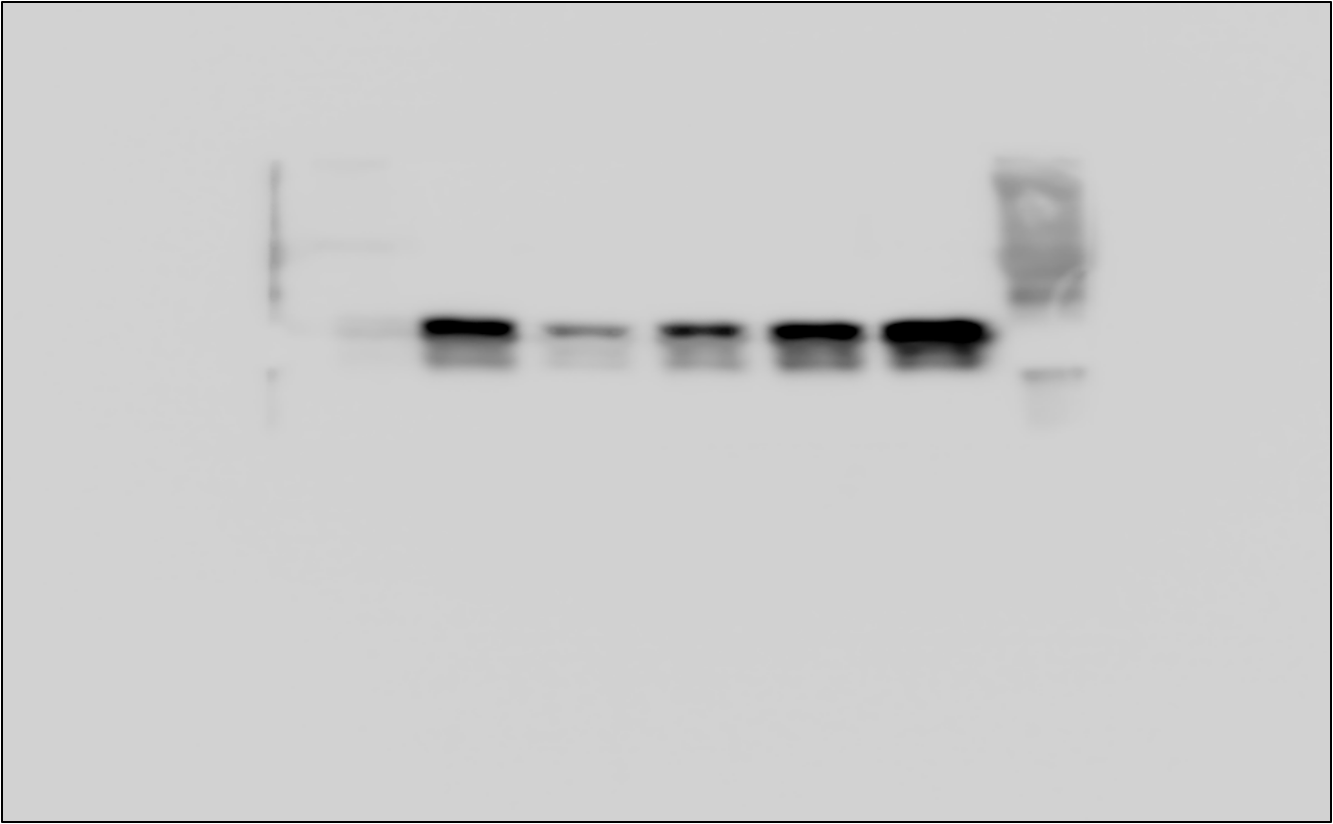

Supplement: Figure 3—figure supplement 1—source data 2. [file elife-108048-fig3-figsupp1-data2.zip › Figure 3-figure supplement 1/Figure S3 H-G.tif]

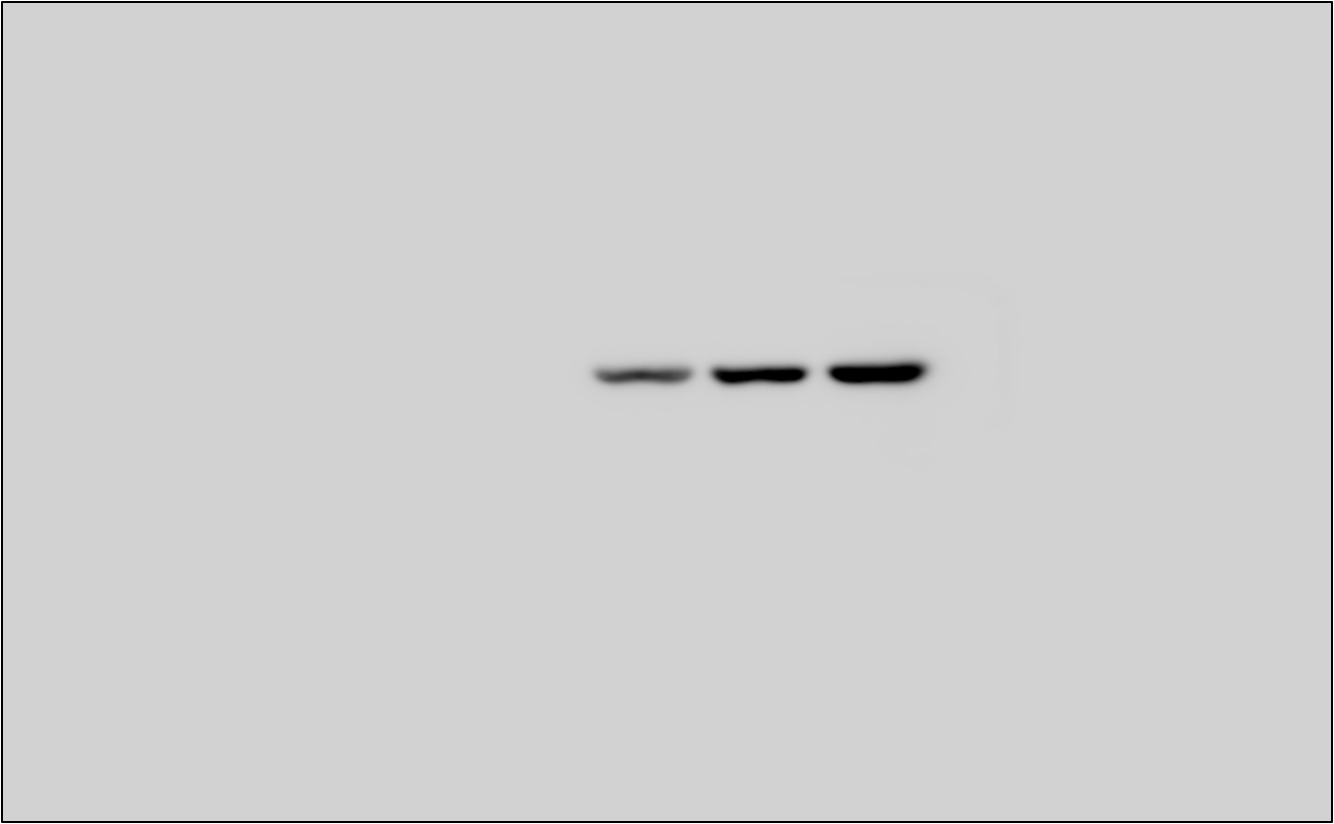

Supplement: Figure 3—figure supplement 1—source data 2. [file elife-108048-fig3-figsupp1-data2.zip › Figure 3-figure supplement 1/Figure S3 H-N.tif]

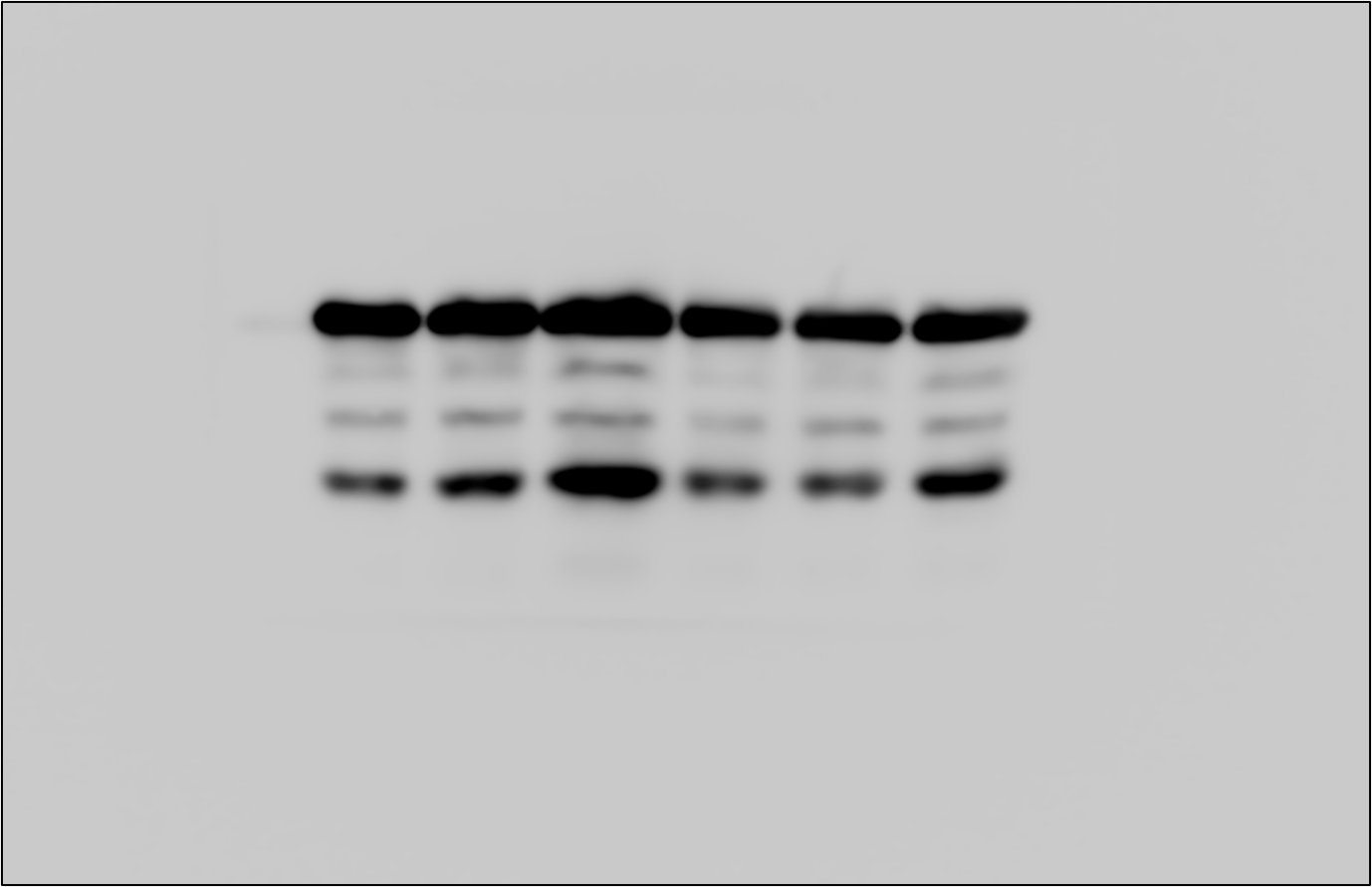

Supplement: Figure 3—figure supplement 1—source data 2. [file elife-108048-fig3-figsupp1-data2.zip › Figure 3-figure supplement 1/Figure S3 H-P.tif]

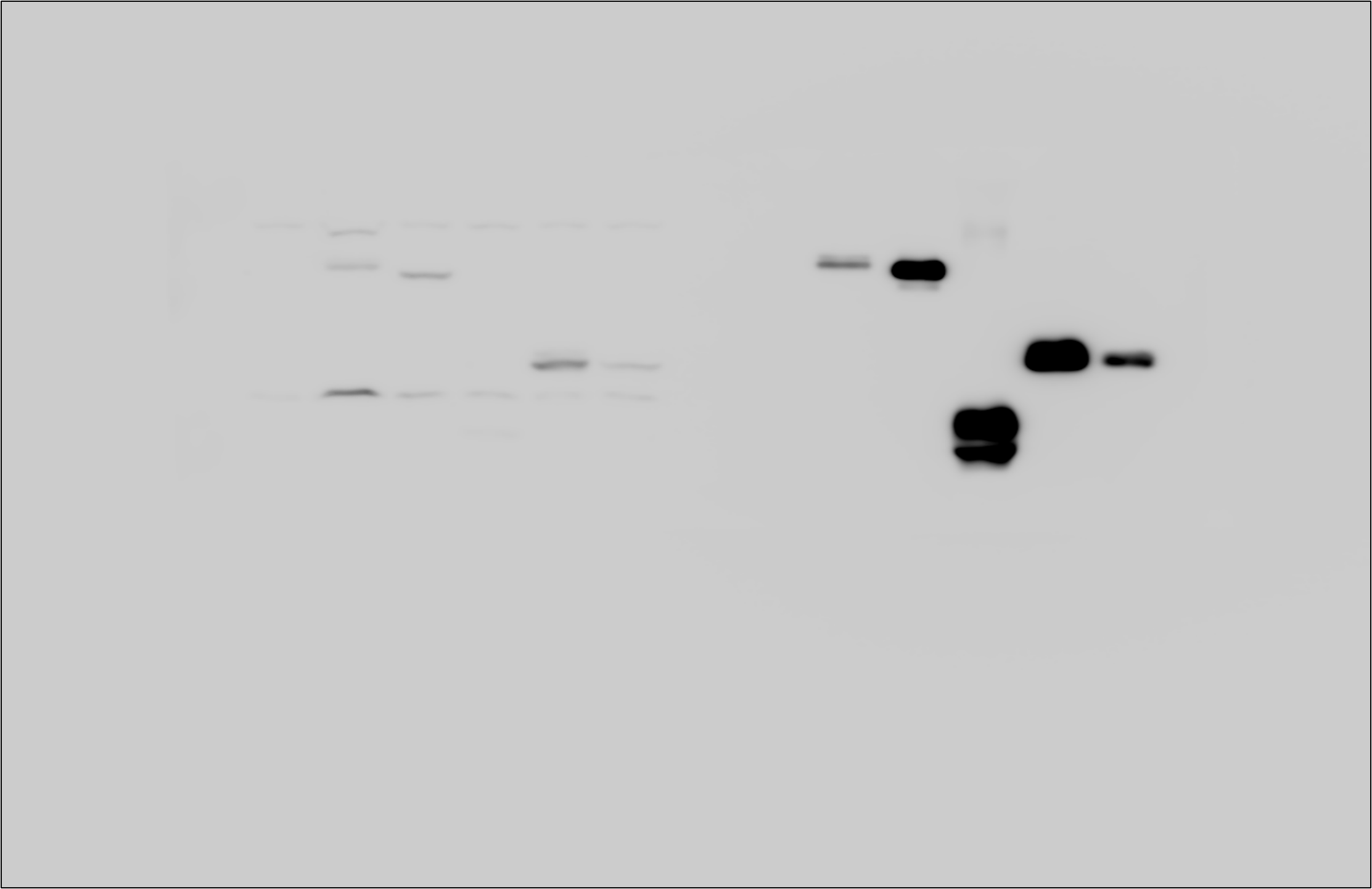

Supplement: Figure 4—source data 2. [file elife-108048-fig4-data2.zip › Figure 4/Figure 4 A IP-Flag.tif]

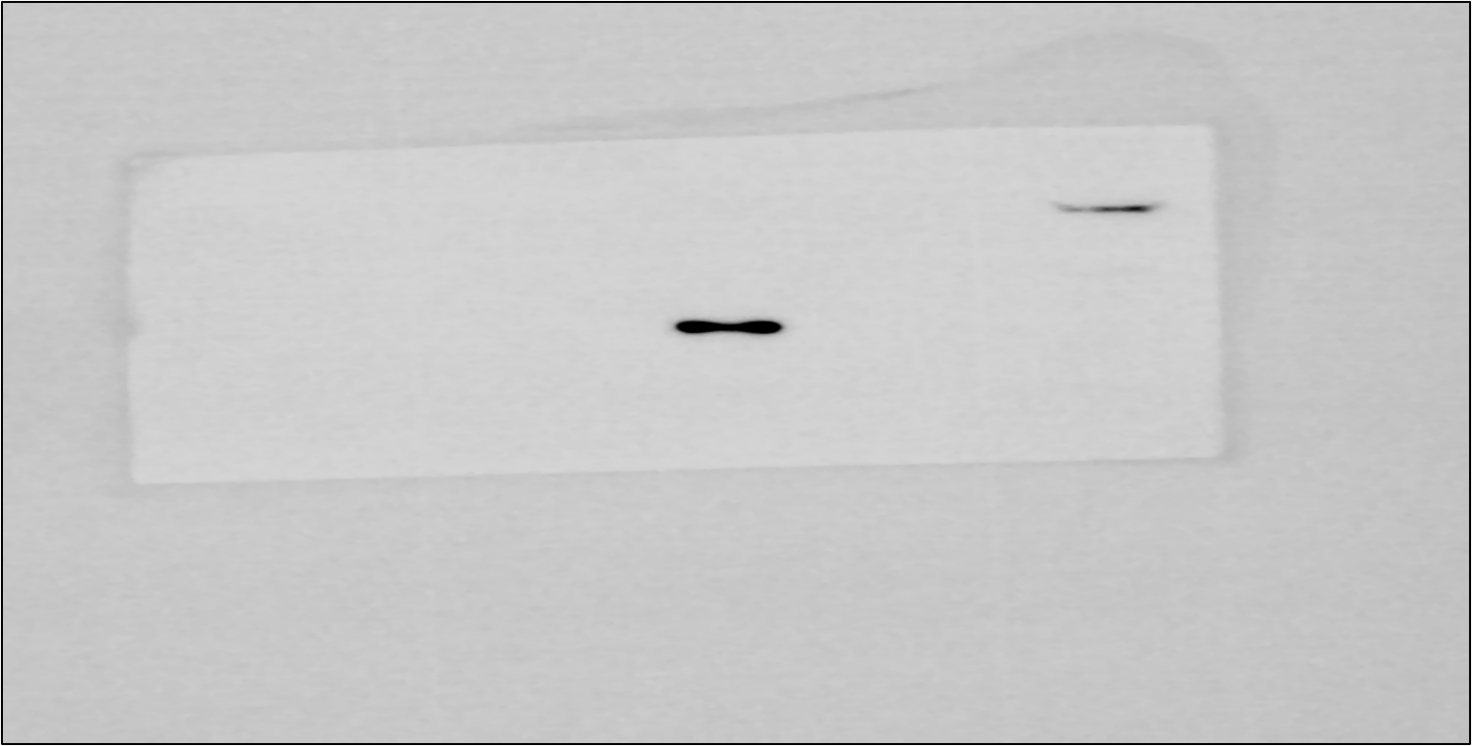

Supplement: Figure 4—source data 2. [file elife-108048-fig4-data2.zip › Figure 4/Figure 4 A IP-Myc.tif]

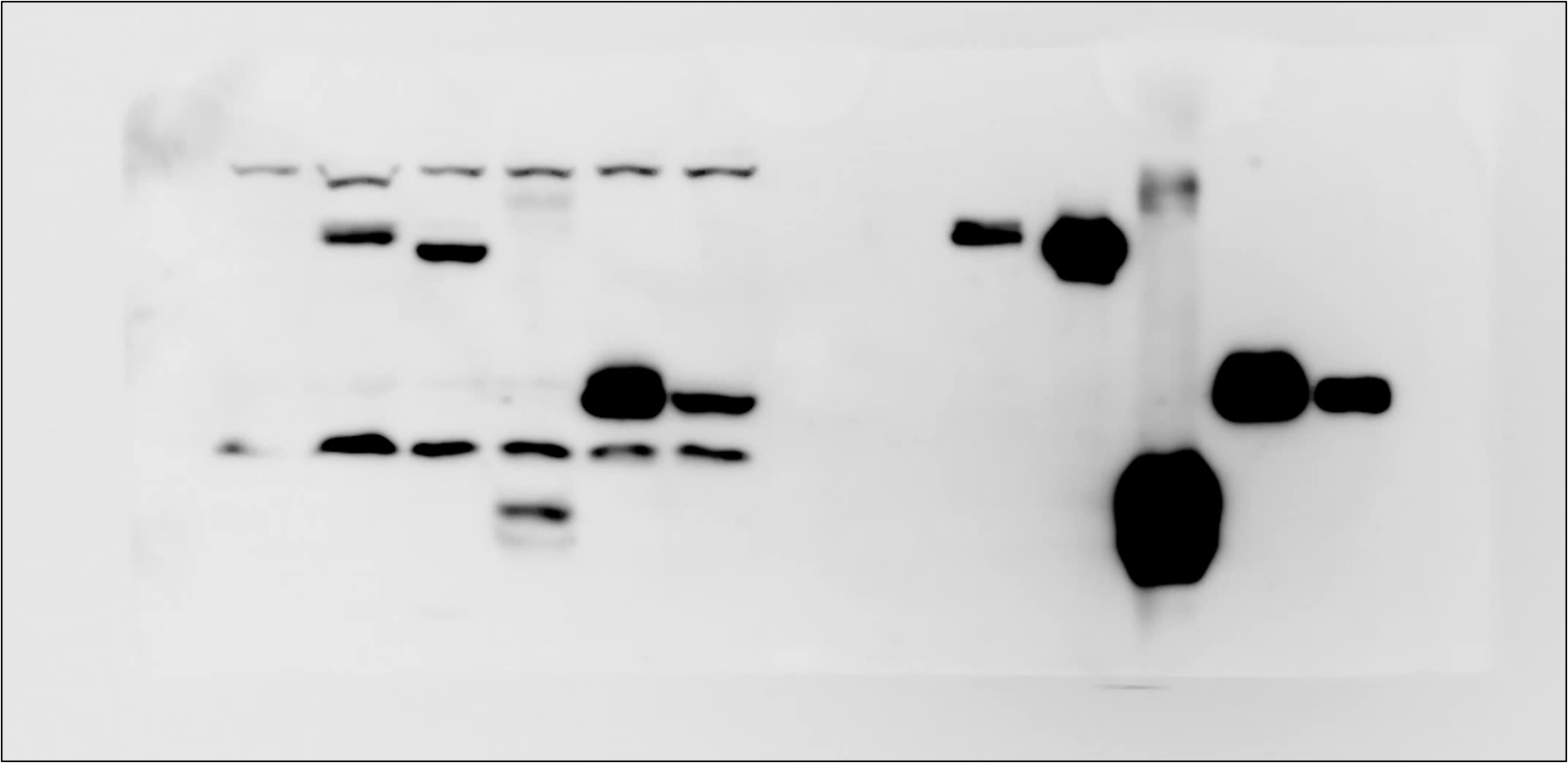

Supplement: Figure 4—source data 2. [file elife-108048-fig4-data2.zip › Figure 4/Figure 4 A WCL-Flag.tif]

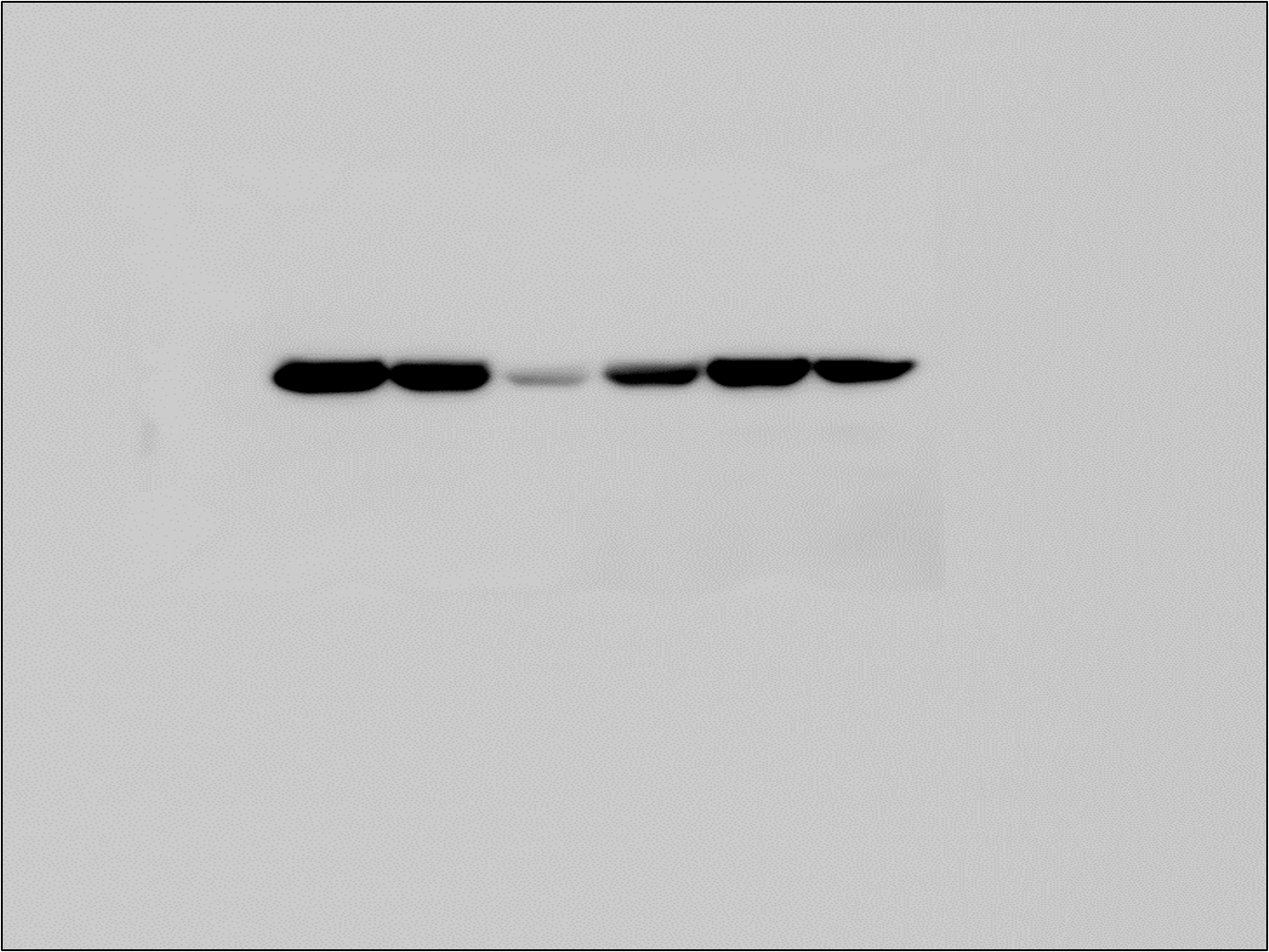

Supplement: Figure 4—source data 2. [file elife-108048-fig4-data2.zip › Figure 4/Figure 4 A WCL-Myc.tif]

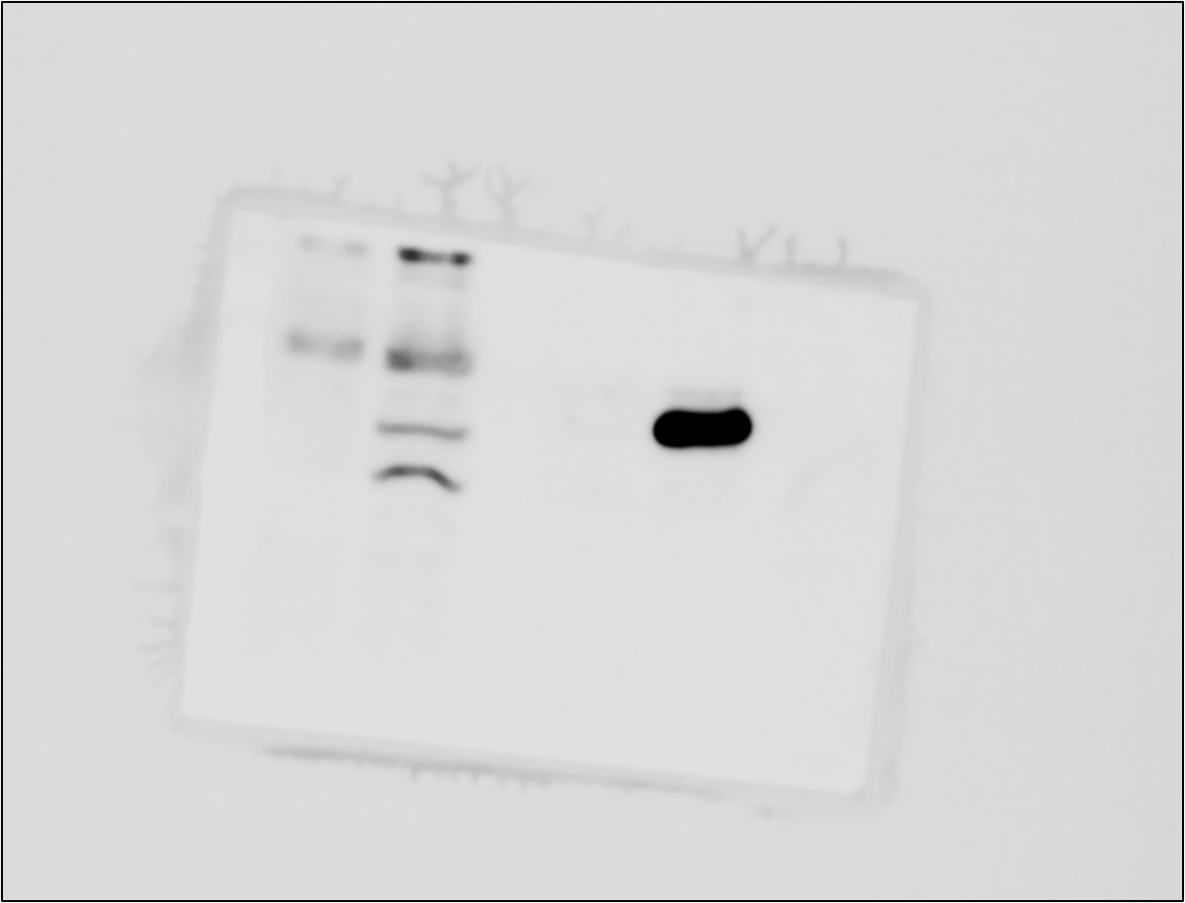

Supplement: Figure 4—source data 2. [file elife-108048-fig4-data2.zip › Figure 4/Figure 4 B IP-HA.tif]

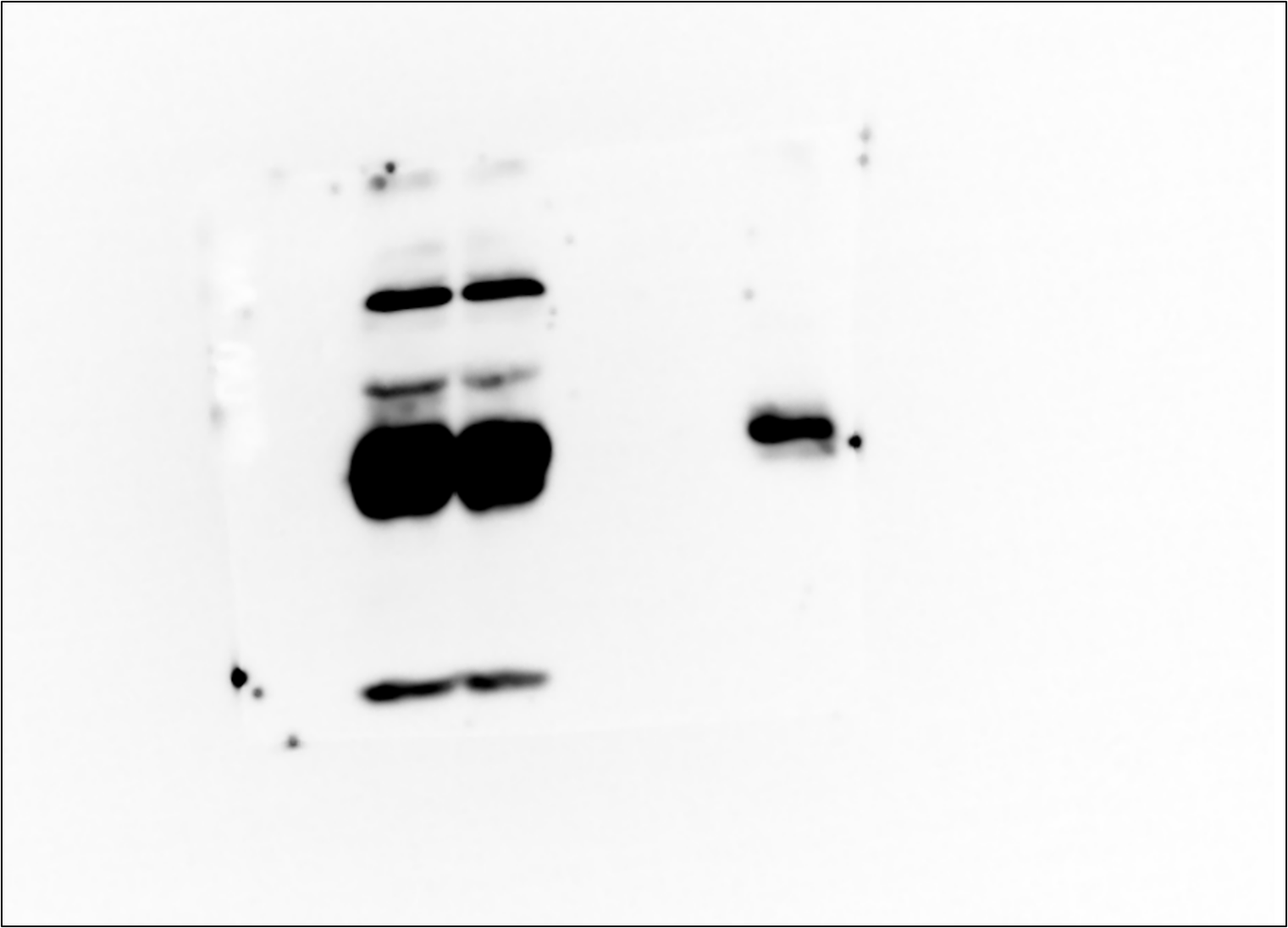

Supplement: Figure 4—source data 2. [file elife-108048-fig4-data2.zip › Figure 4/Figure 4 B IP-Myc.tif]

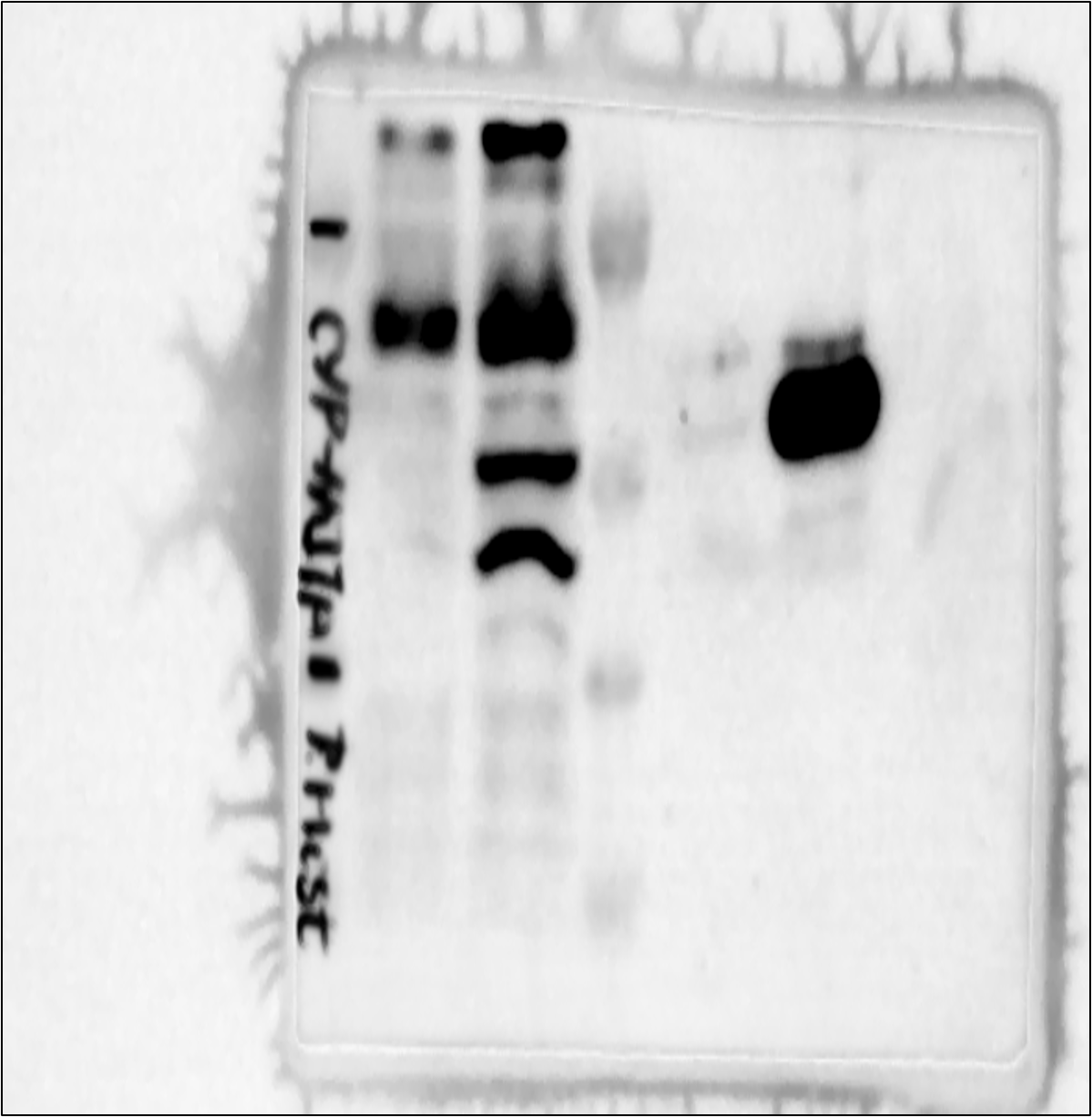

Supplement: Figure 4—source data 2. [file elife-108048-fig4-data2.zip › Figure 4/Figure 4 B WCL-HA.tif]

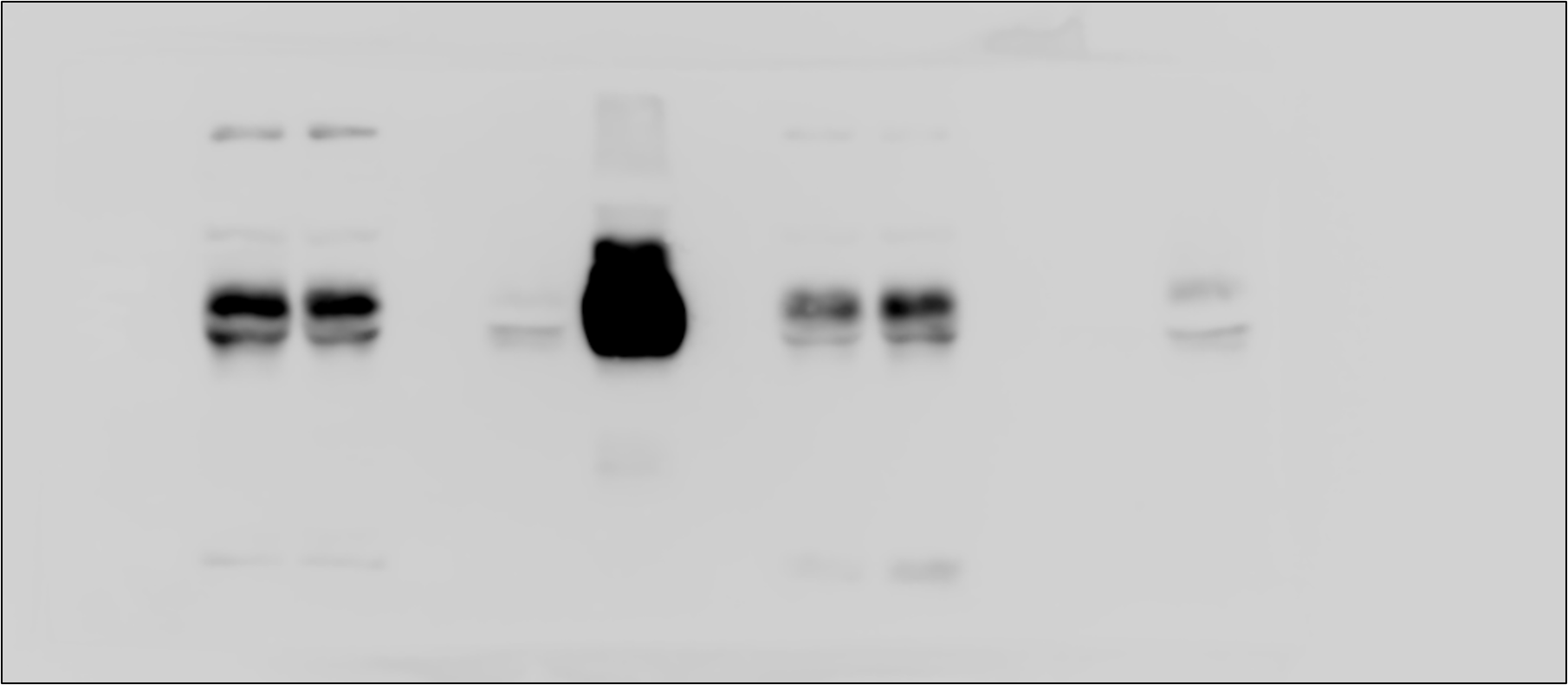

Supplement: Figure 4—source data 2. [file elife-108048-fig4-data2.zip › Figure 4/Figure 4 B WCL-Myc.tif]

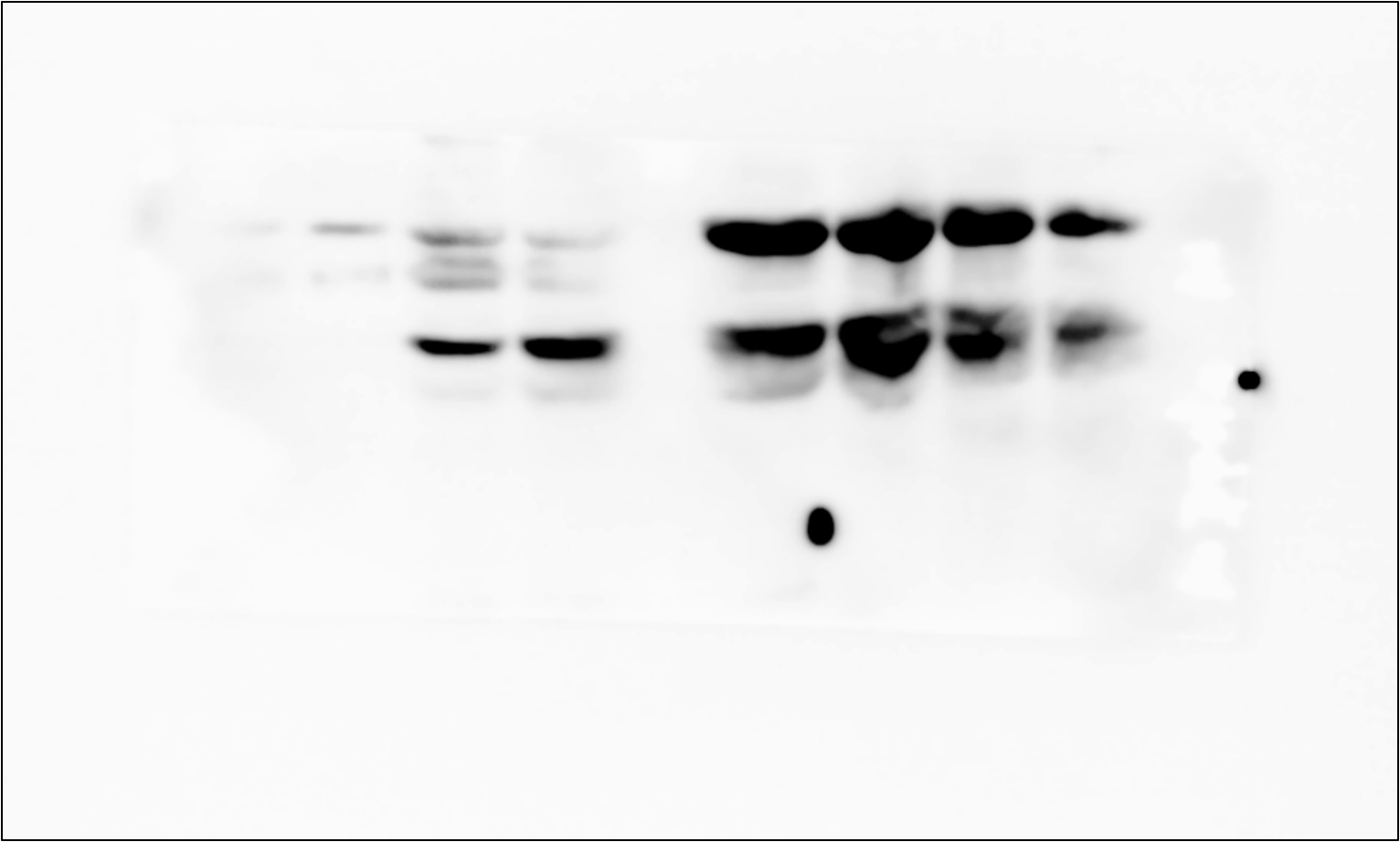

Supplement: Figure 4—source data 2. [file elife-108048-fig4-data2.zip › Figure 4/Figure 4 C Input-STING.tif]

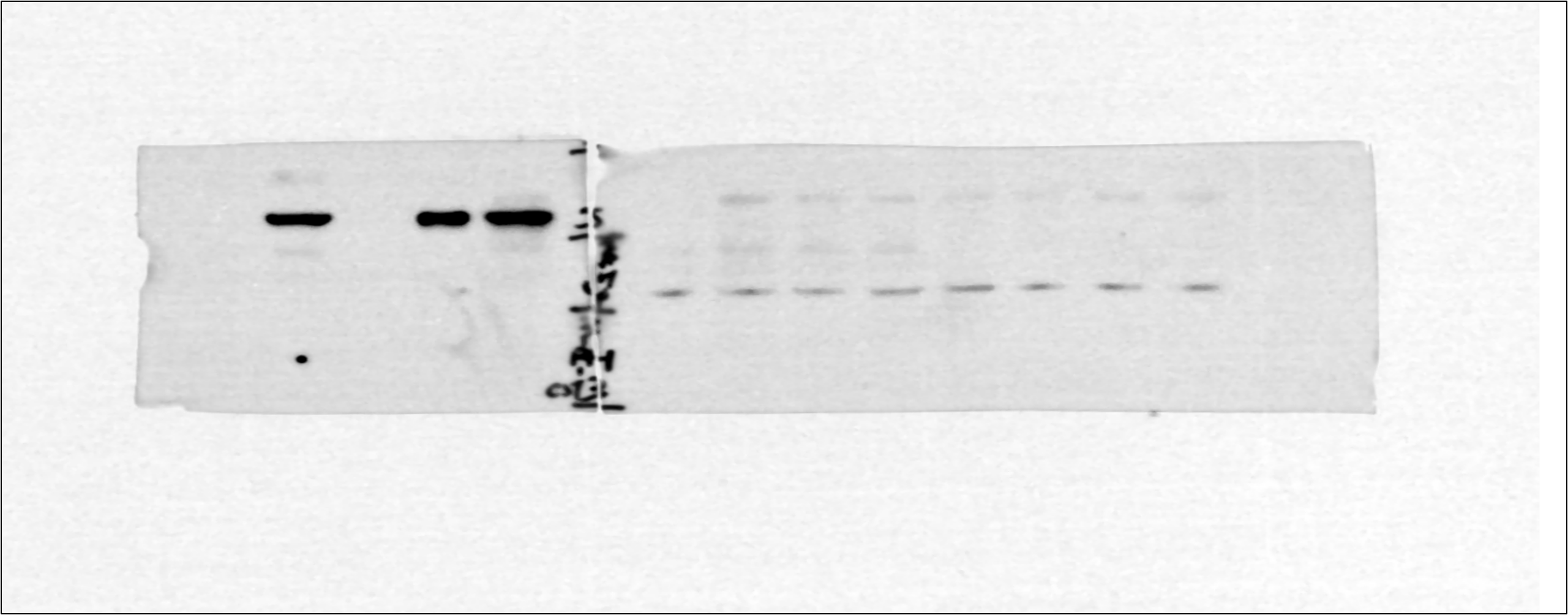

Supplement: Figure 4—source data 2. [file elife-108048-fig4-data2.zip › Figure 4/Figure 4 C IP-HA.tif]

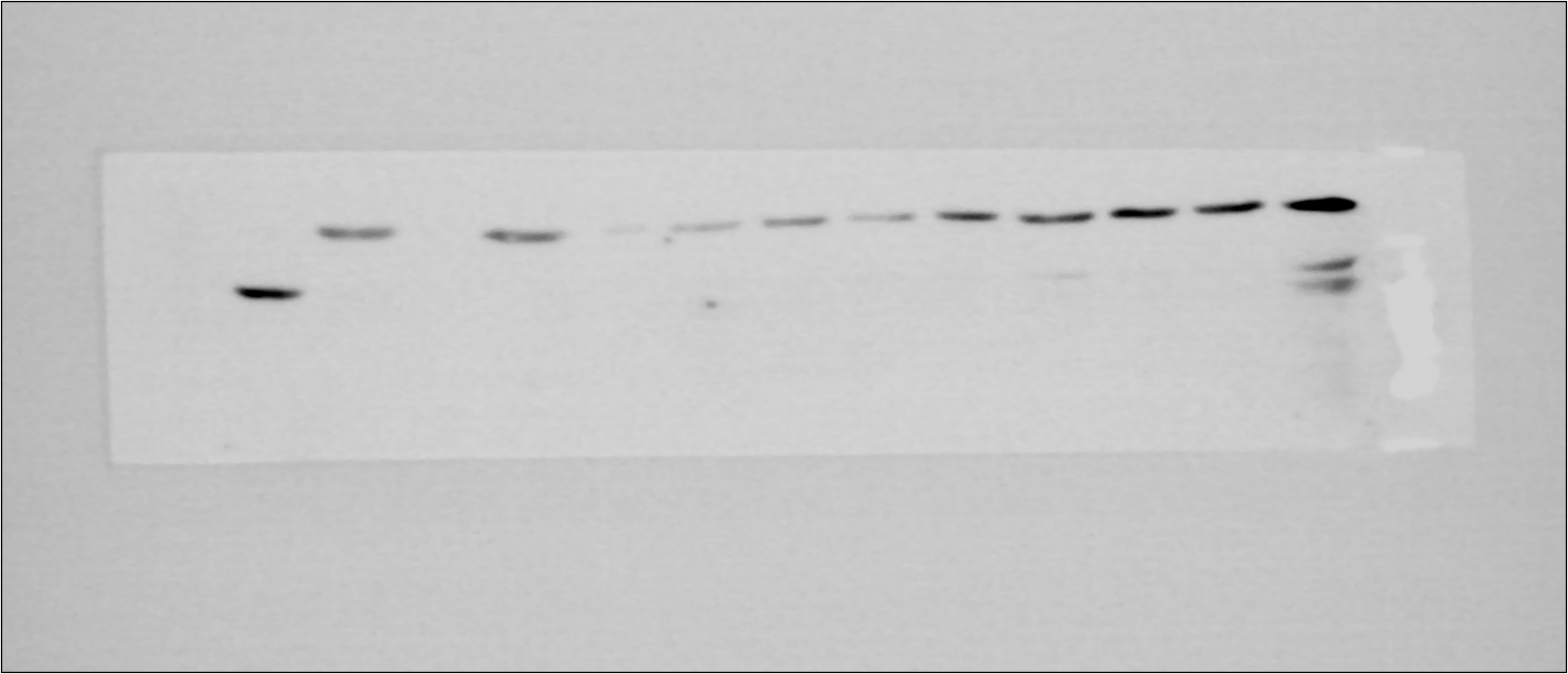

Supplement: Figure 4—source data 2. [file elife-108048-fig4-data2.zip › Figure 4/Figure 4 C IP-STING.tif]

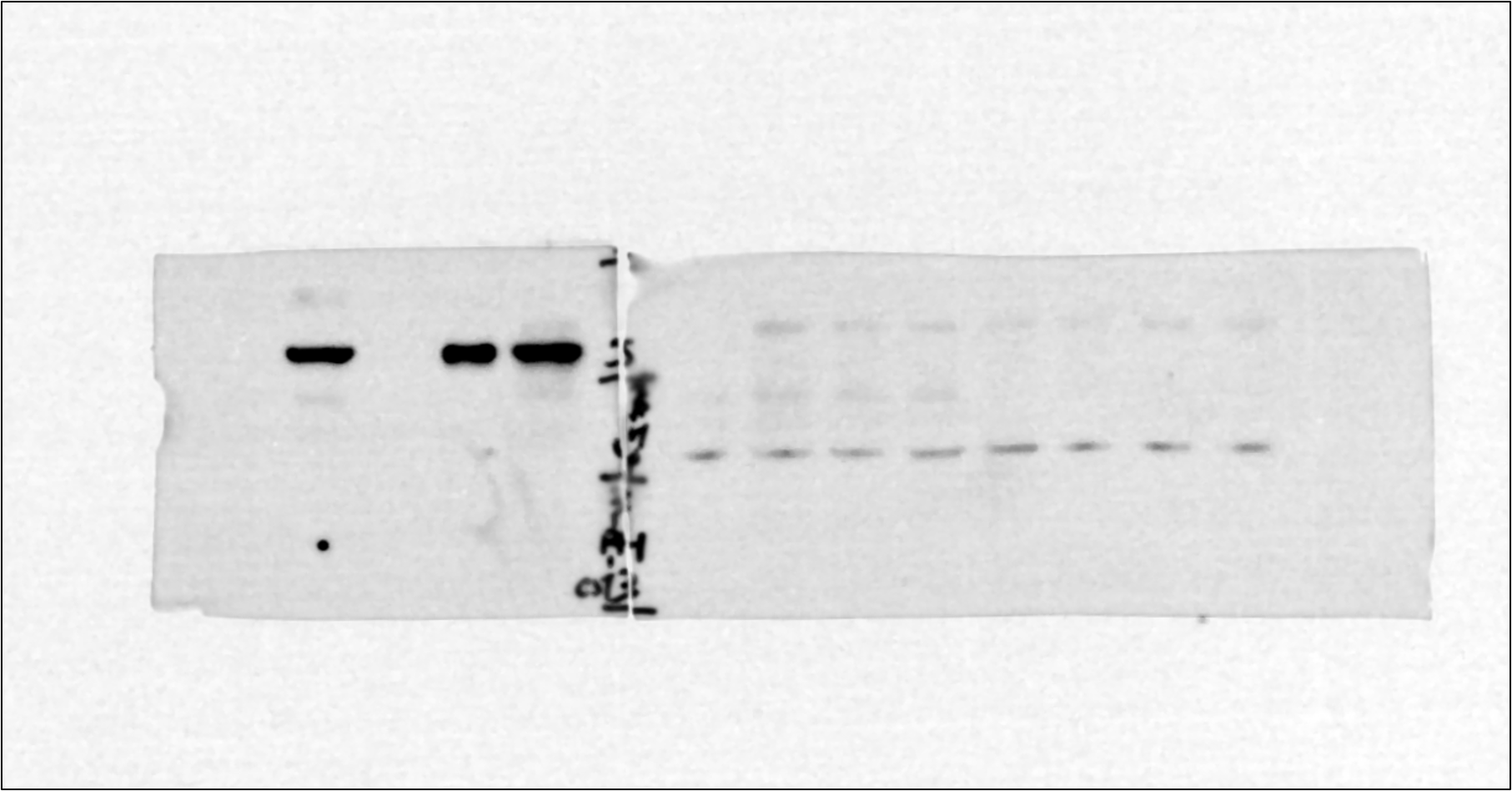

Supplement: Figure 4—source data 2. [file elife-108048-fig4-data2.zip › Figure 4/Figure 4 C WCL-HA.tif]

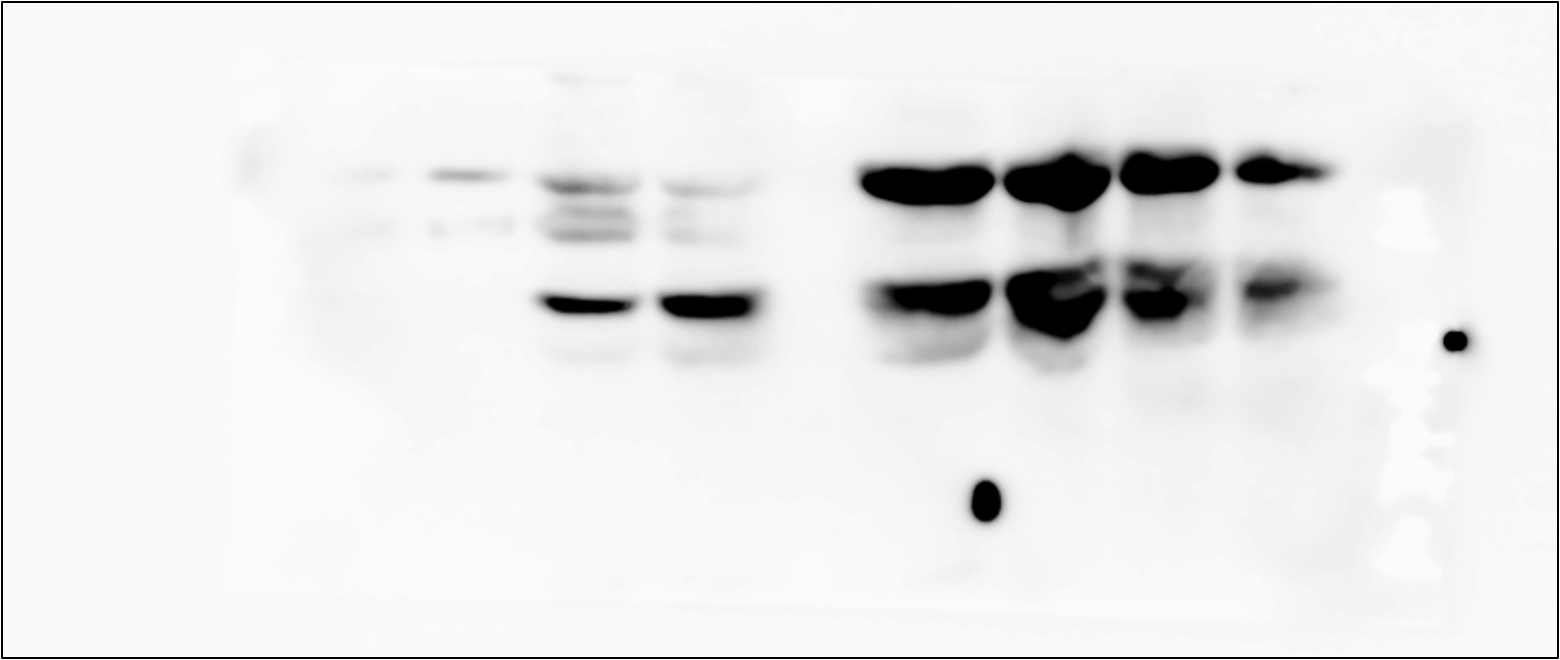

Supplement: Figure 4—source data 2. [file elife-108048-fig4-data2.zip › Figure 4/Figure 4 C WCL-STING.tif]

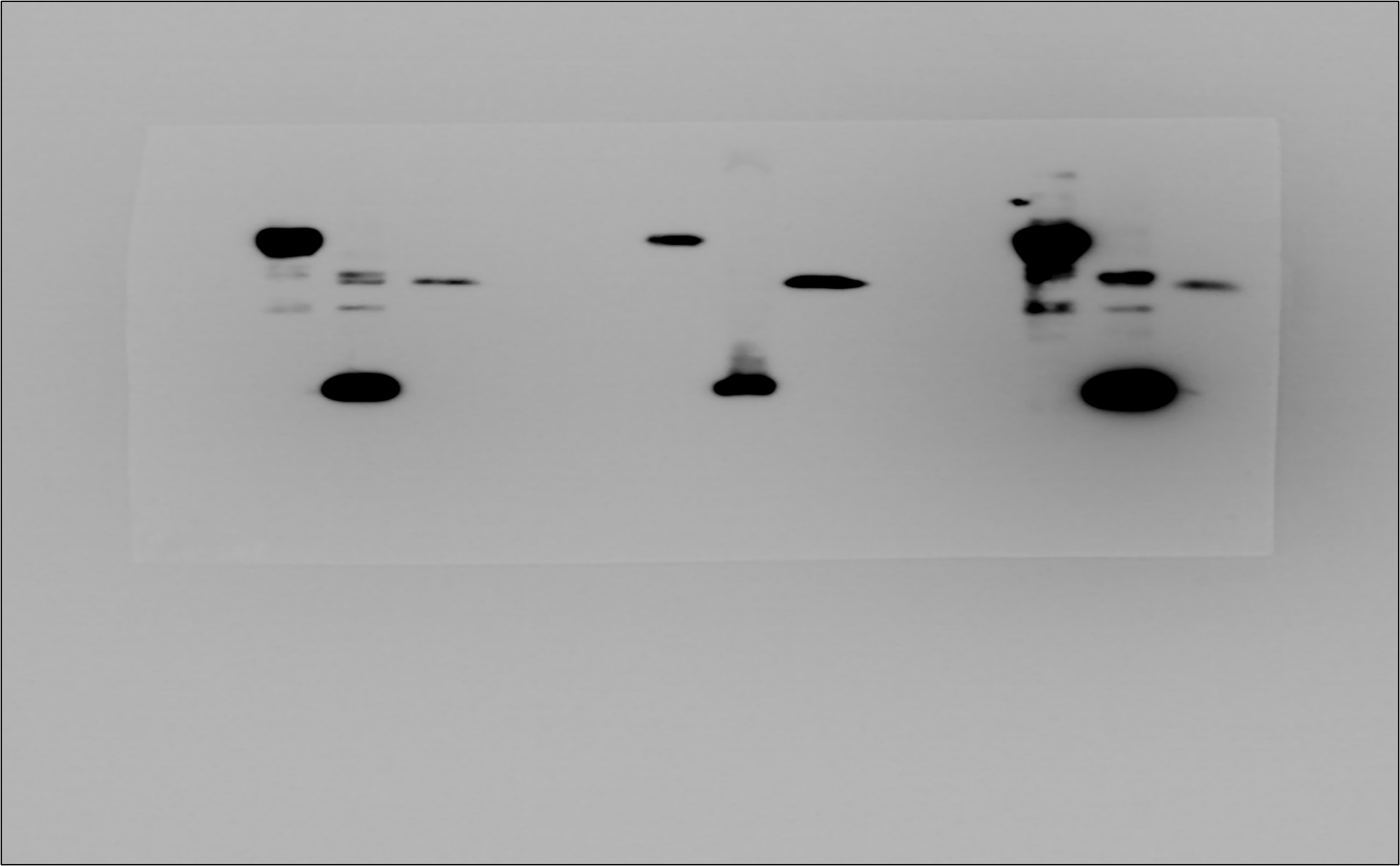

Supplement: Figure 4—source data 2. [file elife-108048-fig4-data2.zip › Figure 4/Figure 4 E IP-HA.tif]

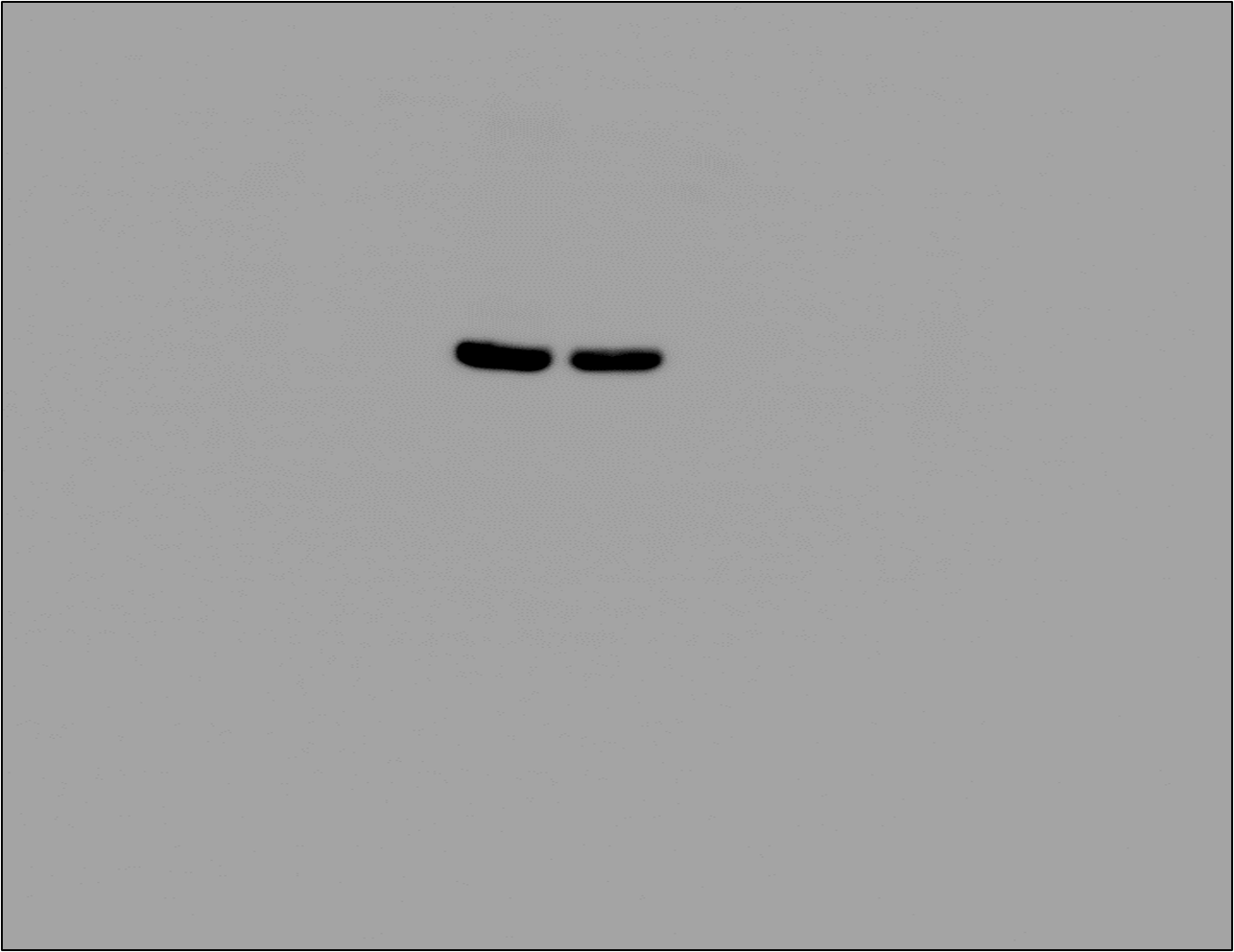

Supplement: Figure 4—source data 2. [file elife-108048-fig4-data2.zip › Figure 4/Figure 4 E IP-Myc.tif]

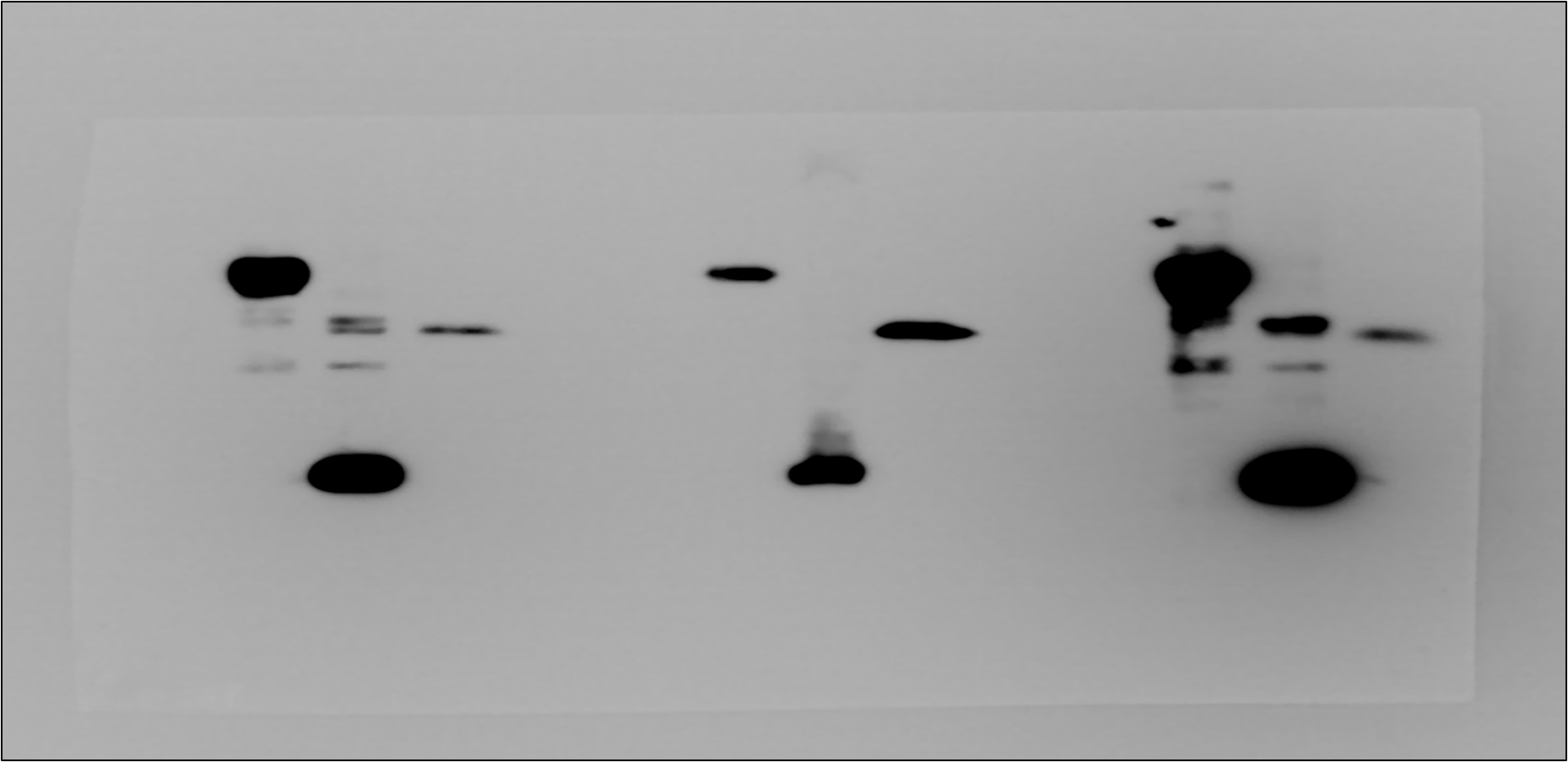

Supplement: Figure 4—source data 2. [file elife-108048-fig4-data2.zip › Figure 4/Figure 4 E WCL-HA.tif]

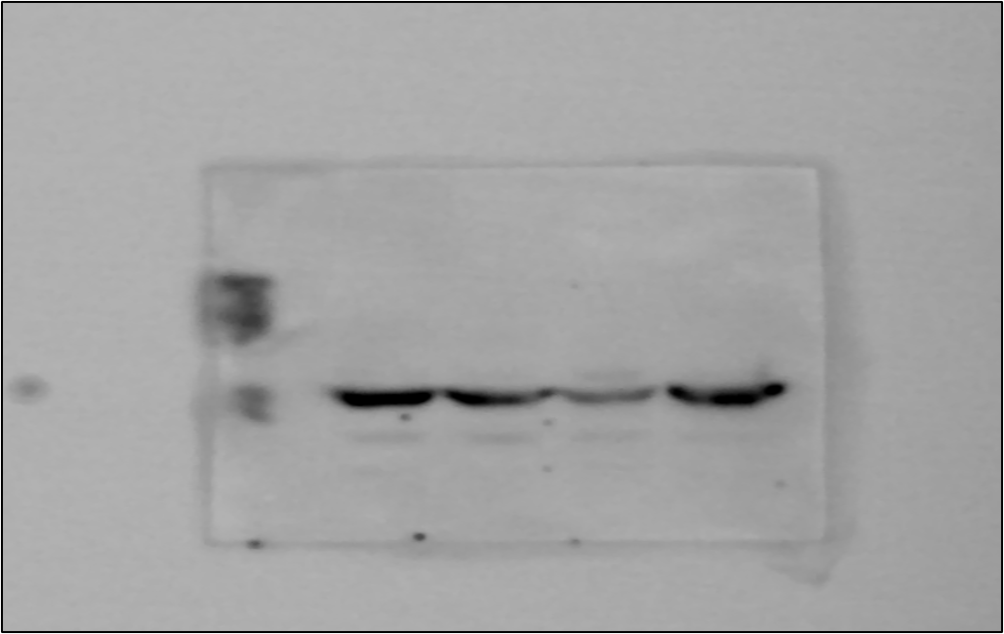

Supplement: Figure 4—source data 2. [file elife-108048-fig4-data2.zip › Figure 4/Figure 4 E WCL-Myc.tif]

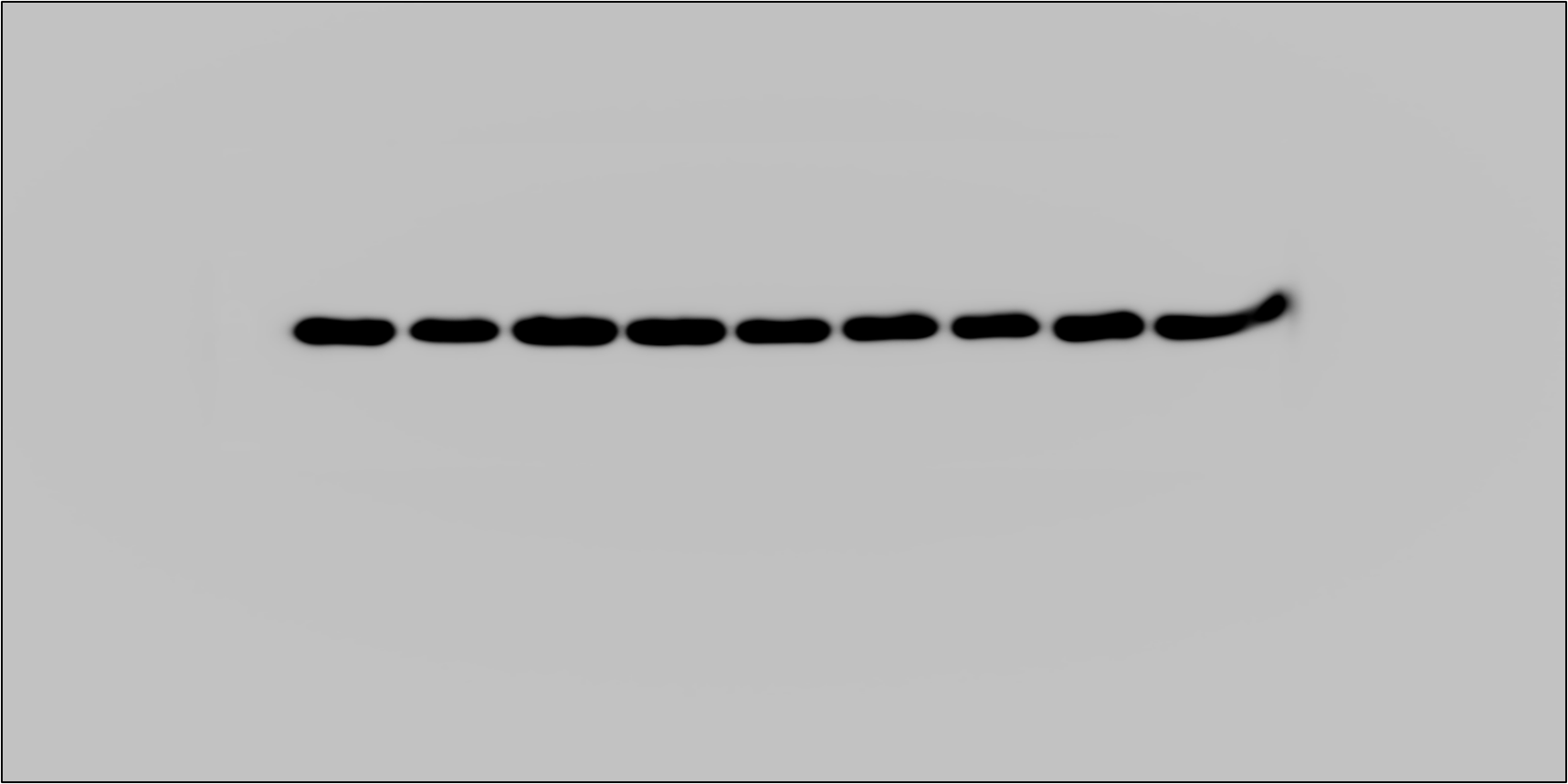

Supplement: Figure 4—source data 2. [file elife-108048-fig4-data2.zip › Figure 4/Figure 4 I-Actin.tif]

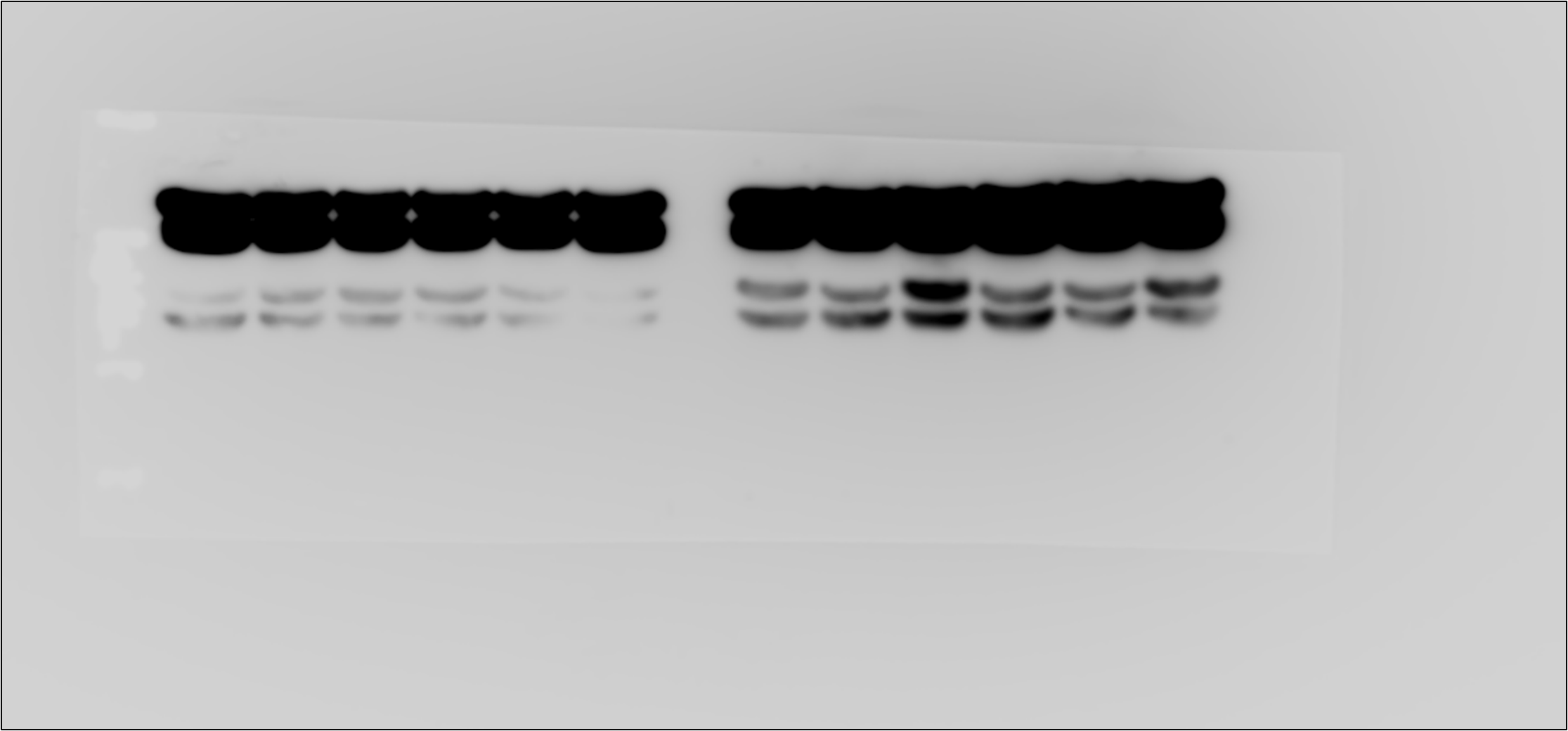

Supplement: Figure 4—source data 2. [file elife-108048-fig4-data2.zip › Figure 4/Figure 4 I-Flag.tif]

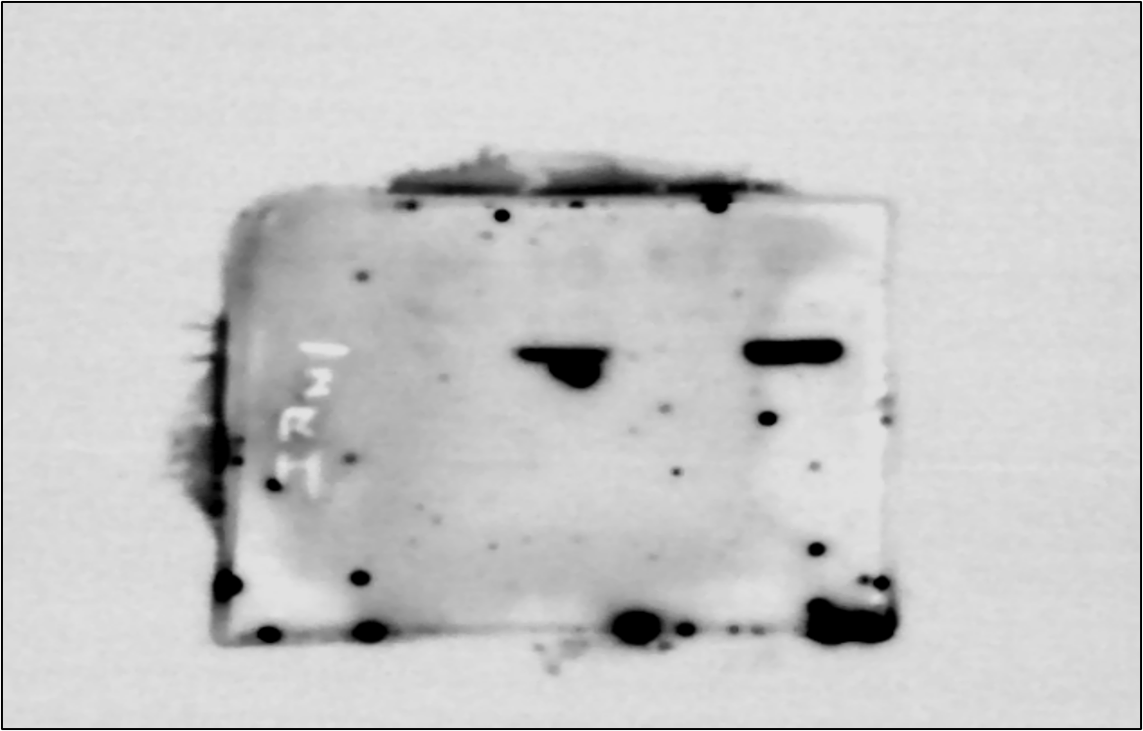

Supplement: Figure 4—source data 2. [file elife-108048-fig4-data2.zip › Figure 4/Figure 4 I-HA.tif]

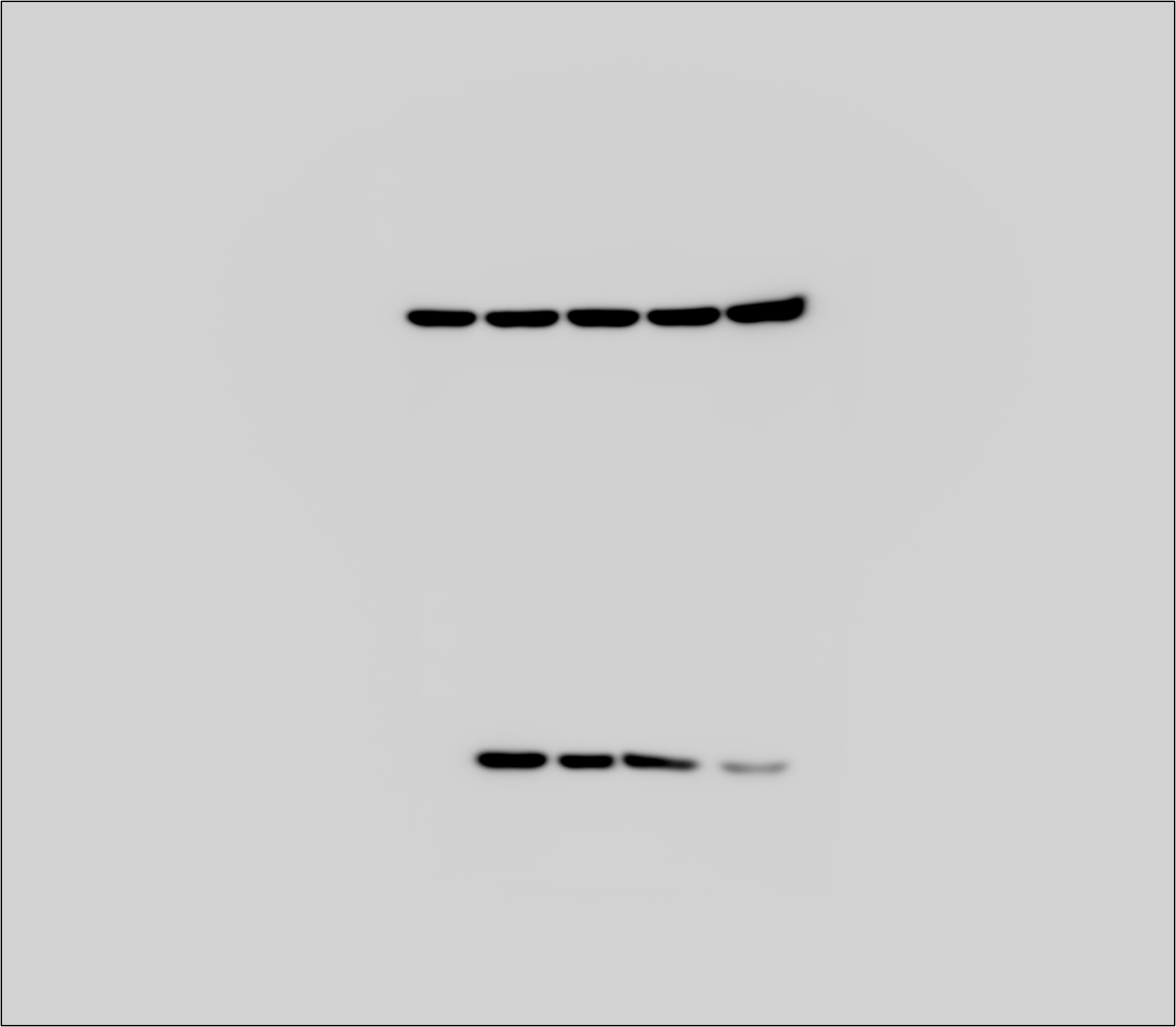

Supplement: Figure 4—source data 2. [file elife-108048-fig4-data2.zip › Figure 4/Figure 4 J-Actin.tif]

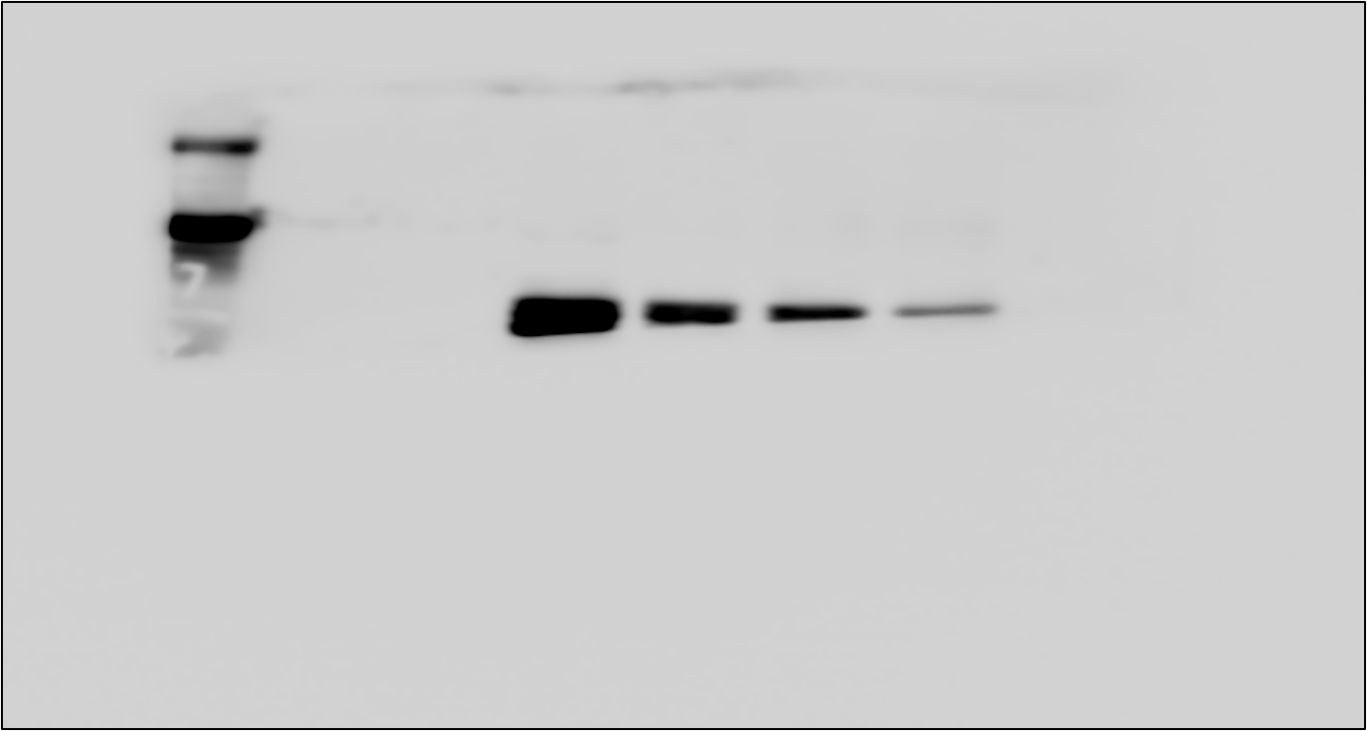

Supplement: Figure 4—source data 2. [file elife-108048-fig4-data2.zip › Figure 4/Figure 4 J-G.tif]

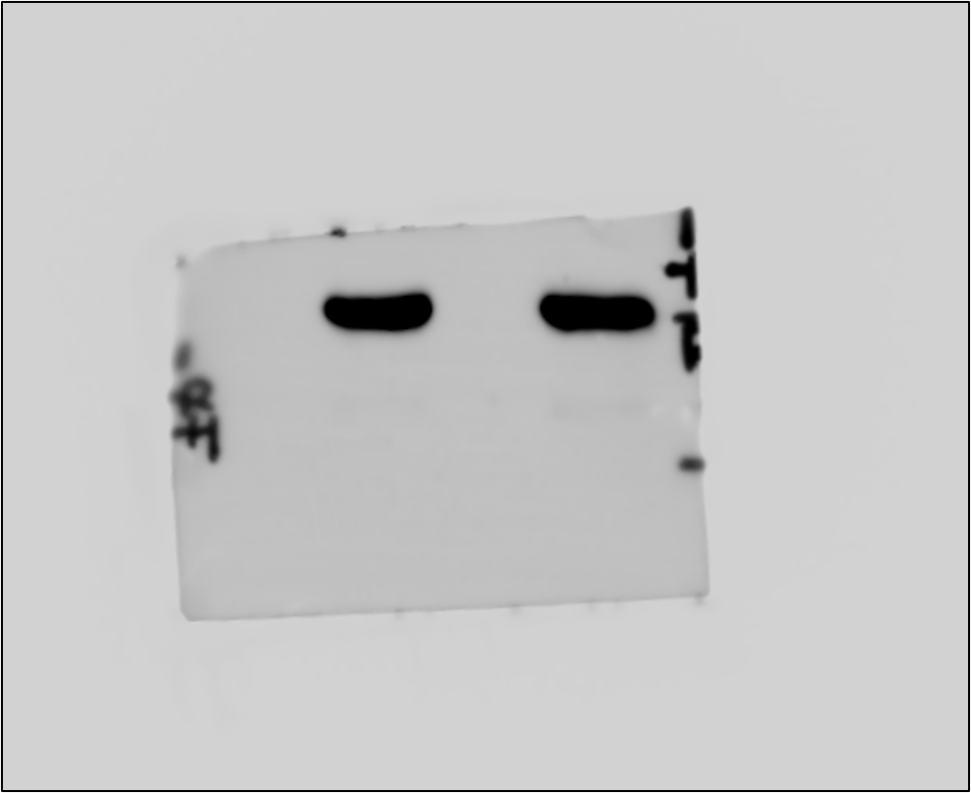

Supplement: Figure 4—source data 2. [file elife-108048-fig4-data2.zip › Figure 4/Figure 4 J-HA.tif]

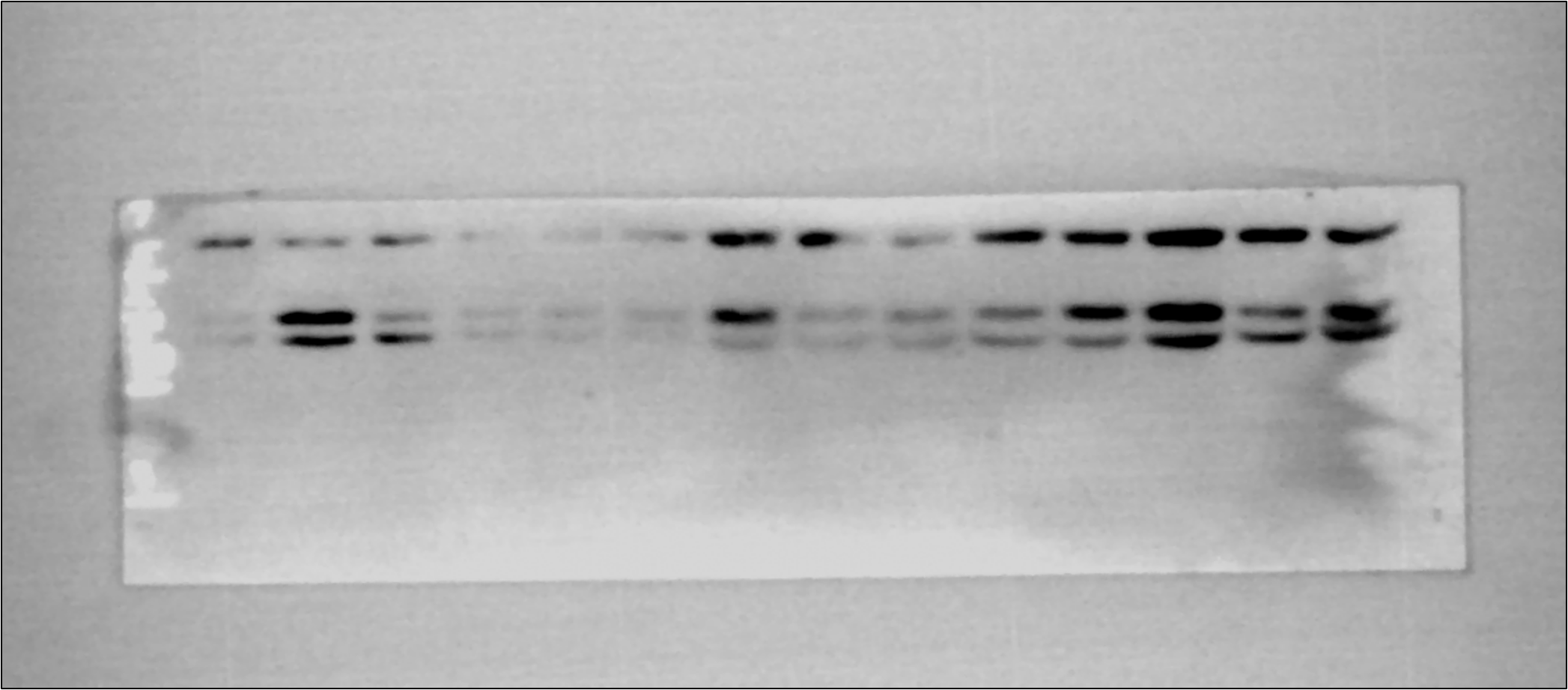

Supplement: Figure 4—source data 2. [file elife-108048-fig4-data2.zip › Figure 4/Figure 4 J-STING.tif]

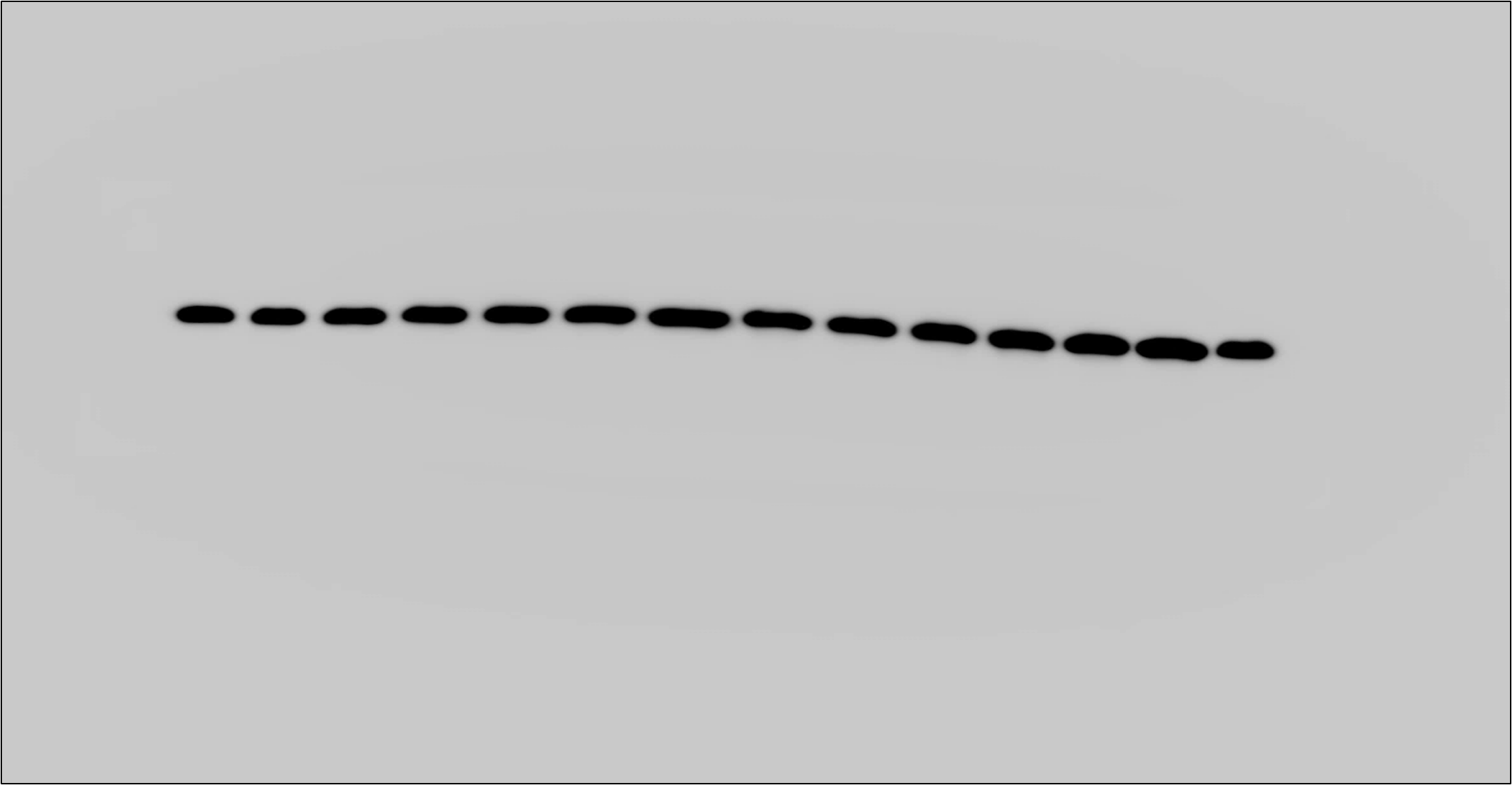

Supplement: Figure 4—source data 2. [file elife-108048-fig4-data2.zip › Figure 4/Figure 4 K-Actin.tif]

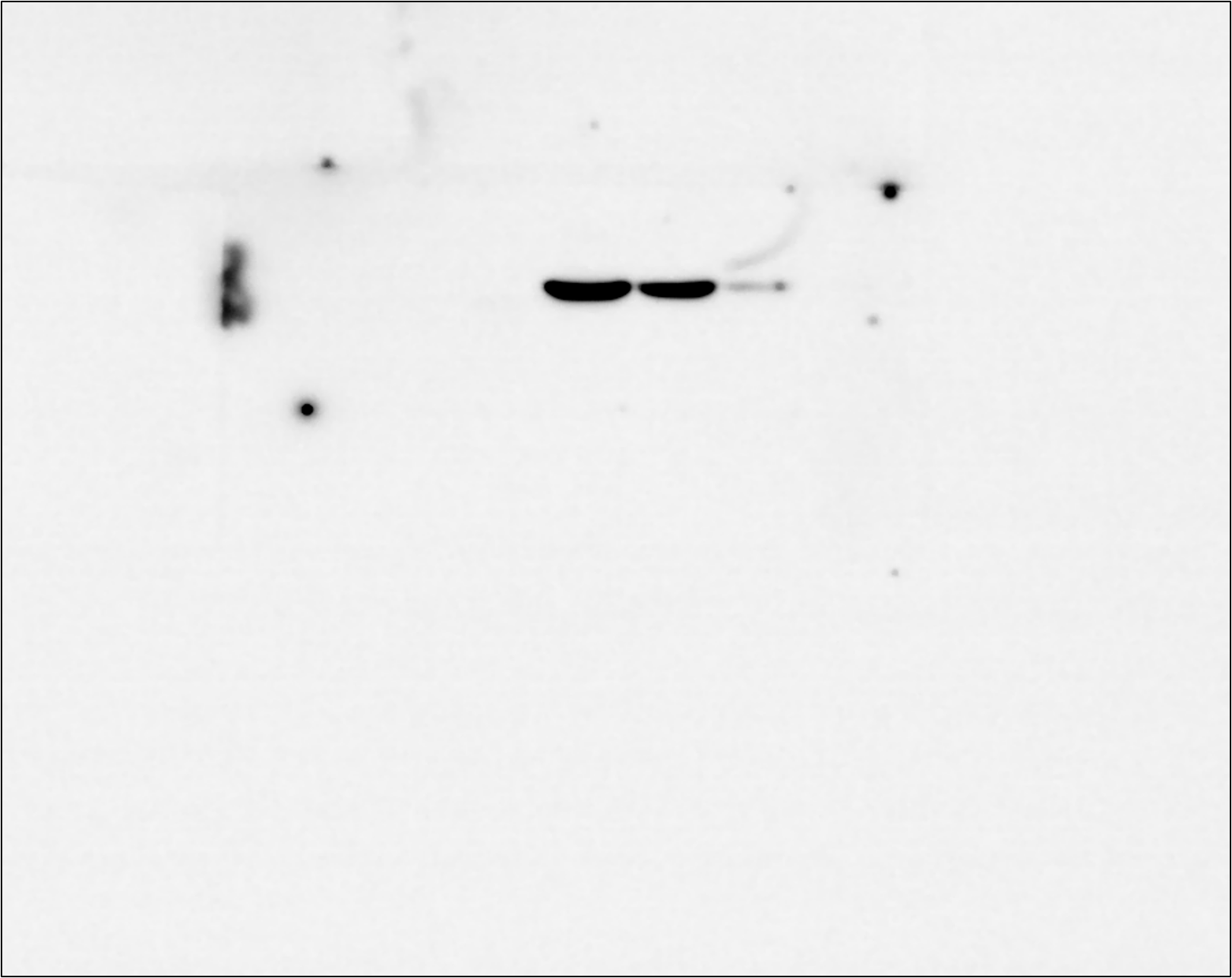

Supplement: Figure 4—source data 2. [file elife-108048-fig4-data2.zip › Figure 4/Figure 4 K-HA.tif]

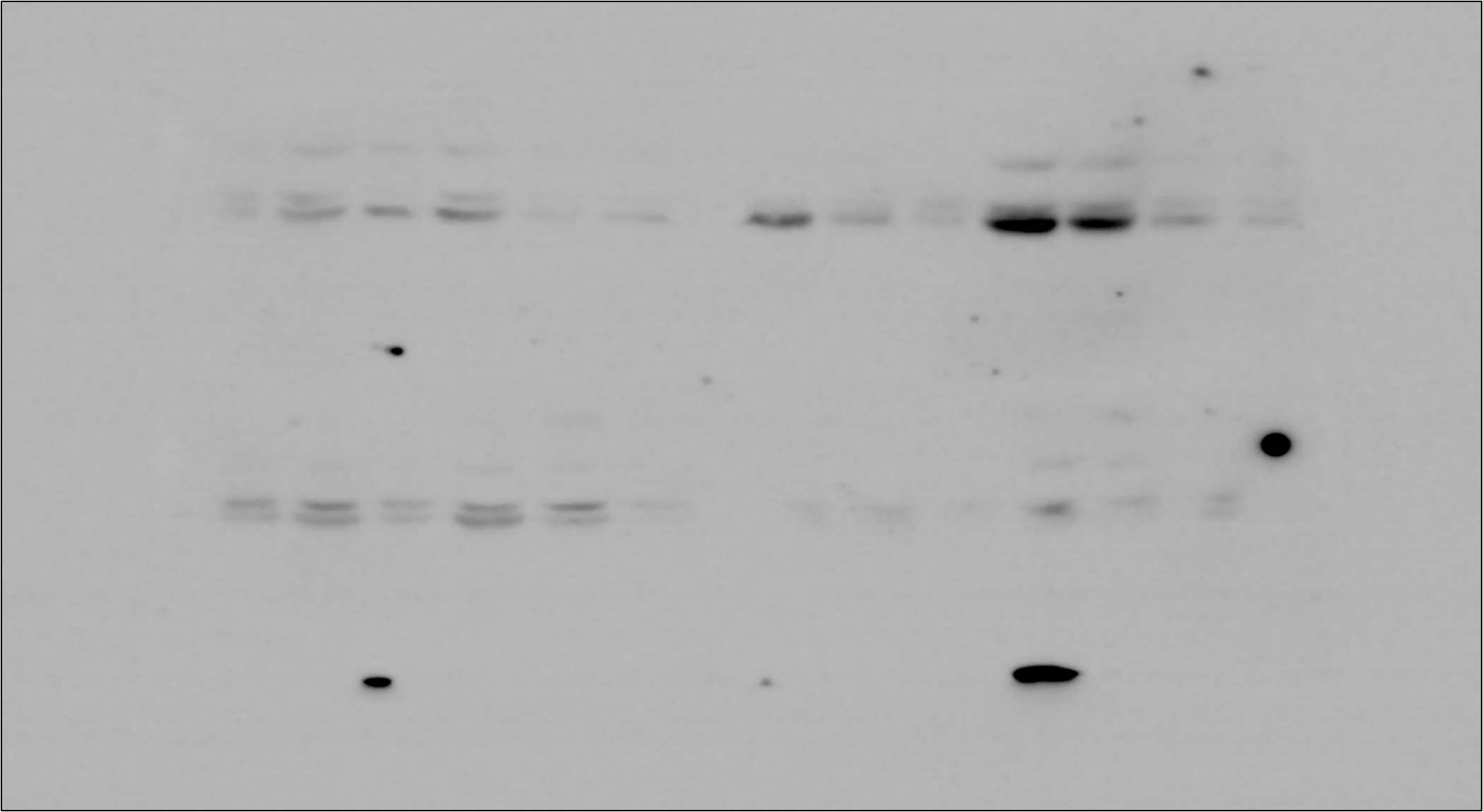

Supplement: Figure 4—source data 2. [file elife-108048-fig4-data2.zip › Figure 4/Figure 4 K-STING.tif]

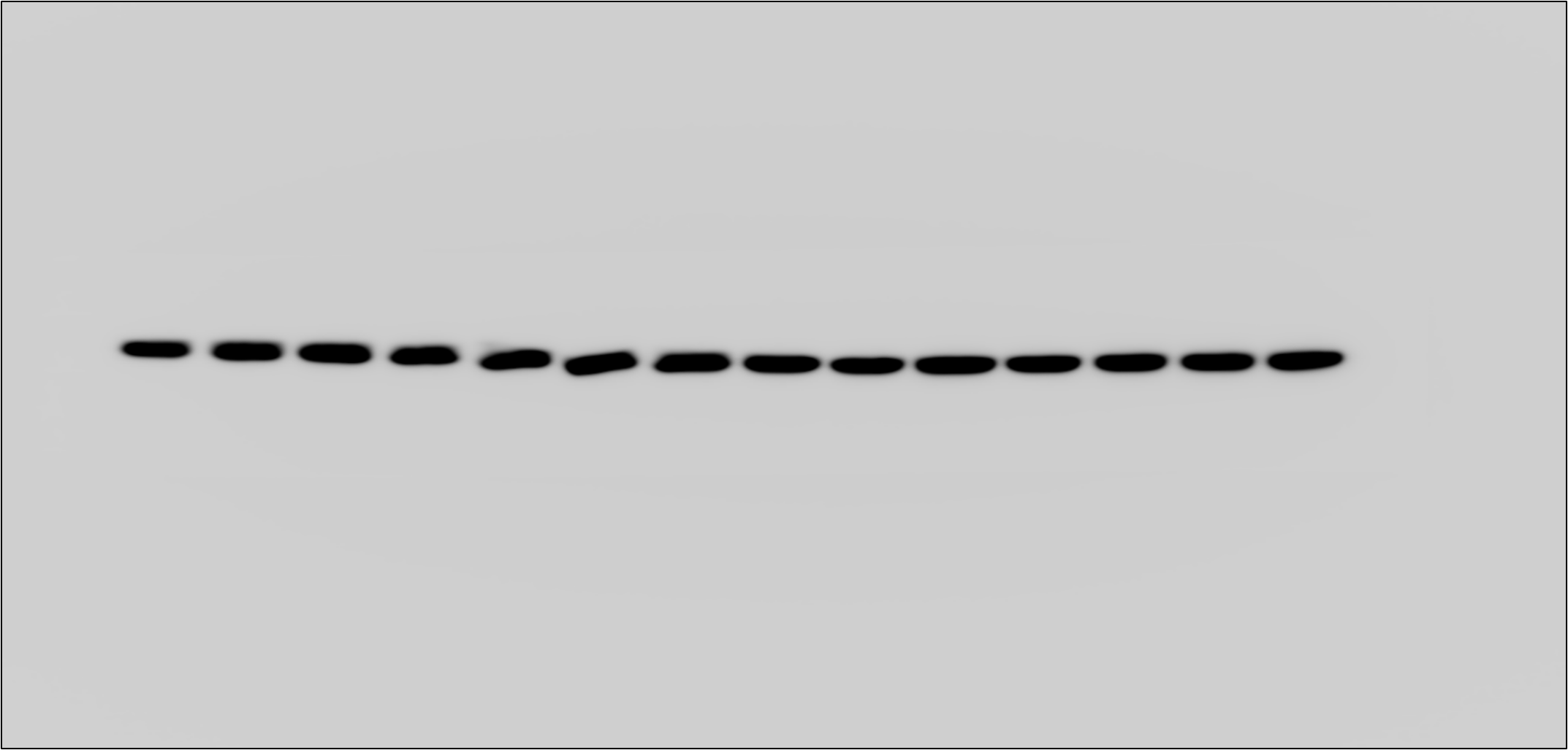

Supplement: Figure 4—figure supplement 1—source data 2. [file elife-108048-fig4-figsupp1-data2.zip › Figure 4-figure supplement 1/Figure S4 C-Actin.tif]

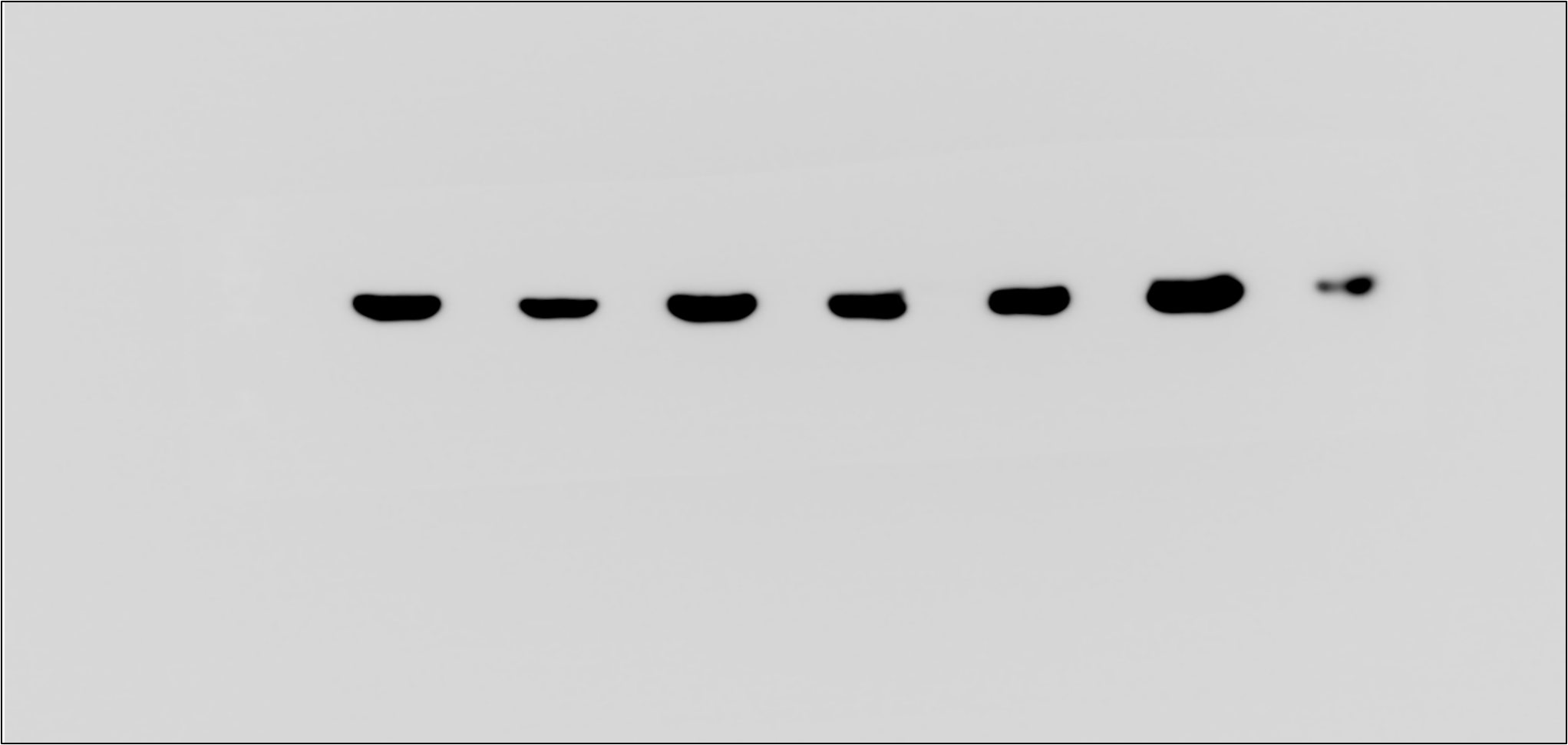

Supplement: Figure 4—figure supplement 1—source data 2. [file elife-108048-fig4-figsupp1-data2.zip › Figure 4-figure supplement 1/Figure S4 C-HA.tif]

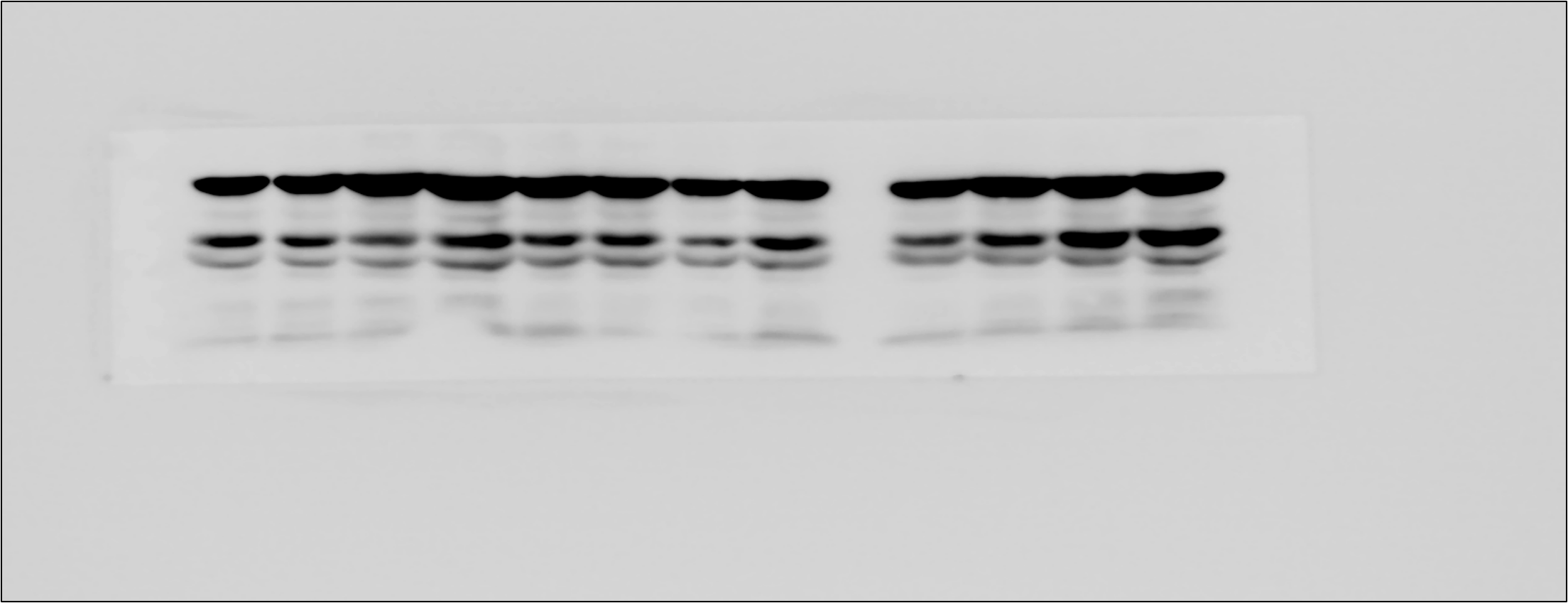

Supplement: Figure 4—figure supplement 1—source data 2. [file elife-108048-fig4-figsupp1-data2.zip › Figure 4-figure supplement 1/Figure S4 C-STING.tif]

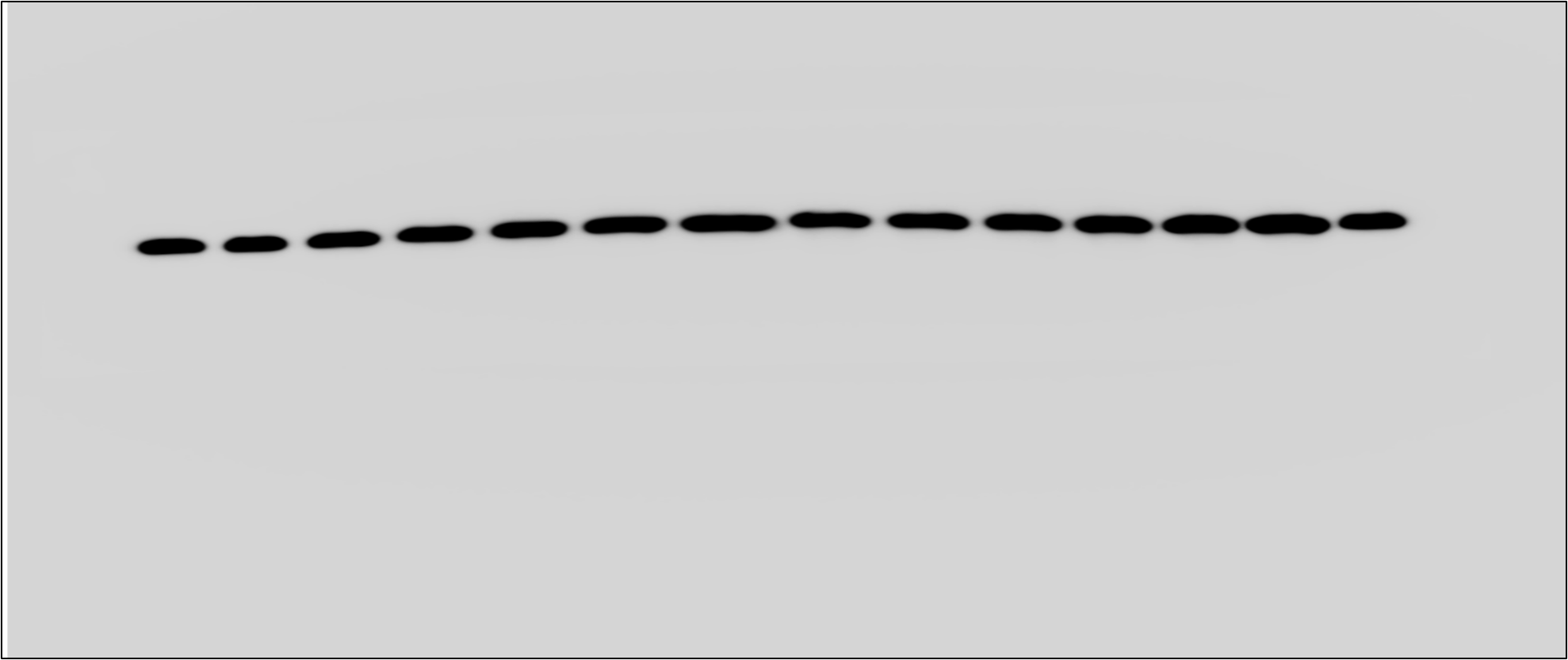

Supplement: Figure 4—figure supplement 1—source data 2. [file elife-108048-fig4-figsupp1-data2.zip › Figure 4-figure supplement 1/Figure S4 D-Actin.tif]

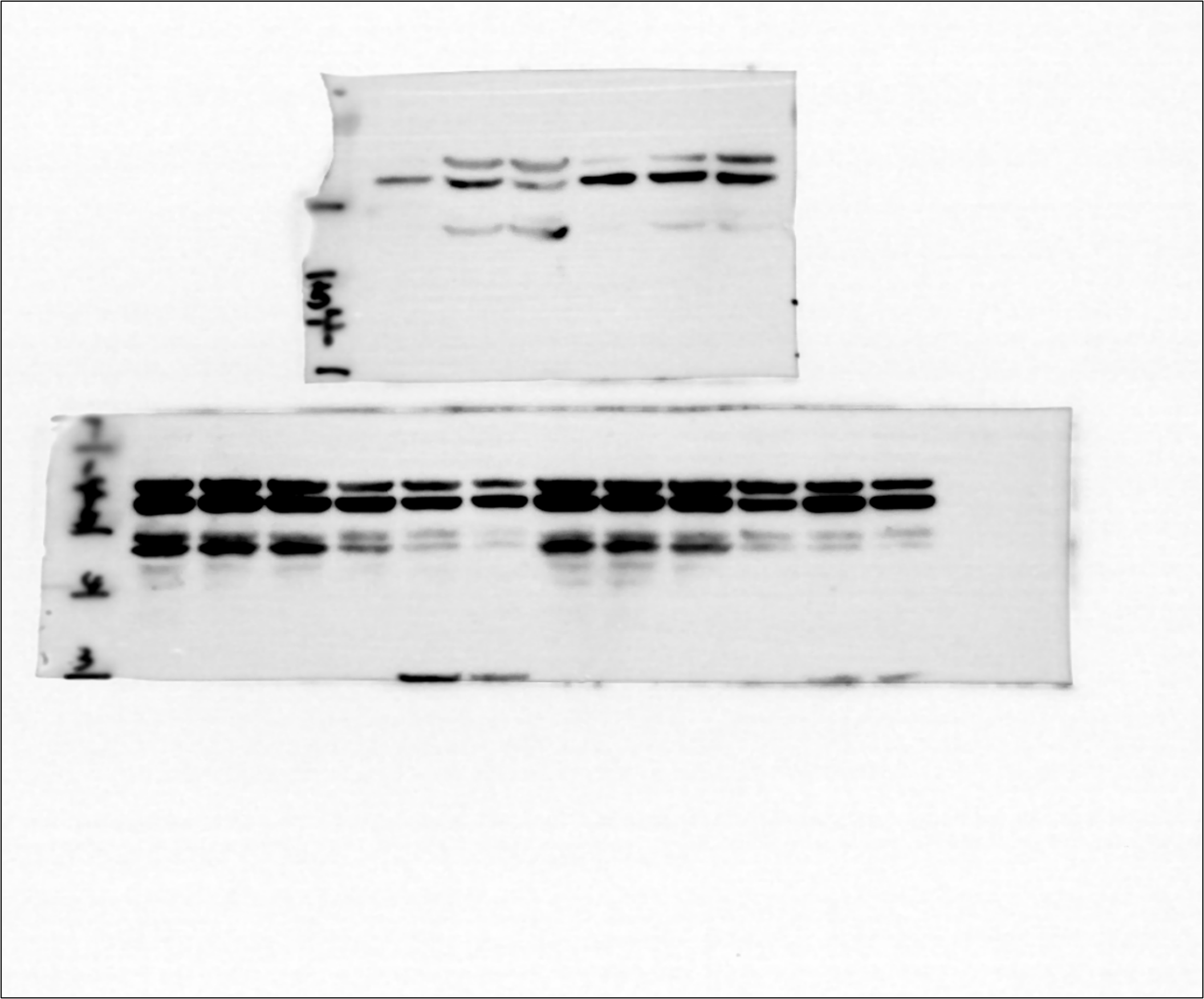

Supplement: Figure 4—figure supplement 1—source data 2. [file elife-108048-fig4-figsupp1-data2.zip › Figure 4-figure supplement 1/Figure S4 D-Flag.tif]

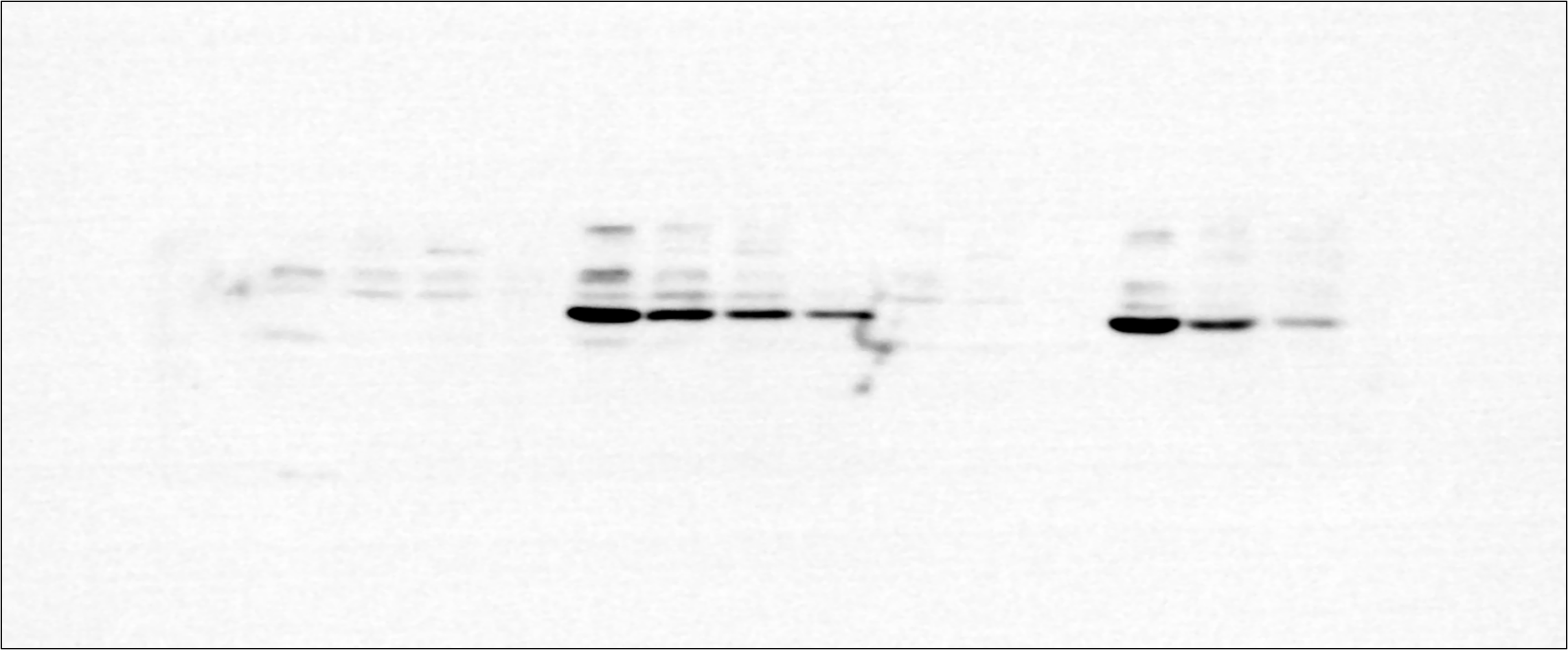

Supplement: Figure 4—figure supplement 1—source data 2. [file elife-108048-fig4-figsupp1-data2.zip › Figure 4-figure supplement 1/Figure S4 D-HA.tif]

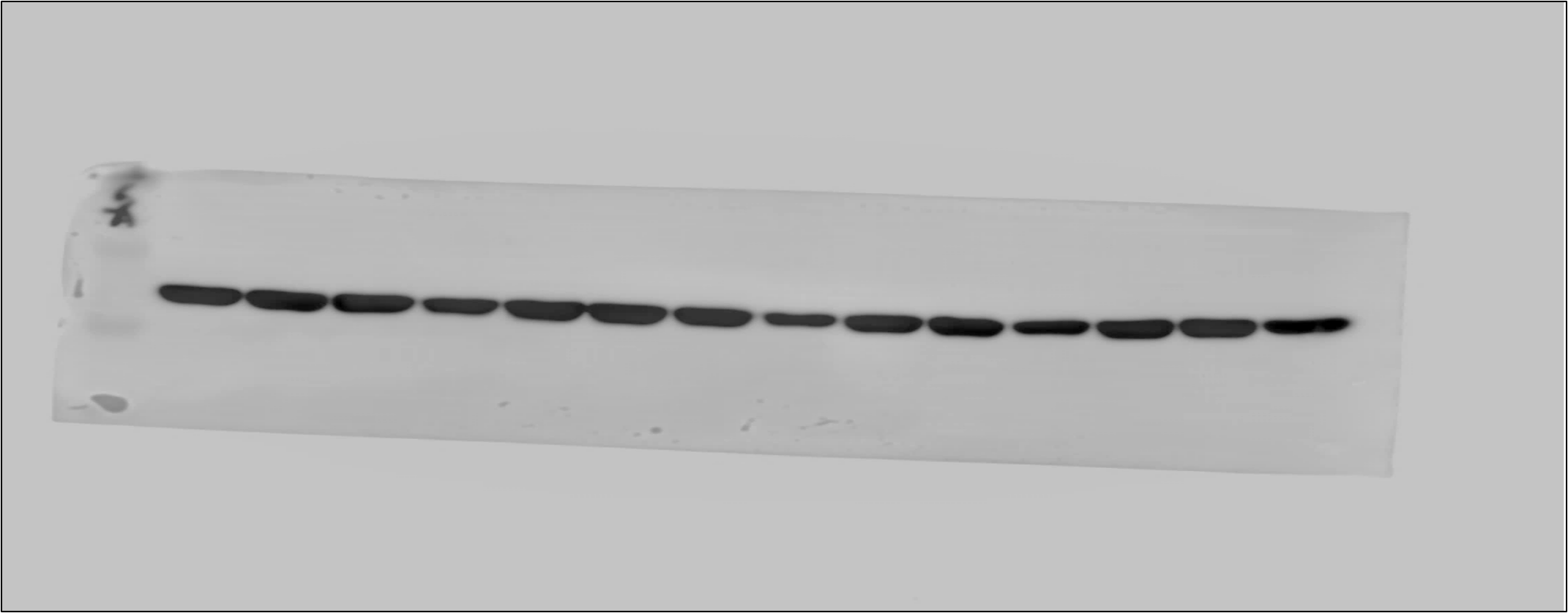

Supplement: Figure 4—figure supplement 1—source data 2. [file elife-108048-fig4-figsupp1-data2.zip › Figure 4-figure supplement 1/Figure S4 E-Actin.tif]

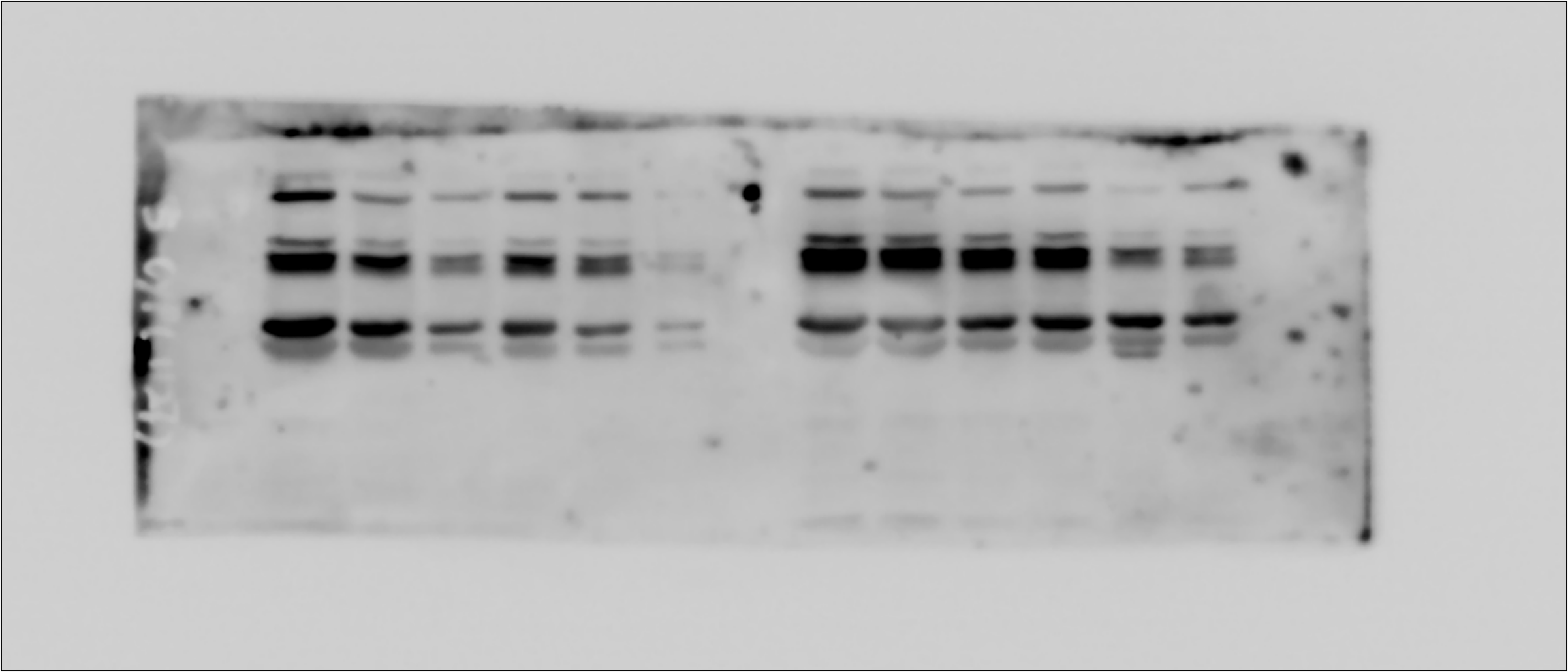

Supplement: Figure 4—figure supplement 1—source data 2. [file elife-108048-fig4-figsupp1-data2.zip › Figure 4-figure supplement 1/Figure S4 E-cyp17a2.tif]

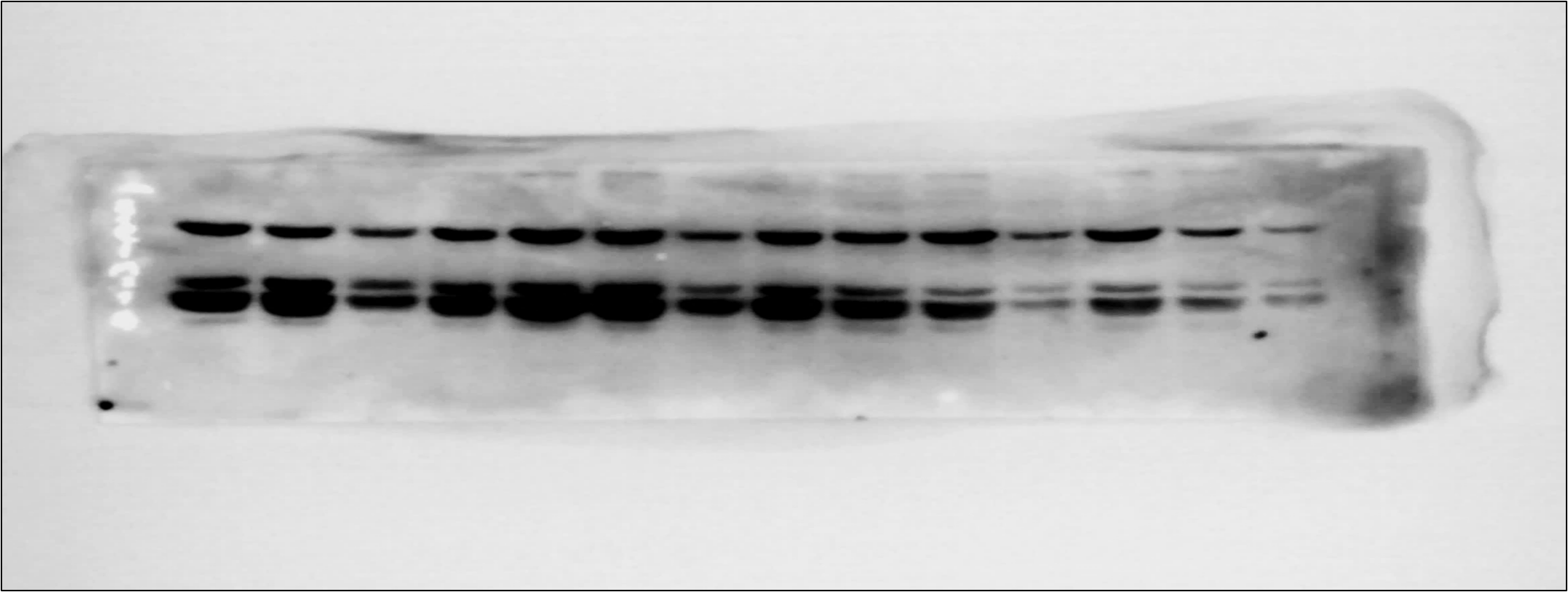

Supplement: Figure 4—figure supplement 1—source data 2. [file elife-108048-fig4-figsupp1-data2.zip › Figure 4-figure supplement 1/Figure S4 E-STING.tif]

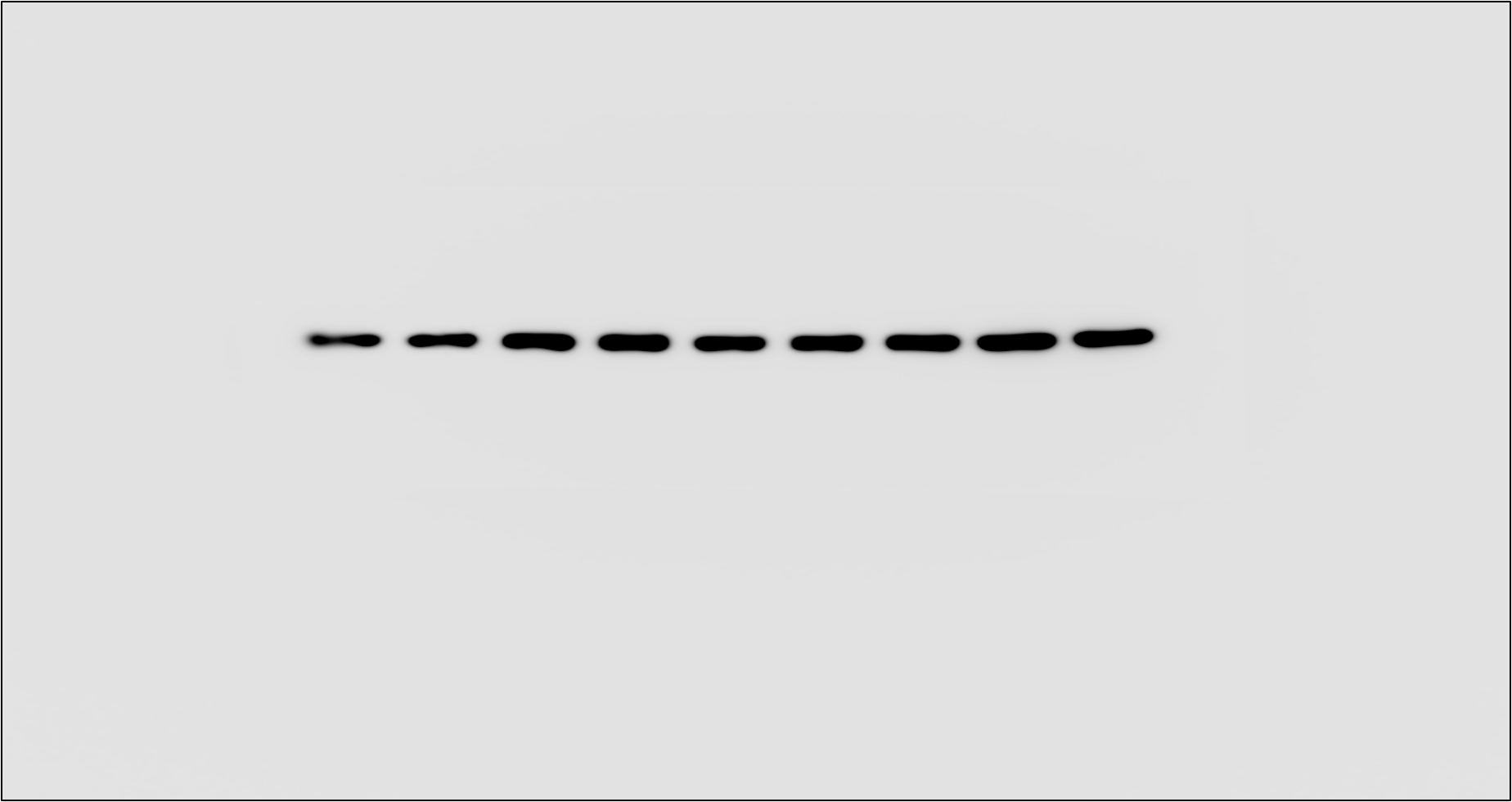

Supplement: Figure 5—source data 2. [file elife-108048-fig5-data2.zip › Figure 5/Figure 5 F-Actin.tif]

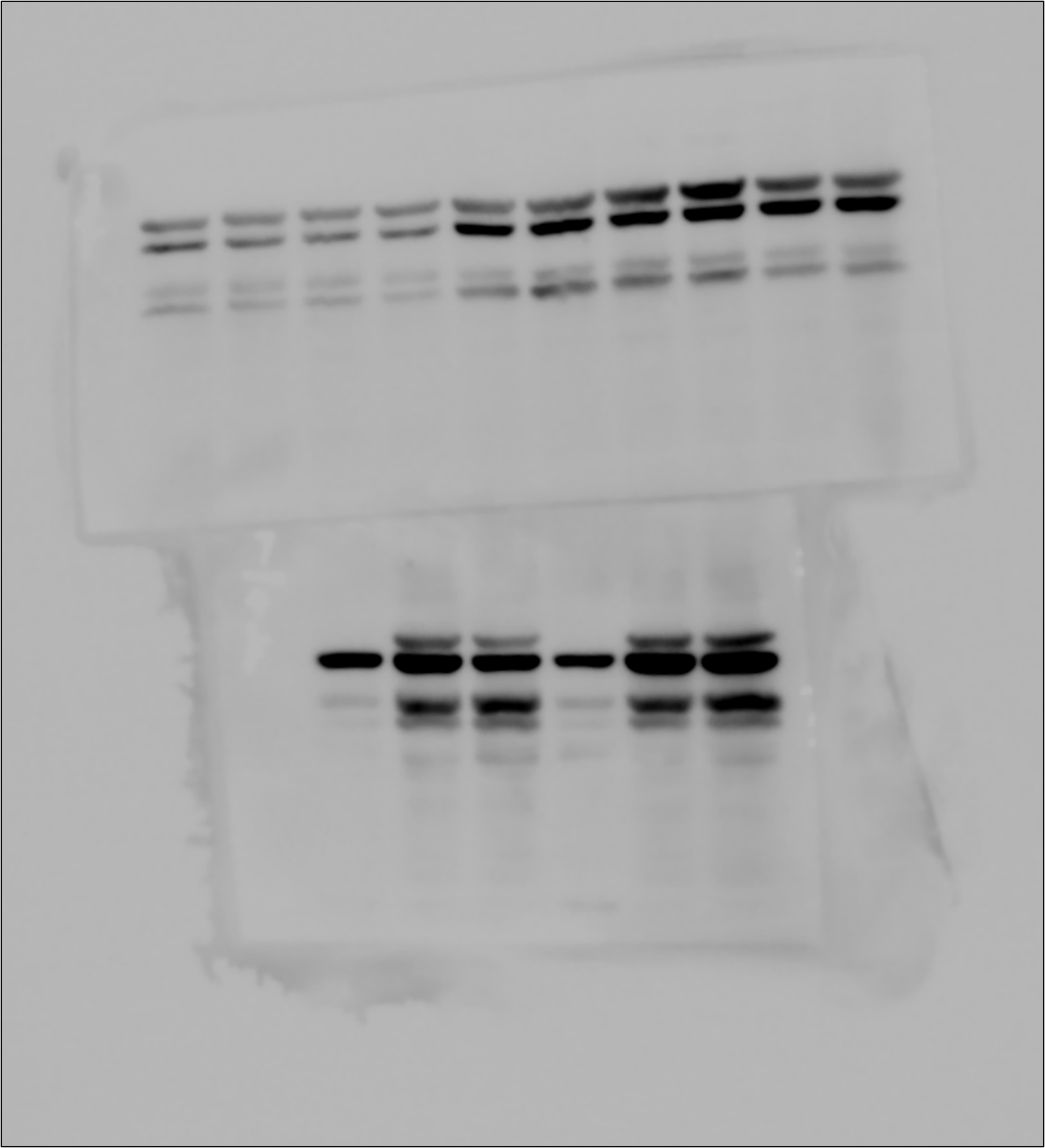

Supplement: Figure 5—source data 2. [file elife-108048-fig5-data2.zip › Figure 5/Figure 5 F-Flag.tif]

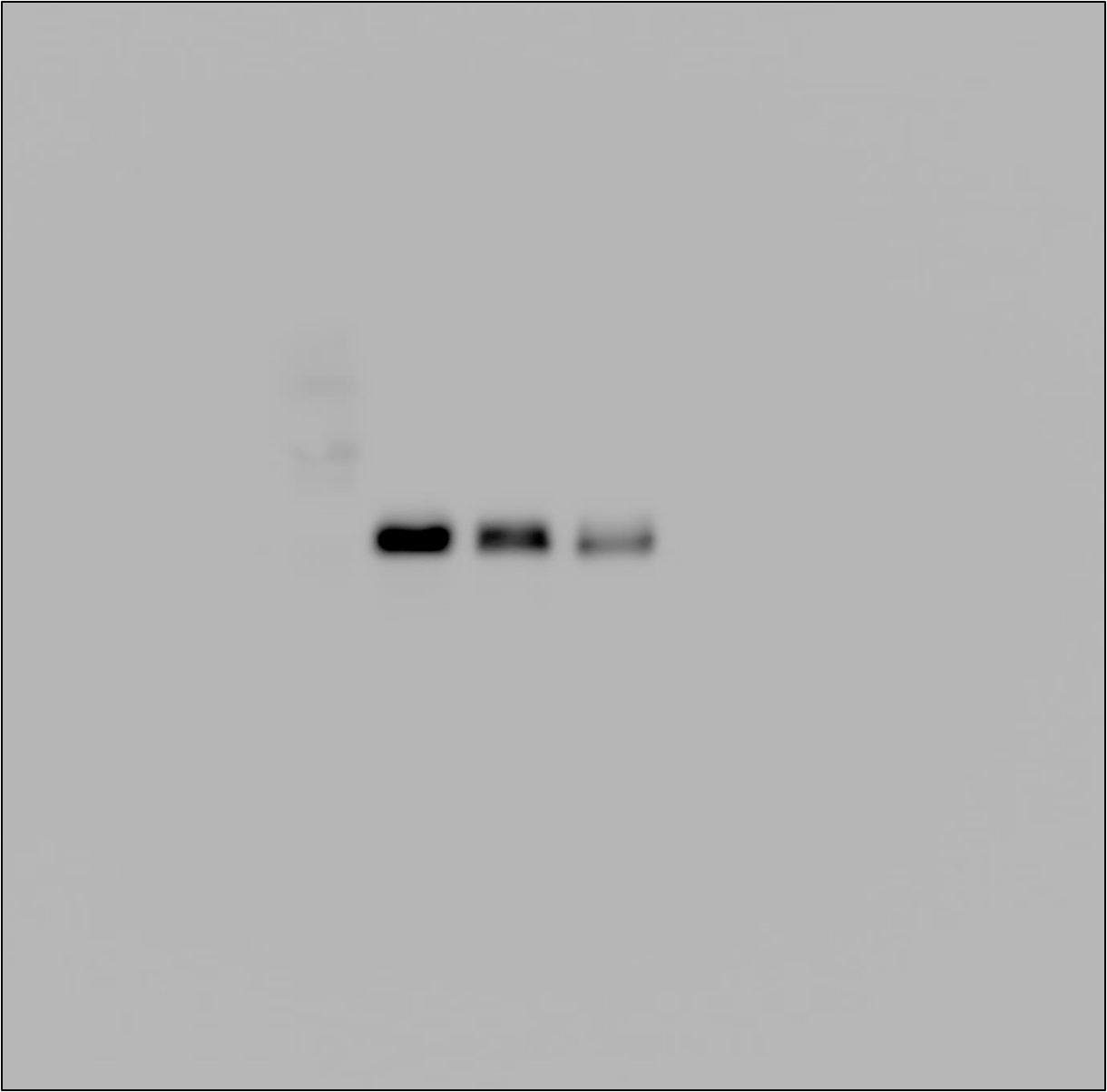

Supplement: Figure 5—source data 2. [file elife-108048-fig5-data2.zip › Figure 5/Figure 5 F-G.tif]

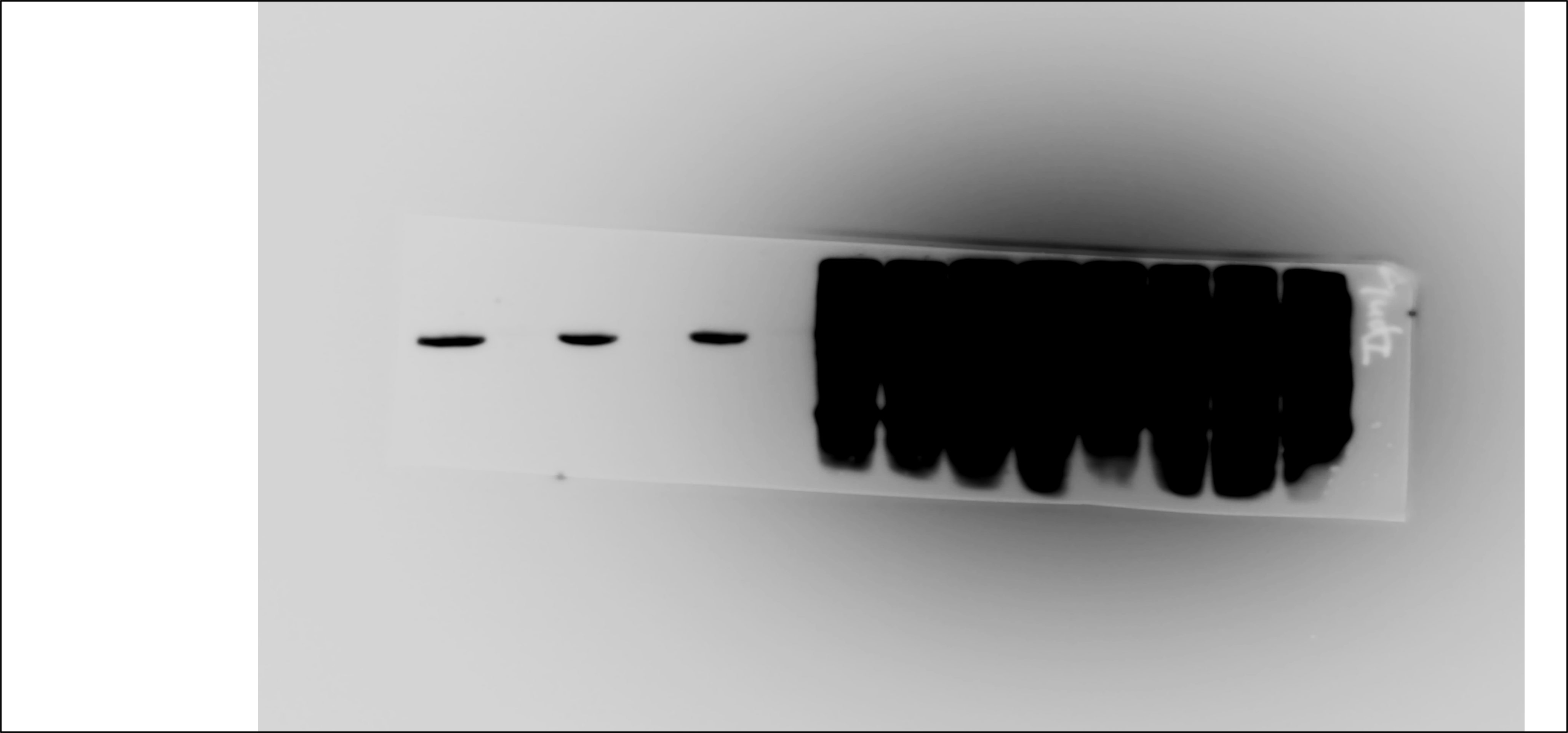

Supplement: Figure 5—source data 2. [file elife-108048-fig5-data2.zip › Figure 5/Figure 5 F-HA.tif]

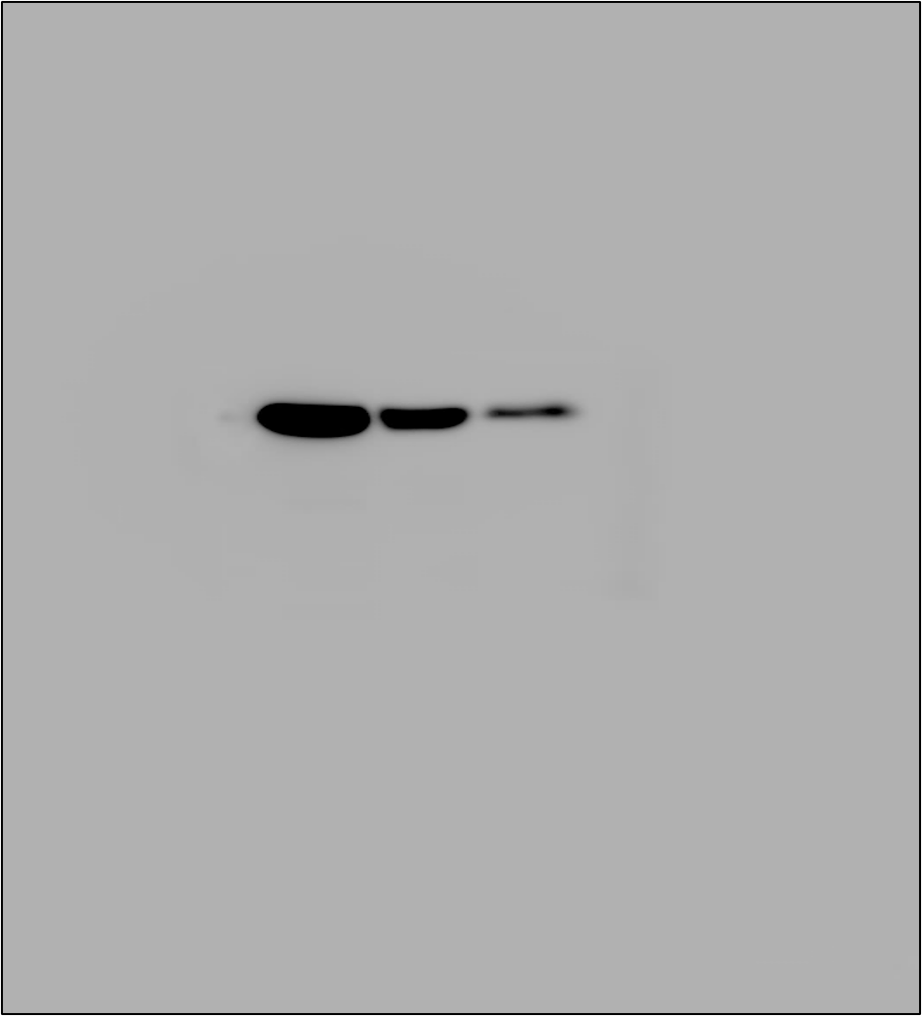

Supplement: Figure 5—source data 2. [file elife-108048-fig5-data2.zip › Figure 5/Figure 5 F-N.tif]

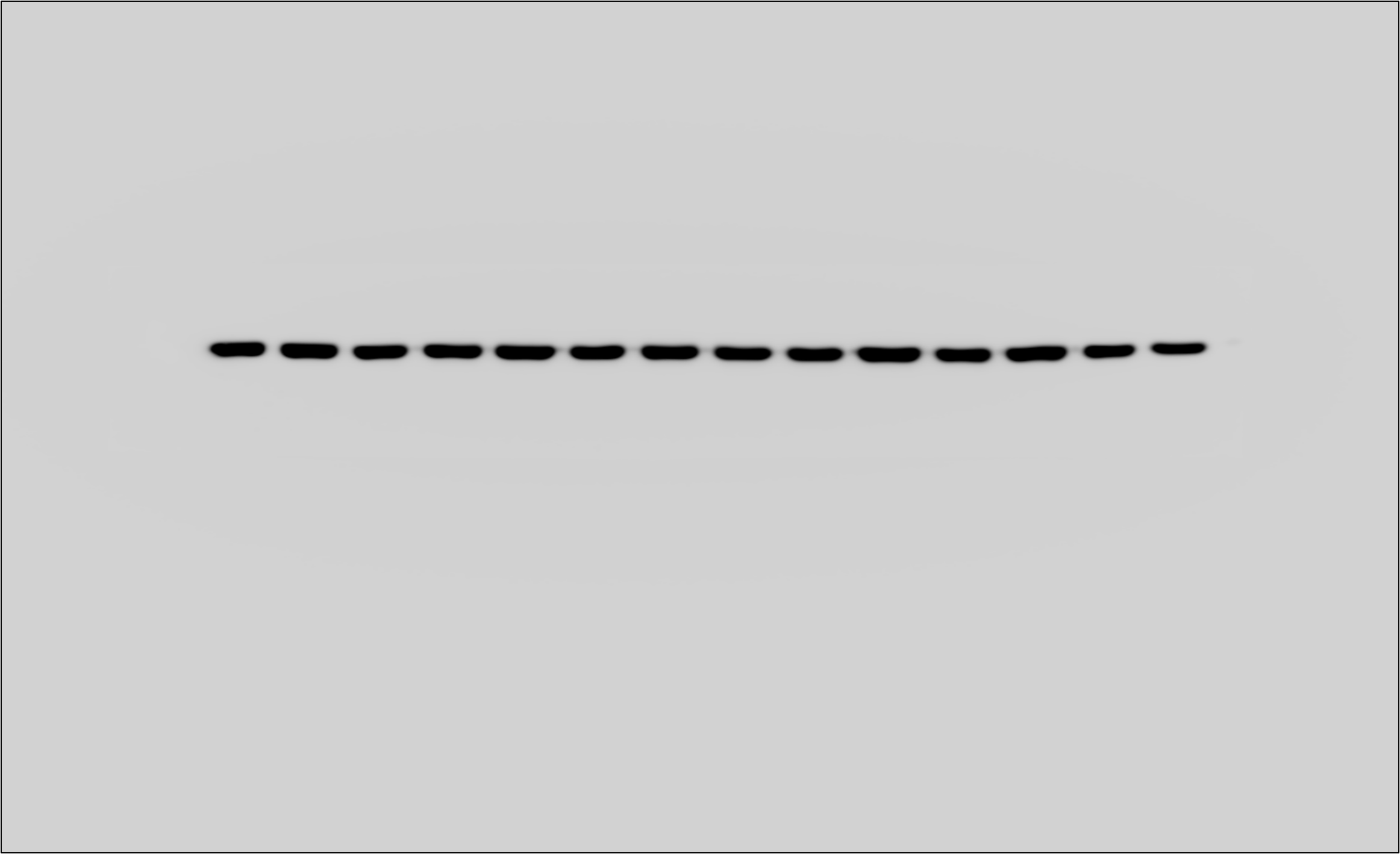

Supplement: Figure 5—source data 2. [file elife-108048-fig5-data2.zip › Figure 5/Figure 5 J-Actin.tif]

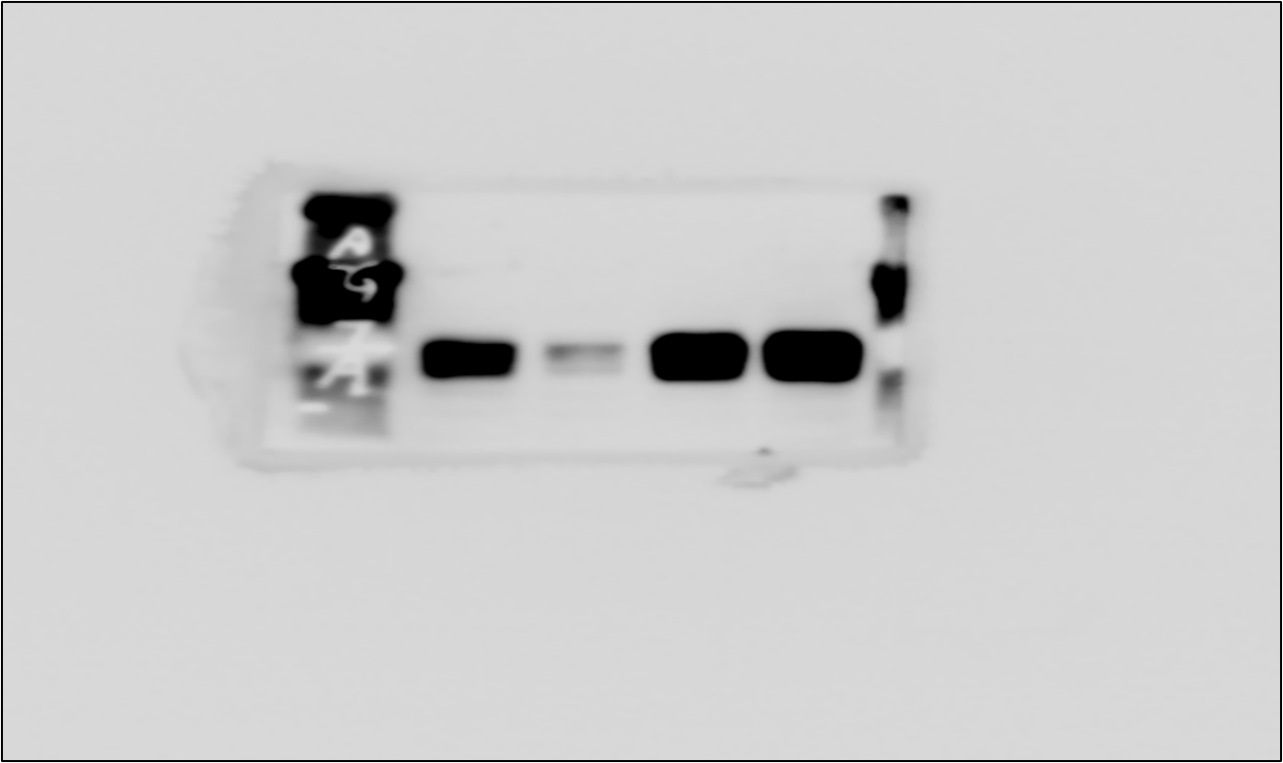

Supplement: Figure 5—source data 2. [file elife-108048-fig5-data2.zip › Figure 5/Figure 5 J-G.tif]

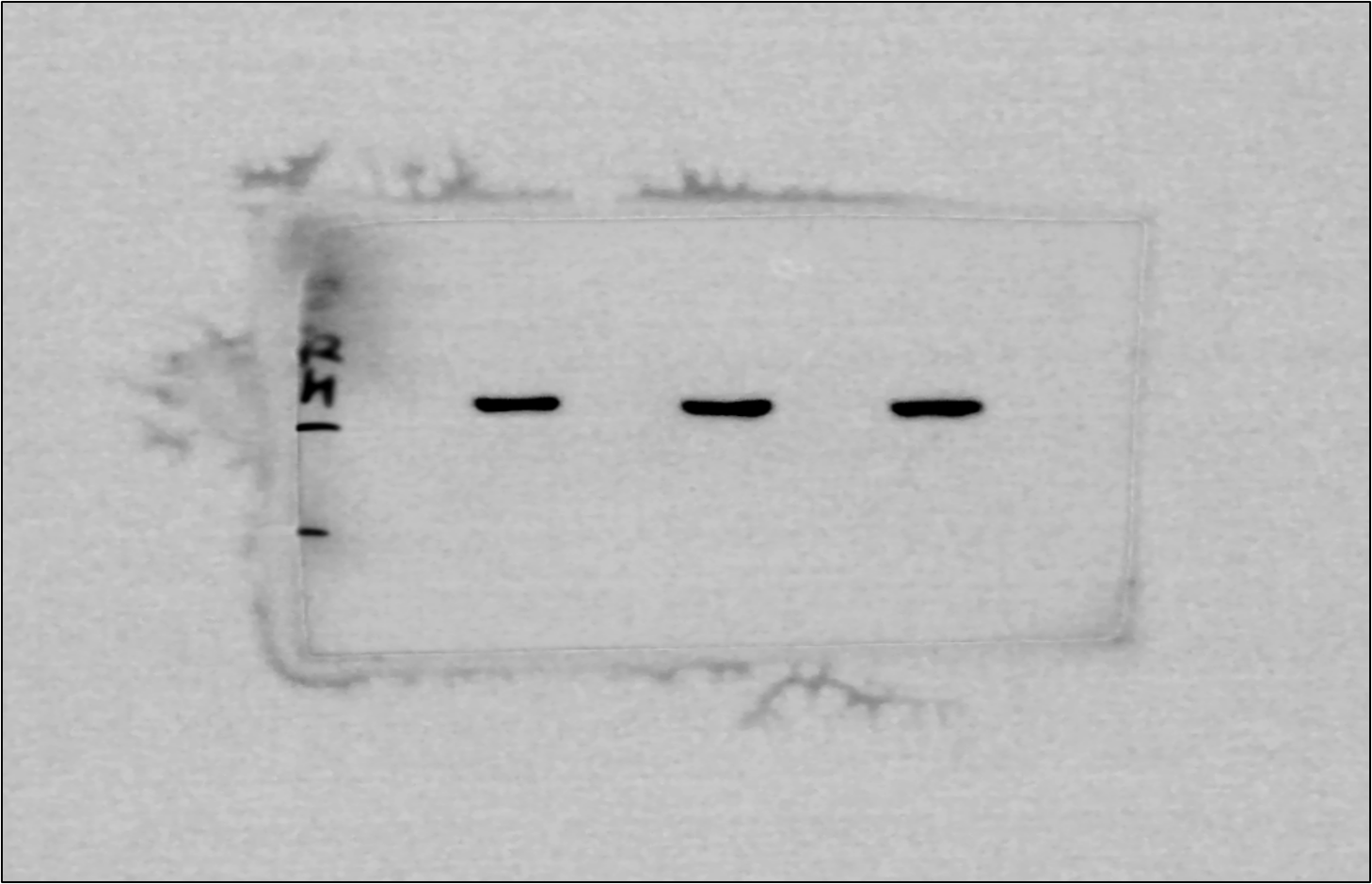

Supplement: Figure 5—source data 2. [file elife-108048-fig5-data2.zip › Figure 5/Figure 5 J-HA.tif]

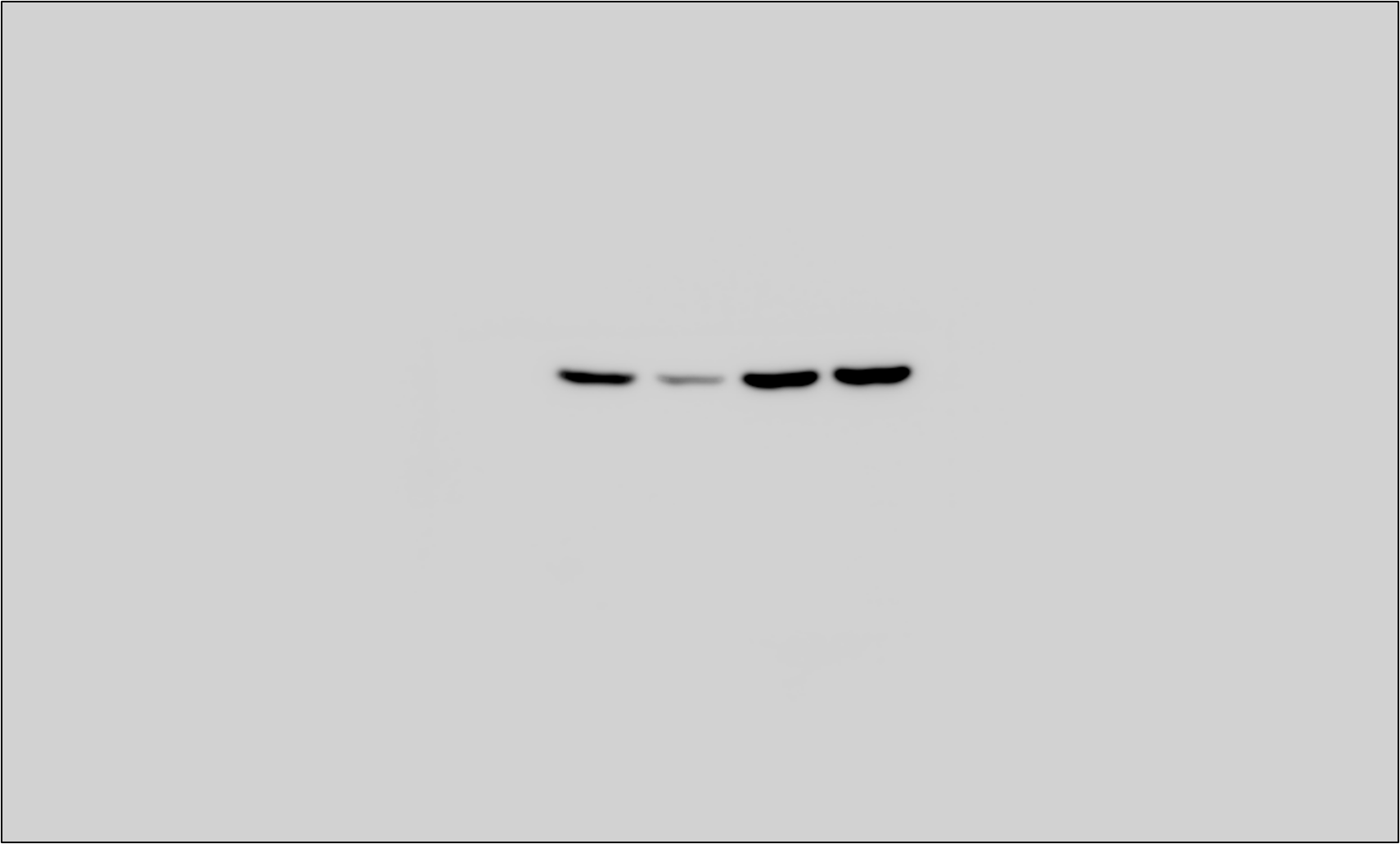

Supplement: Figure 5—source data 2. [file elife-108048-fig5-data2.zip › Figure 5/Figure 5 J-N.tif]

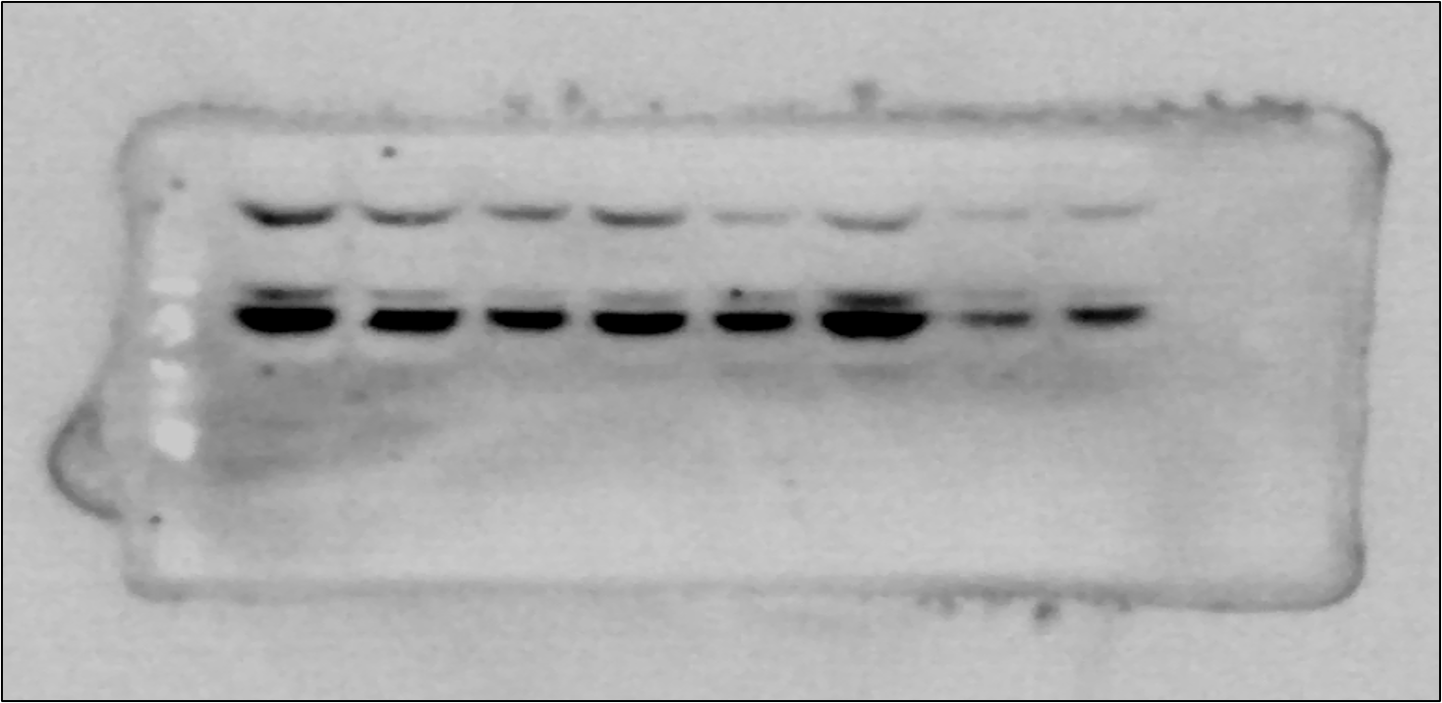

Supplement: Figure 5—source data 2. [file elife-108048-fig5-data2.zip › Figure 5/Figure 5 J-STING.tif]

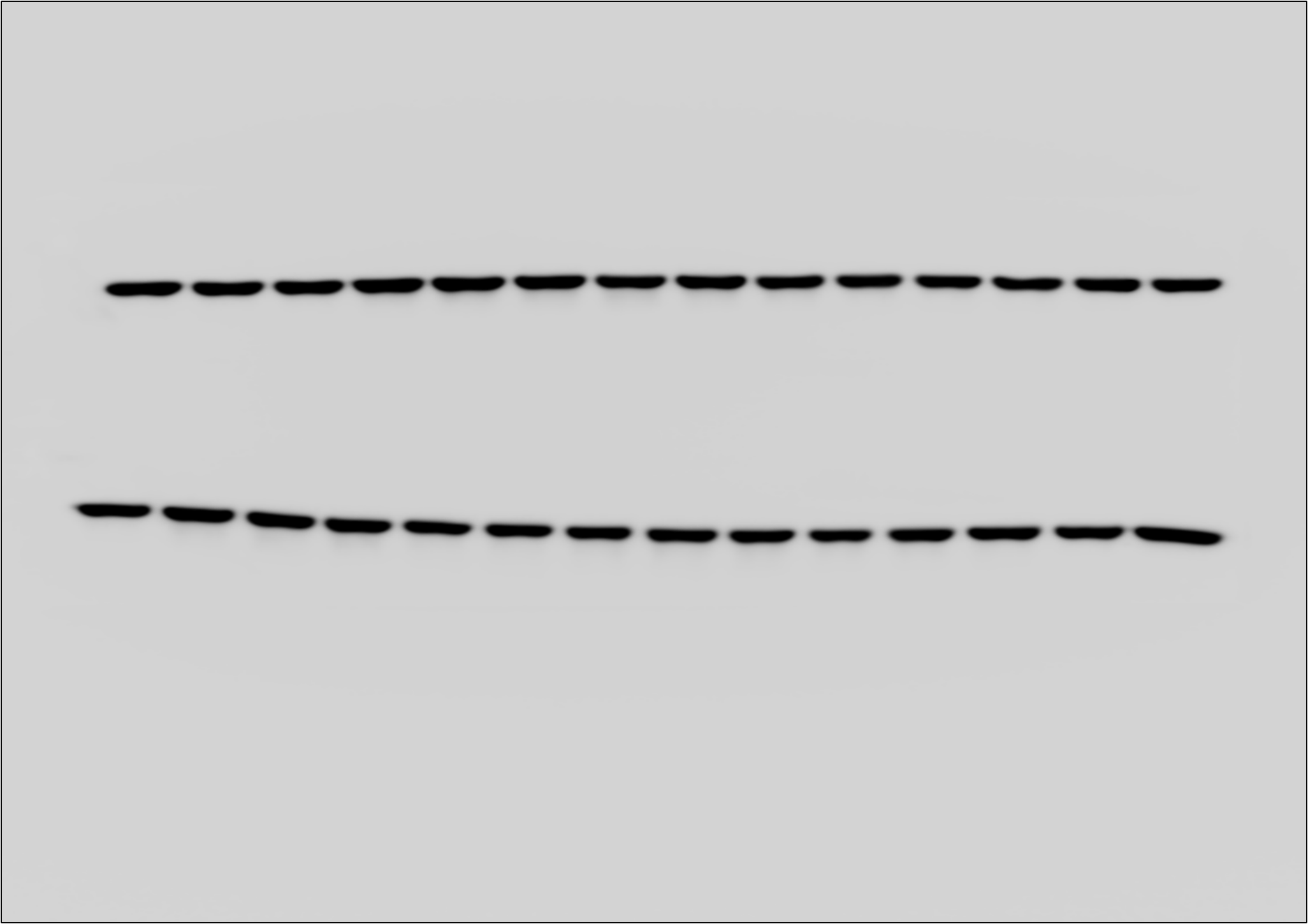

Supplement: Figure 5—figure supplement 1—source data 2. [file elife-108048-fig5-figsupp1-data2.zip › Figure 5-figure supplement 1/Figure S5-G-Actin-2.tif]

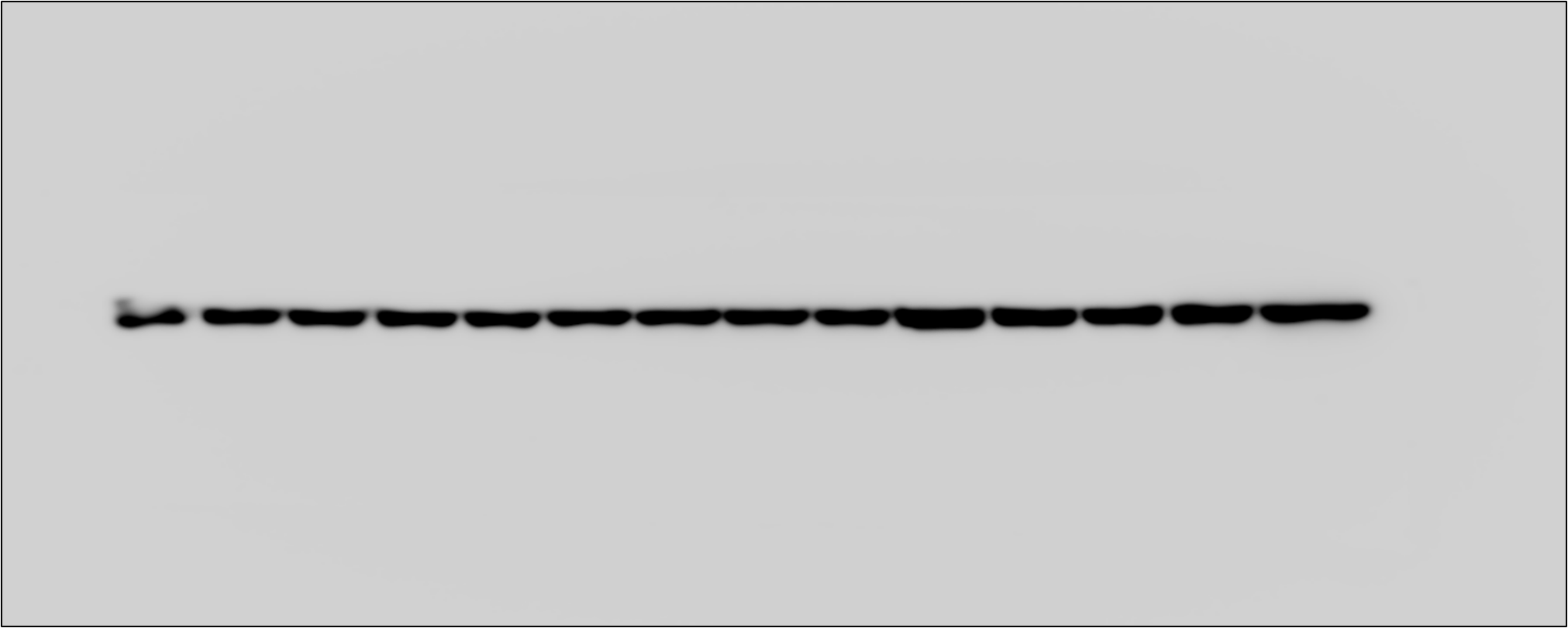

Supplement: Figure 5—figure supplement 1—source data 2. [file elife-108048-fig5-figsupp1-data2.zip › Figure 5-figure supplement 1/Figure S5-G-Actin.tif]

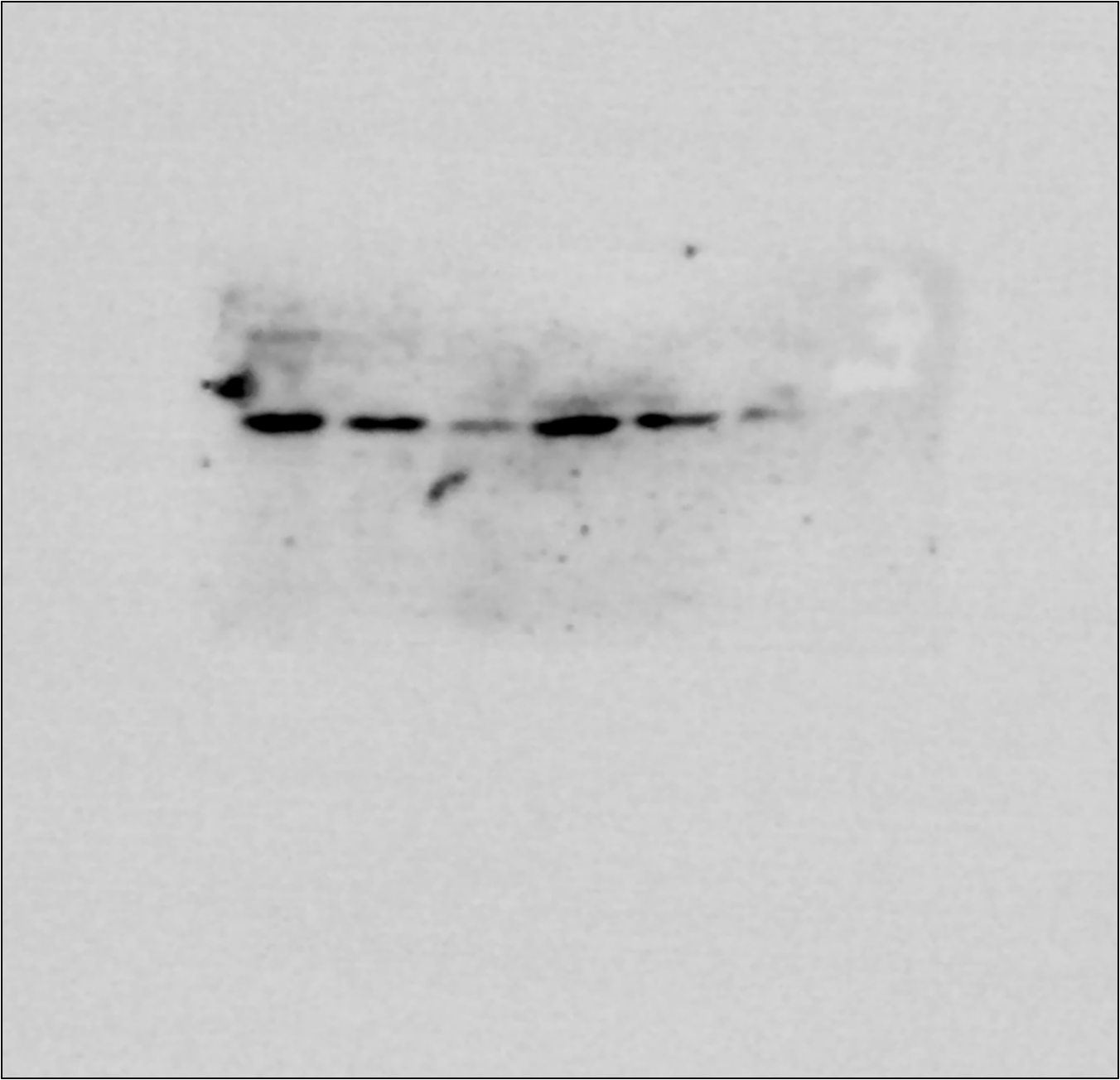

Supplement: Figure 5—figure supplement 1—source data 2. [file elife-108048-fig5-figsupp1-data2.zip › Figure 5-figure supplement 1/Figure S5-G-Myc.tif]

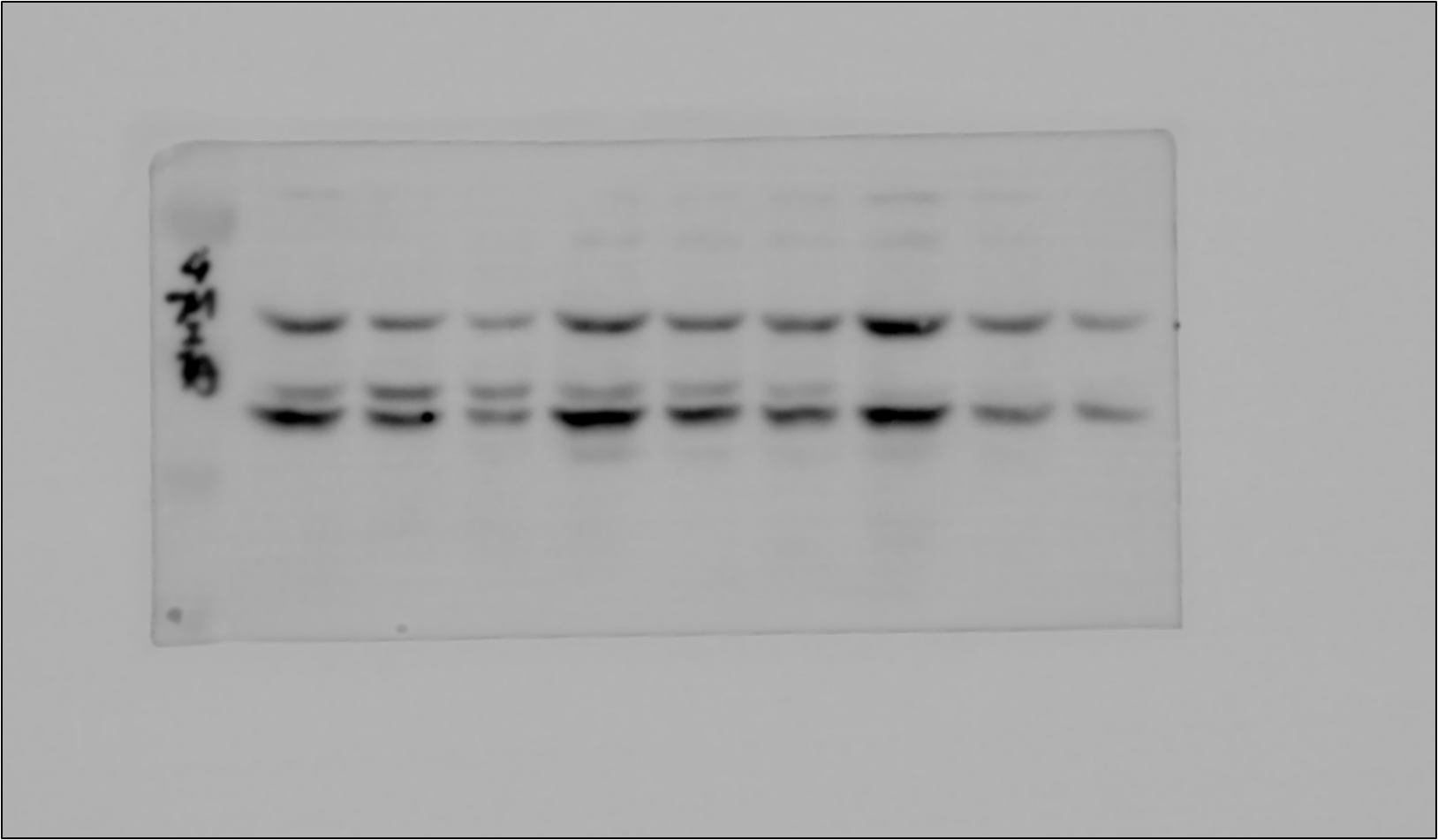

Supplement: Figure 5—figure supplement 1—source data 2. [file elife-108048-fig5-figsupp1-data2.zip › Figure 5-figure supplement 1/Figure S5-G-STING.tif]

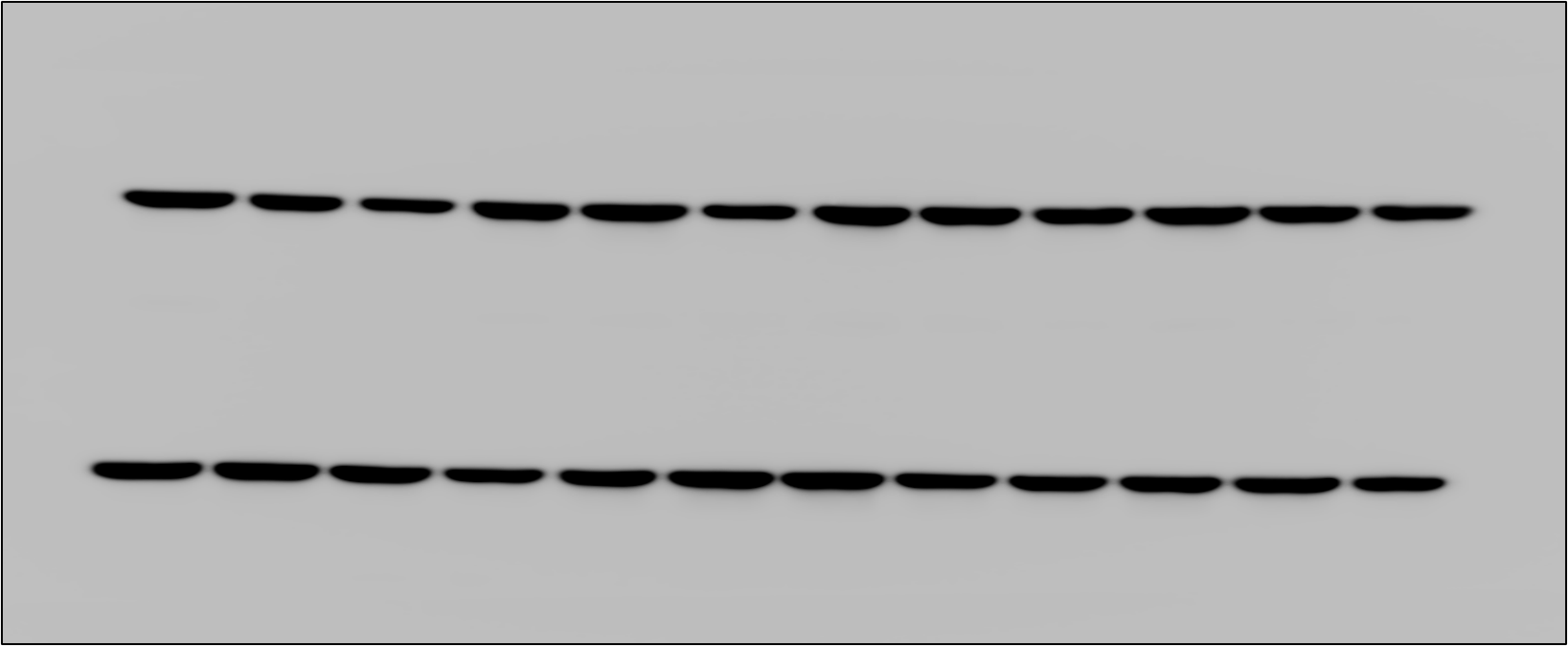

Supplement: Figure 6—source data 2. [file elife-108048-fig6-data2.zip › Figure 6/Figure 6 A-Actin-2.tif]

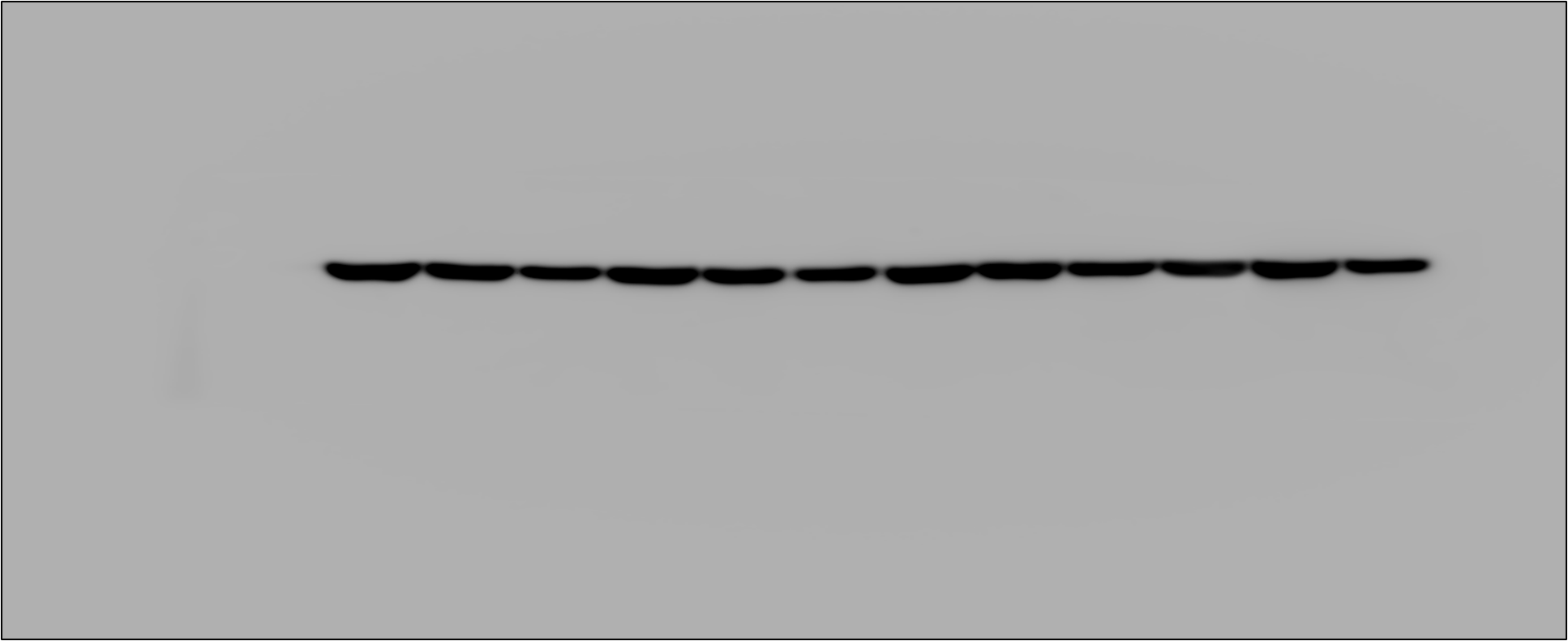

Supplement: Figure 6—source data 2. [file elife-108048-fig6-data2.zip › Figure 6/Figure 6 A-Actin-3.tif]
