# Supplementary material for: Male-biased Cyp17a2 orchestrates antiviral sexual dimorphism in fish via STING stabilization and viral protein degradation
Source: eLife. 2026 Feb 18;14:RP108048. doi: 10.7554/eLife.108048 (PMC12916102; doi:10.7554/eLife.108048)
Supplement: Figure 2—source data 1. [file elife-108048-fig2-data1.pdf]

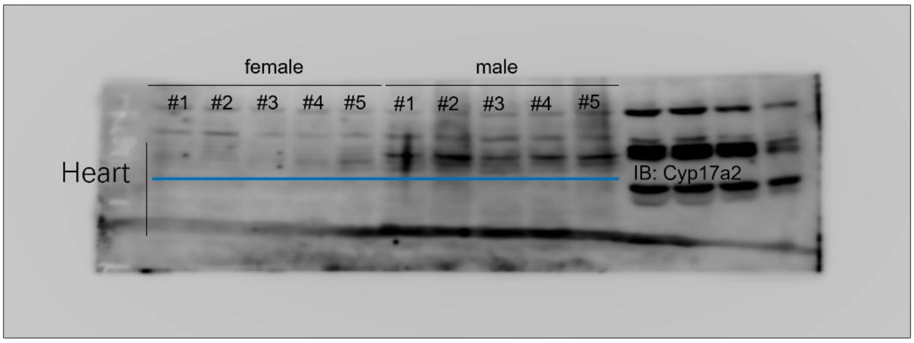
female
male
#1 #2 #3 #4 #5 #1 #2 #3 #4 #5
Heart
IB: Cyp17a2

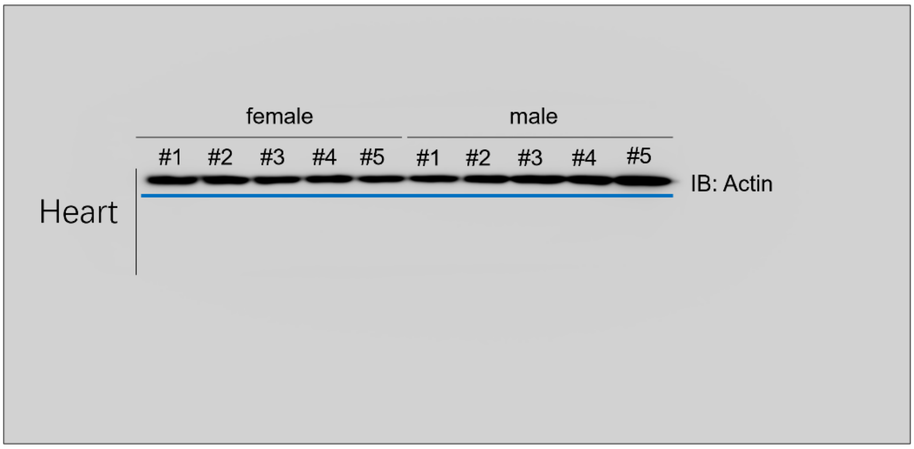
female
male
#1 #2 #3 #4 #5 #1 #2 #3 #4 #5
Heart
IB: Actin

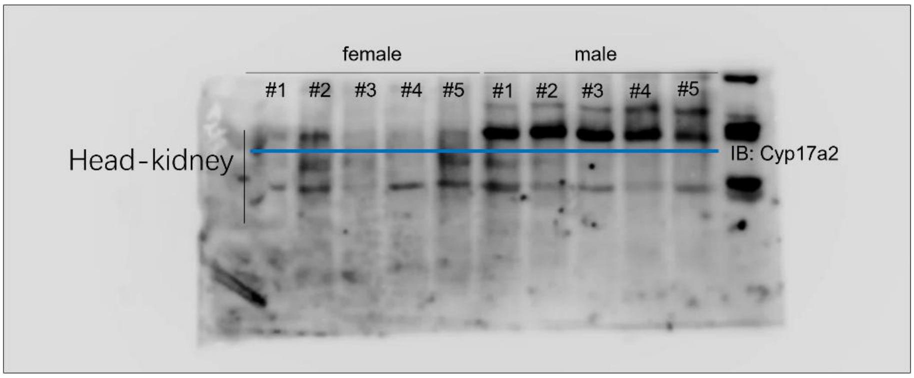
female
male
#1 #2 #3 #4 #5 #1 #2 #3 #4 #5
Head-kidney
IB: Cyp17a2

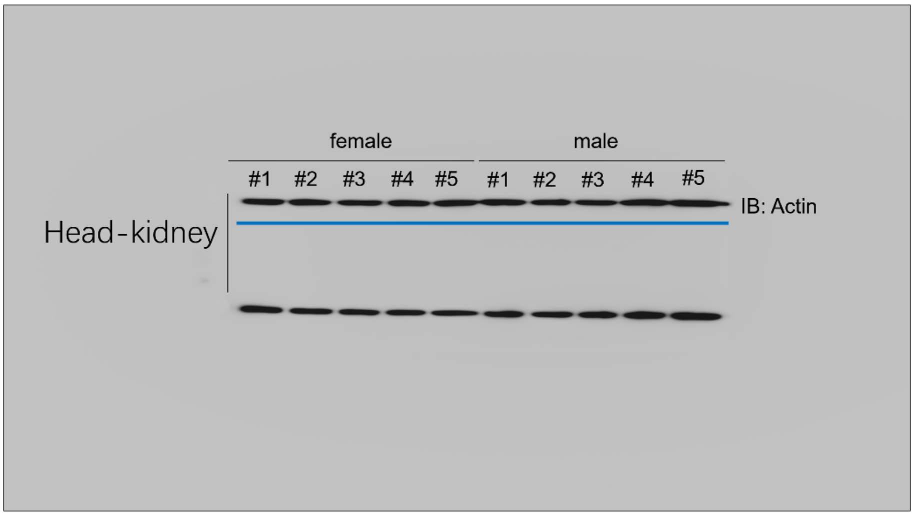
female
male
#1 #2 #3 #4 #5 #1 #2 #3 #4 #5
Head-kidney
IB: Actin

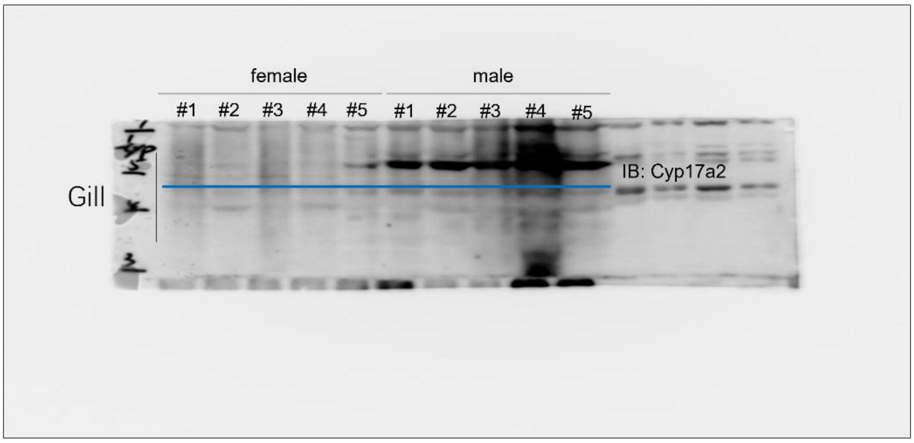
female
male
#1 #2 #3 #4 #5 #1 #2 #3 #4 #5
Gill
IB: Cyp17a2

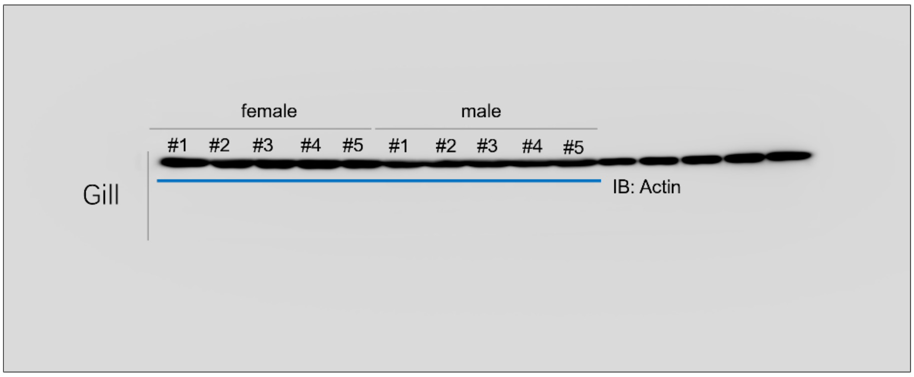
female
male
#1 #2 #3 #4 #5 #1 #2 #3 #4 #5
Gill
IB: Actin

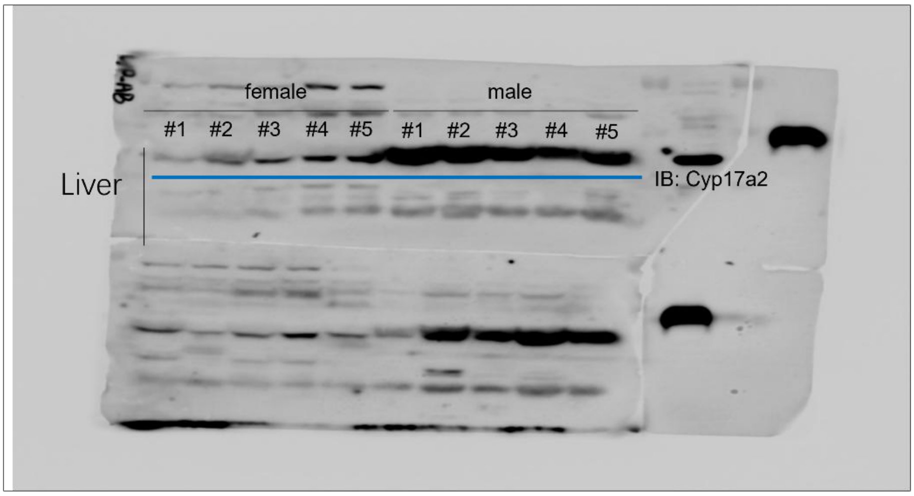
female
male
#1 #2 #3 #4 #5 #1 #2 #3 #4 #5
Liver
IB: Cyp17a2

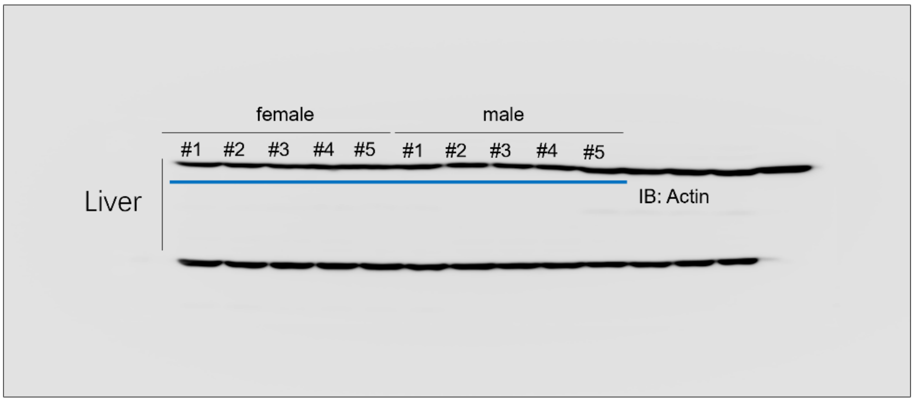
female
male
#1 #2 #3 #4 #5 #1 #2 #3 #4 #5
Liver
IB: Actin

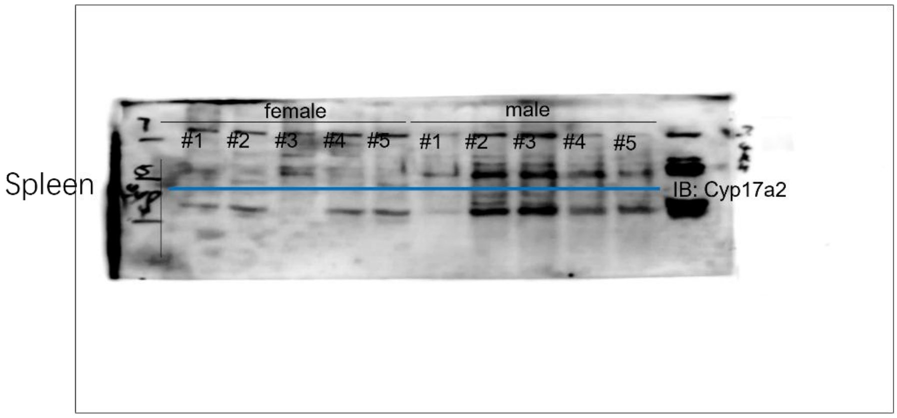
Spleen
female
male
#1 #2 #3 #4 #5 #1 #2 #3 #4 #5
IB: Cyp17a2

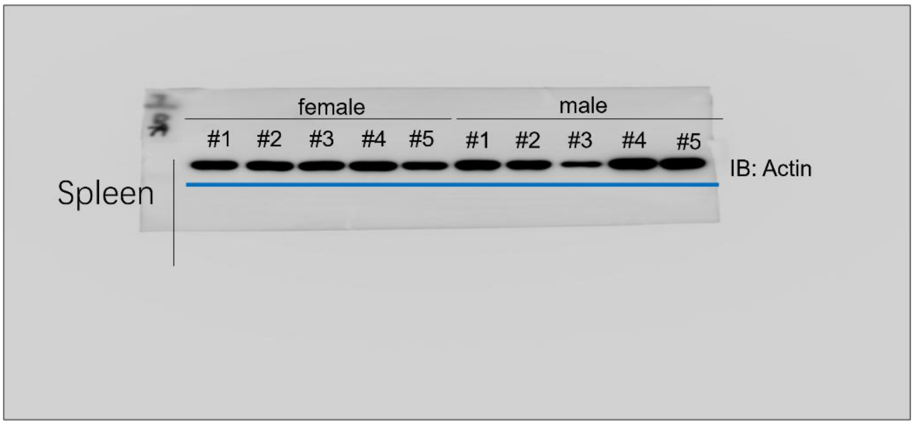
Spleen
female
male
#1 #2 #3 #4 #5 #1 #2 #3 #4 #5
IB: Actin

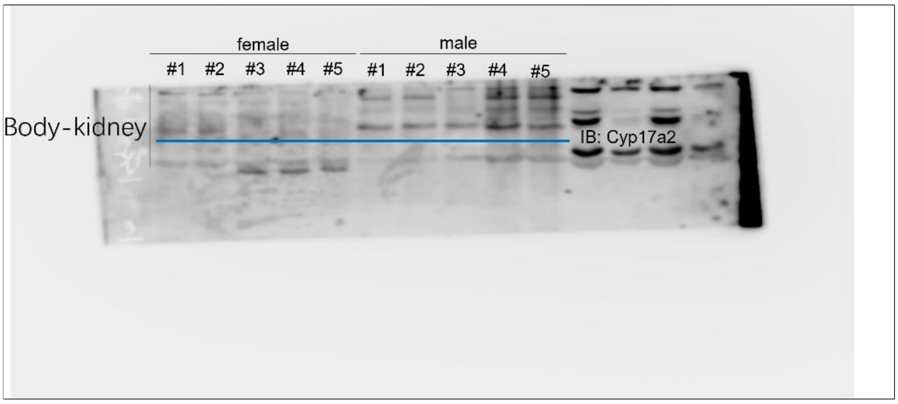
Body-kidney
female
male
#1 #2 #3 #4 #5 #1 #2 #3 #4 #5
IB: Cyp17a2

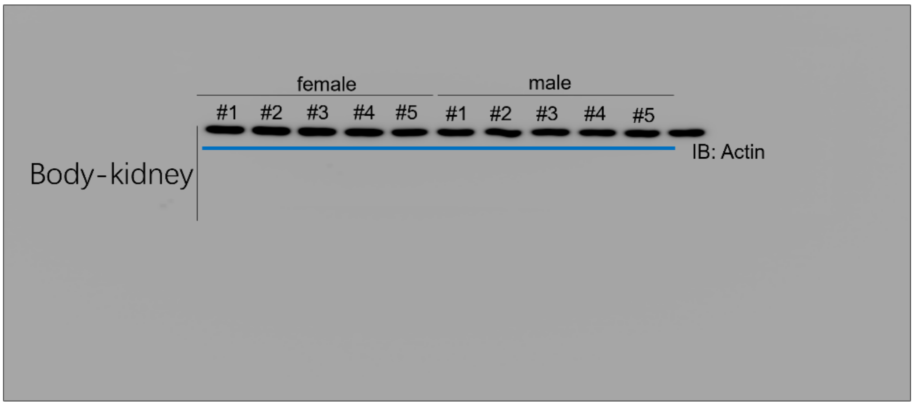
Body-kidney
female
male
#1 #2 #3 #4 #5 #1 #2 #3 #4 #5
IB: Actin

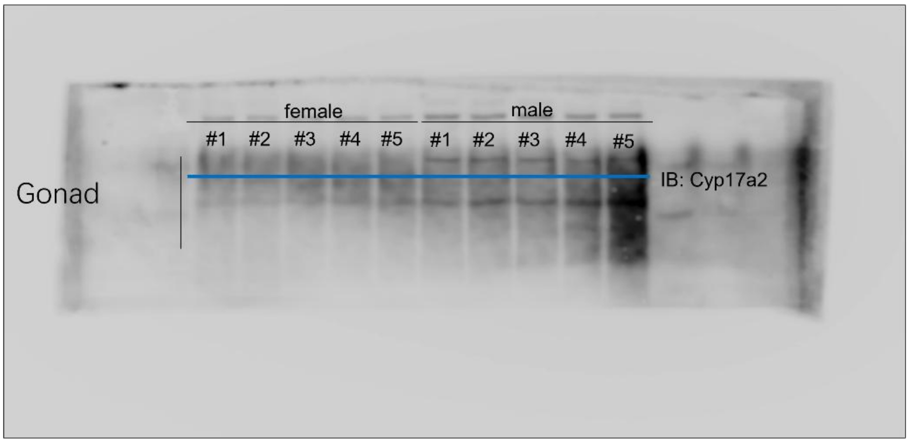
female
male
#1 #2 #3 #4 #5 #1 #2 #3 #4 #5
Gonad
IB: Cyp17a2

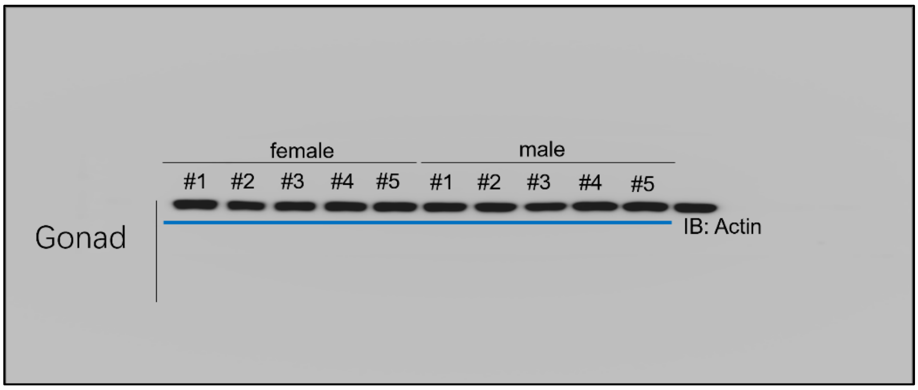
female
male
#1 #2 #3 #4 #5 #1 #2 #3 #4 #5
Gonad
IB: Actin

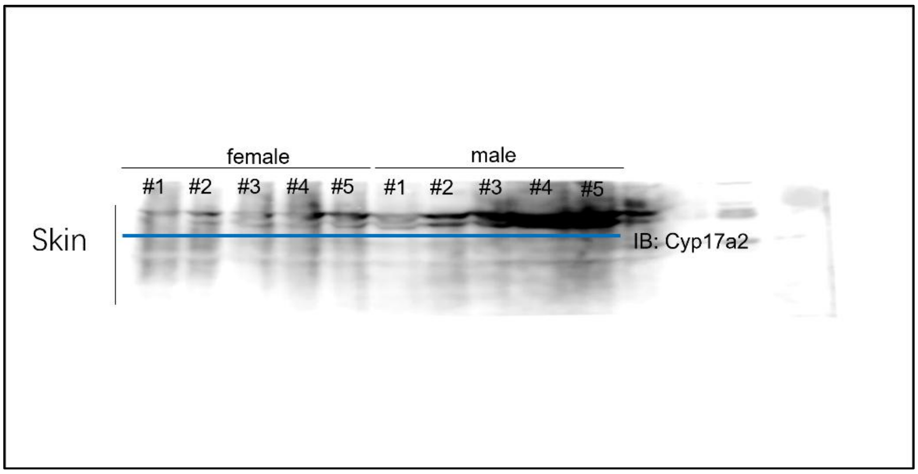
female
male
#1 #2 #3 #4 #5 #1 #2 #3 #4 #5
Skin
IB: Cyp17a2

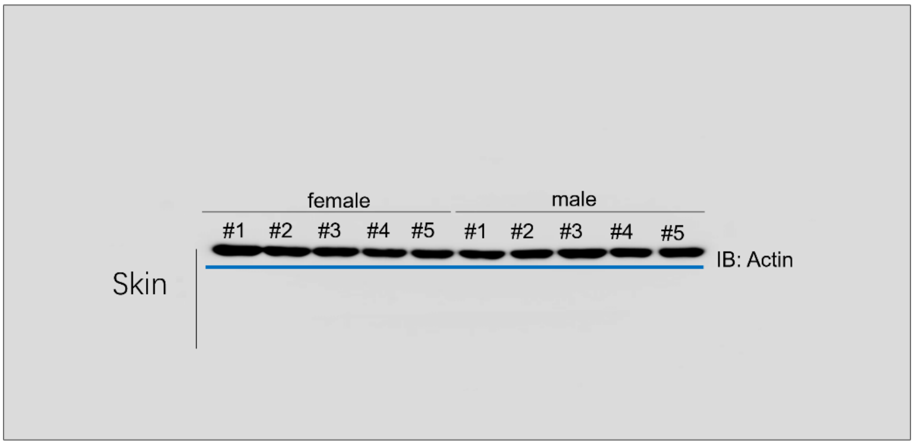
female
male
#1 #2 #3 #4 #5 #1 #2 #3 #4 #5
Skin
IB: Actin

Figure 2, Source Data 1. Original membranes corresponding to Figure 2, panel D. Each membrane is labelled with the relevant information. The blue lines indicate the corresponding bands.
