# Supplementary material for: Male-biased Cyp17a2 orchestrates antiviral sexual dimorphism in fish via STING stabilization and viral protein degradation
Source: eLife. 2026 Feb 18;14:RP108048. doi: 10.7554/eLife.108048 (PMC12916102; doi:10.7554/eLife.108048)
Supplement: Figure 4—figure supplement 1—source data 1. [file elife-108048-fig4-figsupp1-data1.pdf]

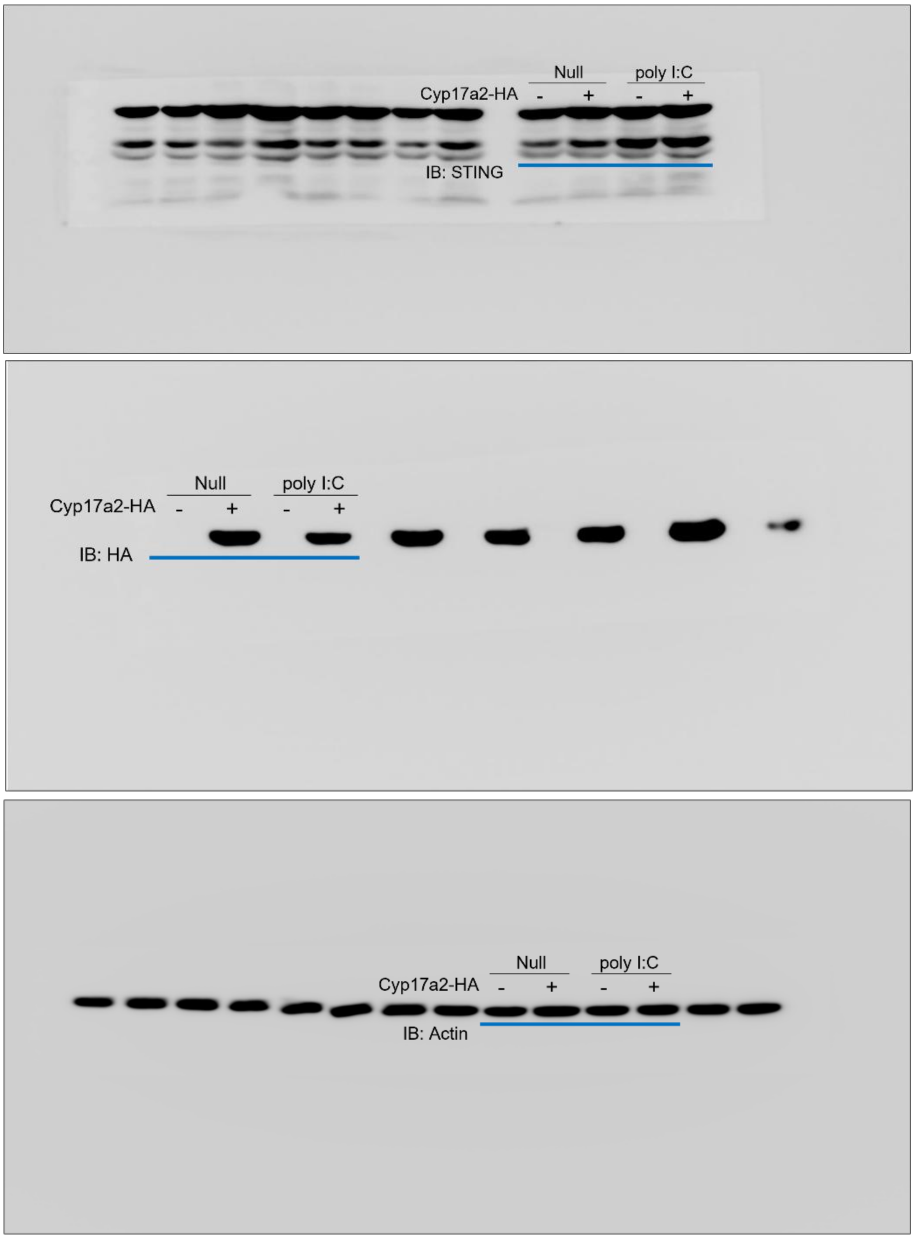

Figure 4-figure supplement 1, Source Data 1. Original membranes corresponding to Figure 4-figure supplement 1, panel C. Each membrane is labelled with the relevant information. The blue lines indicate the corresponding bands.

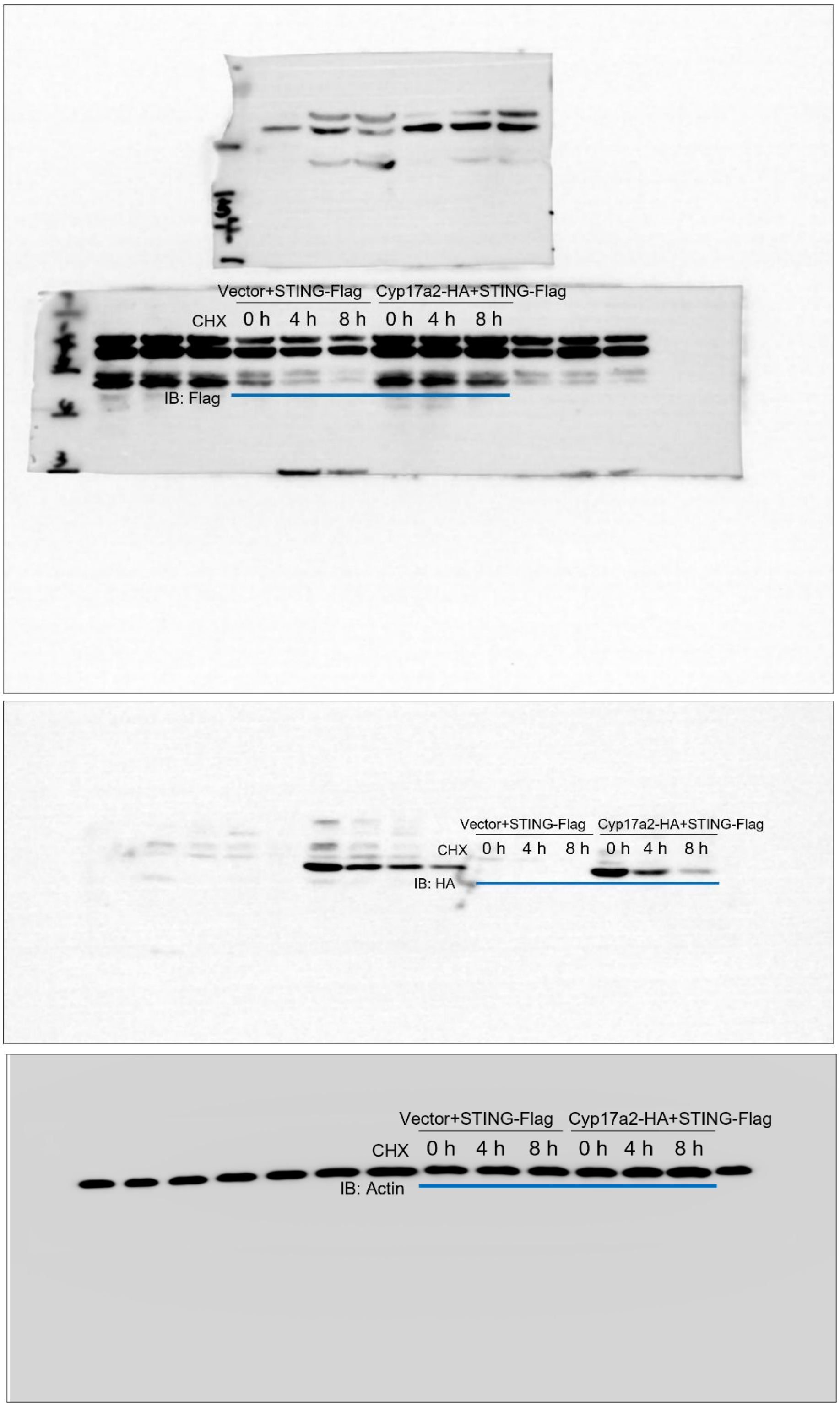

Figure 4-figure supplement 1, Source Data 1. Original membranes corresponding to Figure 4-figure supplement 1, panel D. Each membrane is labelled with the relevant information. The blue lines indicate the corresponding bands.

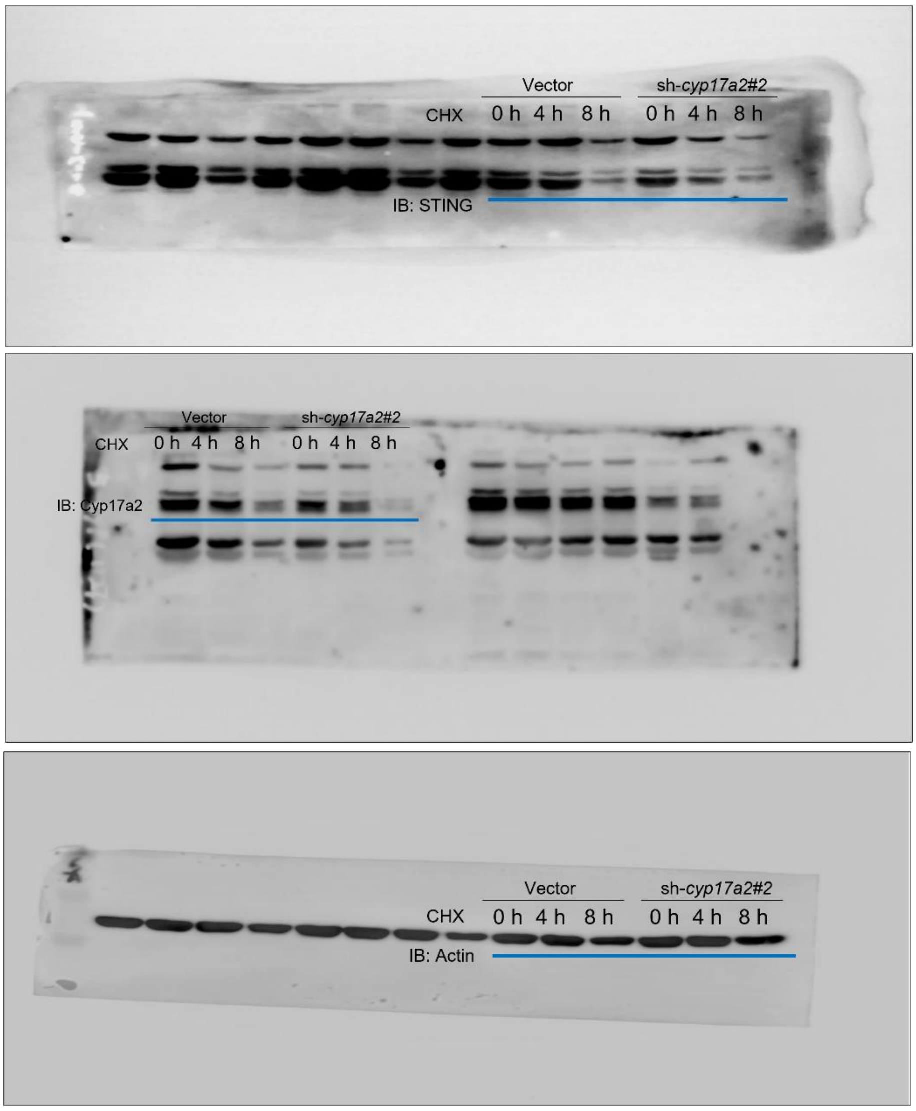

Figure 4-figure supplement 1, Source Data 1. Original membranes corresponding to Figure 4-figure supplement 1, panel E. Each membrane is labelled with the relevant information. The blue lines indicate the corresponding bands.
