# Supplementary material for: Male-biased Cyp17a2 orchestrates antiviral sexual dimorphism in fish via STING stabilization and viral protein degradation
Source: eLife. 2026 Feb 18;14:RP108048. doi: 10.7554/eLife.108048 (PMC12916102; doi:10.7554/eLife.108048)
Supplement: Figure 6—source data 1. [file elife-108048-fig6-data1.pdf]

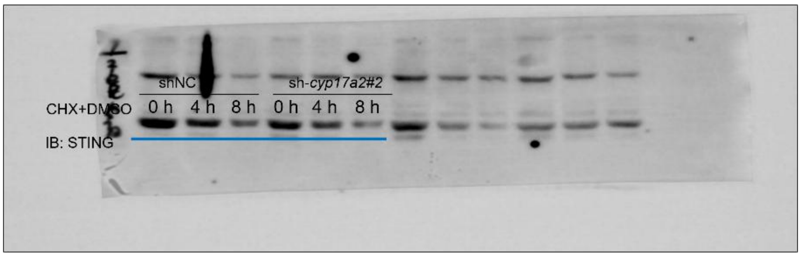
shNC
sh-cyp17a2#2
CHX+DMSO
0 h 4 h 8 h 0 h 4 h 8 h
IB: STING

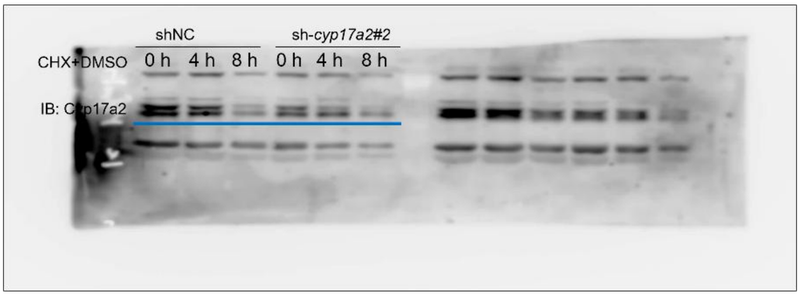
shNC
sh-cyp17a2#2
CHX+DMSO
0 h 4 h 8 h 0 h 4 h 8 h
IB: Cyp17a2

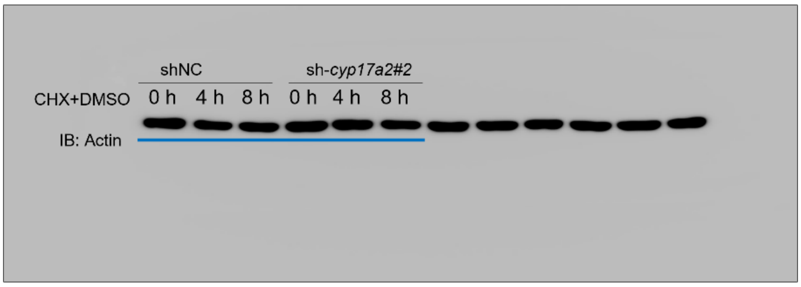
shNC
sh-cyp17a2#2
CHX+DMSO
0 h 4 h 8 h 0 h 4 h 8 h
IB: Actin

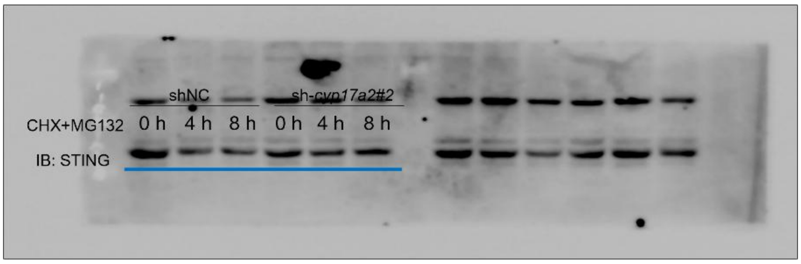
shNC
sh-cyp17a2#2
CHX+MG132
0 h 4 h 8 h 0 h 4 h 8 h
IB: STING

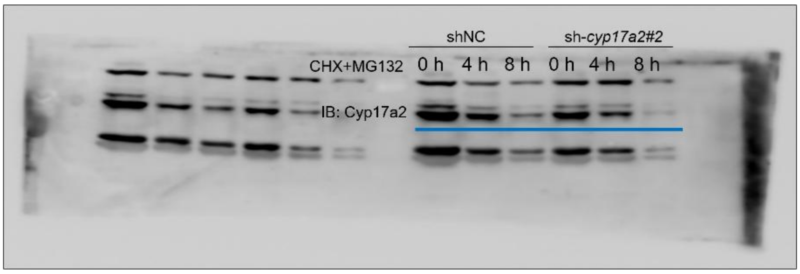
shNC
sh-cyp17a2#2
CHX+MG132
0 h 4 h 8 h 0 h 4 h 8 h
IB: Cyp17a2

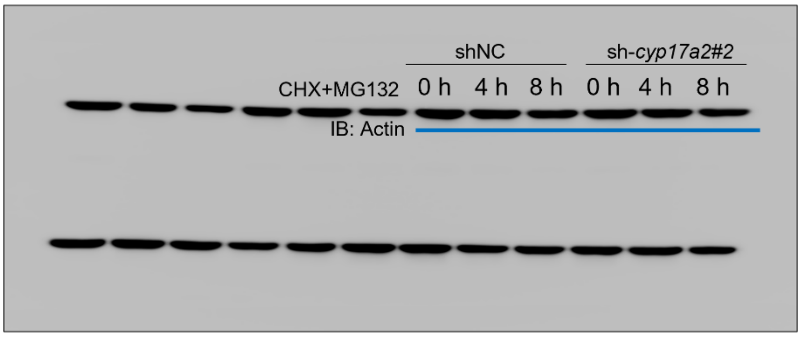
shNC
sh-cyp17a2#2
CHX+MG132
0 h 4 h 8 h 0 h 4 h 8 h
IB: Actin

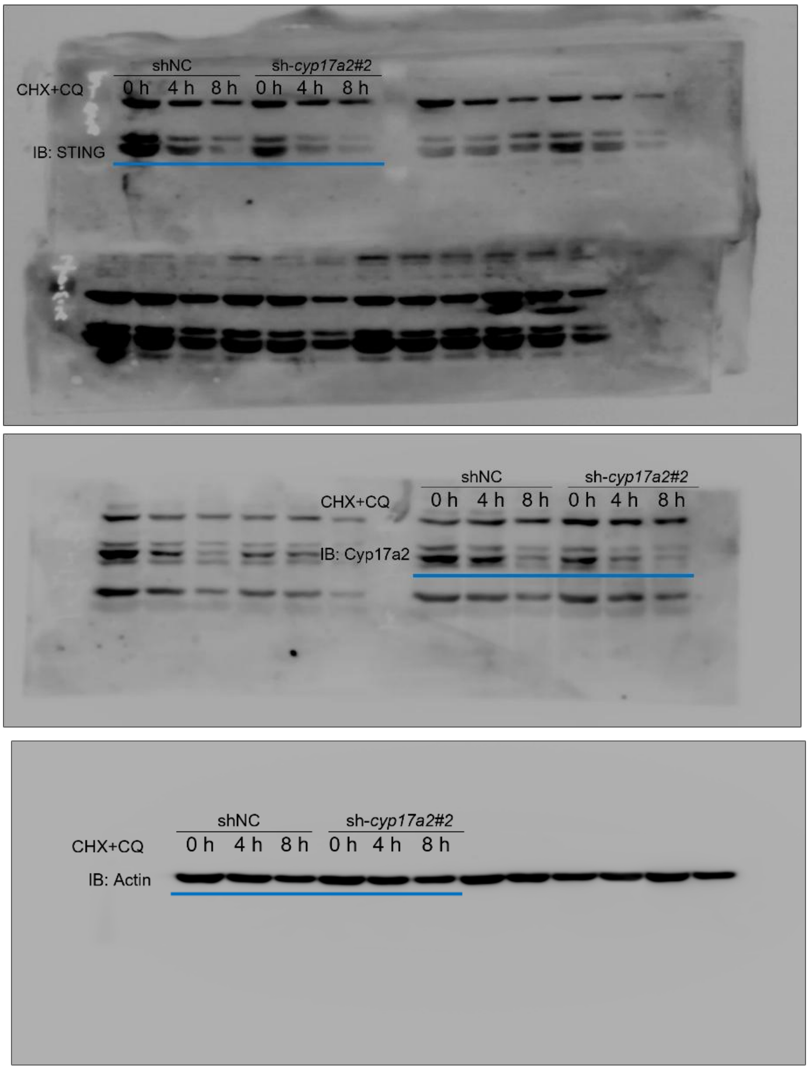

Figure 6, Source Data 1. Original membranes corresponding to Figure 6, panel A. Each membrane is labelled with the relevant information. The blue lines indicate the corresponding bands.

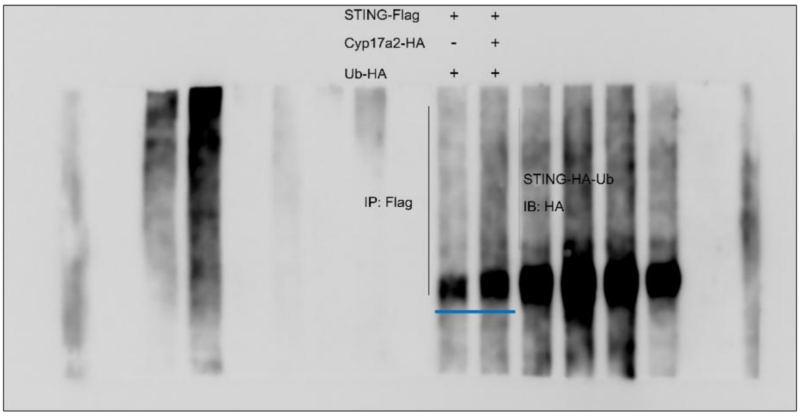

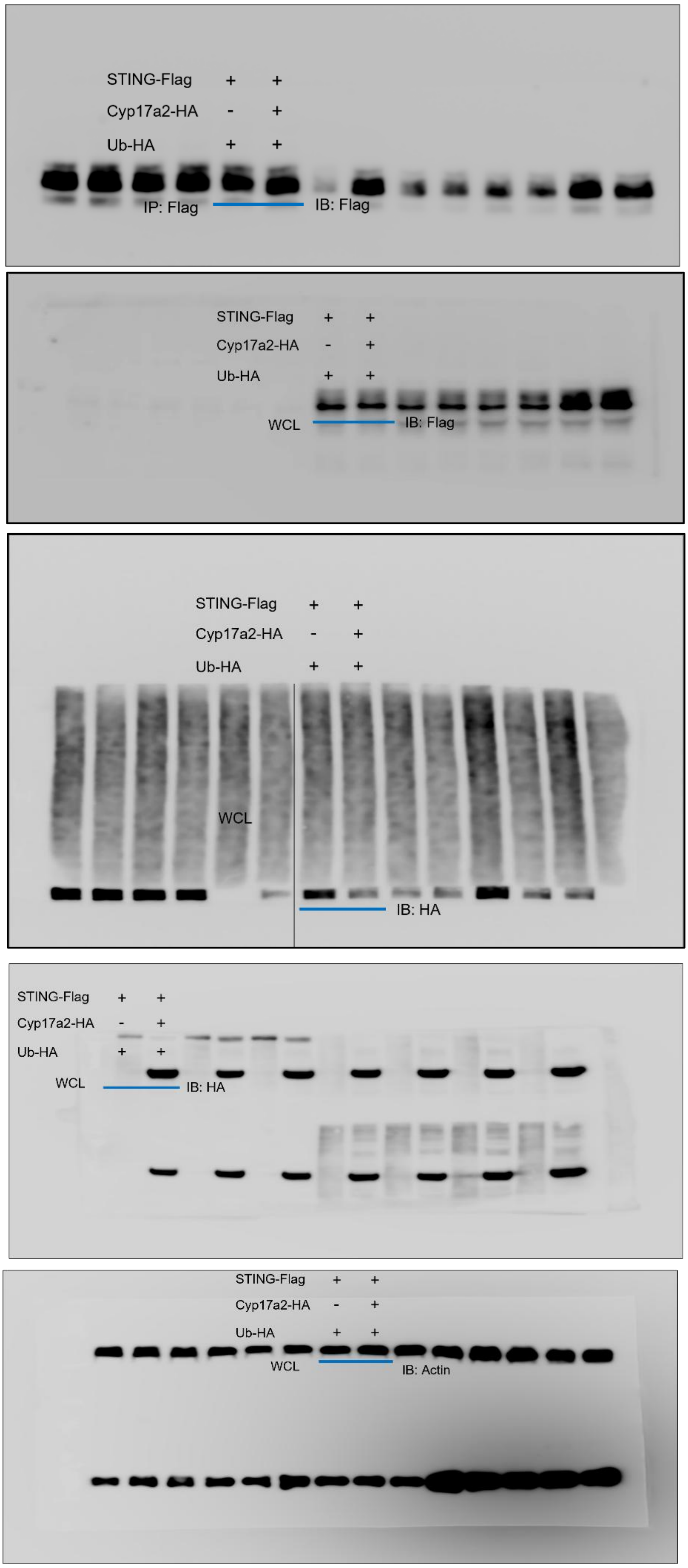

Figure 6, Source Data 1. Original membranes corresponding to Figure 6, panel B. Each

membrane is labelled with the relevant information. The blue lines indicate the corresponding bands.

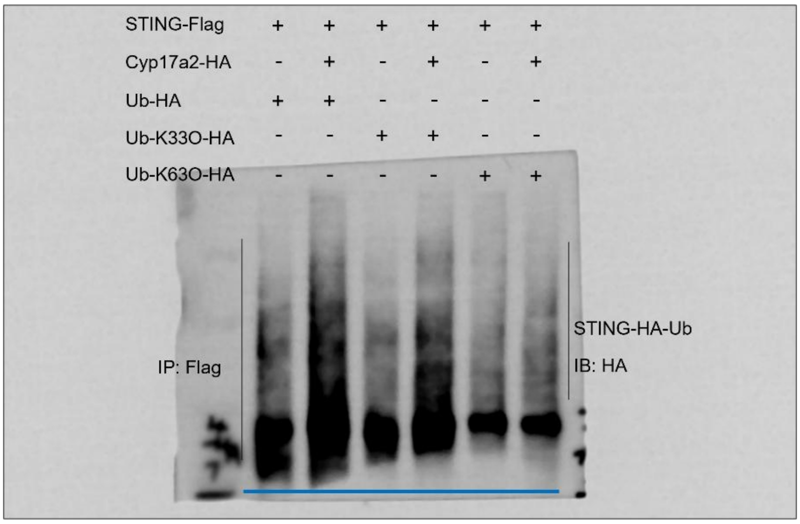

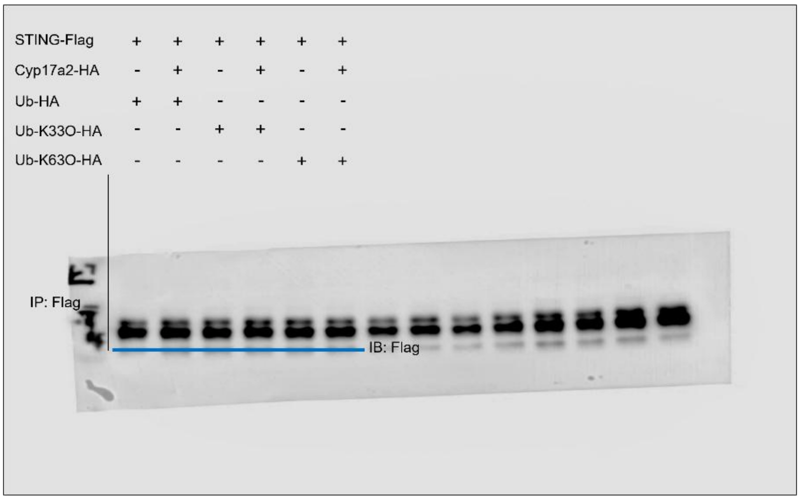

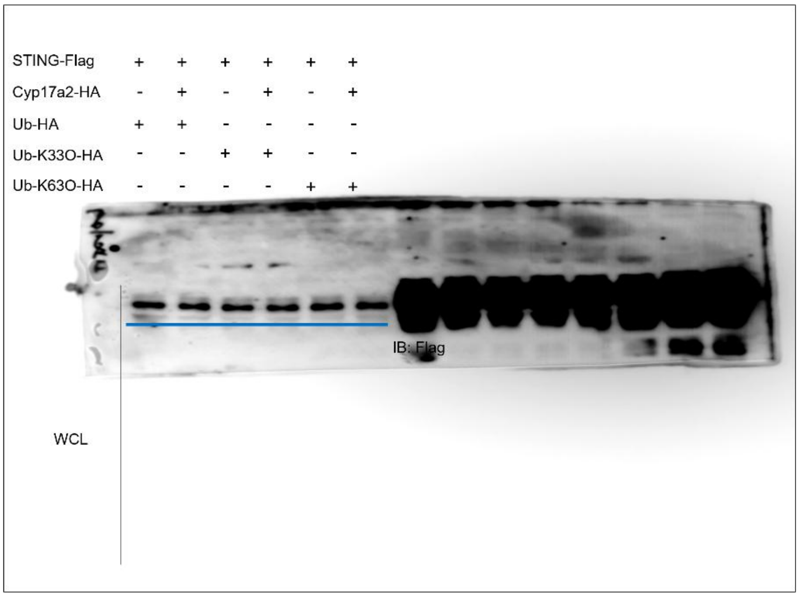

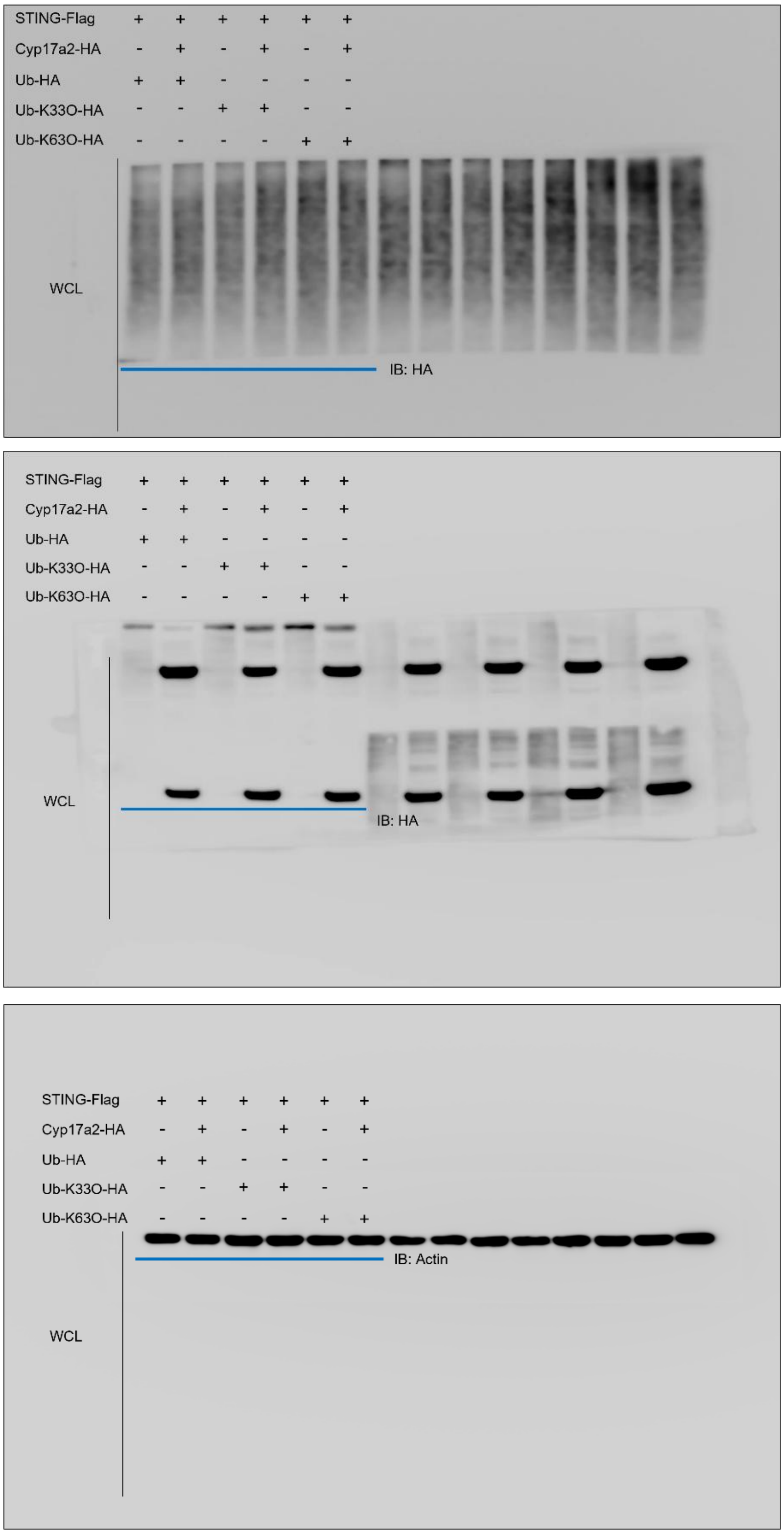

Figure 6, Source Data 1. Original membranes corresponding to Figure 6, panel C. Each membrane is labelled with the relevant information. The blue lines indicate the corresponding bands.

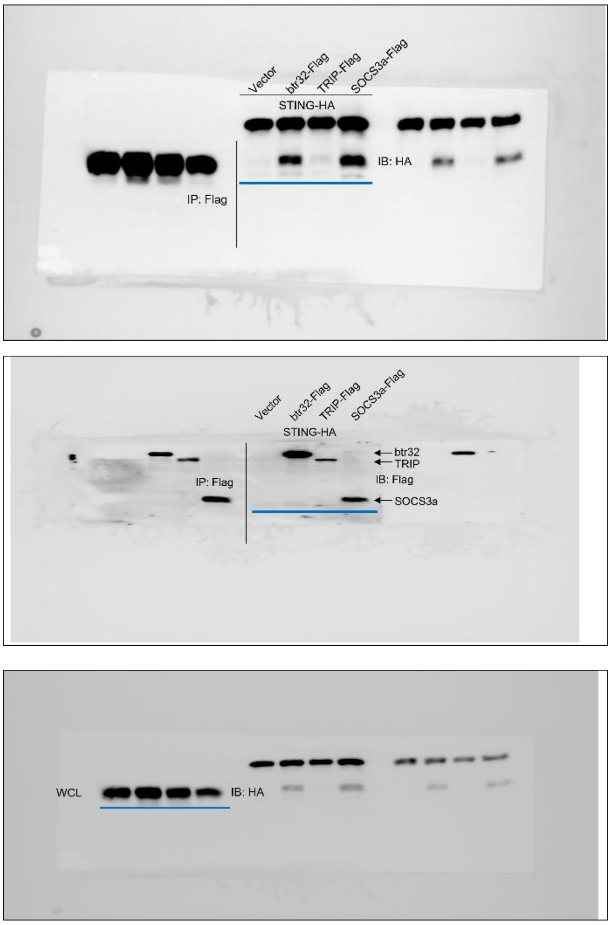

Figure 6, Source Data 1. Original membranes corresponding to Figure 6, panel D. Each membrane is labelled with the relevant information. The blue lines indicate the corresponding bands.

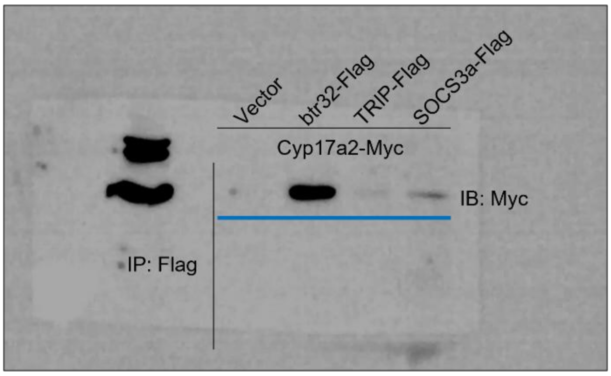

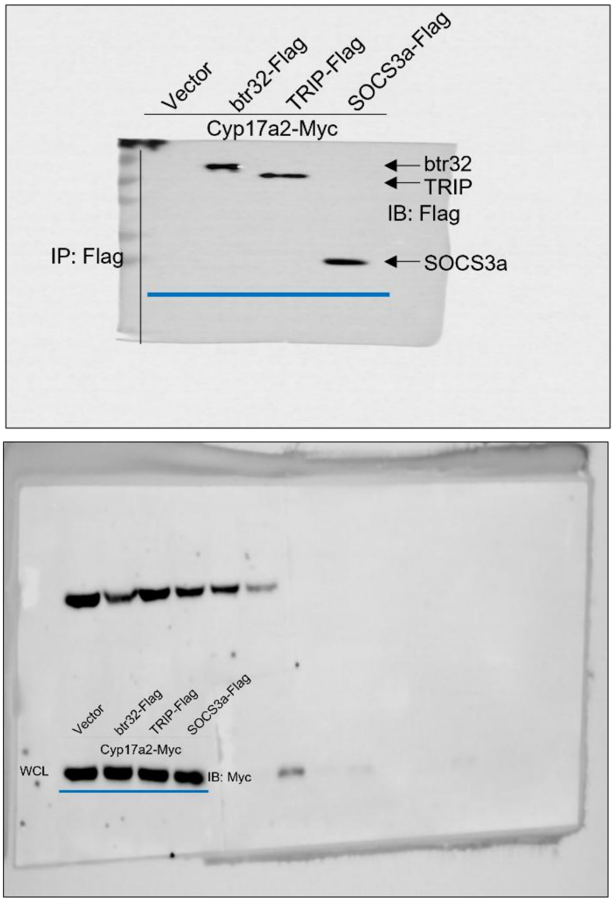

Figure 6, Source Data 1. Original membranes corresponding to Figure 6, panel E. Each membrane is labelled with the relevant information. The blue lines indicate the corresponding bands.

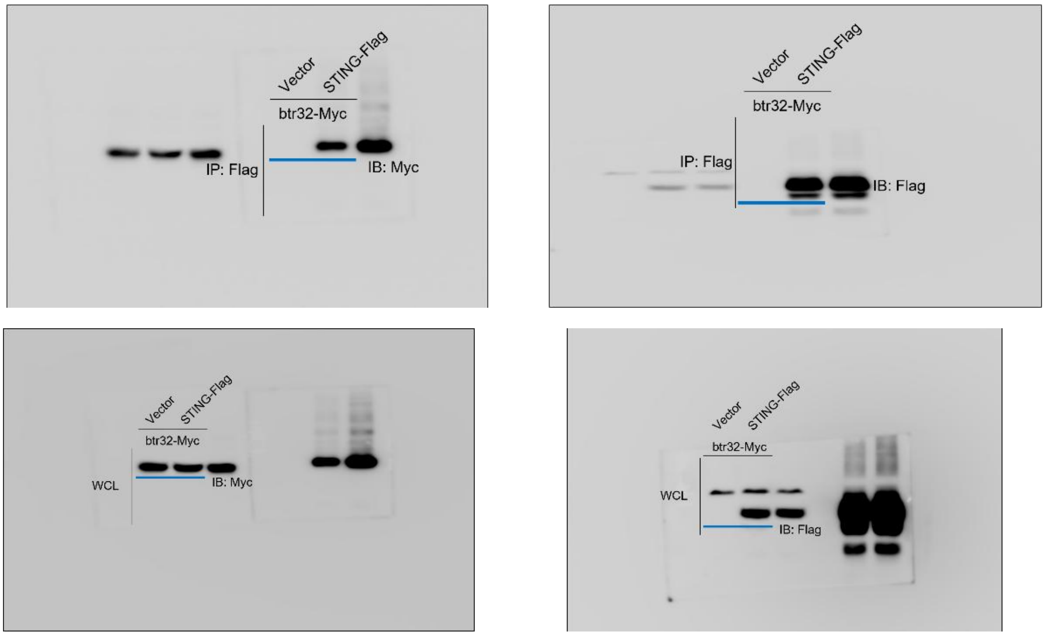

Figure 6, Source Data 1. Original membranes corresponding to Figure 6, panel F. Each membrane is labelled with the relevant information. The blue lines indicate the corresponding bands.

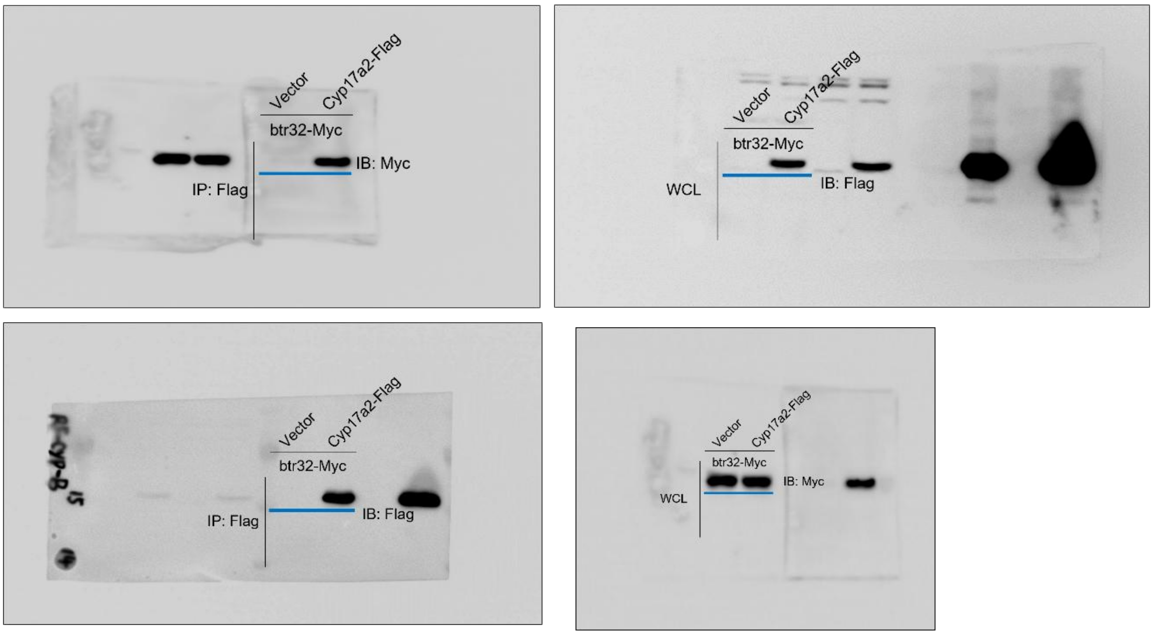

Figure 6, Source Data 1. Original membranes corresponding to Figure 6, panel G. Each membrane is labelled with the relevant information. The blue lines indicate the corresponding bands.

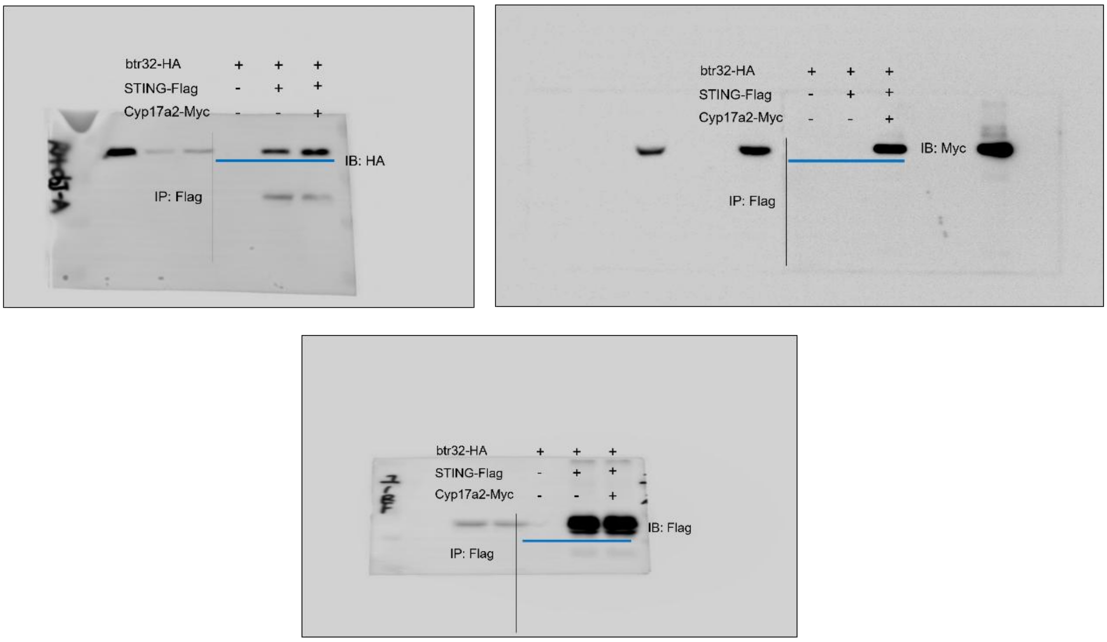

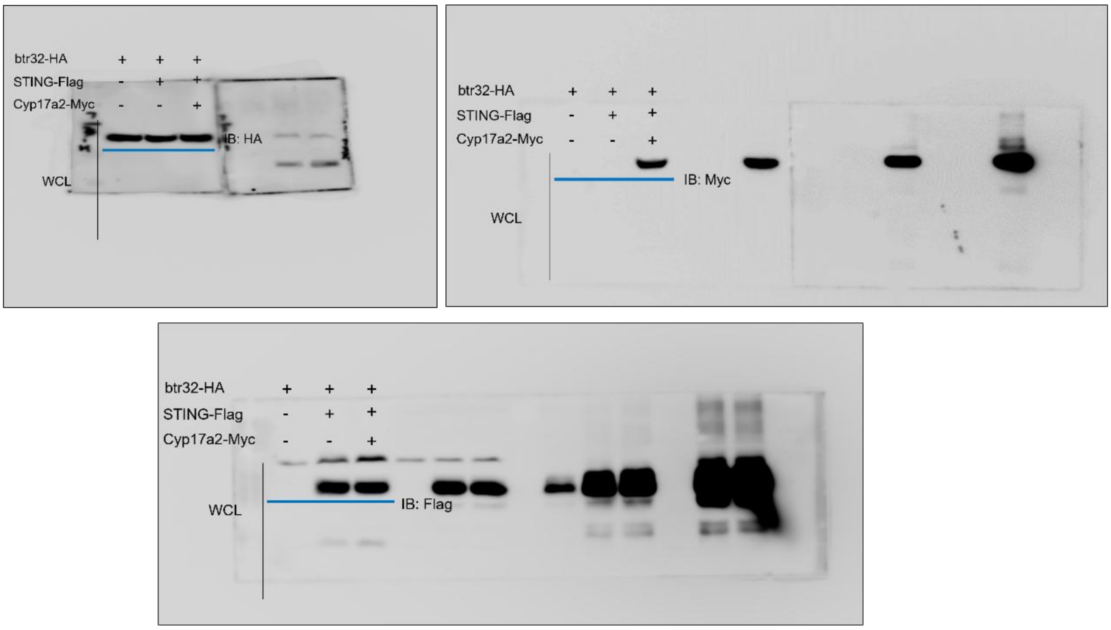

Figure 6, Source Data 1. Original membranes corresponding to Figure 6, panel K. Each membrane is labelled with the relevant information. The blue lines indicate the corresponding bands.

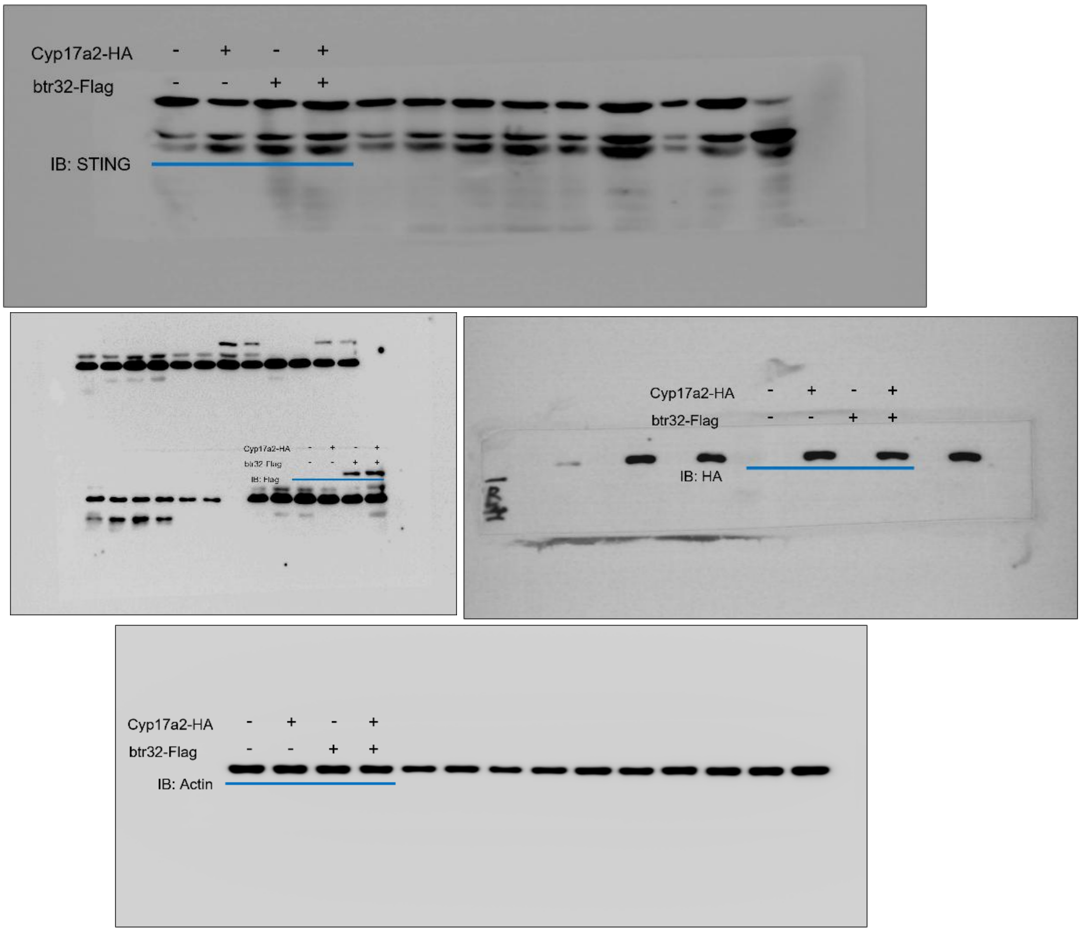

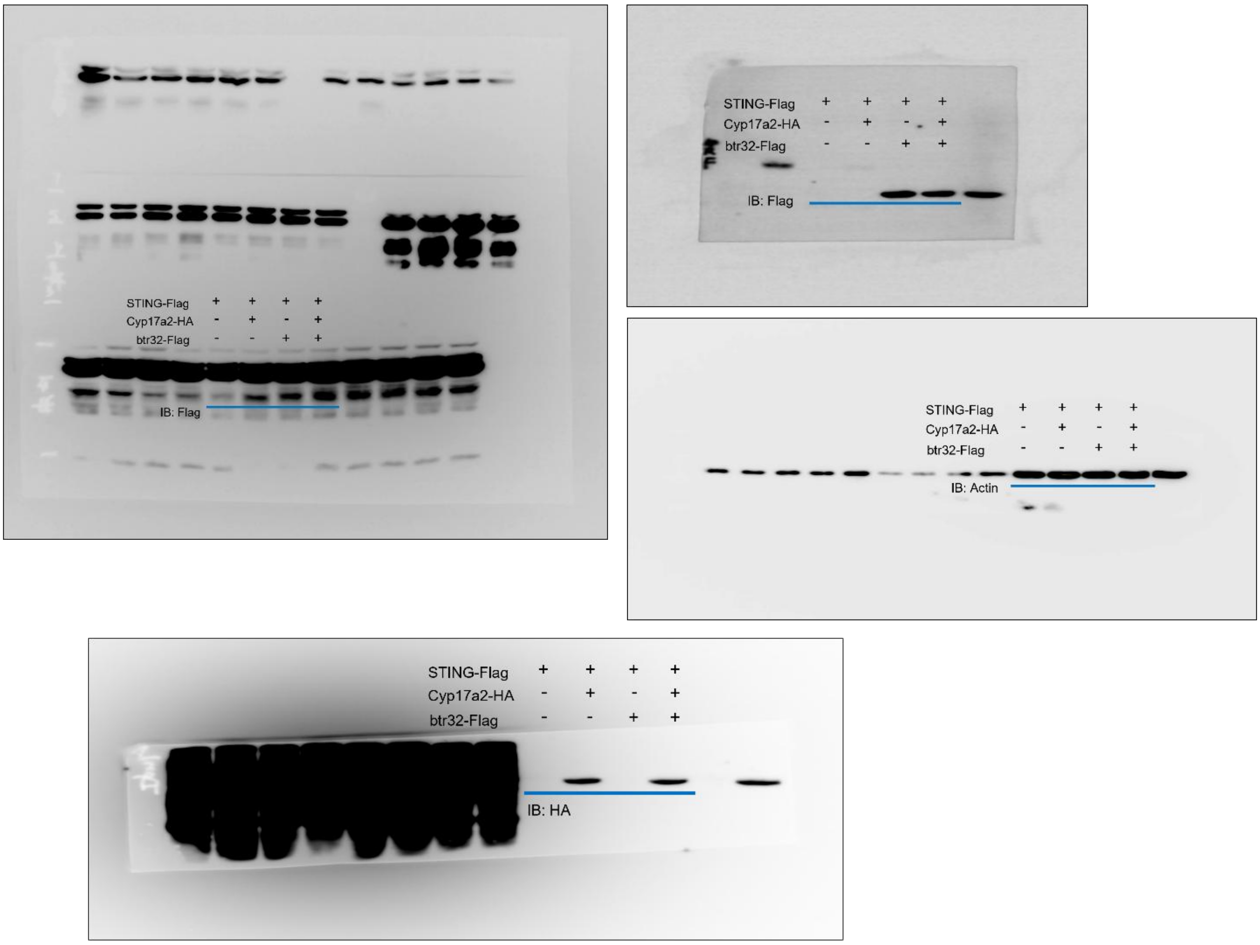

Figure 6, Source Data 1. Original membranes corresponding to Figure 6, panel N. Each membrane is labelled with the relevant information. The blue lines indicate the corresponding bands.
