# Supplementary figures and images for: Male-biased Cyp17a2 orchestrates antiviral sexual dimorphism in fish via STING stabilization and viral protein degradation (part 2 of 5)
Source: eLife. 2026 Feb 18;14:RP108048. doi: 10.7554/eLife.108048 (PMC12916102; doi:10.7554/eLife.108048)

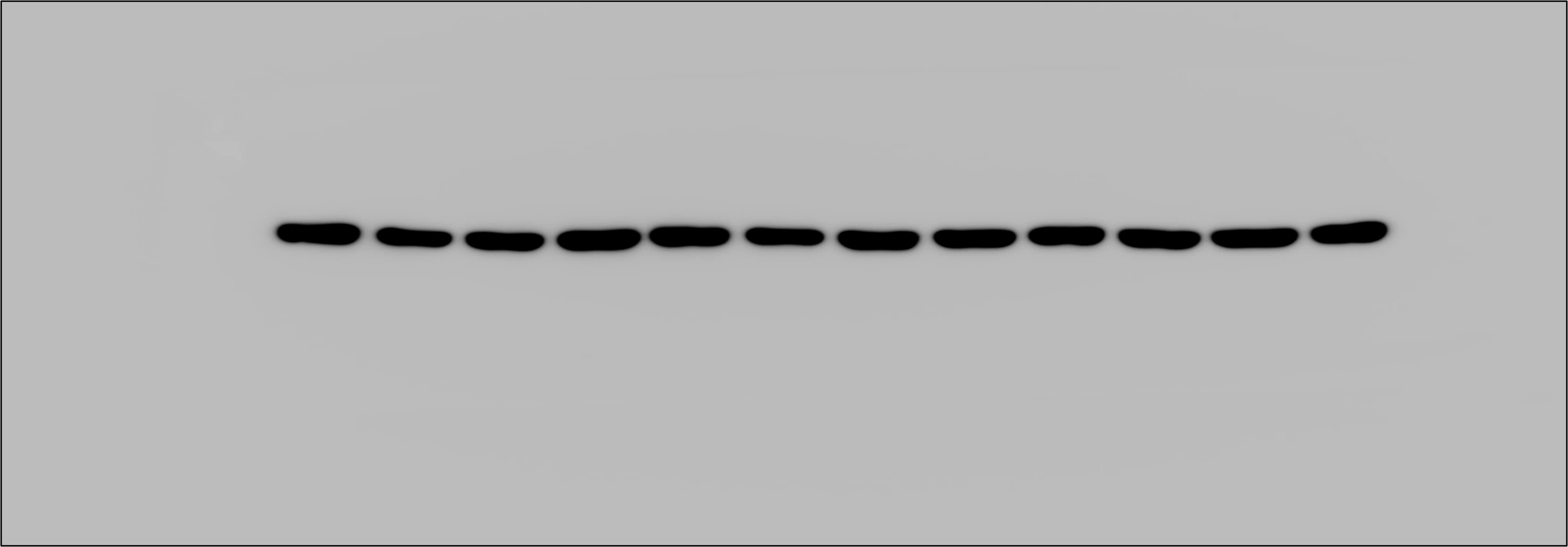

Supplement: Figure 6—source data 2. [file elife-108048-fig6-data2.zip › Figure 6/Figure 6 A-Actin.tif]

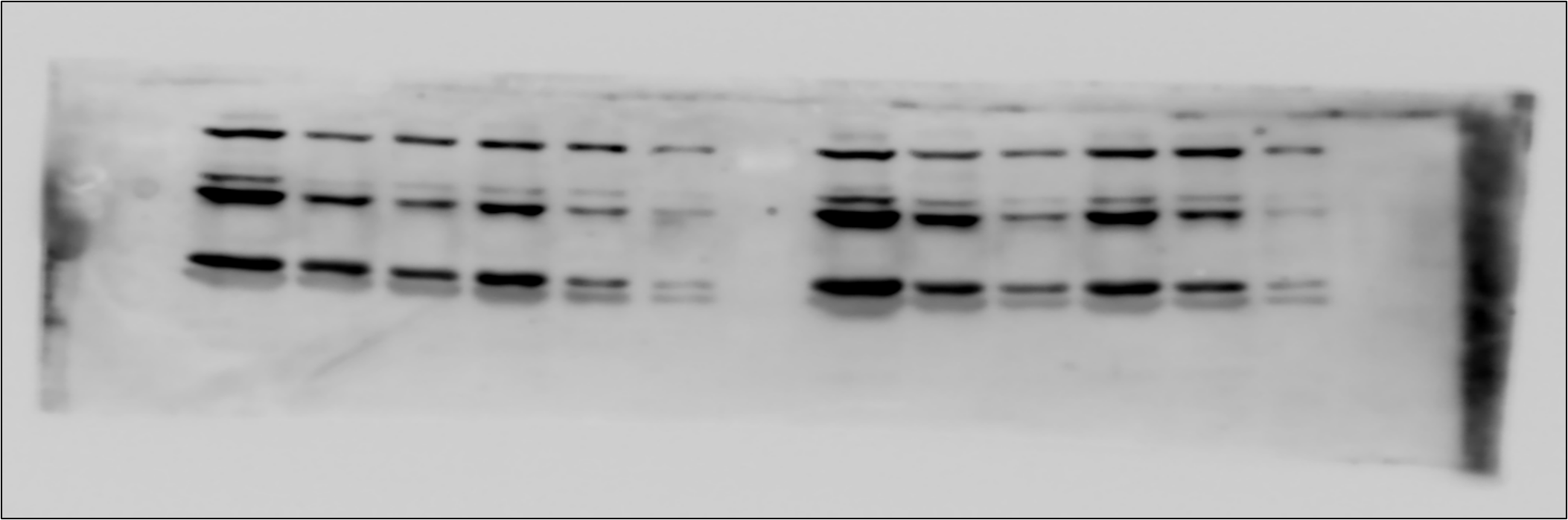

Supplement: Figure 6—source data 2. [file elife-108048-fig6-data2.zip › Figure 6/Figure 6 A-cyp17a2-2.tif]

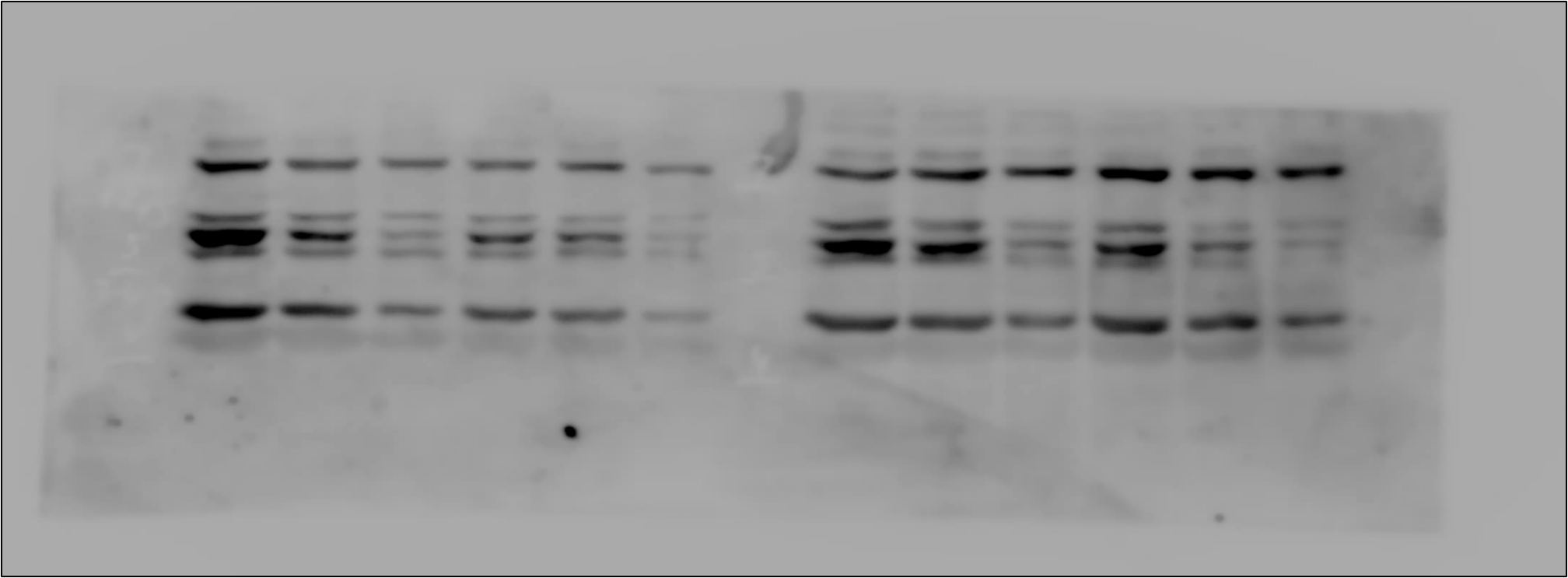

Supplement: Figure 6—source data 2. [file elife-108048-fig6-data2.zip › Figure 6/Figure 6 A-cyp17a2-3.tif]

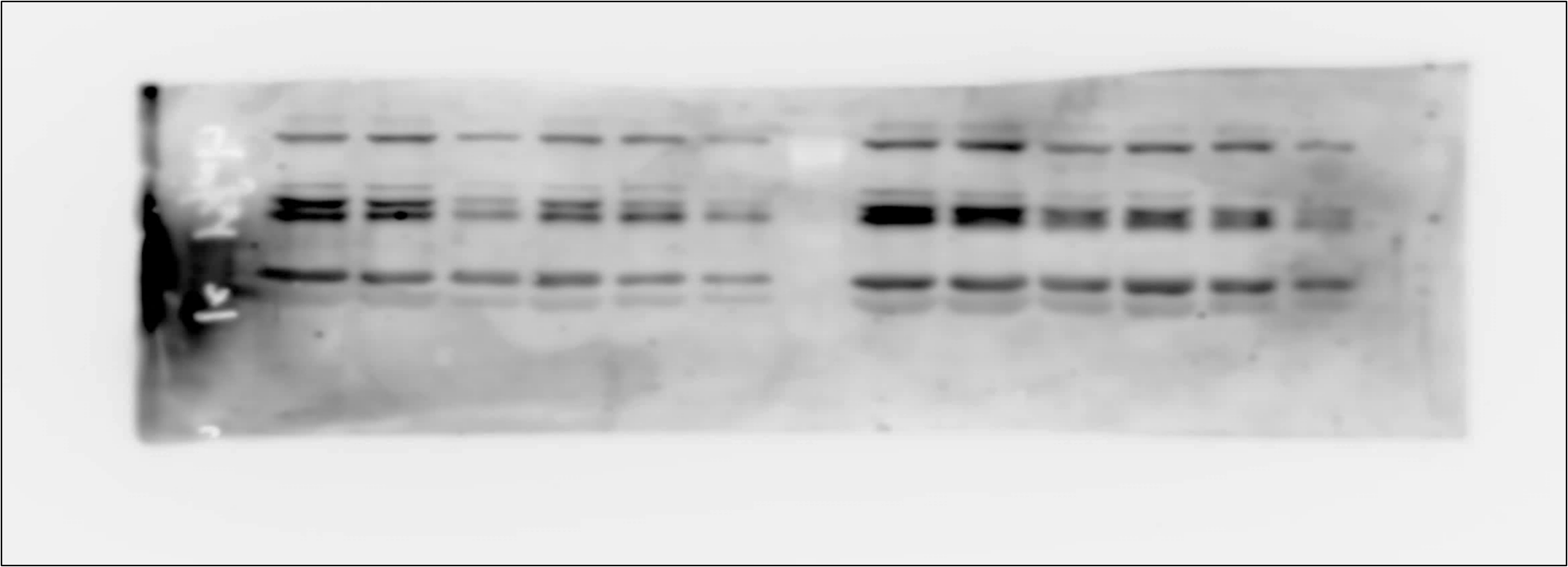

Supplement: Figure 6—source data 2. [file elife-108048-fig6-data2.zip › Figure 6/Figure 6 A-cyp17a2.tif]

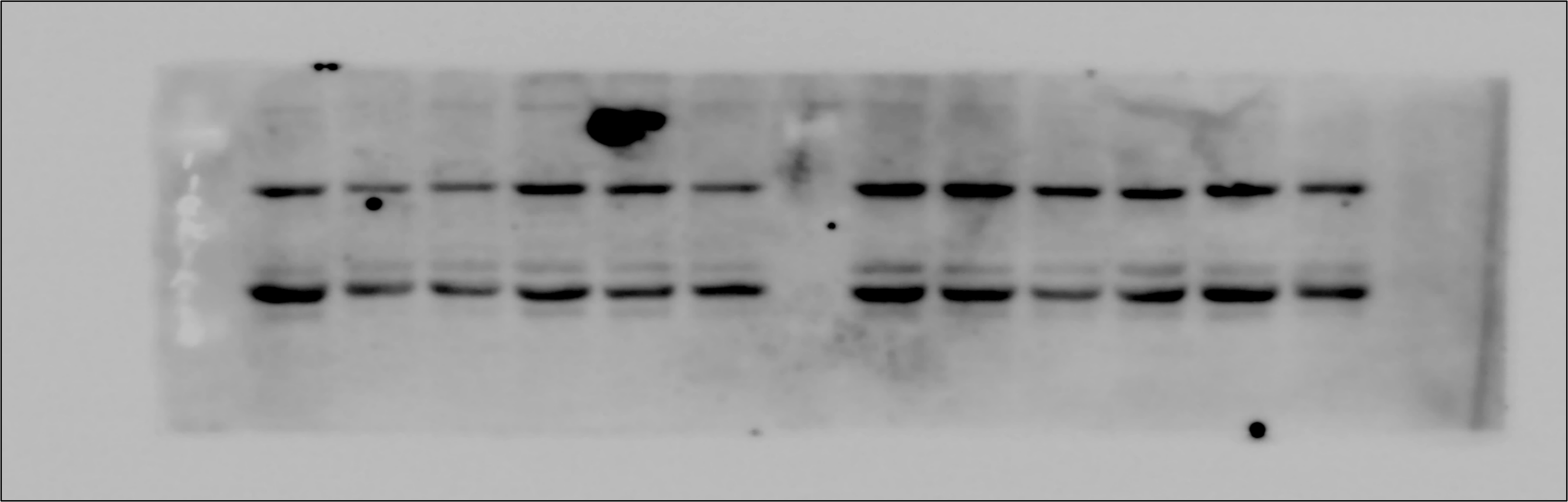

Supplement: Figure 6—source data 2. [file elife-108048-fig6-data2.zip › Figure 6/Figure 6 A-STING-2.tif]

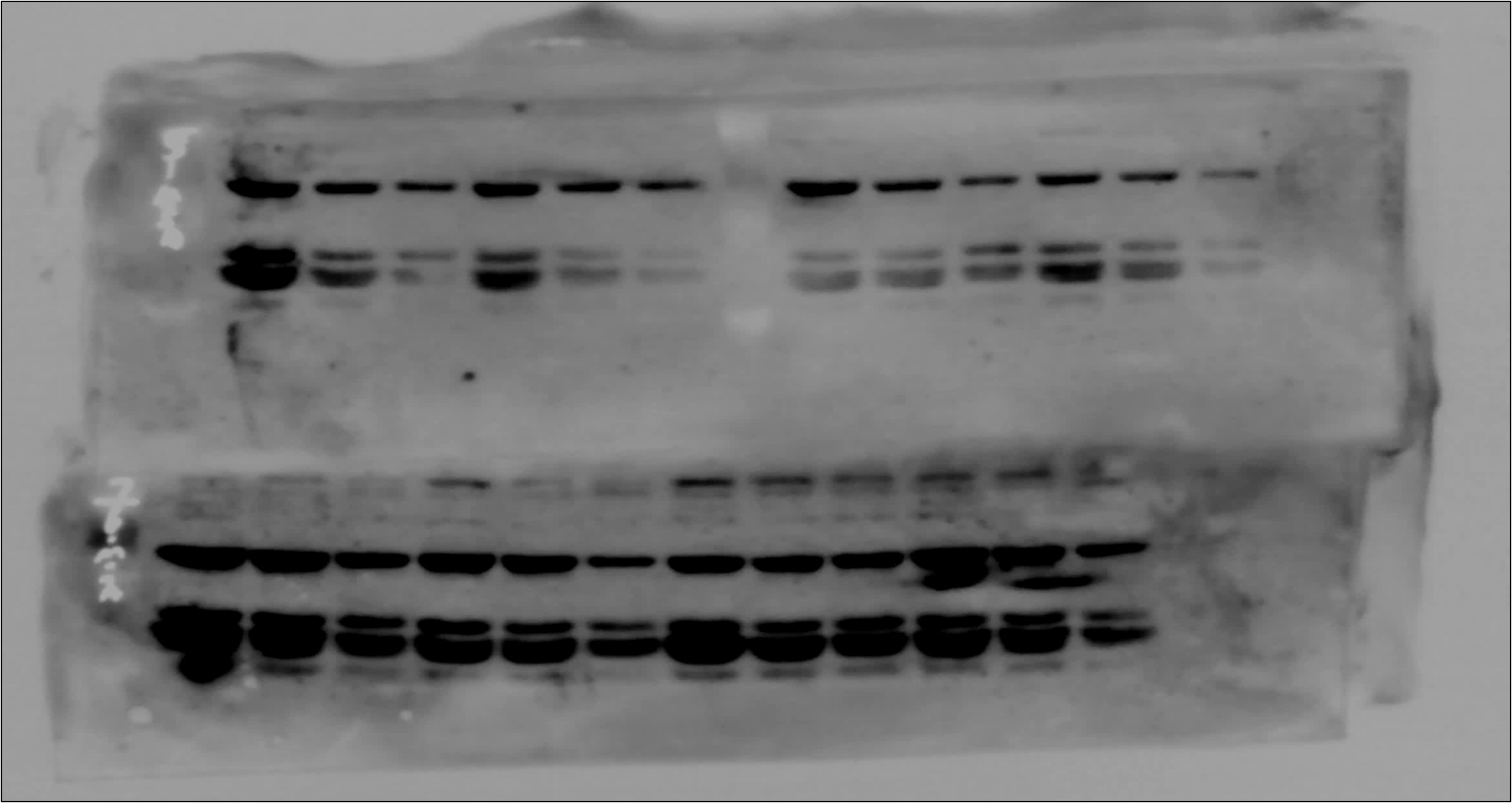

Supplement: Figure 6—source data 2. [file elife-108048-fig6-data2.zip › Figure 6/Figure 6 A-STING-3.tif]

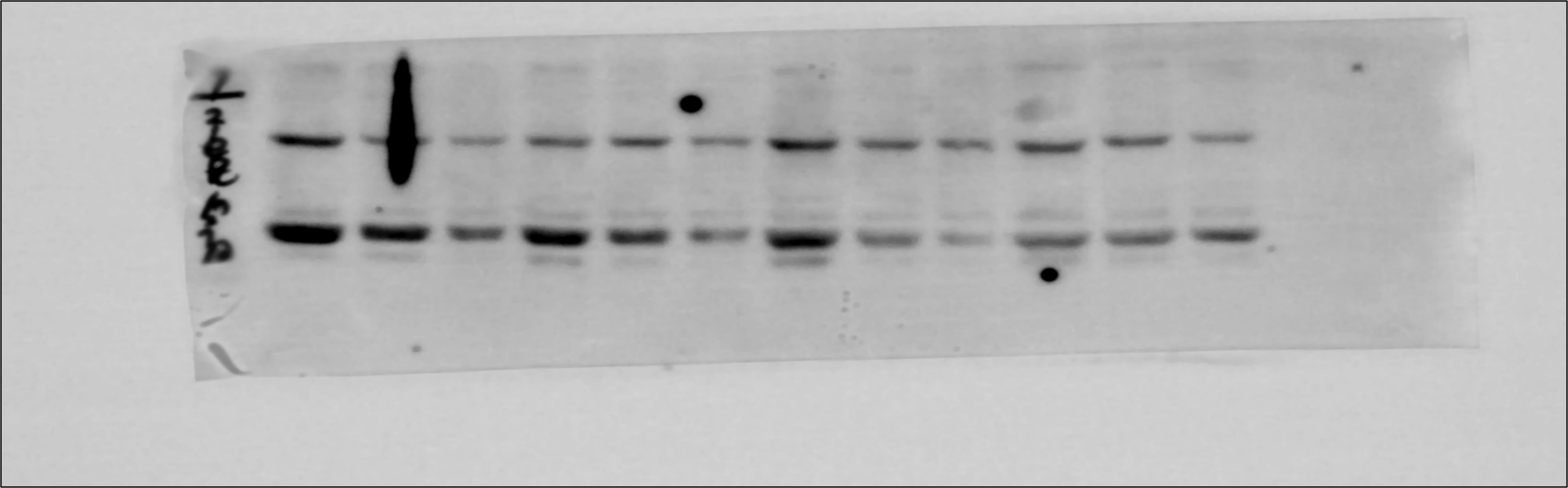

Supplement: Figure 6—source data 2. [file elife-108048-fig6-data2.zip › Figure 6/Figure 6 A-STING.tif]

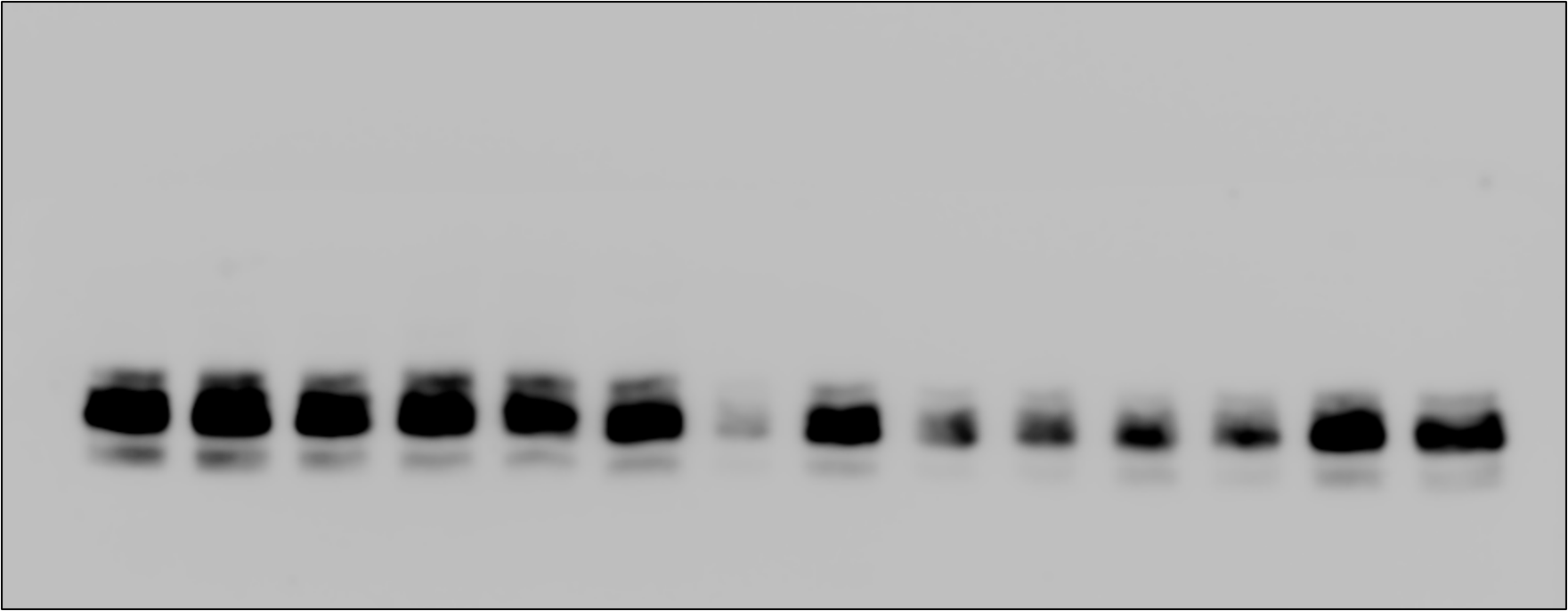

Supplement: Figure 6—source data 2. [file elife-108048-fig6-data2.zip › Figure 6/Figure 6 B-IP-Flag.tif]

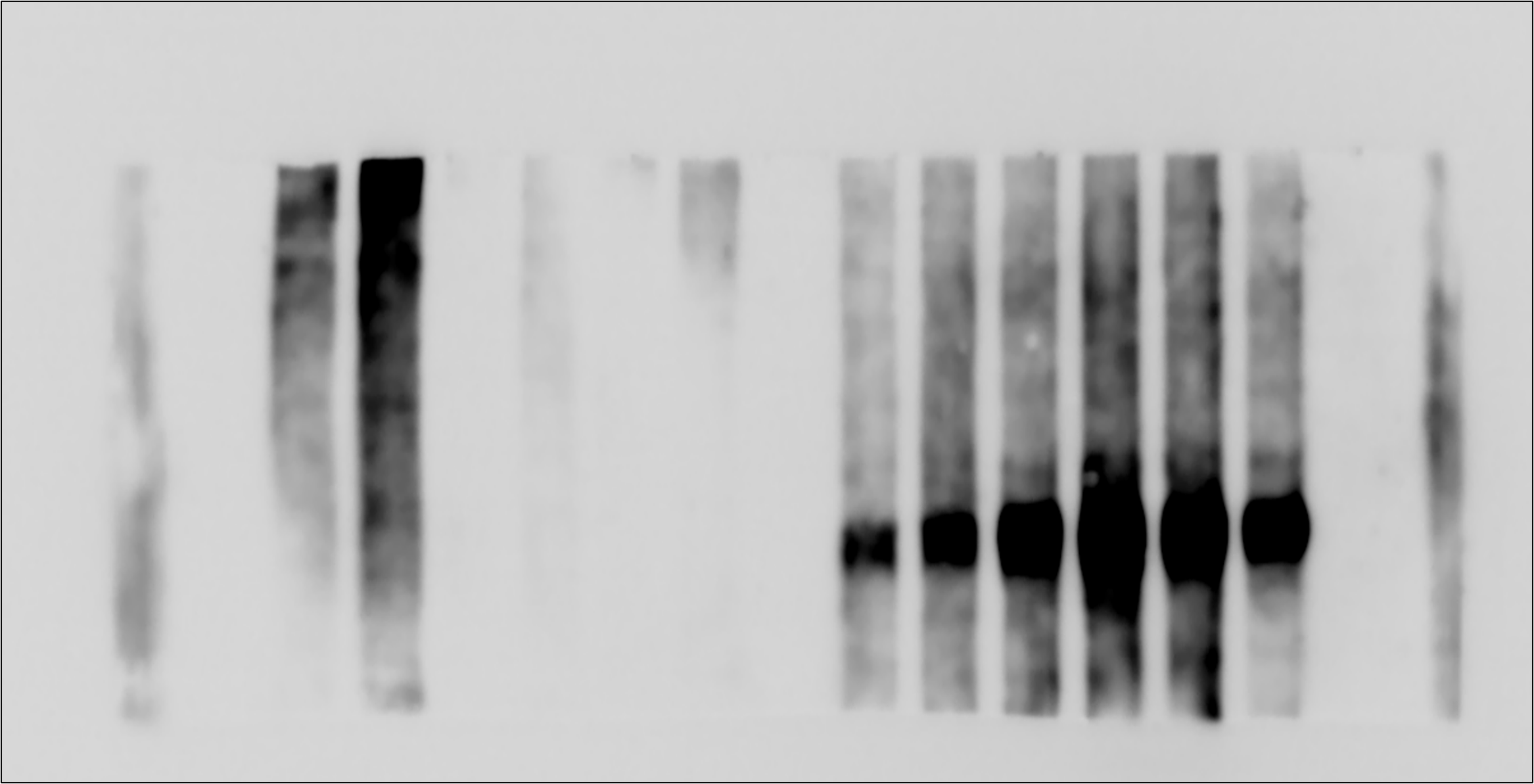

Supplement: Figure 6—source data 2. [file elife-108048-fig6-data2.zip › Figure 6/Figure 6 B-IP-HA.tif]

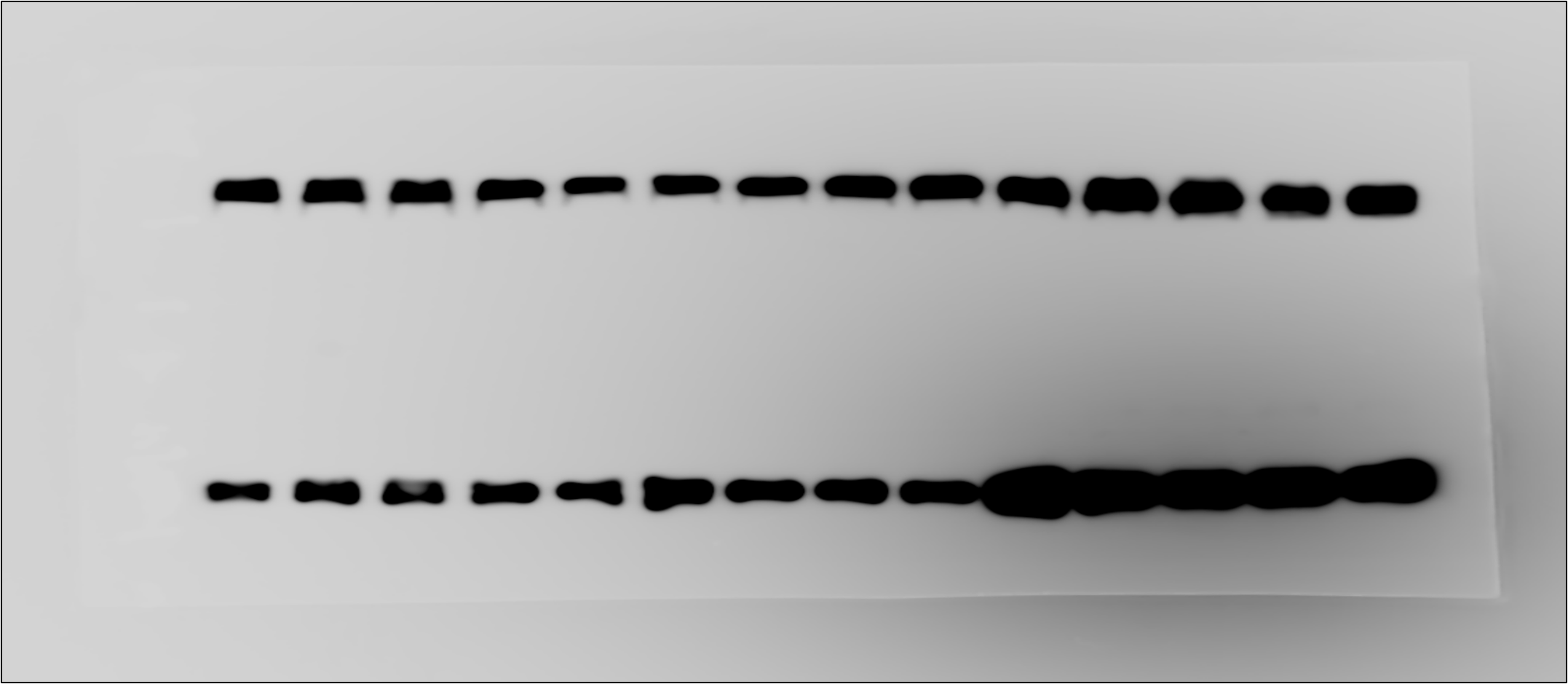

Supplement: Figure 6—source data 2. [file elife-108048-fig6-data2.zip › Figure 6/Figure 6 B-WCL-Actin.tif]

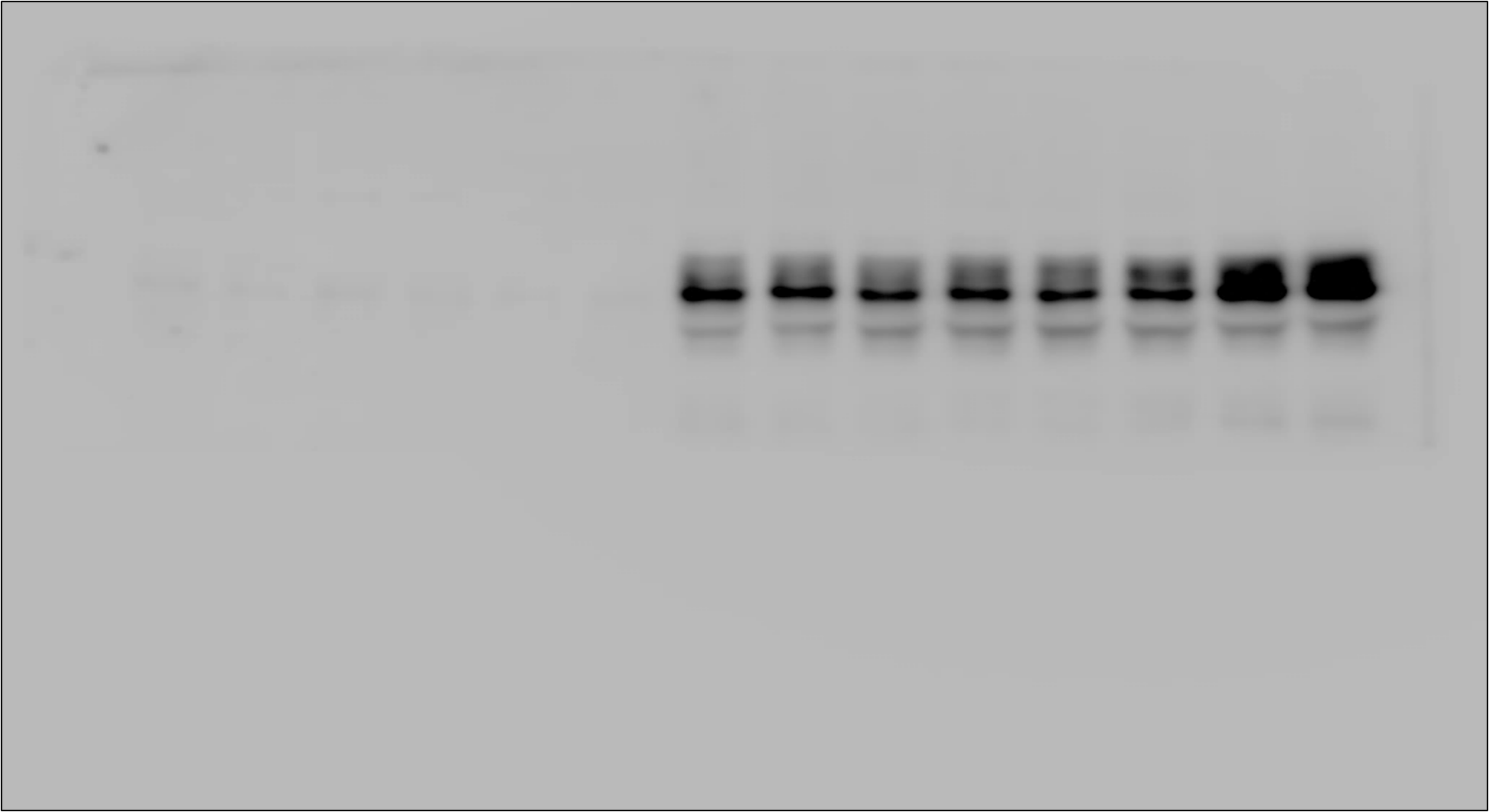

Supplement: Figure 6—source data 2. [file elife-108048-fig6-data2.zip › Figure 6/Figure 6 B-WCL-Flag.tif]

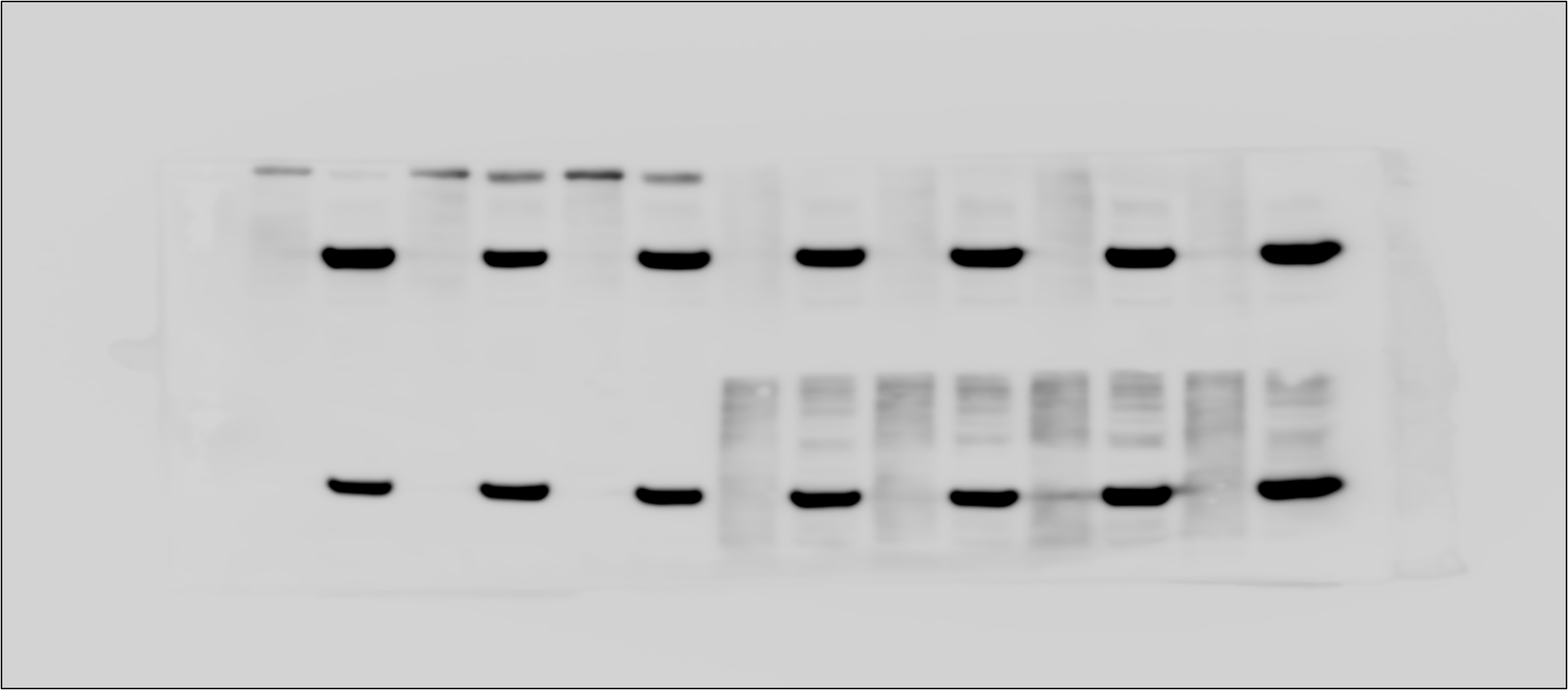

Supplement: Figure 6—source data 2. [file elife-108048-fig6-data2.zip › Figure 6/Figure 6 B-WCL-HA-cyp17a2.tif]

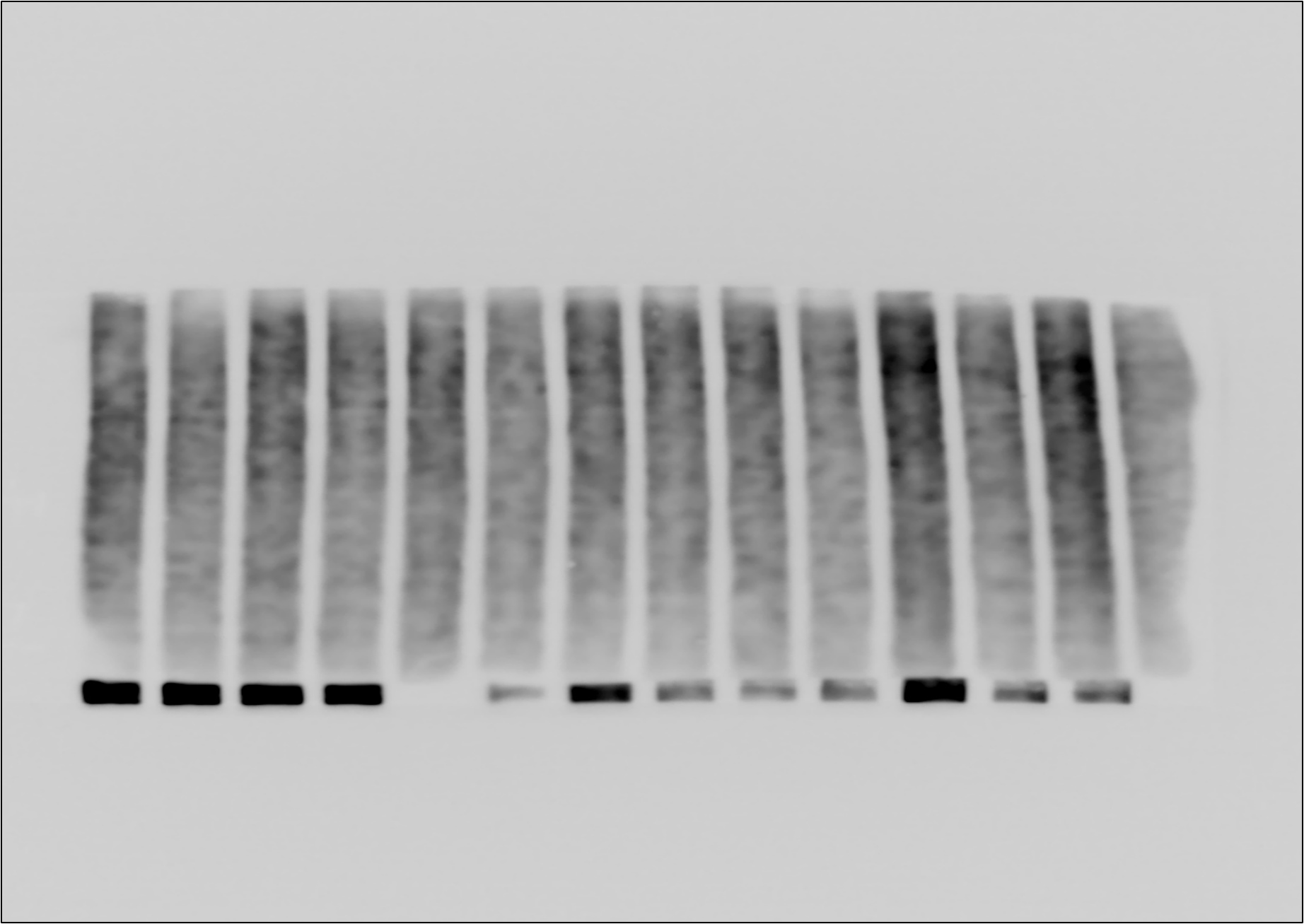

Supplement: Figure 6—source data 2. [file elife-108048-fig6-data2.zip › Figure 6/Figure 6 B-WCL-HA.tif]

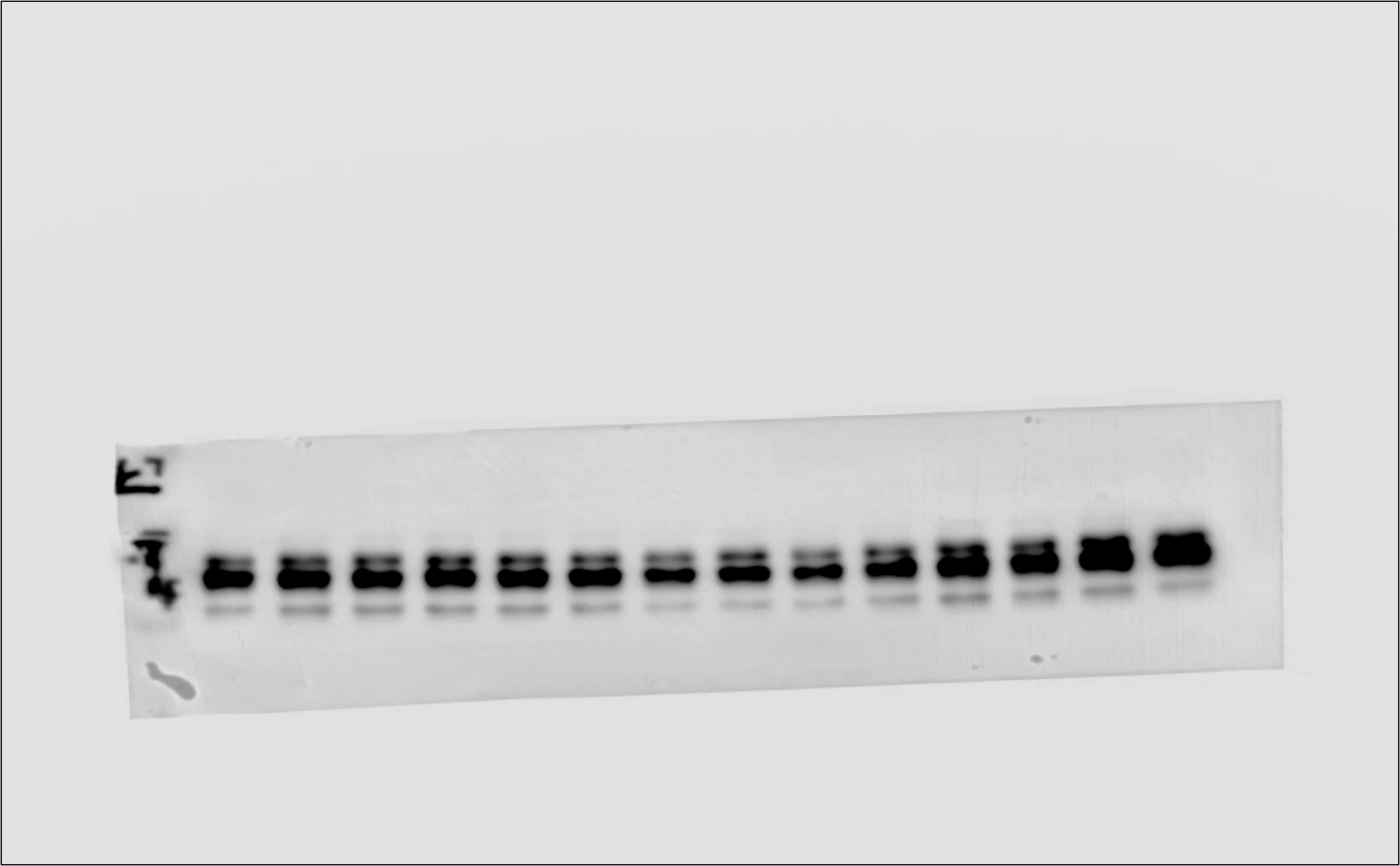

Supplement: Figure 6—source data 2. [file elife-108048-fig6-data2.zip › Figure 6/Figure 6 C-IP-Flag.tif]

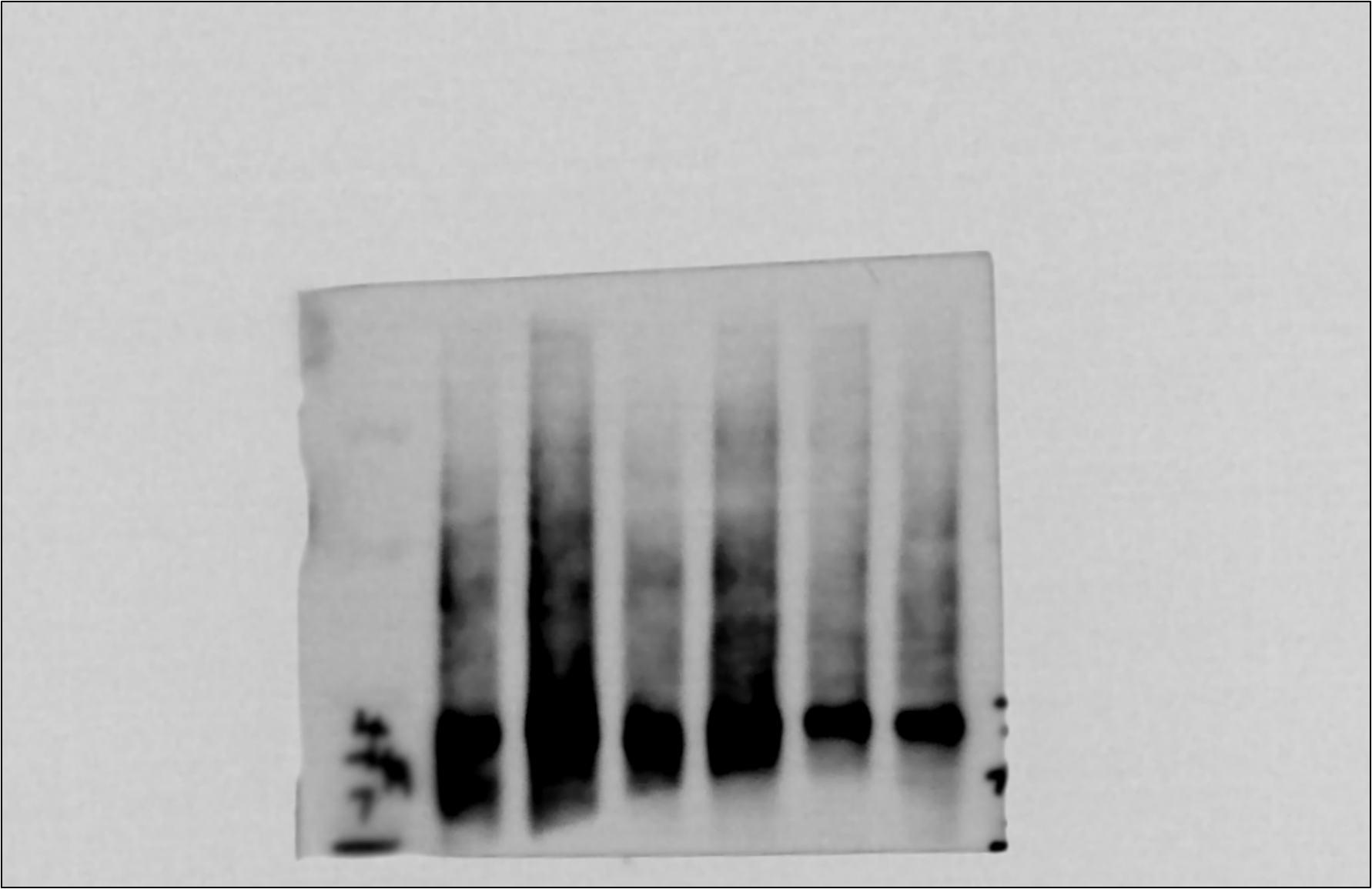

Supplement: Figure 6—source data 2. [file elife-108048-fig6-data2.zip › Figure 6/Figure 6 C-IP-HA.tif]

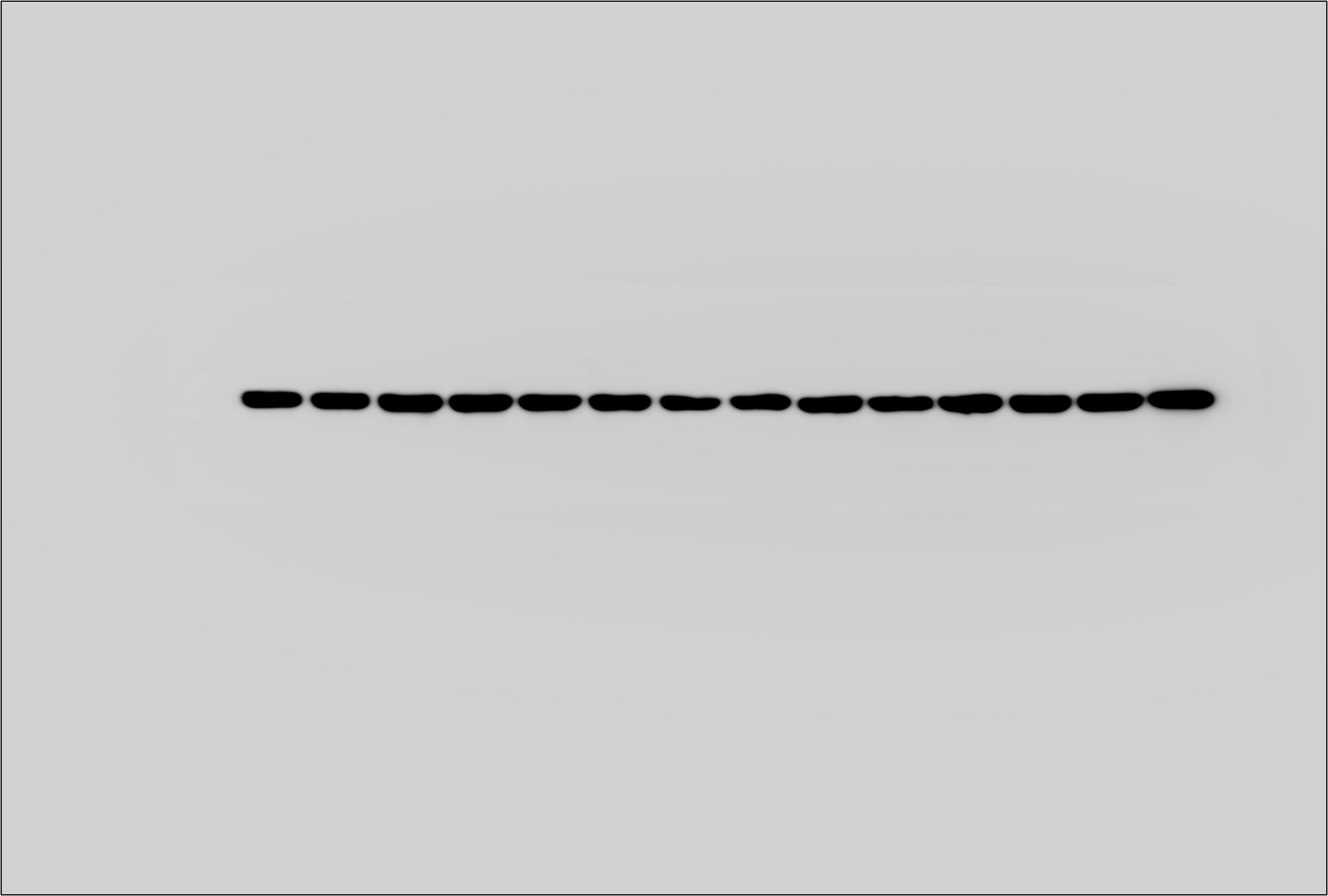

Supplement: Figure 6—source data 2. [file elife-108048-fig6-data2.zip › Figure 6/Figure 6 C-WCL-Actin.tif]

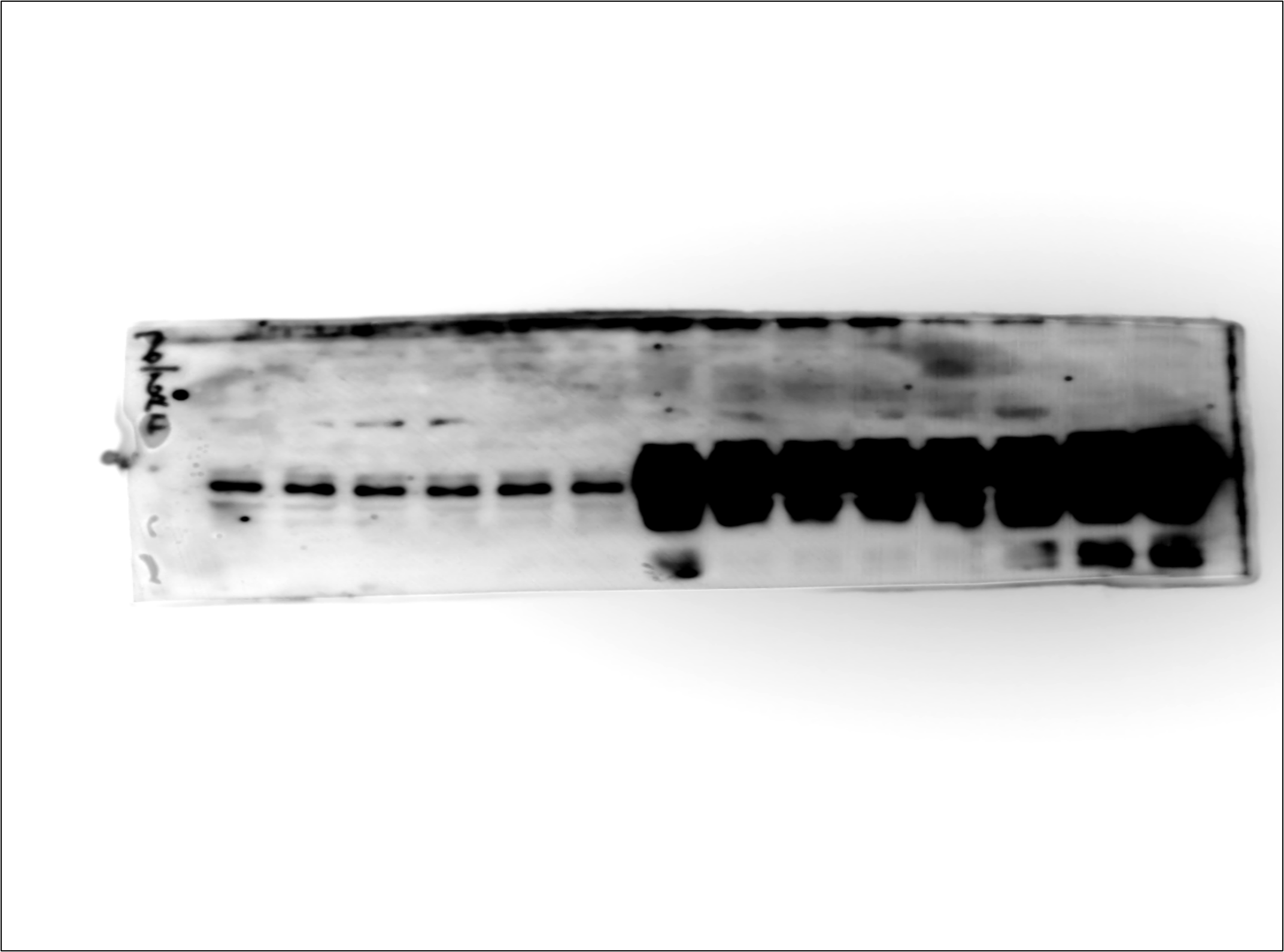

Supplement: Figure 6—source data 2. [file elife-108048-fig6-data2.zip › Figure 6/Figure 6 C-WCL-Flag.tif]

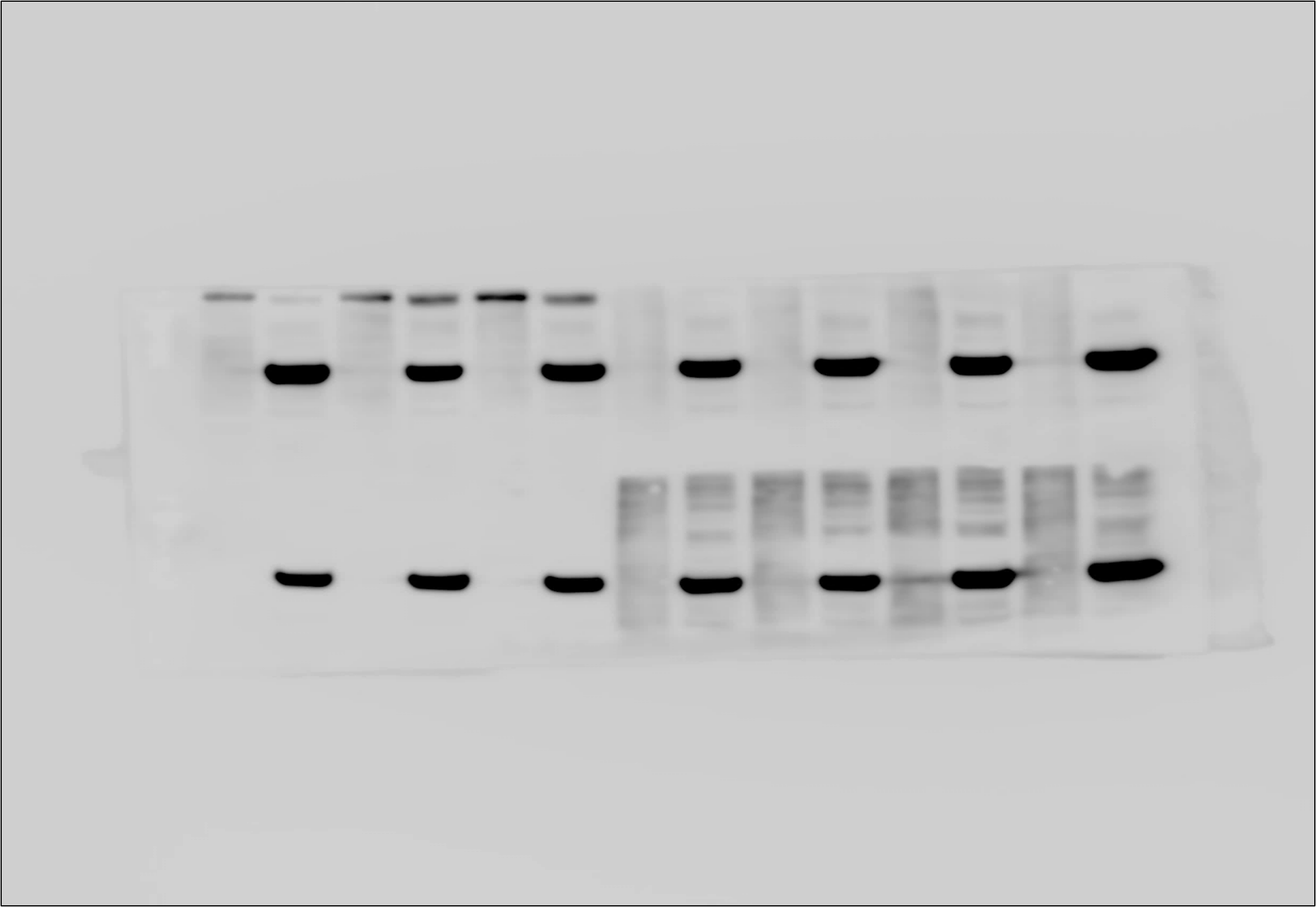

Supplement: Figure 6—source data 2. [file elife-108048-fig6-data2.zip › Figure 6/Figure 6 C-WCL-HA-cyp17a2.tif]

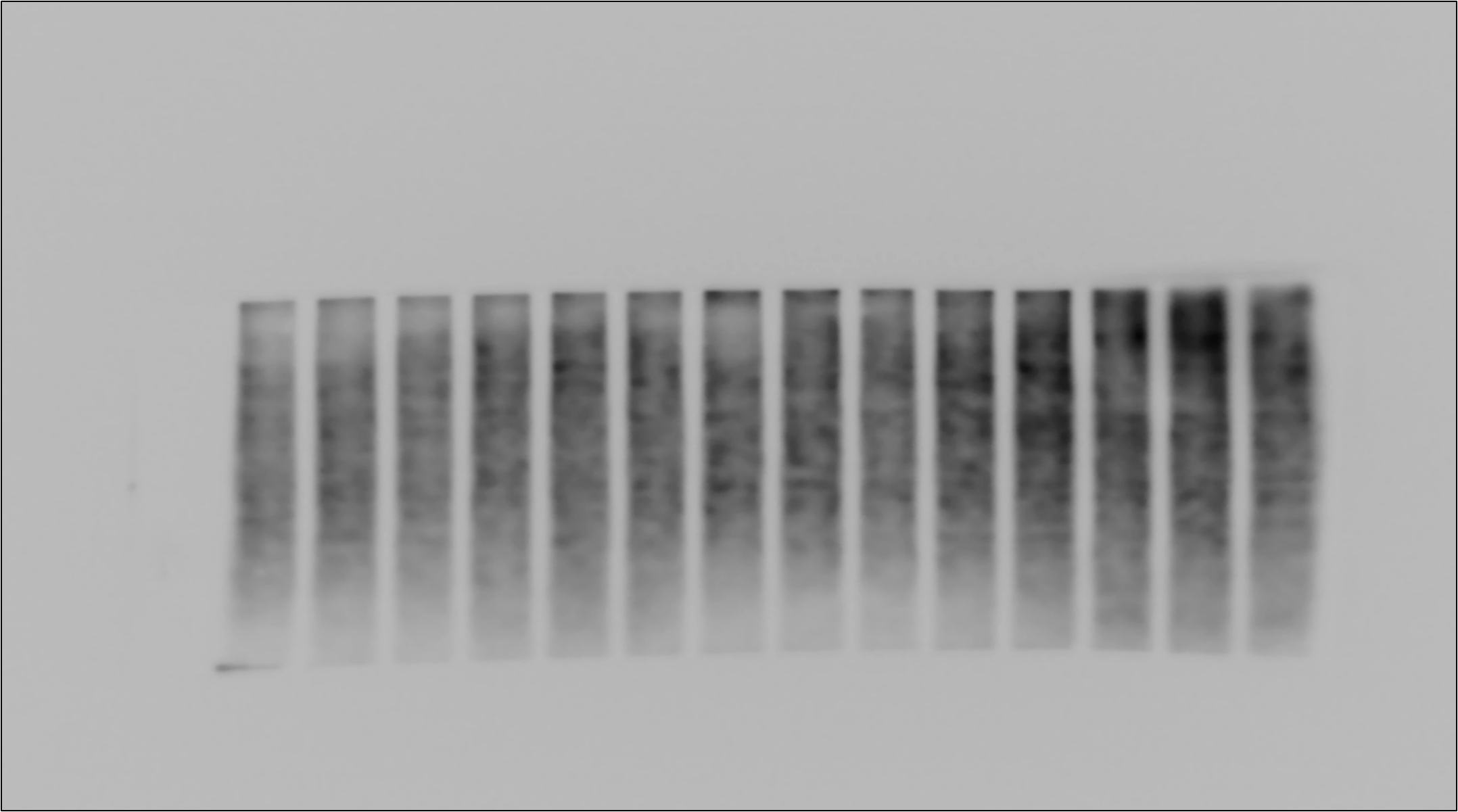

Supplement: Figure 6—source data 2. [file elife-108048-fig6-data2.zip › Figure 6/Figure 6 C-WCL-HA.tif]

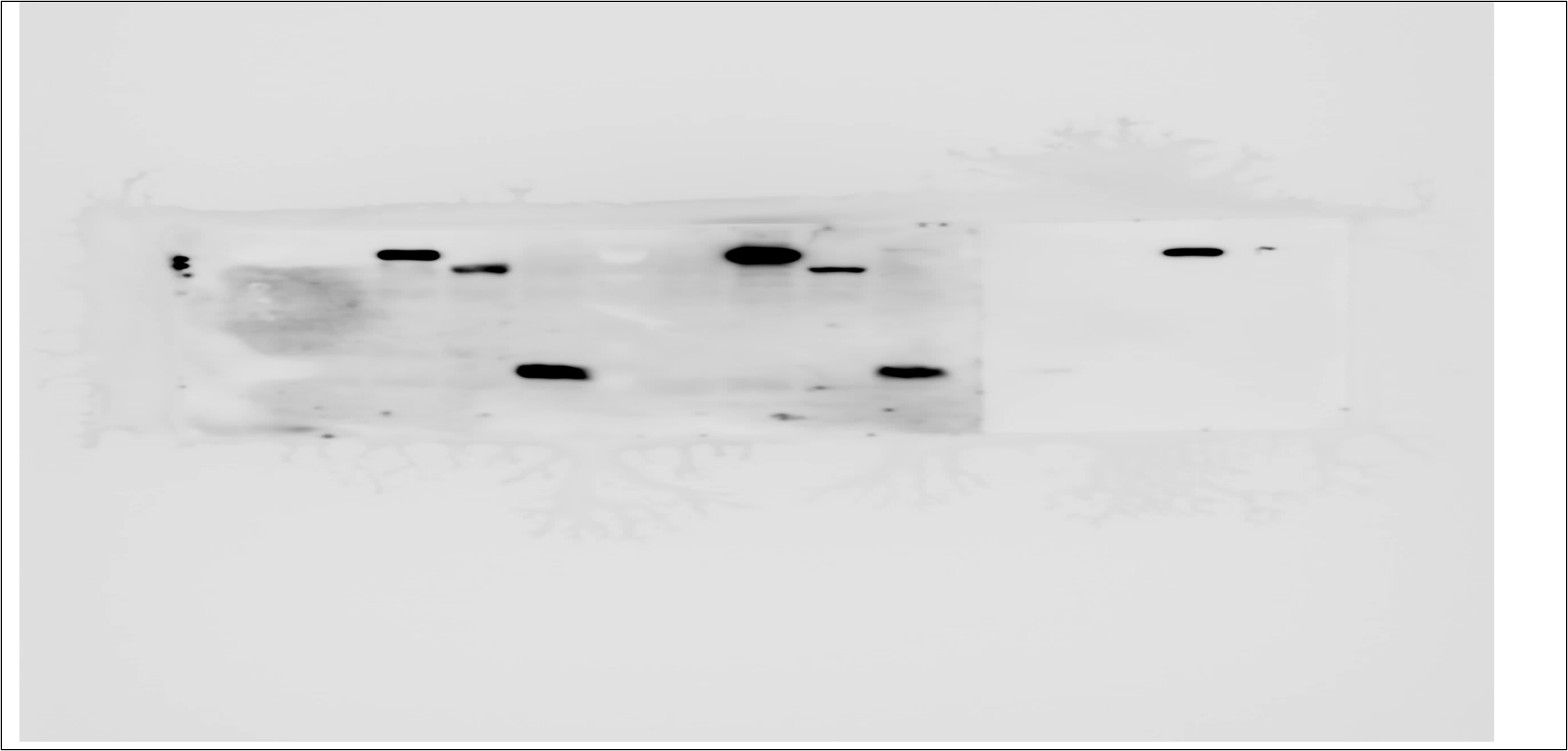

Supplement: Figure 6—source data 2. [file elife-108048-fig6-data2.zip › Figure 6/Figure 6 D-IP-Flag.tif]

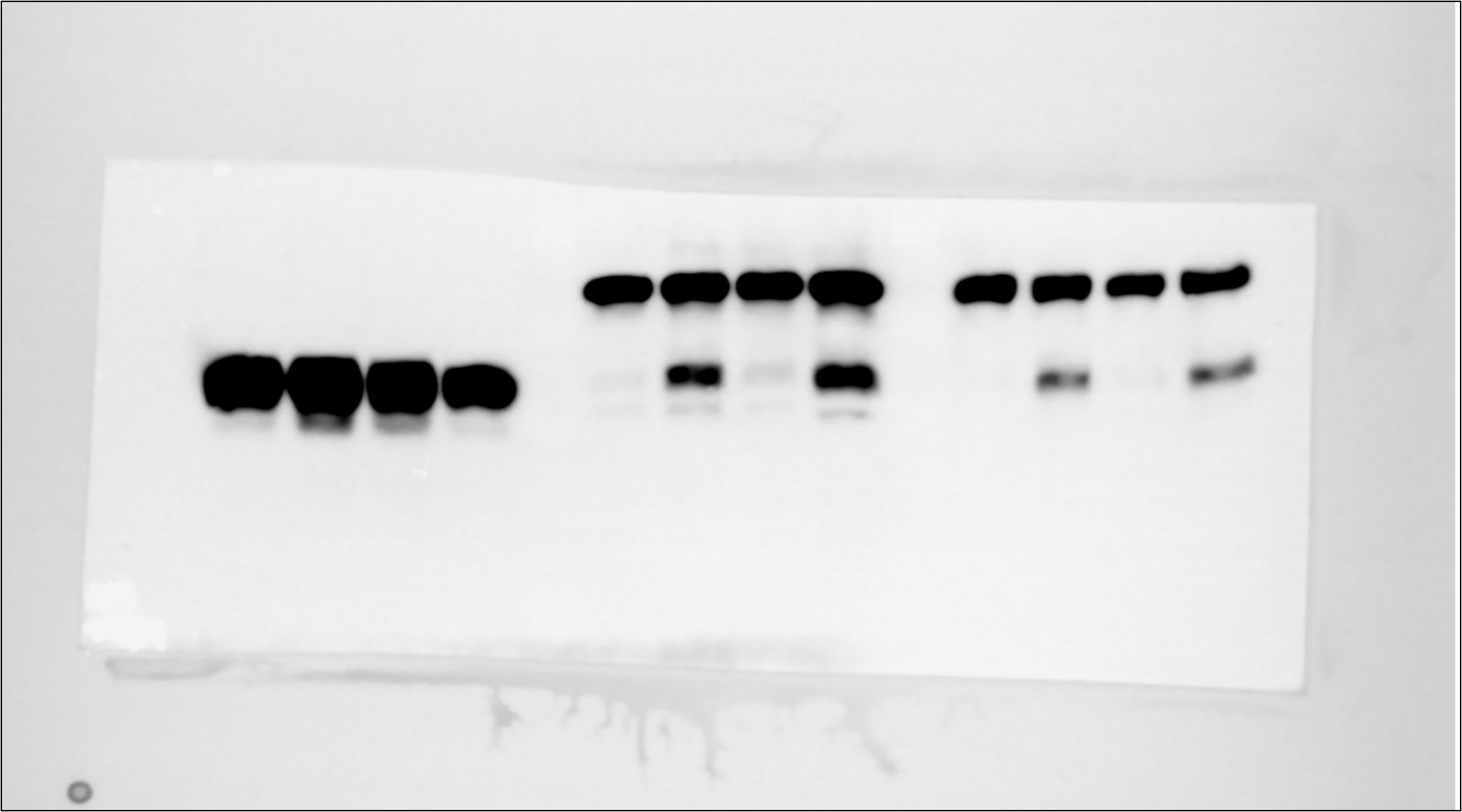

Supplement: Figure 6—source data 2. [file elife-108048-fig6-data2.zip › Figure 6/Figure 6 D-IP-HA.tif]

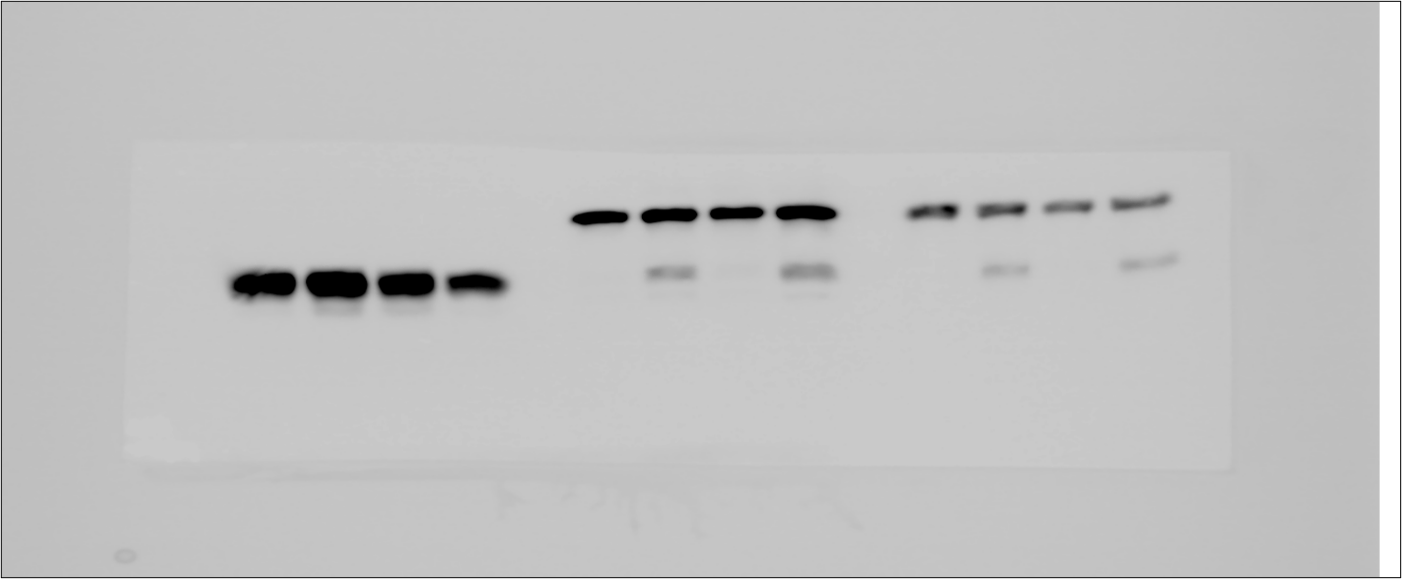

Supplement: Figure 6—source data 2. [file elife-108048-fig6-data2.zip › Figure 6/Figure 6 D-WCL-HA.tif]

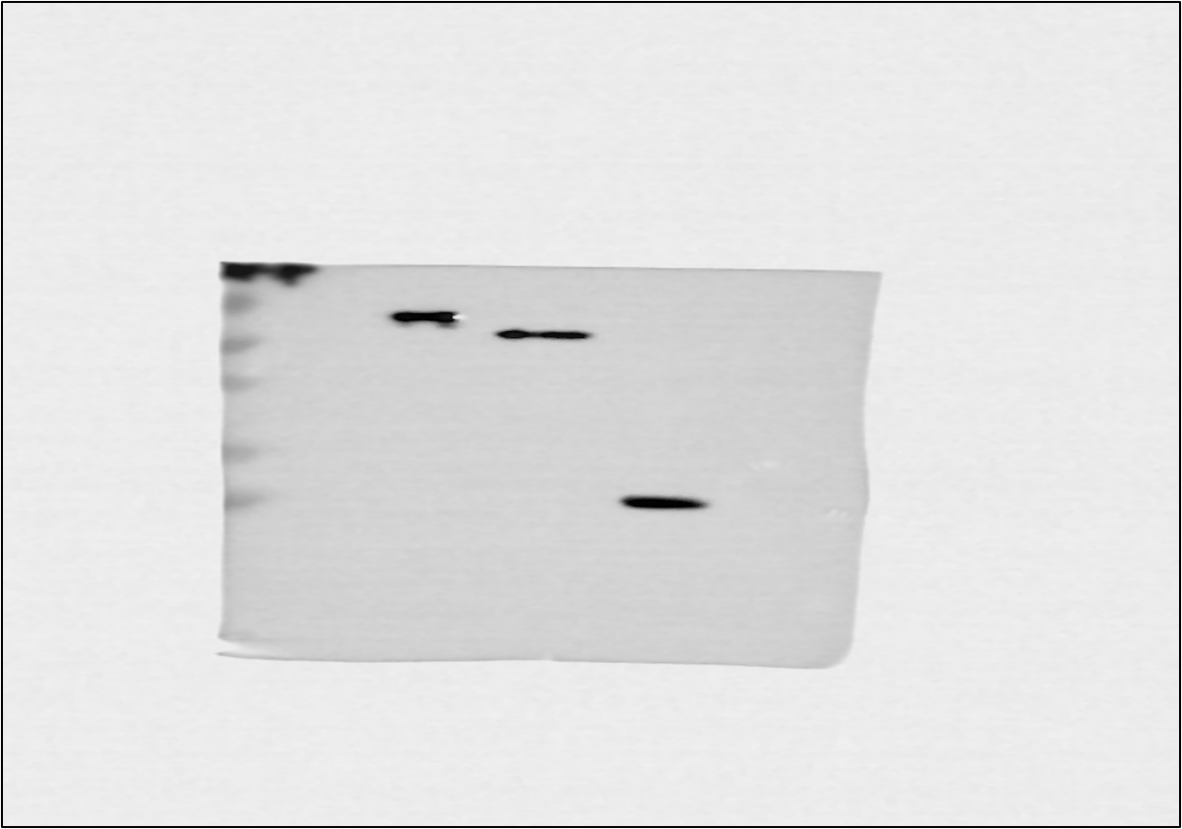

Supplement: Figure 6—source data 2. [file elife-108048-fig6-data2.zip › Figure 6/Figure 6 E-IP-Flag.tif]

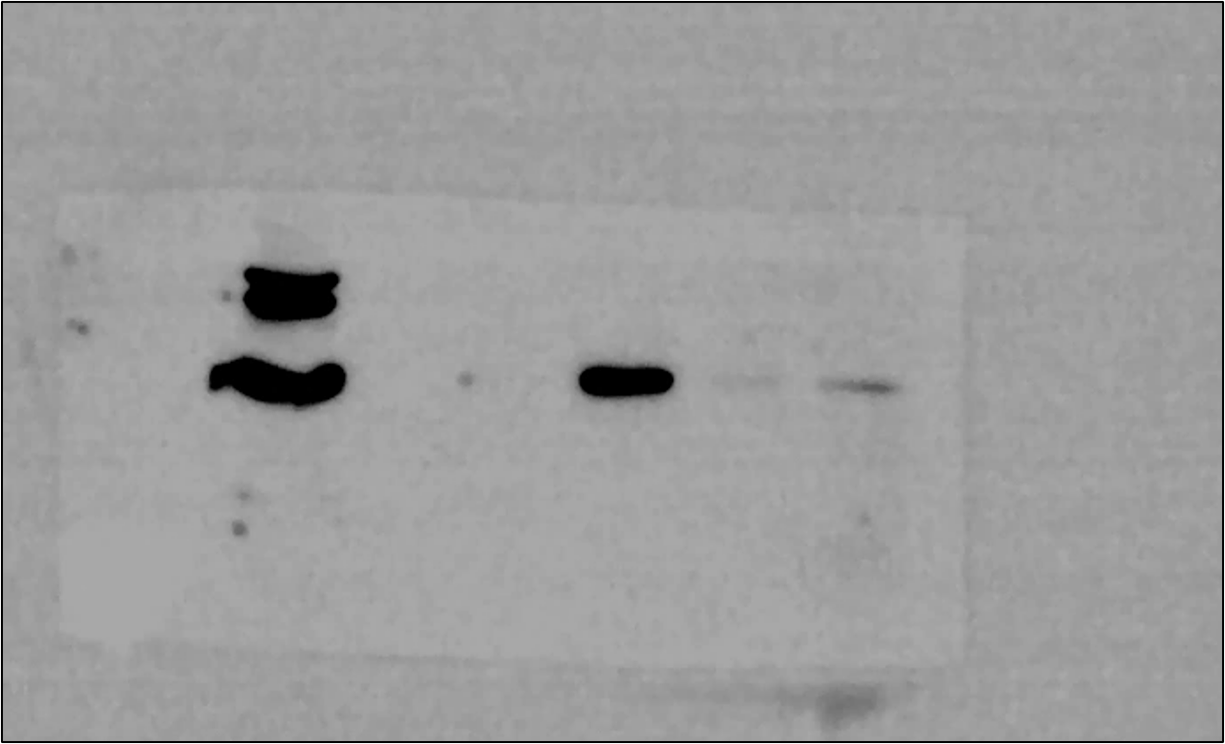

Supplement: Figure 6—source data 2. [file elife-108048-fig6-data2.zip › Figure 6/Figure 6 E-IP-Myc.tif]

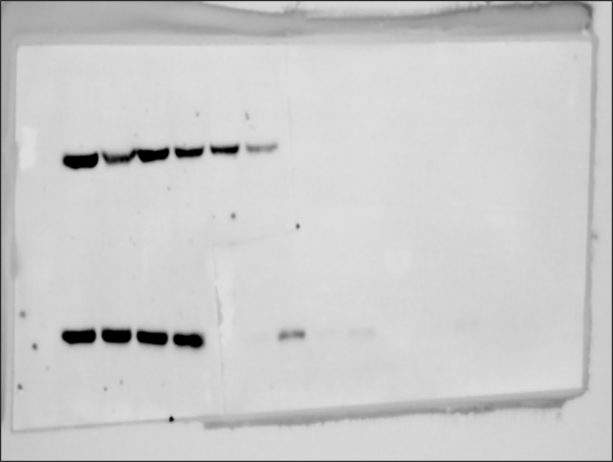

Supplement: Figure 6—source data 2. [file elife-108048-fig6-data2.zip › Figure 6/Figure 6 E-WCL-Myc.tif]

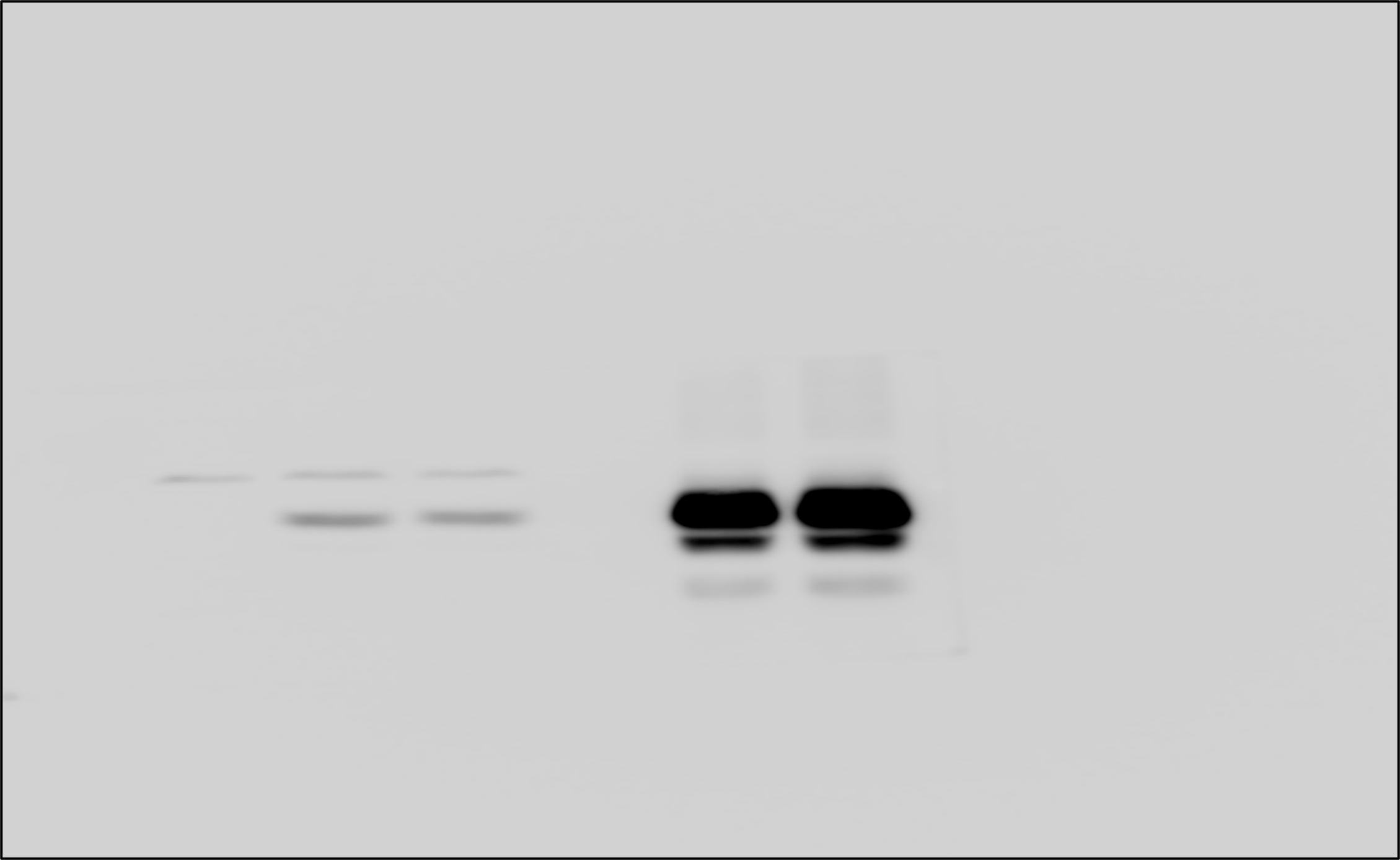

Supplement: Figure 6—source data 2. [file elife-108048-fig6-data2.zip › Figure 6/Figure 6 F-IP-Flag.tif]

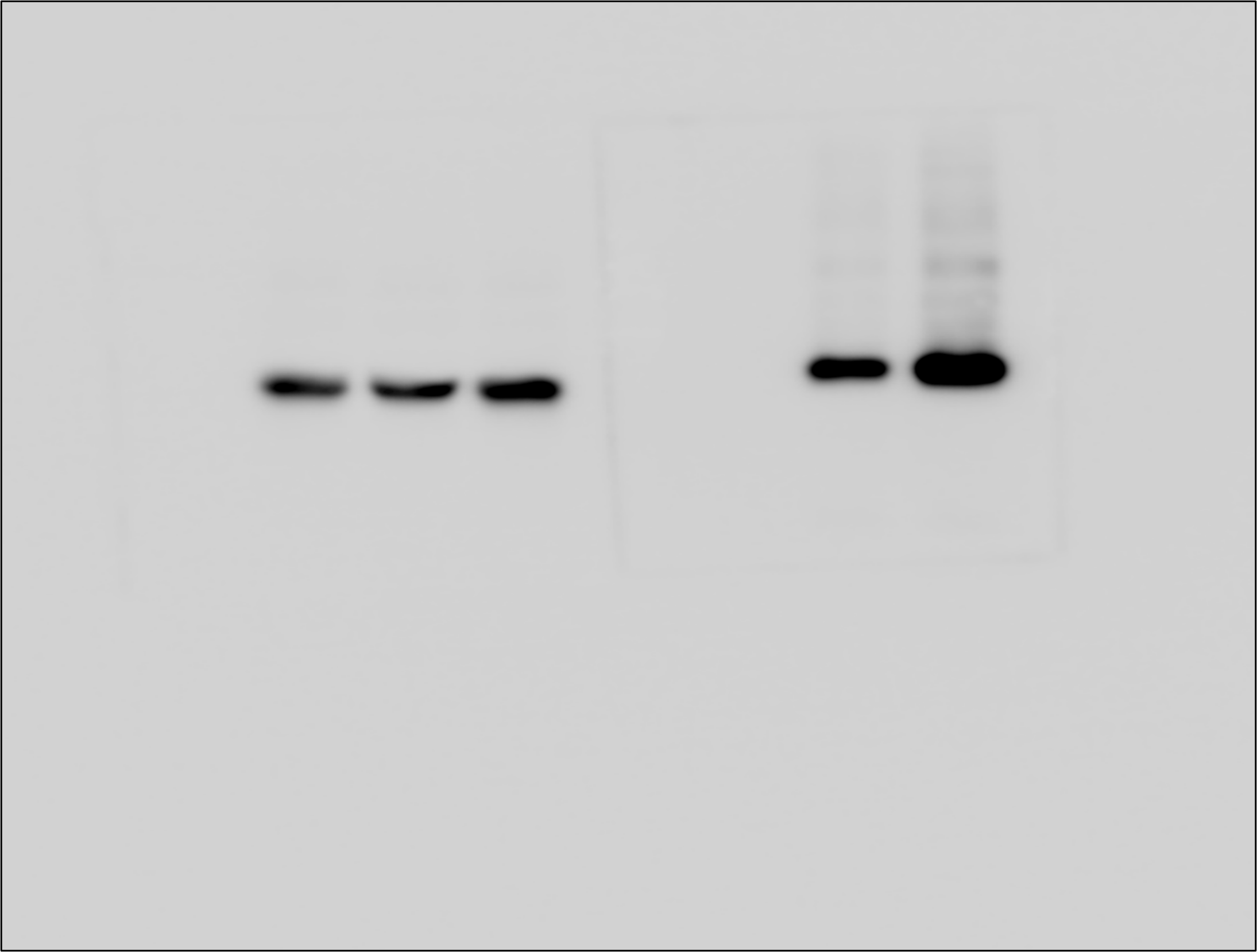

Supplement: Figure 6—source data 2. [file elife-108048-fig6-data2.zip › Figure 6/Figure 6 F-IP-Myc.tif]

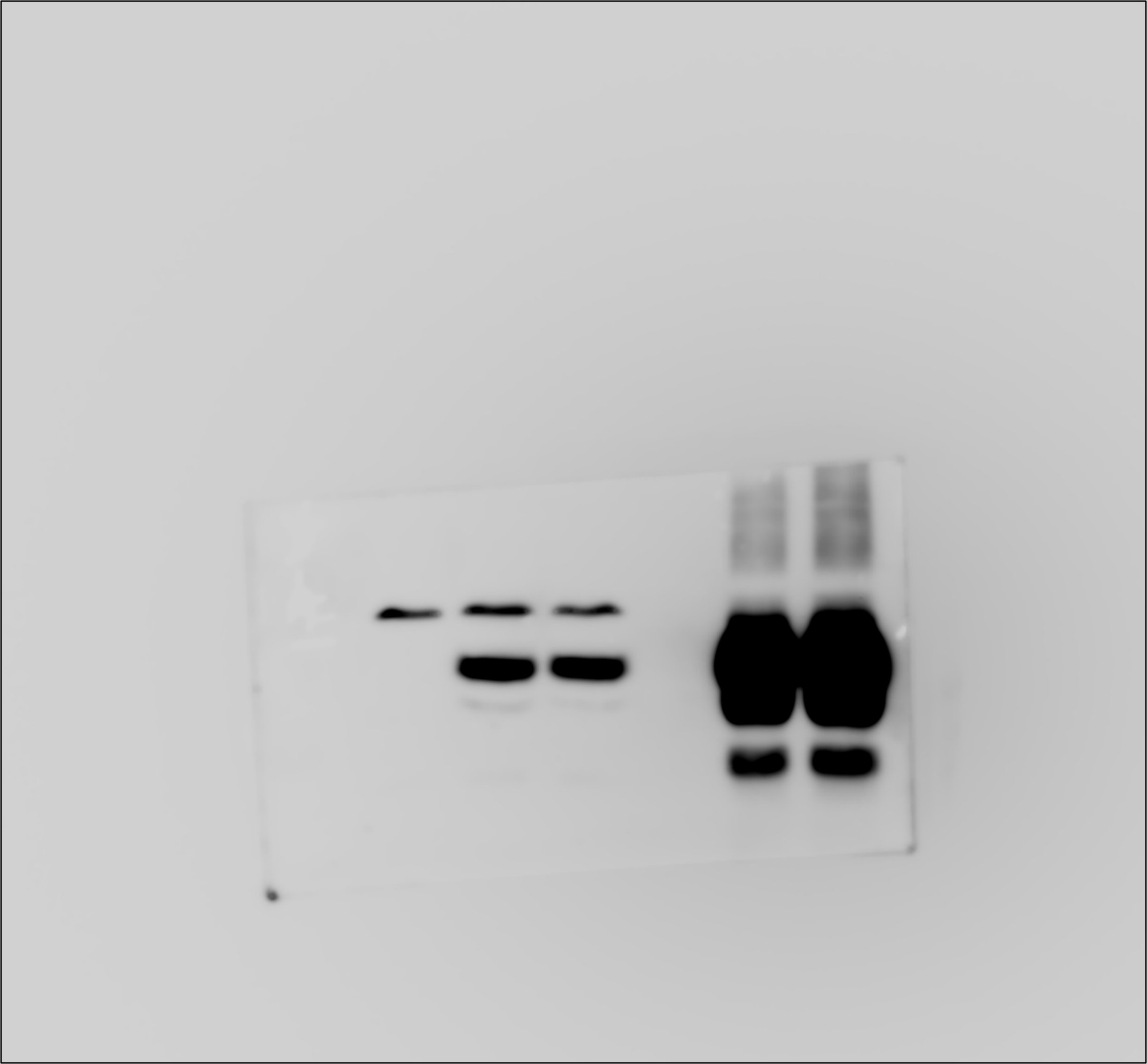

Supplement: Figure 6—source data 2. [file elife-108048-fig6-data2.zip › Figure 6/Figure 6 F-WCL-Flag.tif]

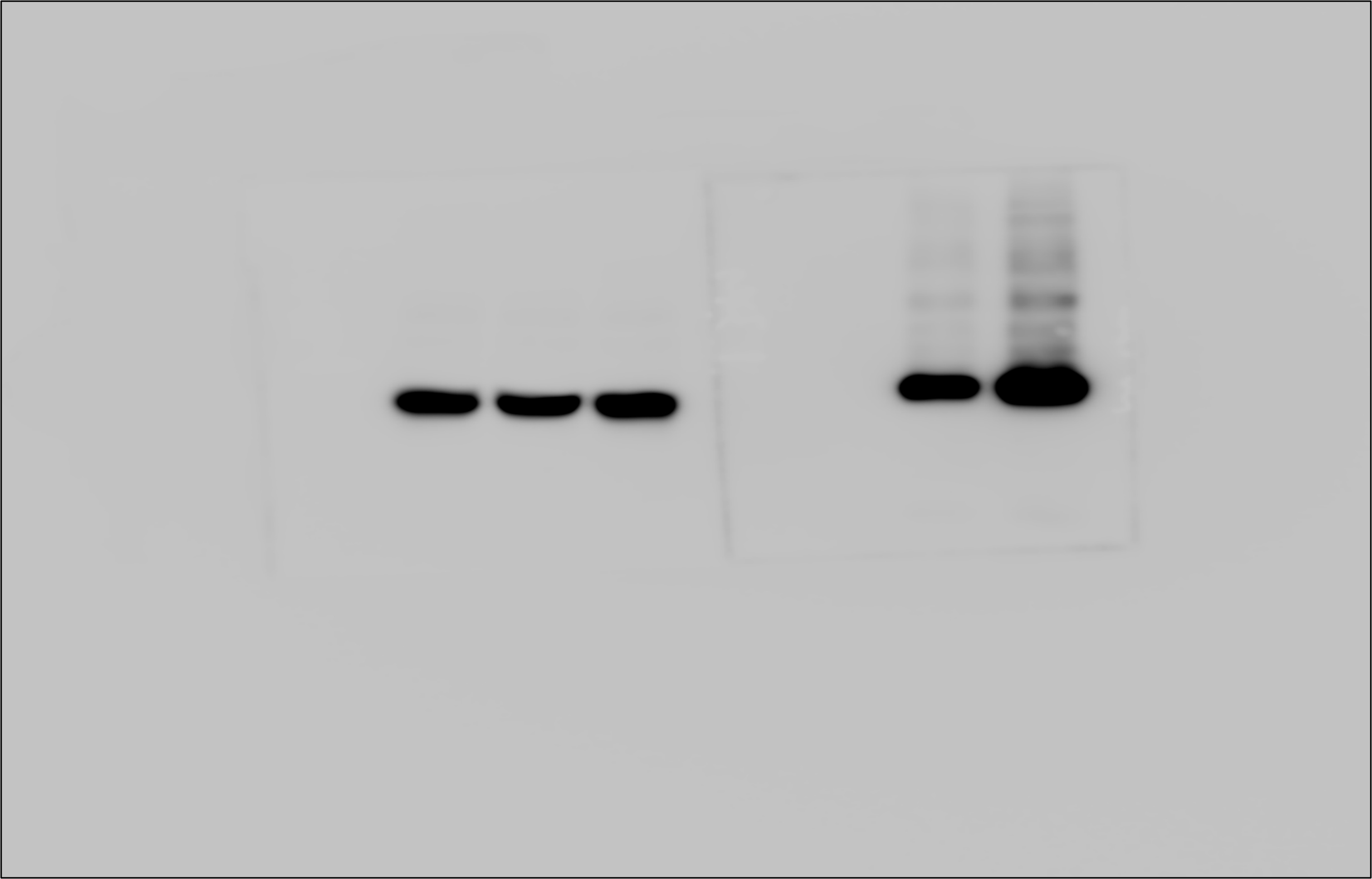

Supplement: Figure 6—source data 2. [file elife-108048-fig6-data2.zip › Figure 6/Figure 6 F-WCL-Myc.tif]

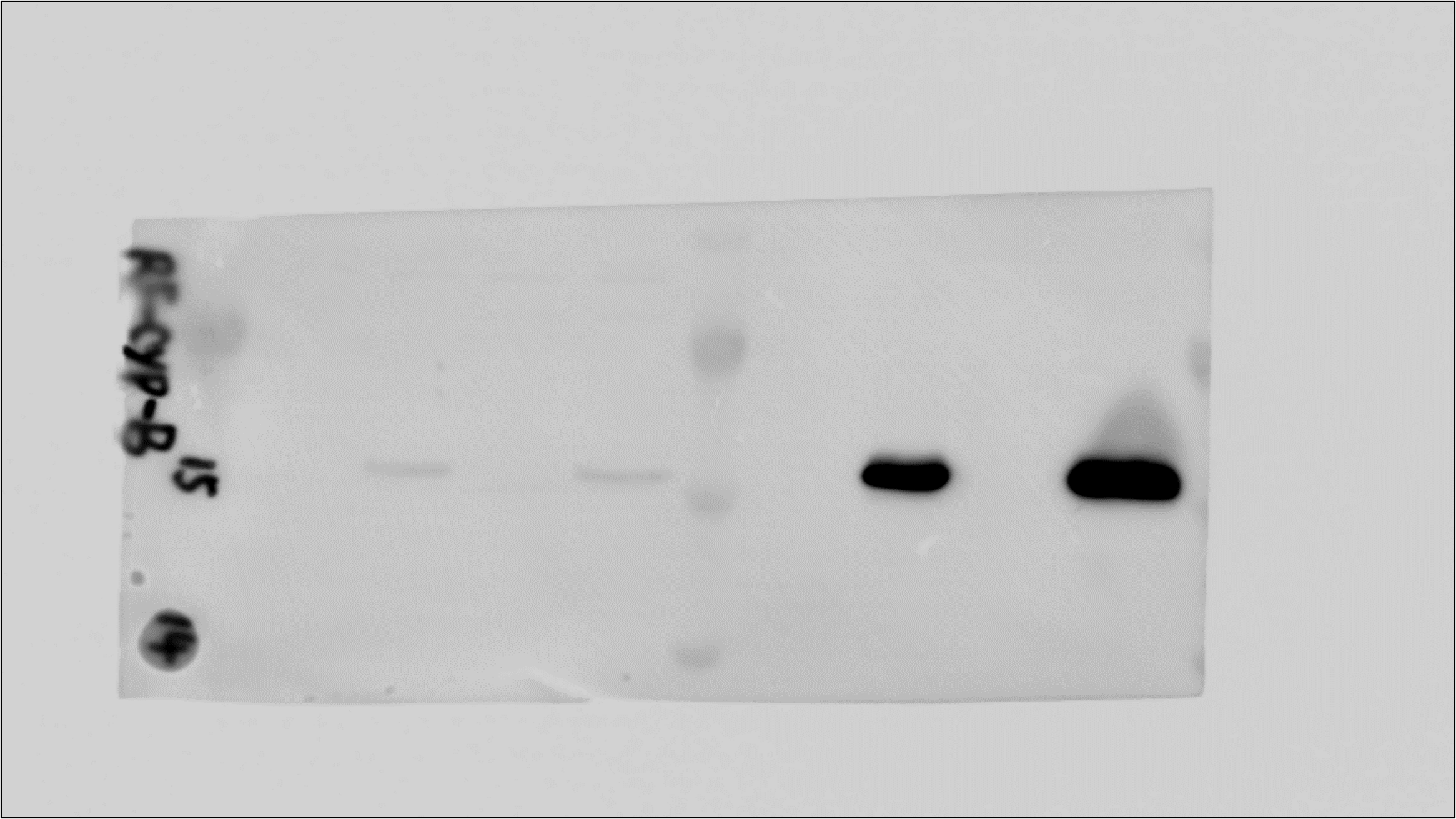

Supplement: Figure 6—source data 2. [file elife-108048-fig6-data2.zip › Figure 6/Figure 6 G-IP-Flag.tif]

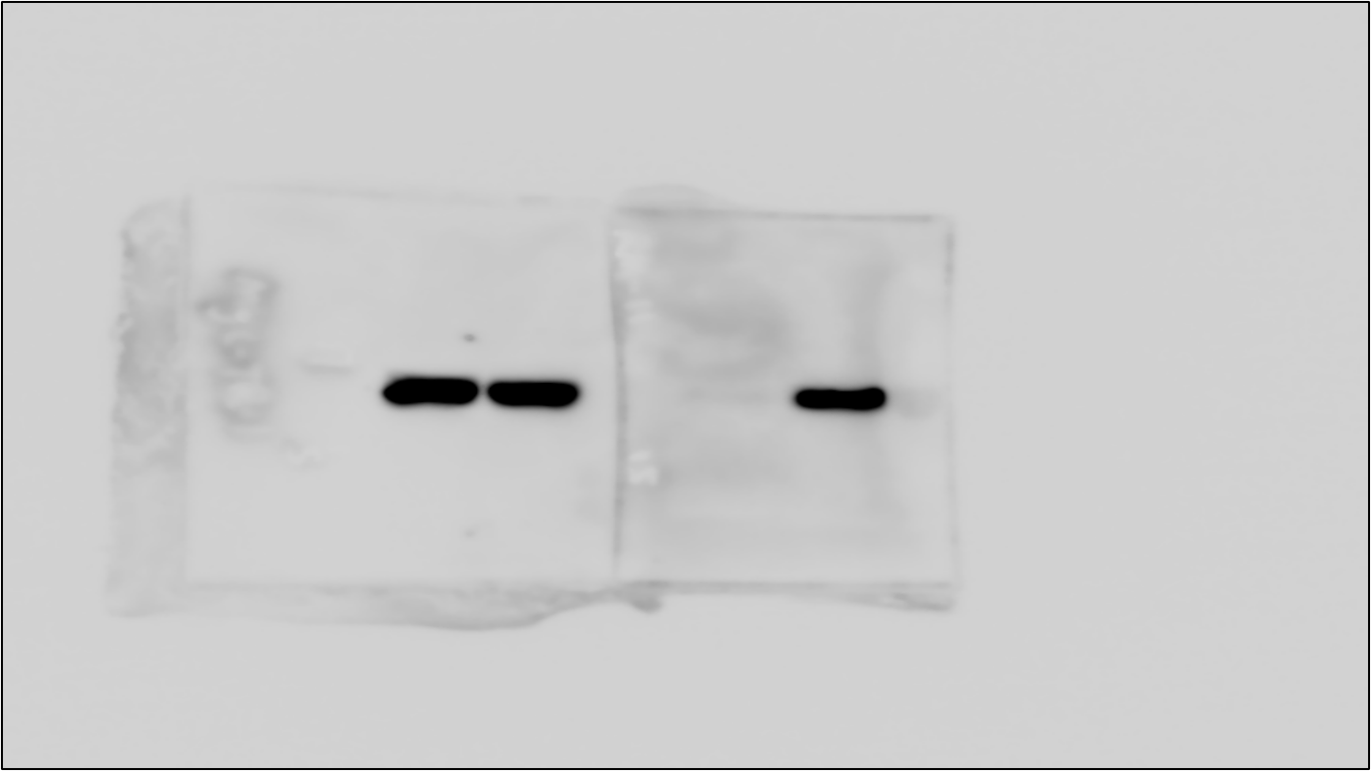

Supplement: Figure 6—source data 2. [file elife-108048-fig6-data2.zip › Figure 6/Figure 6 G-IP-Myc.tif]

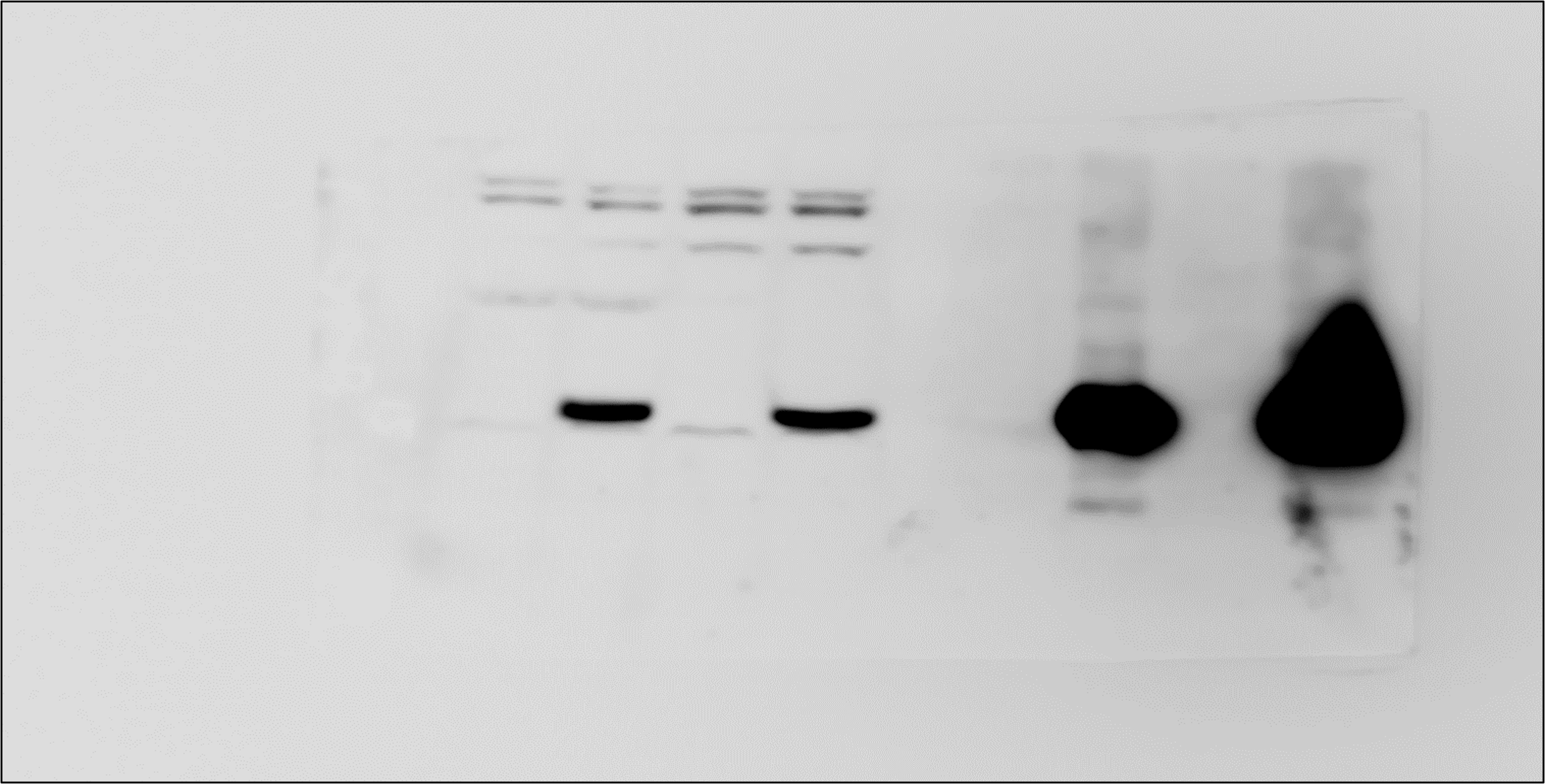

Supplement: Figure 6—source data 2. [file elife-108048-fig6-data2.zip › Figure 6/Figure 6 G-WCL-Flag.tif]

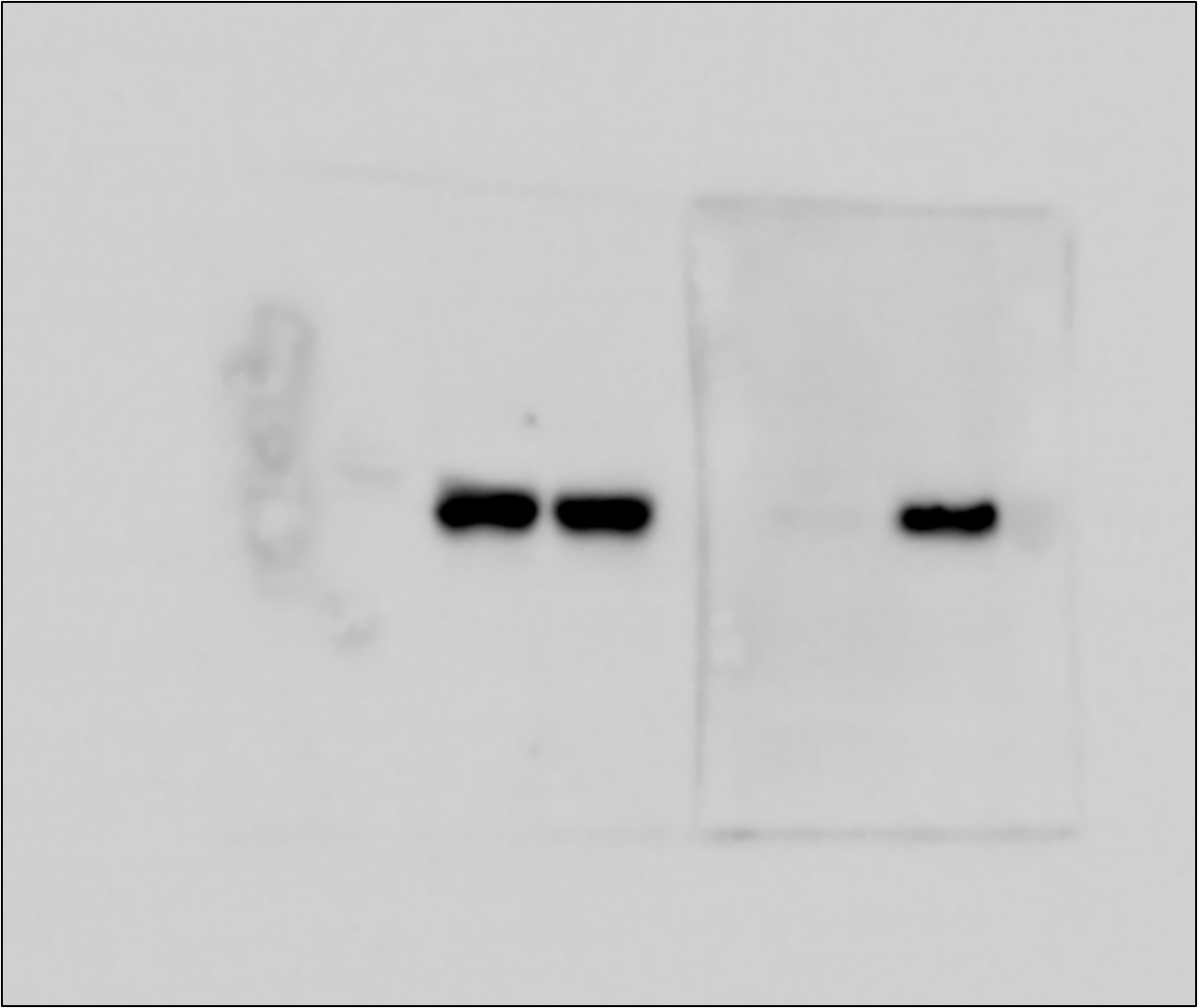

Supplement: Figure 6—source data 2. [file elife-108048-fig6-data2.zip › Figure 6/Figure 6 G-WCL-Myc.tif]

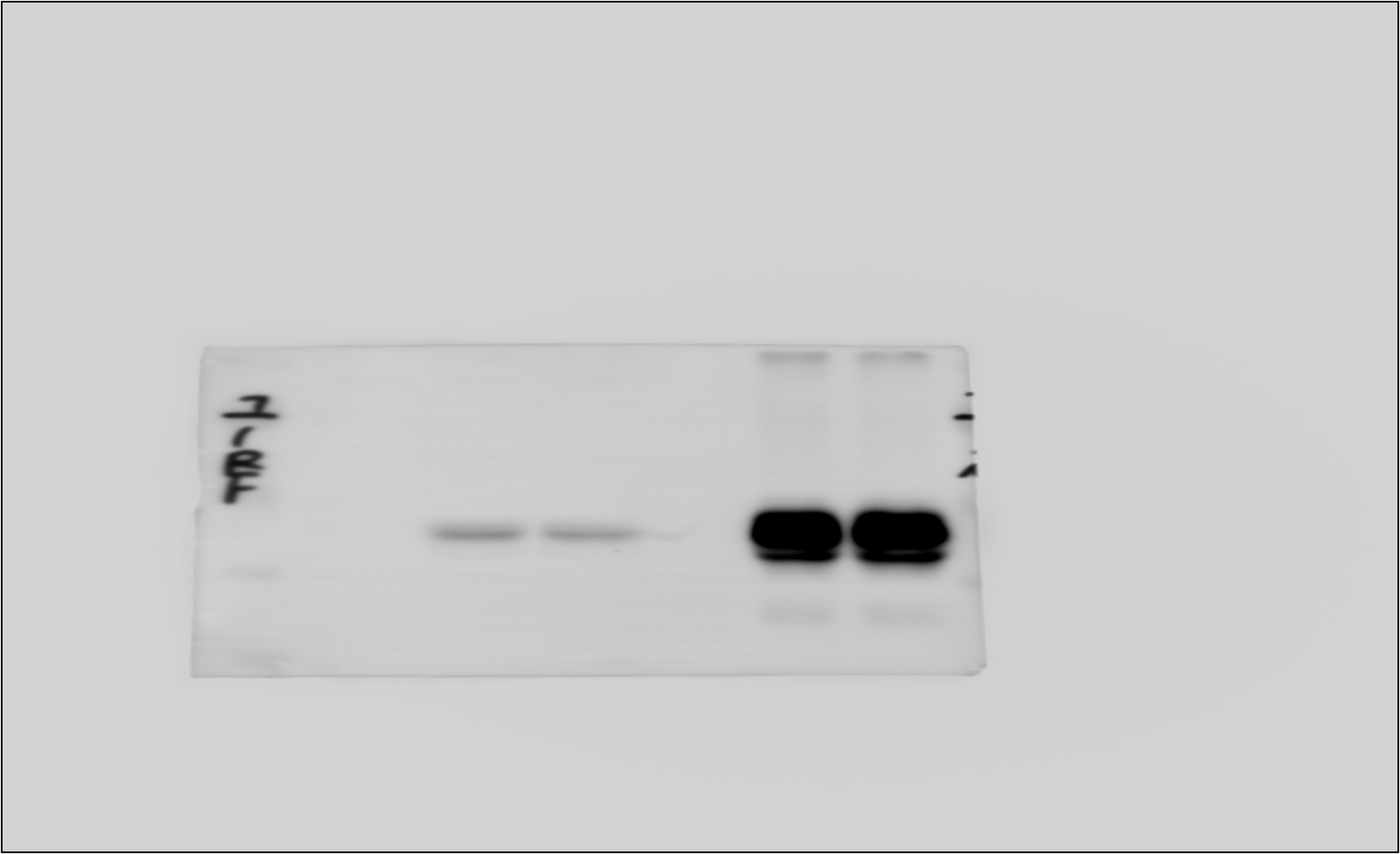

Supplement: Figure 6—source data 2. [file elife-108048-fig6-data2.zip › Figure 6/Figure 6 K-IP-Flag.tif]

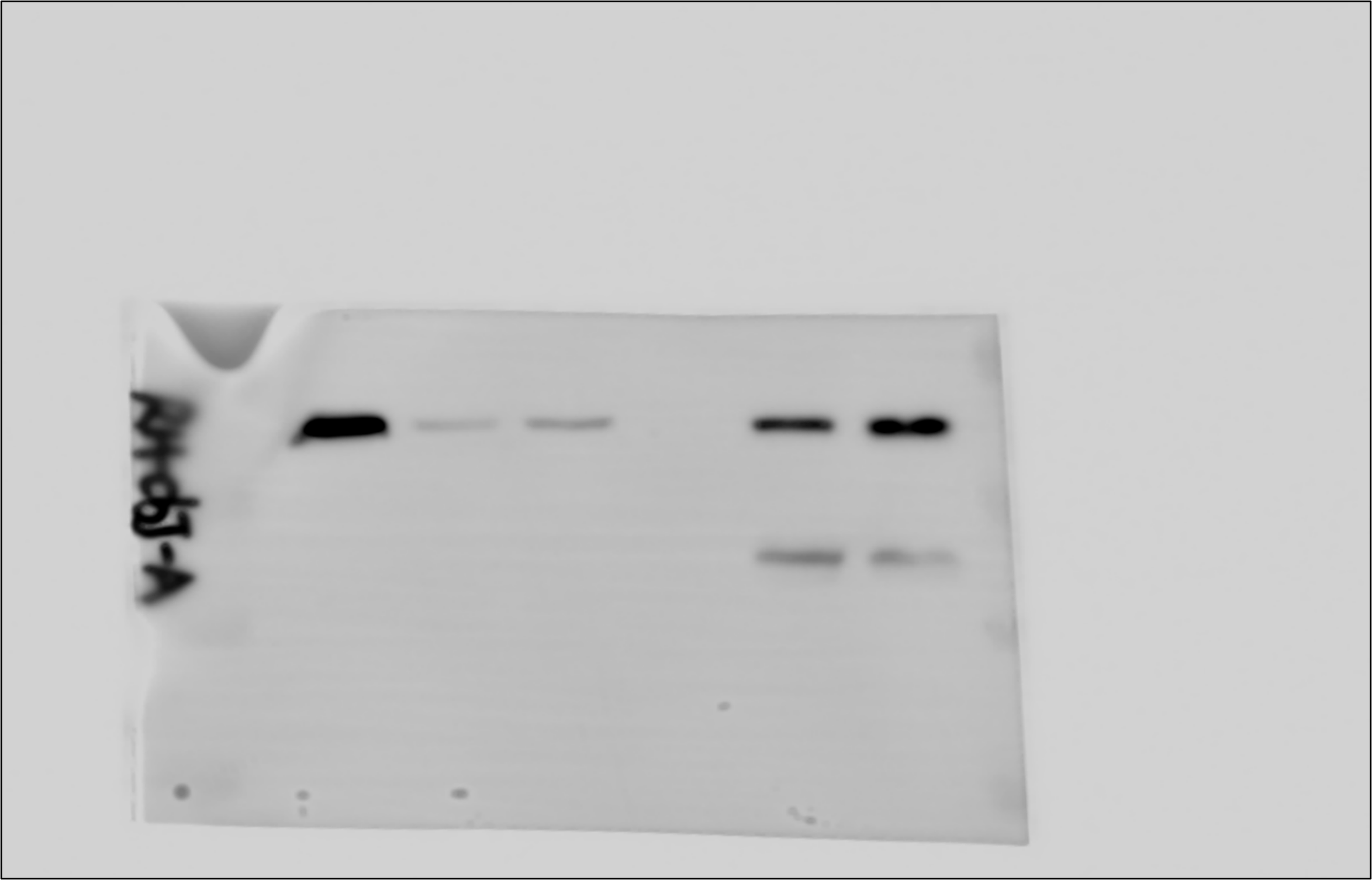

Supplement: Figure 6—source data 2. [file elife-108048-fig6-data2.zip › Figure 6/Figure 6 K-IP-HA.tif]

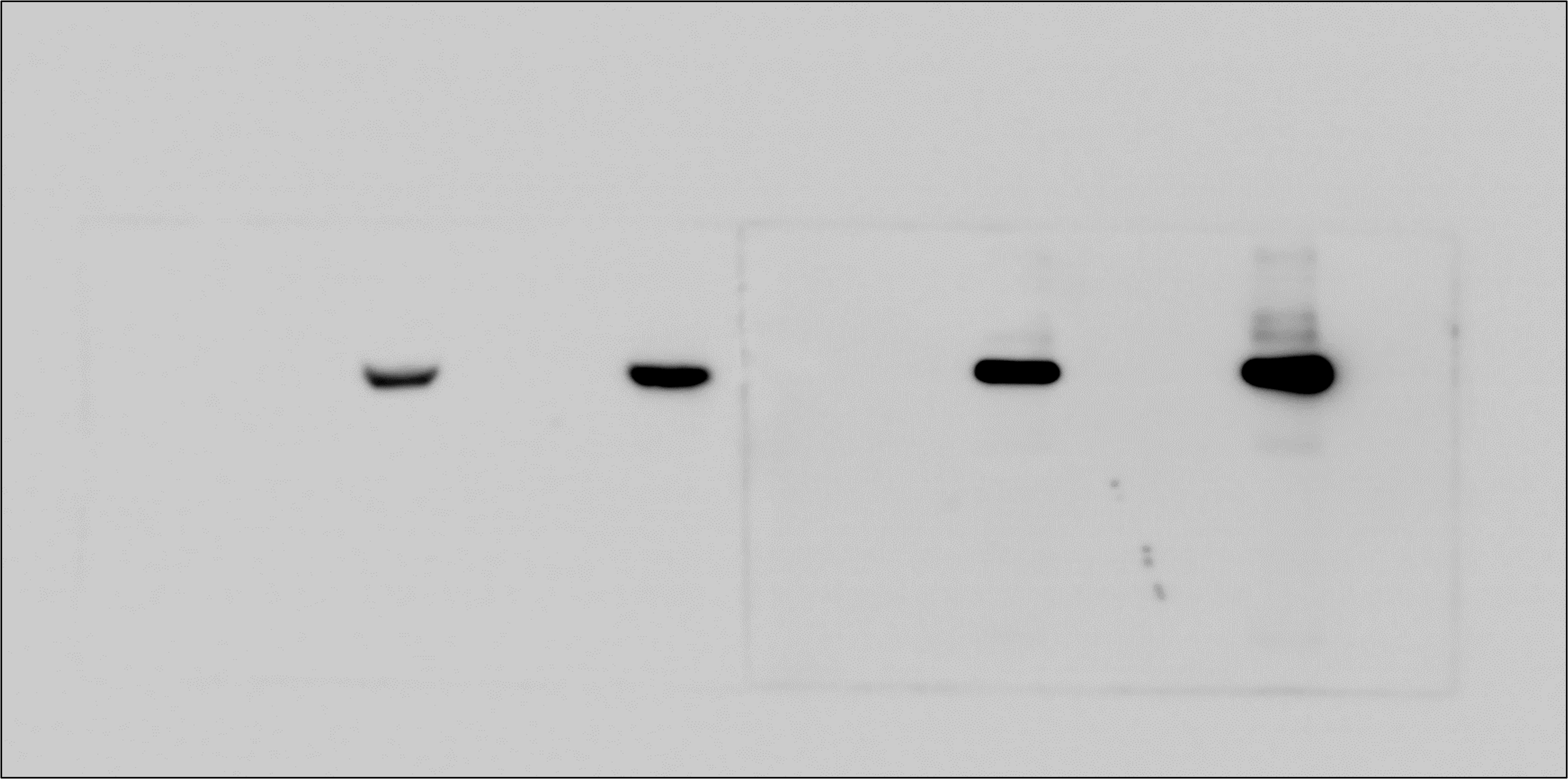

Supplement: Figure 6—source data 2. [file elife-108048-fig6-data2.zip › Figure 6/Figure 6 K-IP-Myc.tif]

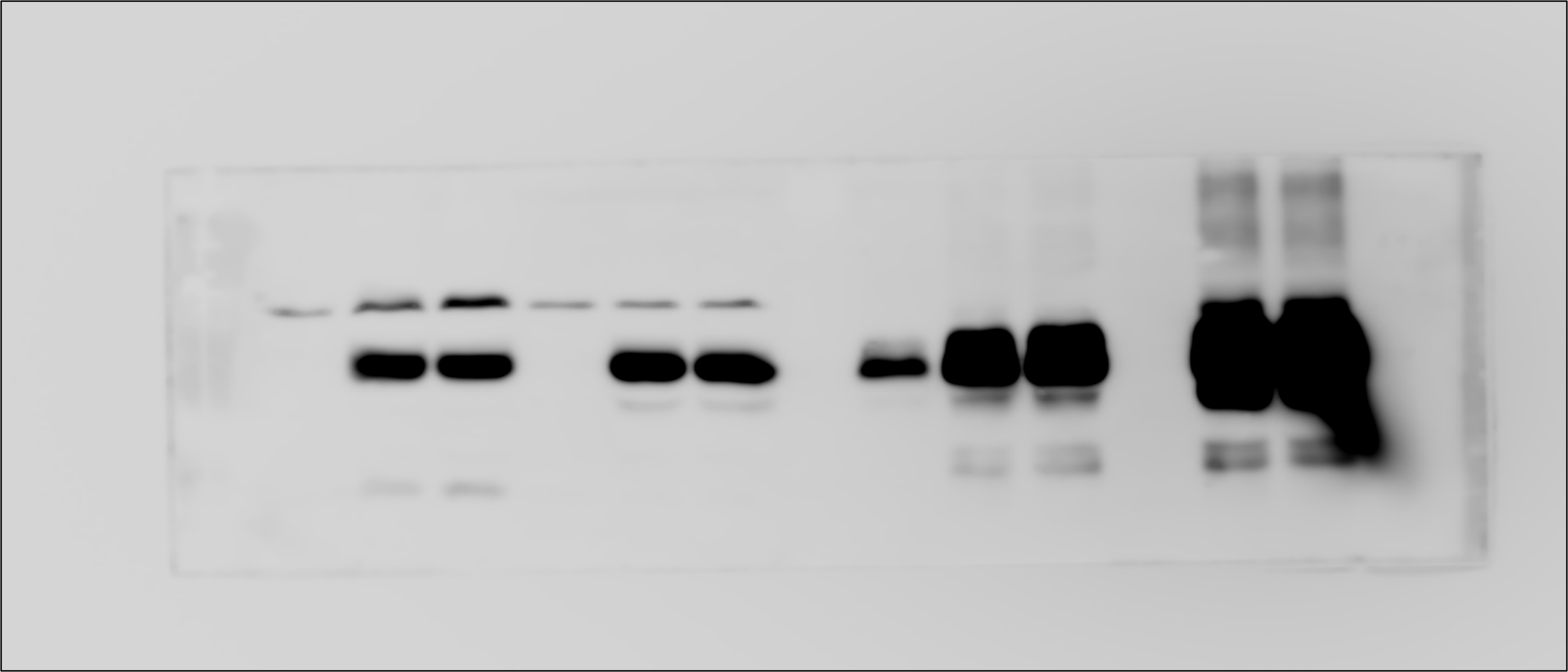

Supplement: Figure 6—source data 2. [file elife-108048-fig6-data2.zip › Figure 6/Figure 6 K-WCL-Flag.tif]

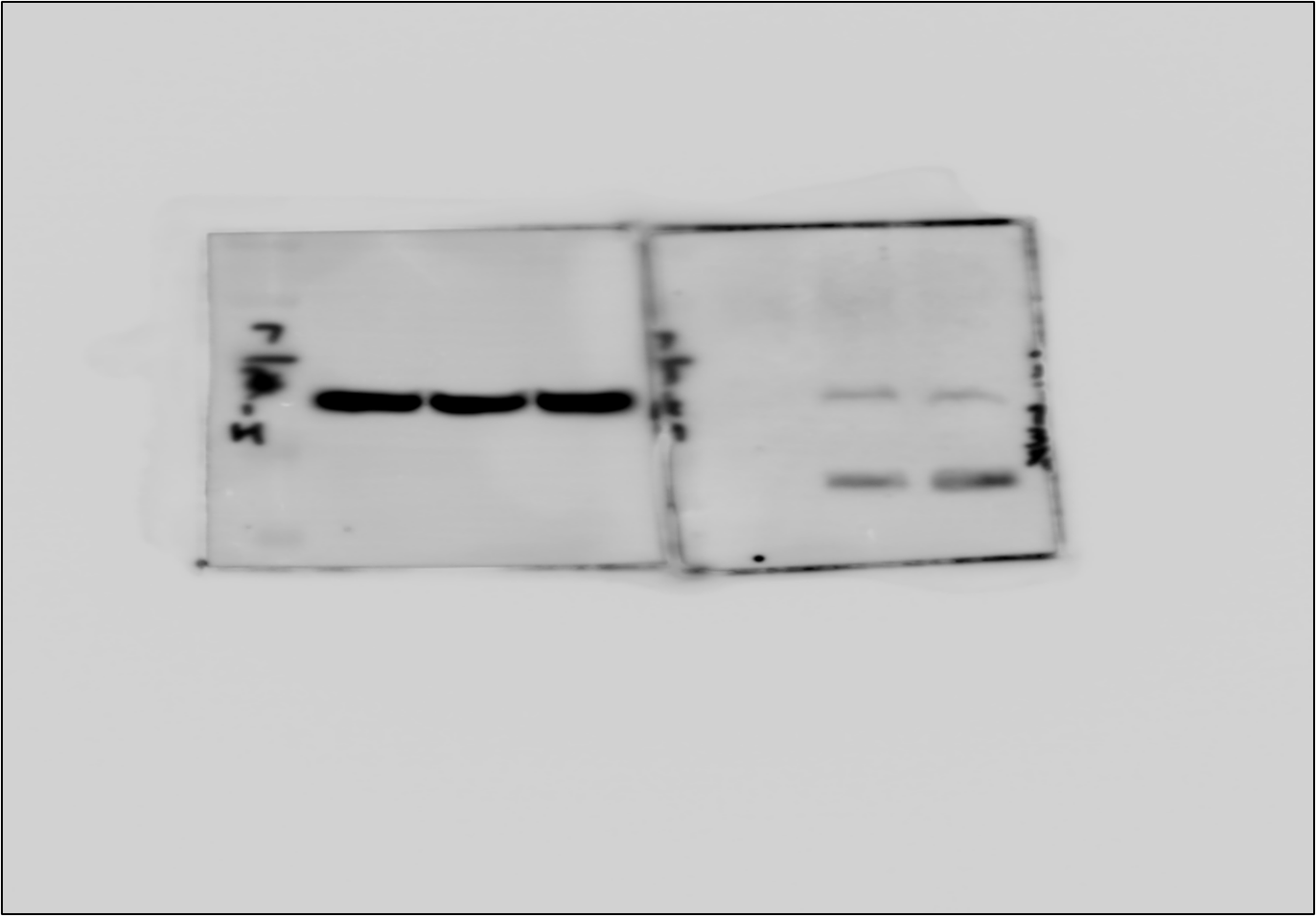

Supplement: Figure 6—source data 2. [file elife-108048-fig6-data2.zip › Figure 6/Figure 6 K-WCL-HA.tif]

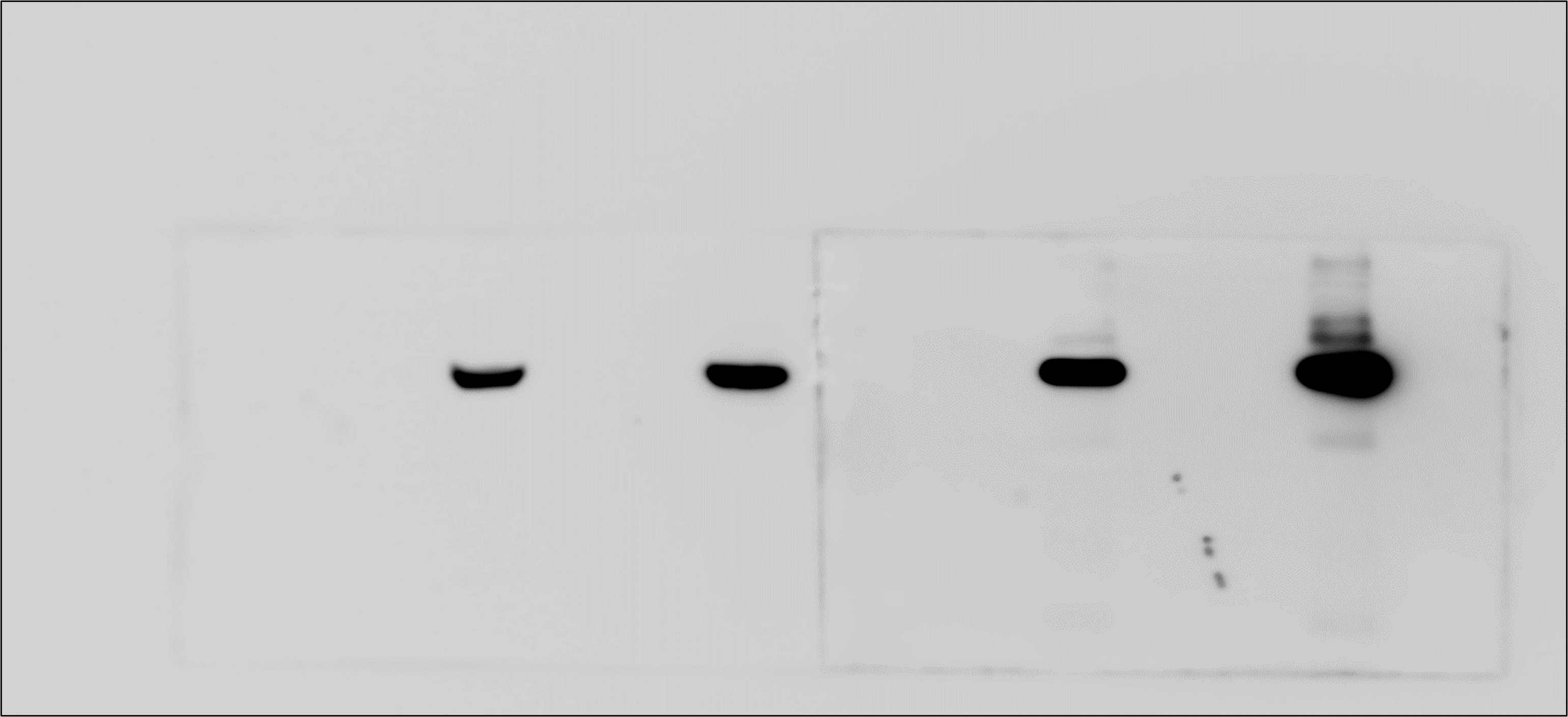

Supplement: Figure 6—source data 2. [file elife-108048-fig6-data2.zip › Figure 6/Figure 6 K-WCL-Myc.tif]

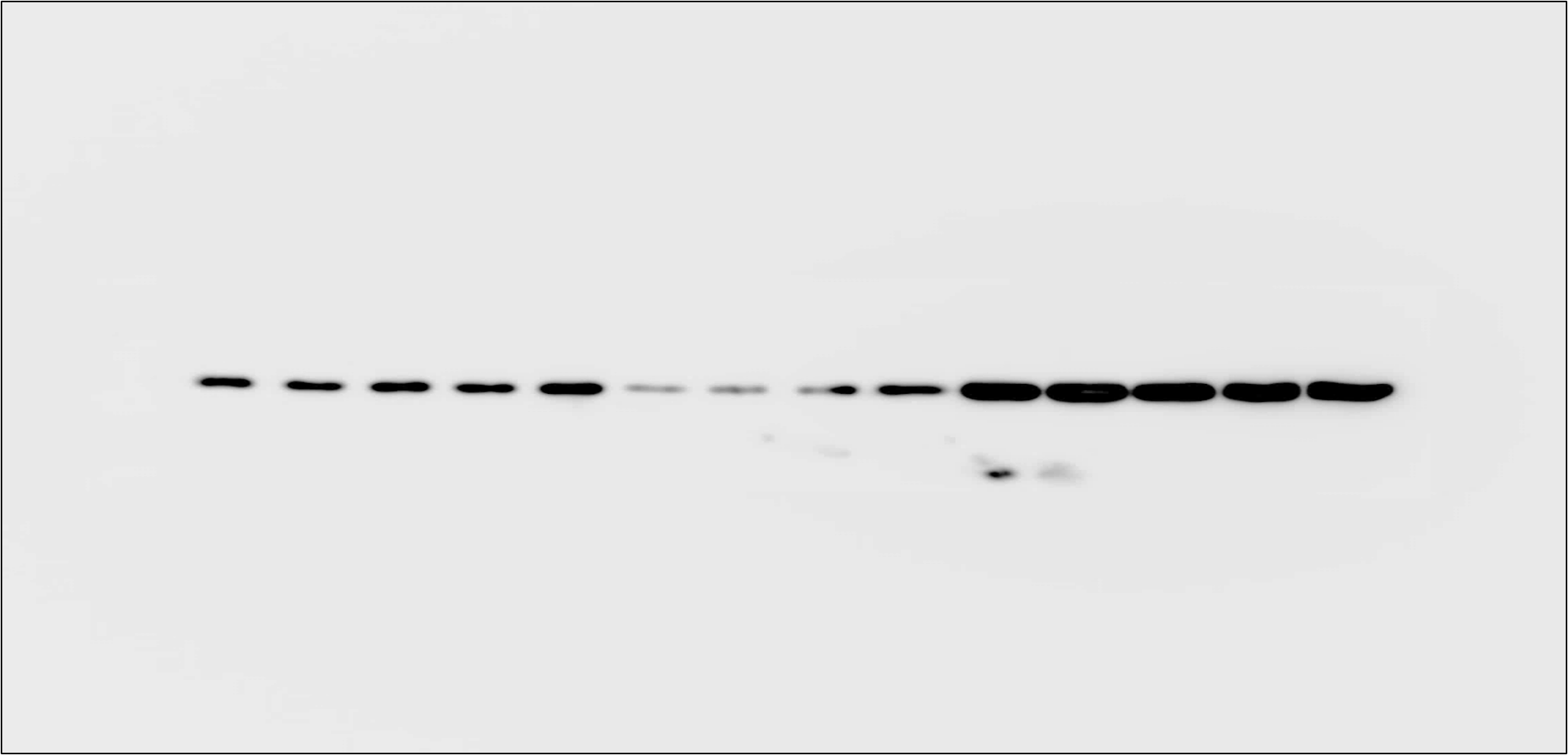

Supplement: Figure 6—source data 2. [file elife-108048-fig6-data2.zip › Figure 6/Figure 6 N-Actin-2.tif]

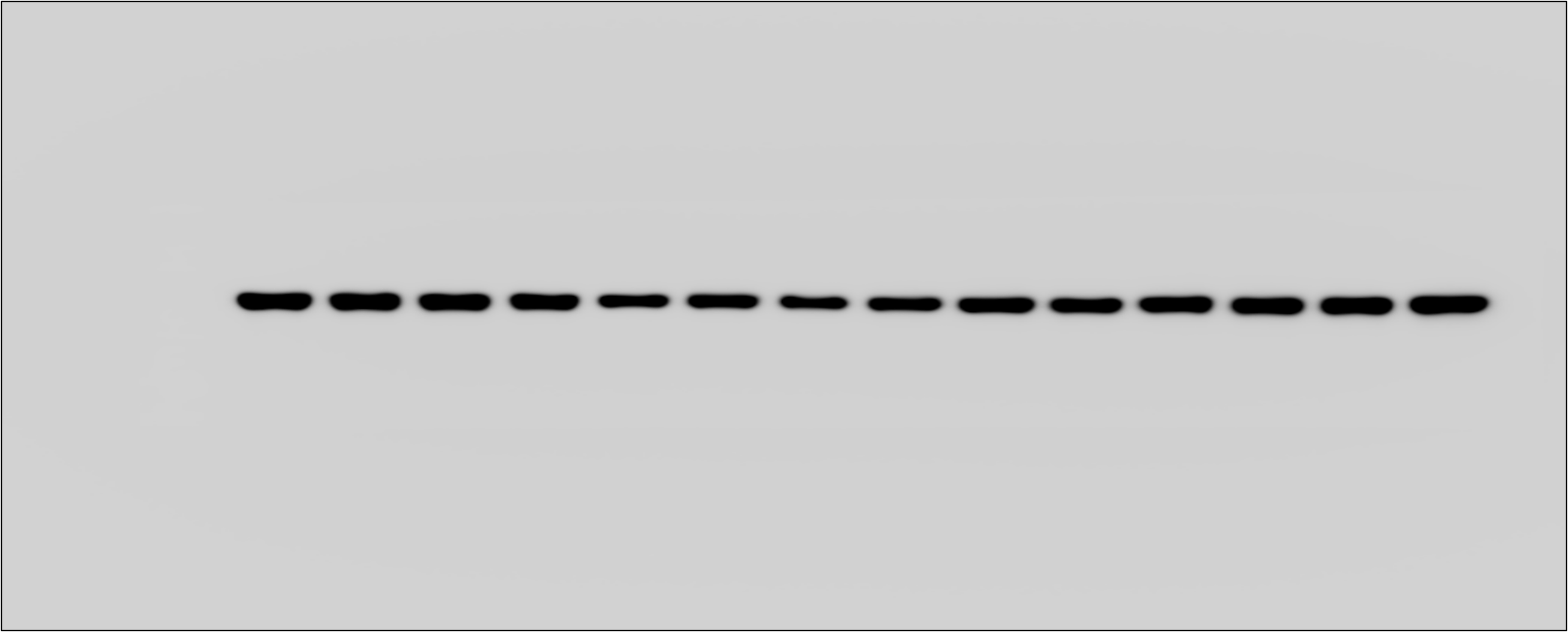

Supplement: Figure 6—source data 2. [file elife-108048-fig6-data2.zip › Figure 6/Figure 6 N-Actin.tif]

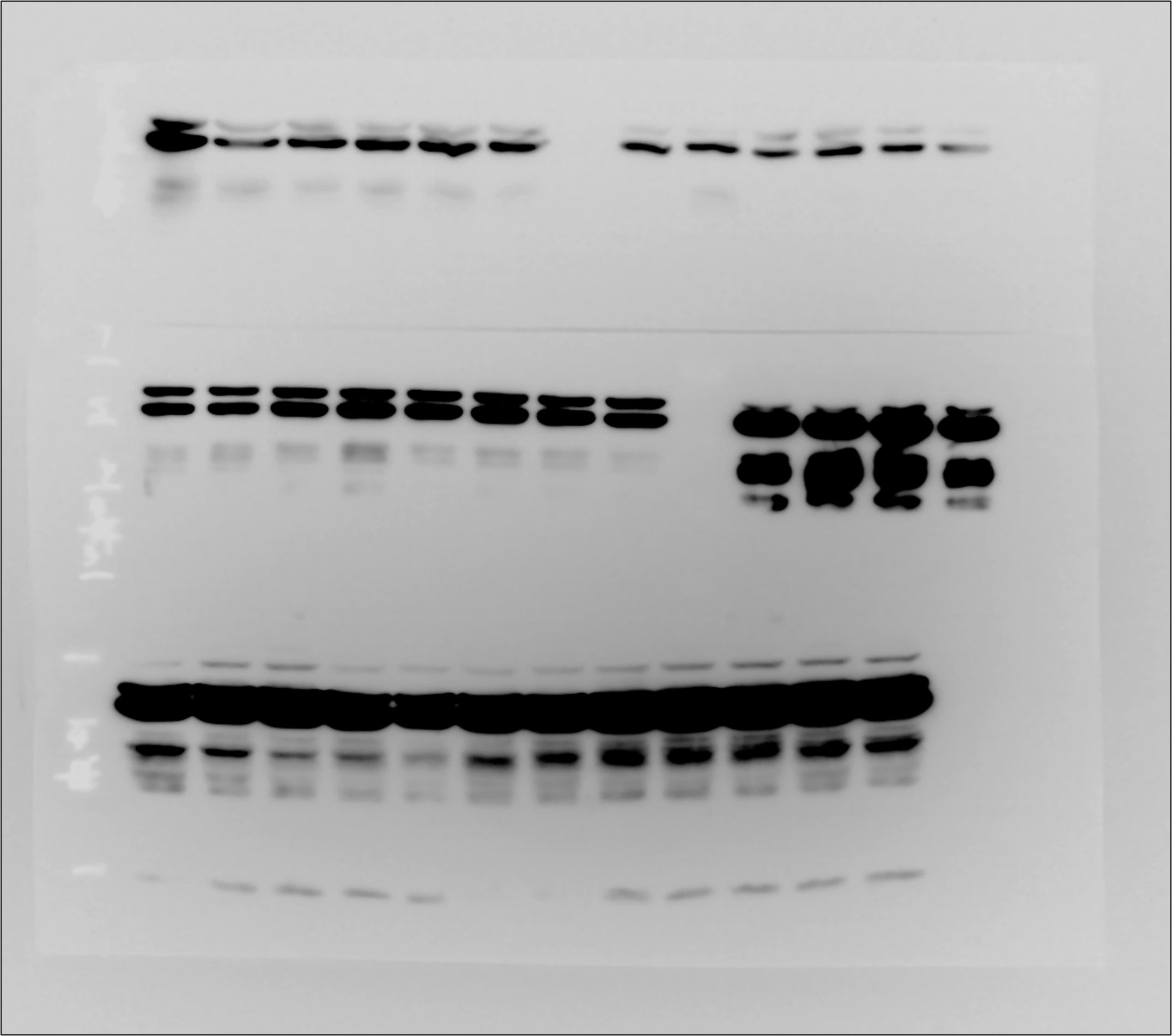

Supplement: Figure 6—source data 2. [file elife-108048-fig6-data2.zip › Figure 6/Figure 6 N-Flag-btr32-2.tif]

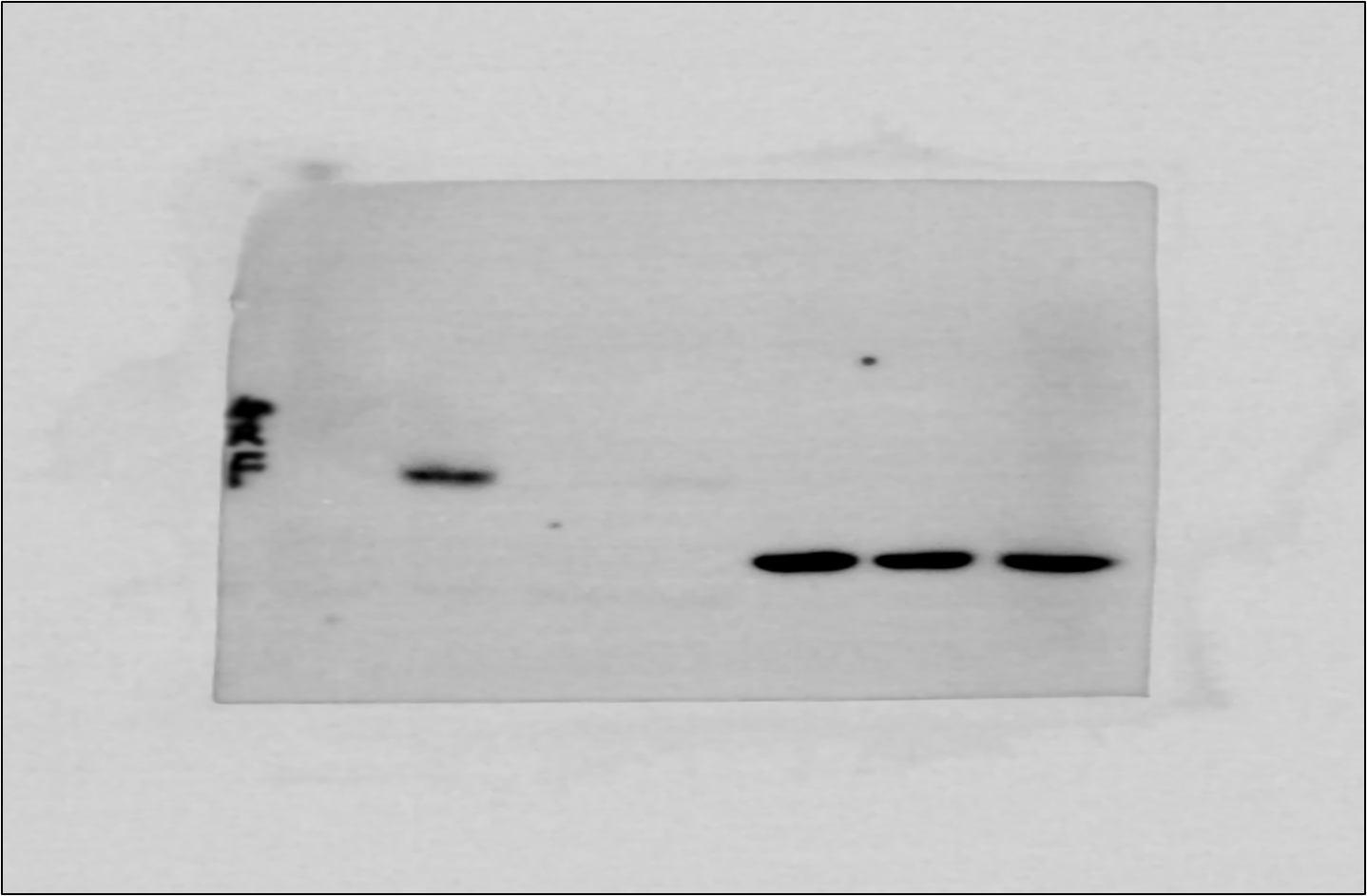

Supplement: Figure 6—source data 2. [file elife-108048-fig6-data2.zip › Figure 6/Figure 6 N-Flag-STING.tif]

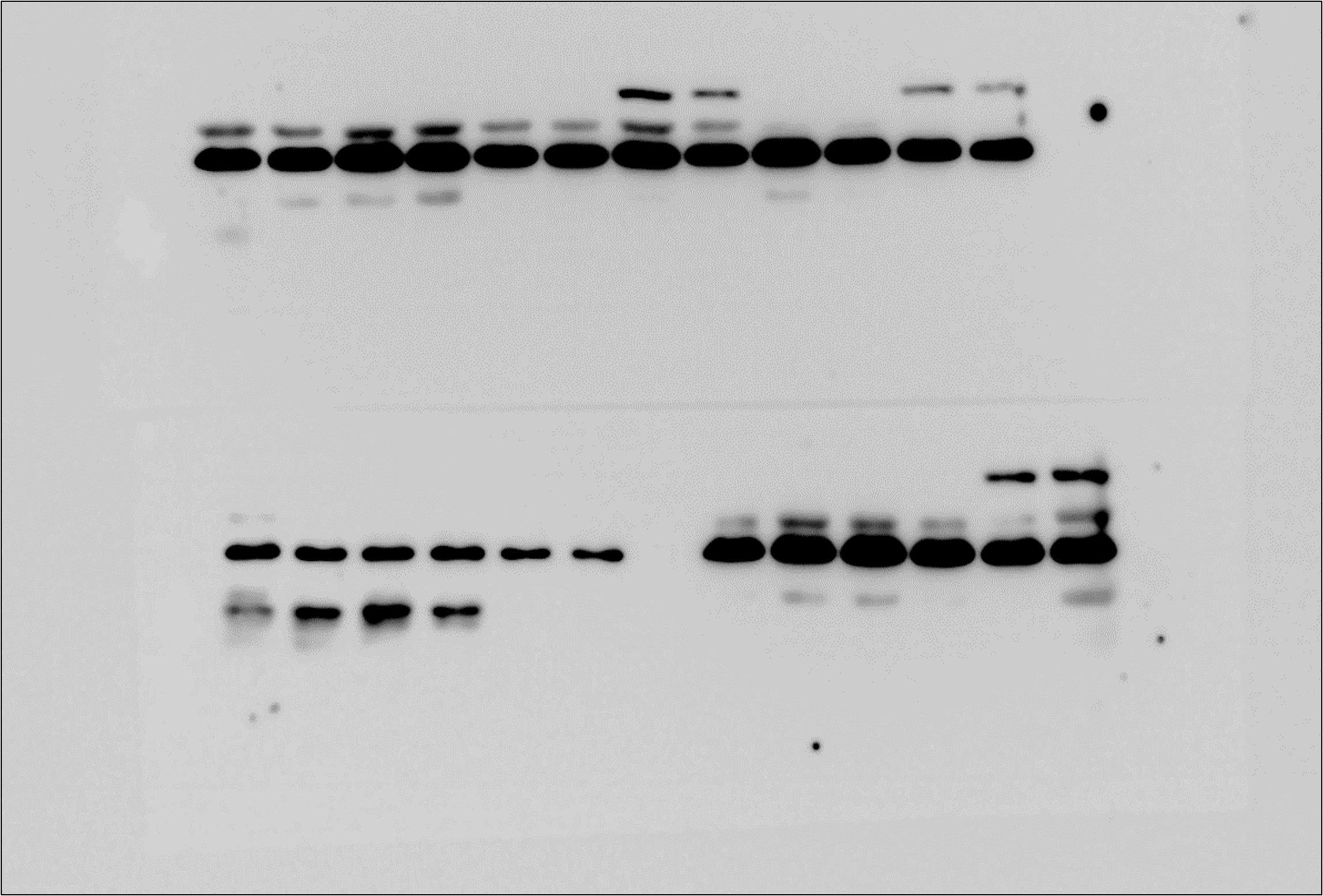

Supplement: Figure 6—source data 2. [file elife-108048-fig6-data2.zip › Figure 6/Figure 6 N-Flag.tif]

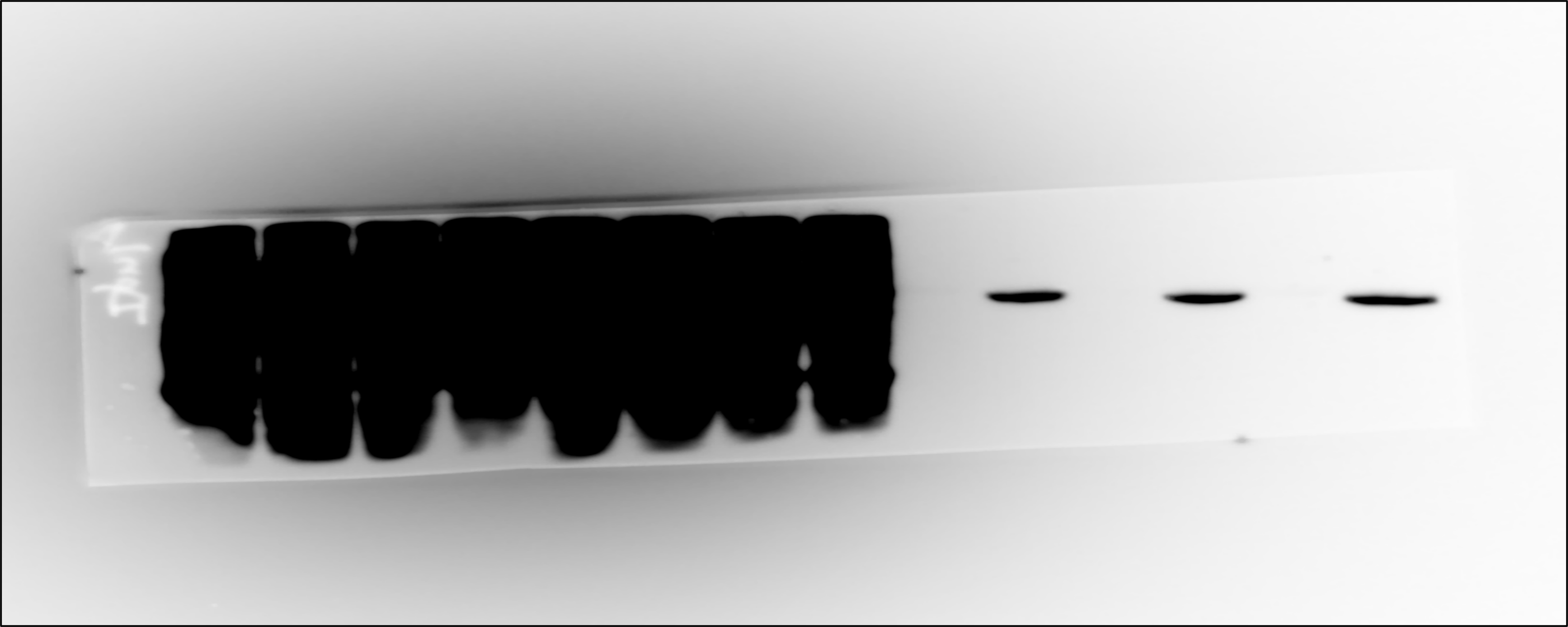

Supplement: Figure 6—source data 2. [file elife-108048-fig6-data2.zip › Figure 6/Figure 6 N-HA-2.tif]

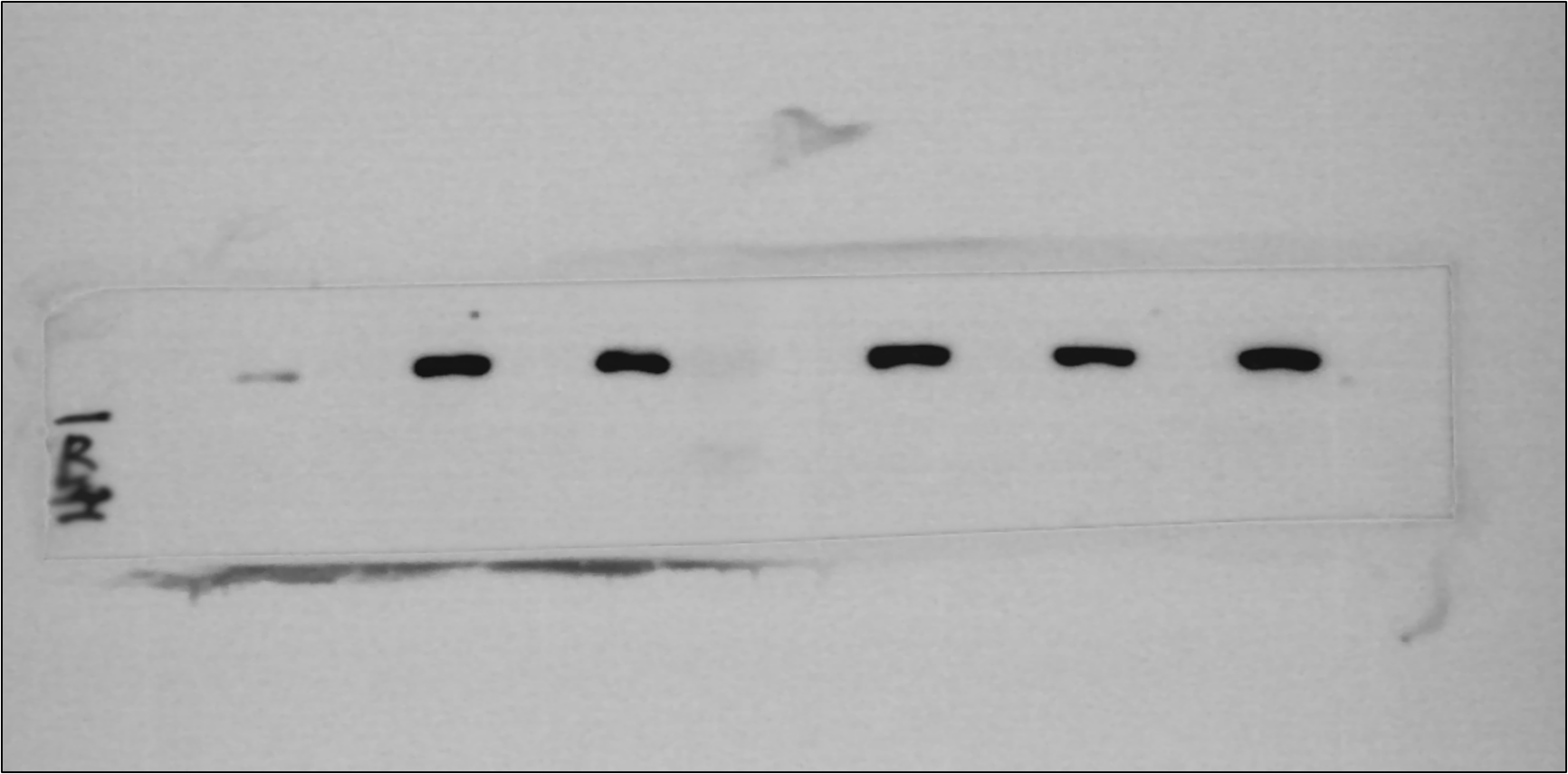

Supplement: Figure 6—source data 2. [file elife-108048-fig6-data2.zip › Figure 6/Figure 6 N-HA.tif]

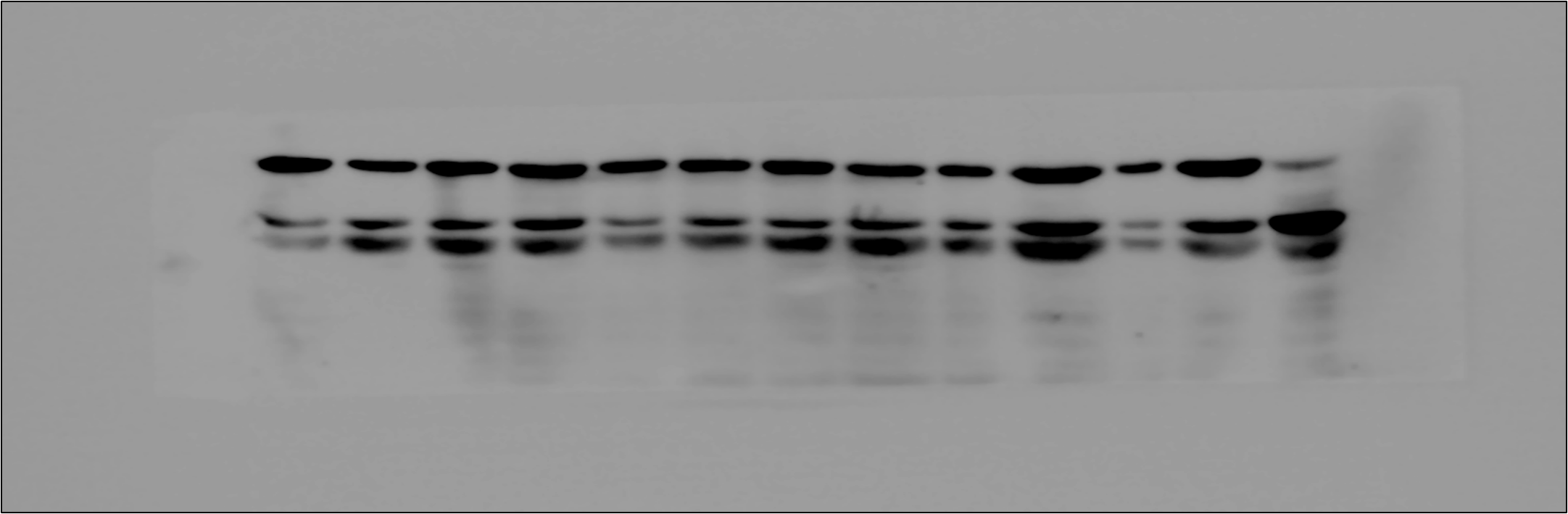

Supplement: Figure 6—source data 2. [file elife-108048-fig6-data2.zip › Figure 6/Figure 6 N-STING.tif]

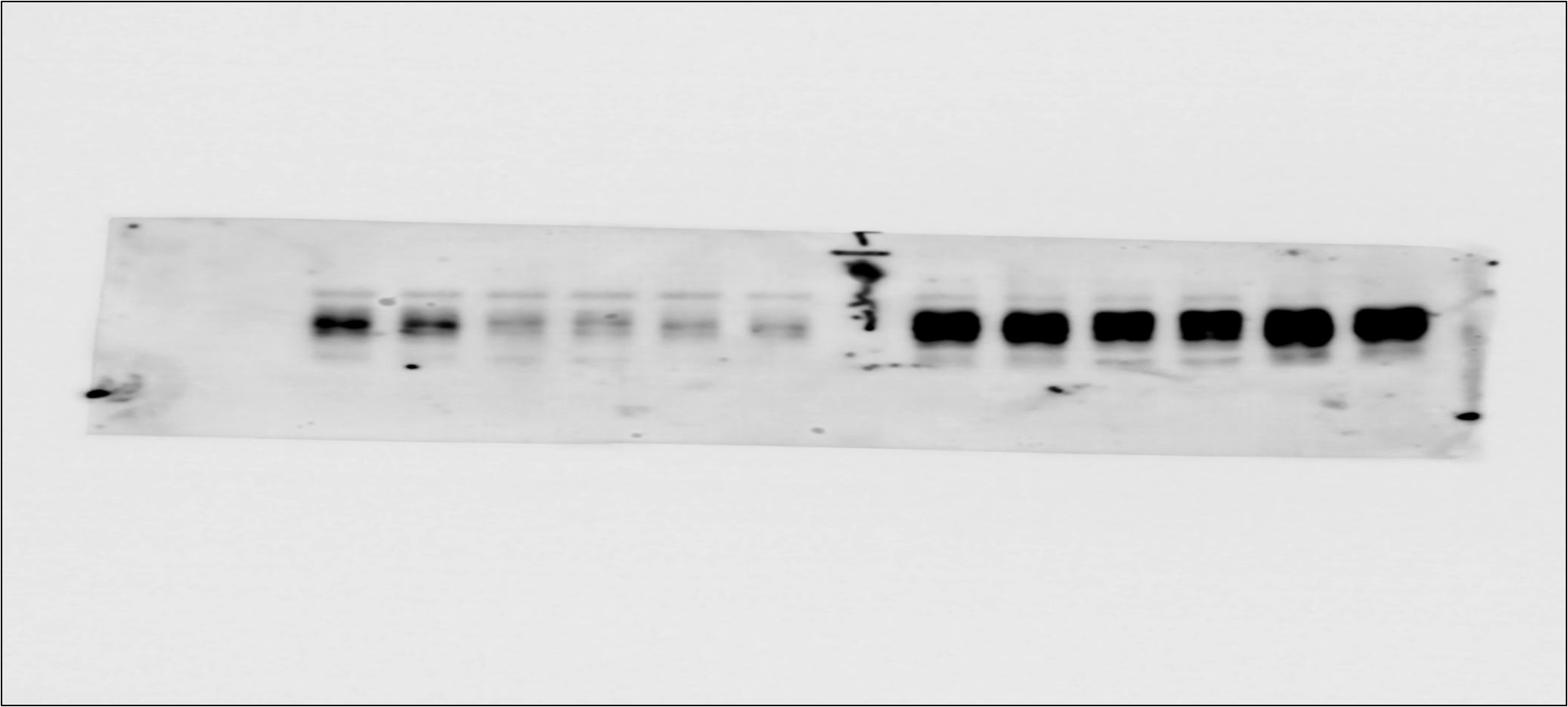

Supplement: Figure 6—figure supplement 1—source data 2. [file elife-108048-fig6-figsupp1-data2.zip › Figure 6-figure supplement 1/Figure S6 A-IP-Flag.tif]

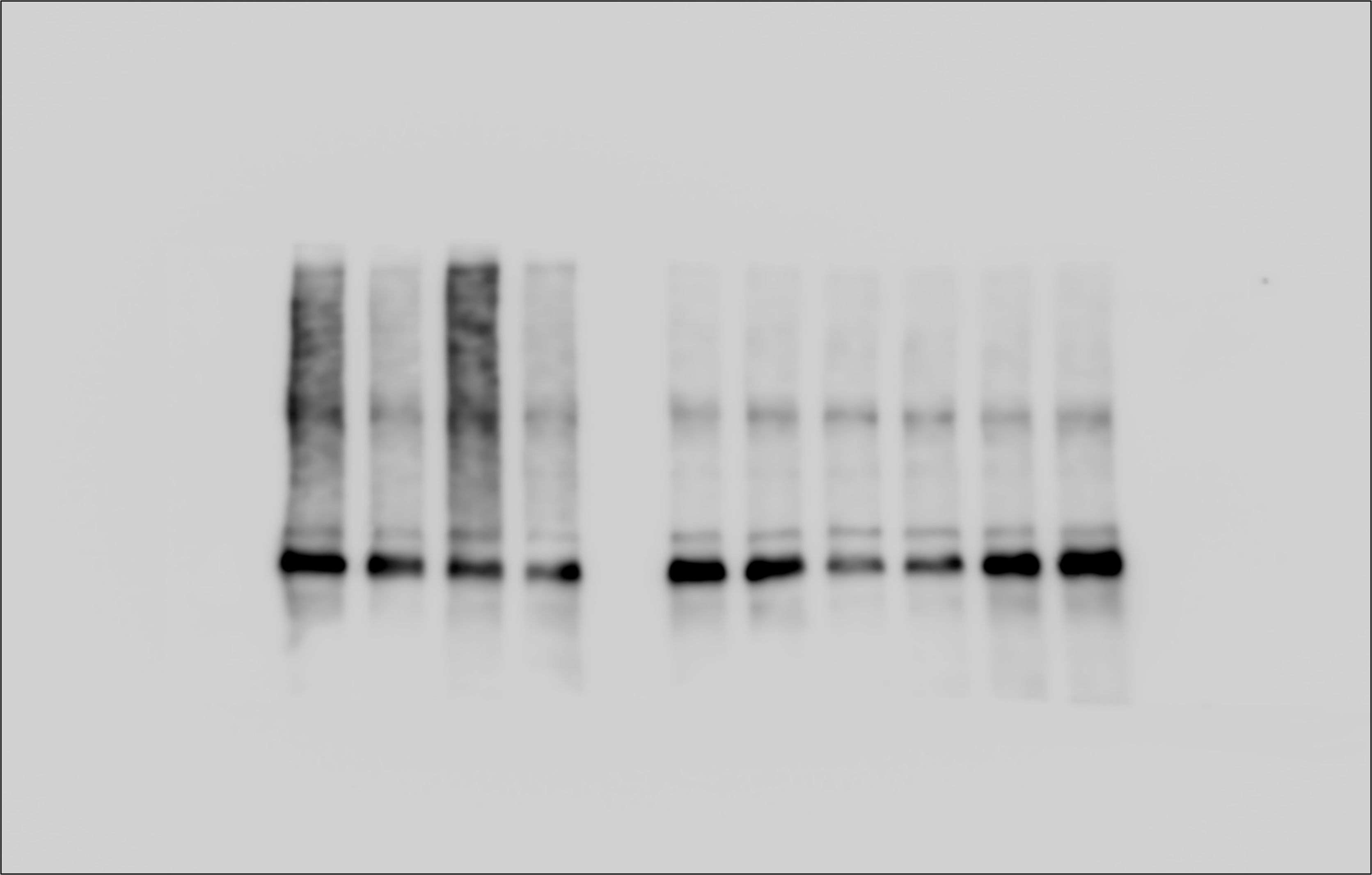

Supplement: Figure 6—figure supplement 1—source data 2. [file elife-108048-fig6-figsupp1-data2.zip › Figure 6-figure supplement 1/Figure S6 A-IP-HA.tif]

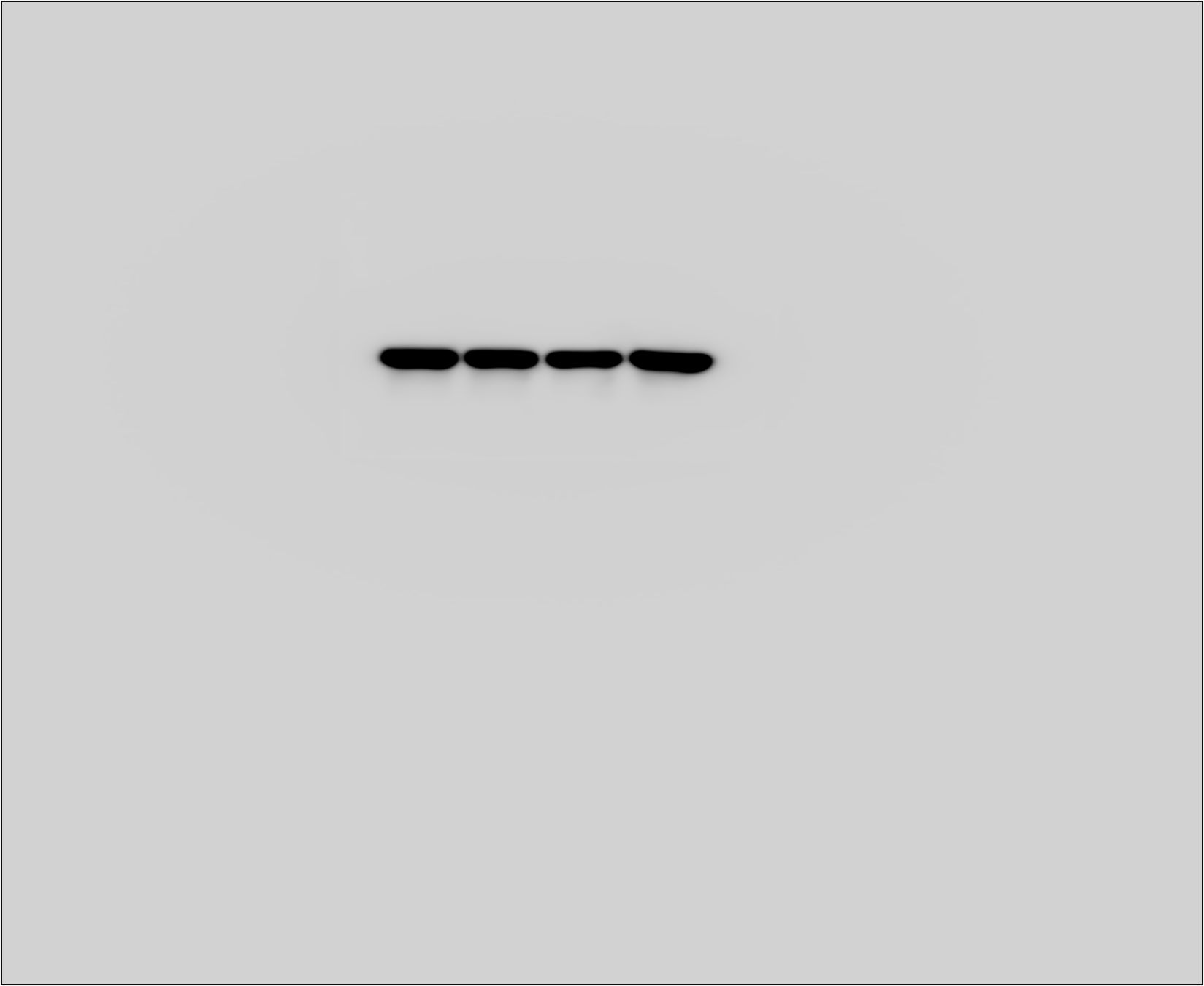

Supplement: Figure 6—figure supplement 1—source data 2. [file elife-108048-fig6-figsupp1-data2.zip › Figure 6-figure supplement 1/Figure S6 A-WCL-Actin.tif]

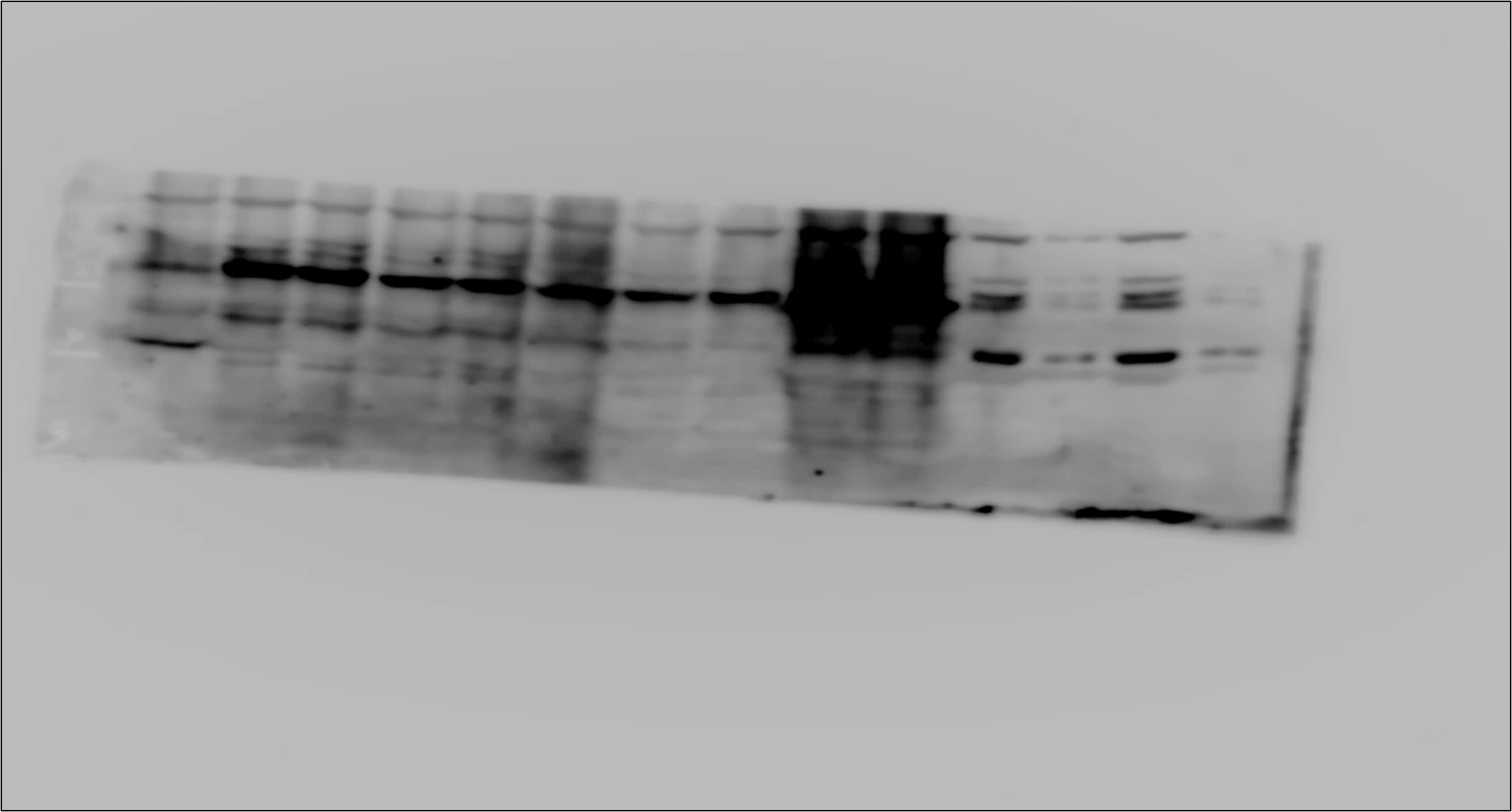

Supplement: Figure 6—figure supplement 1—source data 2. [file elife-108048-fig6-figsupp1-data2.zip › Figure 6-figure supplement 1/Figure S6 A-WCL-cyp17a2.tif]

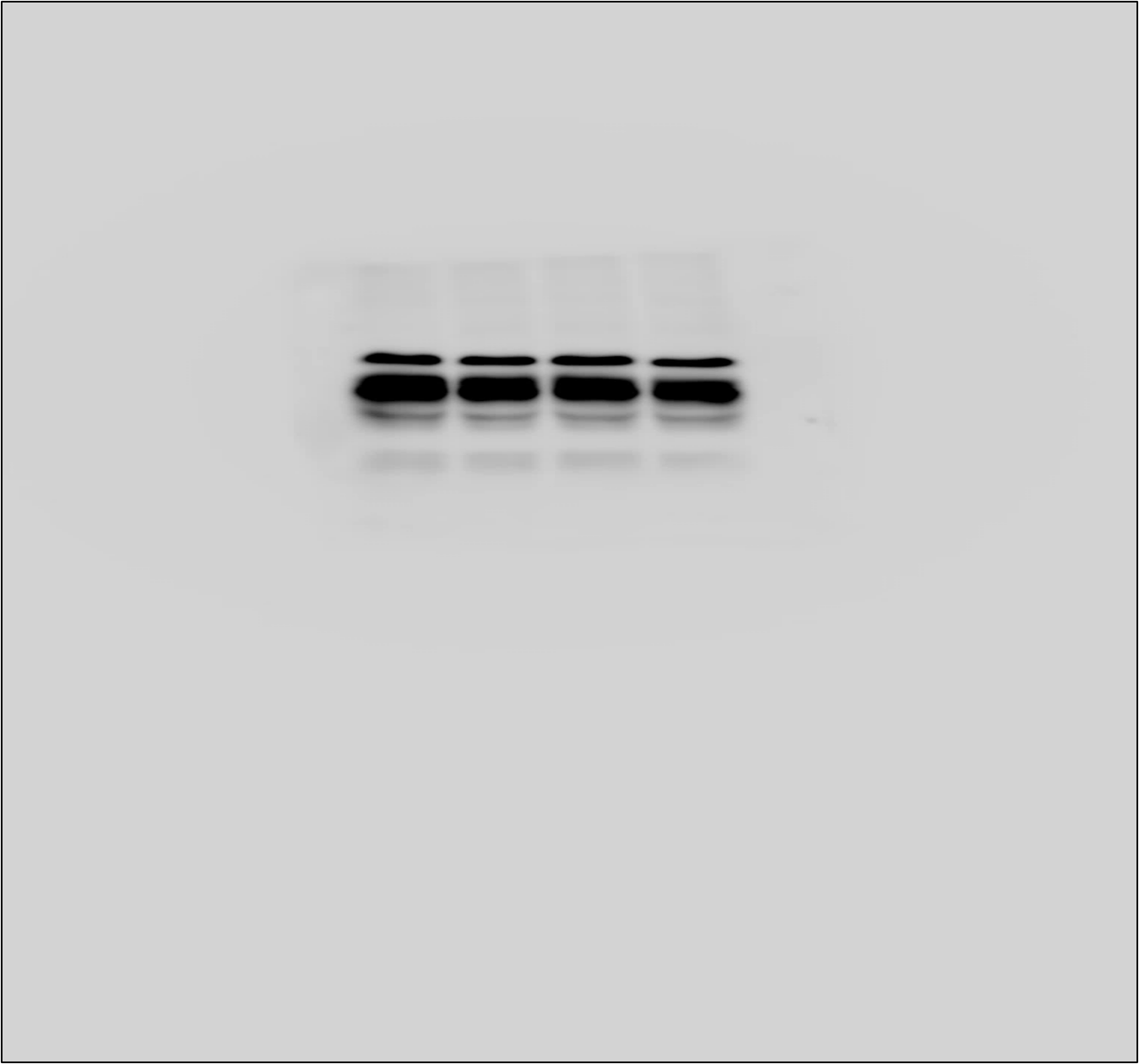

Supplement: Figure 6—figure supplement 1—source data 2. [file elife-108048-fig6-figsupp1-data2.zip › Figure 6-figure supplement 1/Figure S6 A-WCL-Flag.tif]

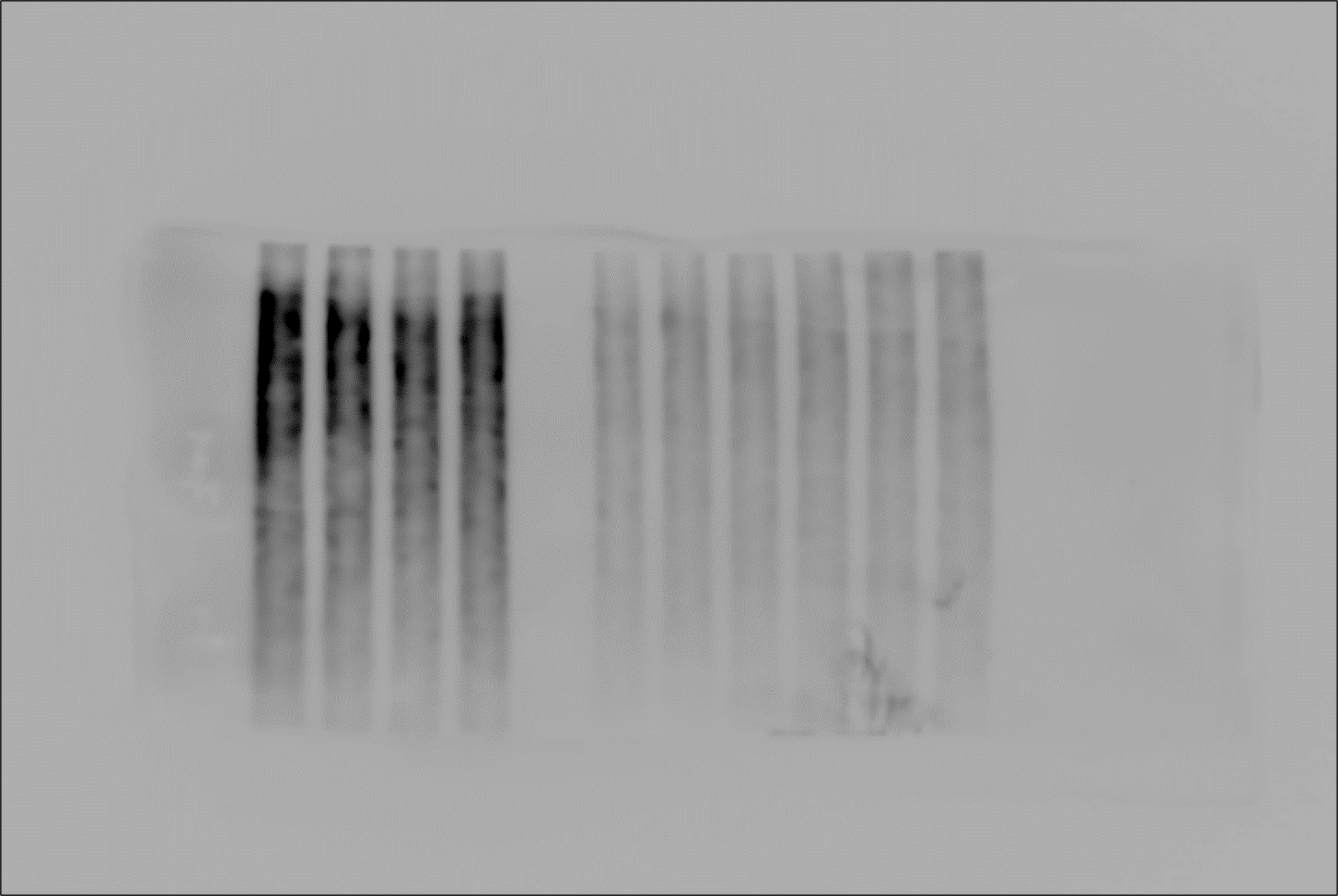

Supplement: Figure 6—figure supplement 1—source data 2. [file elife-108048-fig6-figsupp1-data2.zip › Figure 6-figure supplement 1/Figure S6 A-WCL-HA.tif]

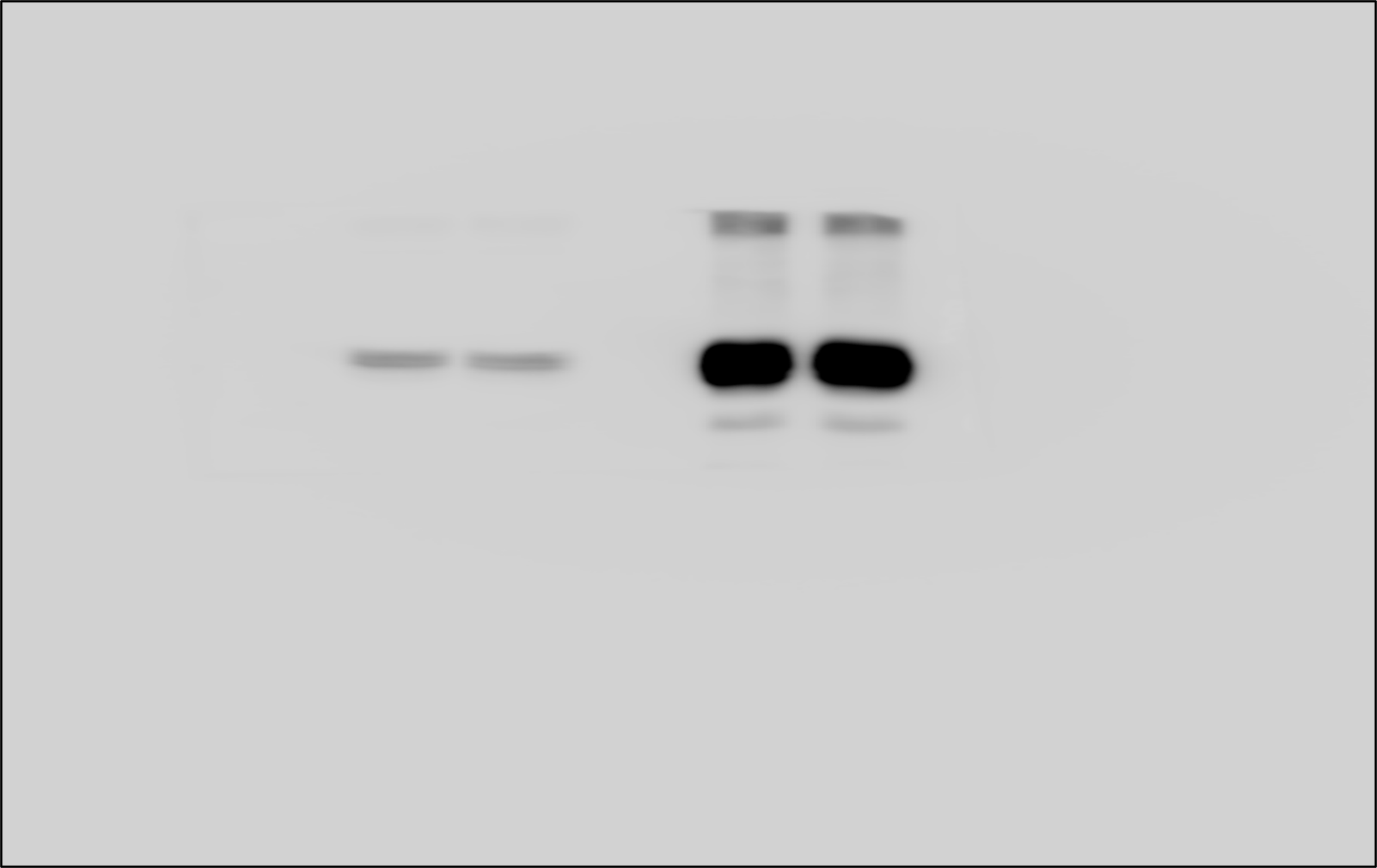

Supplement: Figure 6—figure supplement 1—source data 2. [file elife-108048-fig6-figsupp1-data2.zip › Figure 6-figure supplement 1/Figure S6 B-IP-Flag.tif]

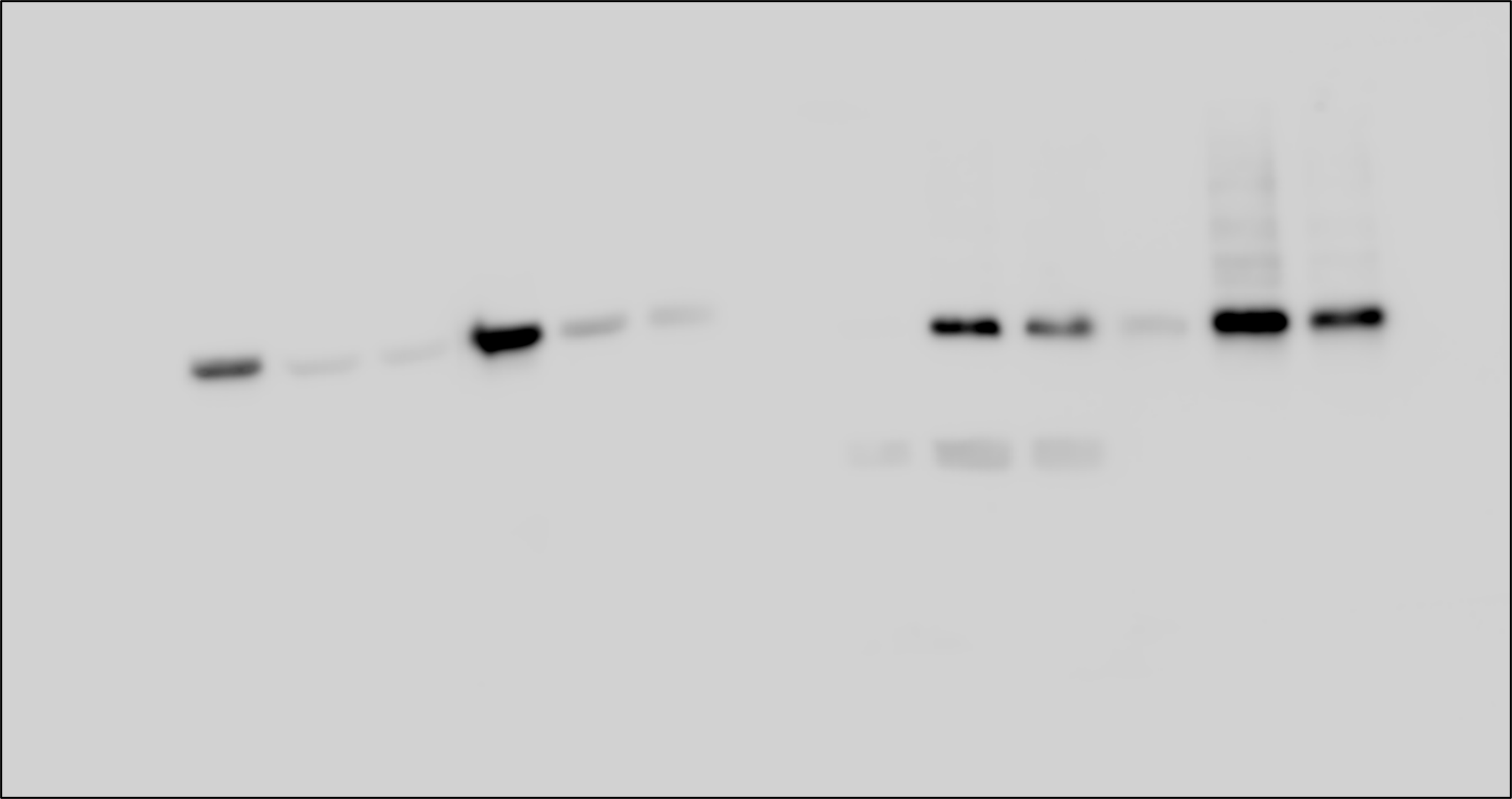

Supplement: Figure 6—figure supplement 1—source data 2. [file elife-108048-fig6-figsupp1-data2.zip › Figure 6-figure supplement 1/Figure S6 B-IP-Myc.tif]

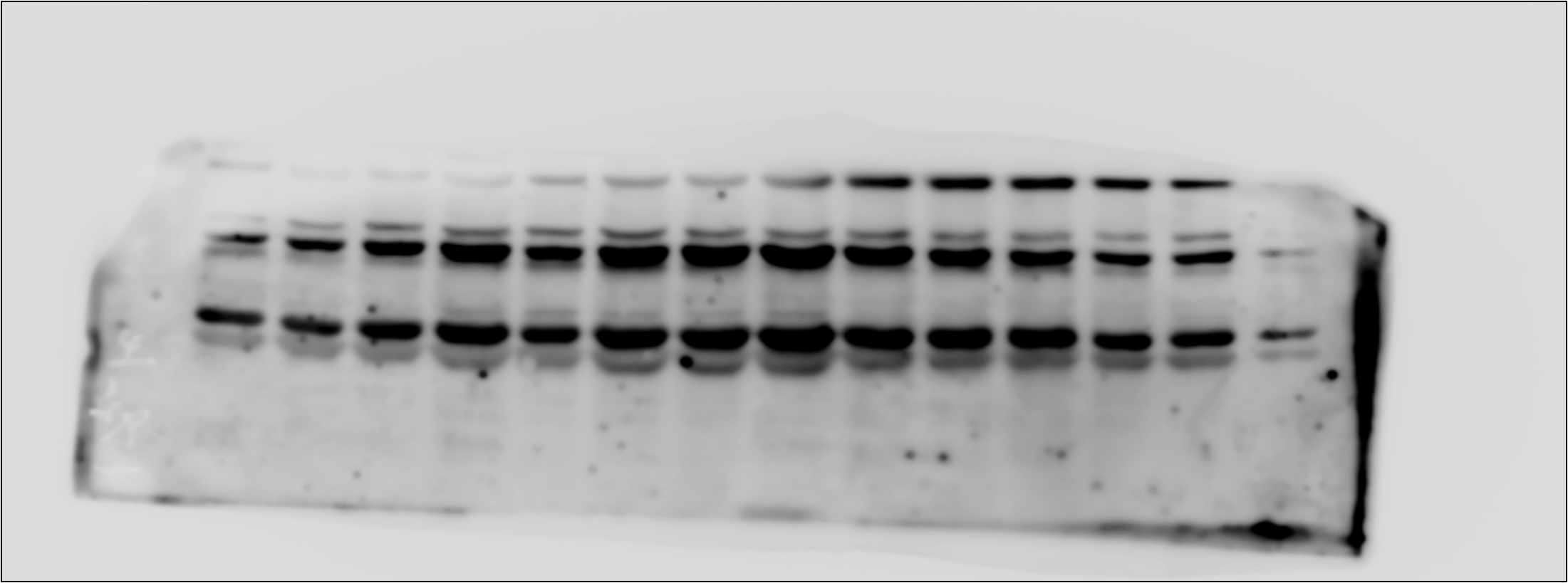

Supplement: Figure 6—figure supplement 1—source data 2. [file elife-108048-fig6-figsupp1-data2.zip › Figure 6-figure supplement 1/Figure S6 B-WCL-cyp17a2.tif]

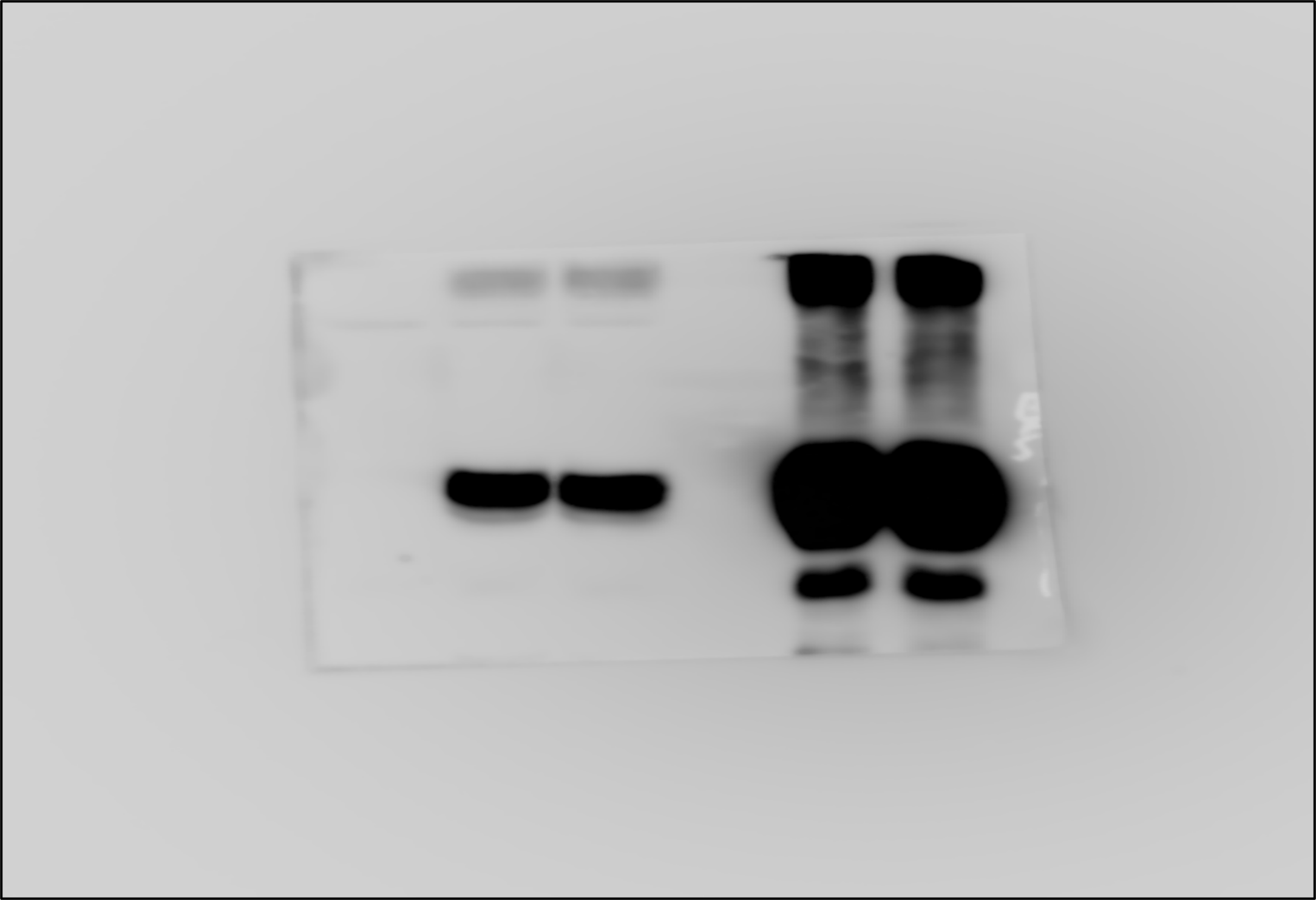

Supplement: Figure 6—figure supplement 1—source data 2. [file elife-108048-fig6-figsupp1-data2.zip › Figure 6-figure supplement 1/Figure S6 B-WCL-Flag.tif]

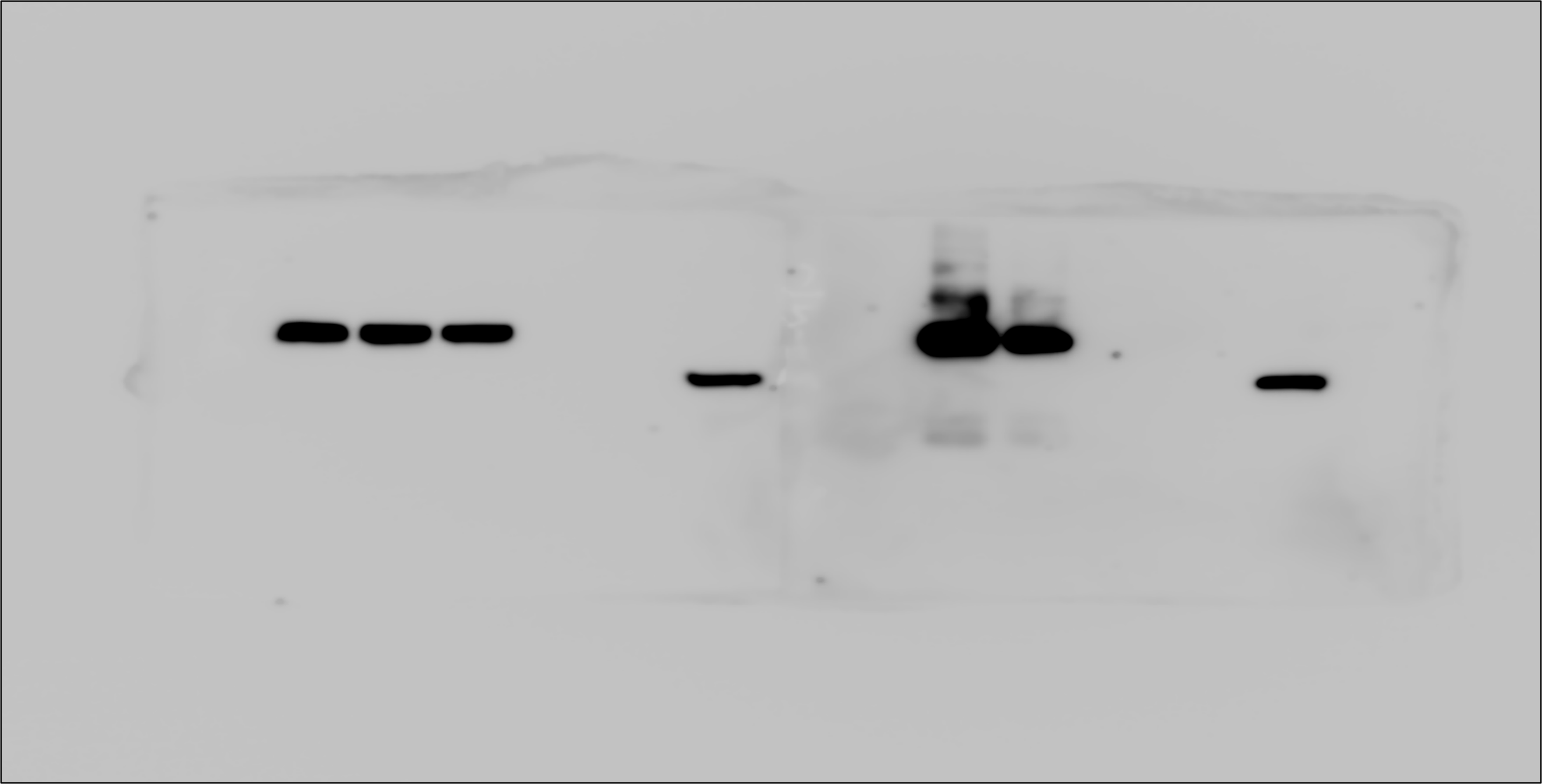

Supplement: Figure 6—figure supplement 1—source data 2. [file elife-108048-fig6-figsupp1-data2.zip › Figure 6-figure supplement 1/Figure S6 B-WCL-Myc.tif]

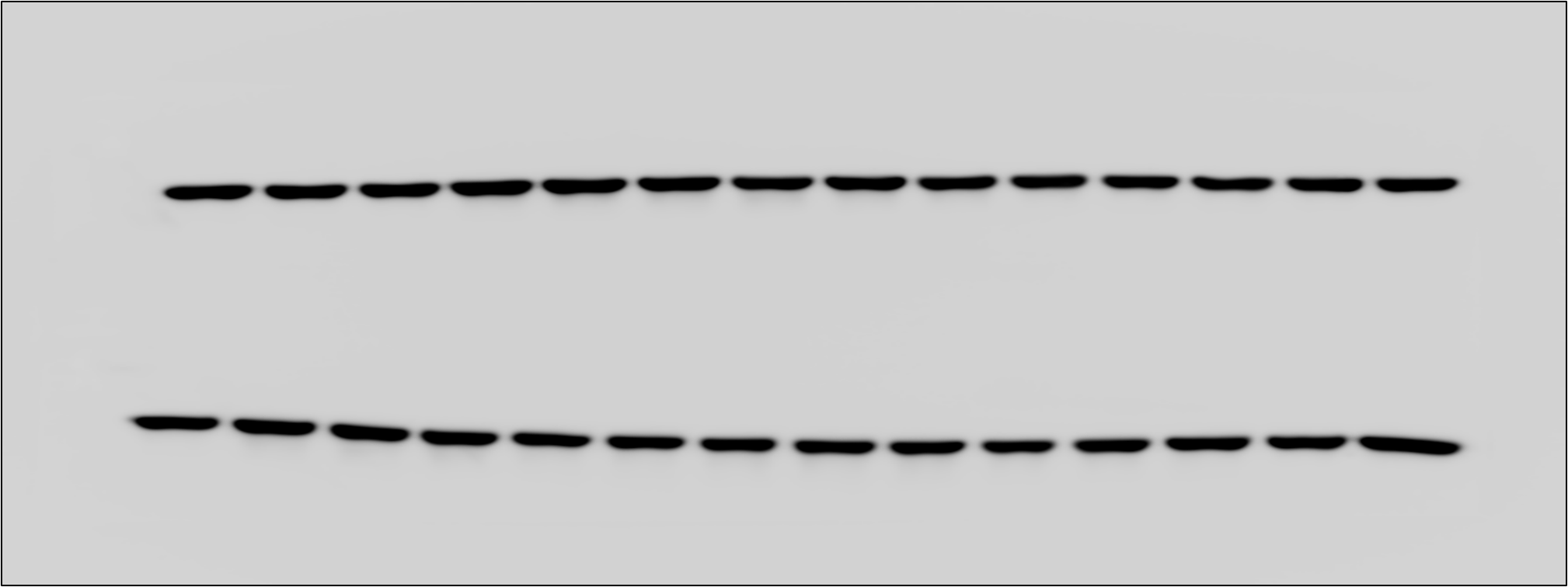

Supplement: Figure 6—figure supplement 1—source data 2. [file elife-108048-fig6-figsupp1-data2.zip › Figure 6-figure supplement 1/Figure S6 C-Actin.tif]

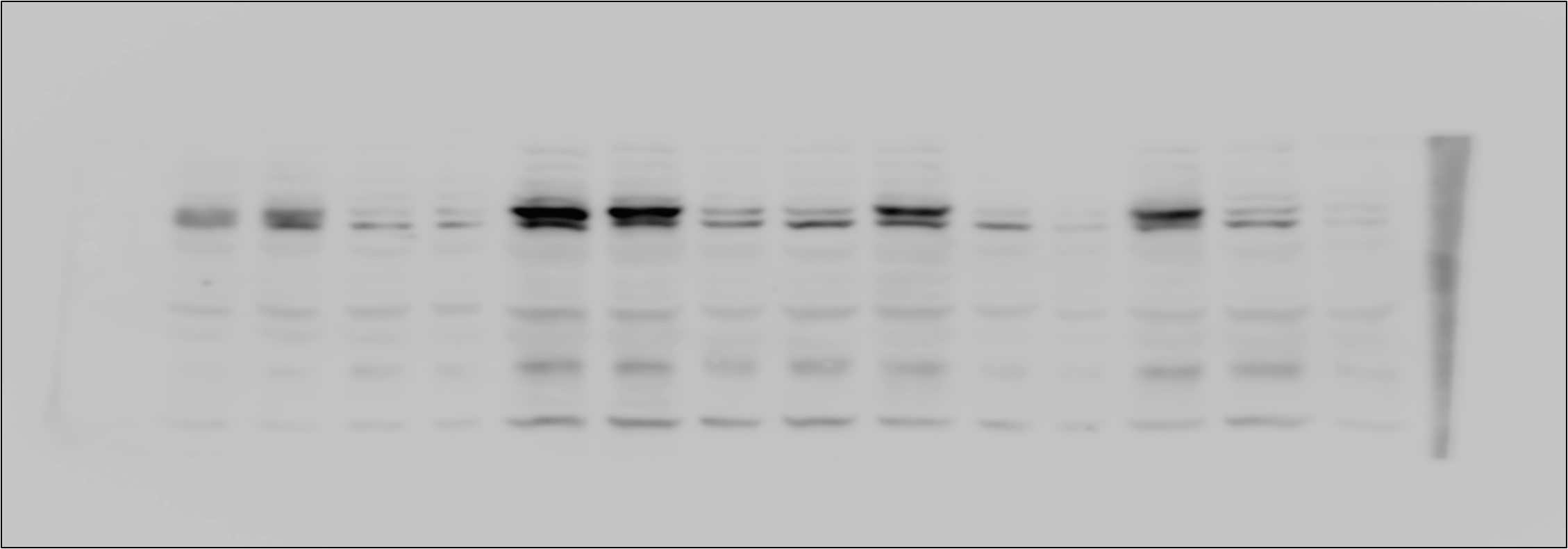

Supplement: Figure 6—figure supplement 1—source data 2. [file elife-108048-fig6-figsupp1-data2.zip › Figure 6-figure supplement 1/Figure S6 C-btr32.tif]

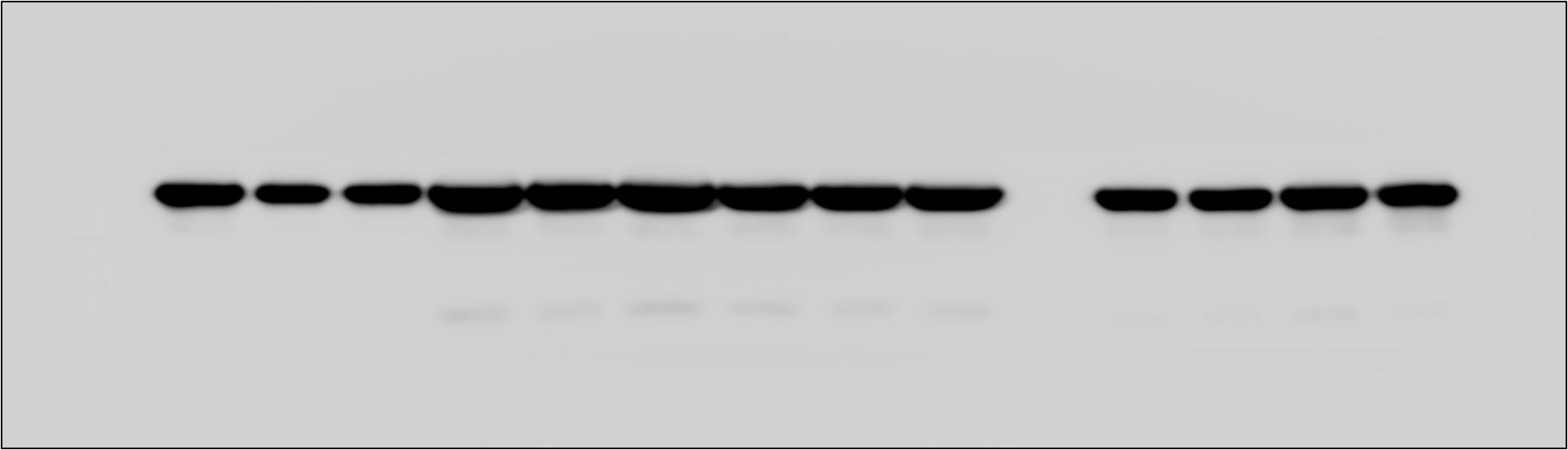

Supplement: Figure 6—figure supplement 1—source data 2. [file elife-108048-fig6-figsupp1-data2.zip › Figure 6-figure supplement 1/Figure S6 D-Actin.tif]

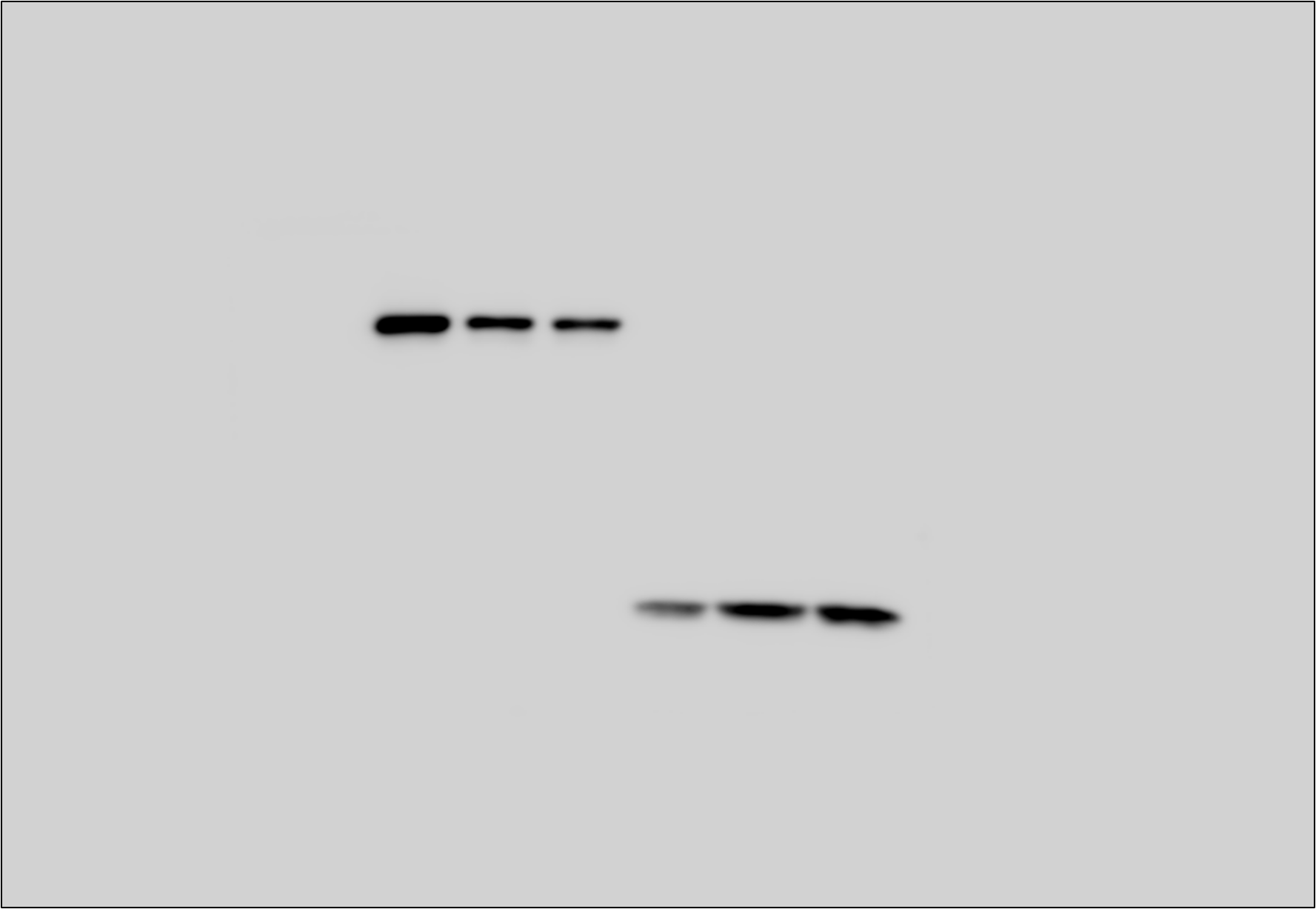

Supplement: Figure 6—figure supplement 1—source data 2. [file elife-108048-fig6-figsupp1-data2.zip › Figure 6-figure supplement 1/Figure S6 D-Myc.tif]

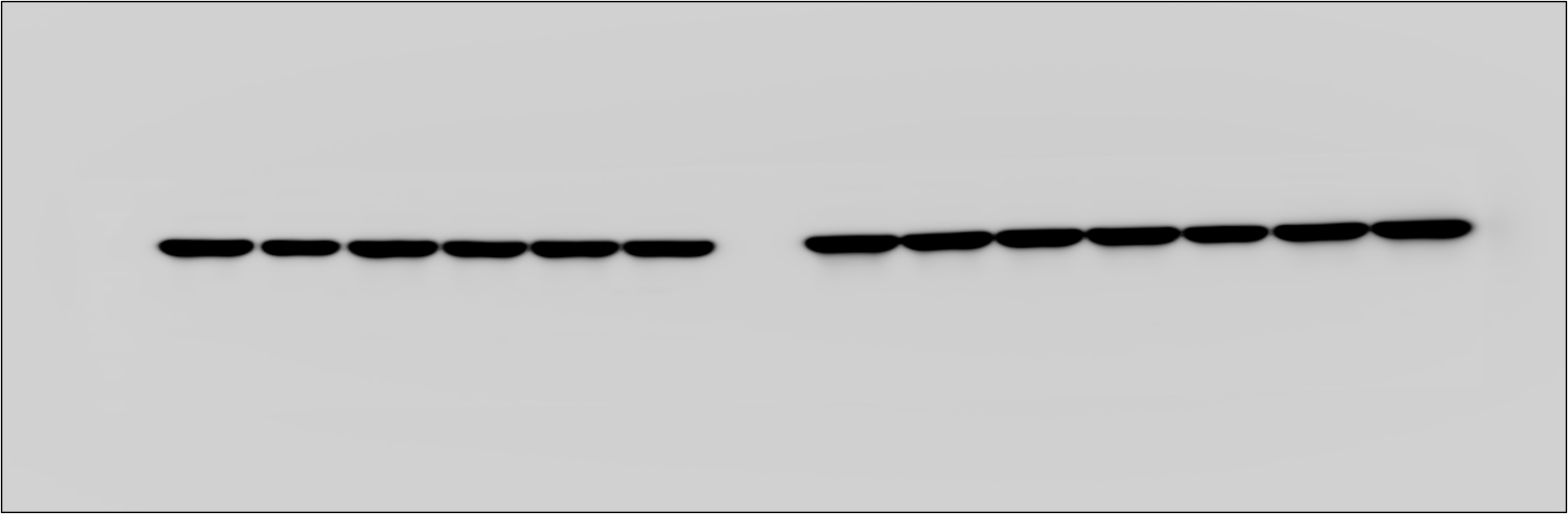

Supplement: Figure 6—figure supplement 1—source data 2. [file elife-108048-fig6-figsupp1-data2.zip › Figure 6-figure supplement 1/Figure S6 G-Actin-2.tif]

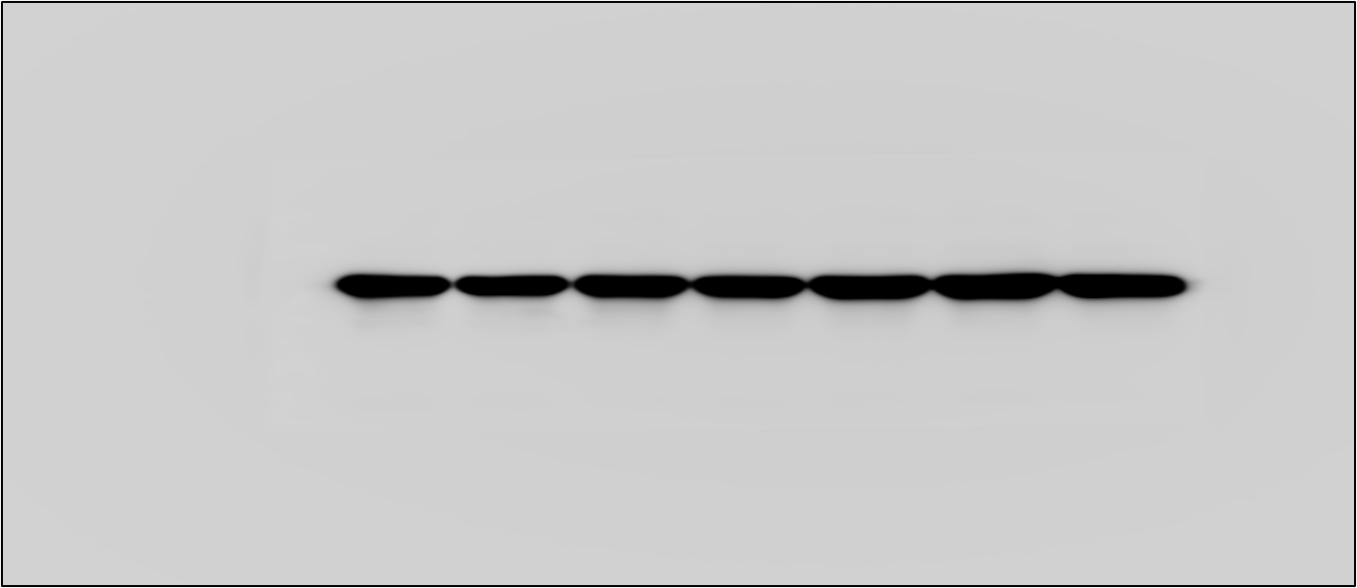

Supplement: Figure 6—figure supplement 1—source data 2. [file elife-108048-fig6-figsupp1-data2.zip › Figure 6-figure supplement 1/Figure S6 G-Actin.tif]

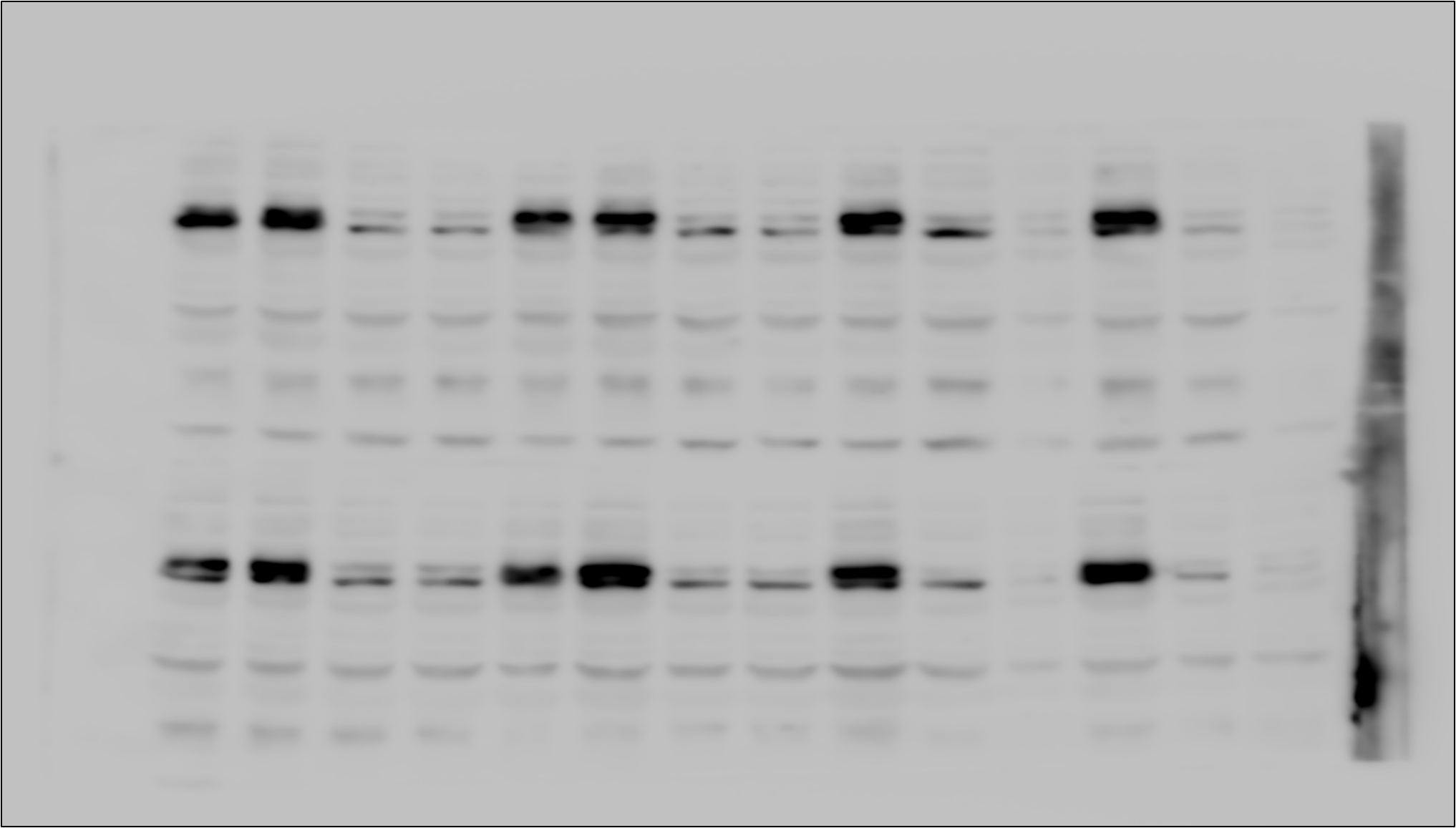

Supplement: Figure 6—figure supplement 1—source data 2. [file elife-108048-fig6-figsupp1-data2.zip › Figure 6-figure supplement 1/Figure S6 G-btr32-2.tif]

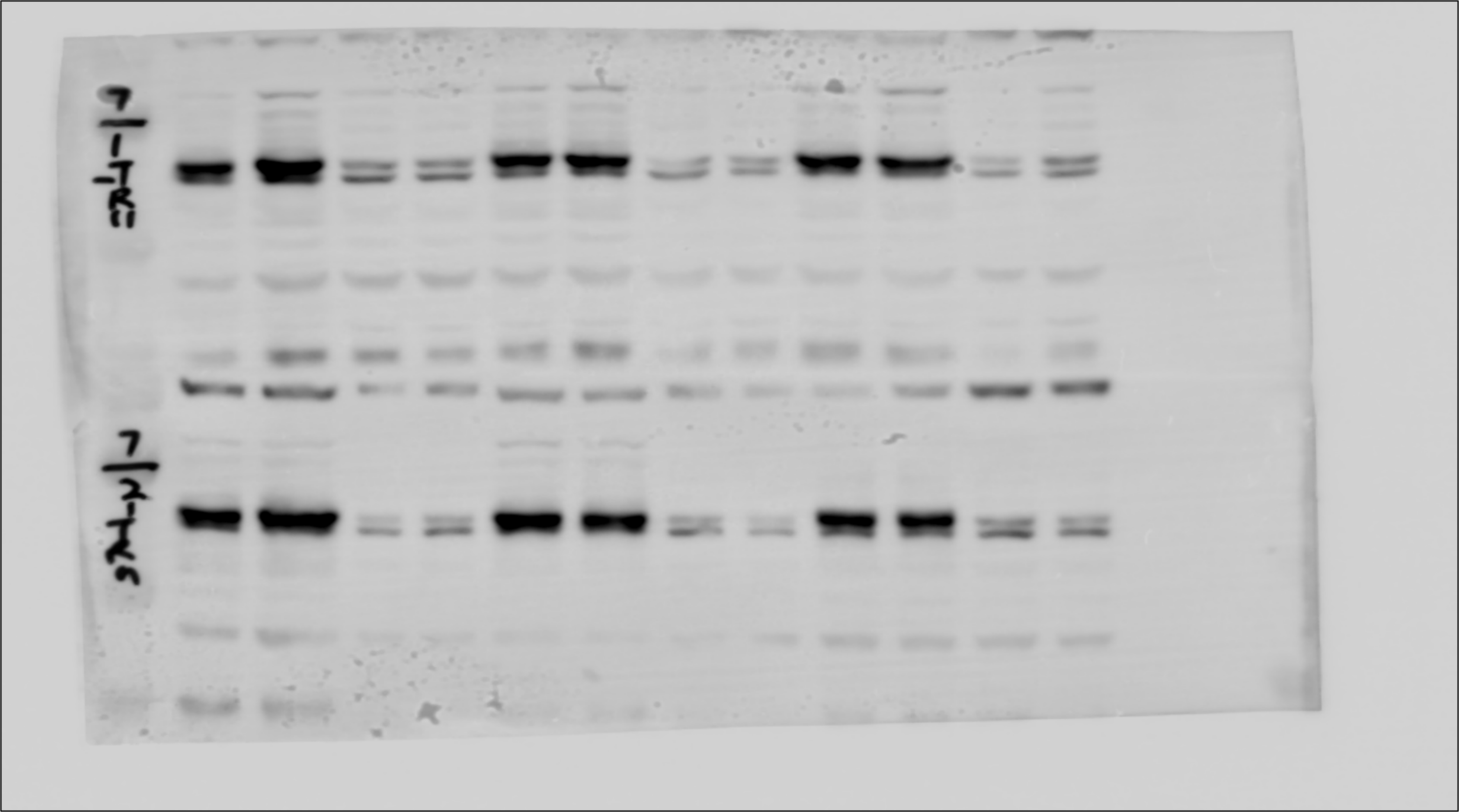

Supplement: Figure 6—figure supplement 1—source data 2. [file elife-108048-fig6-figsupp1-data2.zip › Figure 6-figure supplement 1/Figure S6 G-btr32.tif]

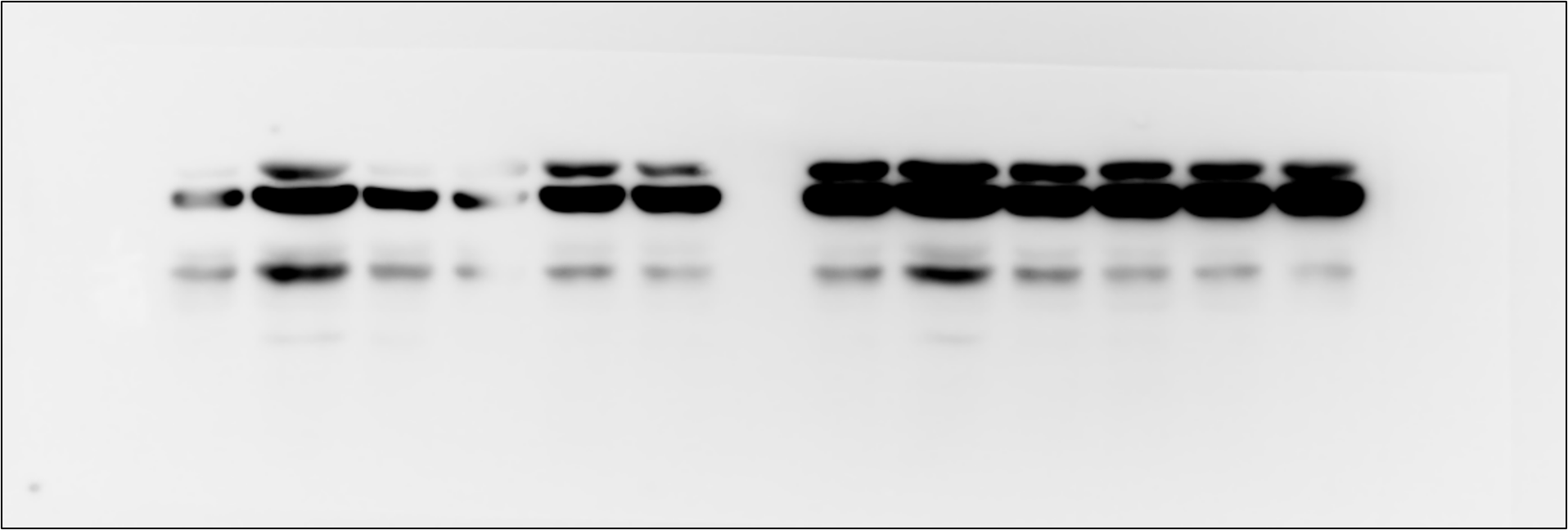

Supplement: Figure 6—figure supplement 1—source data 2. [file elife-108048-fig6-figsupp1-data2.zip › Figure 6-figure supplement 1/Figure S6 G-Flag.tif]

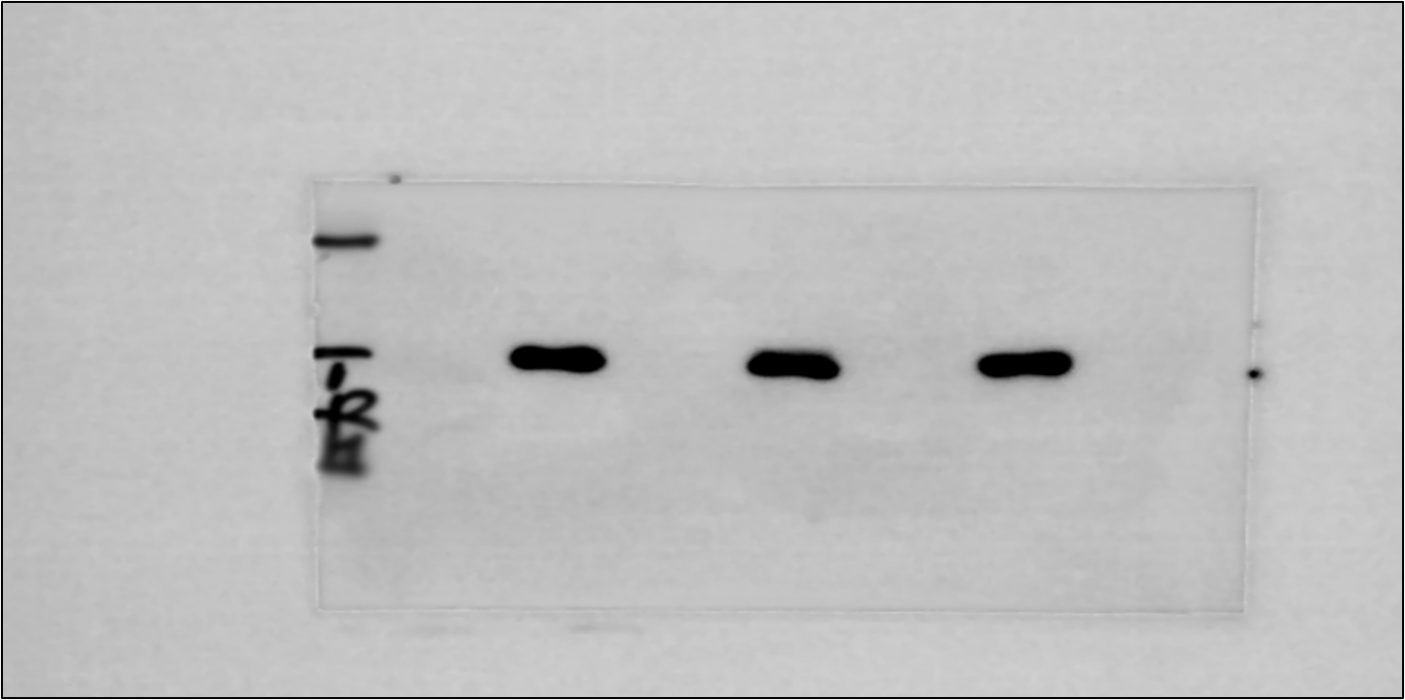

Supplement: Figure 6—figure supplement 1—source data 2. [file elife-108048-fig6-figsupp1-data2.zip › Figure 6-figure supplement 1/Figure S6 G-HA-2.tif]

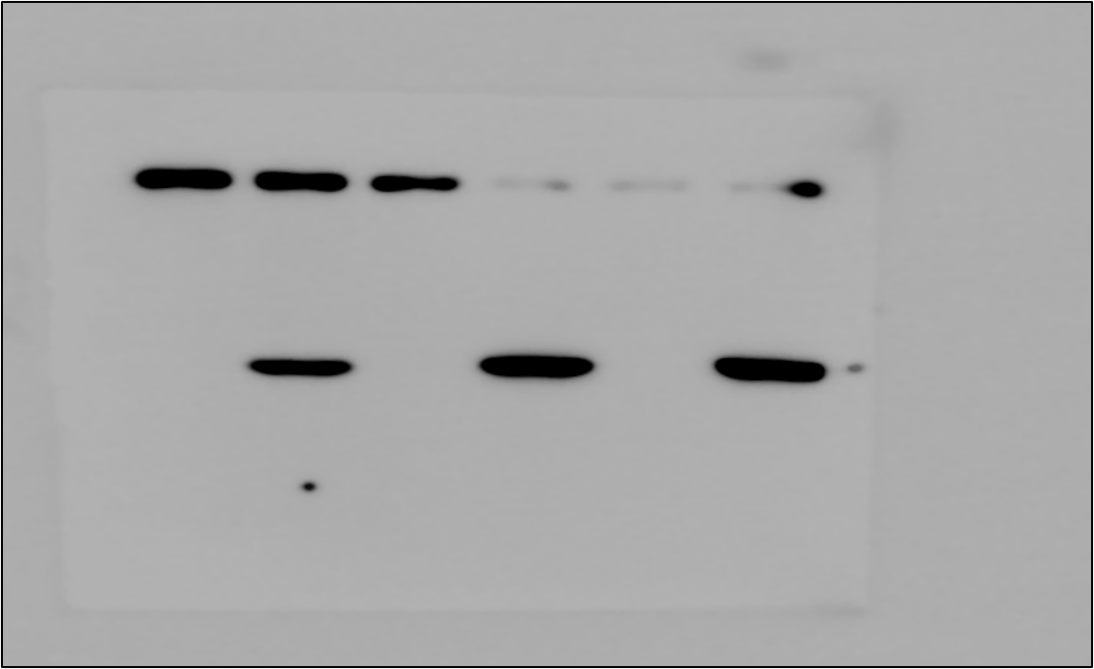

Supplement: Figure 6—figure supplement 1—source data 2. [file elife-108048-fig6-figsupp1-data2.zip › Figure 6-figure supplement 1/Figure S6 G-HA.tif]

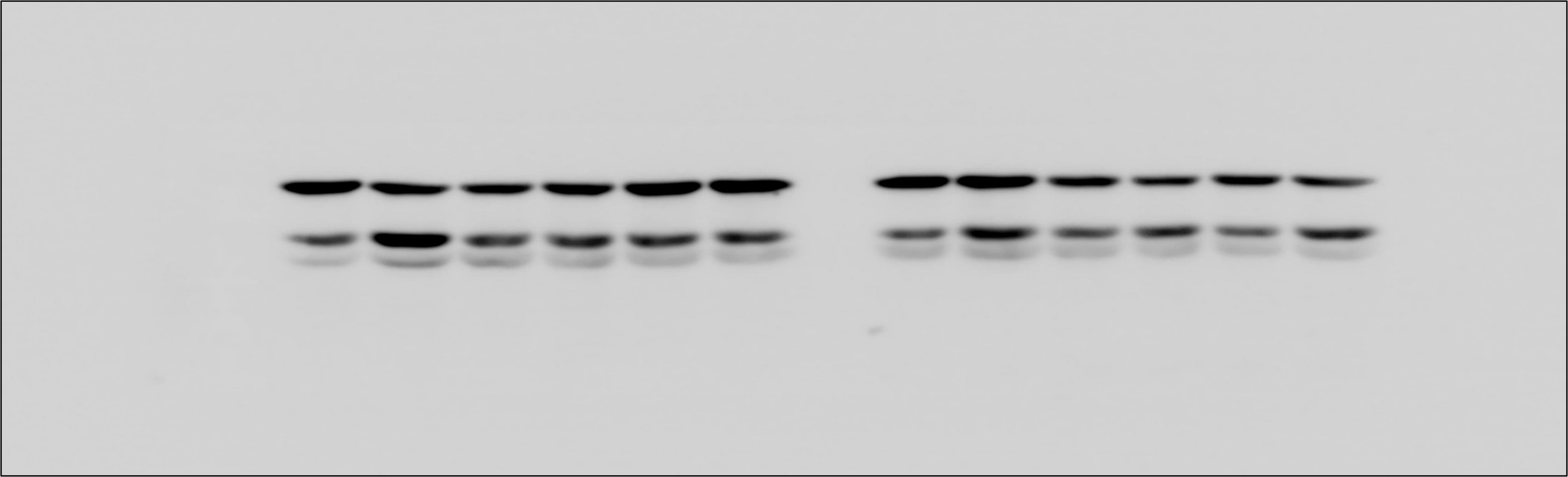

Supplement: Figure 6—figure supplement 1—source data 2. [file elife-108048-fig6-figsupp1-data2.zip › Figure 6-figure supplement 1/Figure S6 G-STING.tif]

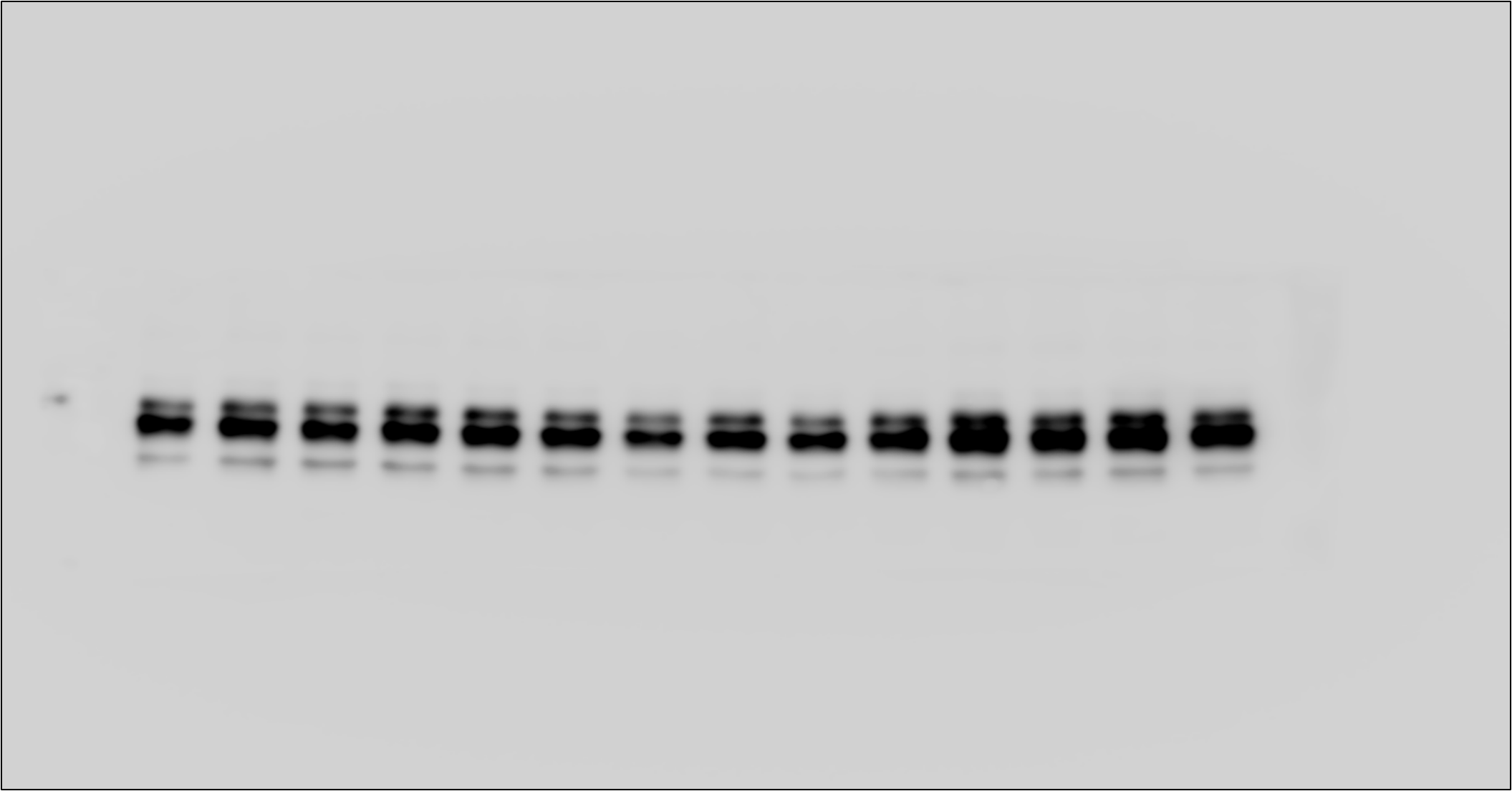

Supplement: Figure 7—source data 2. [file elife-108048-fig7-data2.zip › Figure 7/Figure 7 A-IP-Flag.tif]

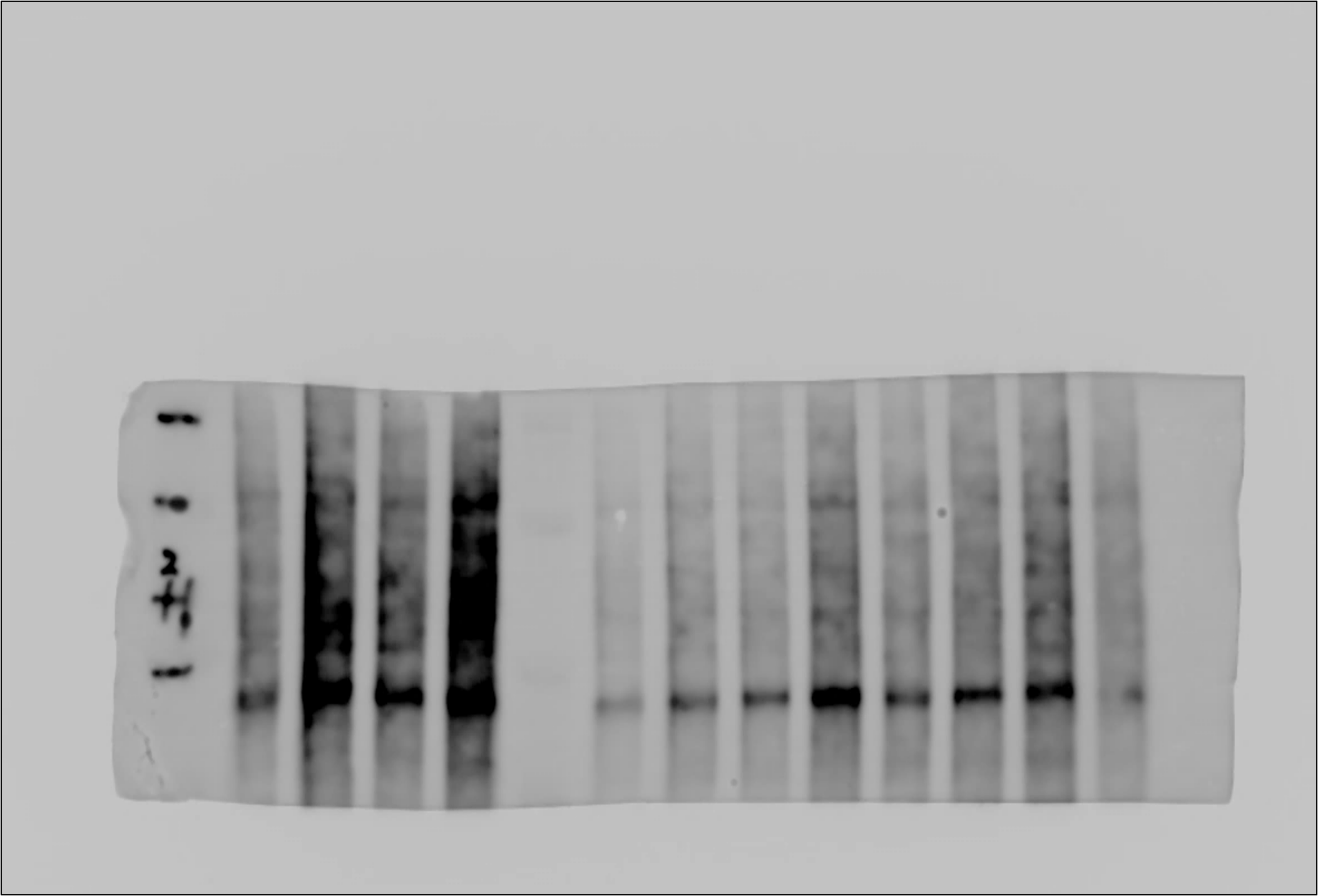

Supplement: Figure 7—source data 2. [file elife-108048-fig7-data2.zip › Figure 7/Figure 7 A-IP-HA.tif]

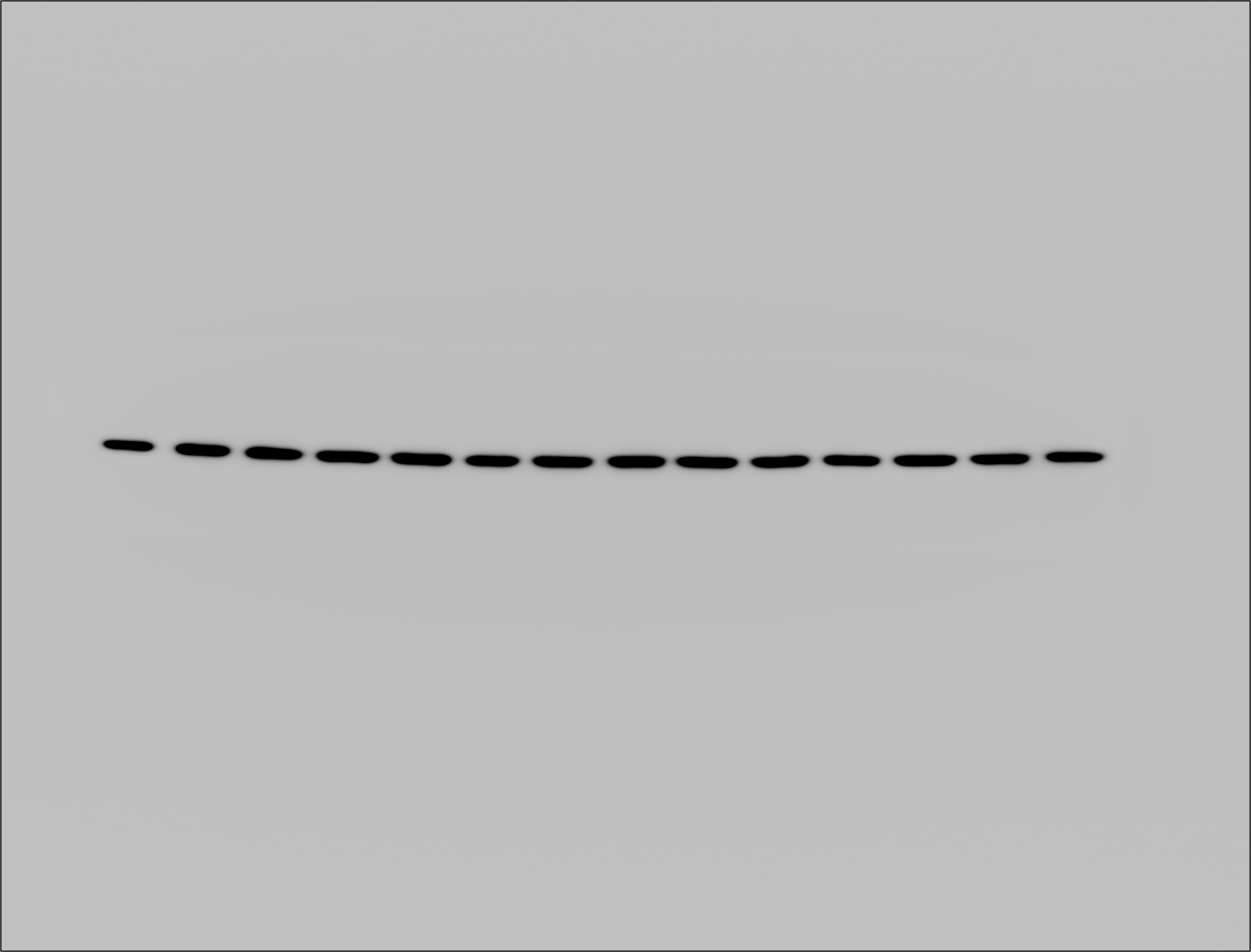

Supplement: Figure 7—source data 2. [file elife-108048-fig7-data2.zip › Figure 7/Figure 7 A-WCL-Actin.tif]

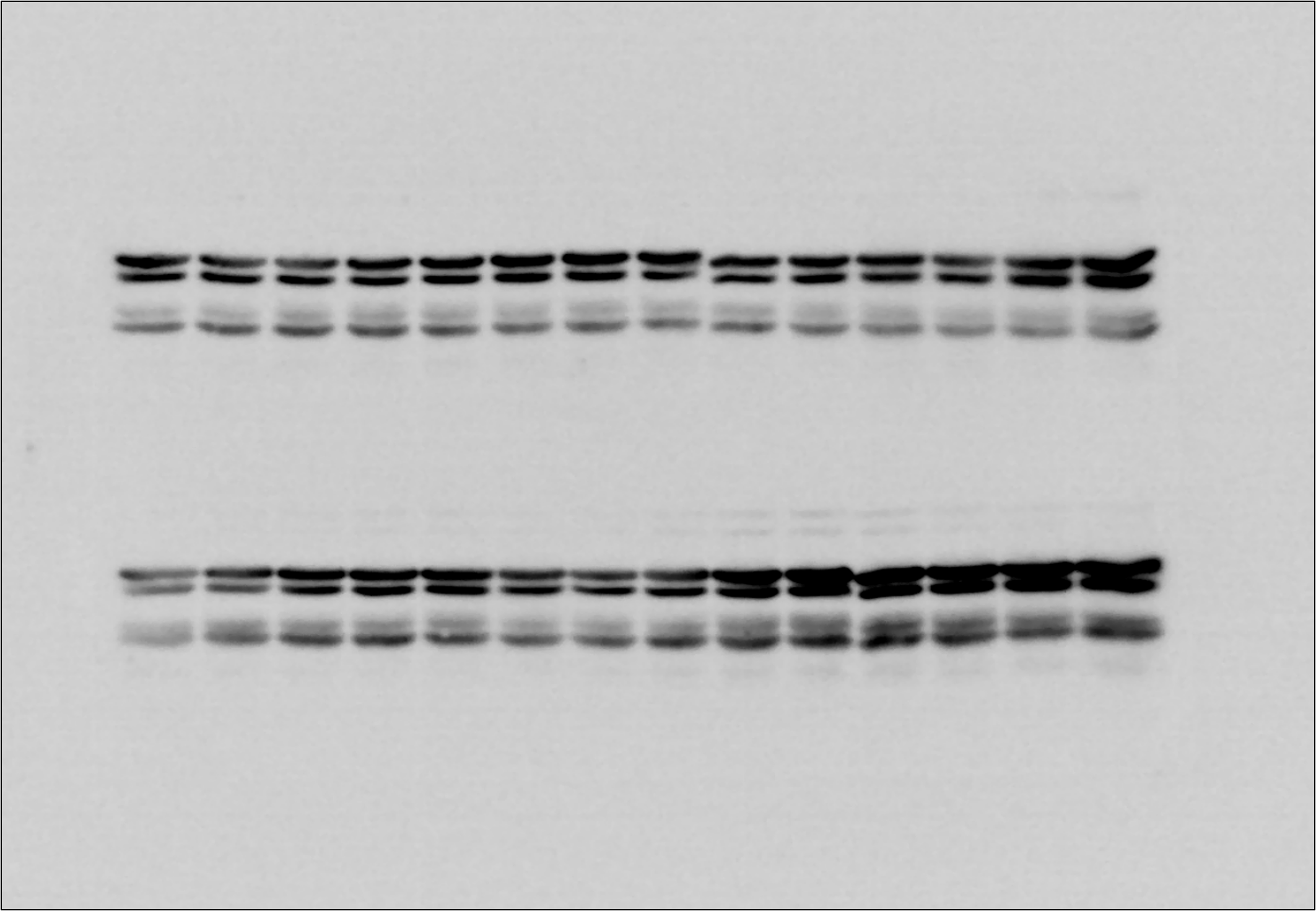

Supplement: Figure 7—source data 2. [file elife-108048-fig7-data2.zip › Figure 7/Figure 7 A-WCL-Flag.tif]

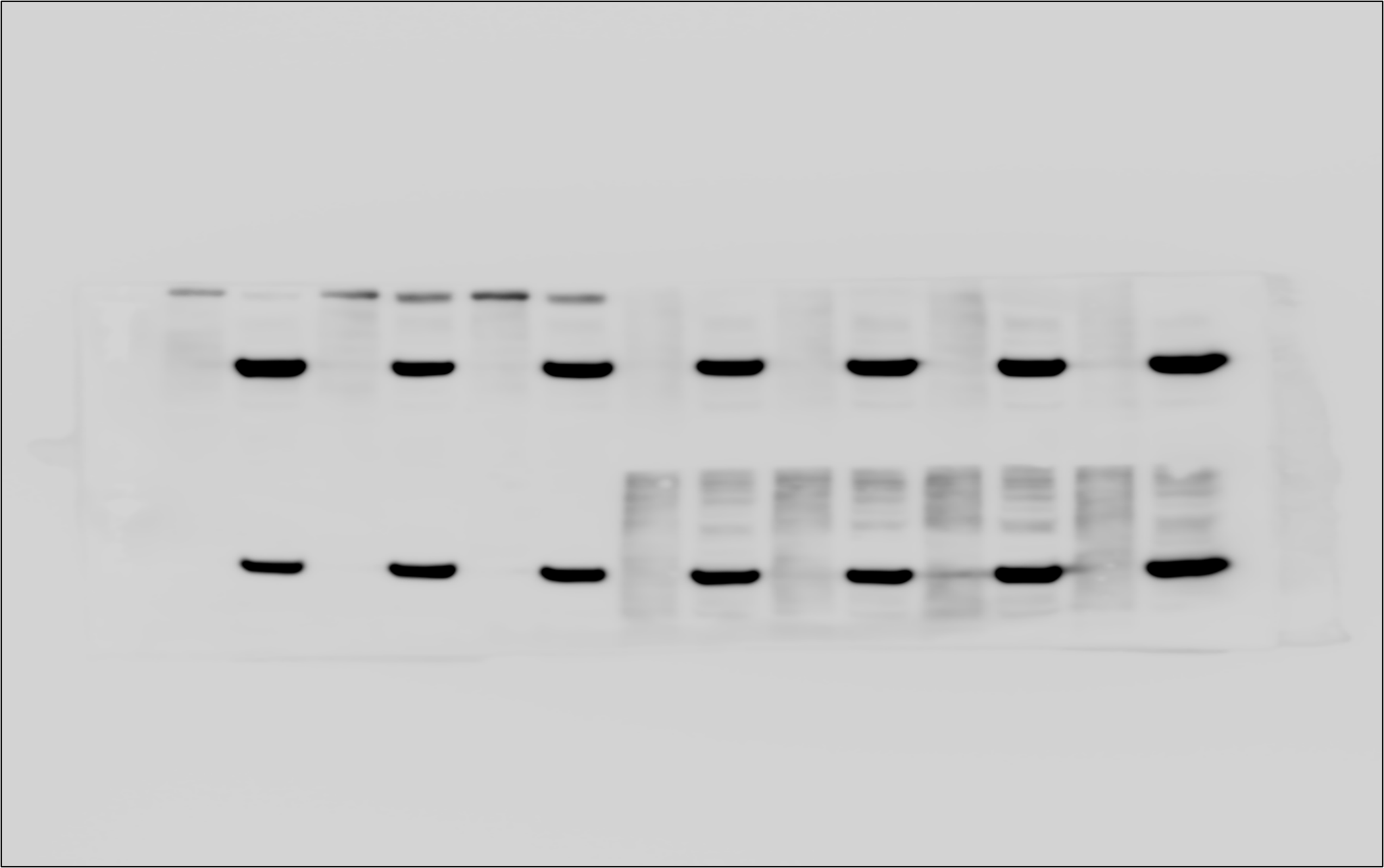

Supplement: Figure 7—source data 2. [file elife-108048-fig7-data2.zip › Figure 7/Figure 7 A-WCL-HA-cyp17a2.tif]

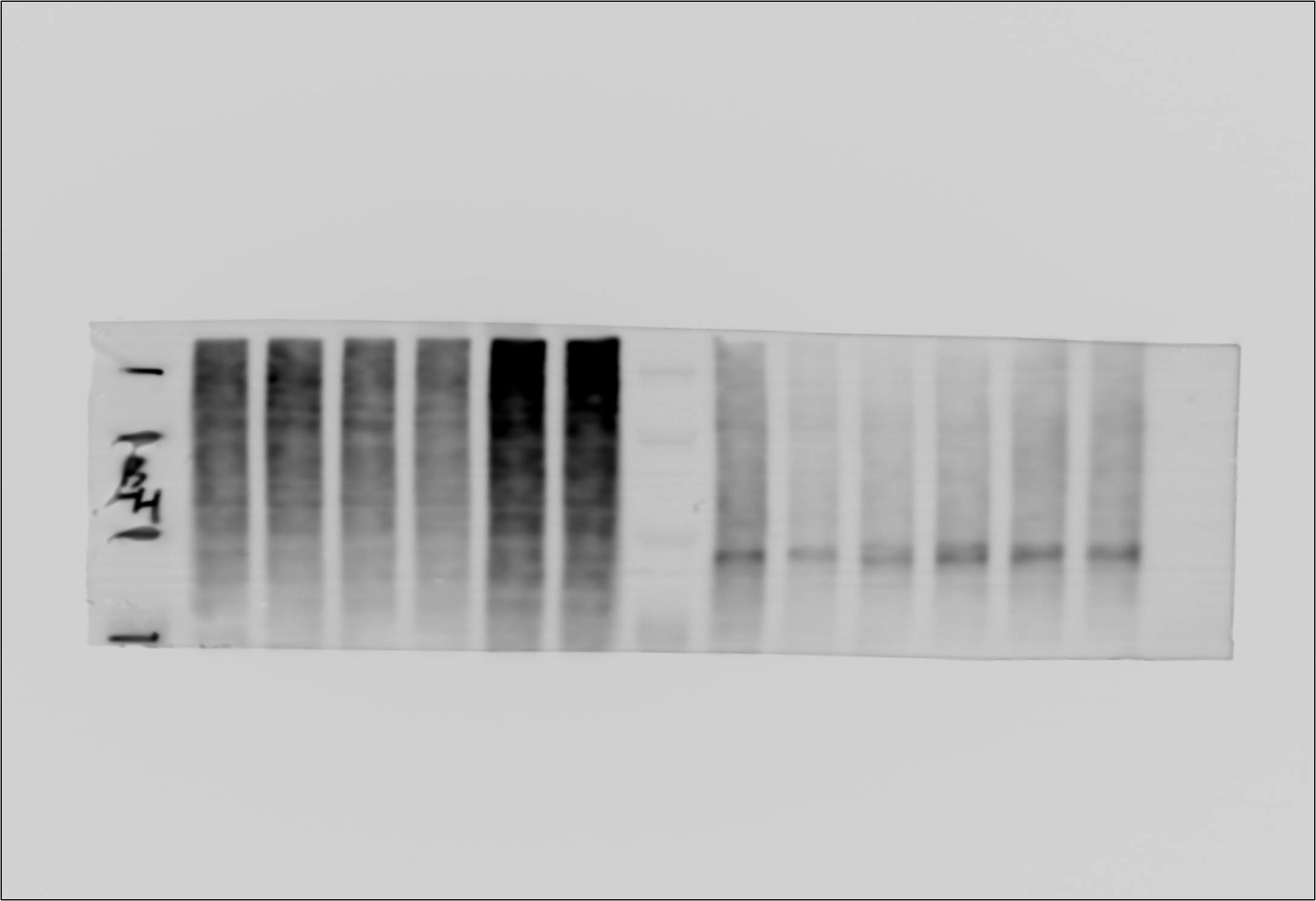

Supplement: Figure 7—source data 2. [file elife-108048-fig7-data2.zip › Figure 7/Figure 7 A-WCL-HA.tif]

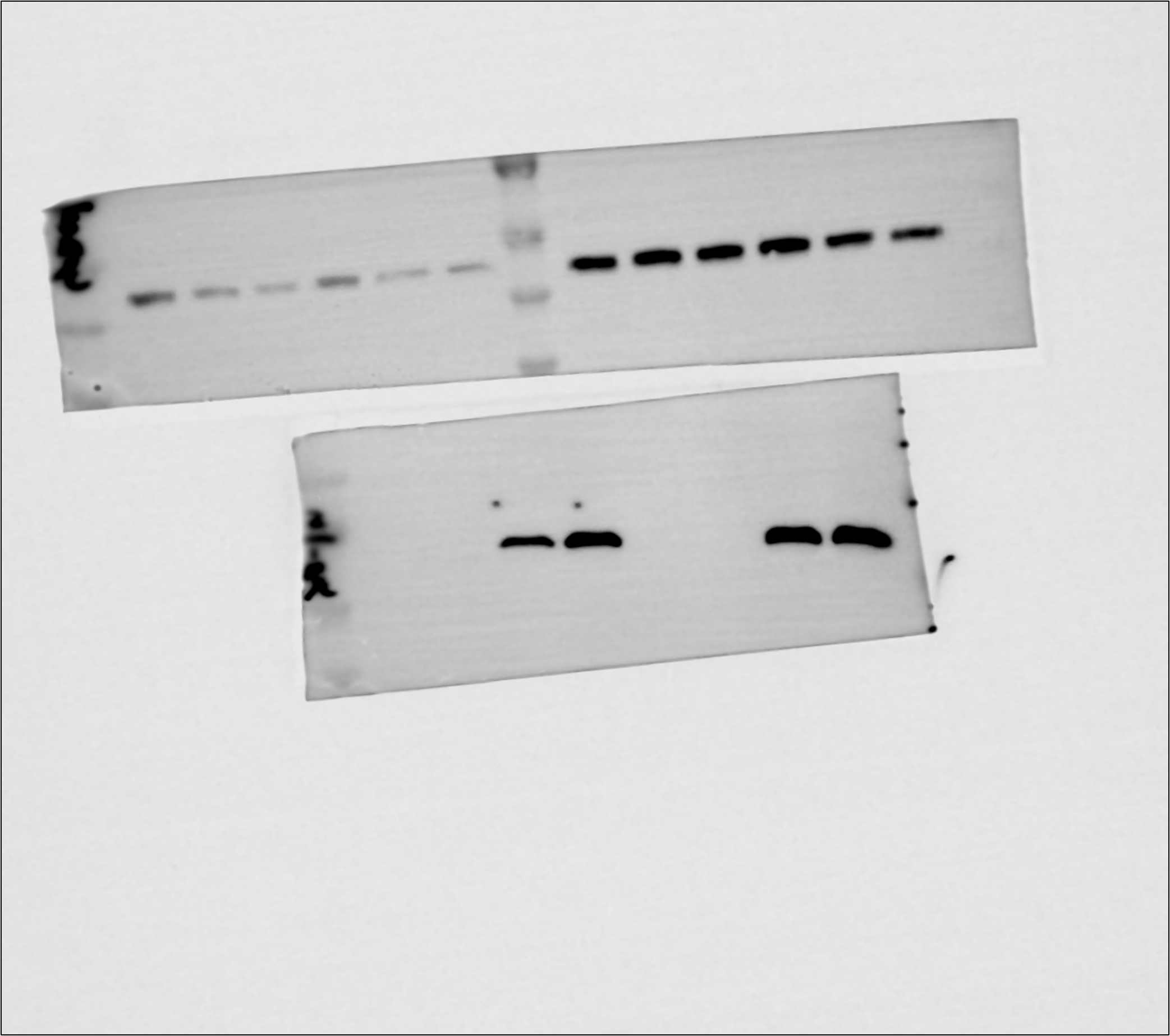

Supplement: Figure 7—source data 2. [file elife-108048-fig7-data2.zip › Figure 7/Figure 7 A-WCL-Myc.tif]

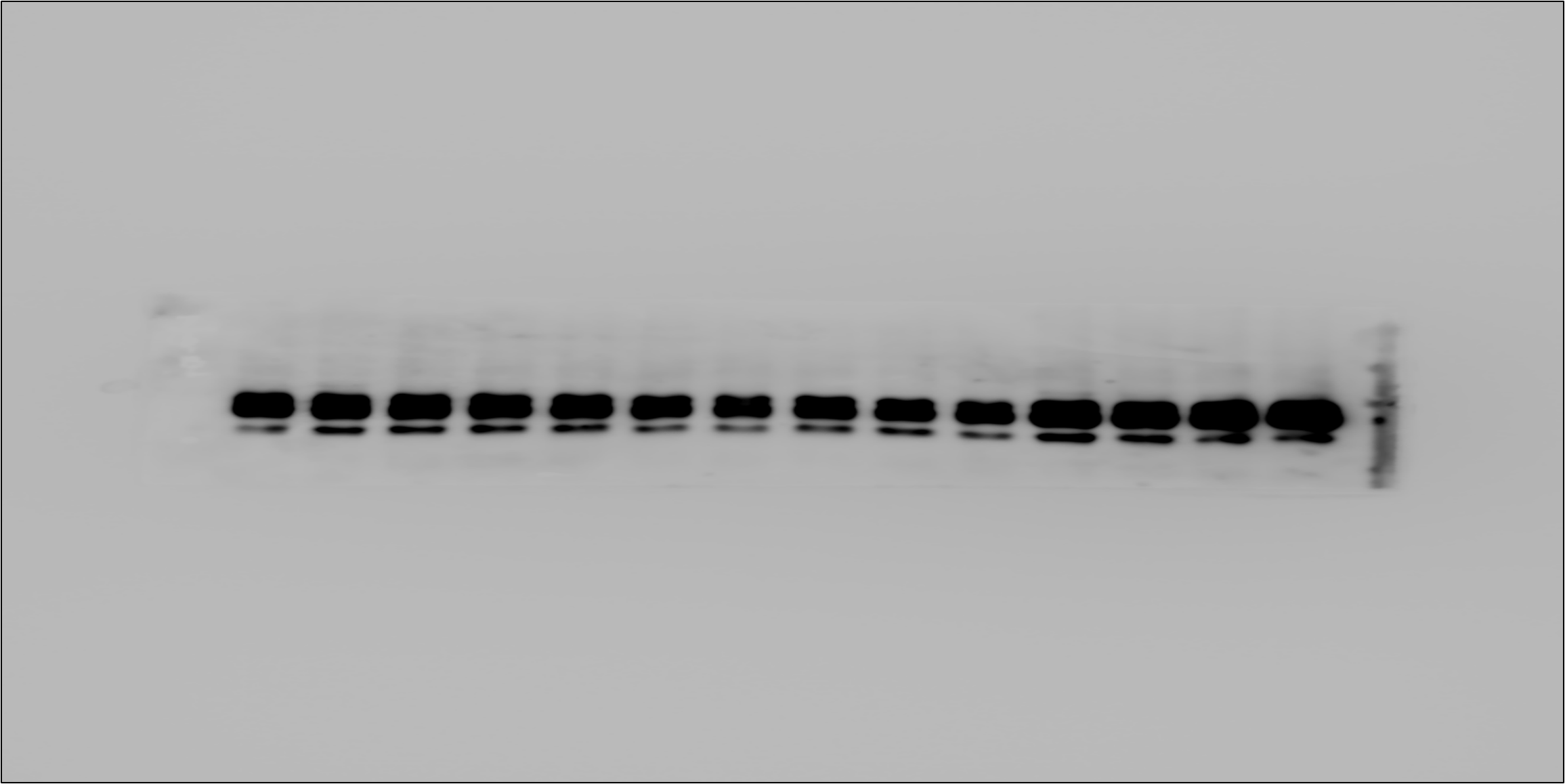

Supplement: Figure 7—source data 2. [file elife-108048-fig7-data2.zip › Figure 7/Figure 7 B-IP-Flag.tif]

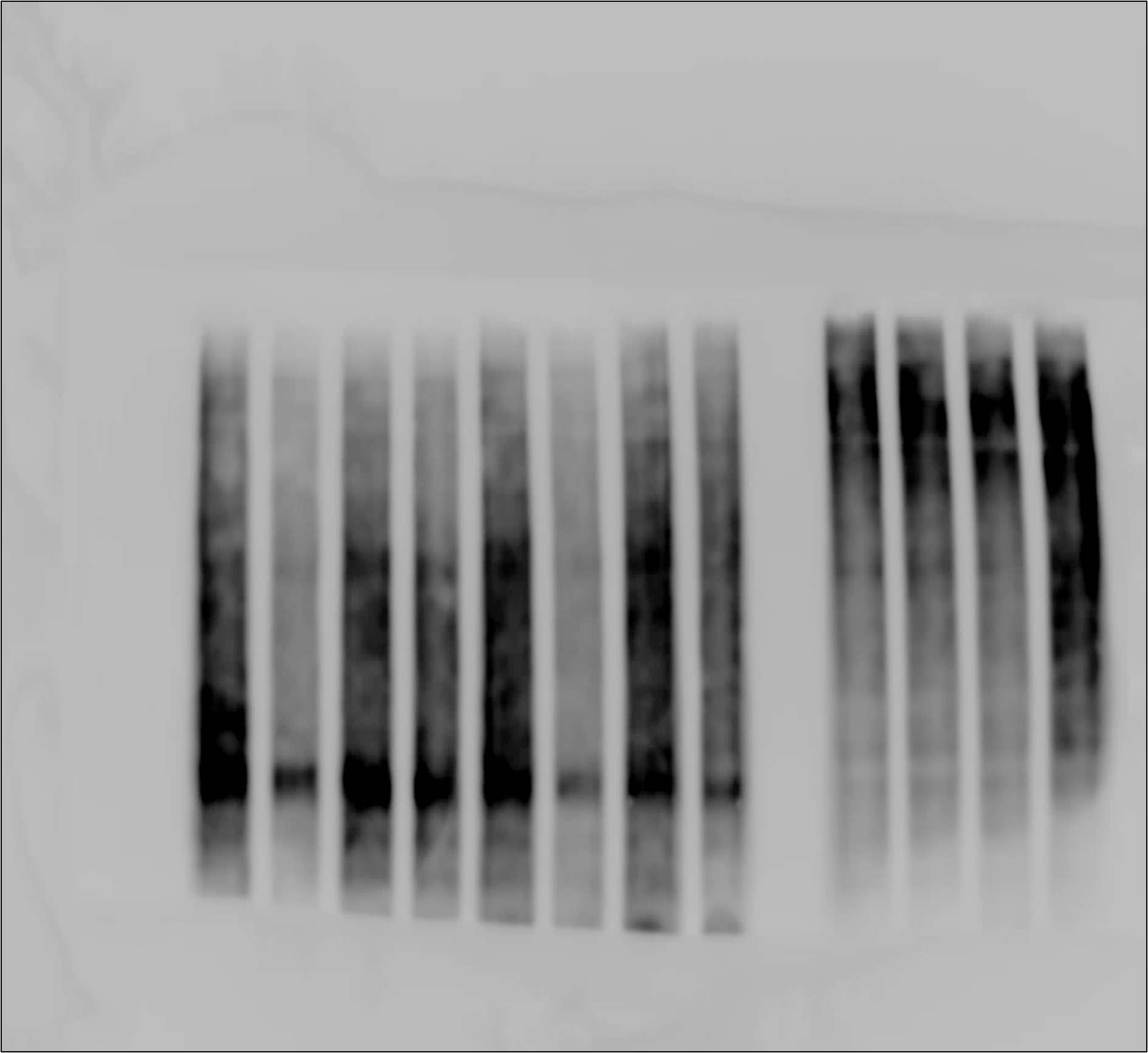

Supplement: Figure 7—source data 2. [file elife-108048-fig7-data2.zip › Figure 7/Figure 7 B-IP-HA.tif]

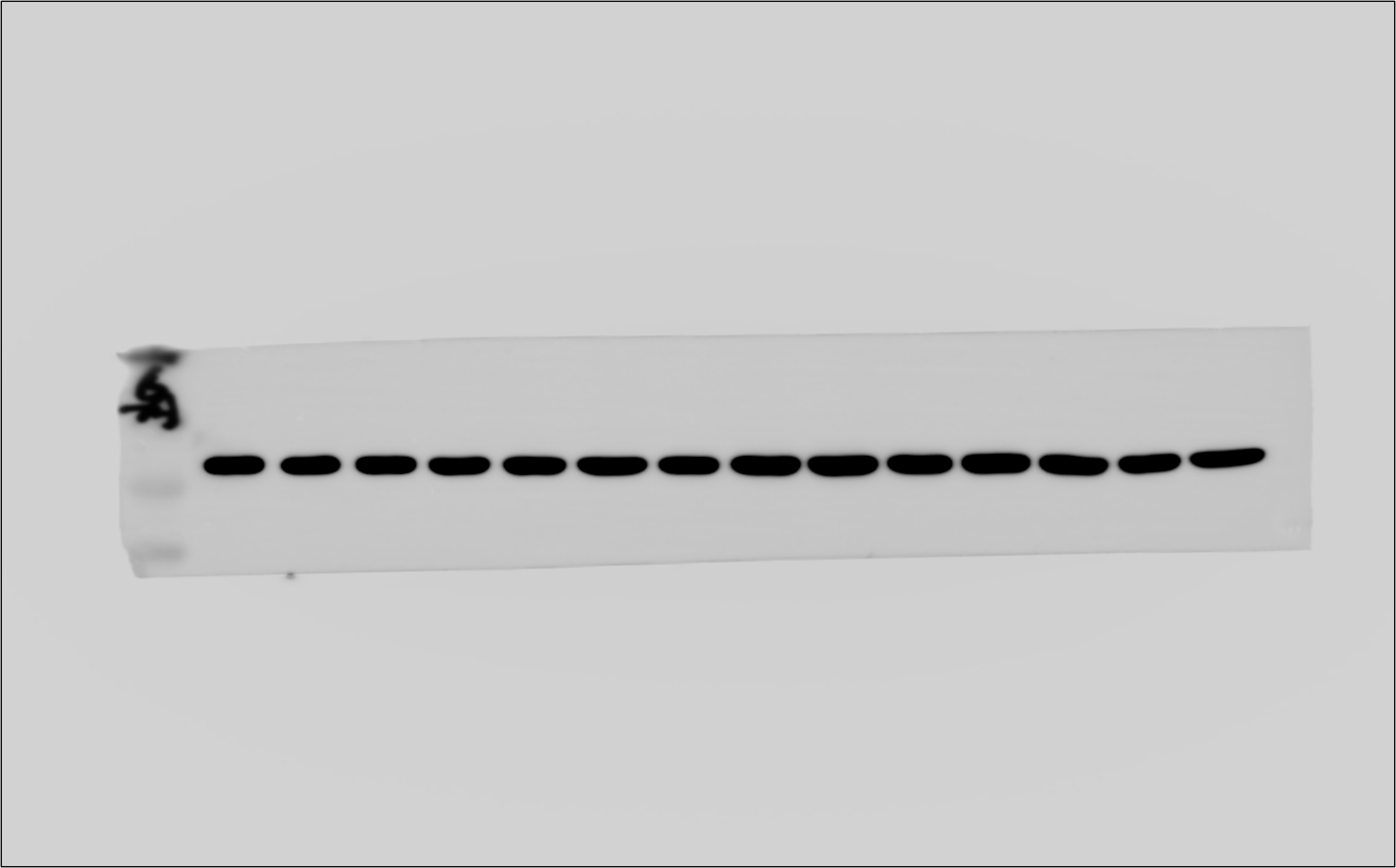

Supplement: Figure 7—source data 2. [file elife-108048-fig7-data2.zip › Figure 7/Figure 7 B-WCL-Actin.tif]

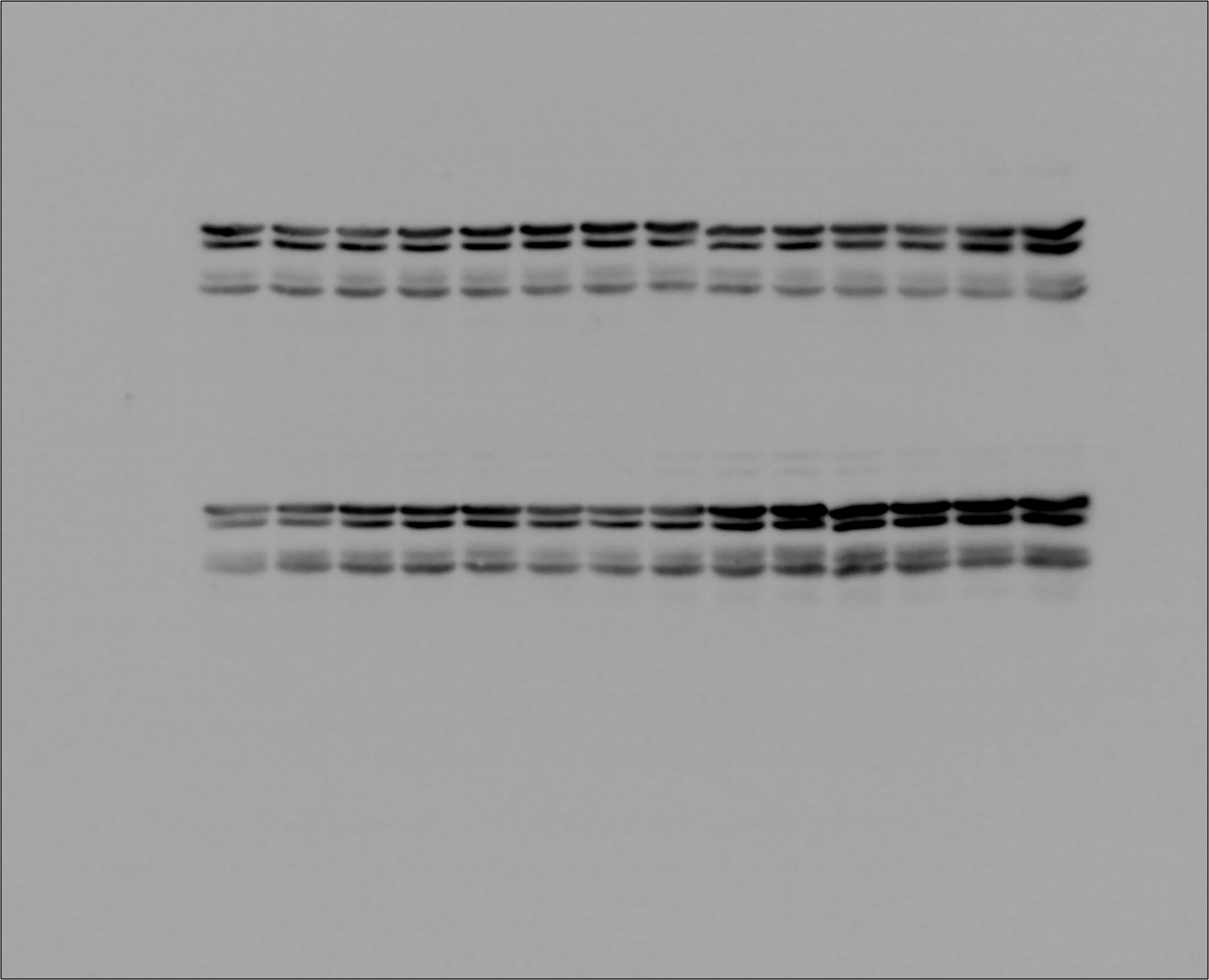

Supplement: Figure 7—source data 2. [file elife-108048-fig7-data2.zip › Figure 7/Figure 7 B-WCL-Flag.tif]

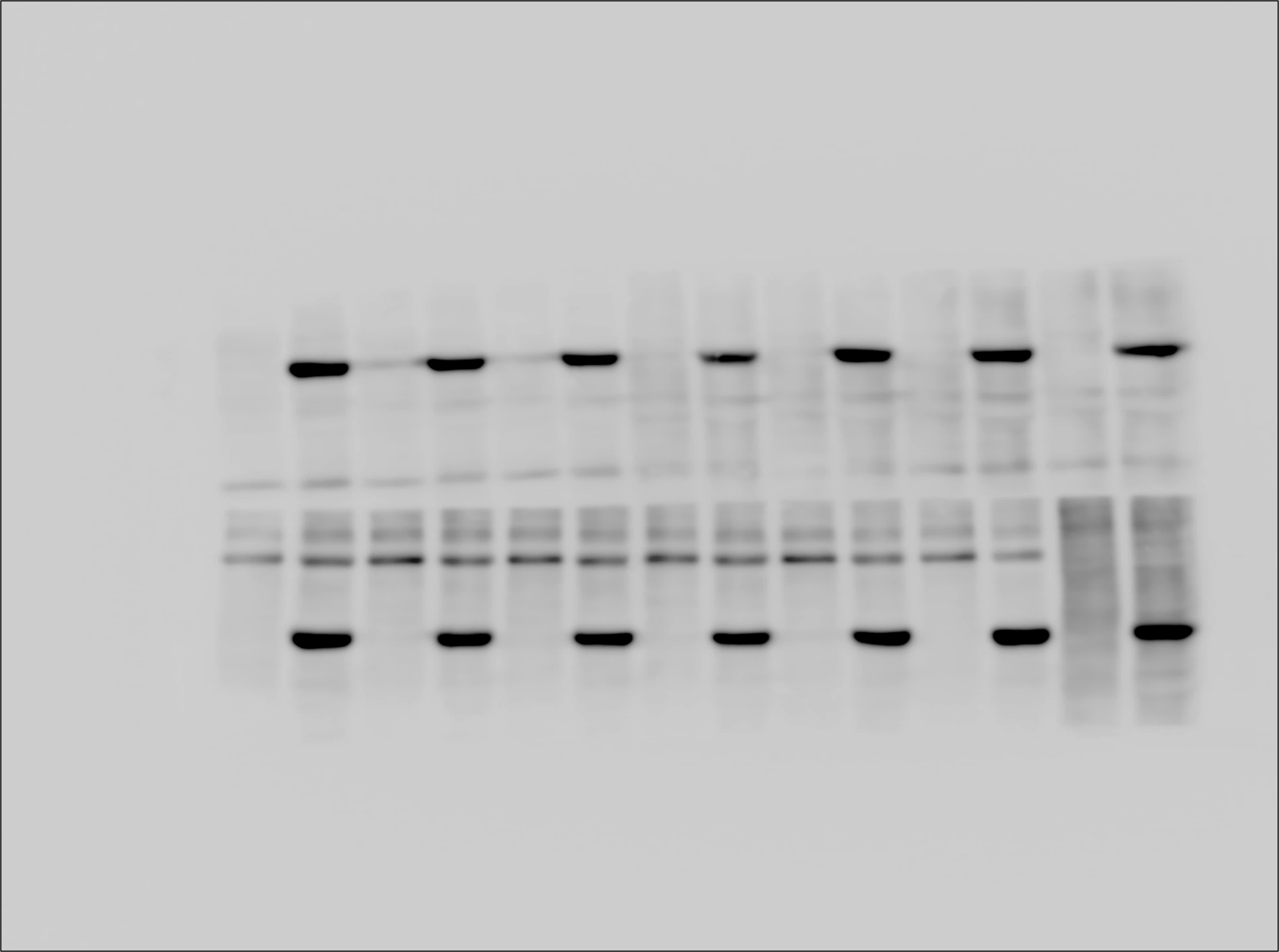

Supplement: Figure 7—source data 2. [file elife-108048-fig7-data2.zip › Figure 7/Figure 7 B-WCL-HA-cyp17a2.tif]

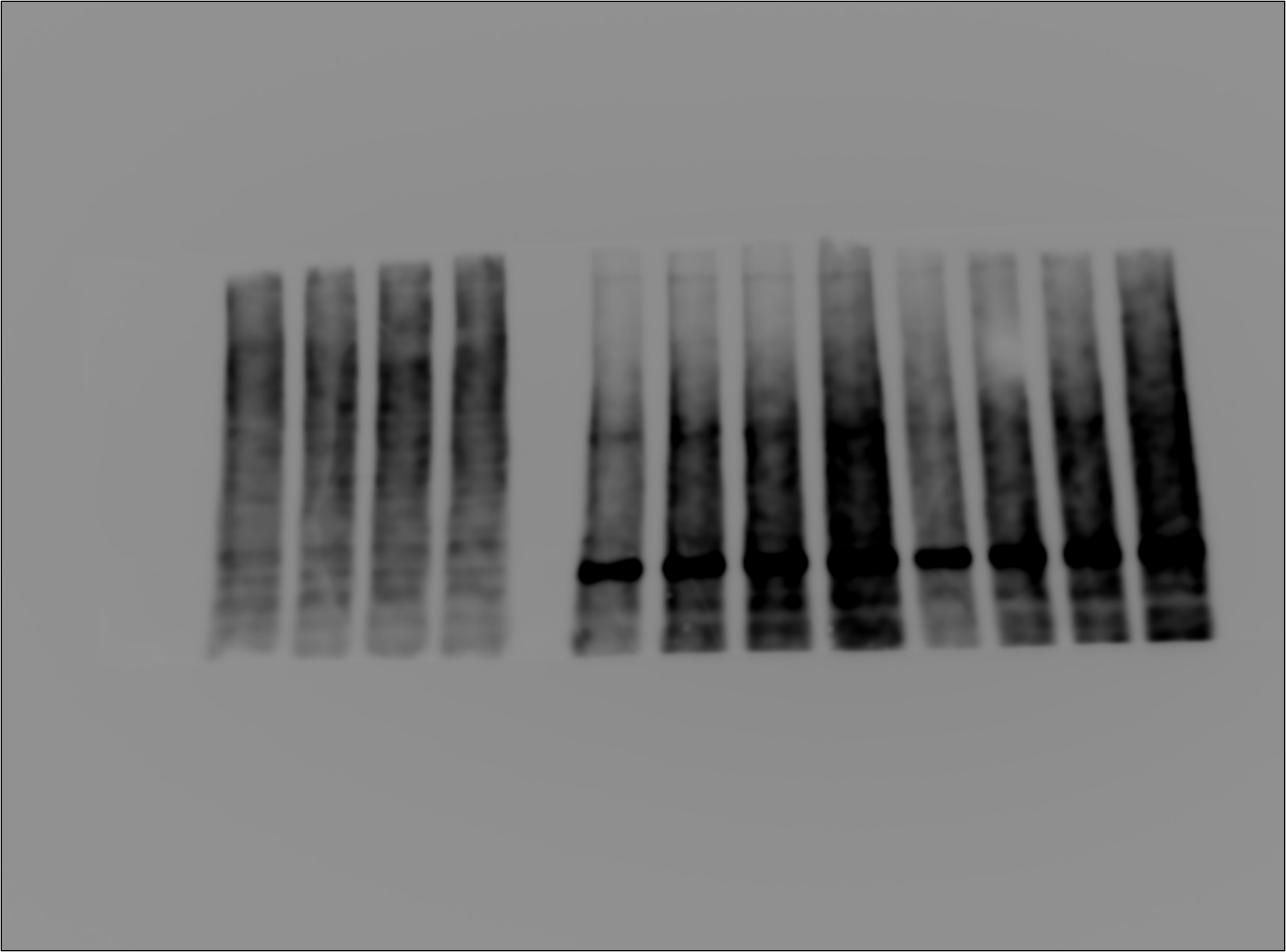

Supplement: Figure 7—source data 2. [file elife-108048-fig7-data2.zip › Figure 7/Figure 7 B-WCL-HA.tif]

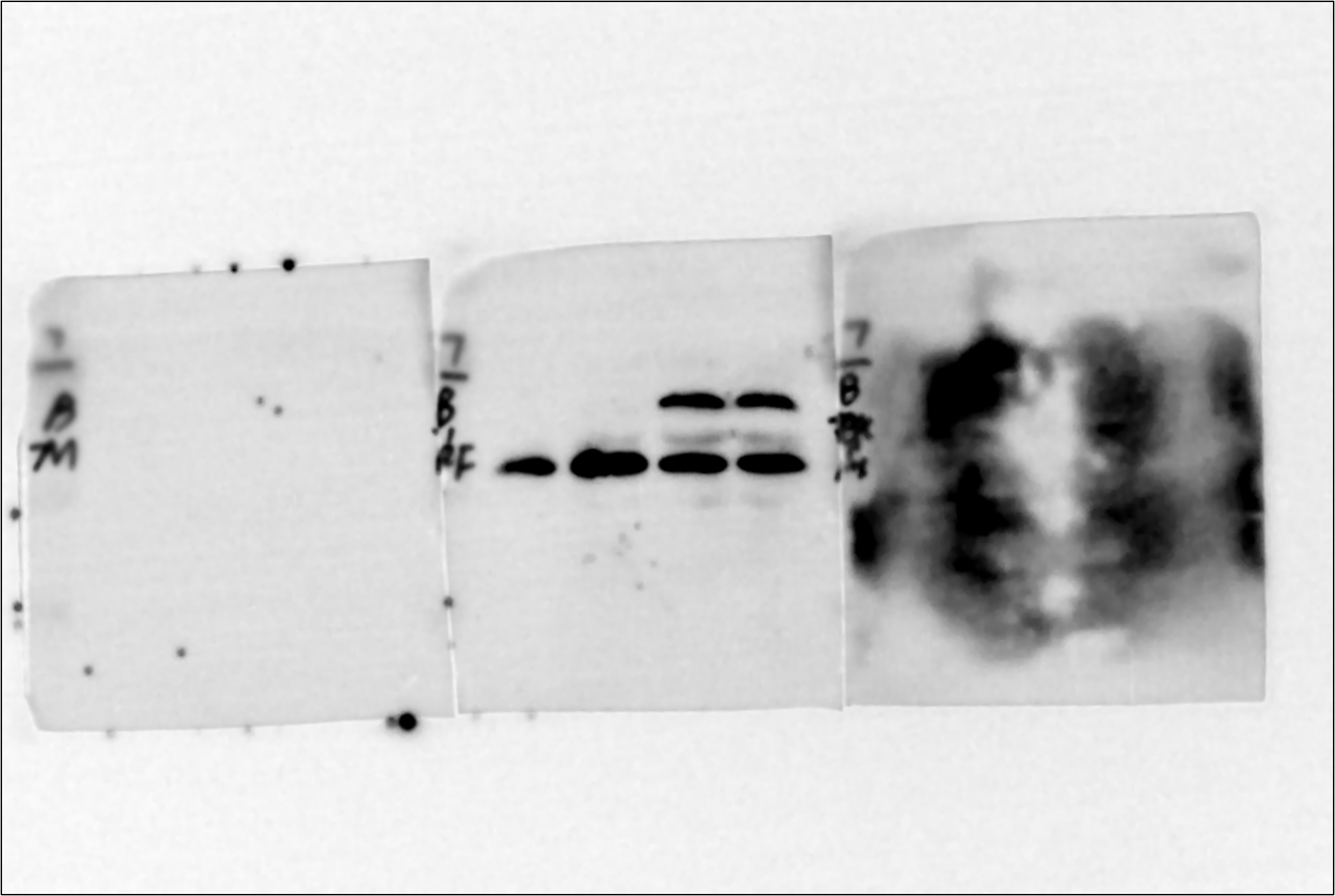

Supplement: Figure 7—source data 2. [file elife-108048-fig7-data2.zip › Figure 7/Figure 7 B-WCL-Myc.tif]

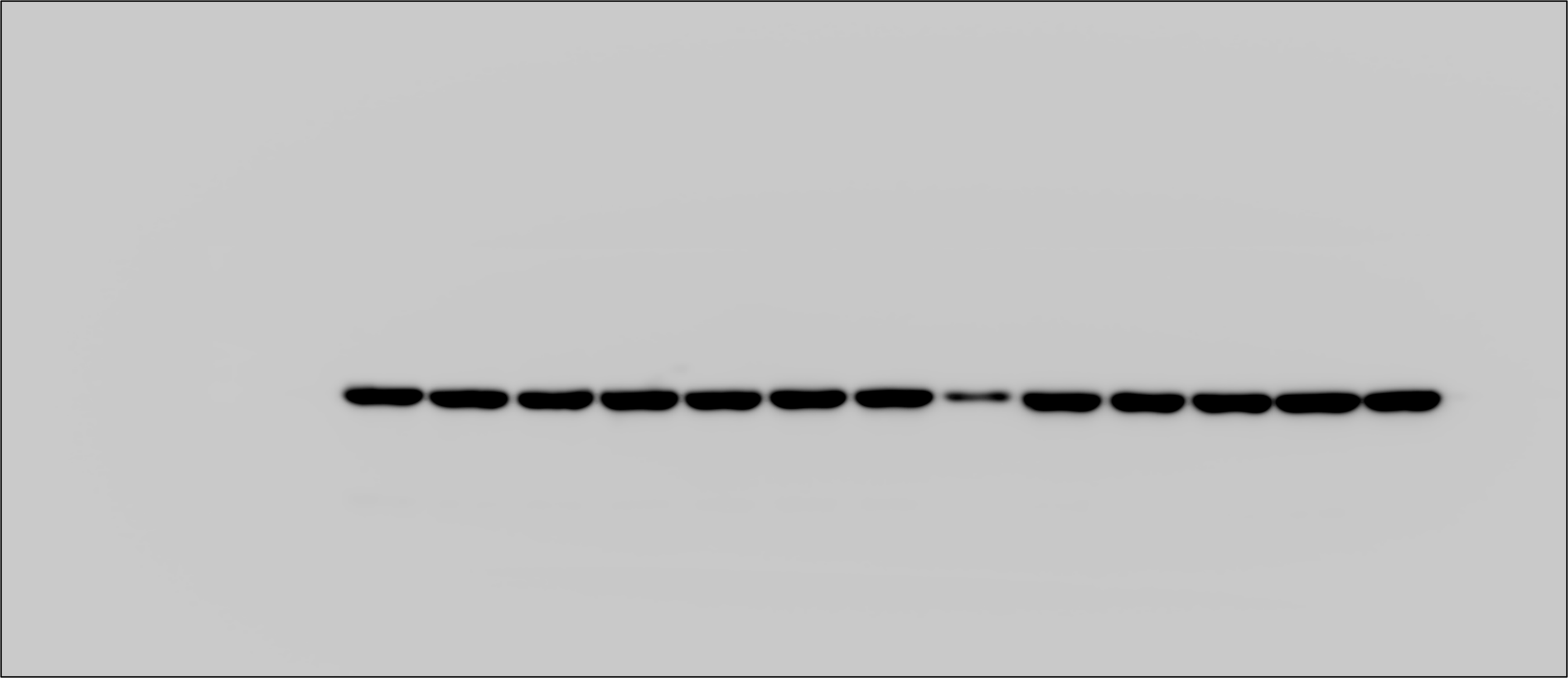

Supplement: Figure 7—source data 2. [file elife-108048-fig7-data2.zip › Figure 7/Figure 7 D-Actin.tif]

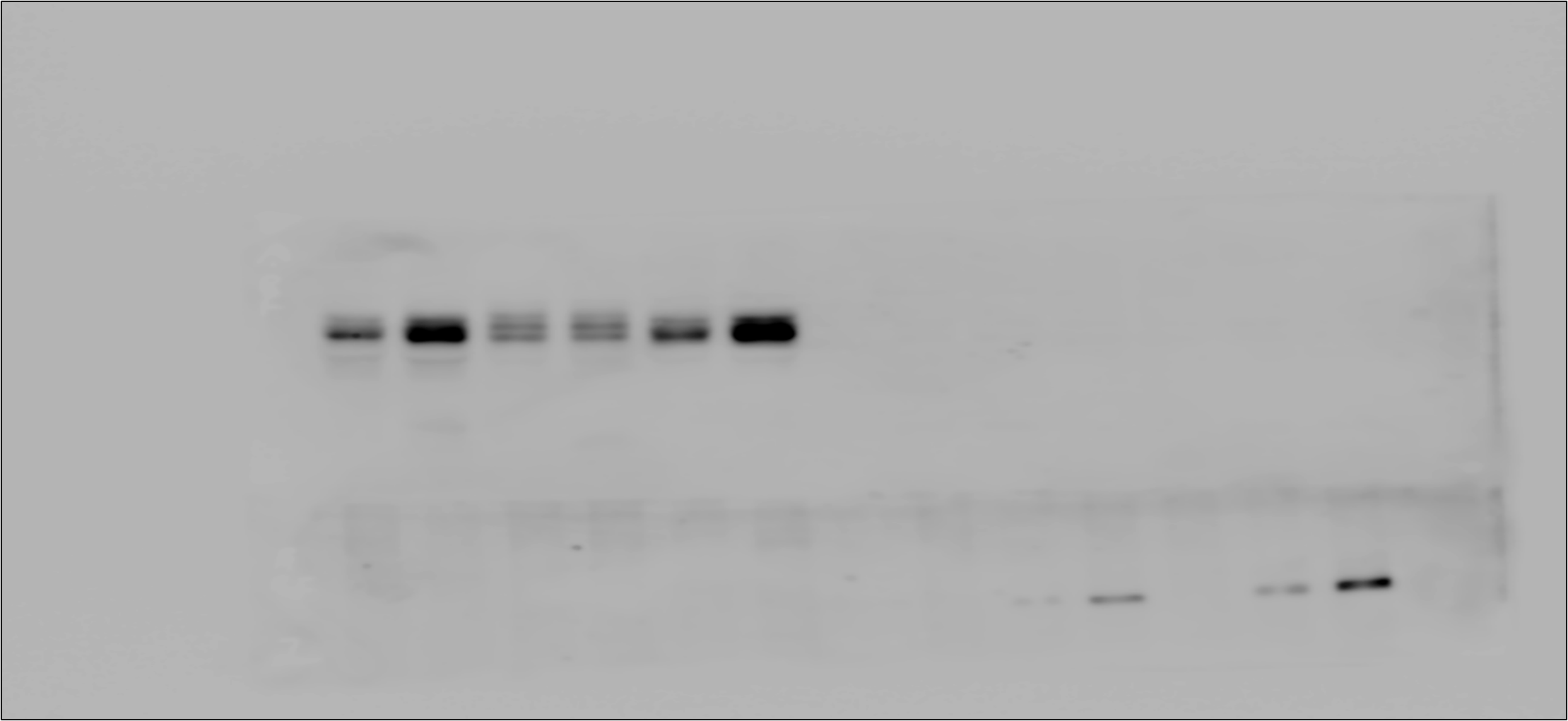

Supplement: Figure 7—source data 2. [file elife-108048-fig7-data2.zip › Figure 7/Figure 7 D-Flag.tif]

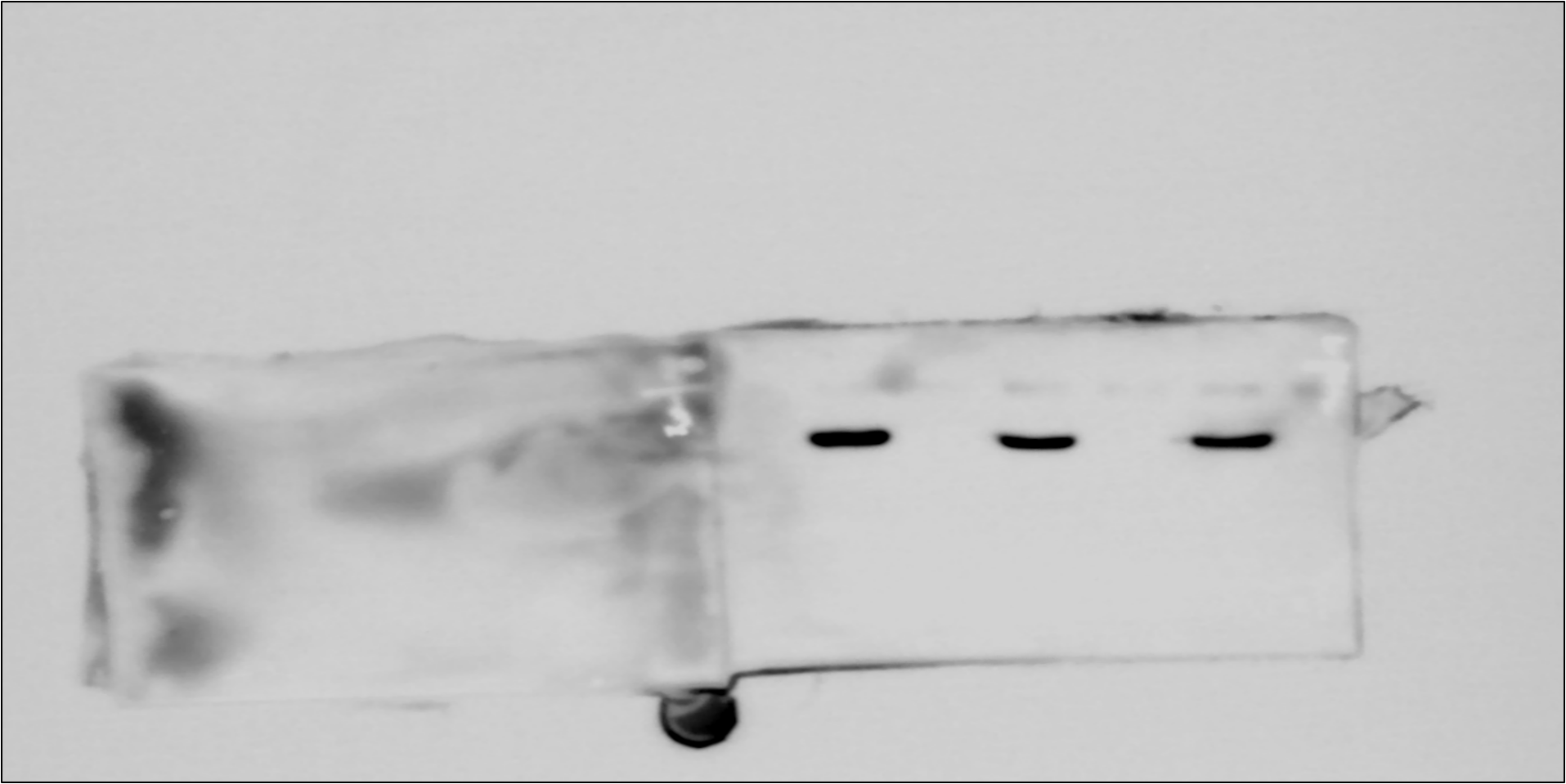

Supplement: Figure 7—source data 2. [file elife-108048-fig7-data2.zip › Figure 7/Figure 7 D-HA.tif]

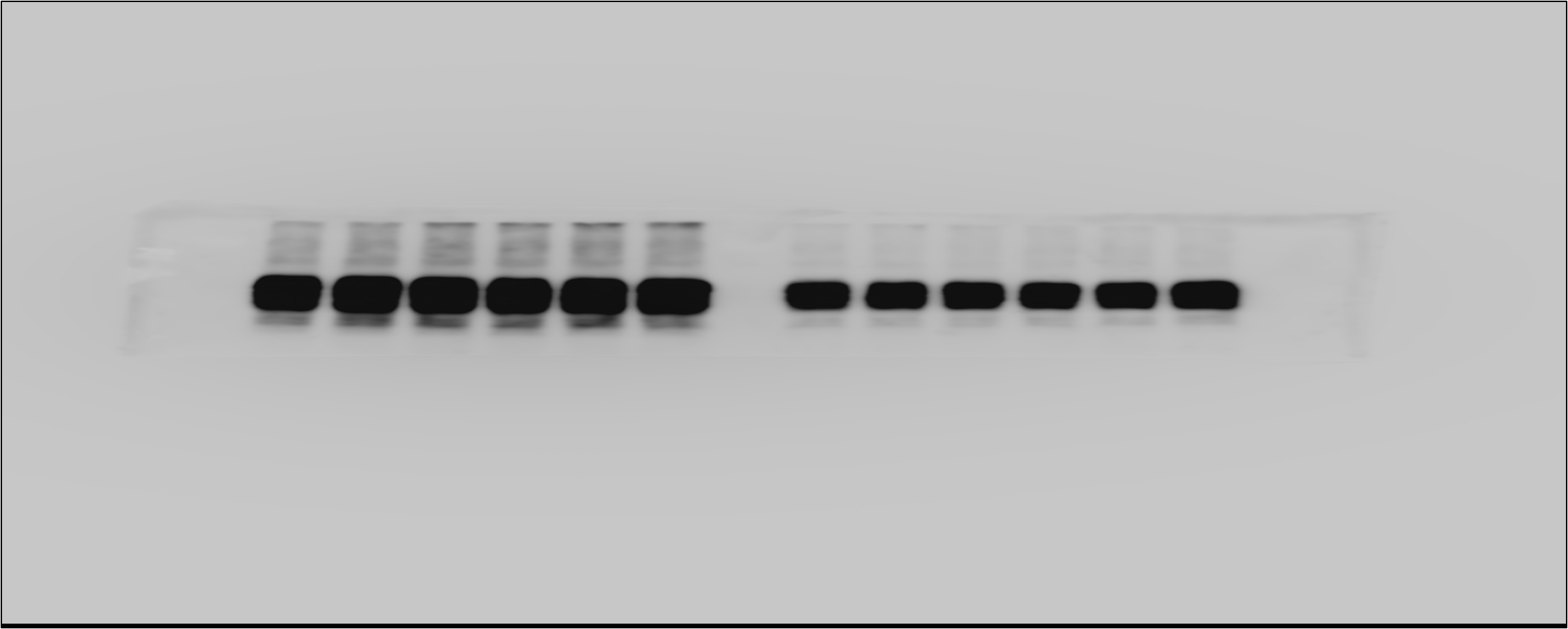

Supplement: Figure 7—source data 2. [file elife-108048-fig7-data2.zip › Figure 7/Figure 7 G-IP-Flag.tif]

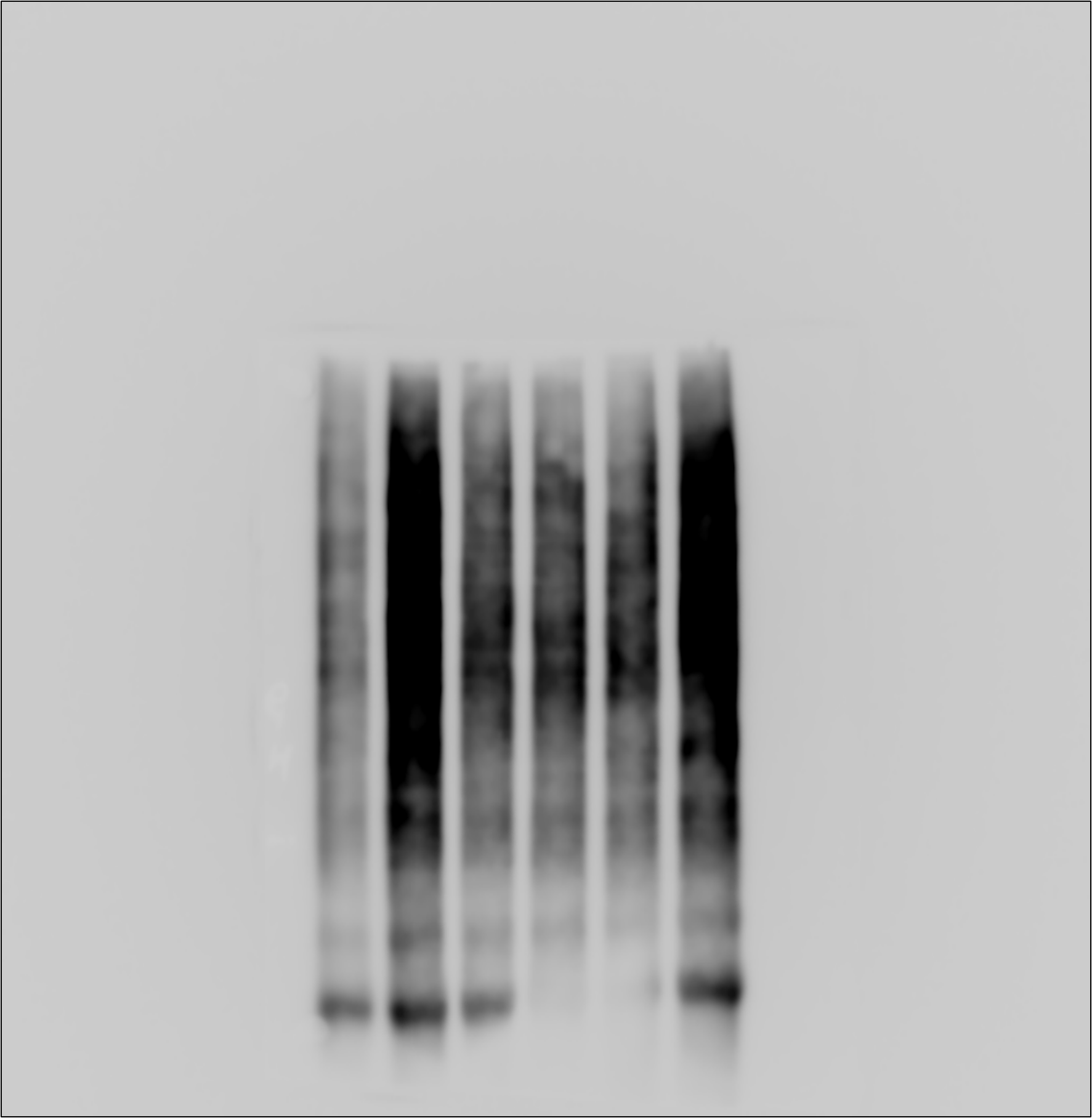

Supplement: Figure 7—source data 2. [file elife-108048-fig7-data2.zip › Figure 7/Figure 7 G-IP-HA.tif]

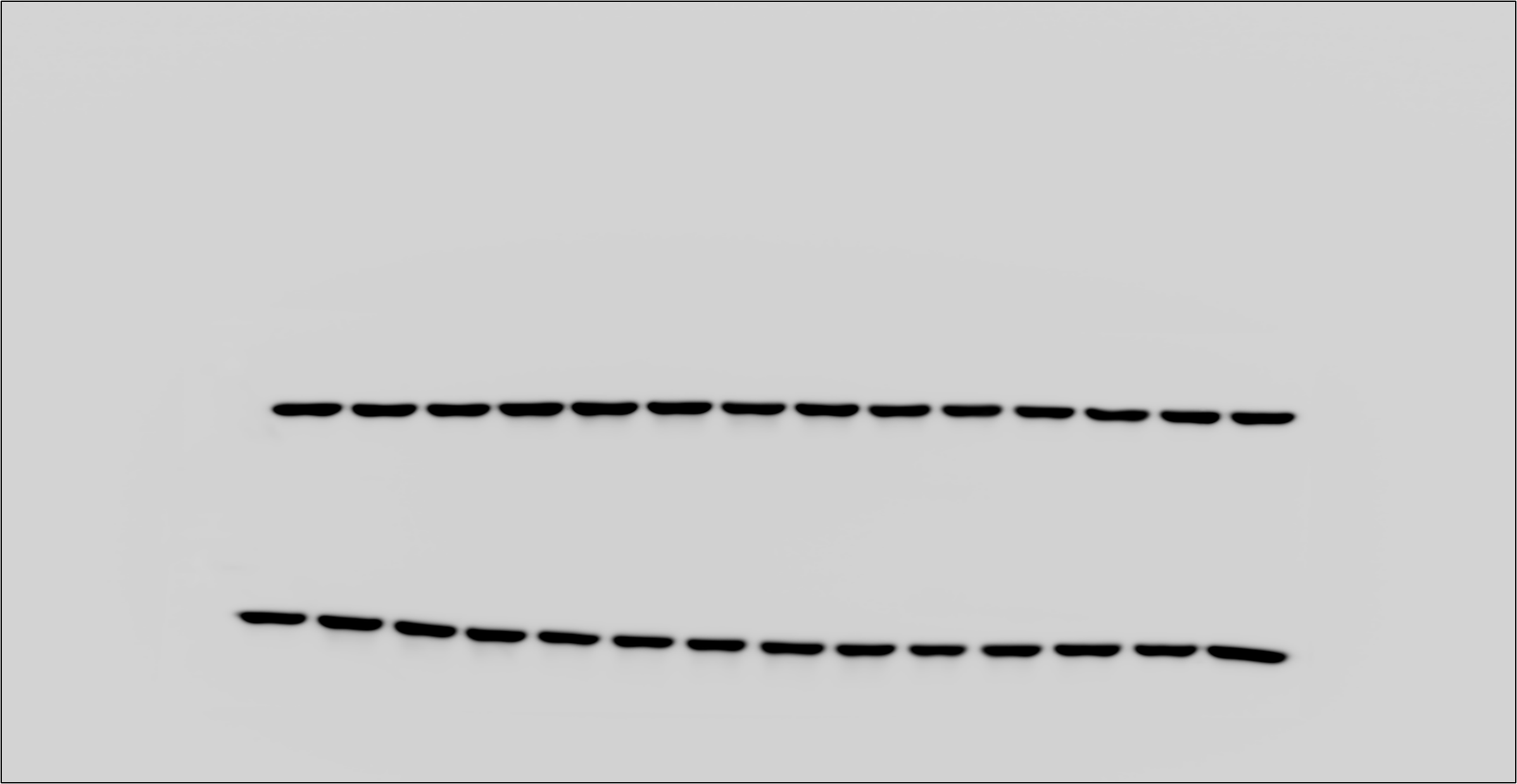

Supplement: Figure 7—source data 2. [file elife-108048-fig7-data2.zip › Figure 7/Figure 7 G-WCL-Actin.tif]

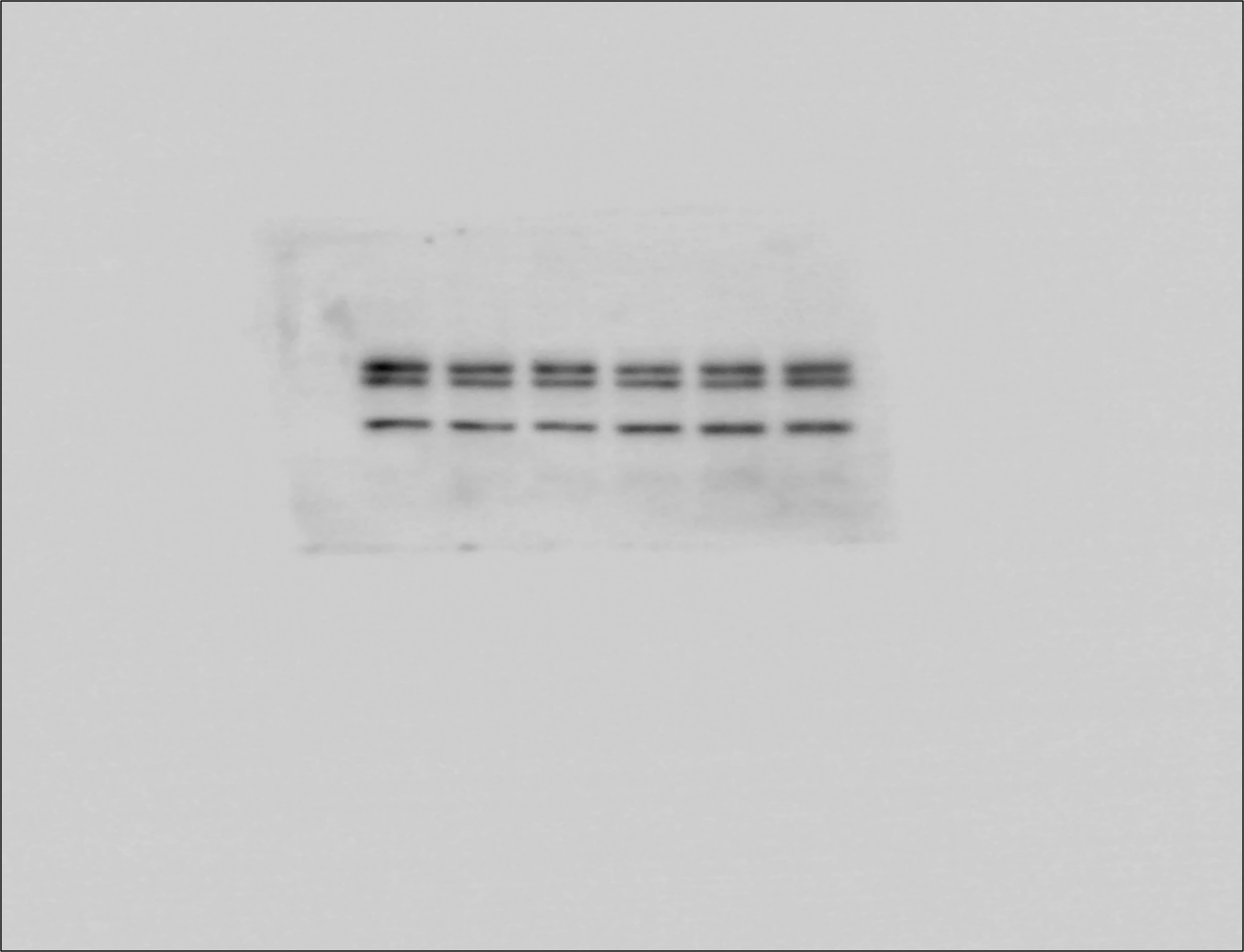

Supplement: Figure 7—source data 2. [file elife-108048-fig7-data2.zip › Figure 7/Figure 7 G-WCL-Flag.tif]

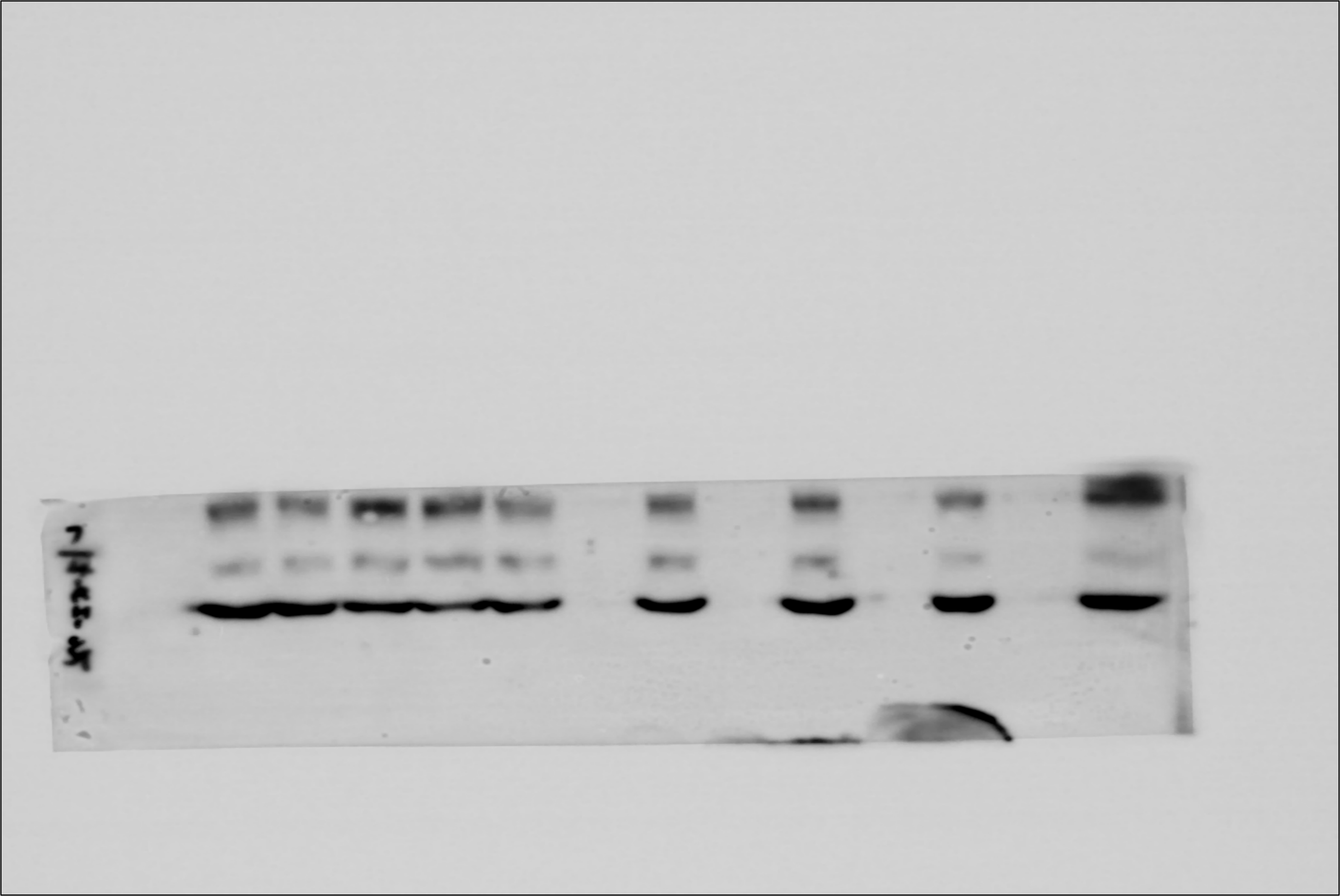

Supplement: Figure 7—source data 2. [file elife-108048-fig7-data2.zip › Figure 7/Figure 7 G-WCL-HA-cyp17a2.tif]

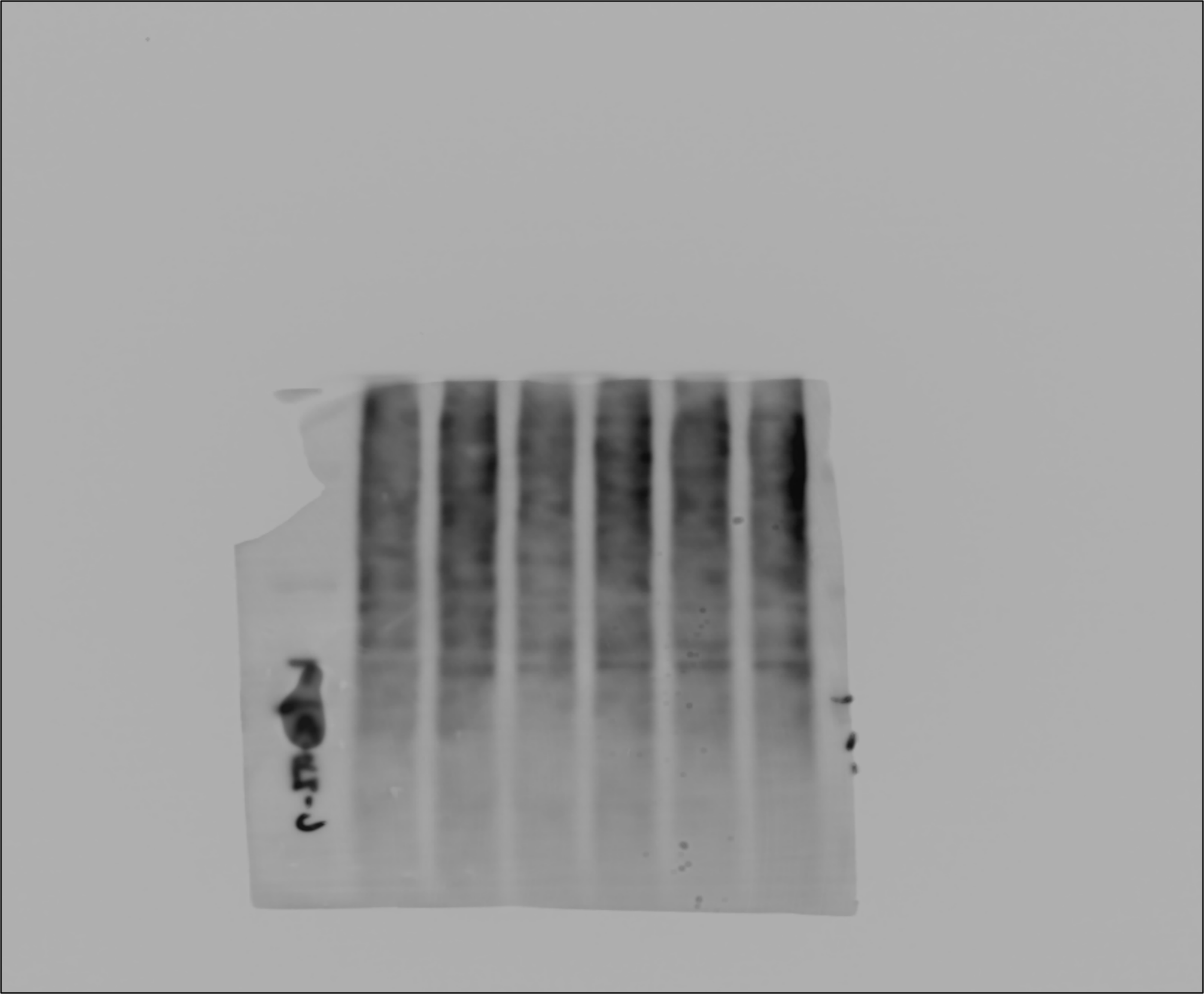

Supplement: Figure 7—source data 2. [file elife-108048-fig7-data2.zip › Figure 7/Figure 7 G-WCL-HA.tif]

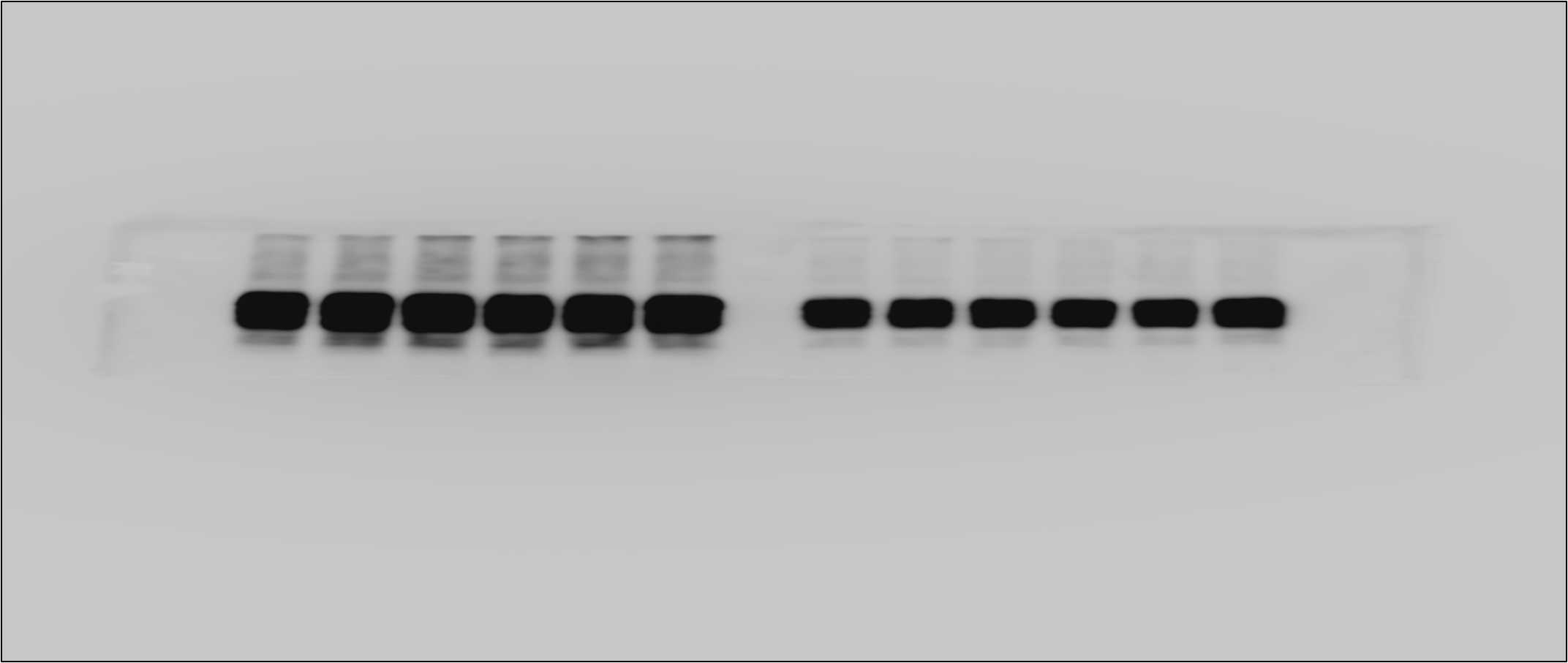

Supplement: Figure 7—source data 2. [file elife-108048-fig7-data2.zip › Figure 7/Figure 7 H-IP-Flag.tif]

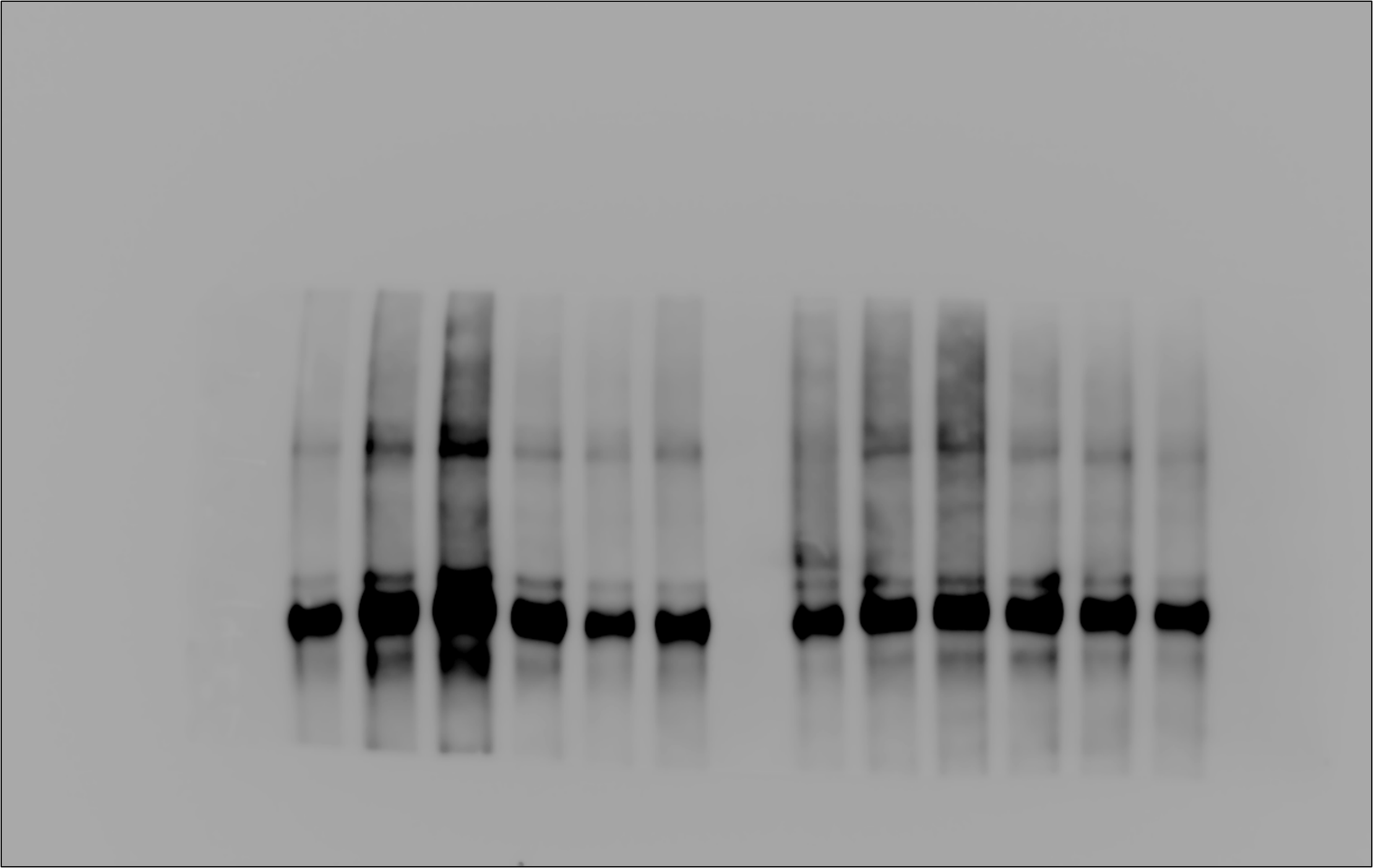

Supplement: Figure 7—source data 2. [file elife-108048-fig7-data2.zip › Figure 7/Figure 7 H-IP-HA.tif]

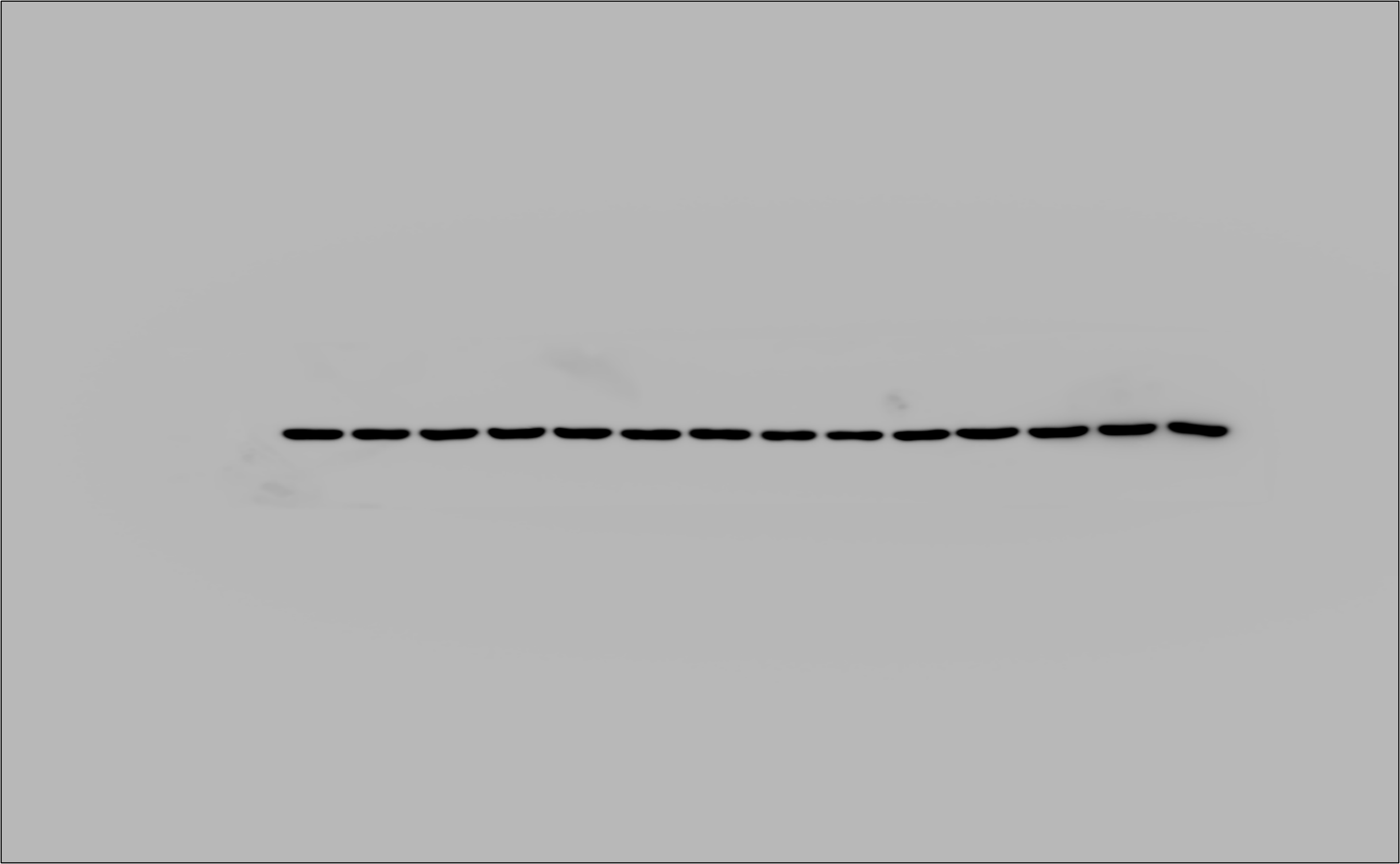

Supplement: Figure 7—source data 2. [file elife-108048-fig7-data2.zip › Figure 7/Figure 7 H-WCL-Actin.tif]

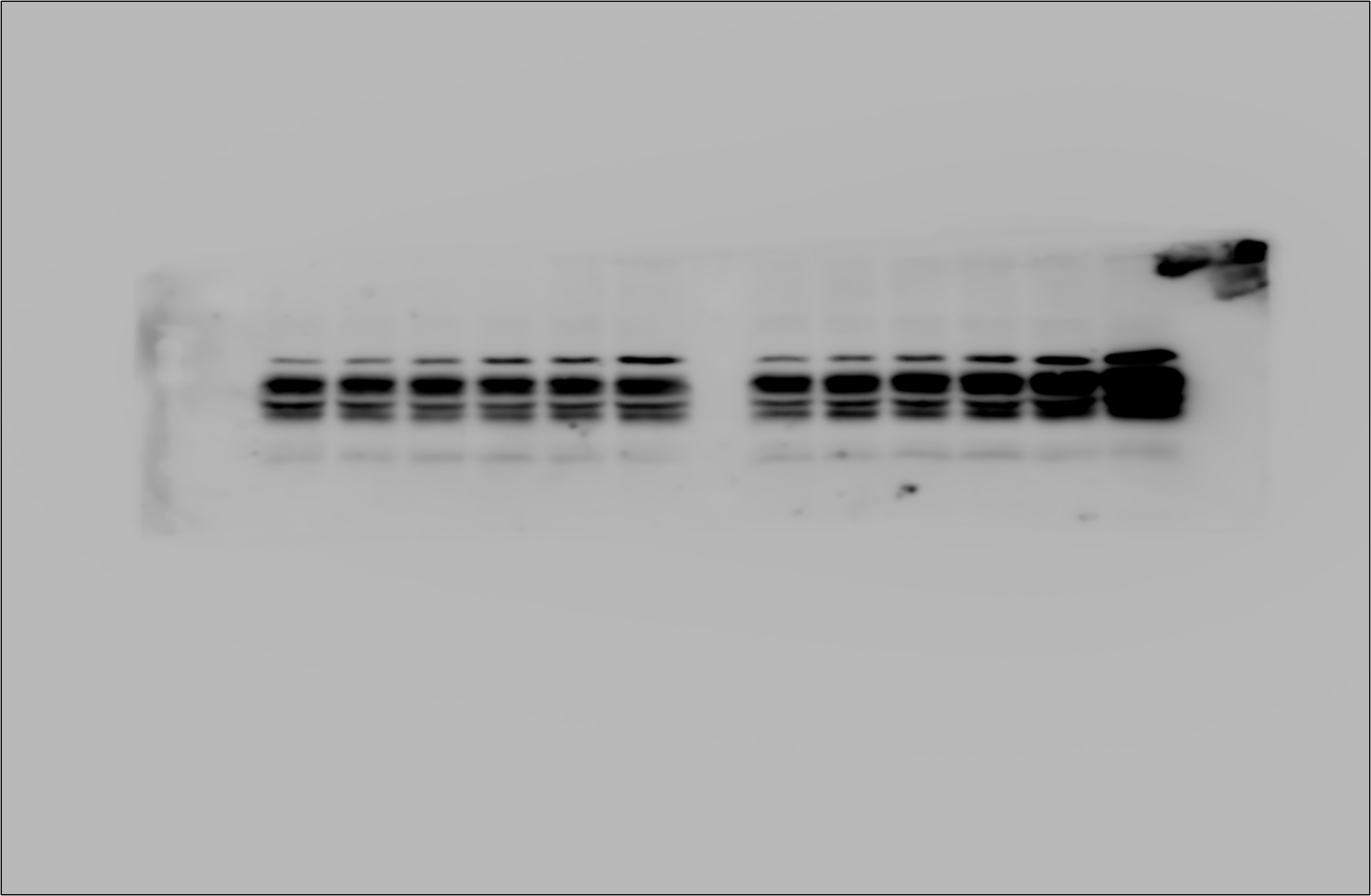

Supplement: Figure 7—source data 2. [file elife-108048-fig7-data2.zip › Figure 7/Figure 7 H-WCL-Flag.tif]

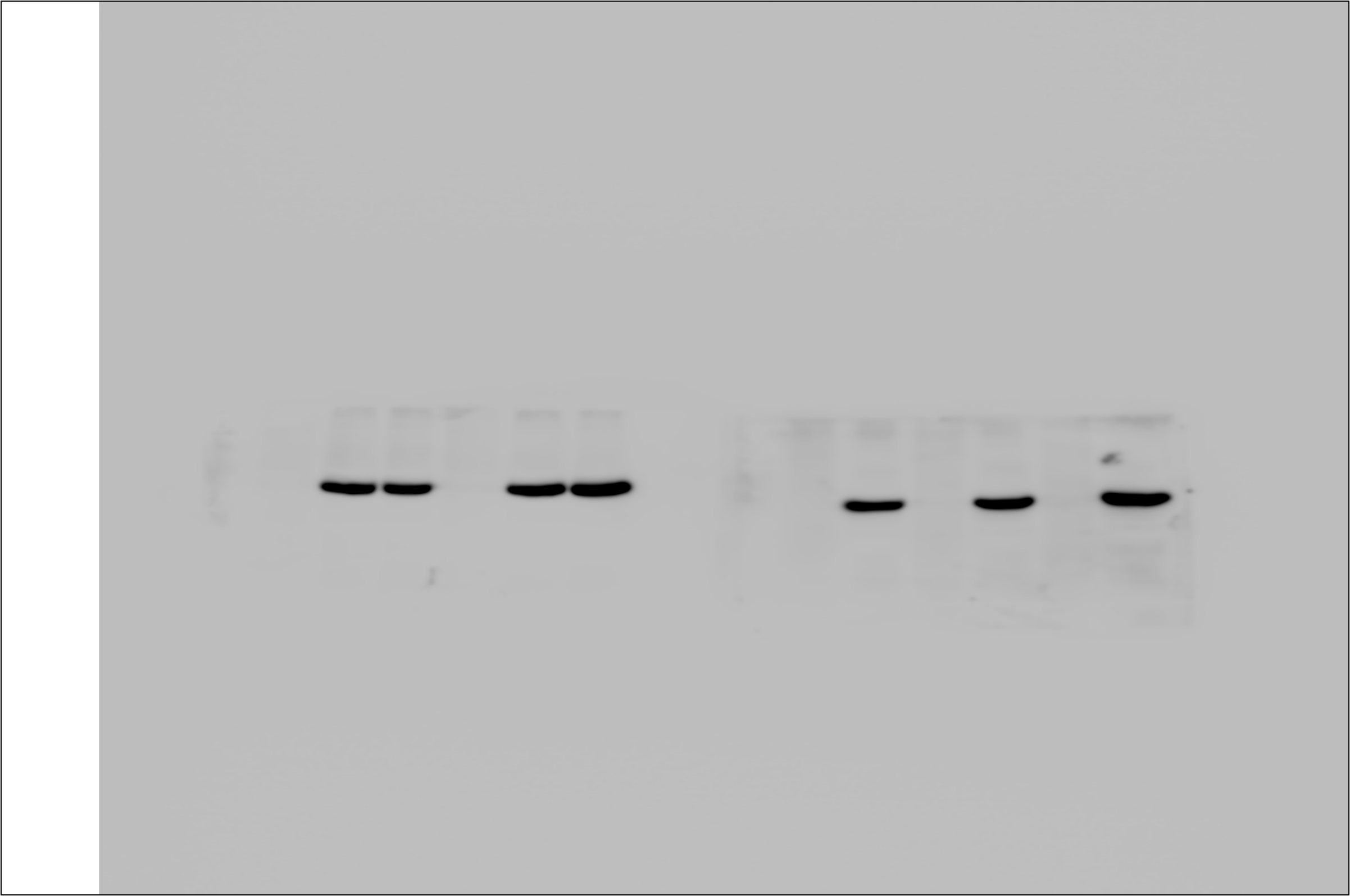

Supplement: Figure 7—source data 2. [file elife-108048-fig7-data2.zip › Figure 7/Figure 7 H-WCL-HA-cyp17a2.tif]

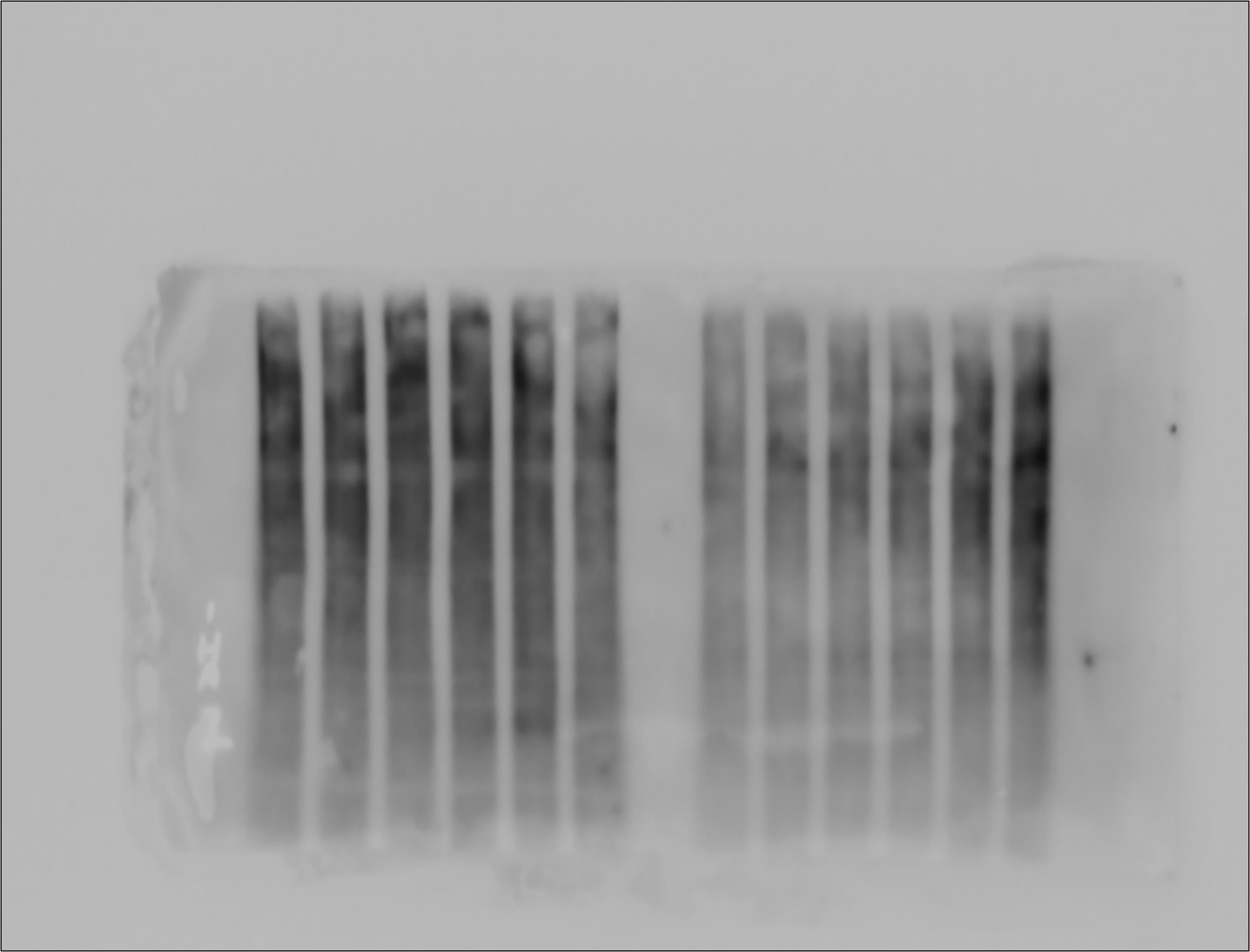

Supplement: Figure 7—source data 2. [file elife-108048-fig7-data2.zip › Figure 7/Figure 7 H-WCL-HA.tif]

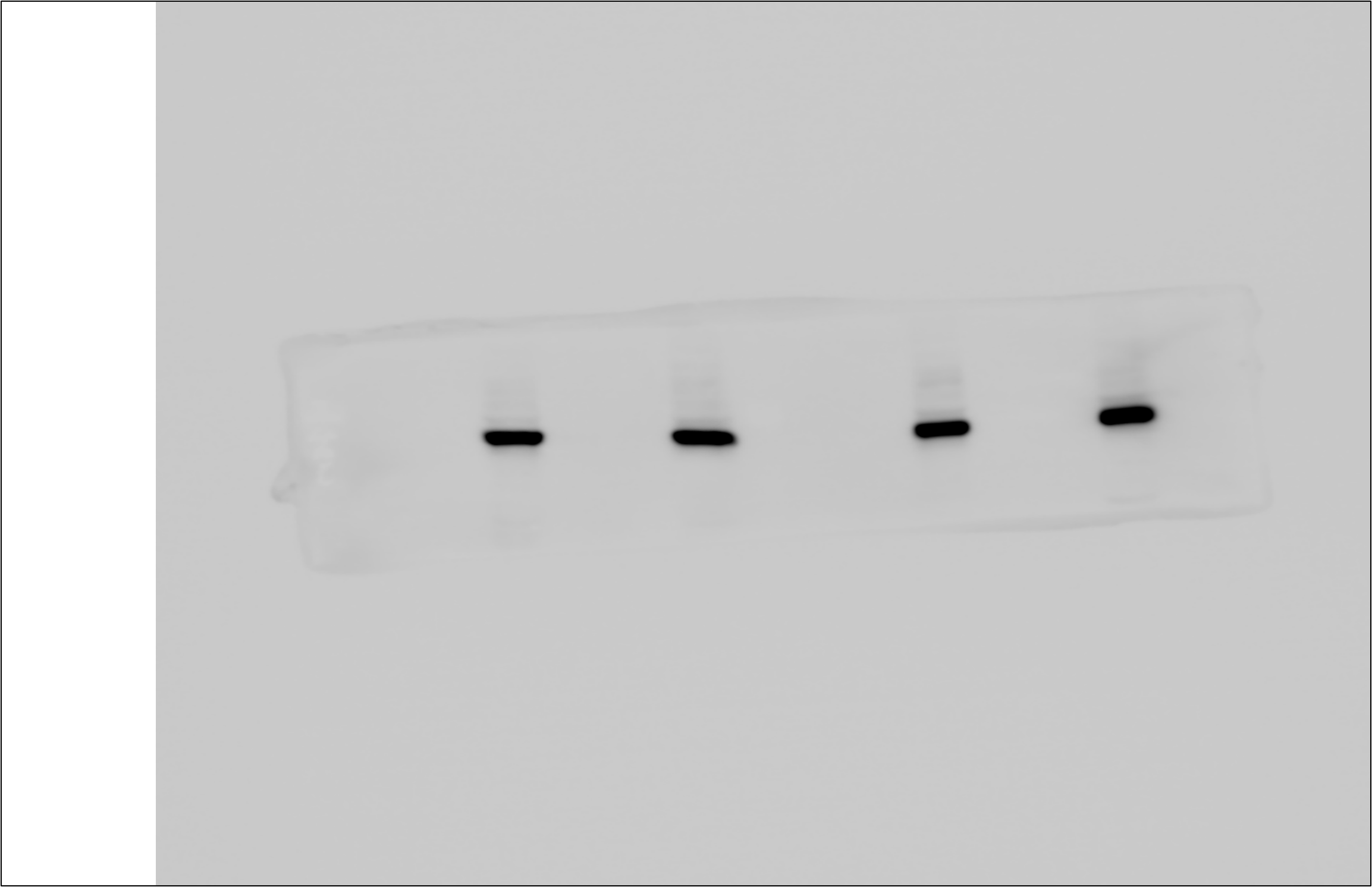

Supplement: Figure 7—source data 2. [file elife-108048-fig7-data2.zip › Figure 7/Figure 7 H-WCL-Myc.tif]
