# Supplementary figures and images for: Male-biased Cyp17a2 orchestrates antiviral sexual dimorphism in fish via STING stabilization and viral protein degradation (part 3 of 5)
Source: eLife. 2026 Feb 18;14:RP108048. doi: 10.7554/eLife.108048 (PMC12916102; doi:10.7554/eLife.108048)

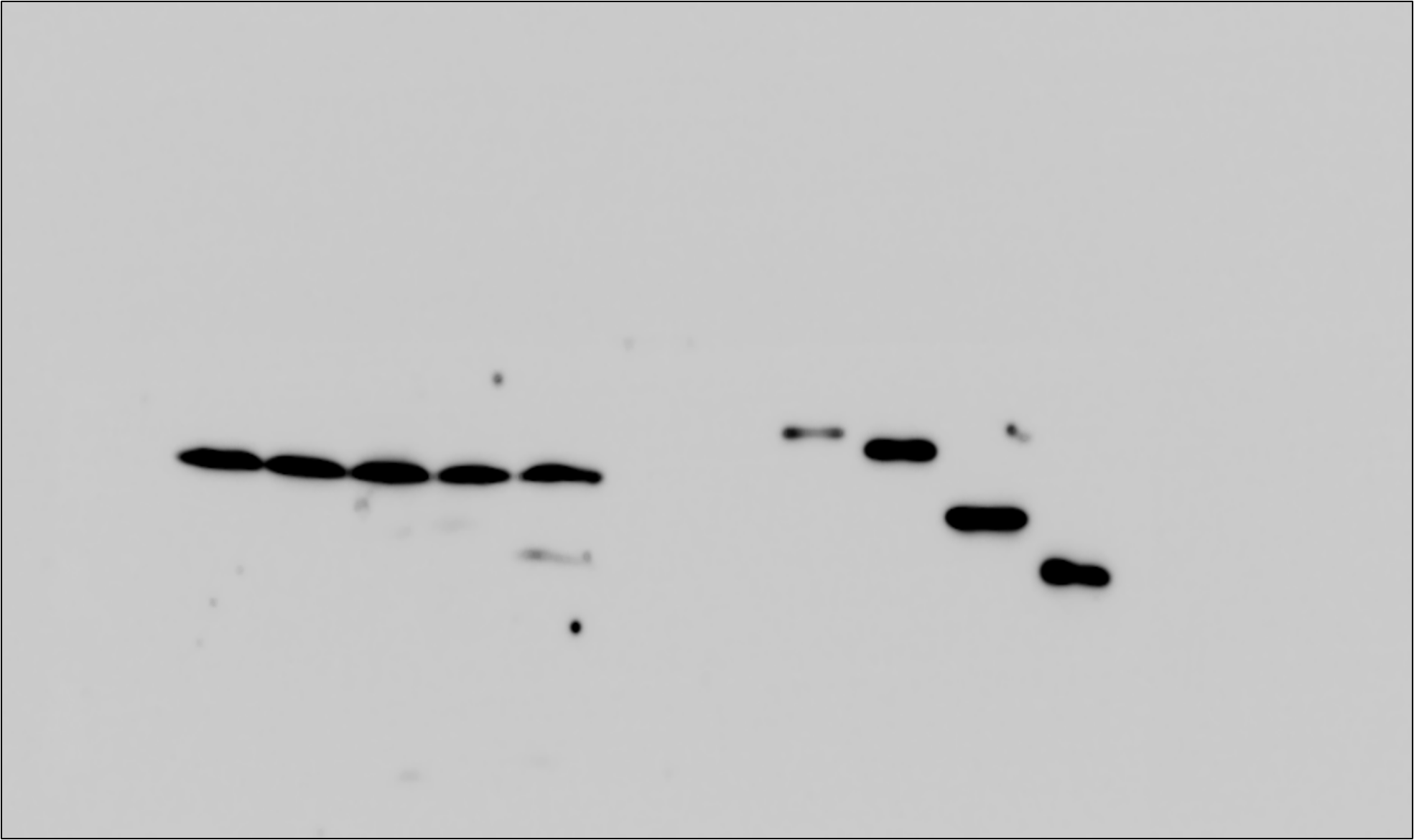

Supplement: Figure 7—source data 2. [file elife-108048-fig7-data2.zip › Figure 7/Figure 7 J-IP-Flag.tif]

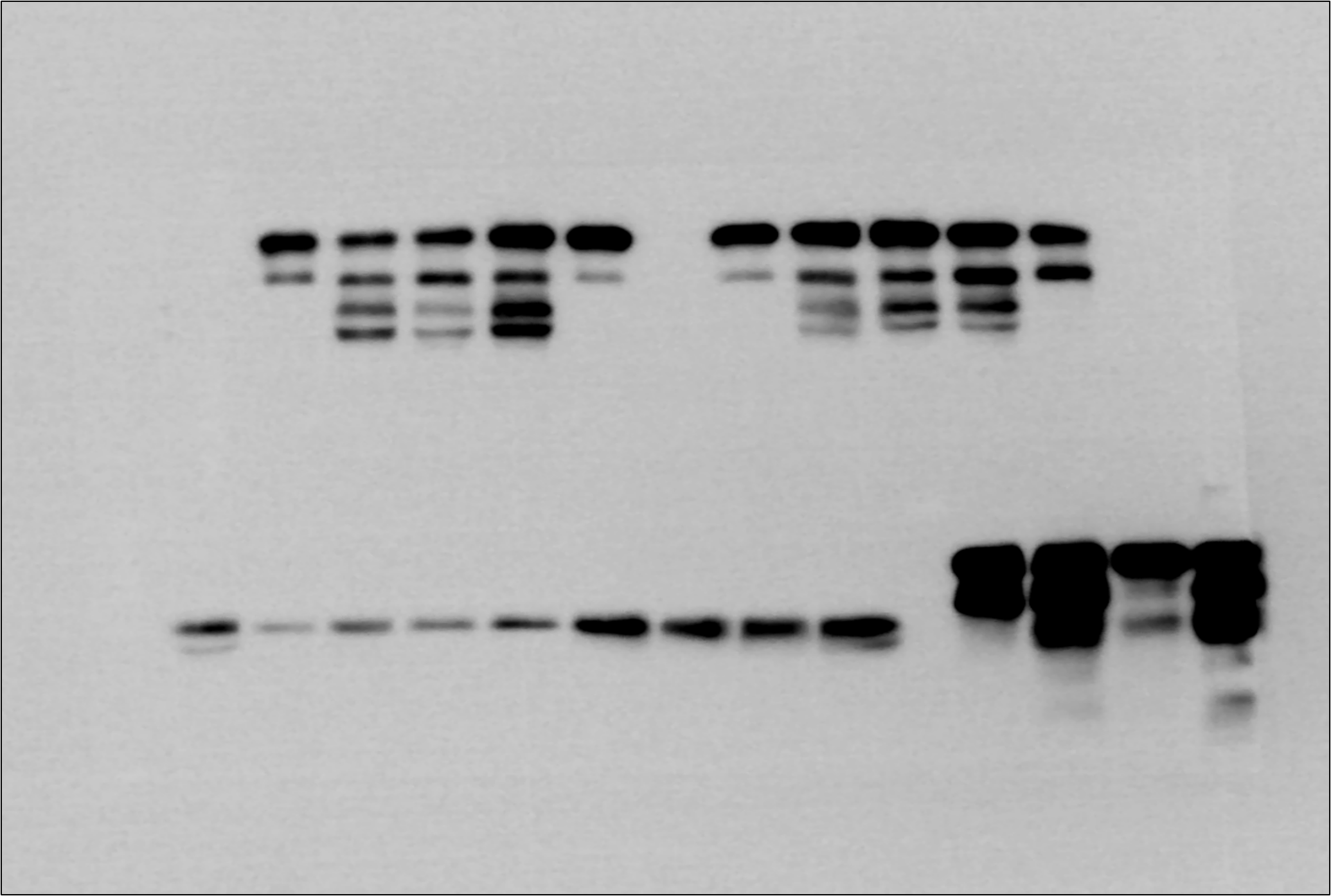

Supplement: Figure 7—source data 2. [file elife-108048-fig7-data2.zip › Figure 7/Figure 7 J-IP-HA.tif]

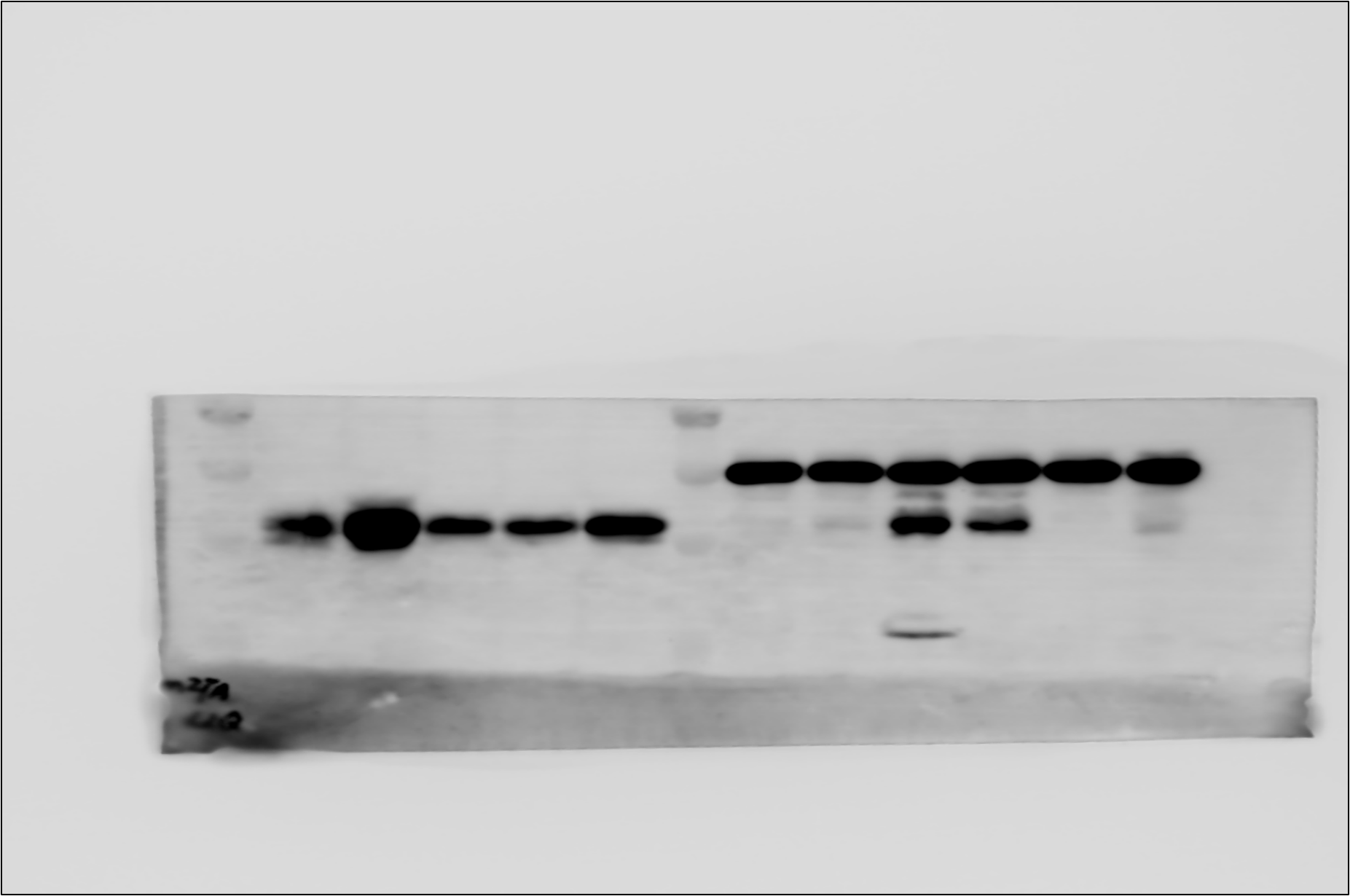

Supplement: Figure 7—source data 2. [file elife-108048-fig7-data2.zip › Figure 7/Figure 7 J-WCL-HA.tif]

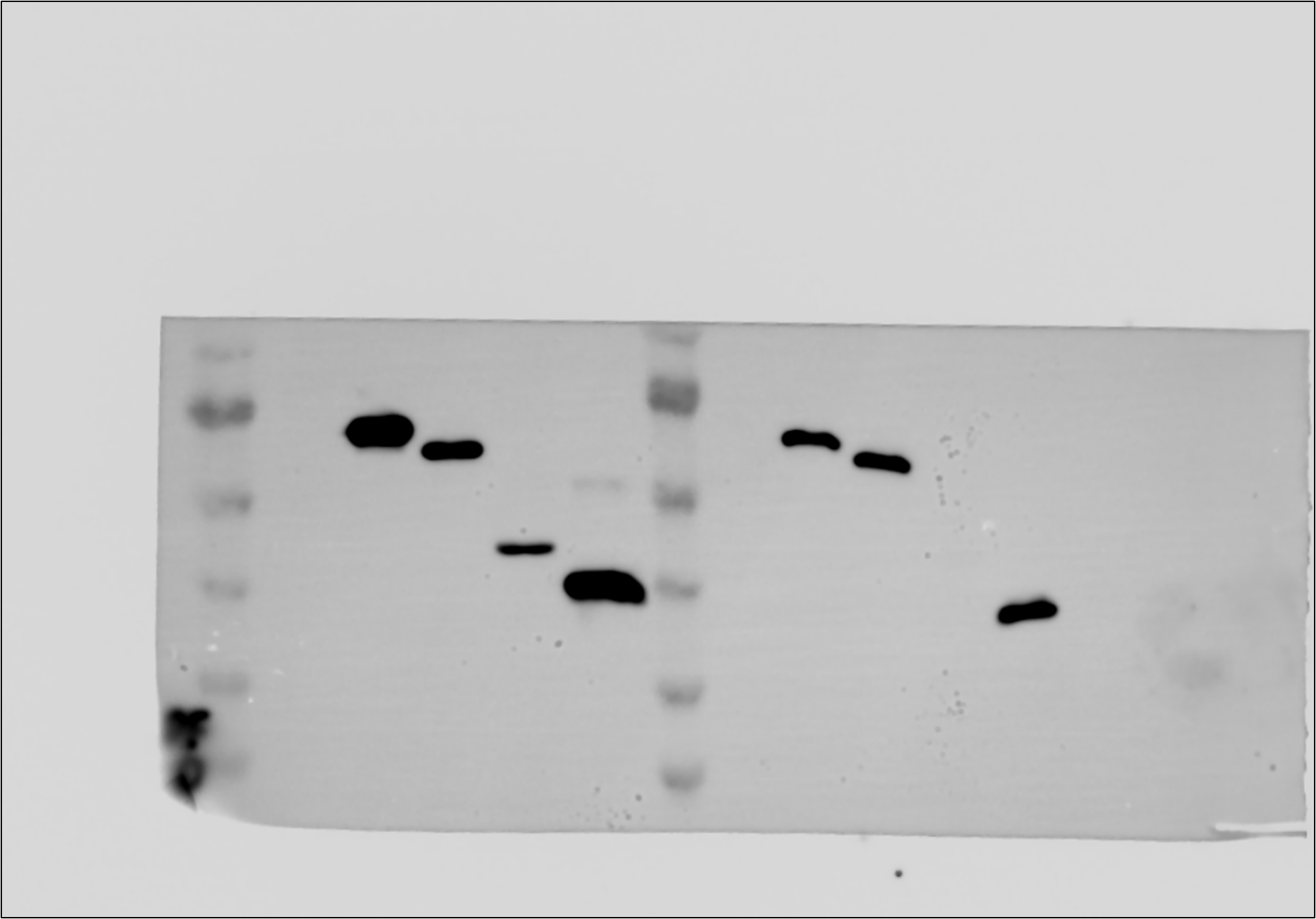

Supplement: Figure 7—source data 2. [file elife-108048-fig7-data2.zip › Figure 7/Figure 7 K-IP-Flag.tif]

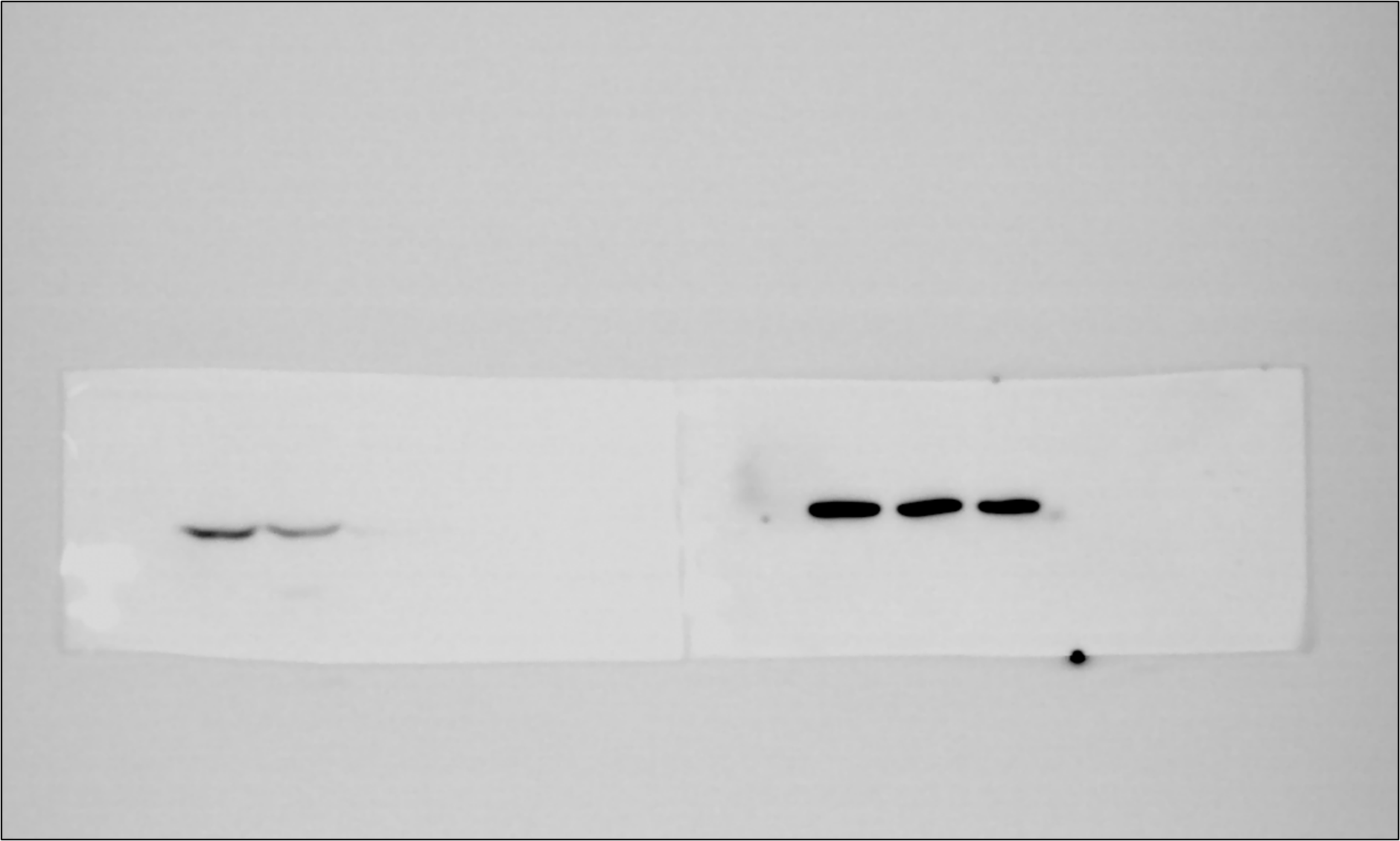

Supplement: Figure 7—source data 2. [file elife-108048-fig7-data2.zip › Figure 7/Figure 7 K-IP-Myc.tif]

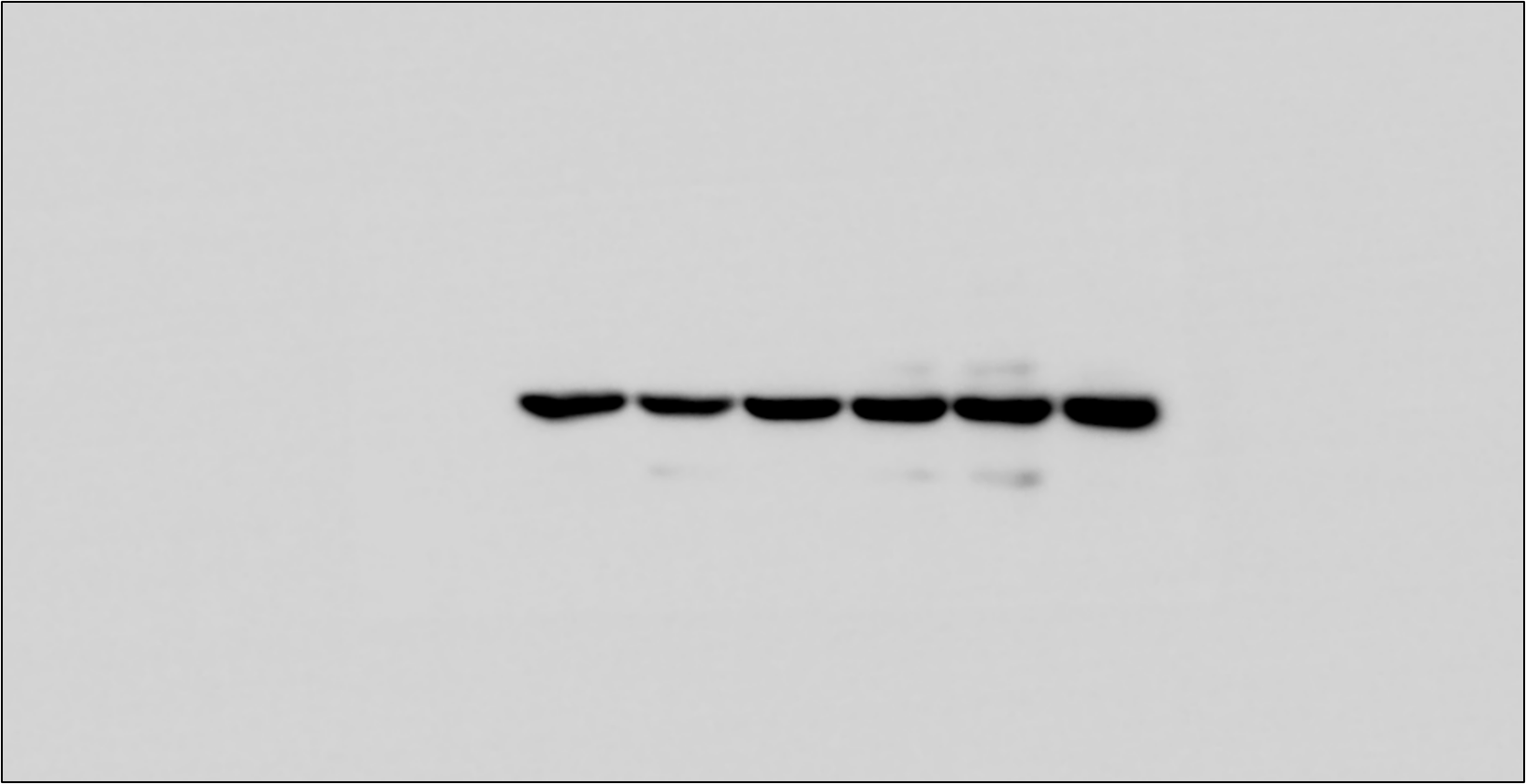

Supplement: Figure 7—source data 2. [file elife-108048-fig7-data2.zip › Figure 7/Figure 7 K-WCL-Myc.tif]

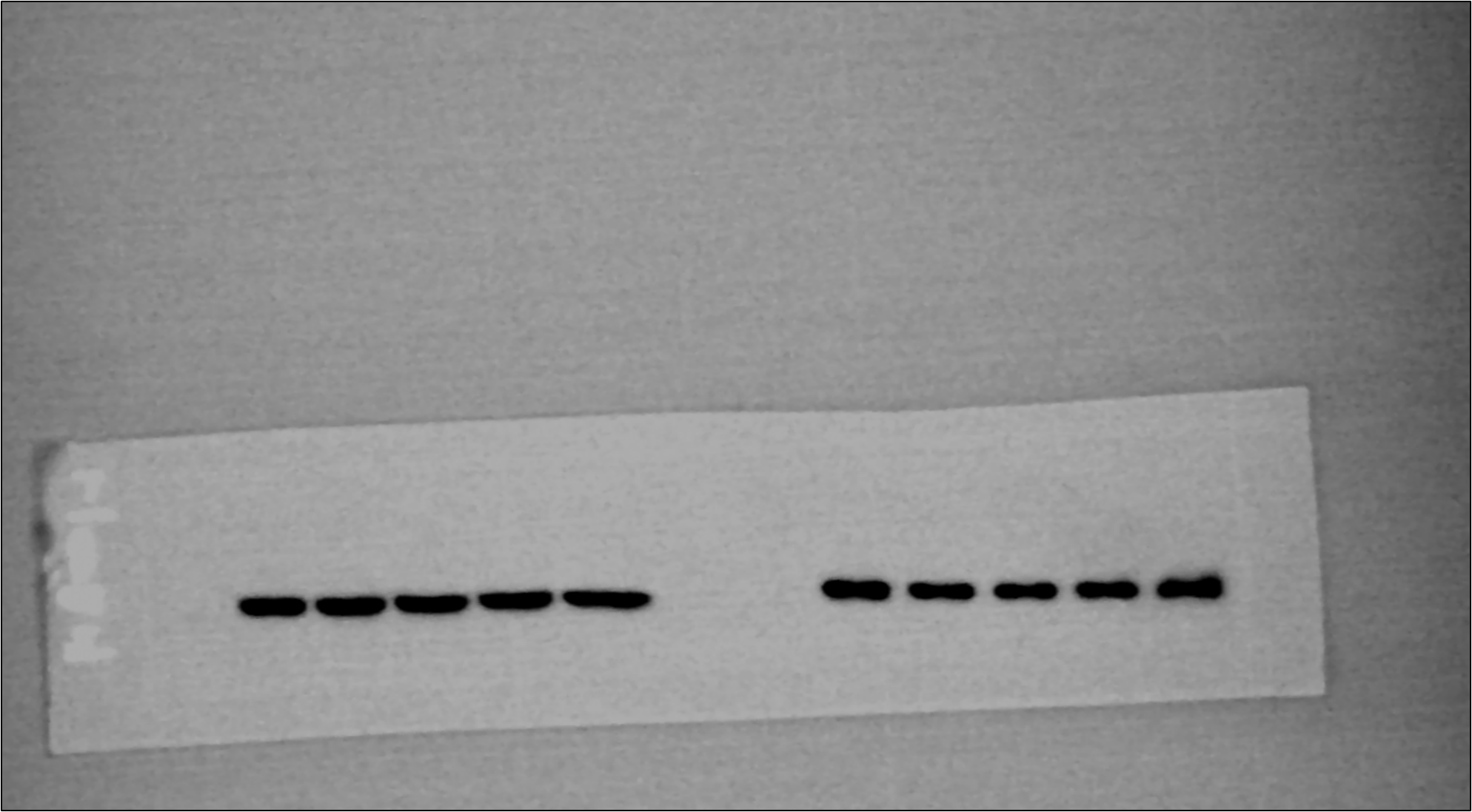

Supplement: Figure 7—source data 2. [file elife-108048-fig7-data2.zip › Figure 7/Figure 7 L-IP-HA.tif]

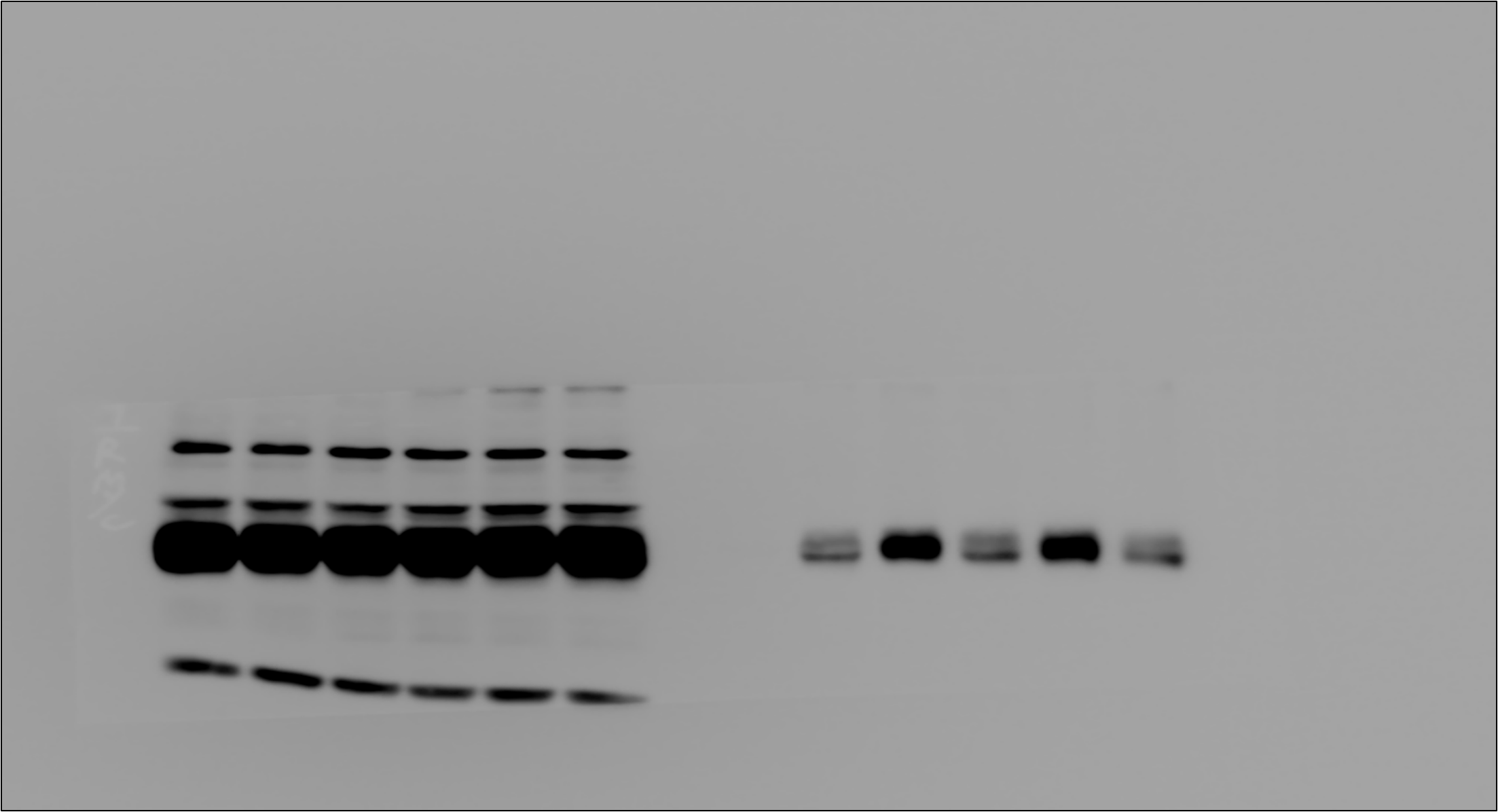

Supplement: Figure 7—source data 2. [file elife-108048-fig7-data2.zip › Figure 7/Figure 7 L-IP-Myc.tif]

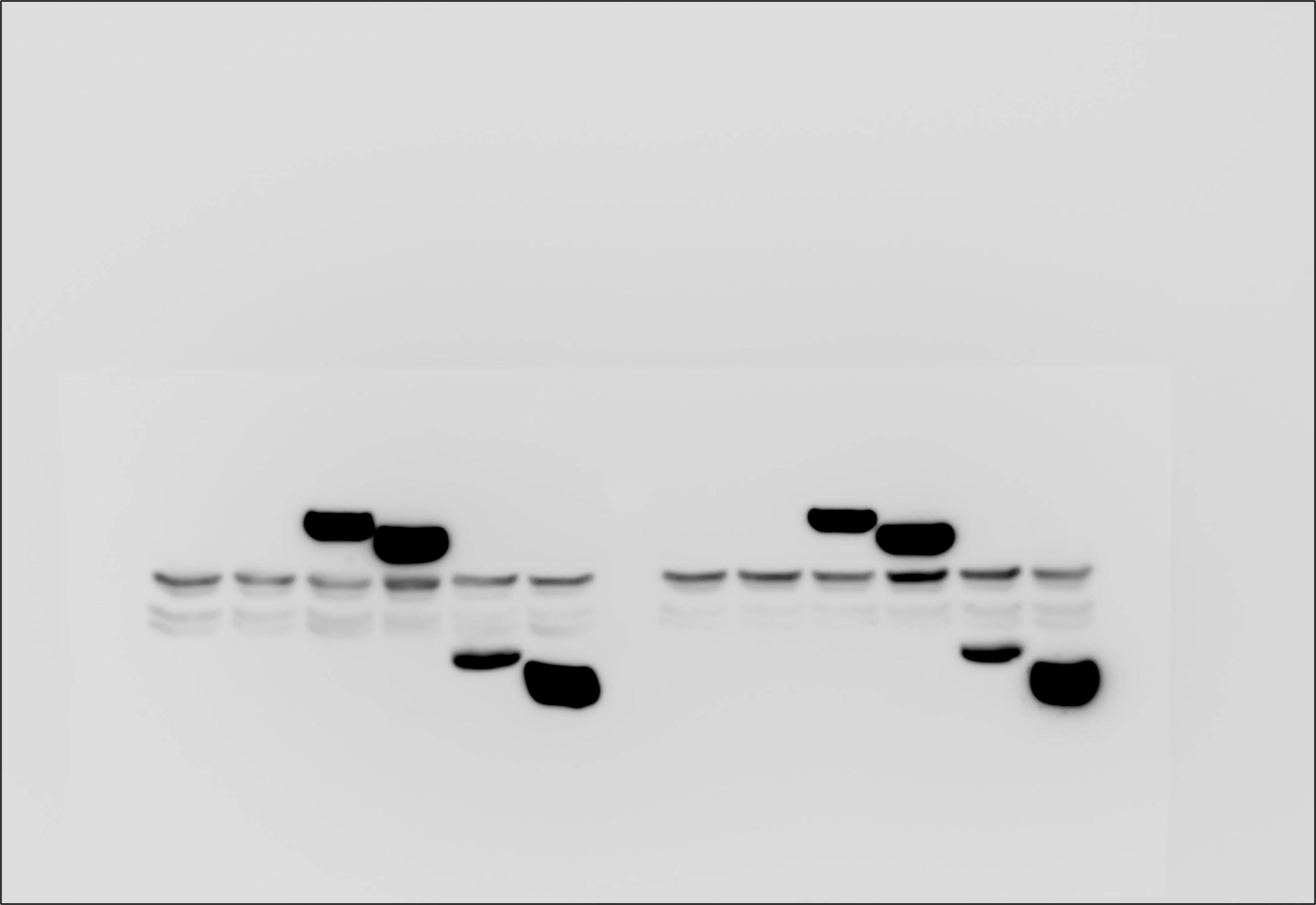

Supplement: Figure 7—source data 2. [file elife-108048-fig7-data2.zip › Figure 7/Figure 7 L-WCL-Flag.tif]

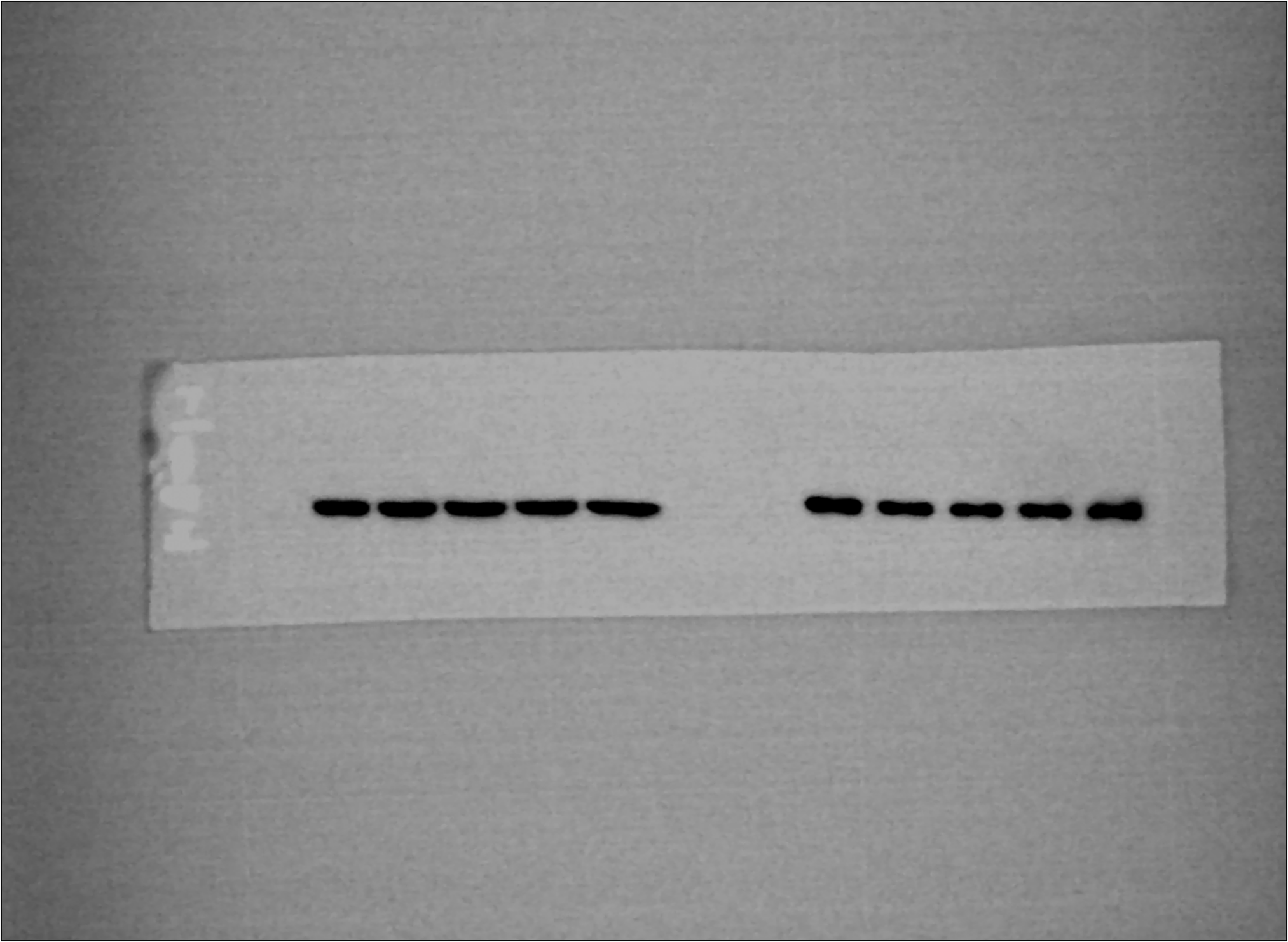

Supplement: Figure 7—source data 2. [file elife-108048-fig7-data2.zip › Figure 7/Figure 7 L-WCL-HA.tif]

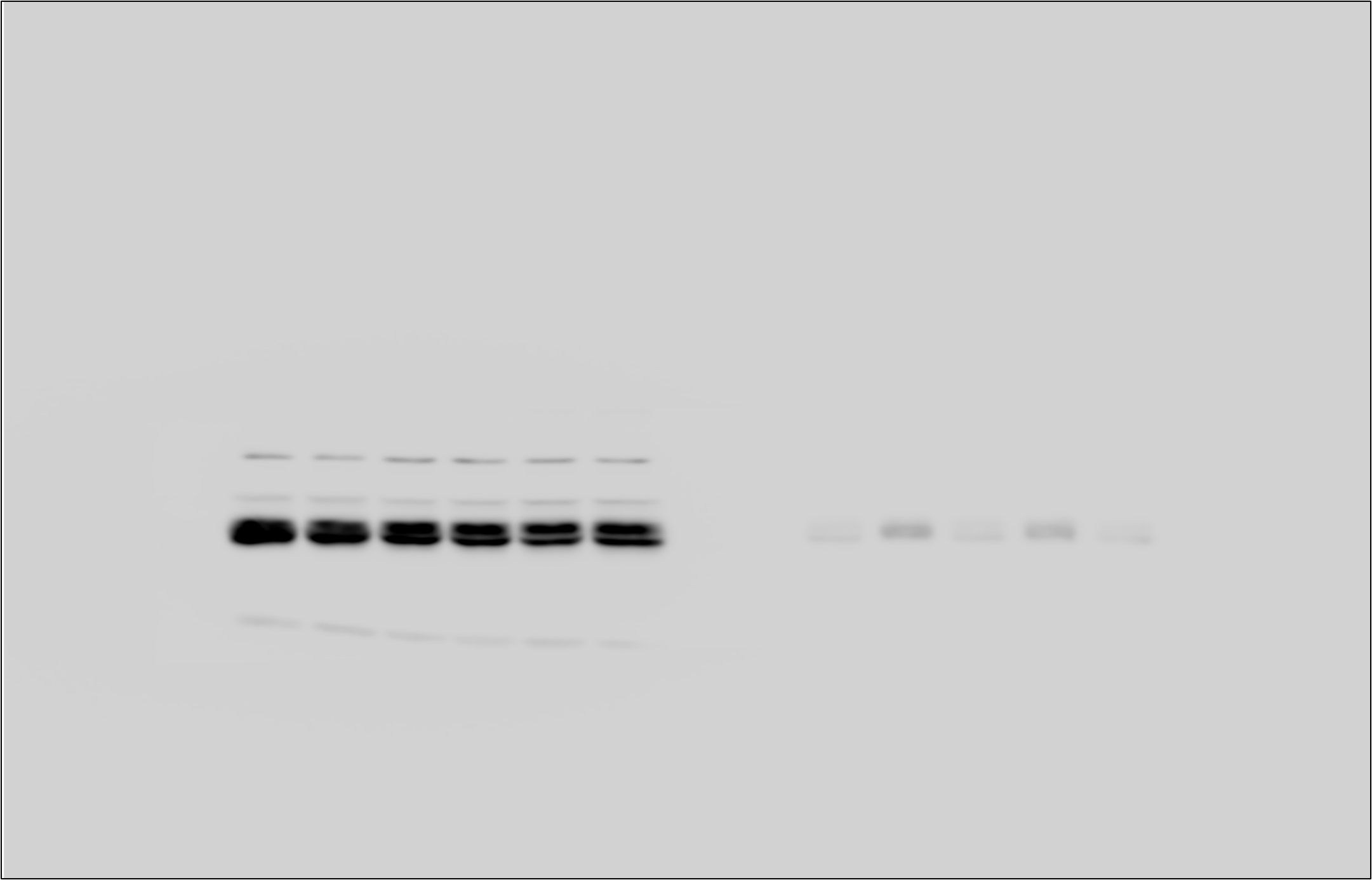

Supplement: Figure 7—source data 2. [file elife-108048-fig7-data2.zip › Figure 7/Figure 7 L-WCL-Myc.tif]

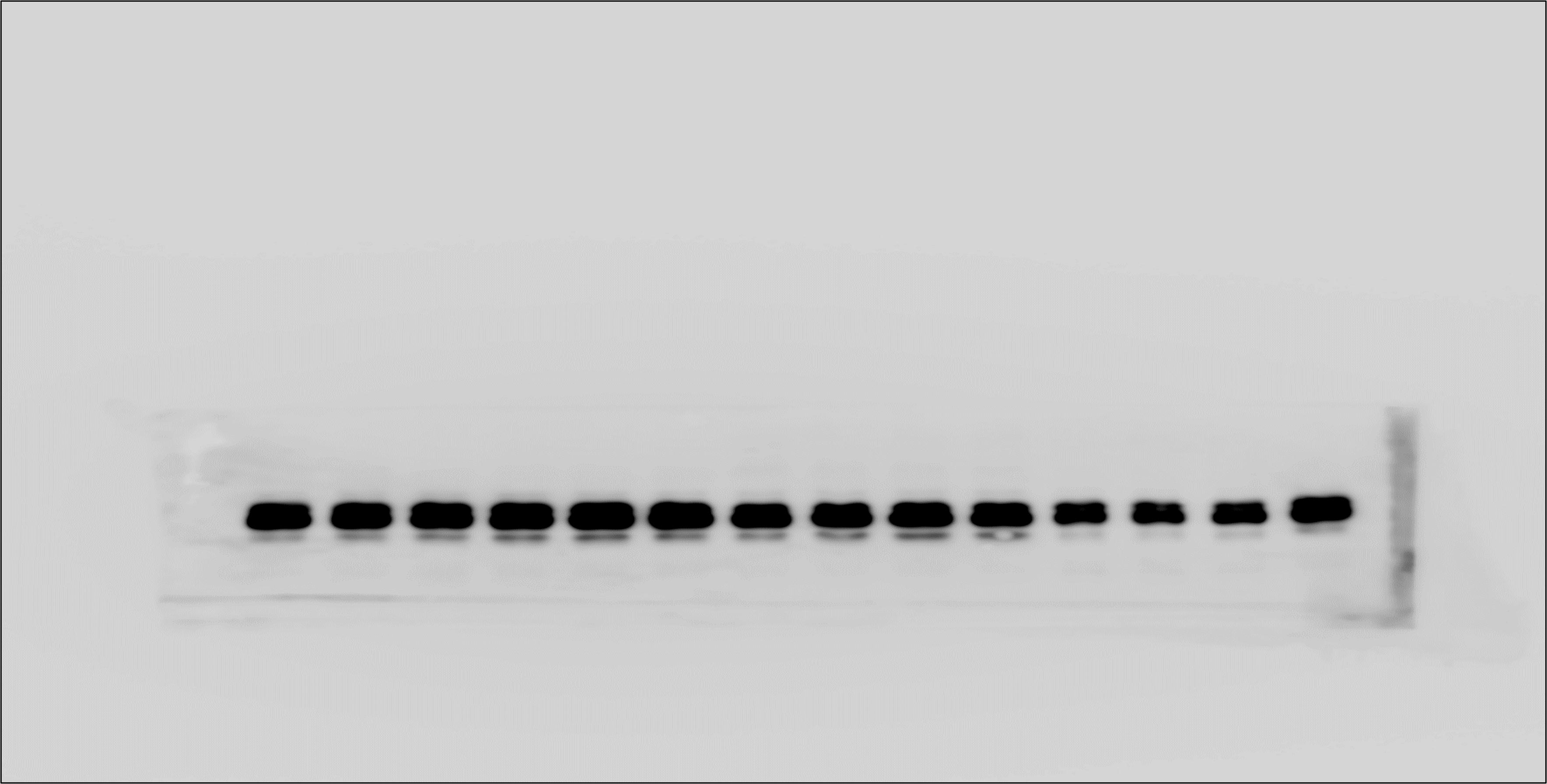

Supplement: Figure 7—source data 2. [file elife-108048-fig7-data2.zip › Figure 7/Figure 7 M-IP-Flag.tif]

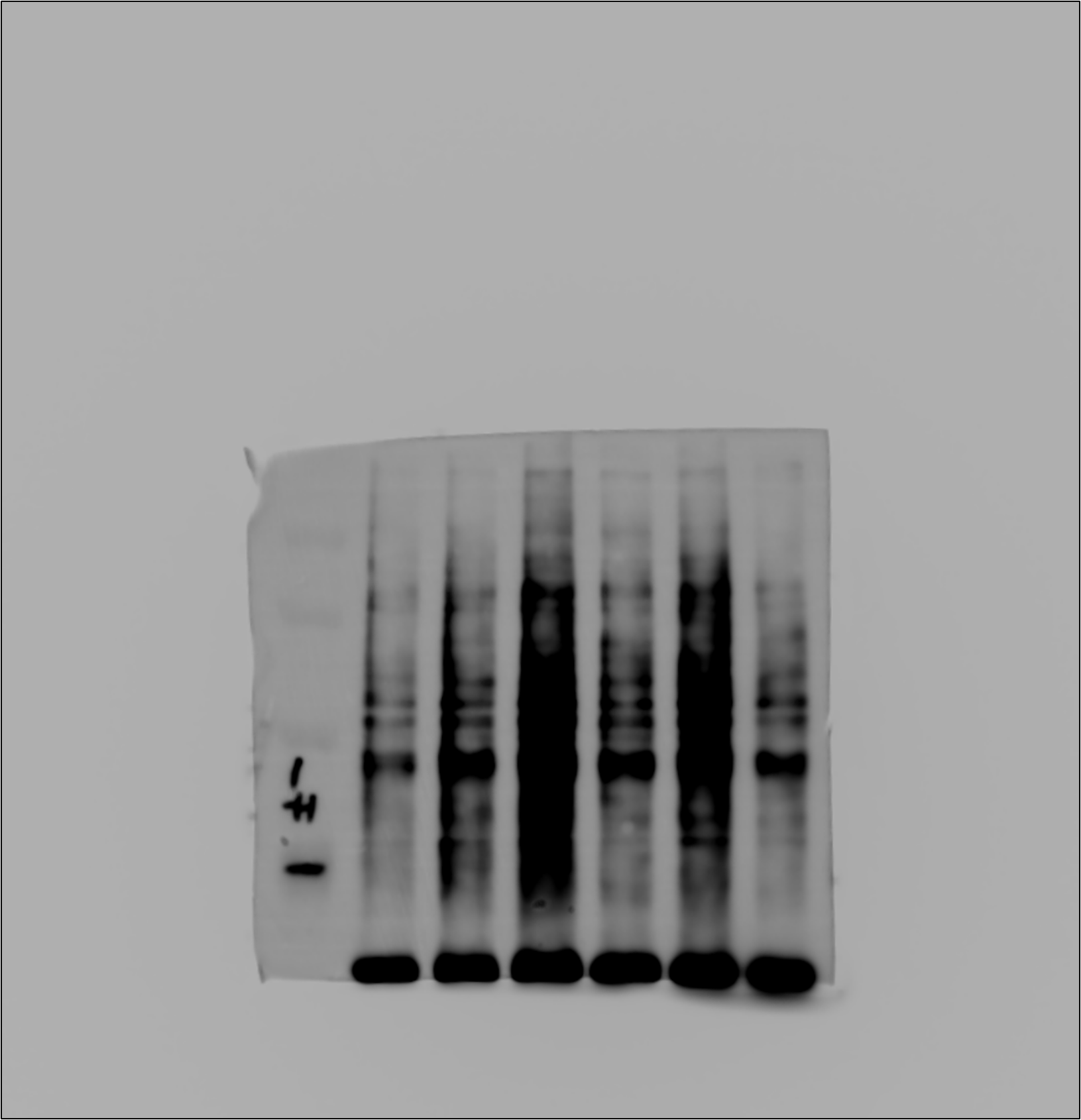

Supplement: Figure 7—source data 2. [file elife-108048-fig7-data2.zip › Figure 7/Figure 7 M-IP-HA.tif]

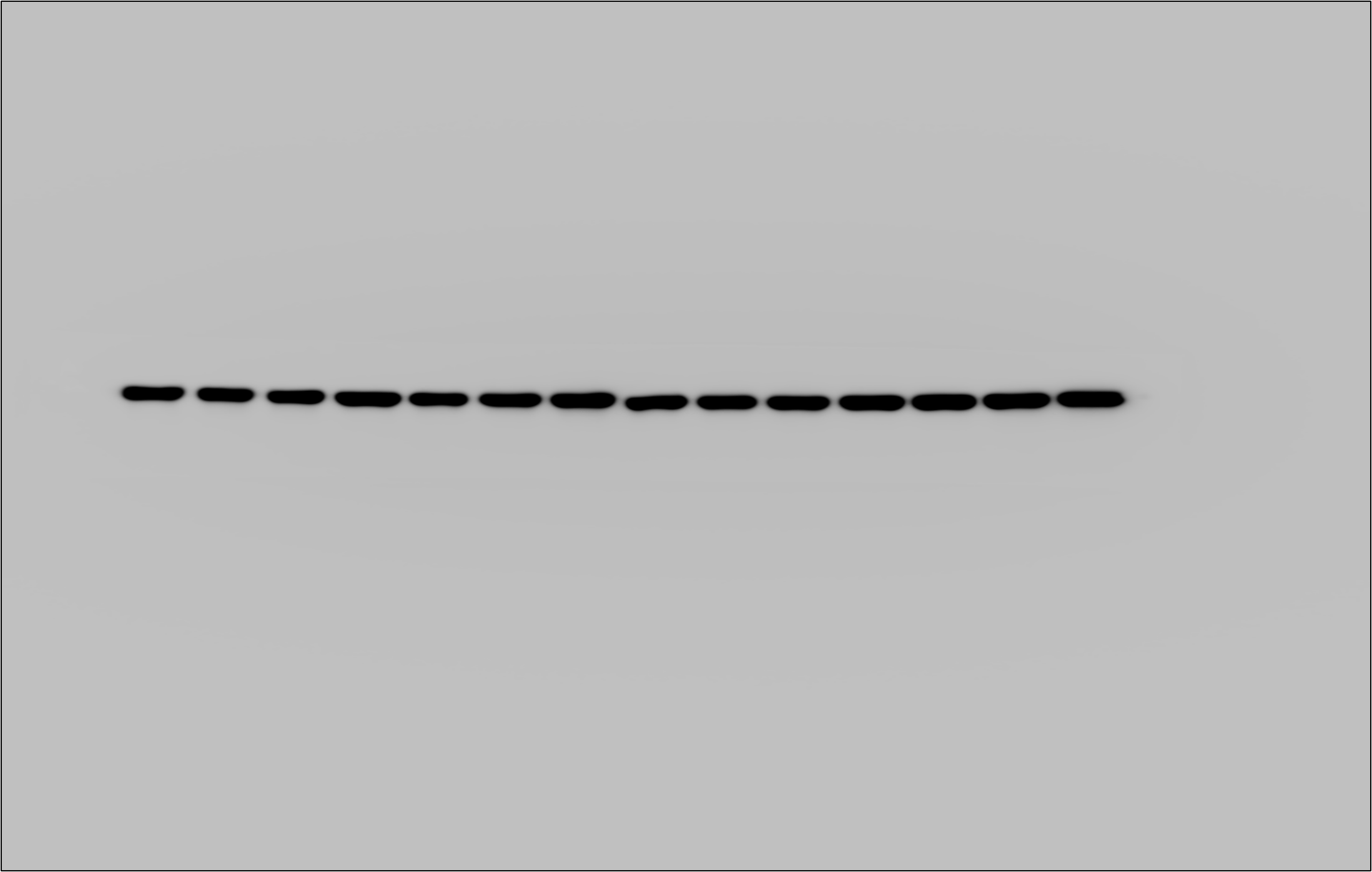

Supplement: Figure 7—source data 2. [file elife-108048-fig7-data2.zip › Figure 7/Figure 7 M-WCL-Actin.tif]

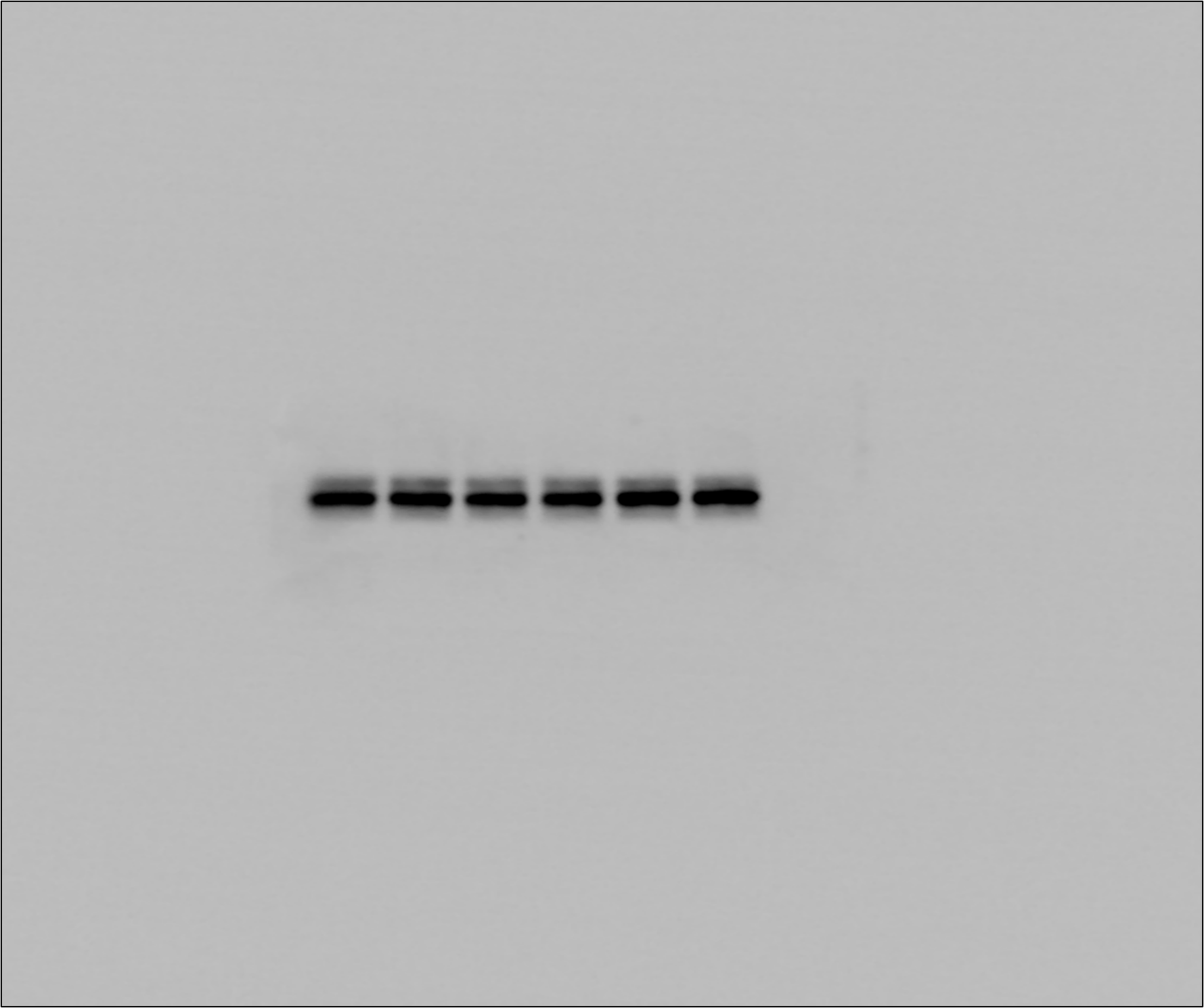

Supplement: Figure 7—source data 2. [file elife-108048-fig7-data2.zip › Figure 7/Figure 7 M-WCL-Flag.tif]

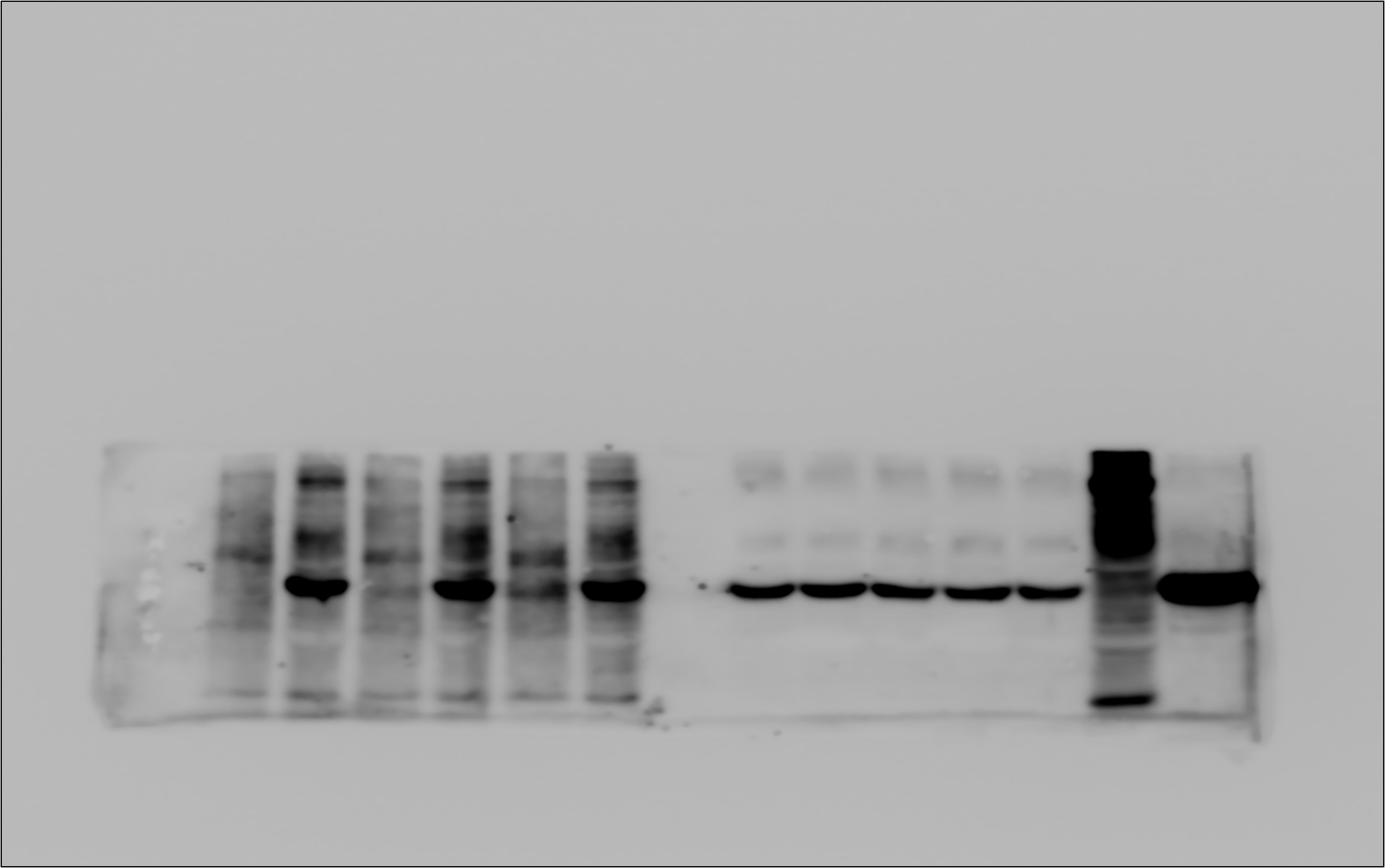

Supplement: Figure 7—source data 2. [file elife-108048-fig7-data2.zip › Figure 7/Figure 7 M-WCL-HA-cyp17a2.tif]

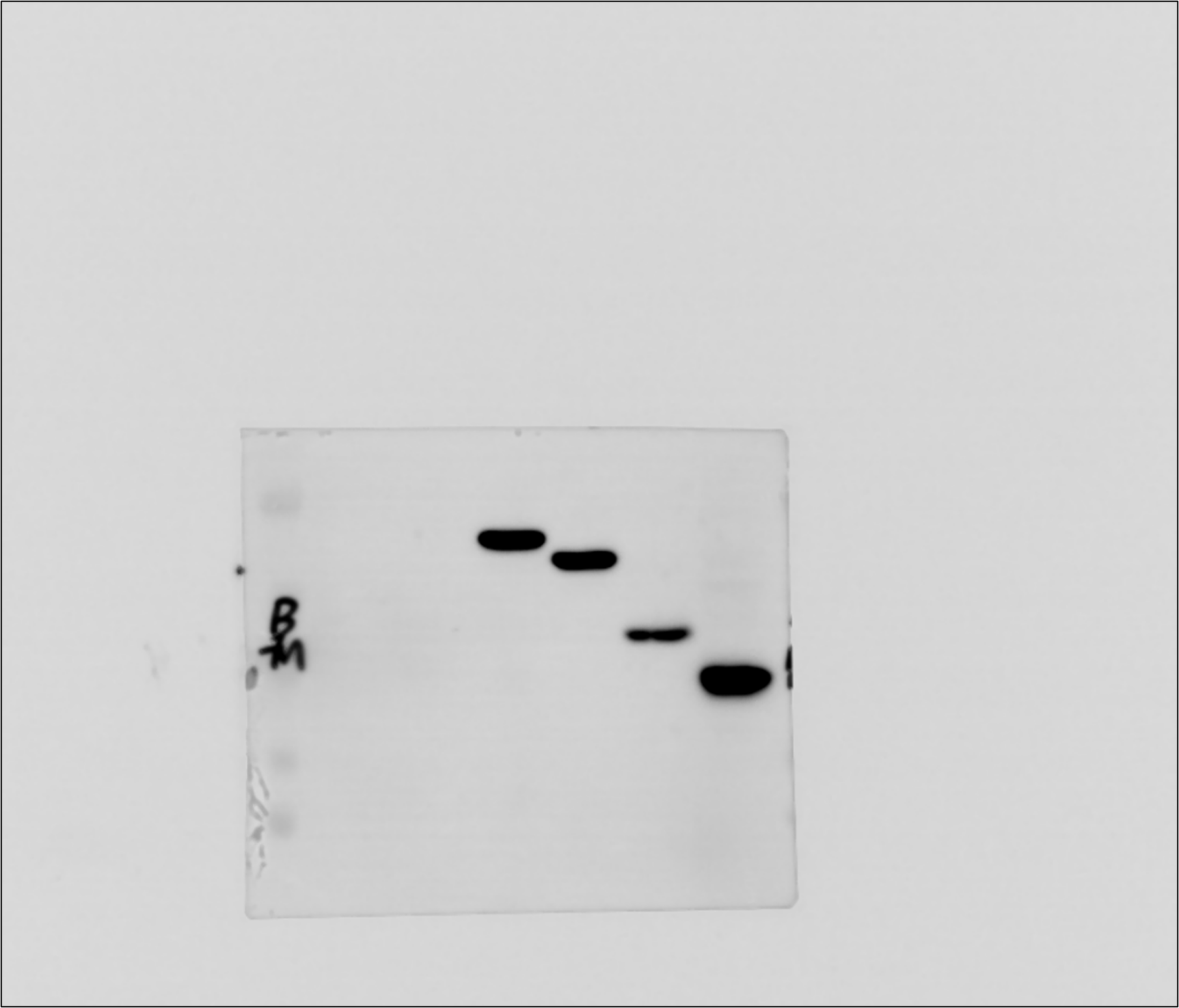

Supplement: Figure 7—source data 2. [file elife-108048-fig7-data2.zip › Figure 7/Figure 7 M-WCL-Myc.tif]

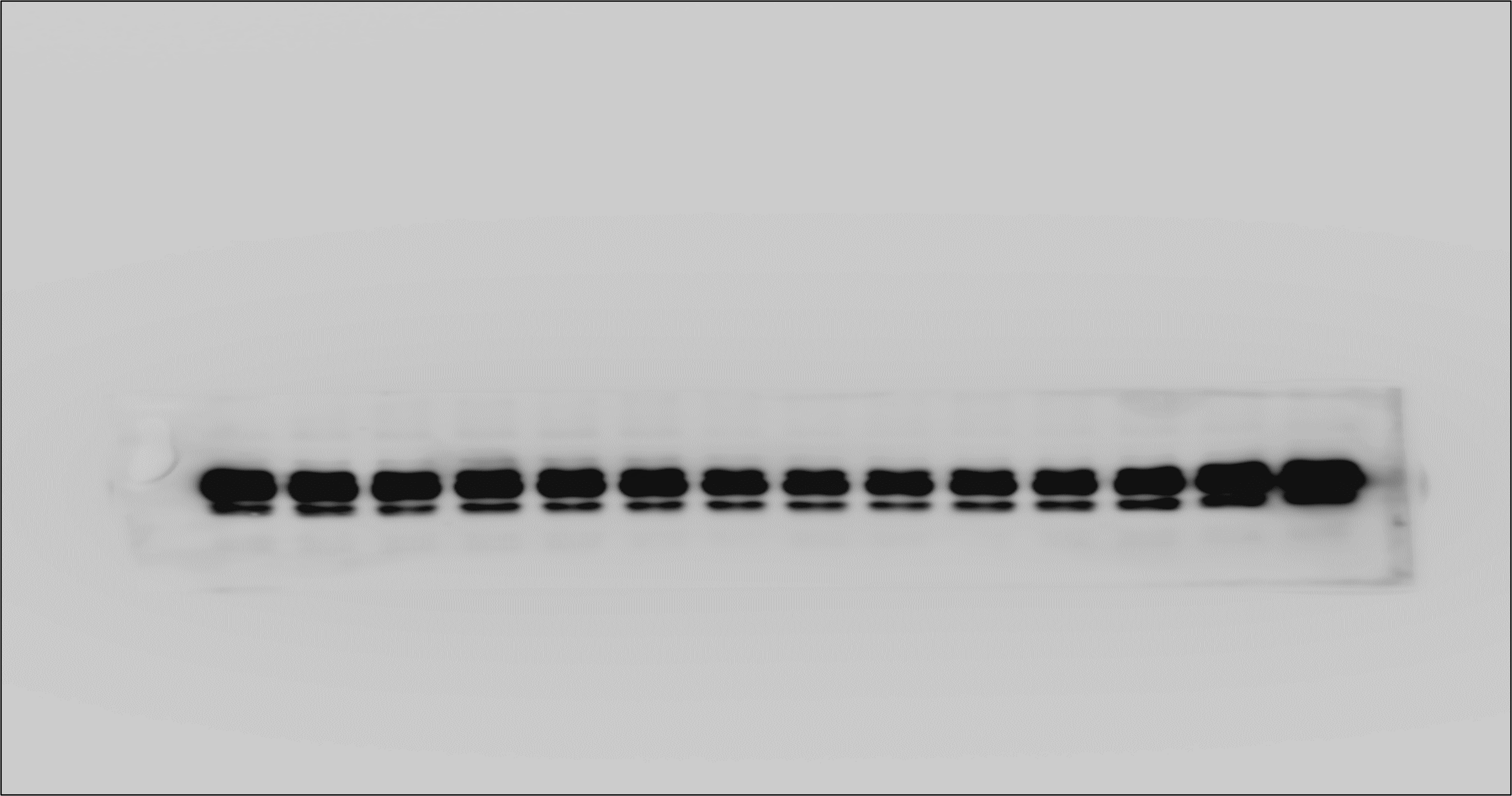

Supplement: Figure 7—figure supplement 1—source data 2. [file elife-108048-fig7-figsupp1-data2.zip › Figure 7-figure supplement 1/Figure S7 A-IP-Flag.tif]

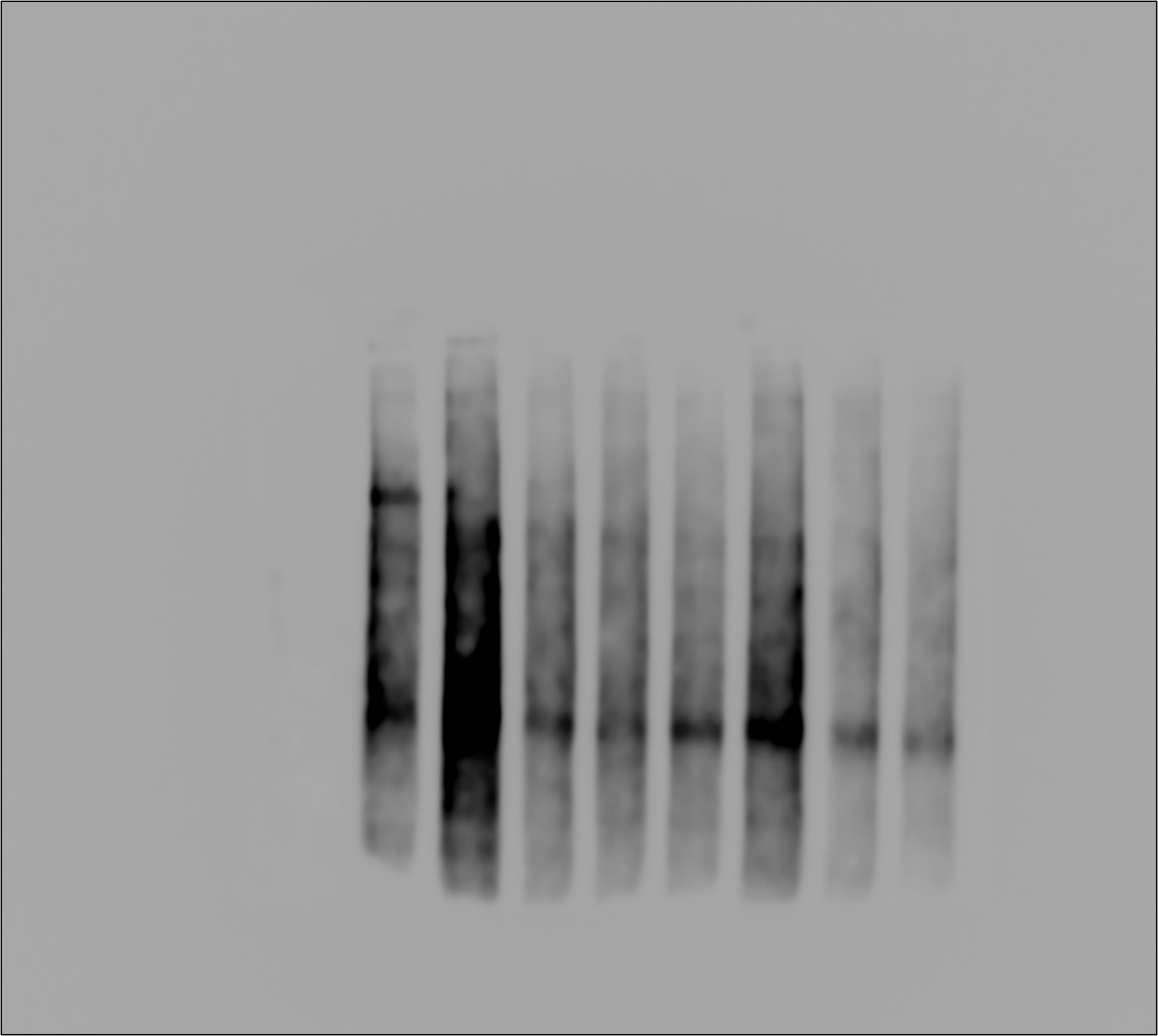

Supplement: Figure 7—figure supplement 1—source data 2. [file elife-108048-fig7-figsupp1-data2.zip › Figure 7-figure supplement 1/Figure S7 A-IP-HA.tif]

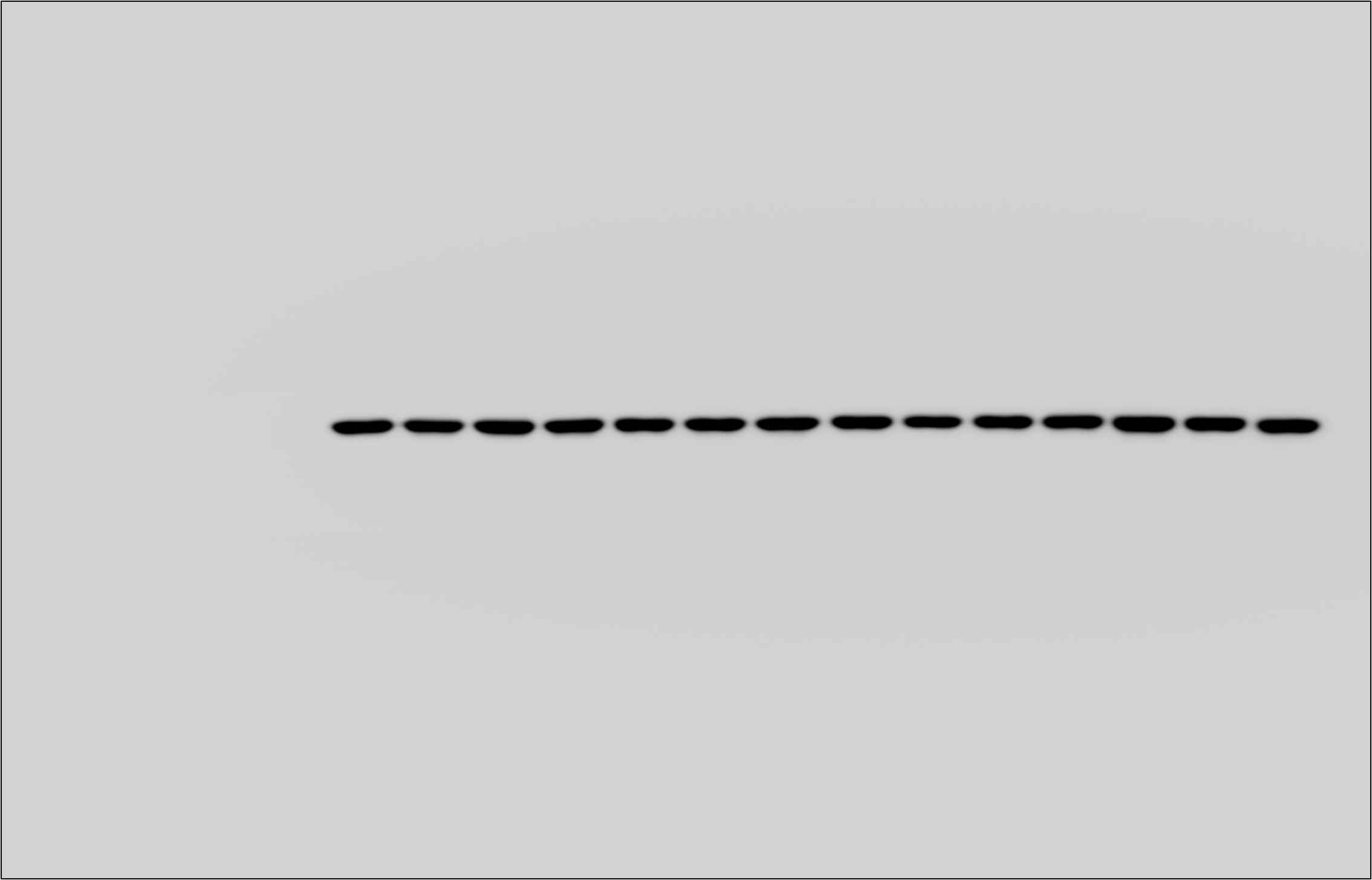

Supplement: Figure 7—figure supplement 1—source data 2. [file elife-108048-fig7-figsupp1-data2.zip › Figure 7-figure supplement 1/Figure S7 A-WCL-Actin.tif]

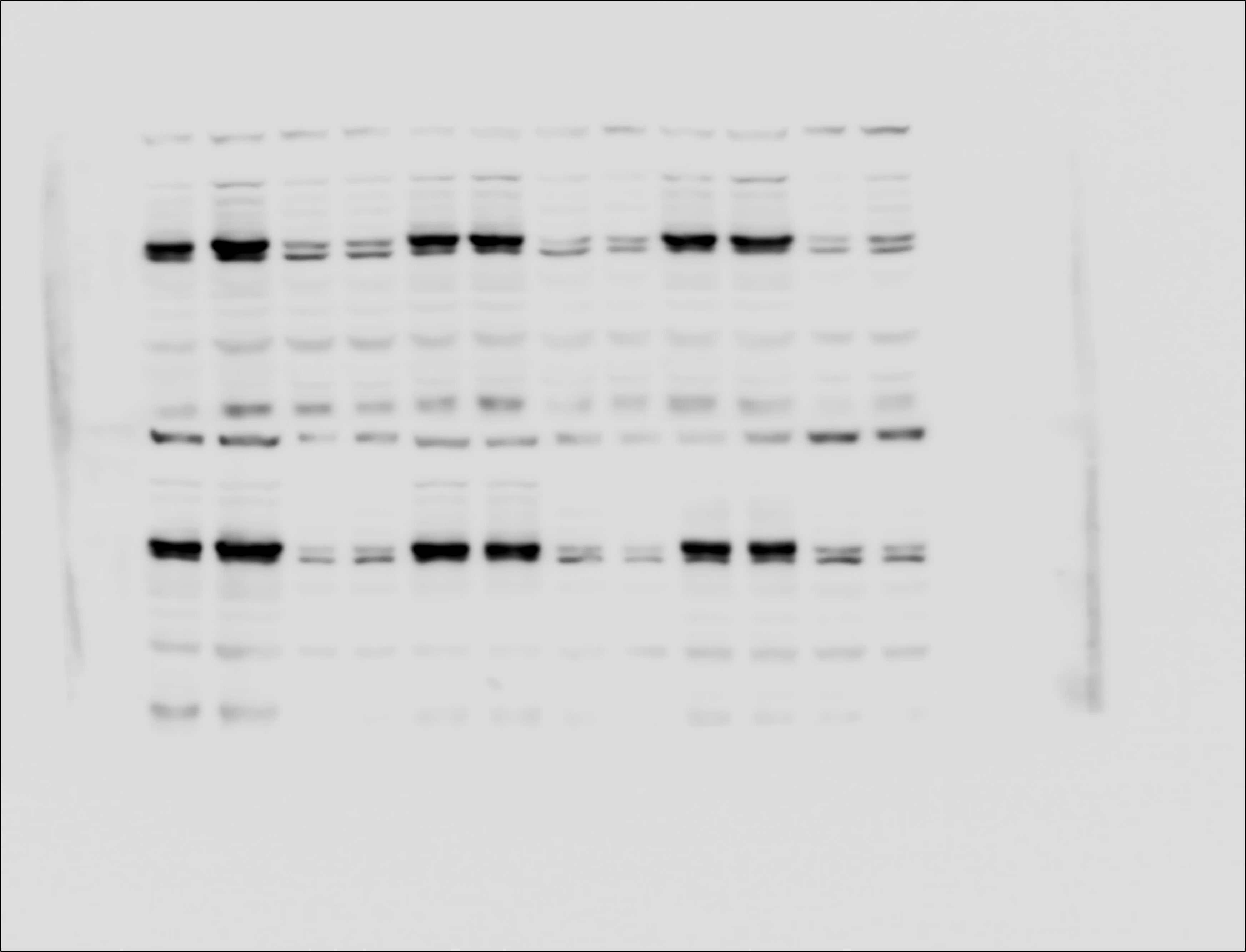

Supplement: Figure 7—figure supplement 1—source data 2. [file elife-108048-fig7-figsupp1-data2.zip › Figure 7-figure supplement 1/Figure S7 A-WCL-btr32.tif]

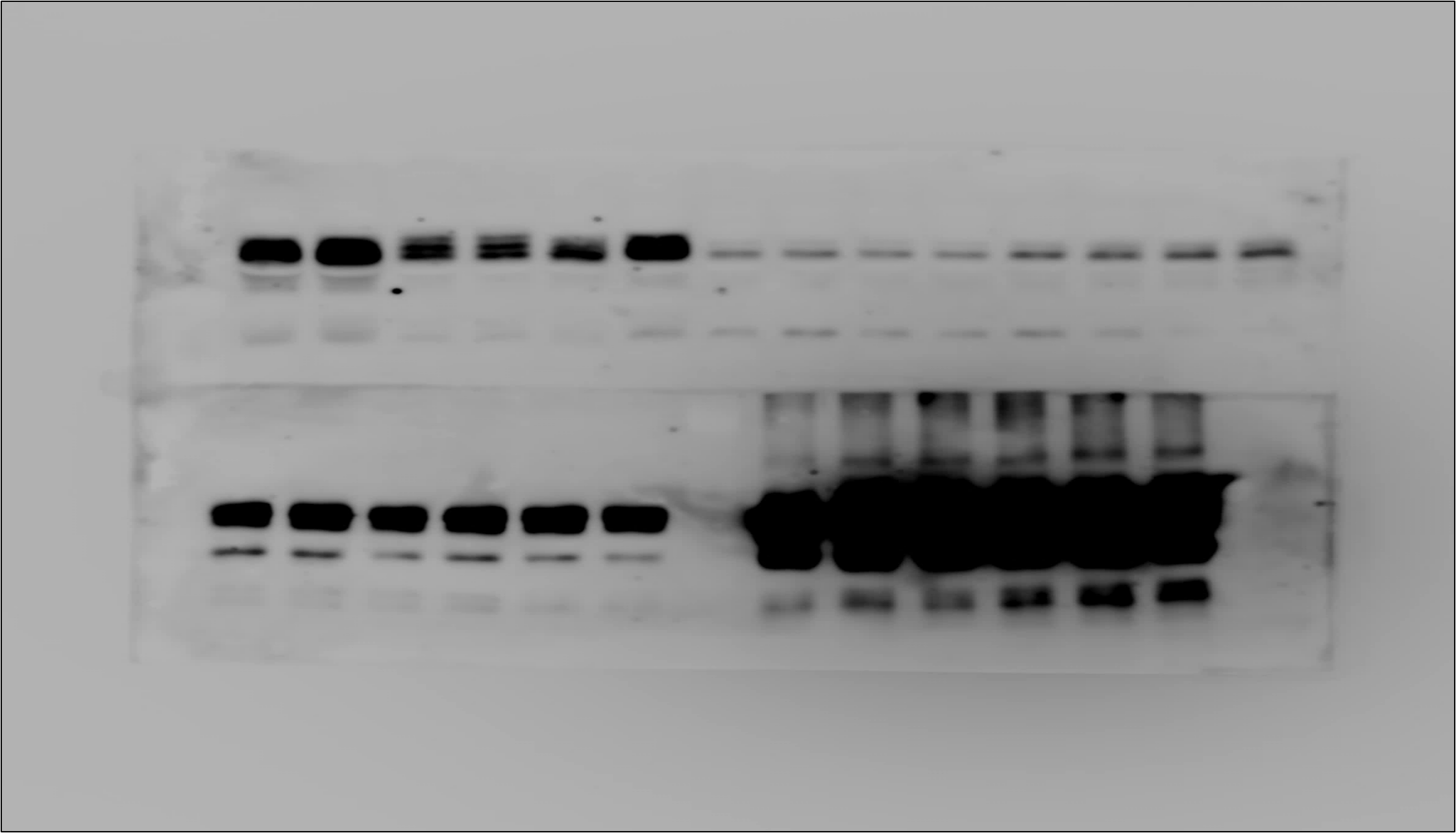

Supplement: Figure 7—figure supplement 1—source data 2. [file elife-108048-fig7-figsupp1-data2.zip › Figure 7-figure supplement 1/Figure S7 A-WCL-Flag.tif]

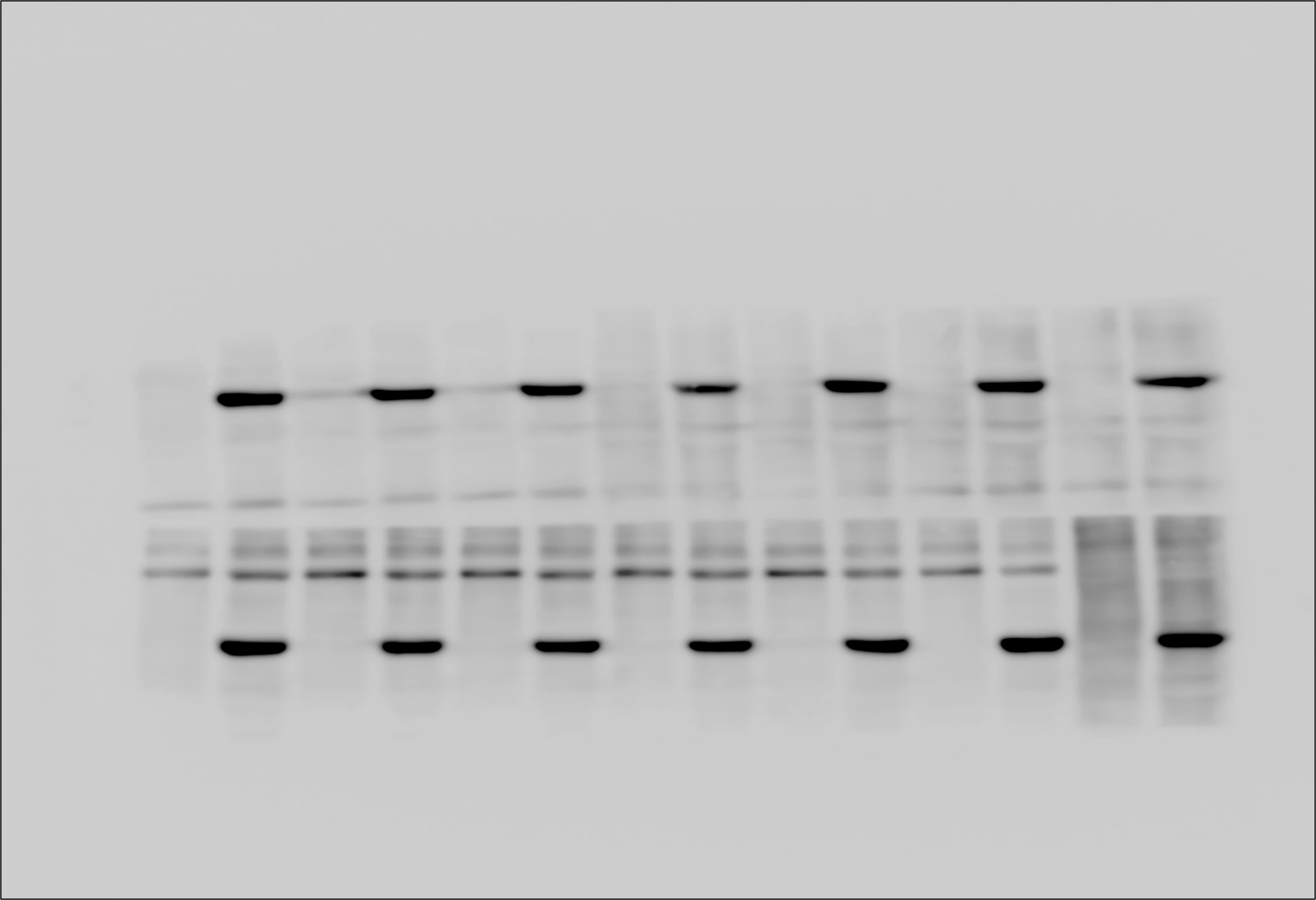

Supplement: Figure 7—figure supplement 1—source data 2. [file elife-108048-fig7-figsupp1-data2.zip › Figure 7-figure supplement 1/Figure S7 A-WCL-HA-cyp17a2.tif]

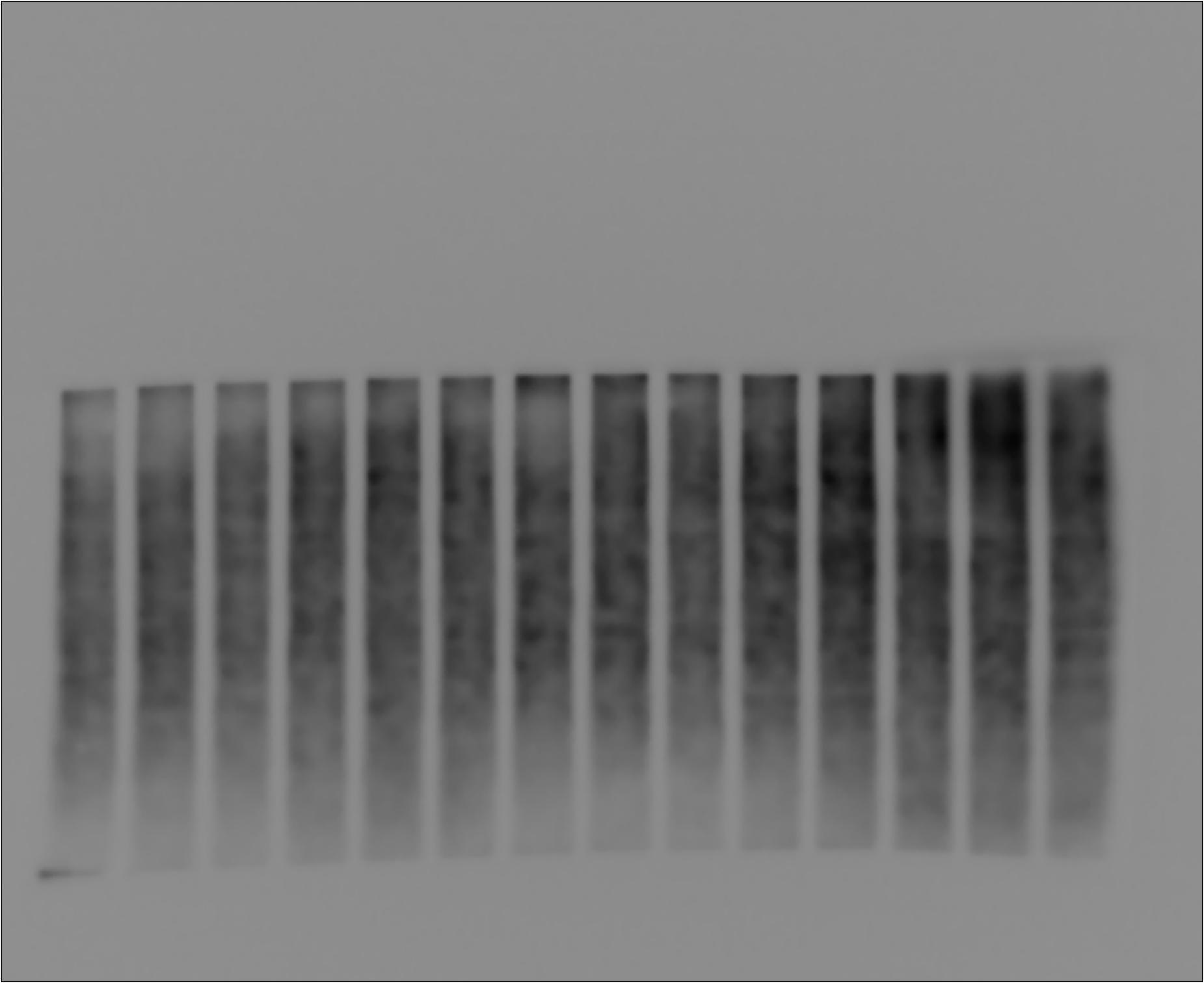

Supplement: Figure 7—figure supplement 1—source data 2. [file elife-108048-fig7-figsupp1-data2.zip › Figure 7-figure supplement 1/Figure S7 A-WCL-HA.tif]

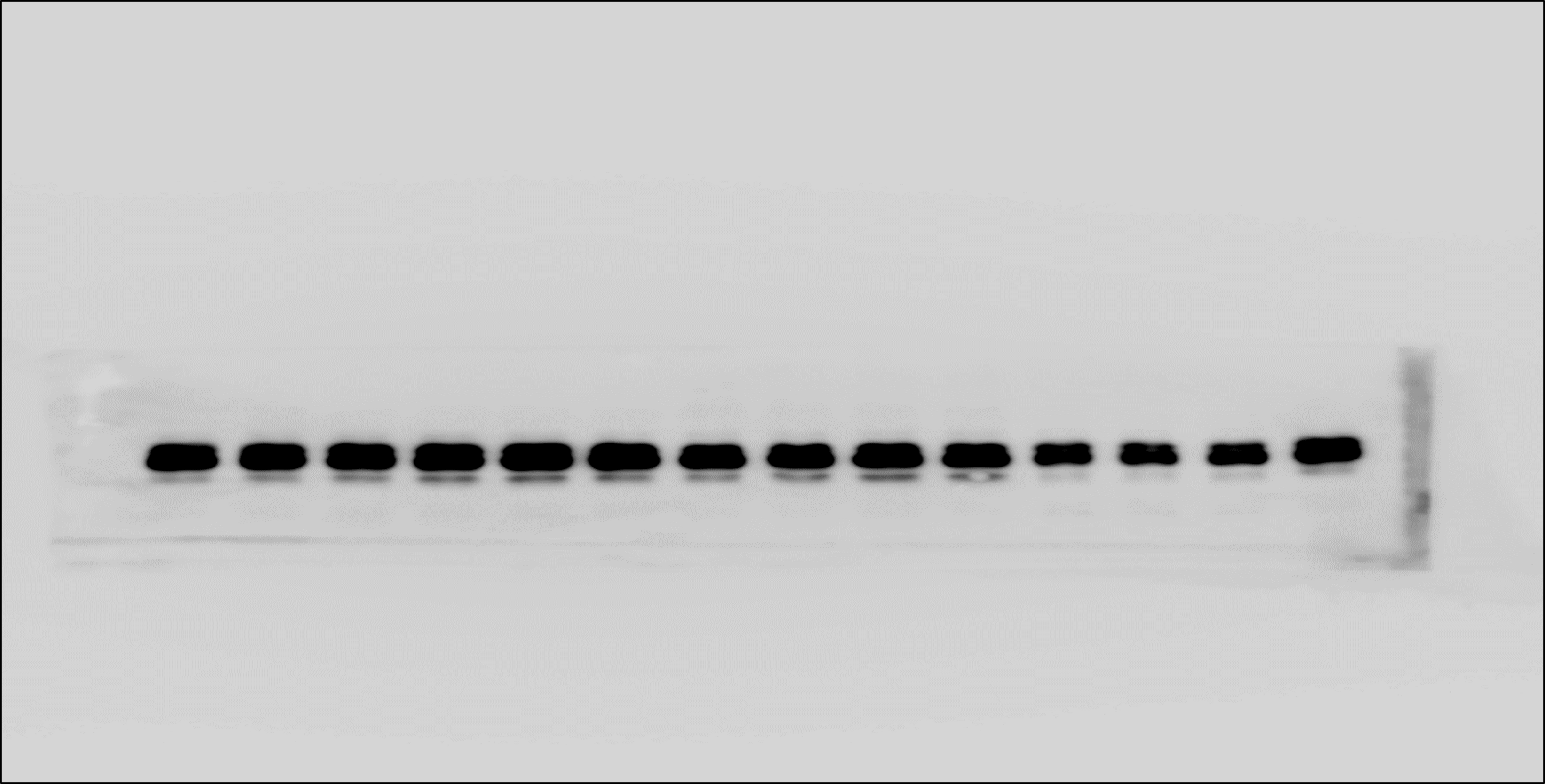

Supplement: Figure 7—figure supplement 1—source data 2. [file elife-108048-fig7-figsupp1-data2.zip › Figure 7-figure supplement 1/Figure S7 B-IP-Flag.tif]

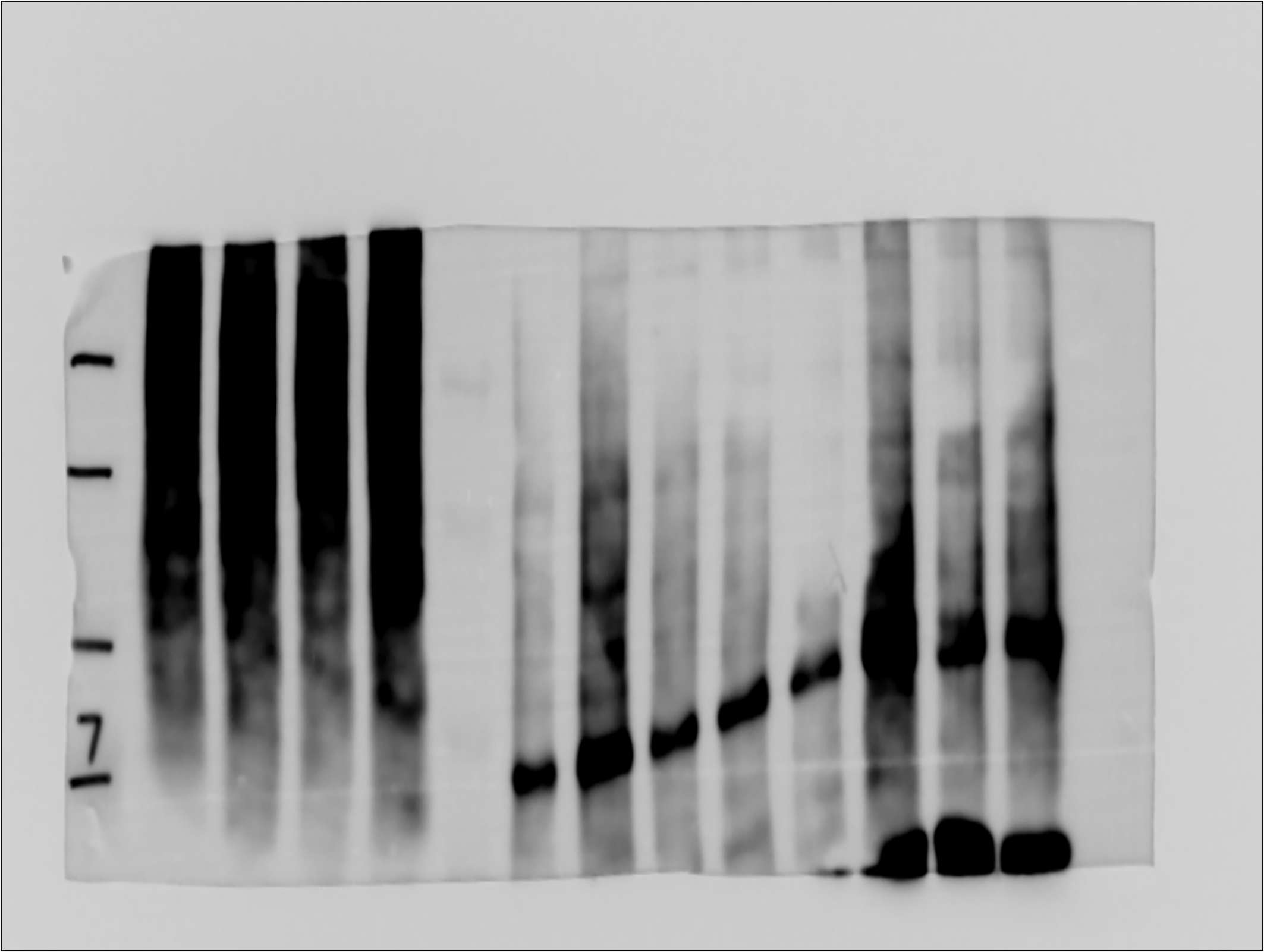

Supplement: Figure 7—figure supplement 1—source data 2. [file elife-108048-fig7-figsupp1-data2.zip › Figure 7-figure supplement 1/Figure S7 B-IP-HA.tif]

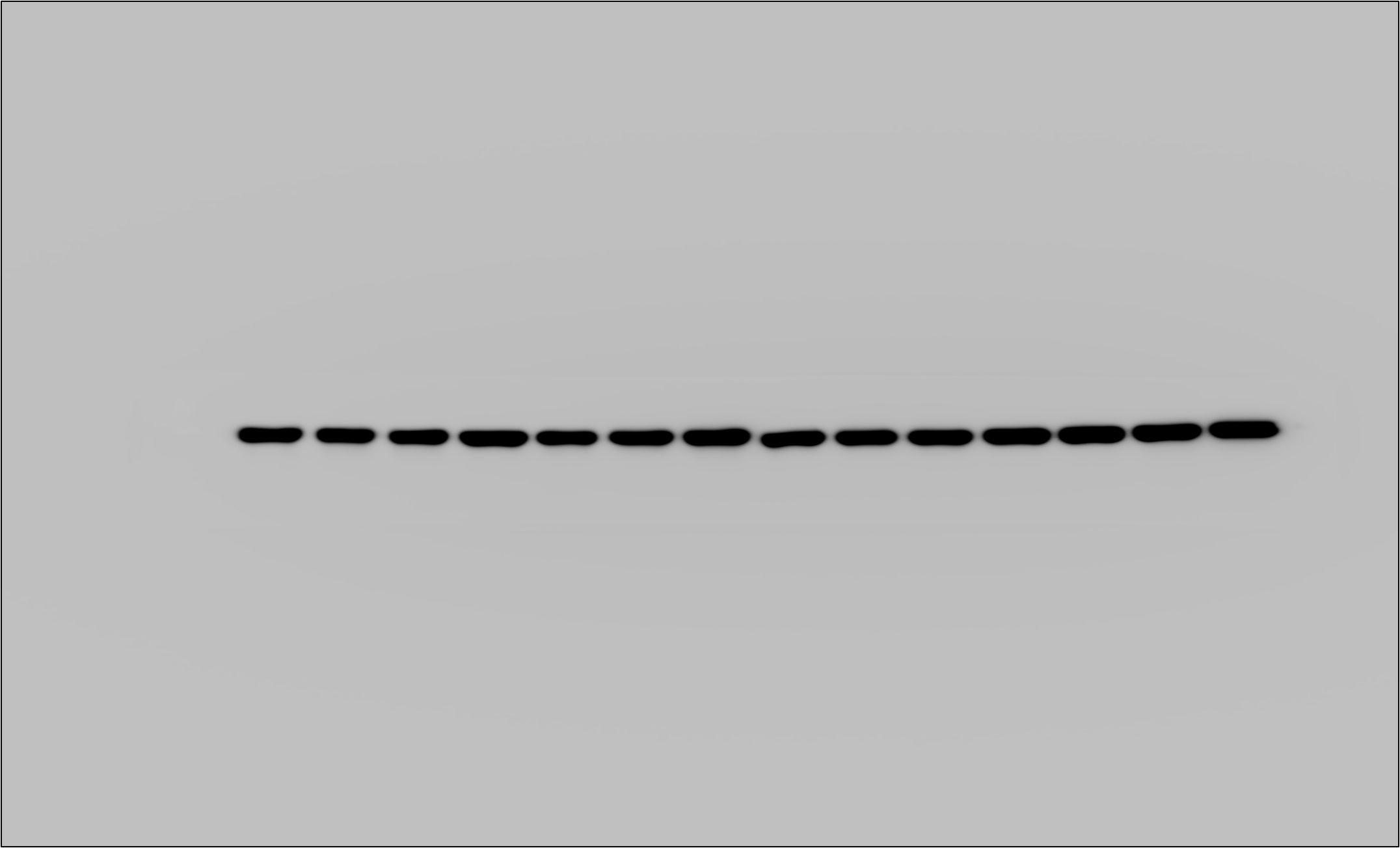

Supplement: Figure 7—figure supplement 1—source data 2. [file elife-108048-fig7-figsupp1-data2.zip › Figure 7-figure supplement 1/Figure S7 B-WCL-Actin.tif]

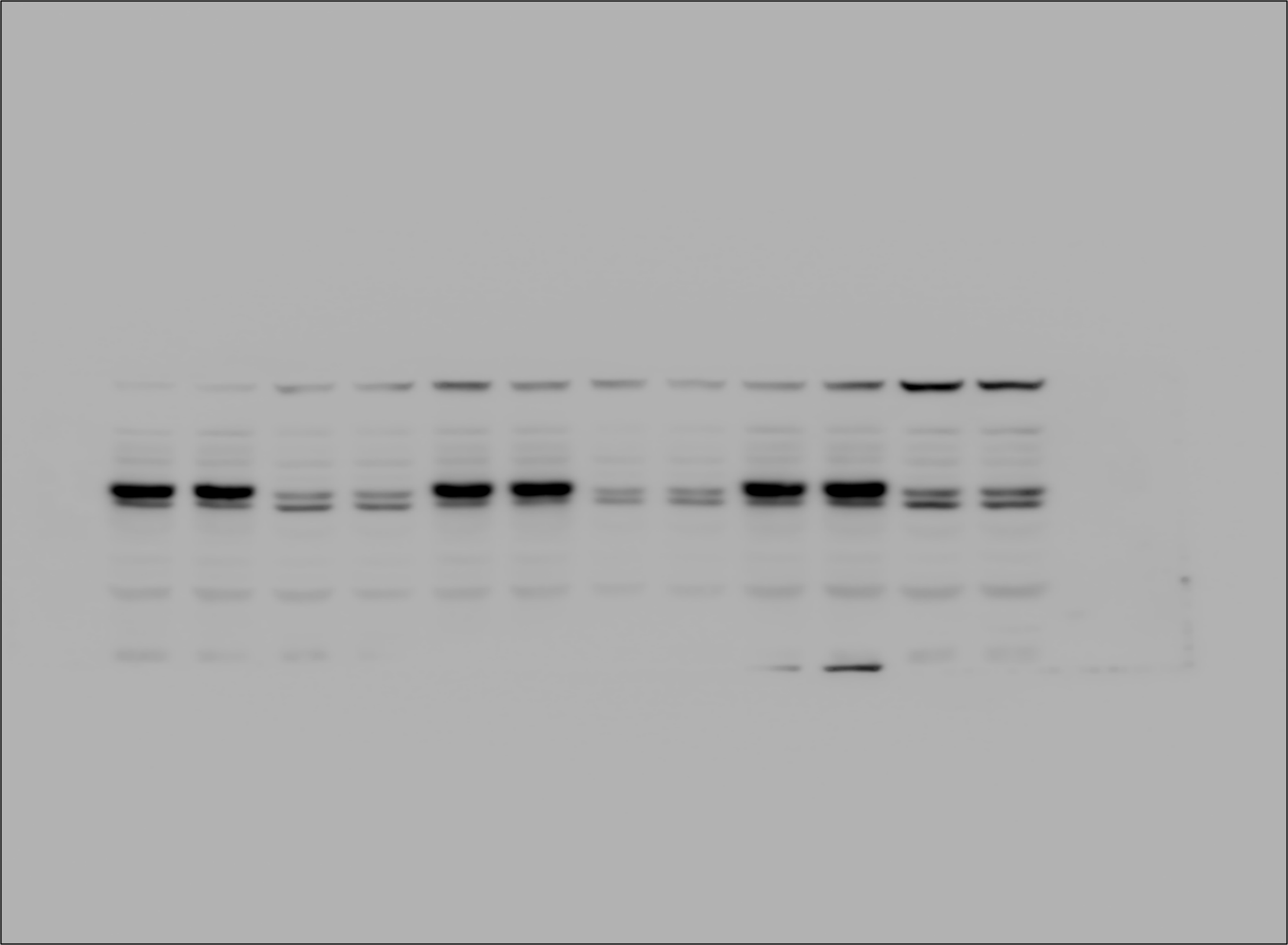

Supplement: Figure 7—figure supplement 1—source data 2. [file elife-108048-fig7-figsupp1-data2.zip › Figure 7-figure supplement 1/Figure S7 B-WCL-btr32.tif]

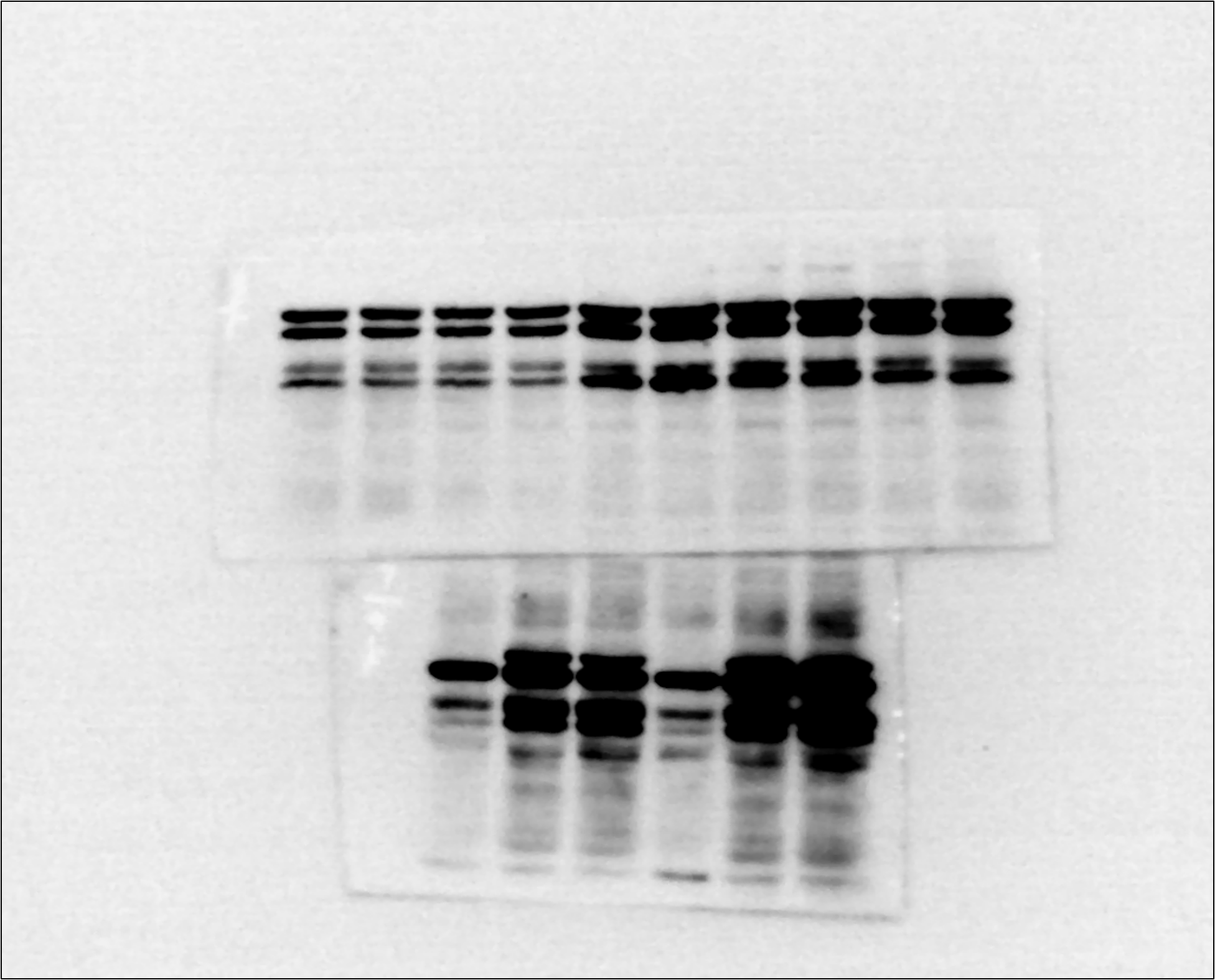

Supplement: Figure 7—figure supplement 1—source data 2. [file elife-108048-fig7-figsupp1-data2.zip › Figure 7-figure supplement 1/Figure S7 B-WCL-Flag.tif]

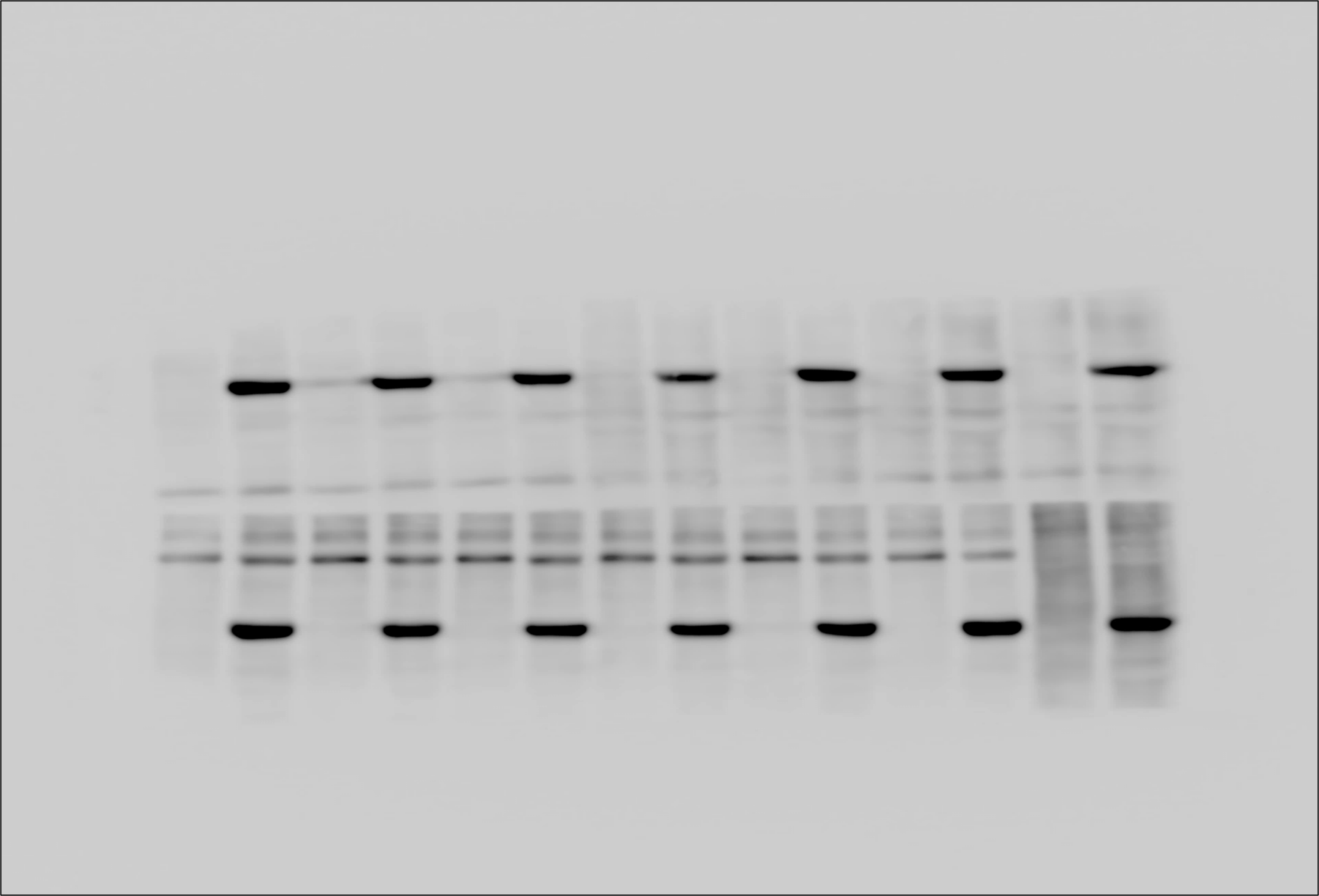

Supplement: Figure 7—figure supplement 1—source data 2. [file elife-108048-fig7-figsupp1-data2.zip › Figure 7-figure supplement 1/Figure S7 B-WCL-HA-cyp17a2.tif]

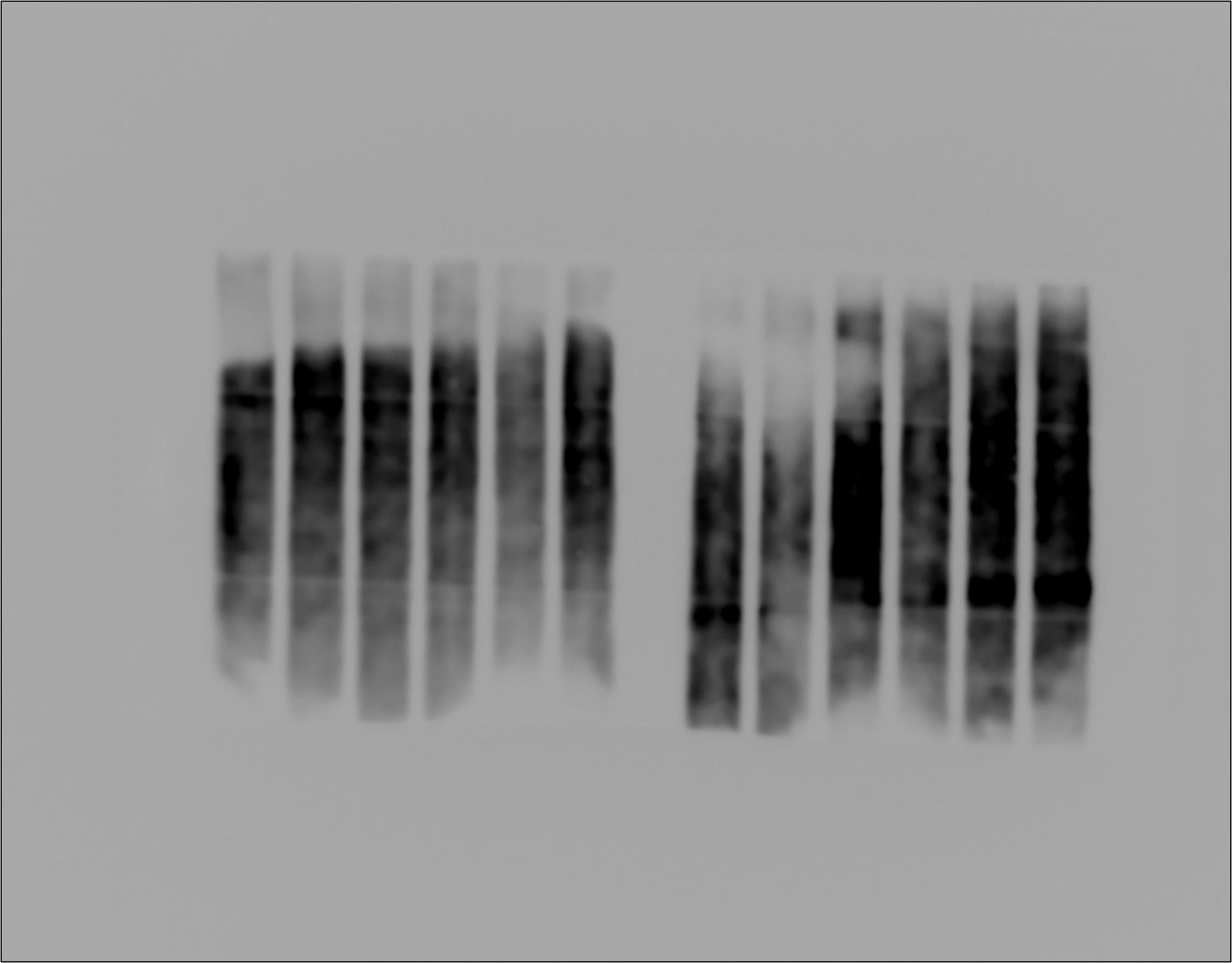

Supplement: Figure 7—figure supplement 1—source data 2. [file elife-108048-fig7-figsupp1-data2.zip › Figure 7-figure supplement 1/Figure S7 B-WCL-HA.tif]

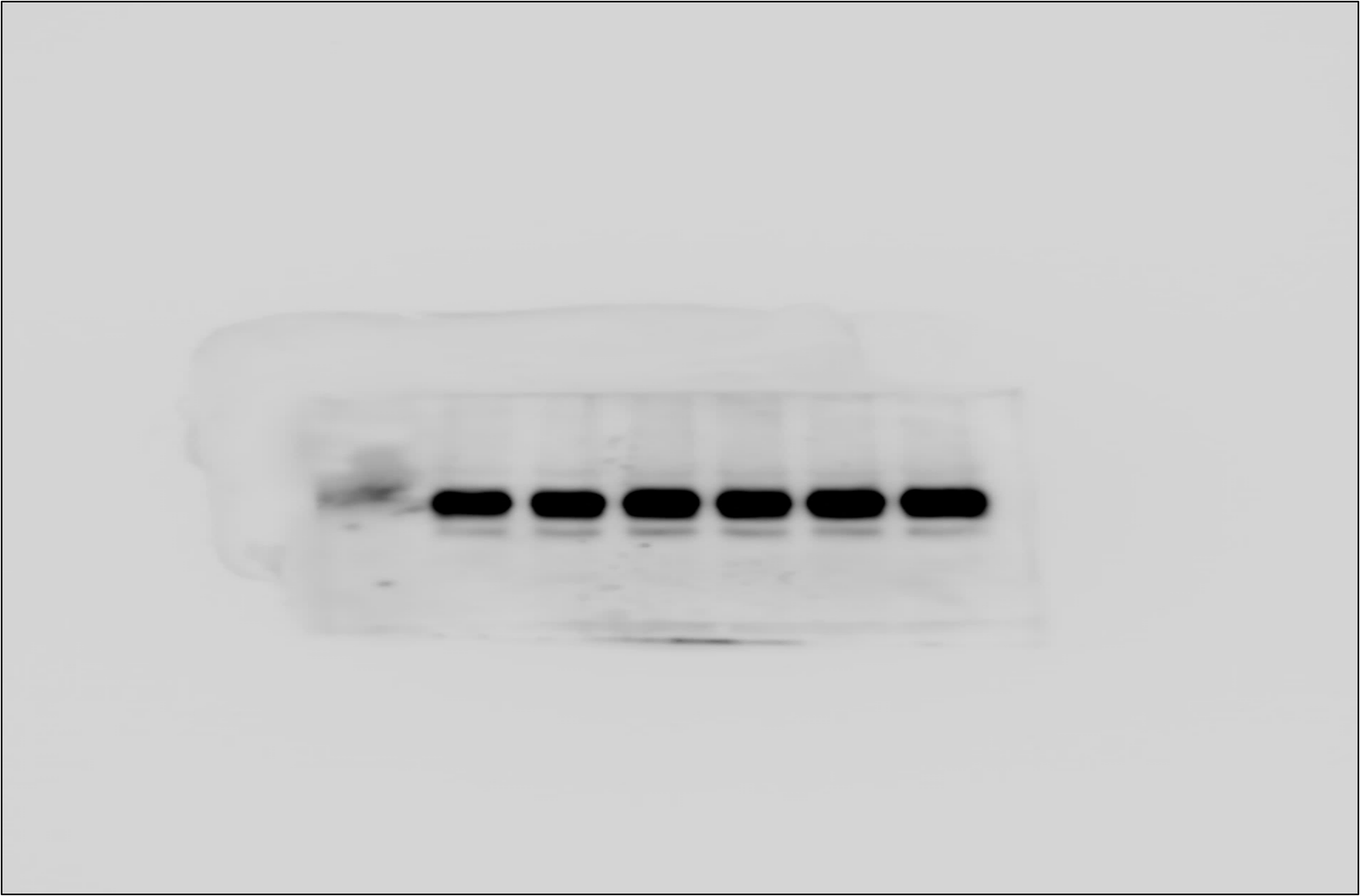

Supplement: Figure 7—figure supplement 1—source data 2. [file elife-108048-fig7-figsupp1-data2.zip › Figure 7-figure supplement 1/Figure S7 C-IP-Flag.tif]

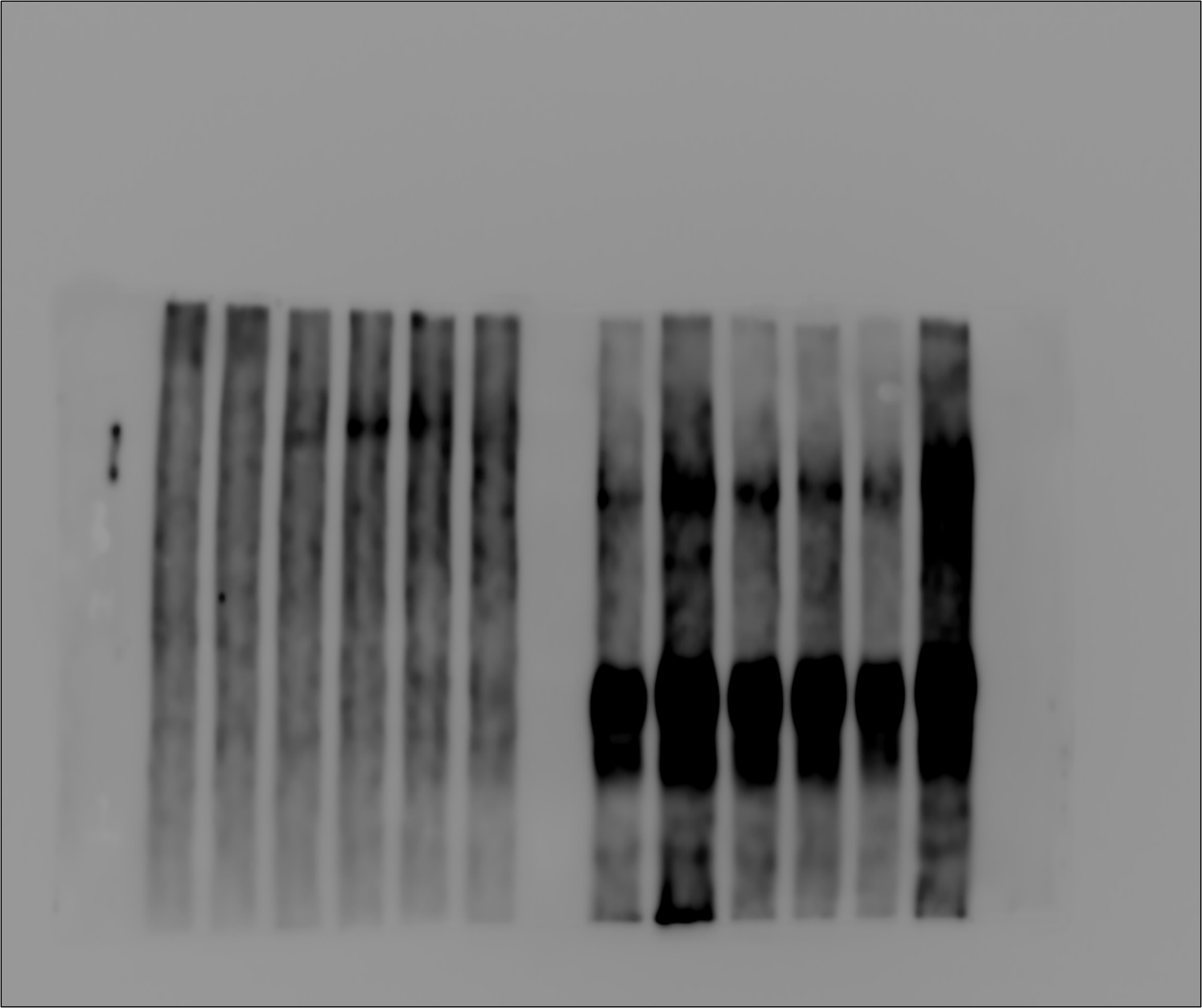

Supplement: Figure 7—figure supplement 1—source data 2. [file elife-108048-fig7-figsupp1-data2.zip › Figure 7-figure supplement 1/Figure S7 C-IP-HA.tif]

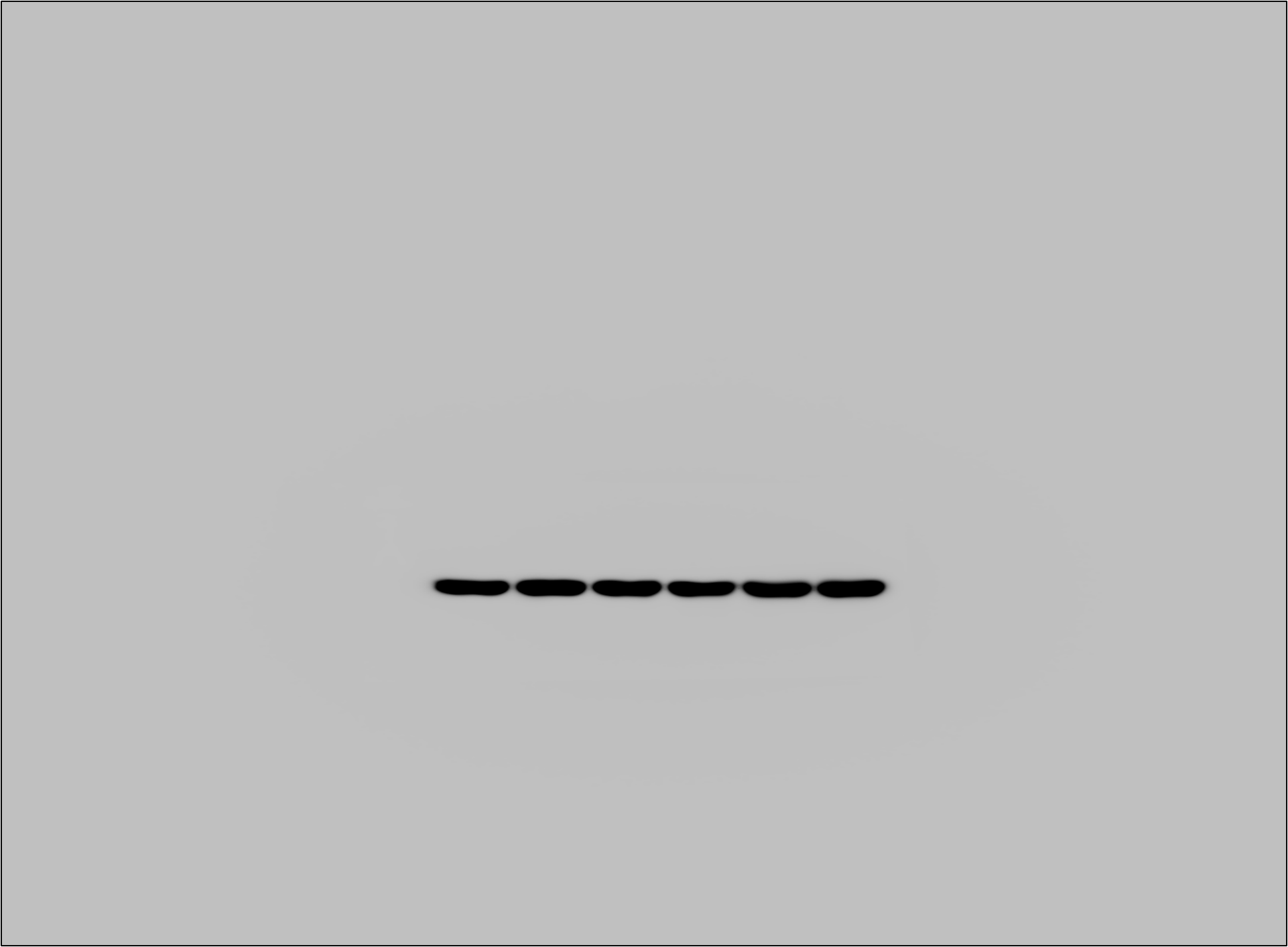

Supplement: Figure 7—figure supplement 1—source data 2. [file elife-108048-fig7-figsupp1-data2.zip › Figure 7-figure supplement 1/Figure S7 C-WCL-Actin.tif]

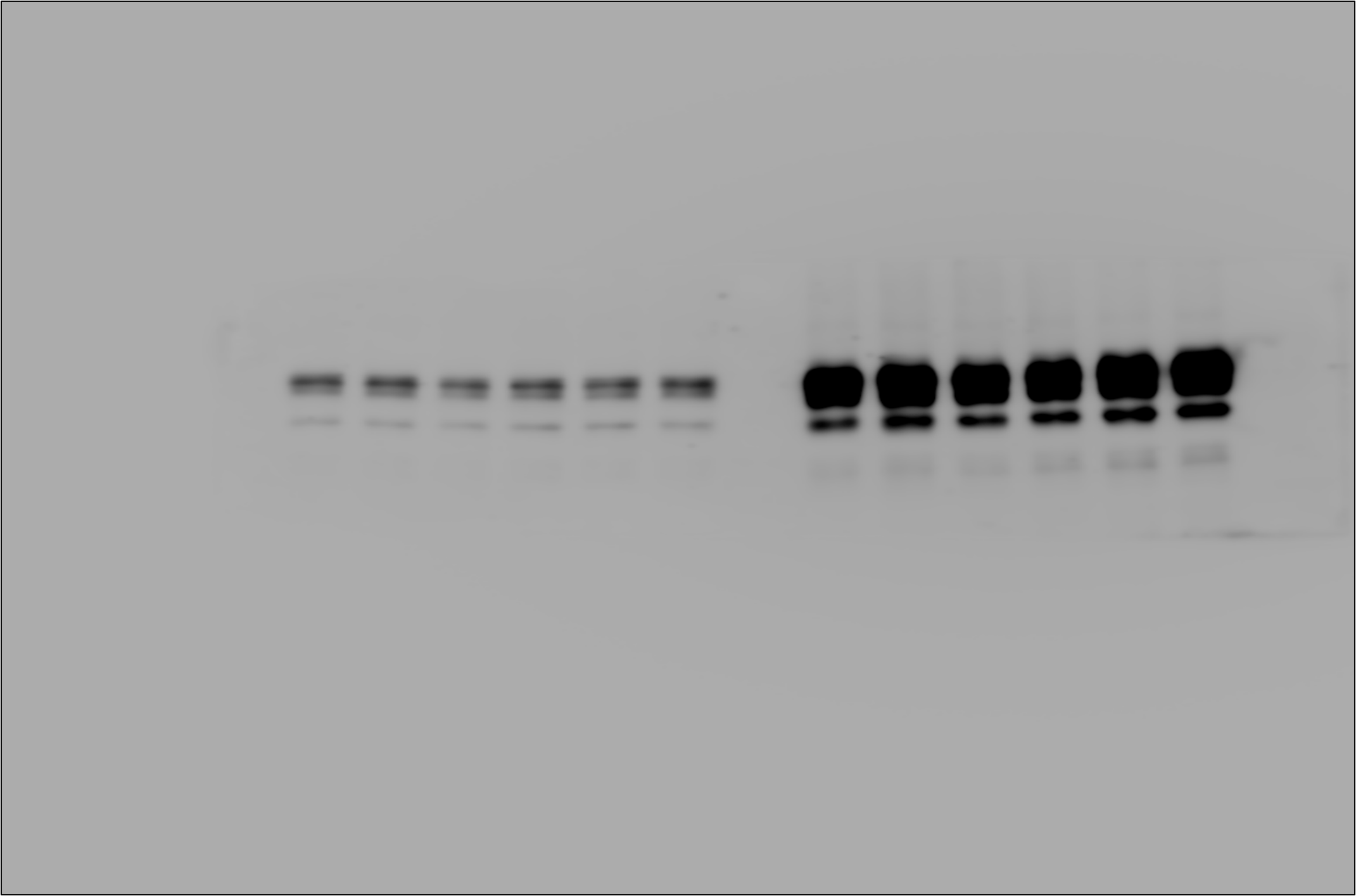

Supplement: Figure 7—figure supplement 1—source data 2. [file elife-108048-fig7-figsupp1-data2.zip › Figure 7-figure supplement 1/Figure S7 C-WCL-Flag.tif]

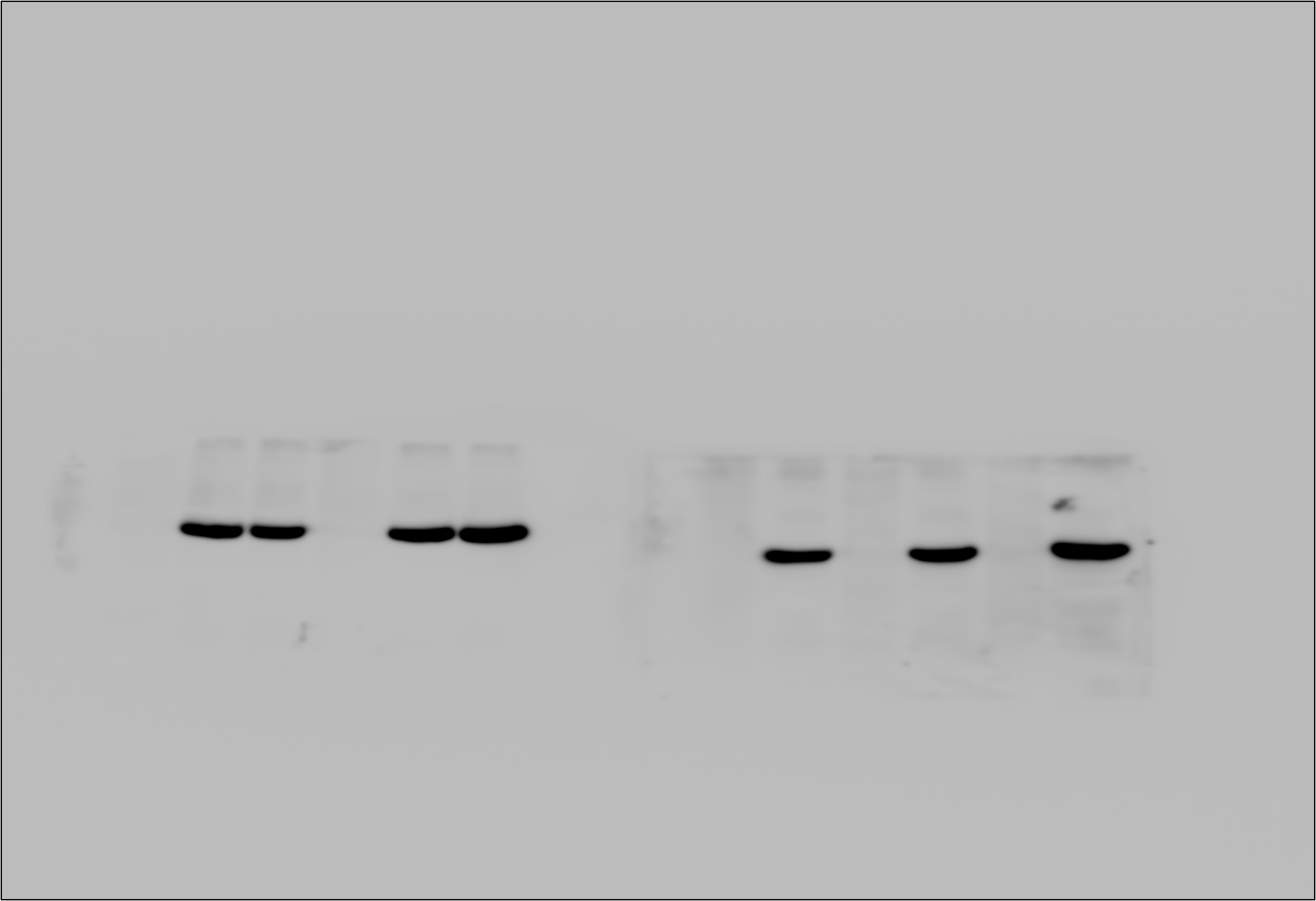

Supplement: Figure 7—figure supplement 1—source data 2. [file elife-108048-fig7-figsupp1-data2.zip › Figure 7-figure supplement 1/Figure S7 C-WCL-HA-cyp17a2.tif]

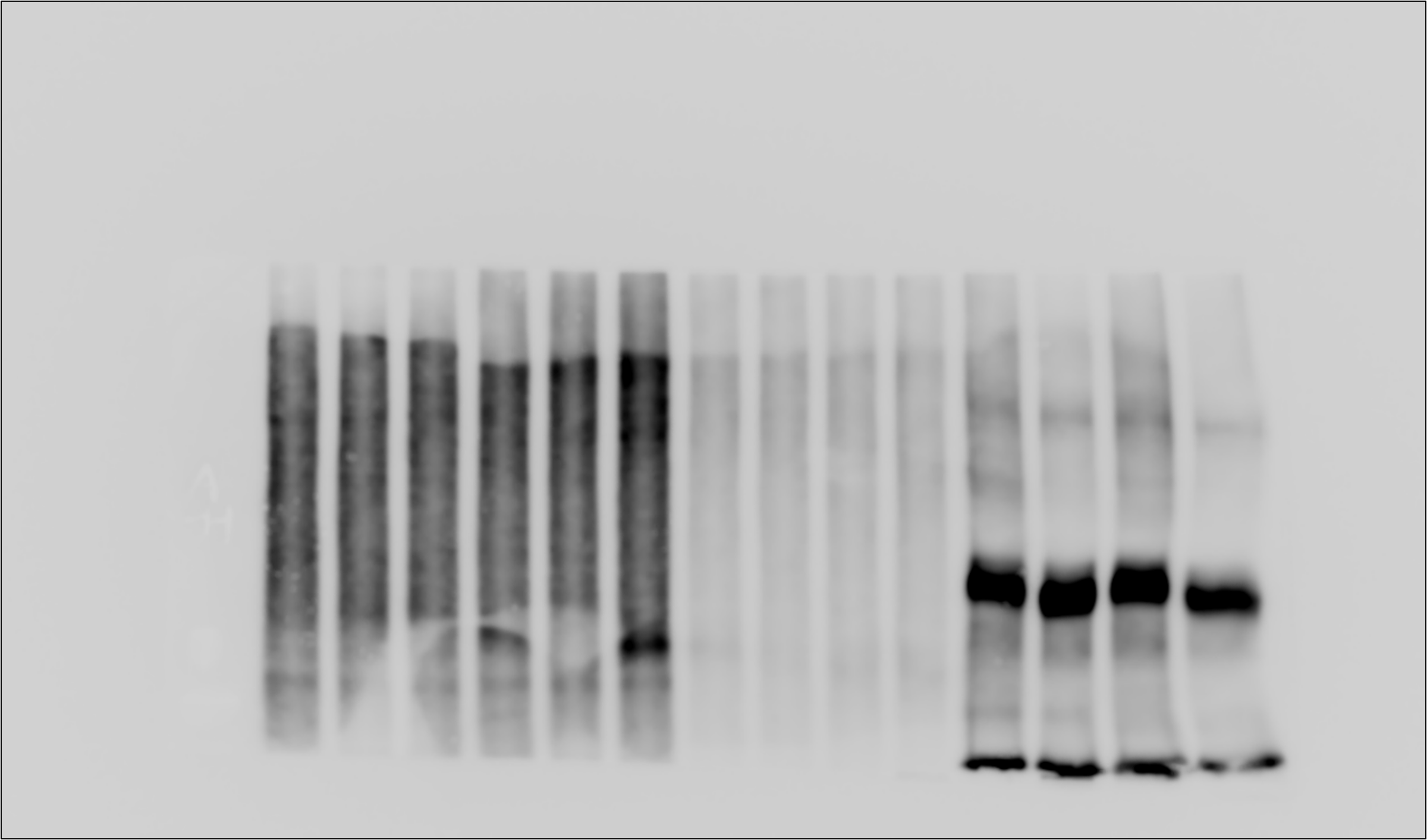

Supplement: Figure 7—figure supplement 1—source data 2. [file elife-108048-fig7-figsupp1-data2.zip › Figure 7-figure supplement 1/Figure S7 C-WCL-HA.tif]

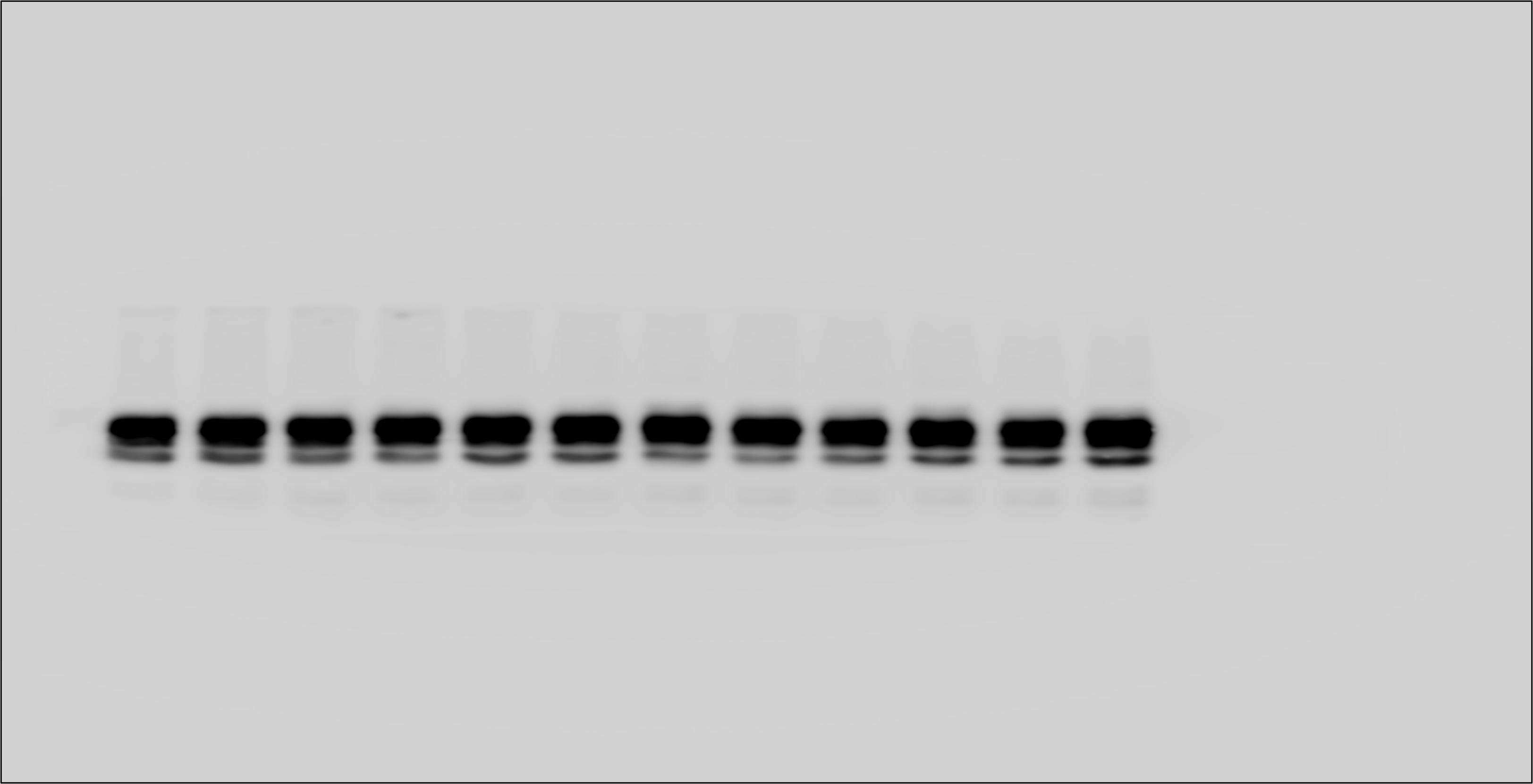

Supplement: Figure 7—figure supplement 1—source data 2. [file elife-108048-fig7-figsupp1-data2.zip › Figure 7-figure supplement 1/Figure S7 D-IP-Flag.tif]

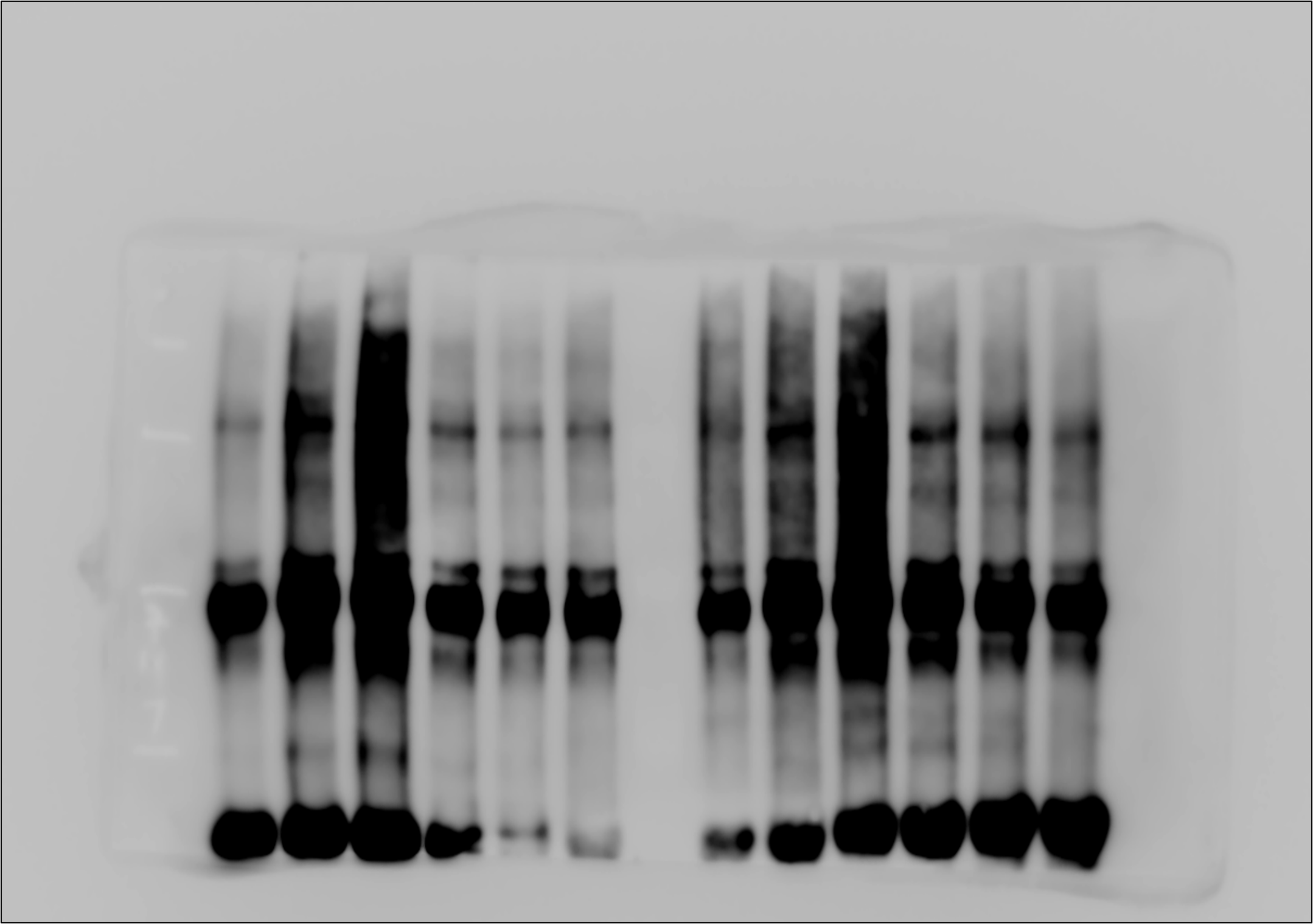

Supplement: Figure 7—figure supplement 1—source data 2. [file elife-108048-fig7-figsupp1-data2.zip › Figure 7-figure supplement 1/Figure S7 D-IP-HA.tif]

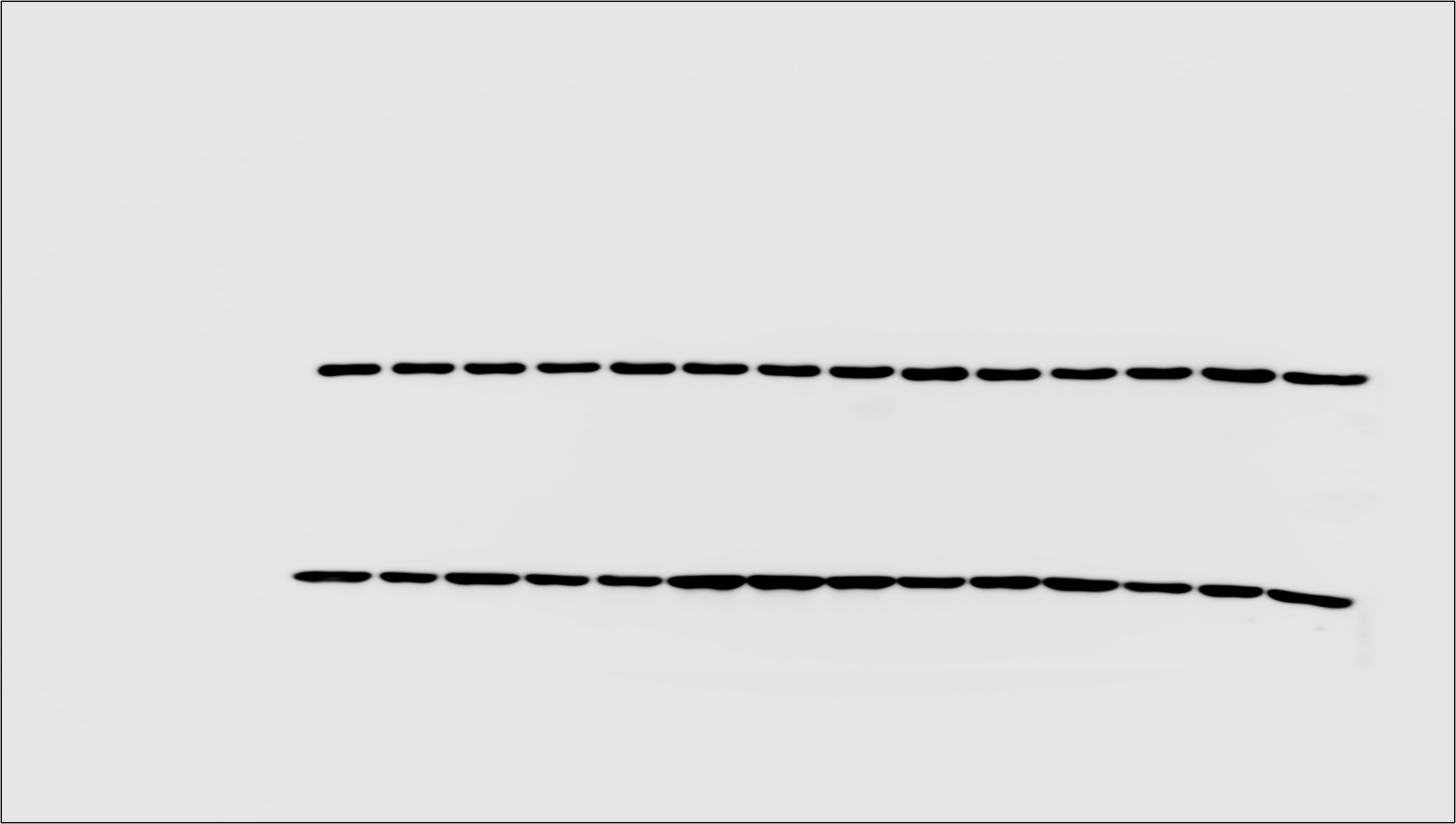

Supplement: Figure 7—figure supplement 1—source data 2. [file elife-108048-fig7-figsupp1-data2.zip › Figure 7-figure supplement 1/Figure S7 D-WCL-Actin.tif]

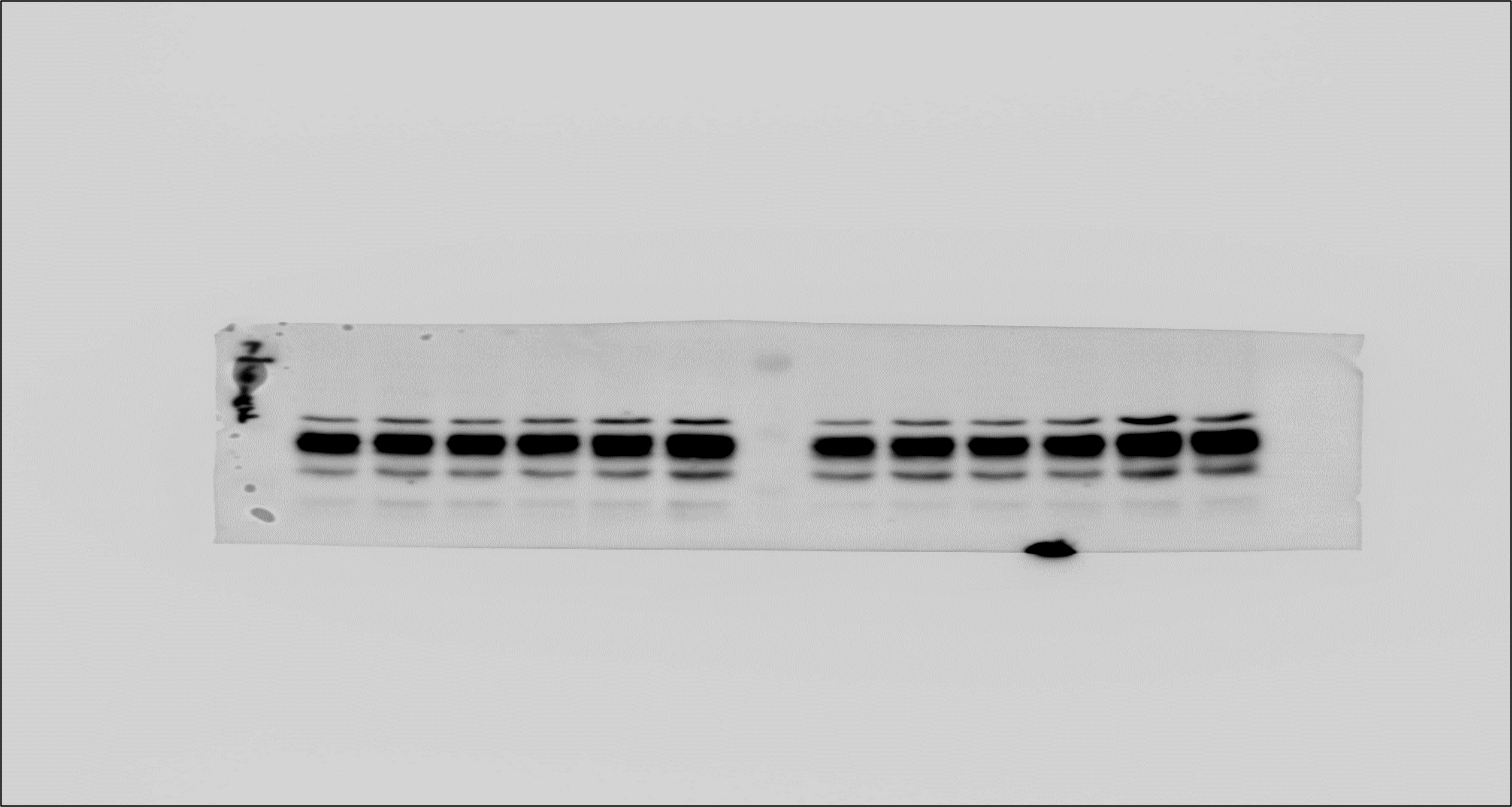

Supplement: Figure 7—figure supplement 1—source data 2. [file elife-108048-fig7-figsupp1-data2.zip › Figure 7-figure supplement 1/Figure S7 D-WCL-Flag.tif]

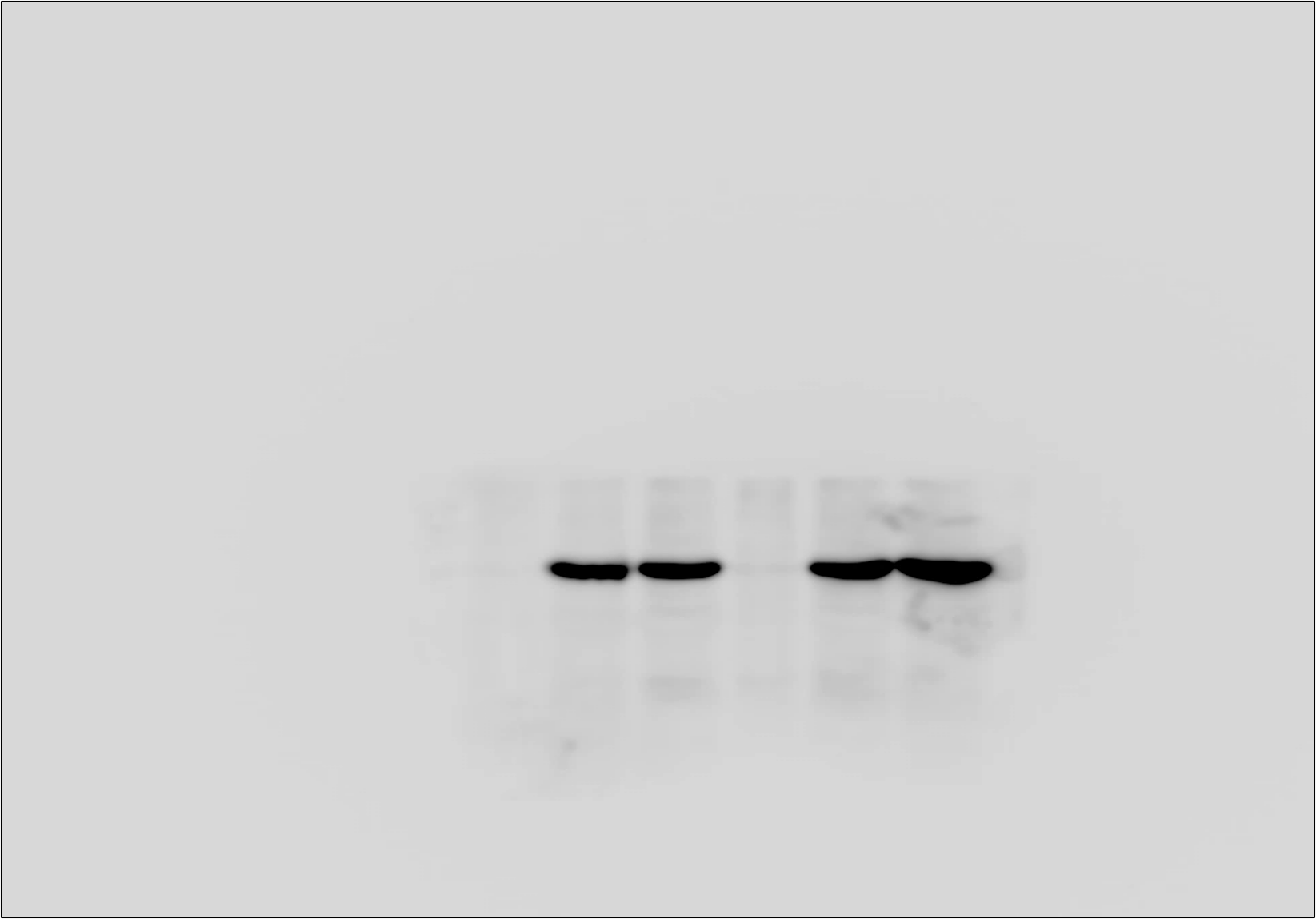

Supplement: Figure 7—figure supplement 1—source data 2. [file elife-108048-fig7-figsupp1-data2.zip › Figure 7-figure supplement 1/Figure S7 D-WCL-HA-cyp17a2.tif]

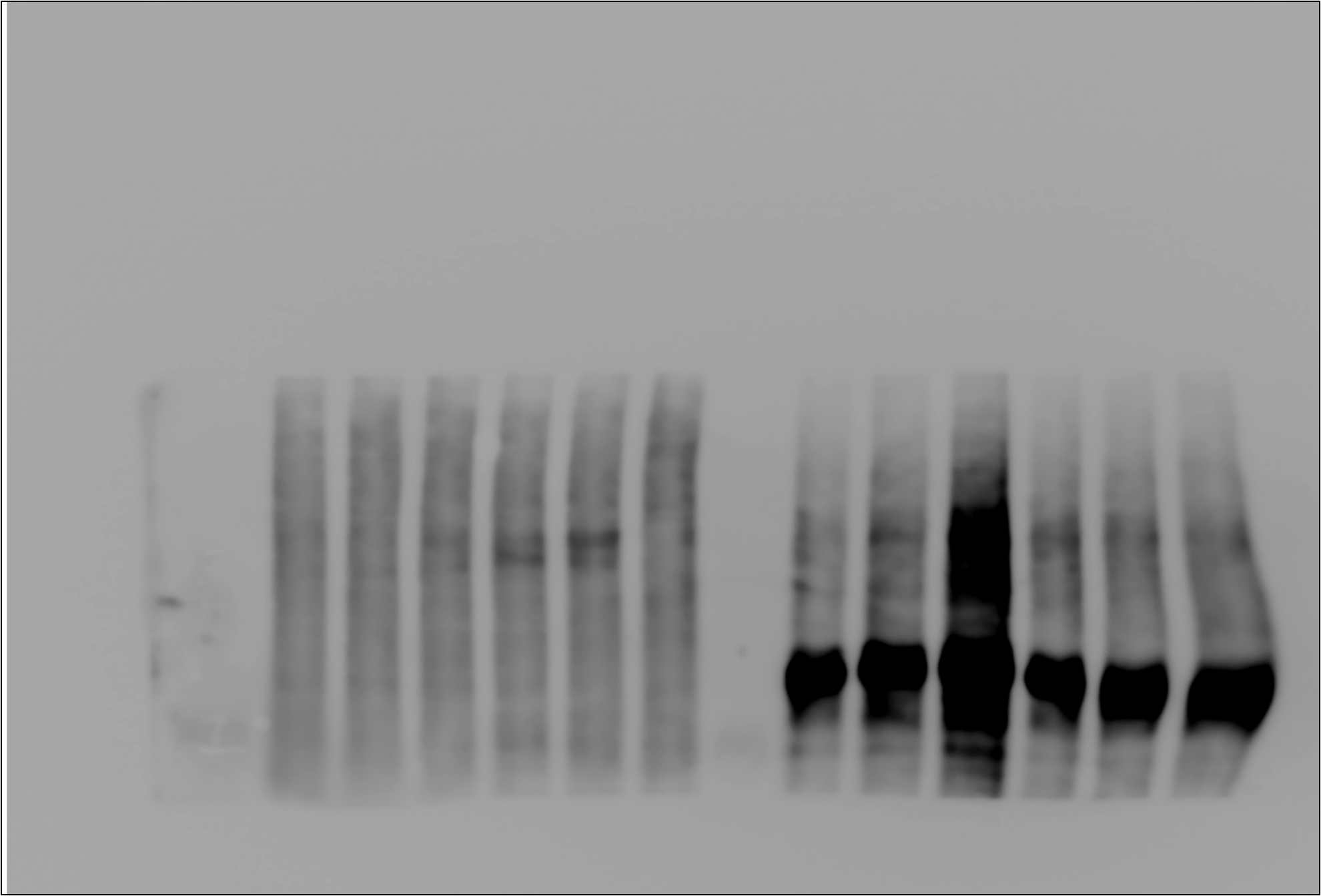

Supplement: Figure 7—figure supplement 1—source data 2. [file elife-108048-fig7-figsupp1-data2.zip › Figure 7-figure supplement 1/Figure S7 D-WCL-HA.tif]

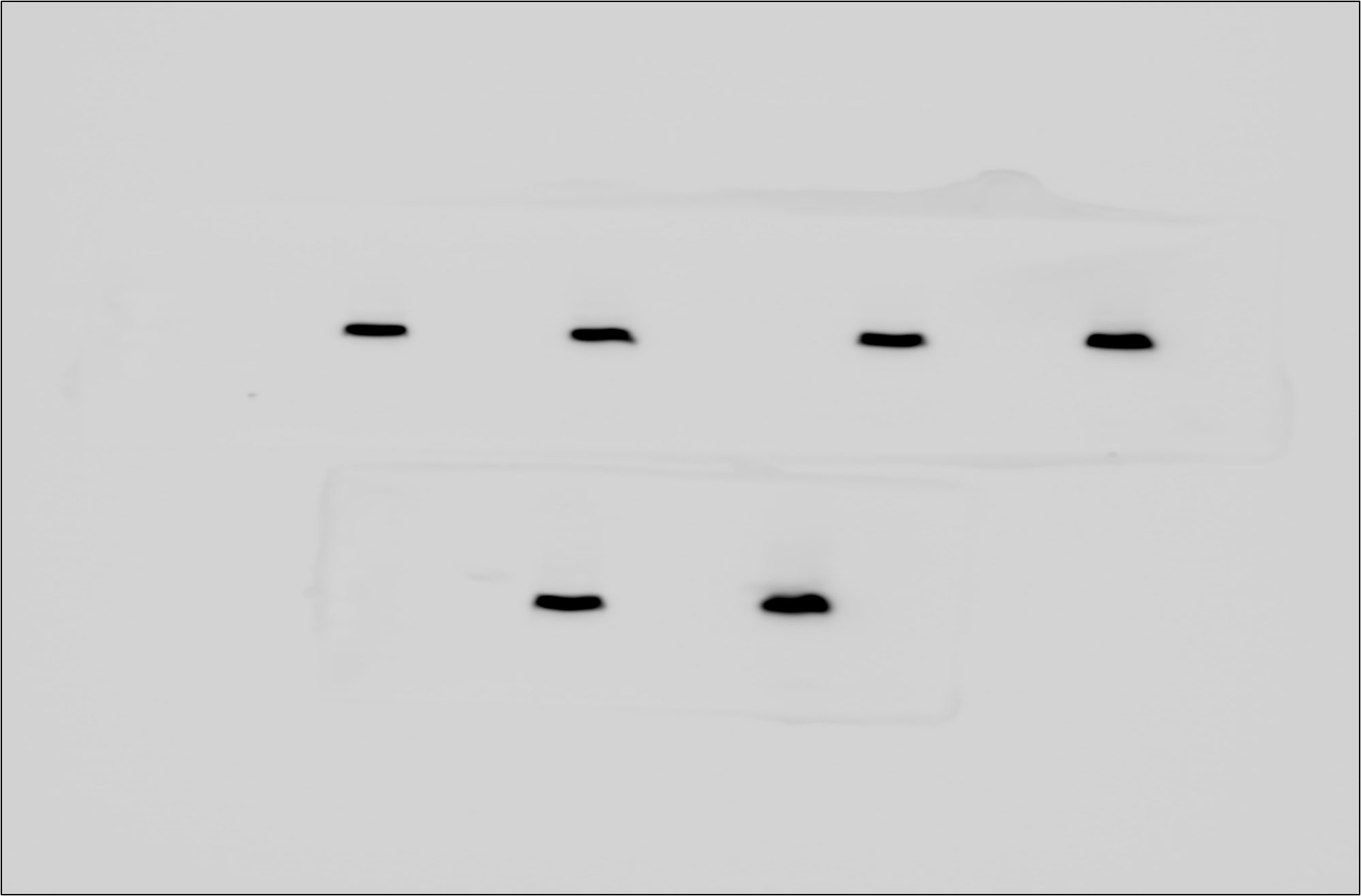

Supplement: Figure 7—figure supplement 1—source data 2. [file elife-108048-fig7-figsupp1-data2.zip › Figure 7-figure supplement 1/Figure S7 D-WCL-Myc.tif]

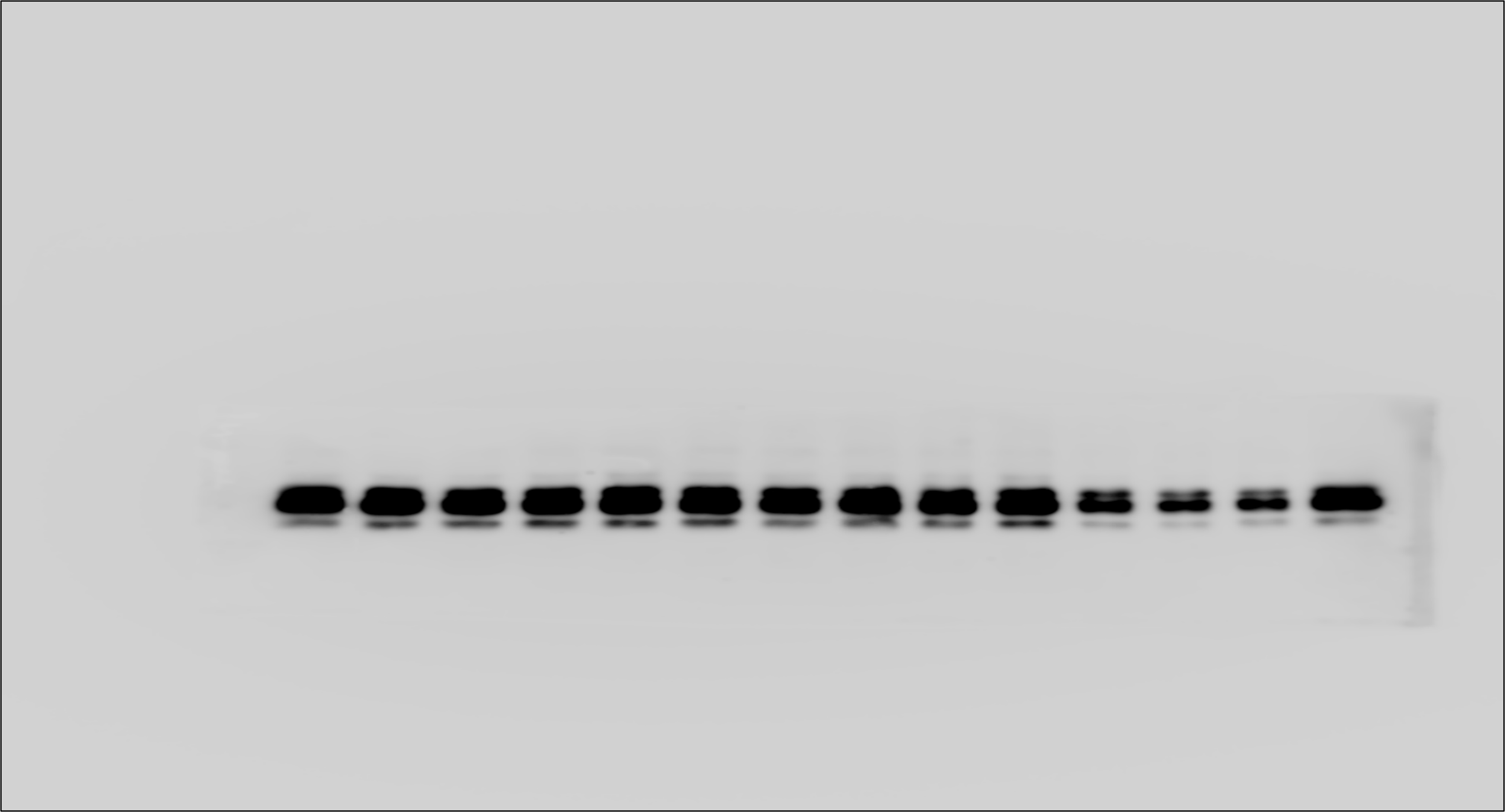

Supplement: Figure 7—figure supplement 1—source data 2. [file elife-108048-fig7-figsupp1-data2.zip › Figure 7-figure supplement 1/Figure S7 E-IP-Flag.tif]

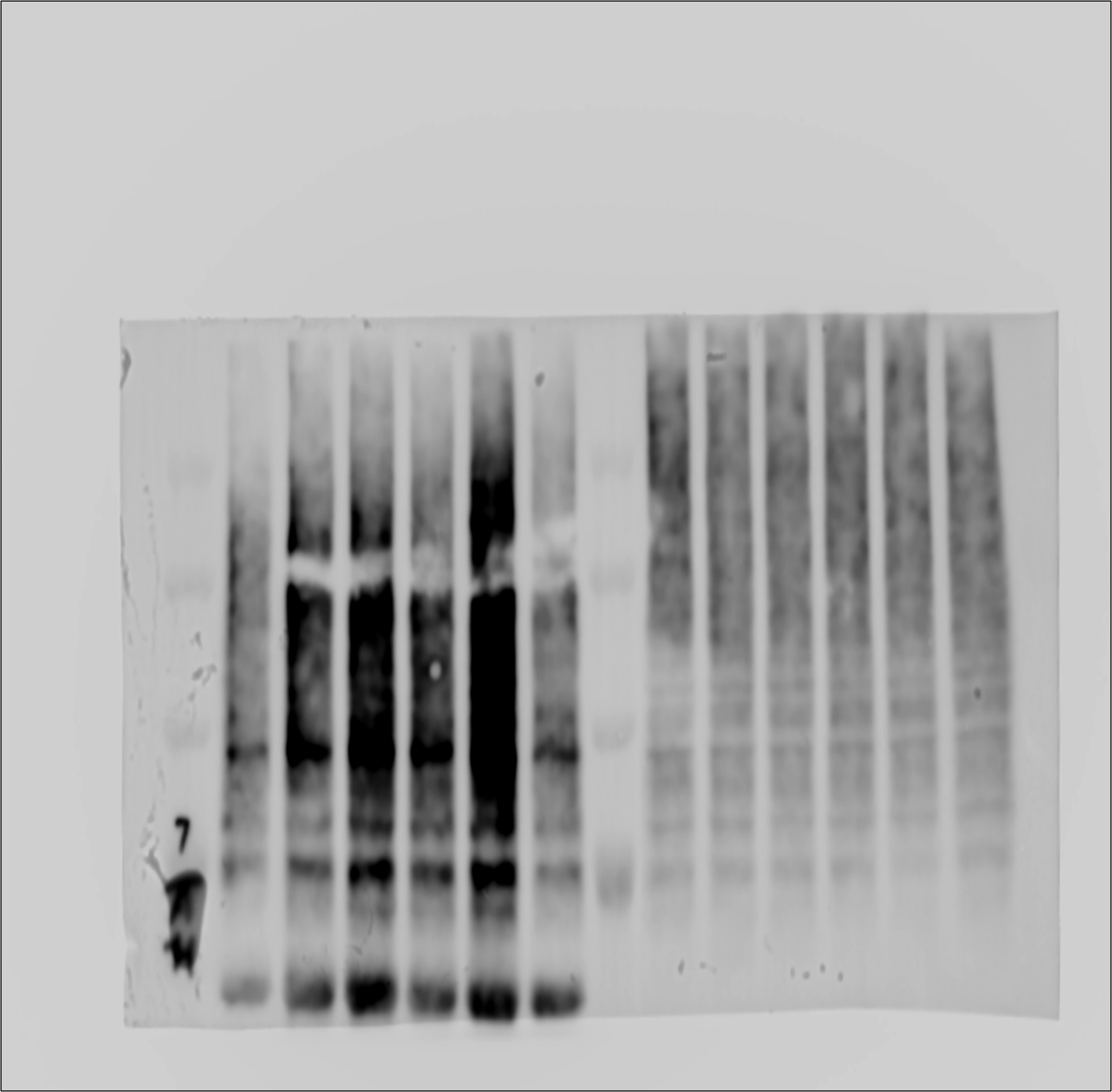

Supplement: Figure 7—figure supplement 1—source data 2. [file elife-108048-fig7-figsupp1-data2.zip › Figure 7-figure supplement 1/Figure S7 E-IP-HA.tif]

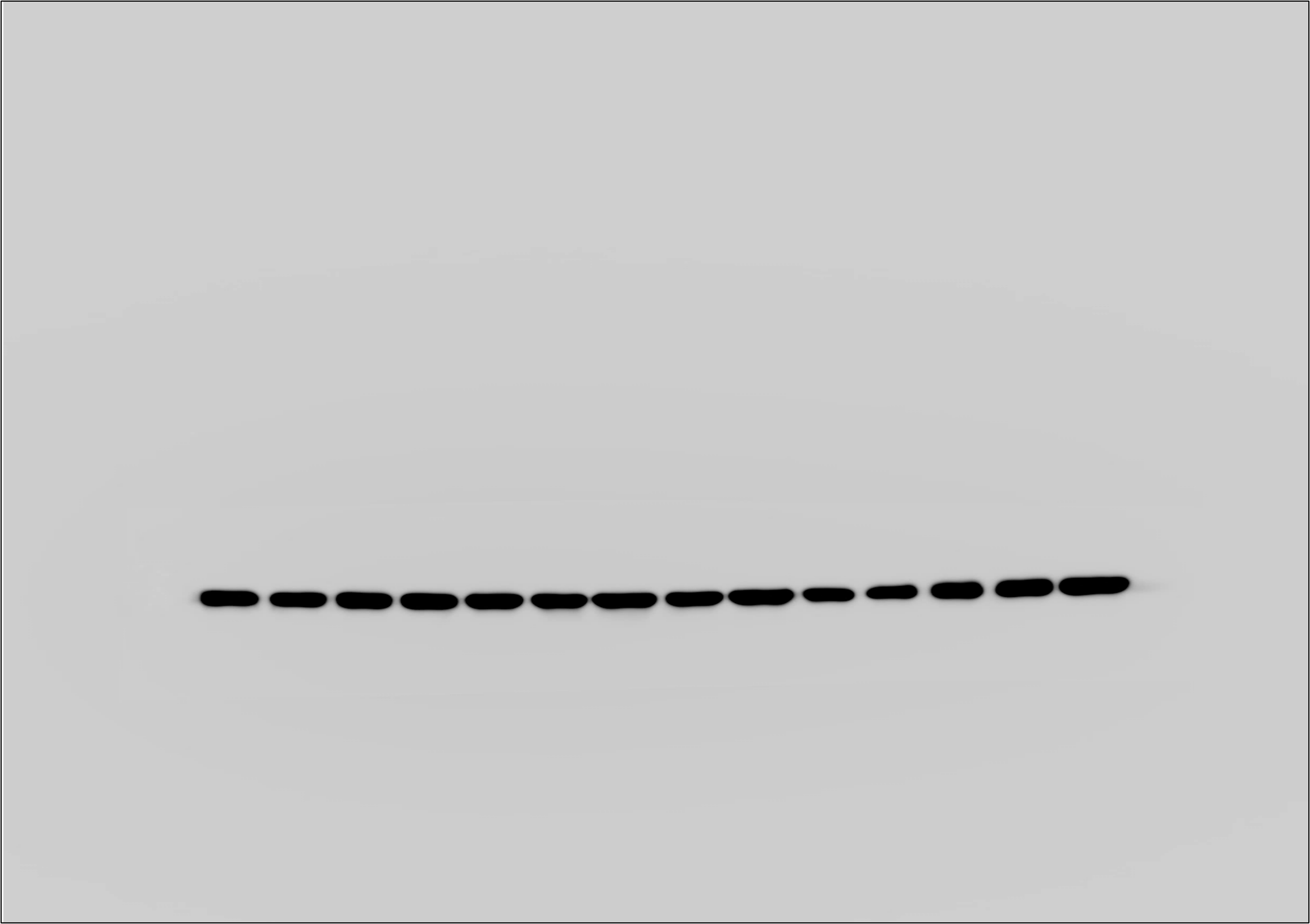

Supplement: Figure 7—figure supplement 1—source data 2. [file elife-108048-fig7-figsupp1-data2.zip › Figure 7-figure supplement 1/Figure S7 E-WCL-Actin.tif]

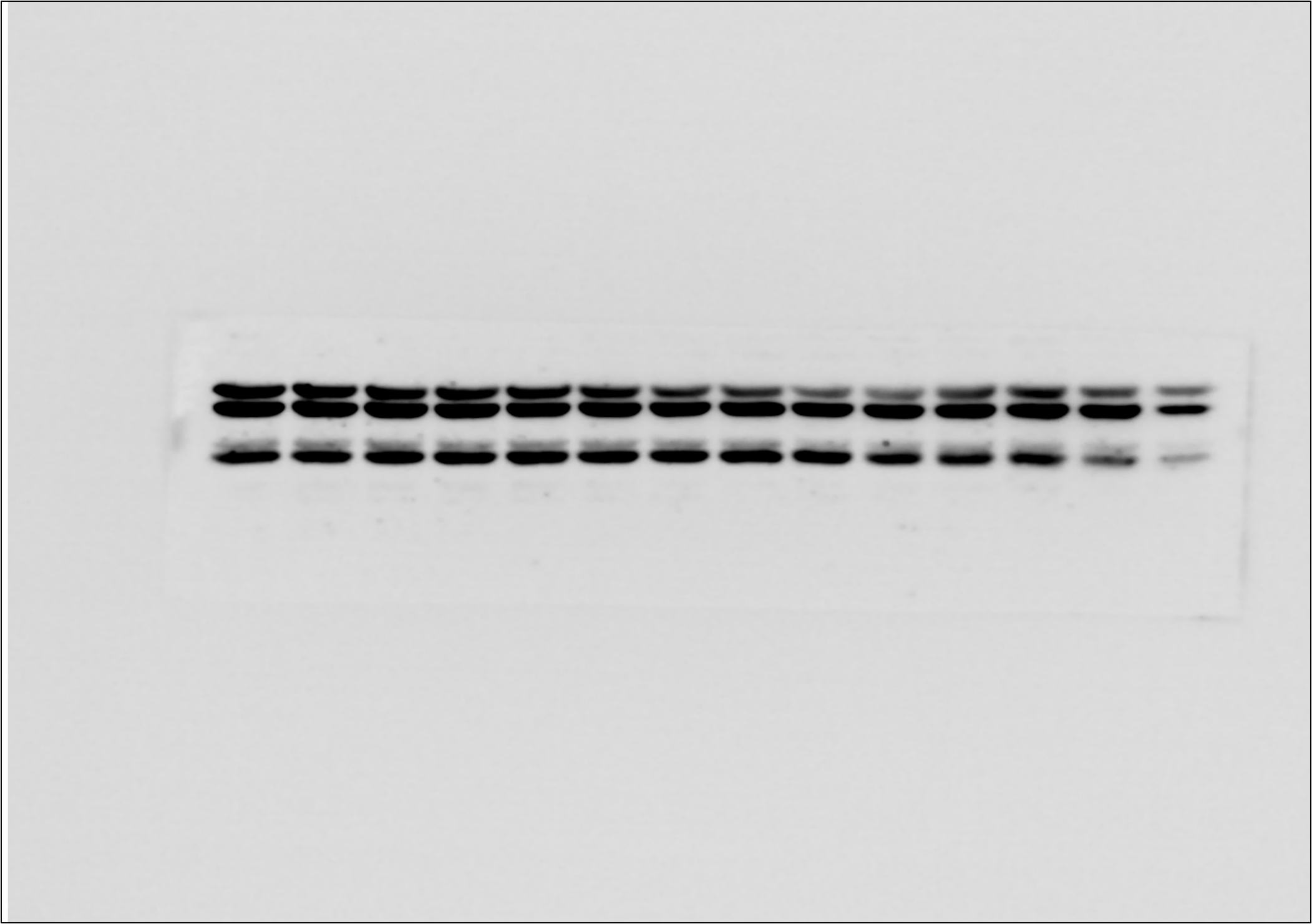

Supplement: Figure 7—figure supplement 1—source data 2. [file elife-108048-fig7-figsupp1-data2.zip › Figure 7-figure supplement 1/Figure S7 E-WCL-Flag.tif]

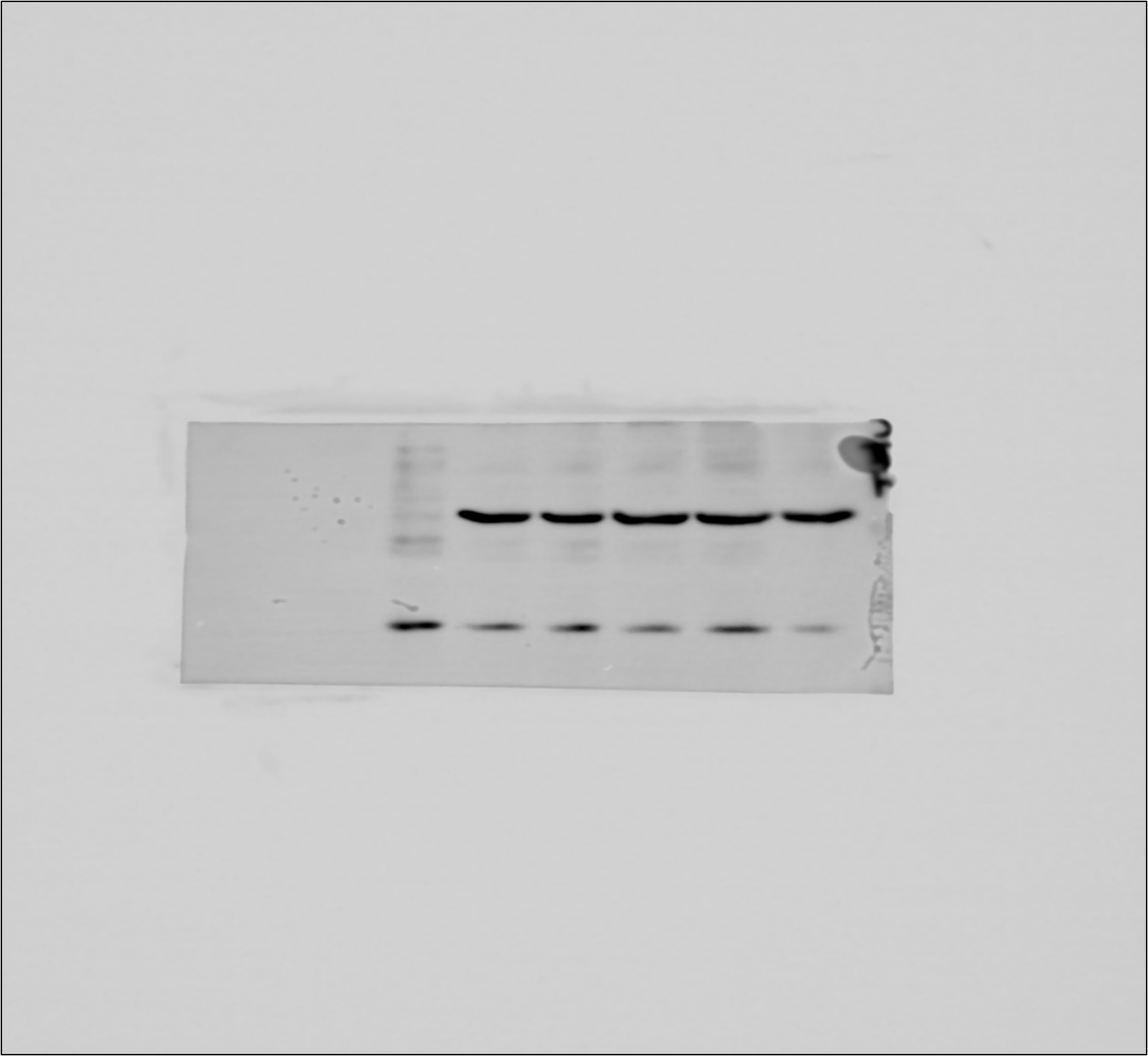

Supplement: Figure 7—figure supplement 1—source data 2. [file elife-108048-fig7-figsupp1-data2.zip › Figure 7-figure supplement 1/Figure S7 E-WCL-HA-cyp17a2.tif]

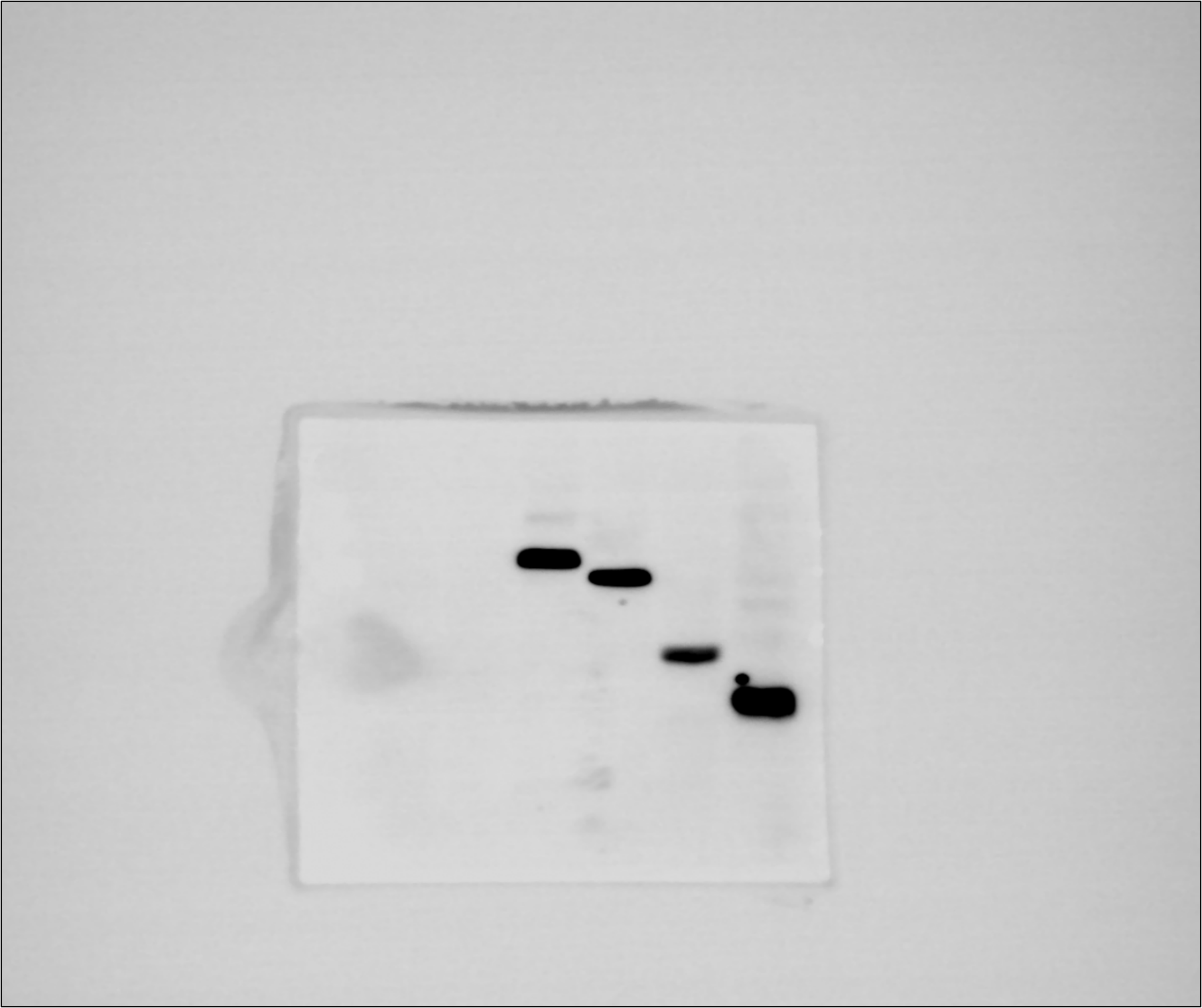

Supplement: Figure 7—figure supplement 1—source data 2. [file elife-108048-fig7-figsupp1-data2.zip › Figure 7-figure supplement 1/Figure S7 E-WCL-Myc.tif]

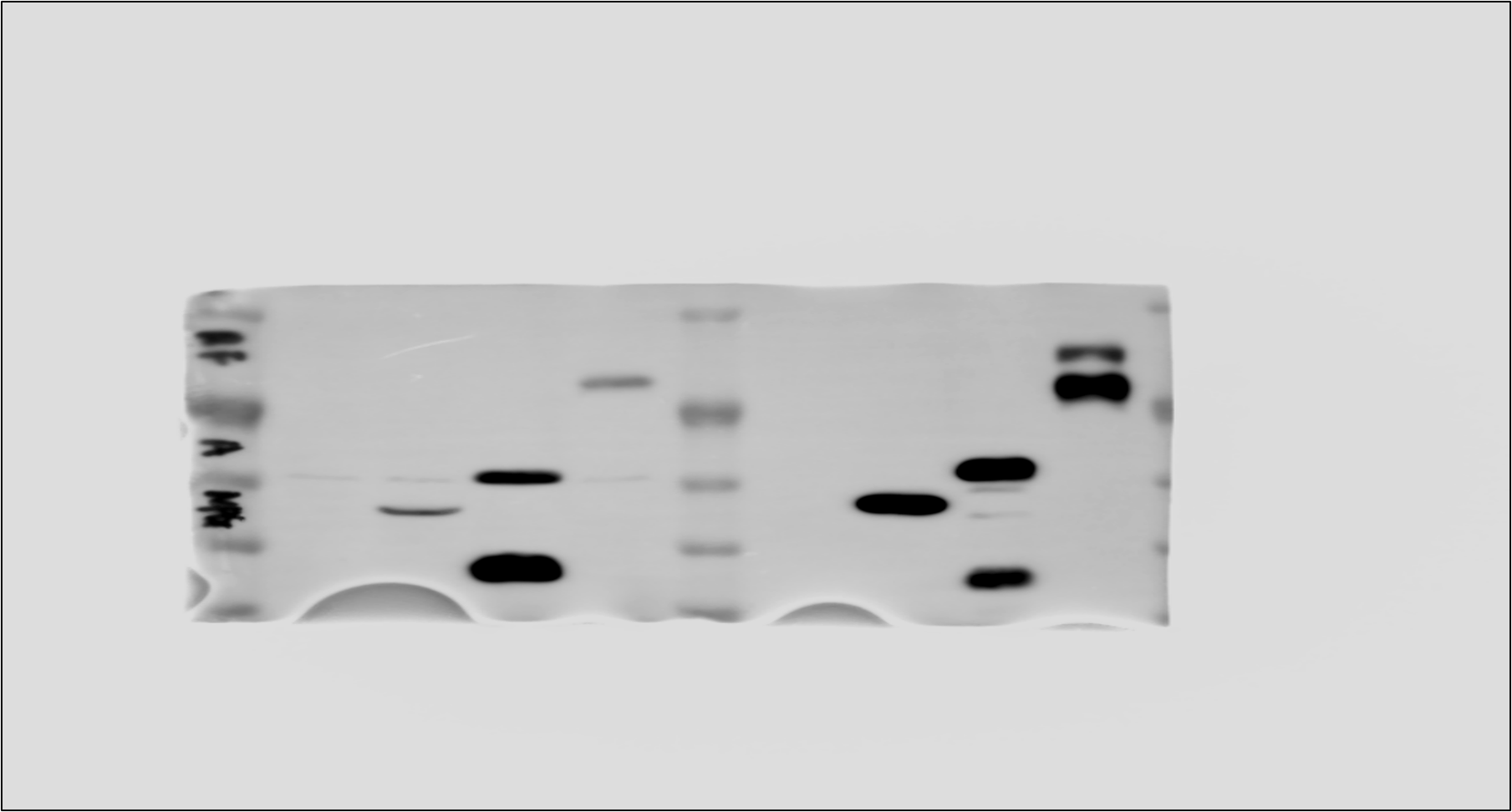

Supplement: Figure 8—source data 2. [file elife-108048-fig8-data2.zip › Figure 8/Figure 8 A-IP-Flag.tif]

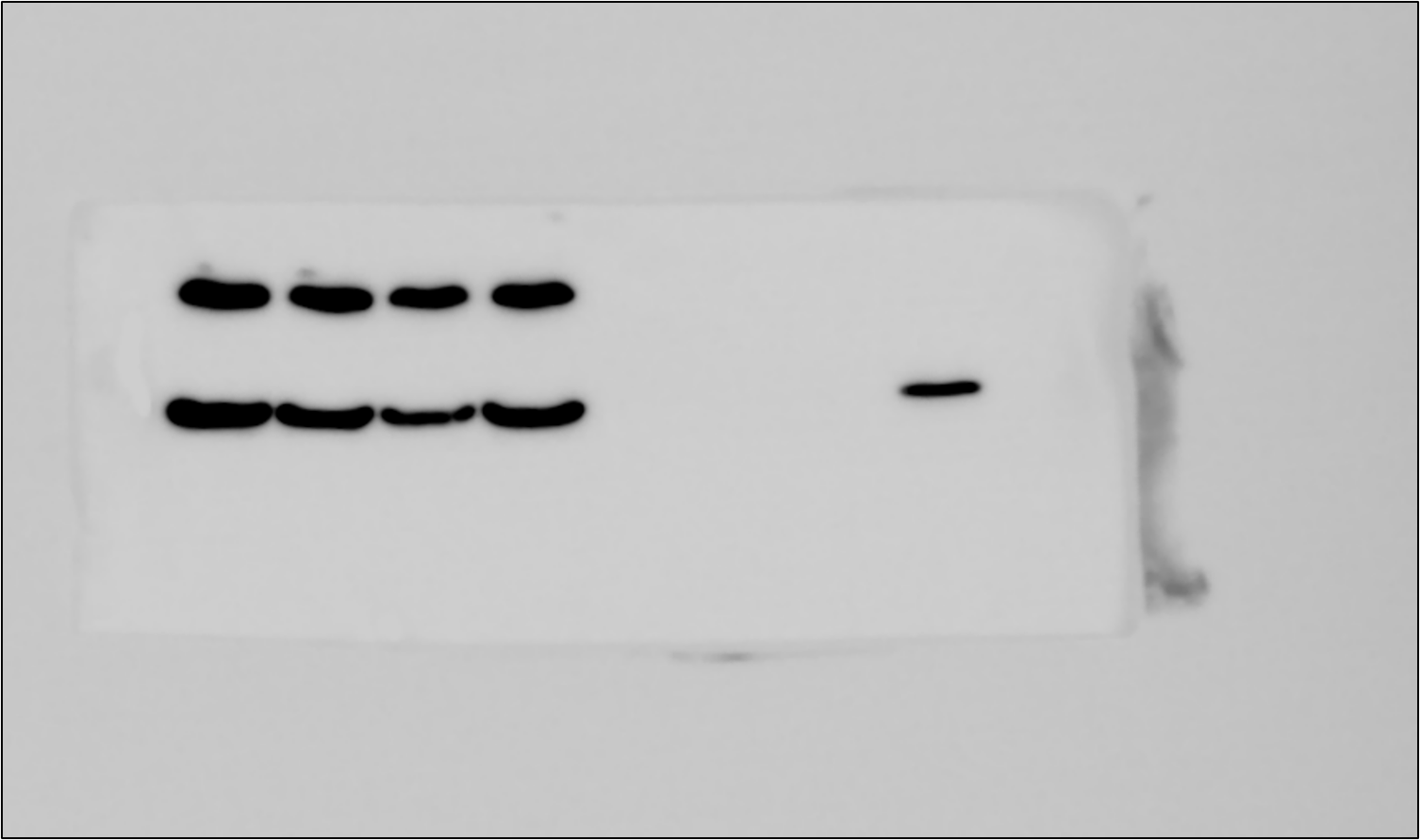

Supplement: Figure 8—source data 2. [file elife-108048-fig8-data2.zip › Figure 8/Figure 8 A-IP-Myc.tif]

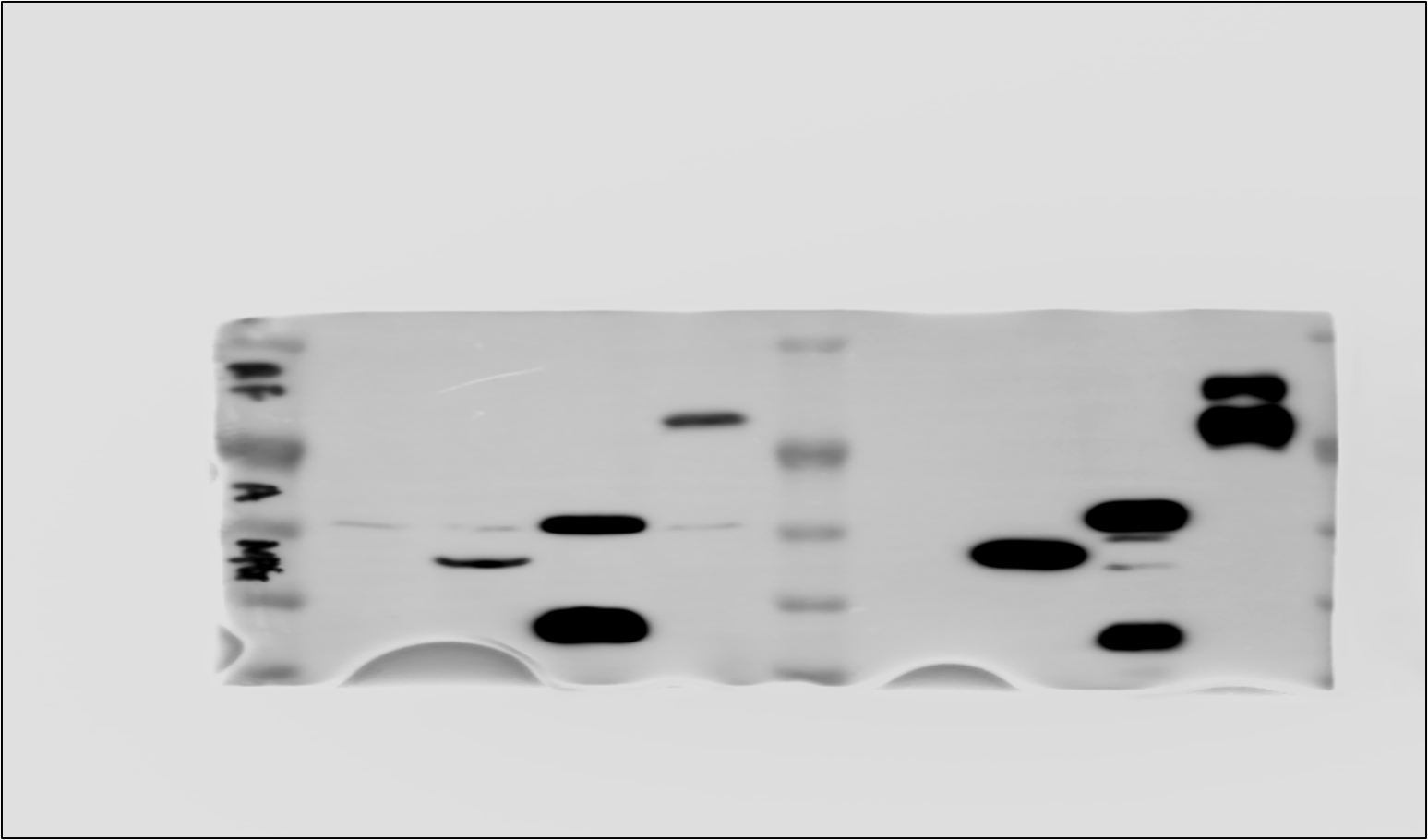

Supplement: Figure 8—source data 2. [file elife-108048-fig8-data2.zip › Figure 8/Figure 8 A-WCL-Flag.tif]

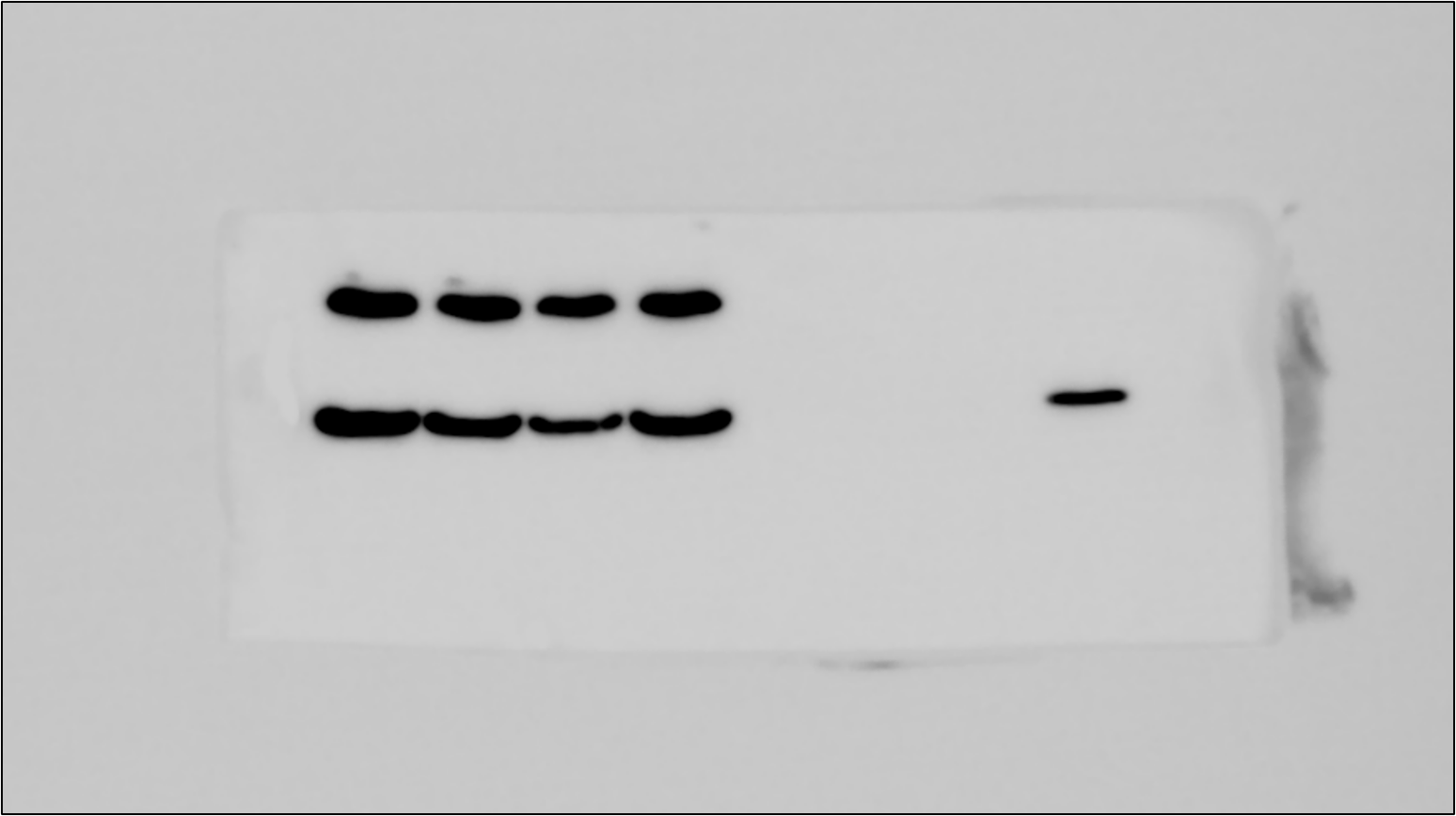

Supplement: Figure 8—source data 2. [file elife-108048-fig8-data2.zip › Figure 8/Figure 8 A-WCL-Myc.tif]

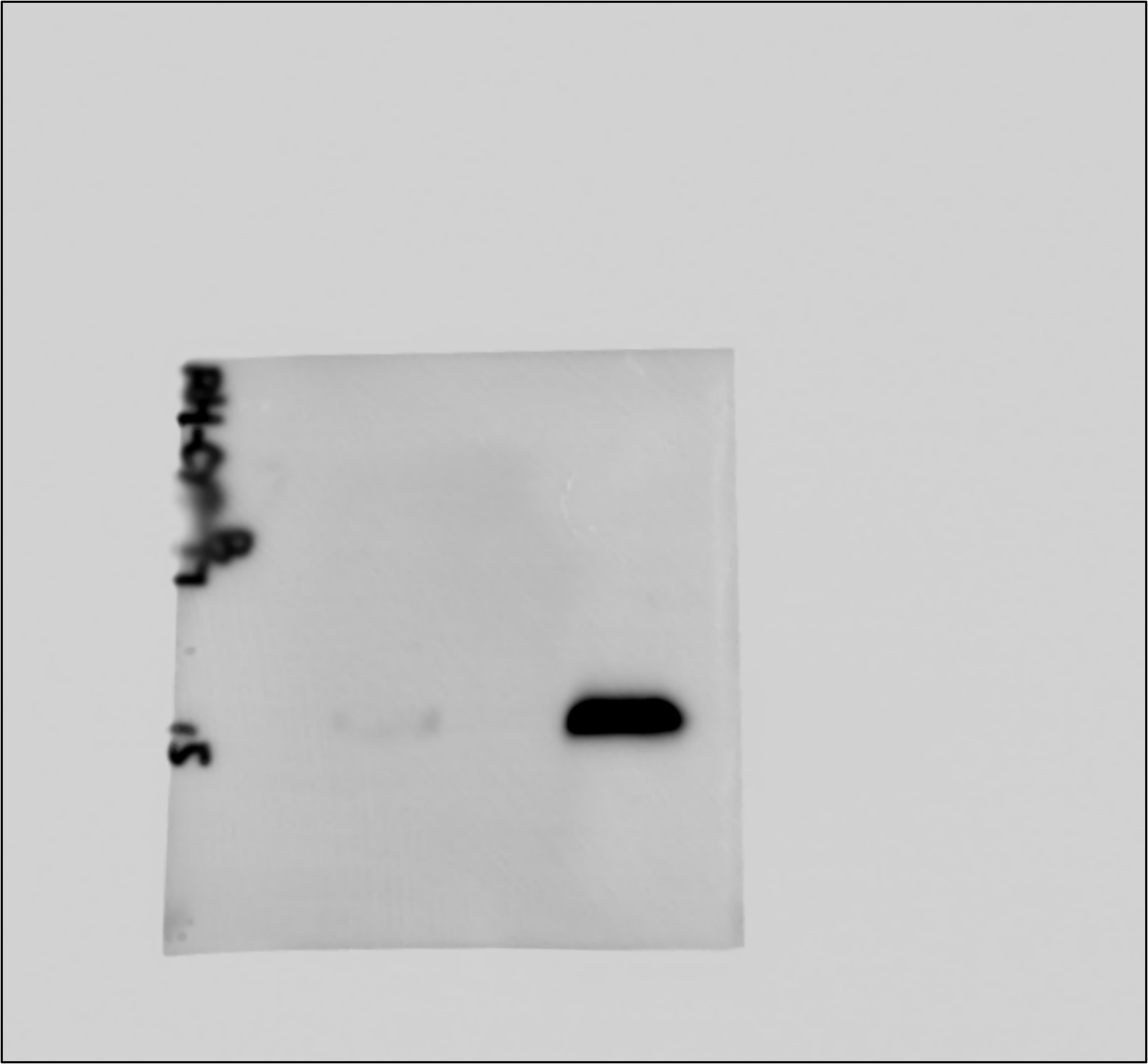

Supplement: Figure 8—source data 2. [file elife-108048-fig8-data2.zip › Figure 8/Figure 8 B-IP-HA.tif]

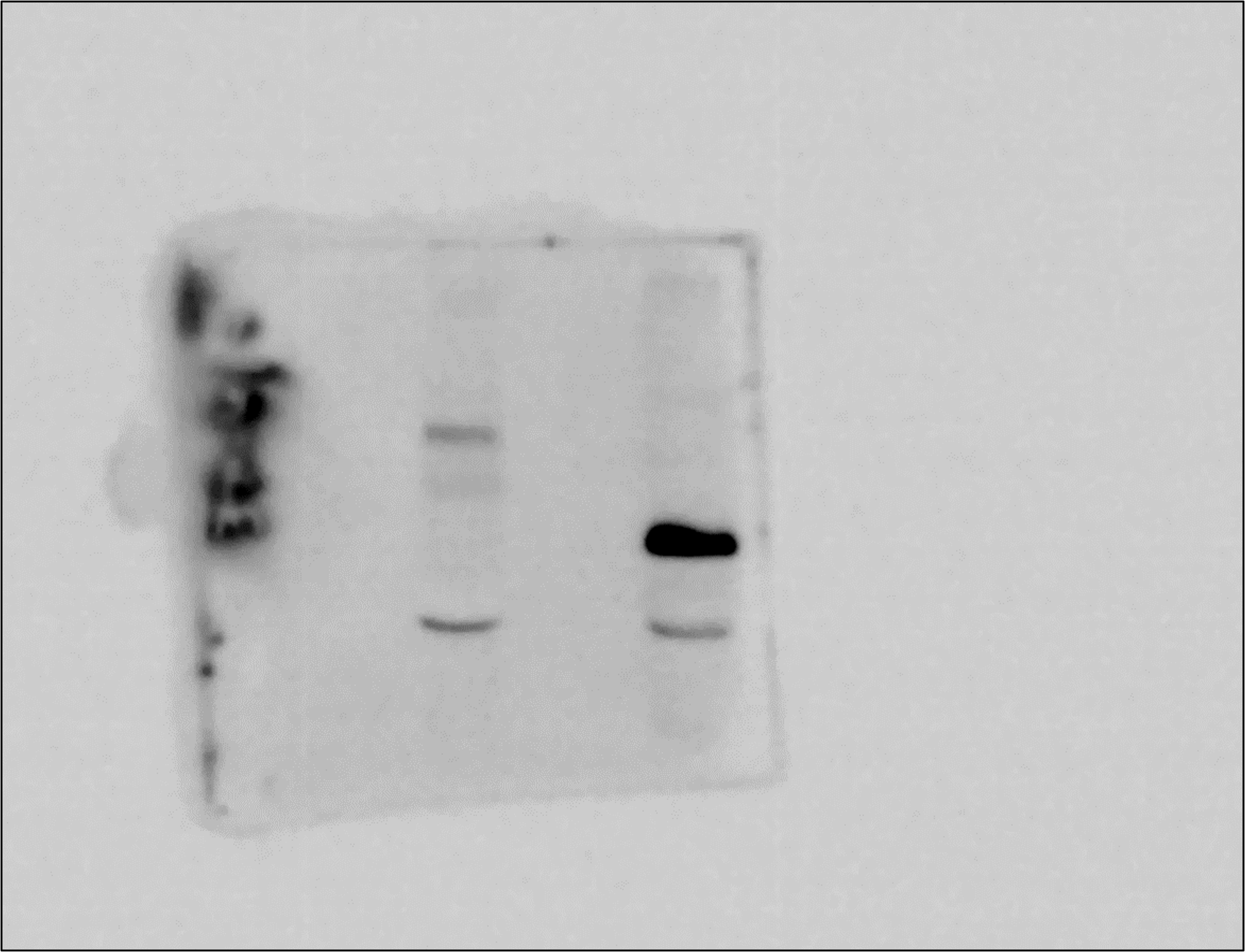

Supplement: Figure 8—source data 2. [file elife-108048-fig8-data2.zip › Figure 8/Figure 8 B-IP-Myc.tif]

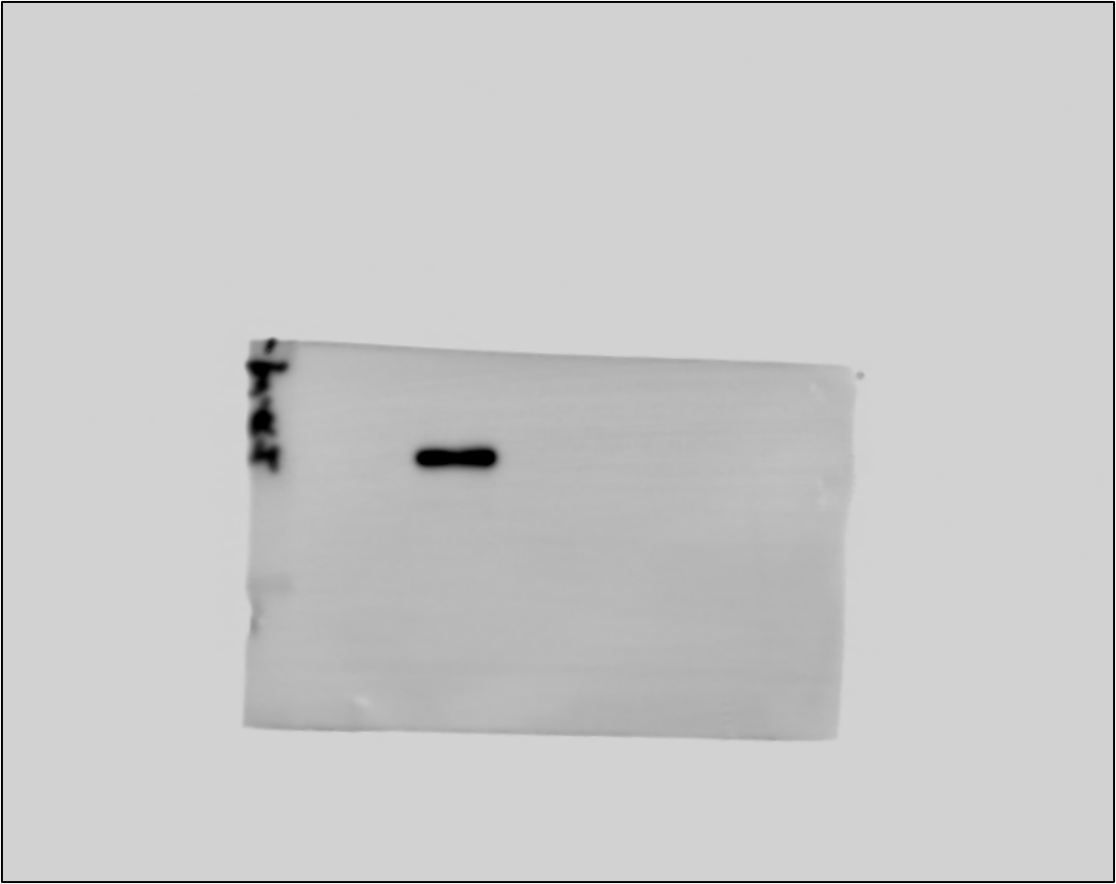

Supplement: Figure 8—source data 2. [file elife-108048-fig8-data2.zip › Figure 8/Figure 8 B-WCL-HA.tif]

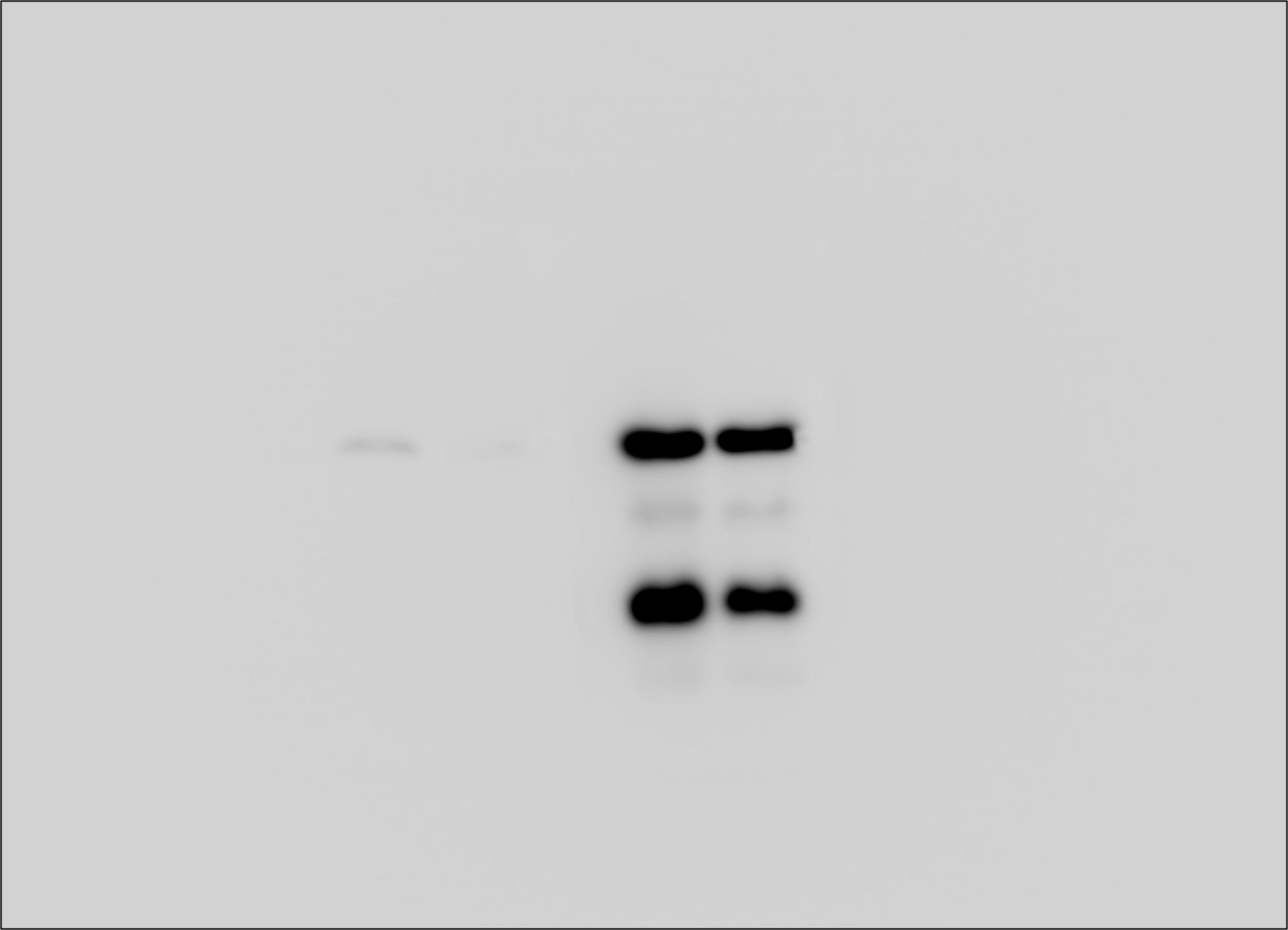

Supplement: Figure 8—source data 2. [file elife-108048-fig8-data2.zip › Figure 8/Figure 8 B-WCL-Myc.tif]

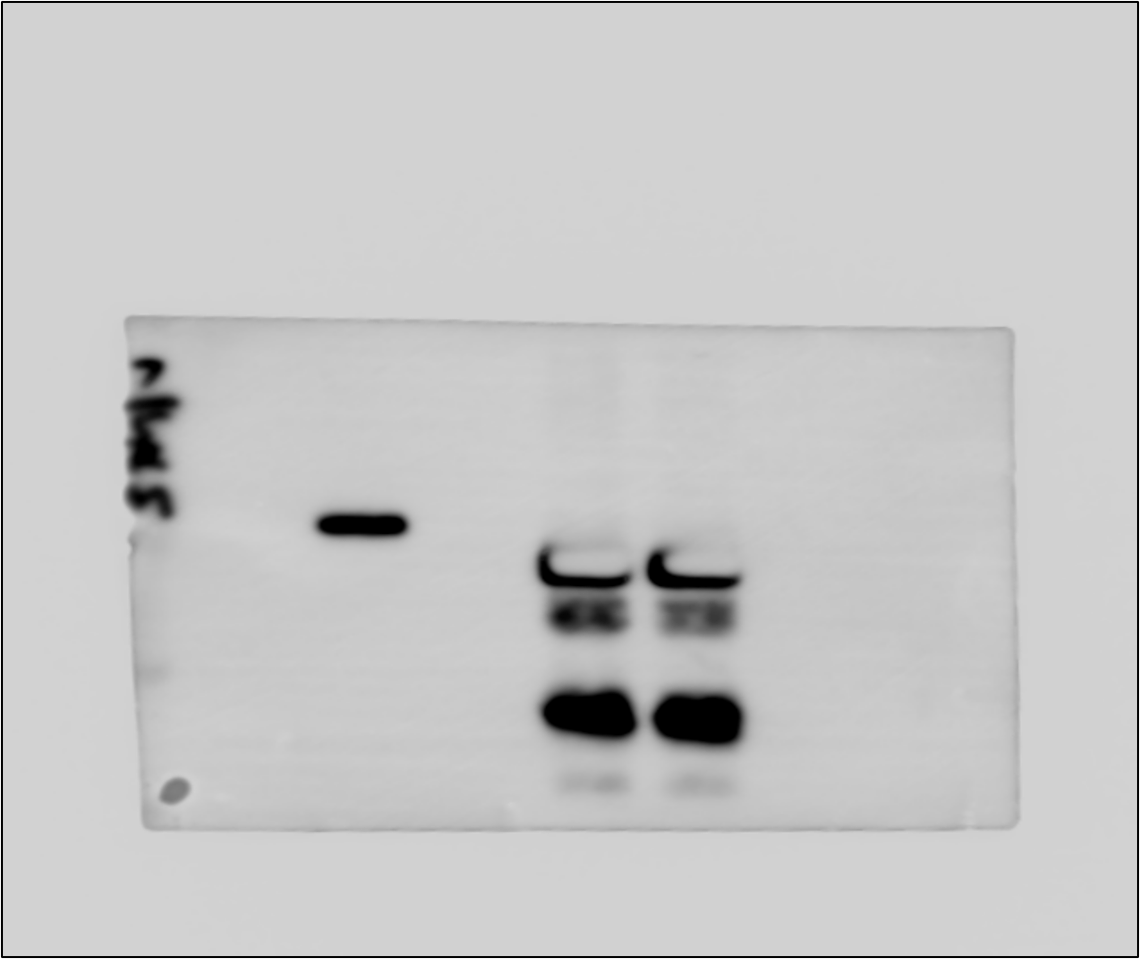

Supplement: Figure 8—source data 2. [file elife-108048-fig8-data2.zip › Figure 8/Figure 8 C-IP-Myc.tif]

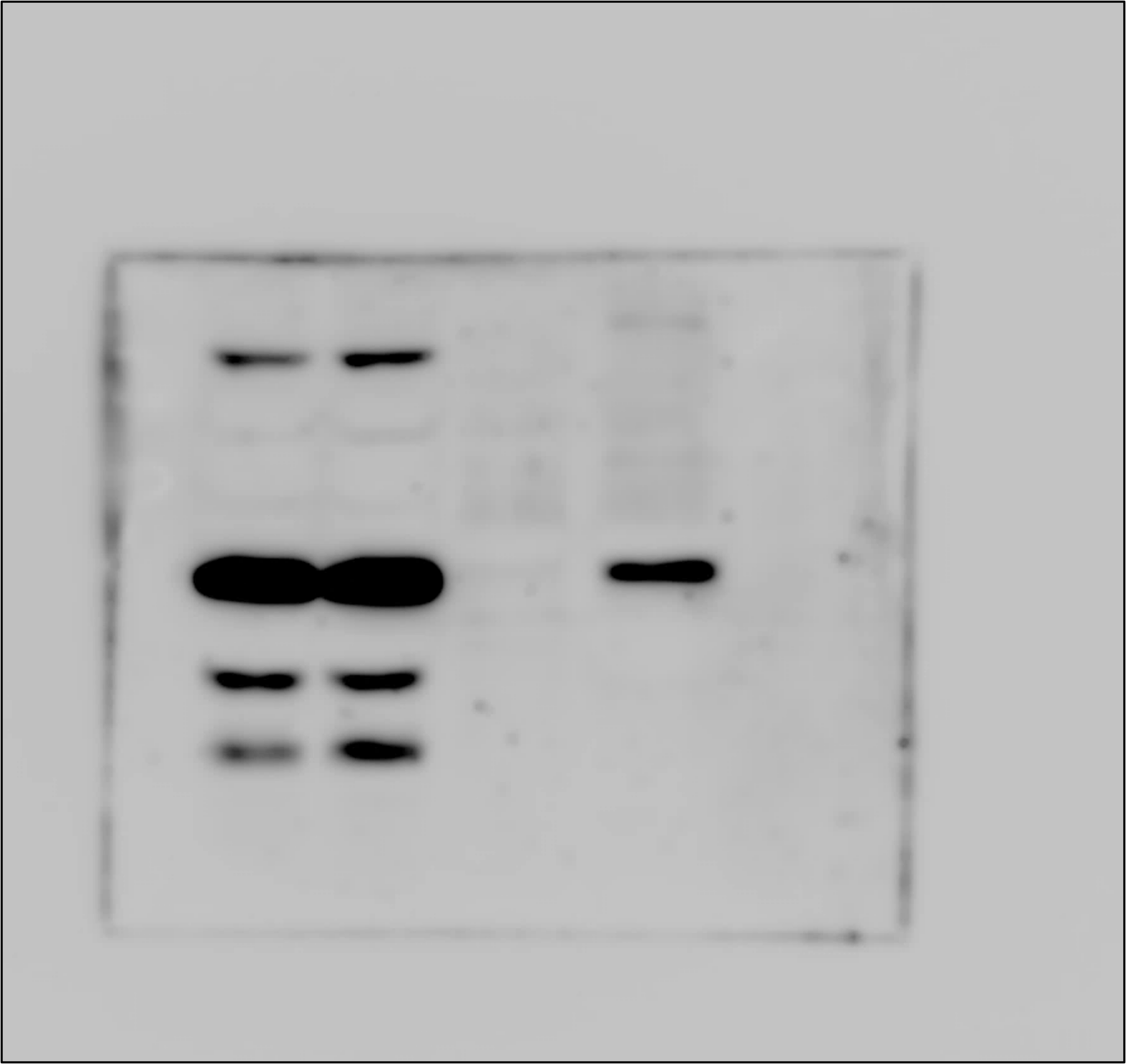

Supplement: Figure 8—source data 2. [file elife-108048-fig8-data2.zip › Figure 8/Figure 8 C-IP-P.tif]

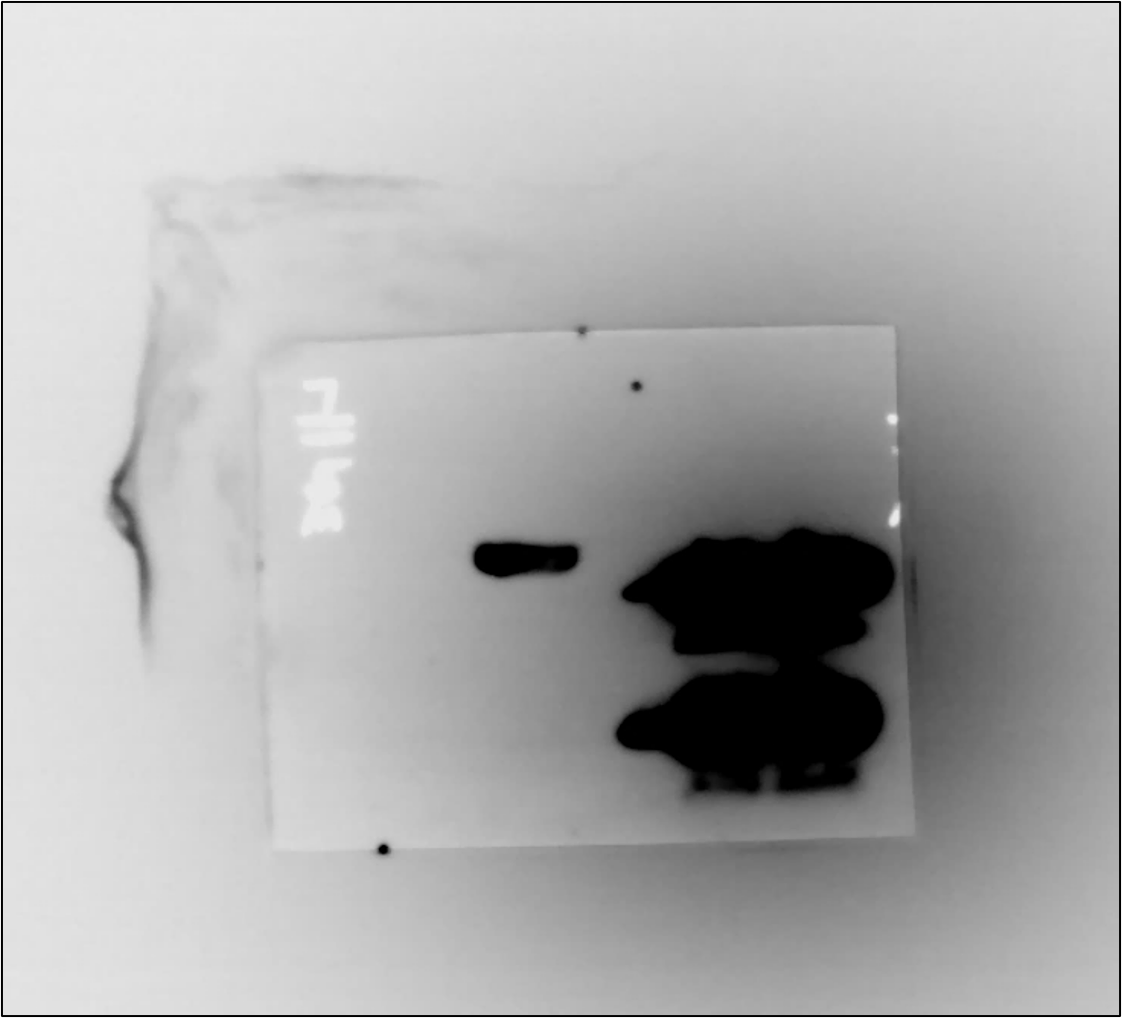

Supplement: Figure 8—source data 2. [file elife-108048-fig8-data2.zip › Figure 8/Figure 8 C-WCL-Myc.tif]

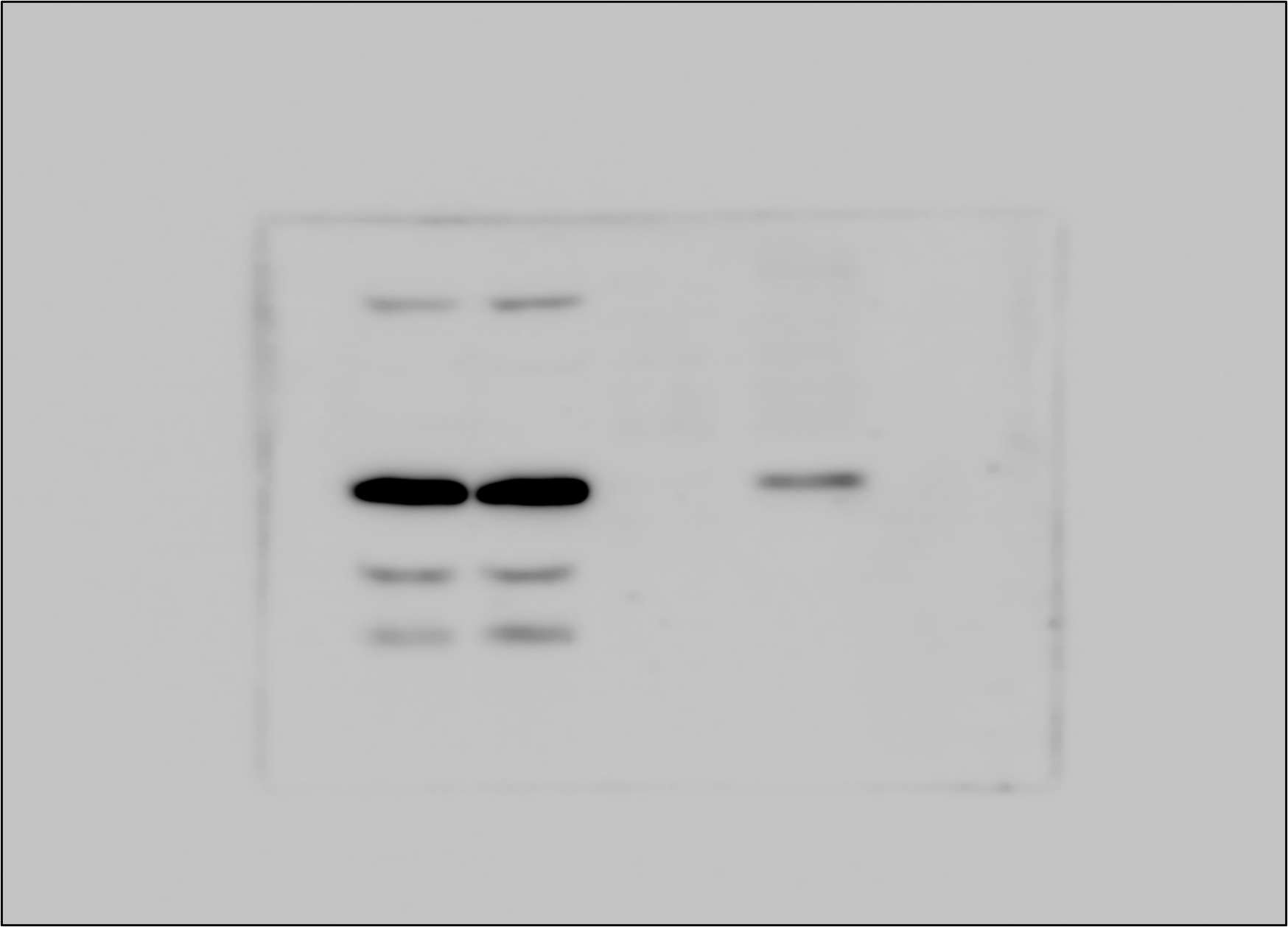

Supplement: Figure 8—source data 2. [file elife-108048-fig8-data2.zip › Figure 8/Figure 8 C-WCL-P.tif]

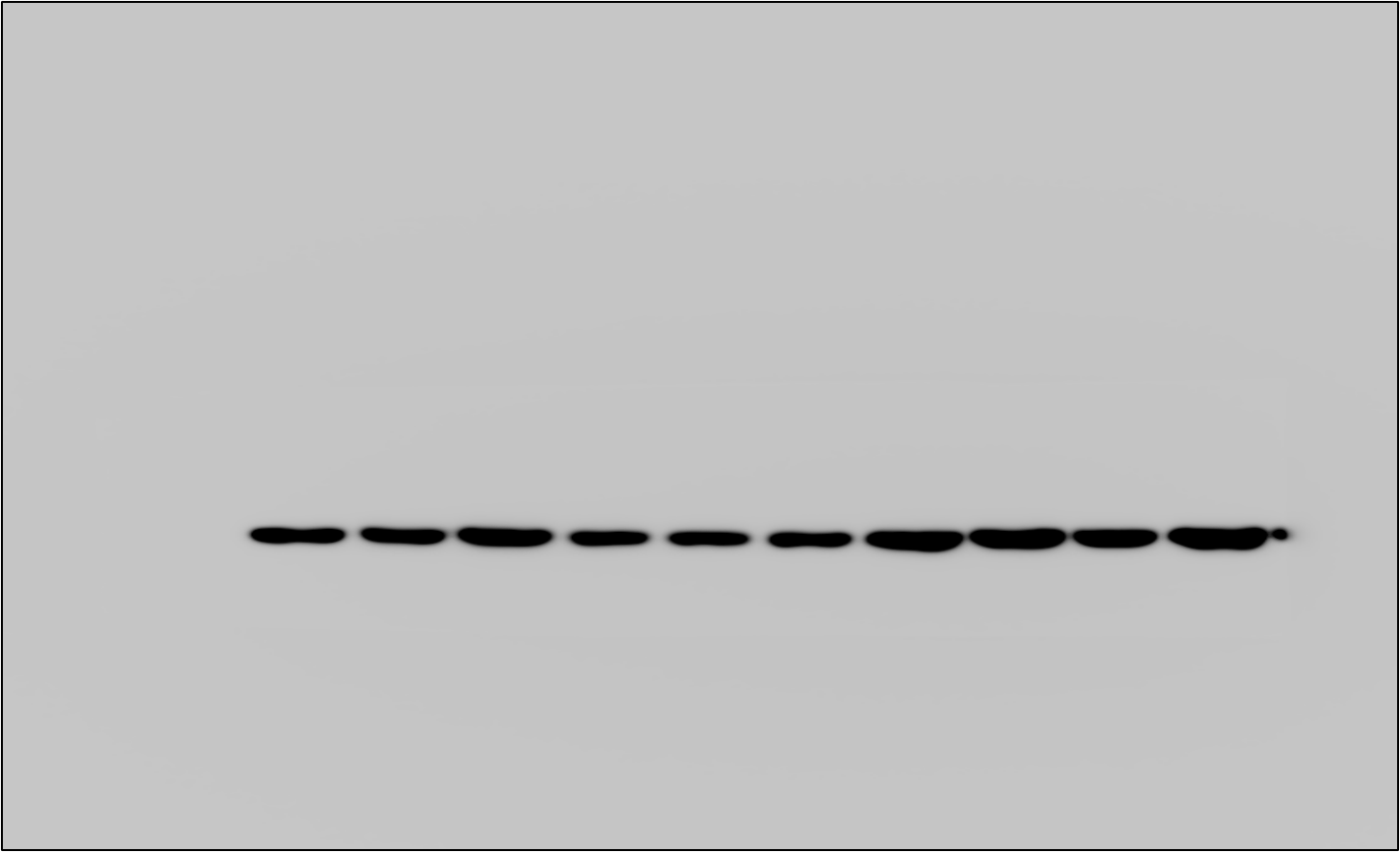

Supplement: Figure 8—source data 2. [file elife-108048-fig8-data2.zip › Figure 8/Figure 8 D-Actin.tif]

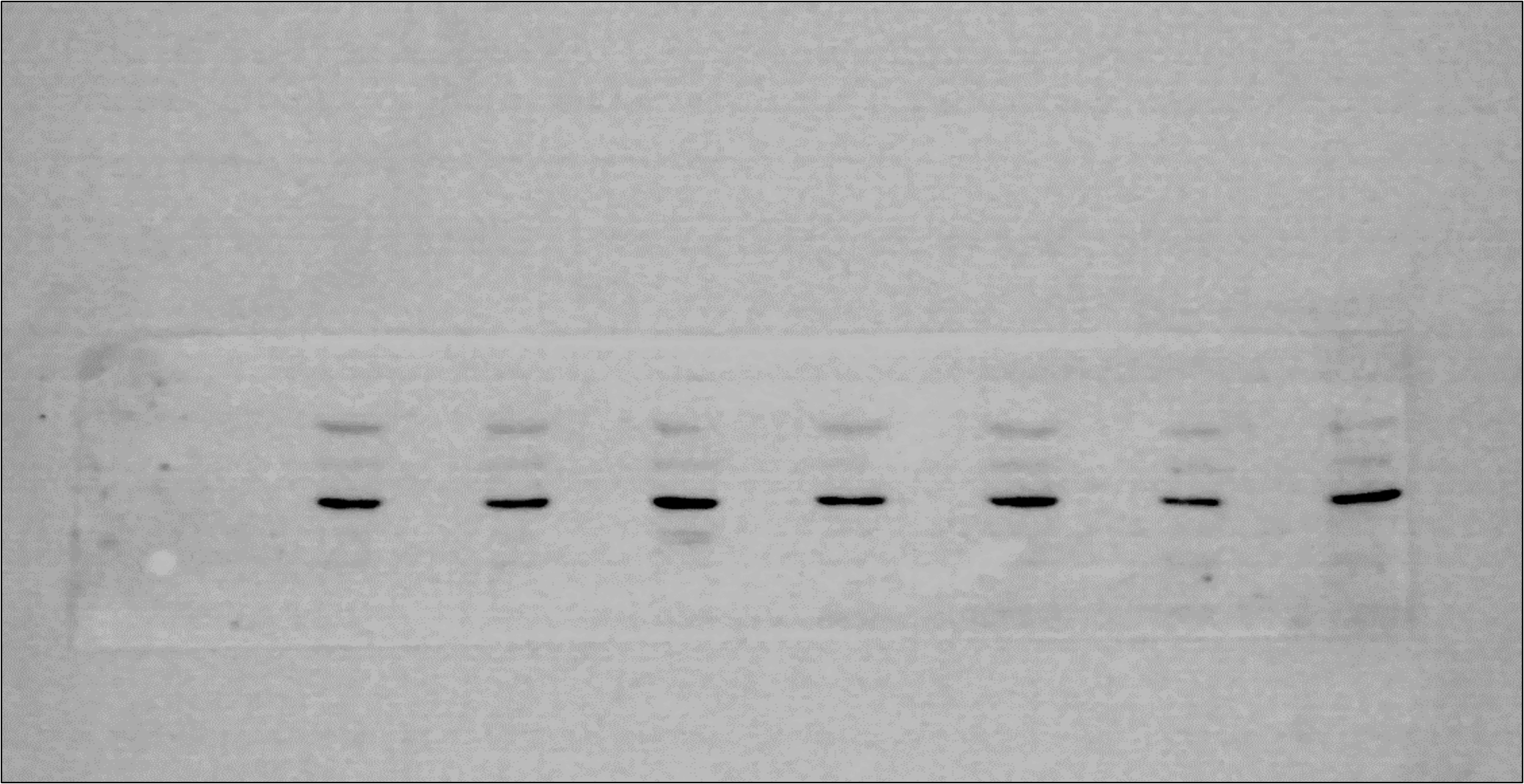

Supplement: Figure 8—source data 2. [file elife-108048-fig8-data2.zip › Figure 8/Figure 8 D-Flag.tif]

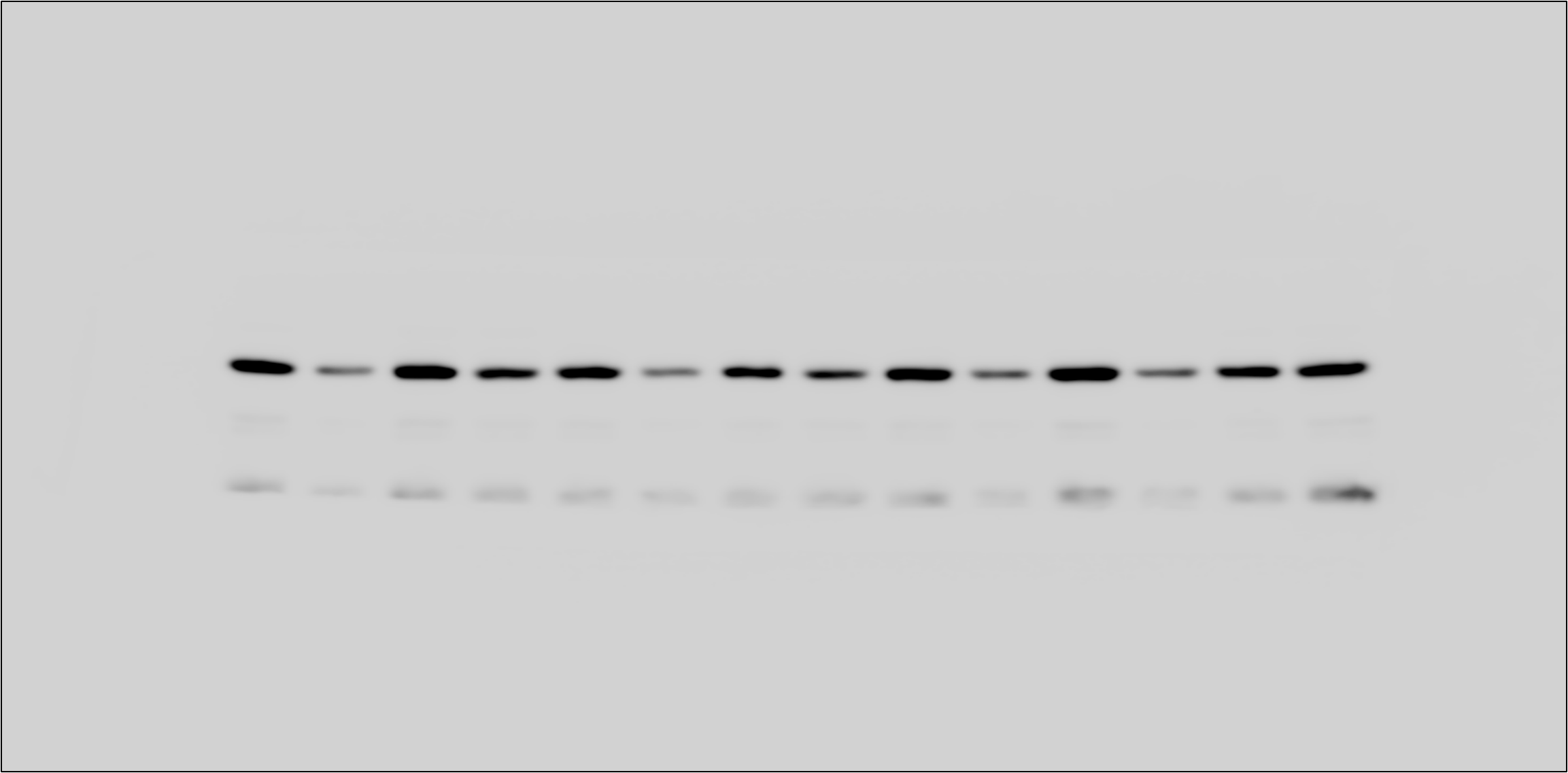

Supplement: Figure 8—source data 2. [file elife-108048-fig8-data2.zip › Figure 8/Figure 8 D-HA.tif]

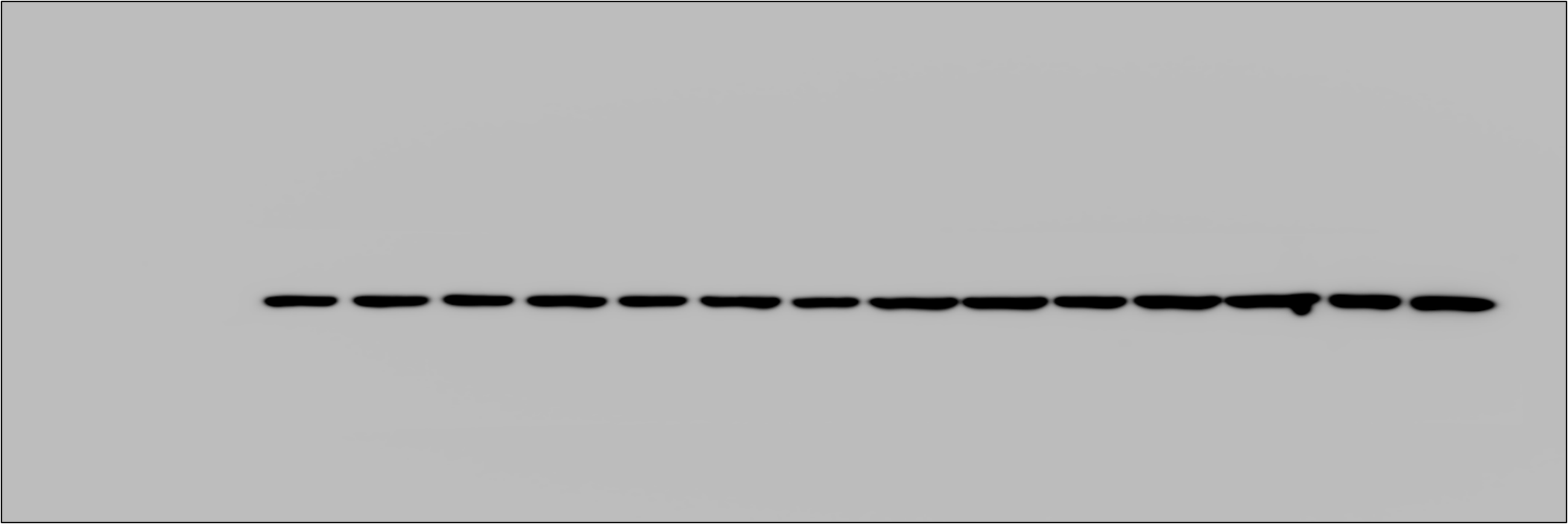

Supplement: Figure 8—source data 2. [file elife-108048-fig8-data2.zip › Figure 8/Figure 8 G-Actin.tif]

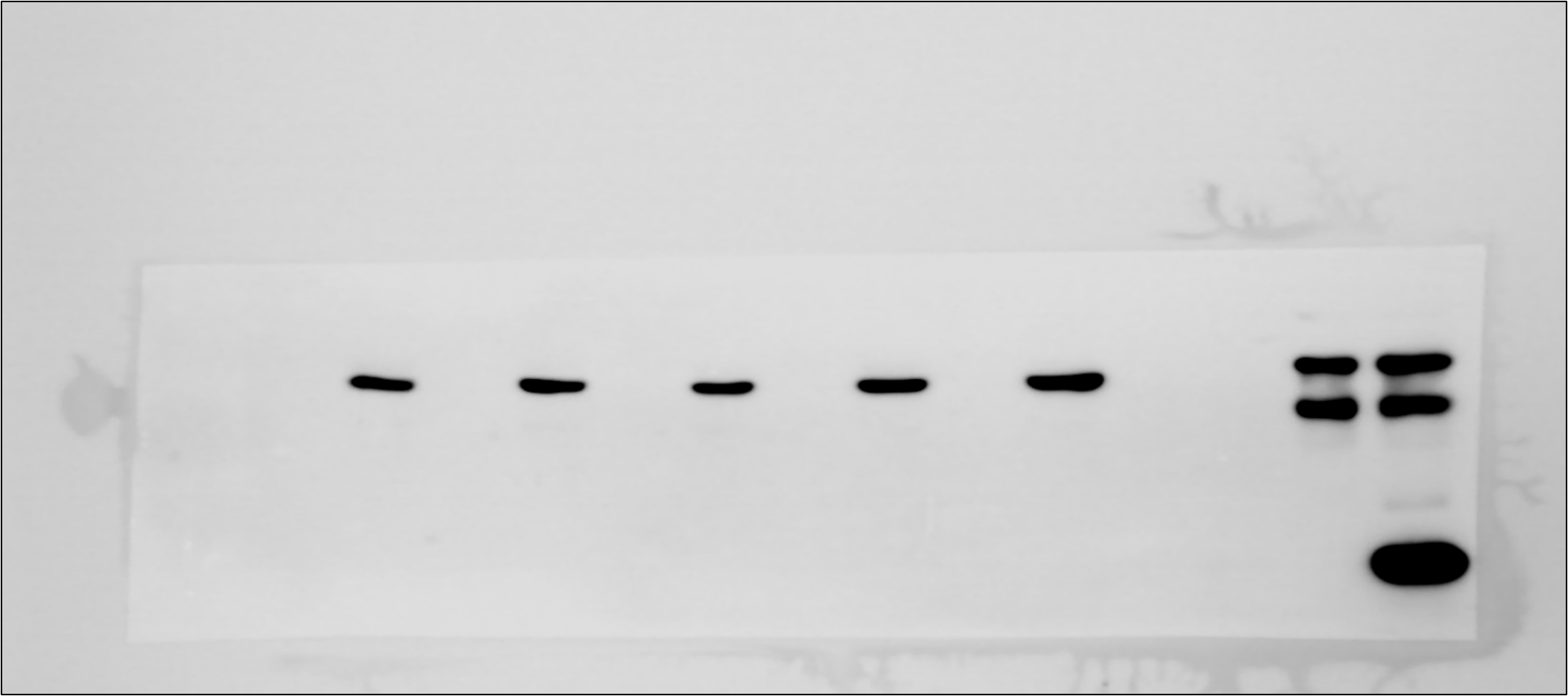

Supplement: Figure 8—source data 2. [file elife-108048-fig8-data2.zip › Figure 8/Figure 8 G-Flag.tif]

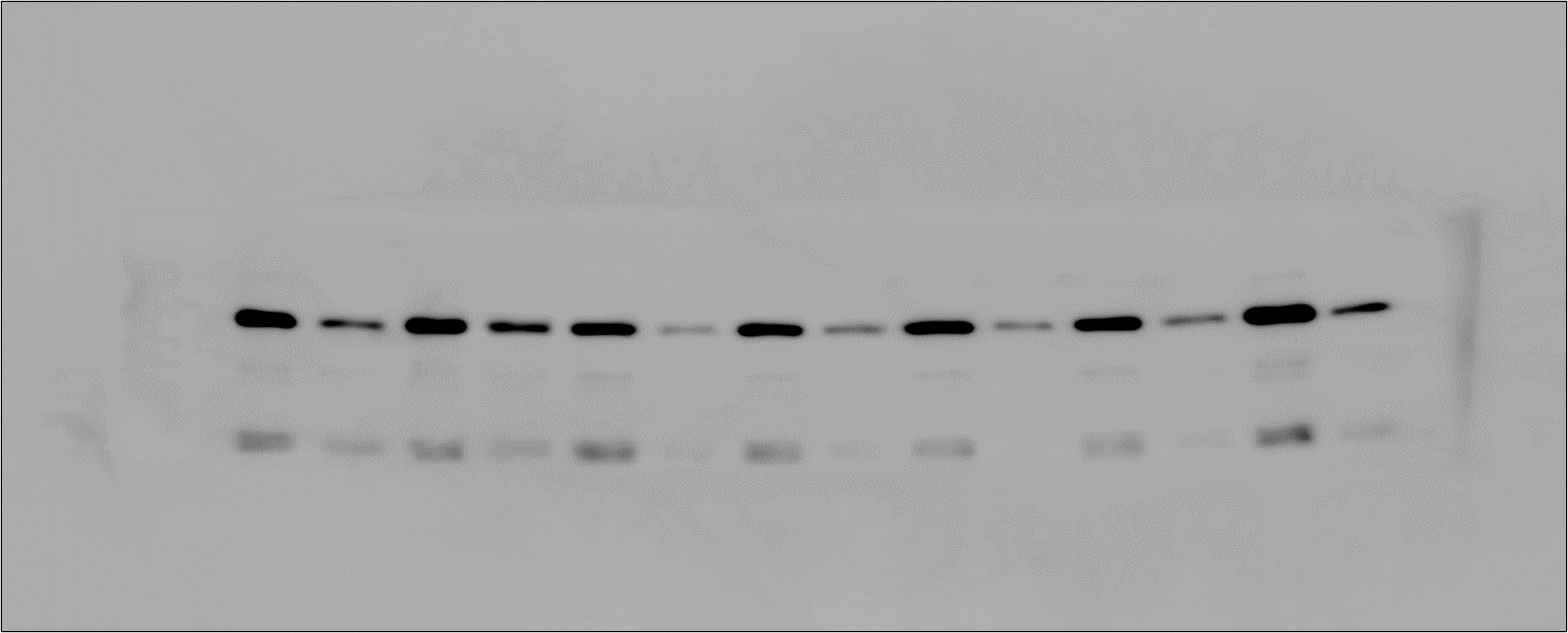

Supplement: Figure 8—source data 2. [file elife-108048-fig8-data2.zip › Figure 8/Figure 8 G-HA.tif]

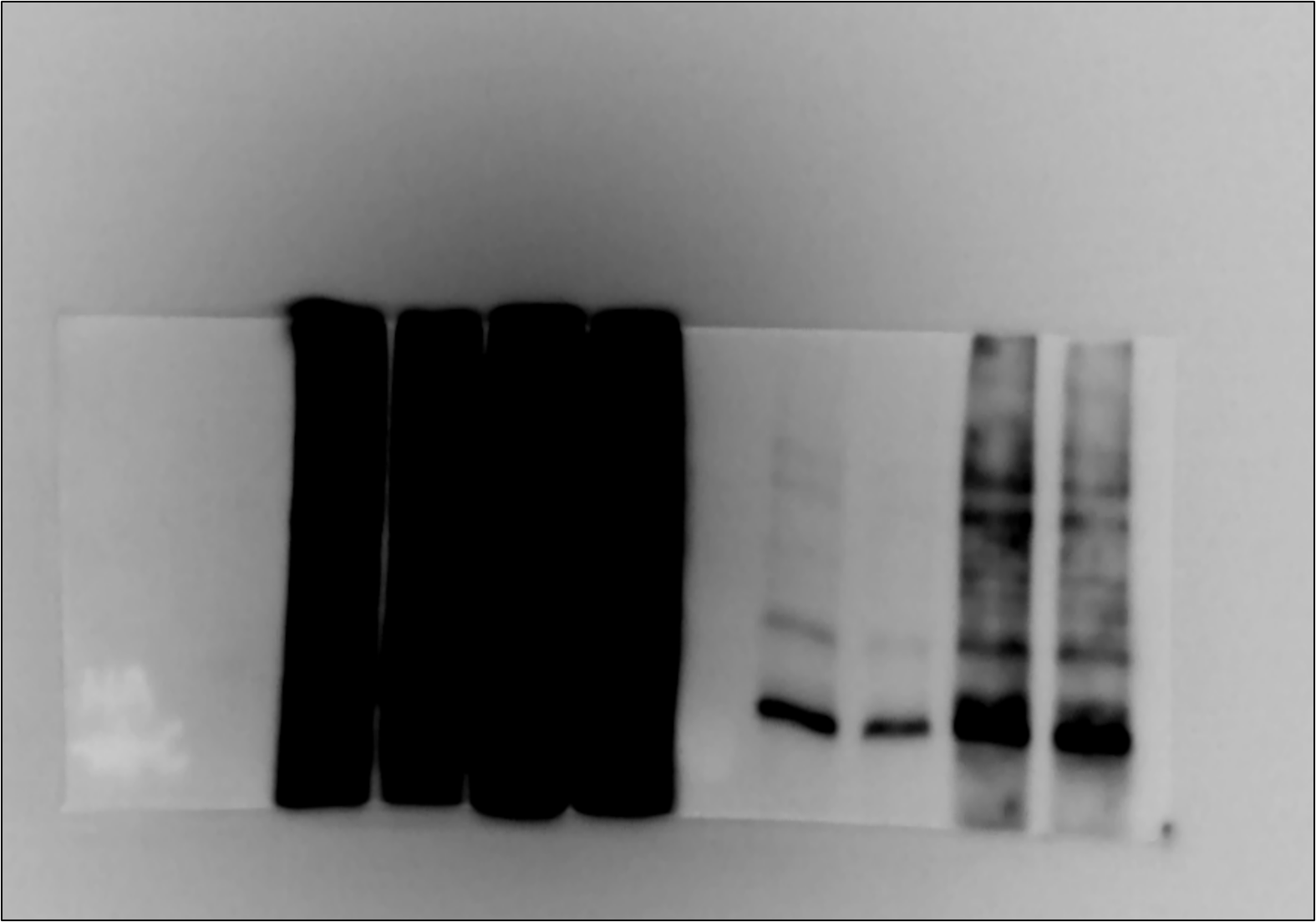

Supplement: Figure 8—source data 2. [file elife-108048-fig8-data2.zip › Figure 8/Figure 8 H-IP-HA.tif]

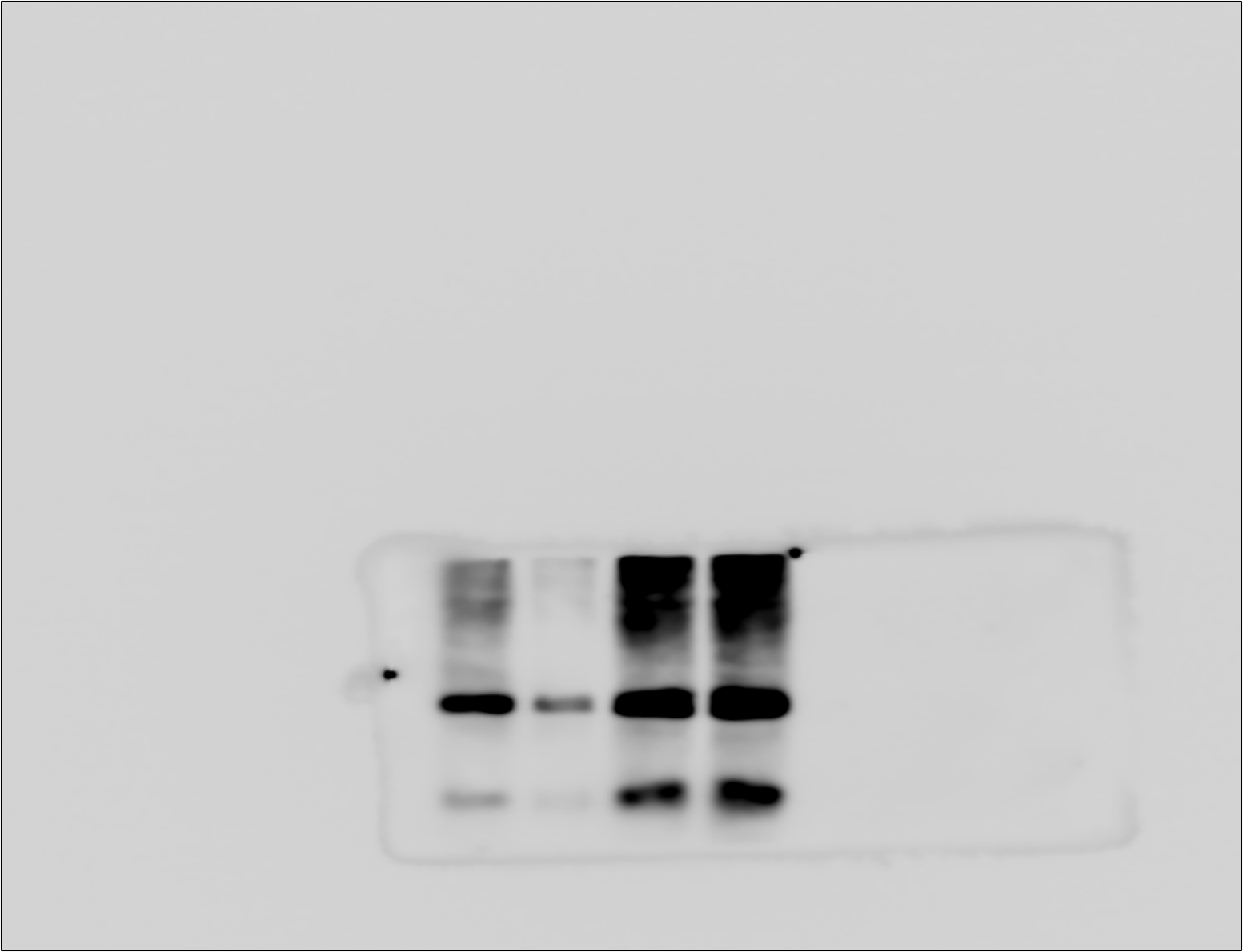

Supplement: Figure 8—source data 2. [file elife-108048-fig8-data2.zip › Figure 8/Figure 8 H-IP-Myc.tif]

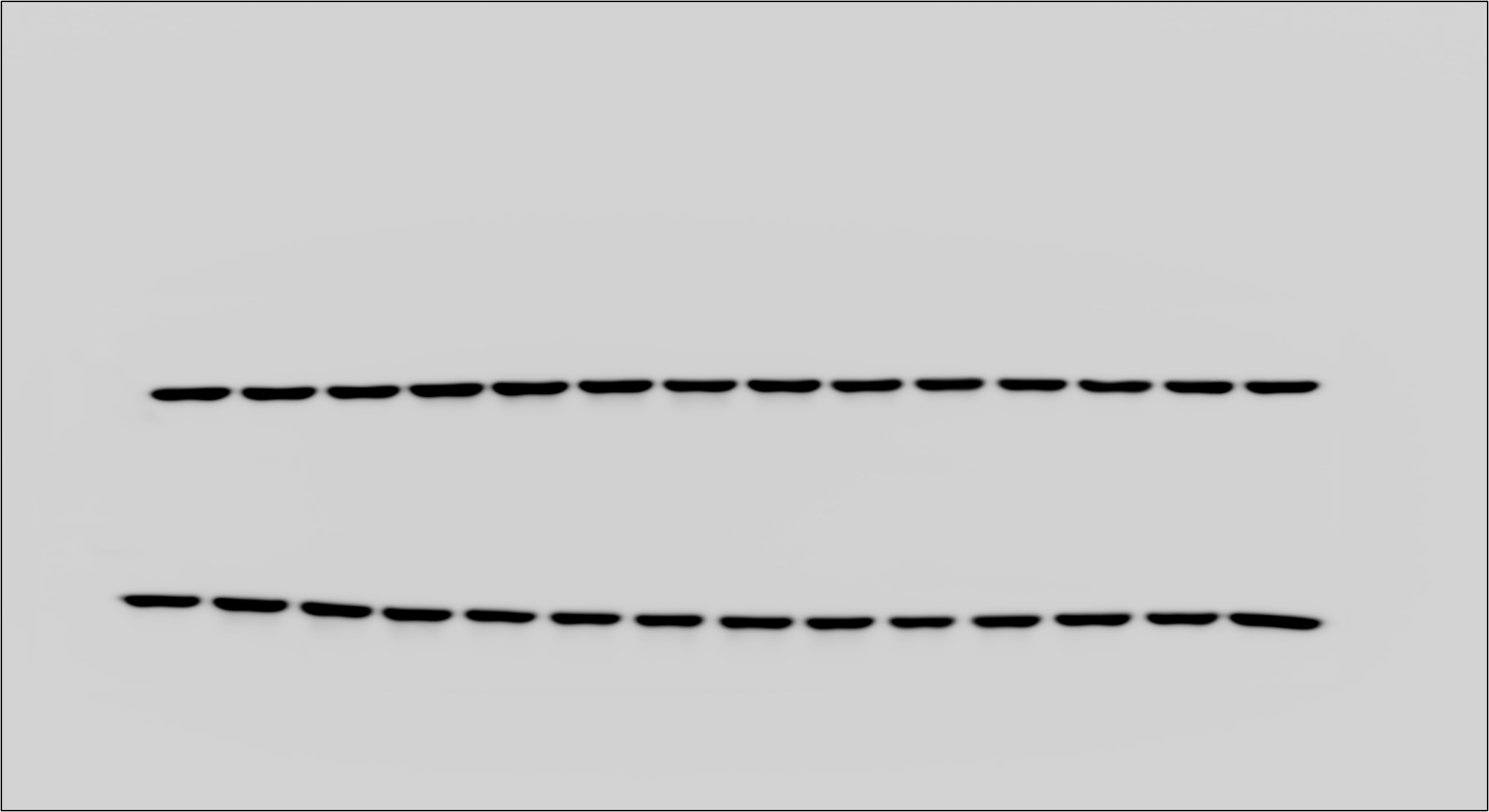

Supplement: Figure 8—source data 2. [file elife-108048-fig8-data2.zip › Figure 8/Figure 8 H-WCL-Actin.tif]

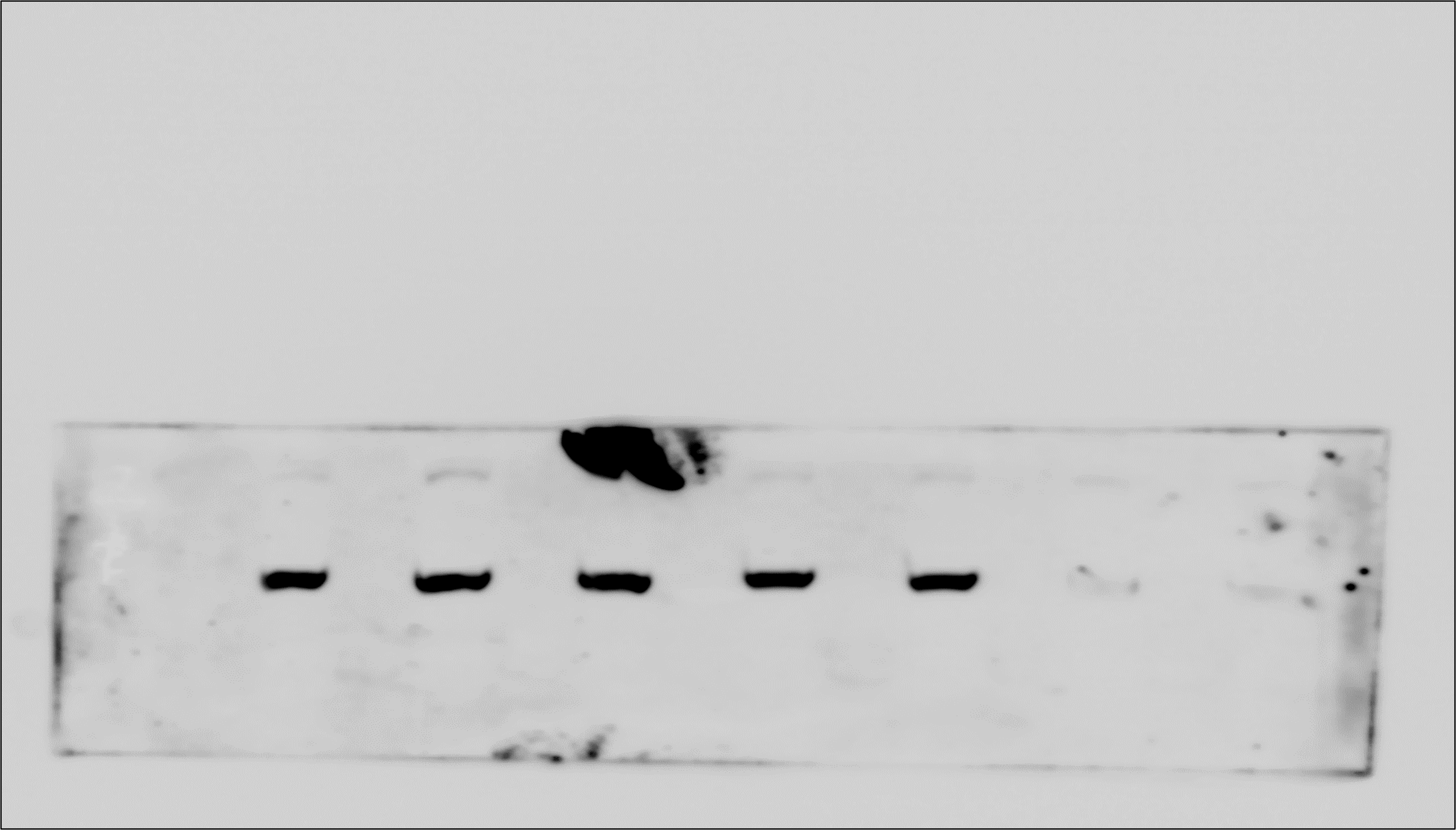

Supplement: Figure 8—source data 2. [file elife-108048-fig8-data2.zip › Figure 8/Figure 8 H-WCL-Flag.tif]

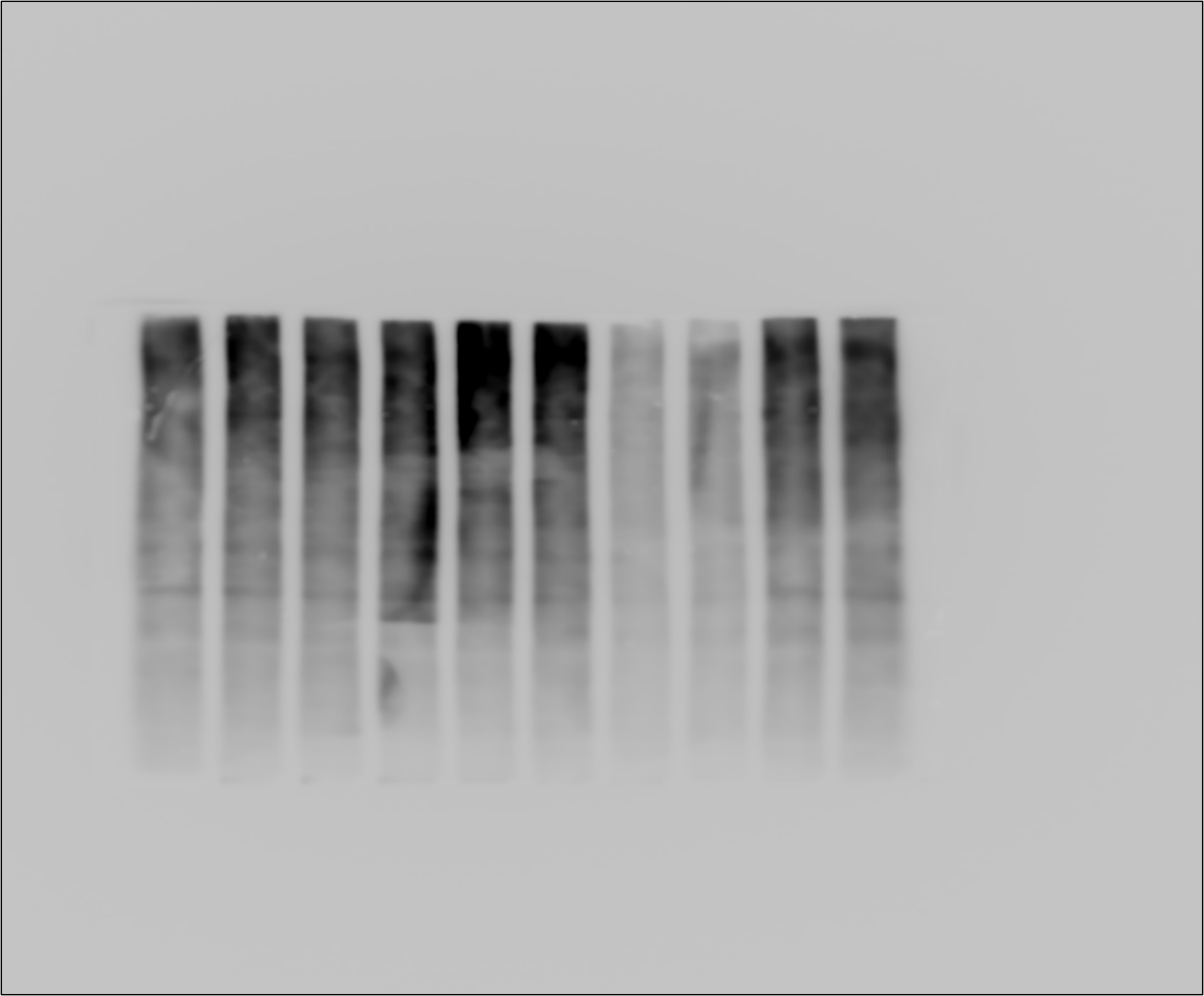

Supplement: Figure 8—source data 2. [file elife-108048-fig8-data2.zip › Figure 8/Figure 8 H-WCL-HA.tif]

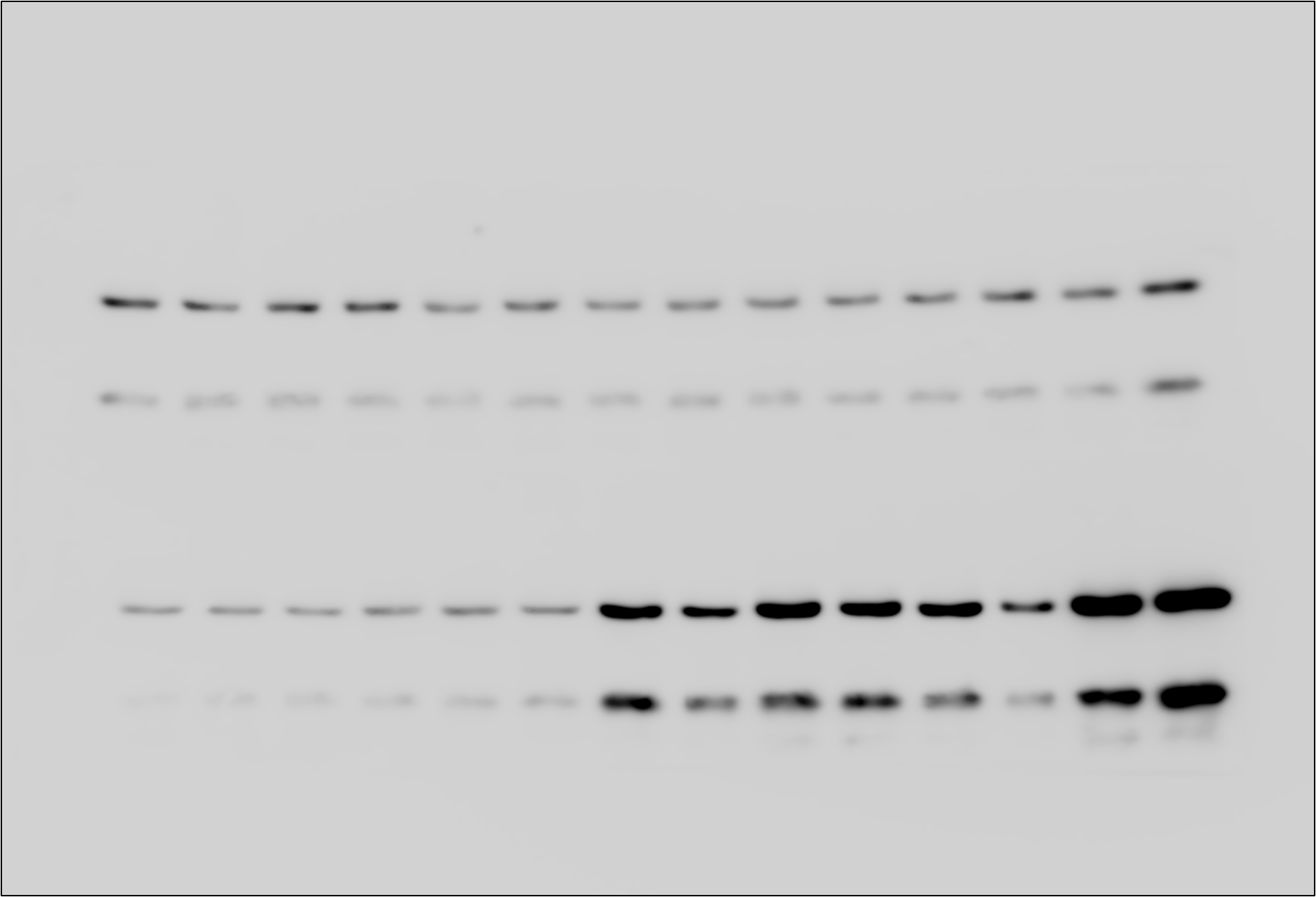

Supplement: Figure 8—source data 2. [file elife-108048-fig8-data2.zip › Figure 8/Figure 8 H-WCL-Myc.tif]

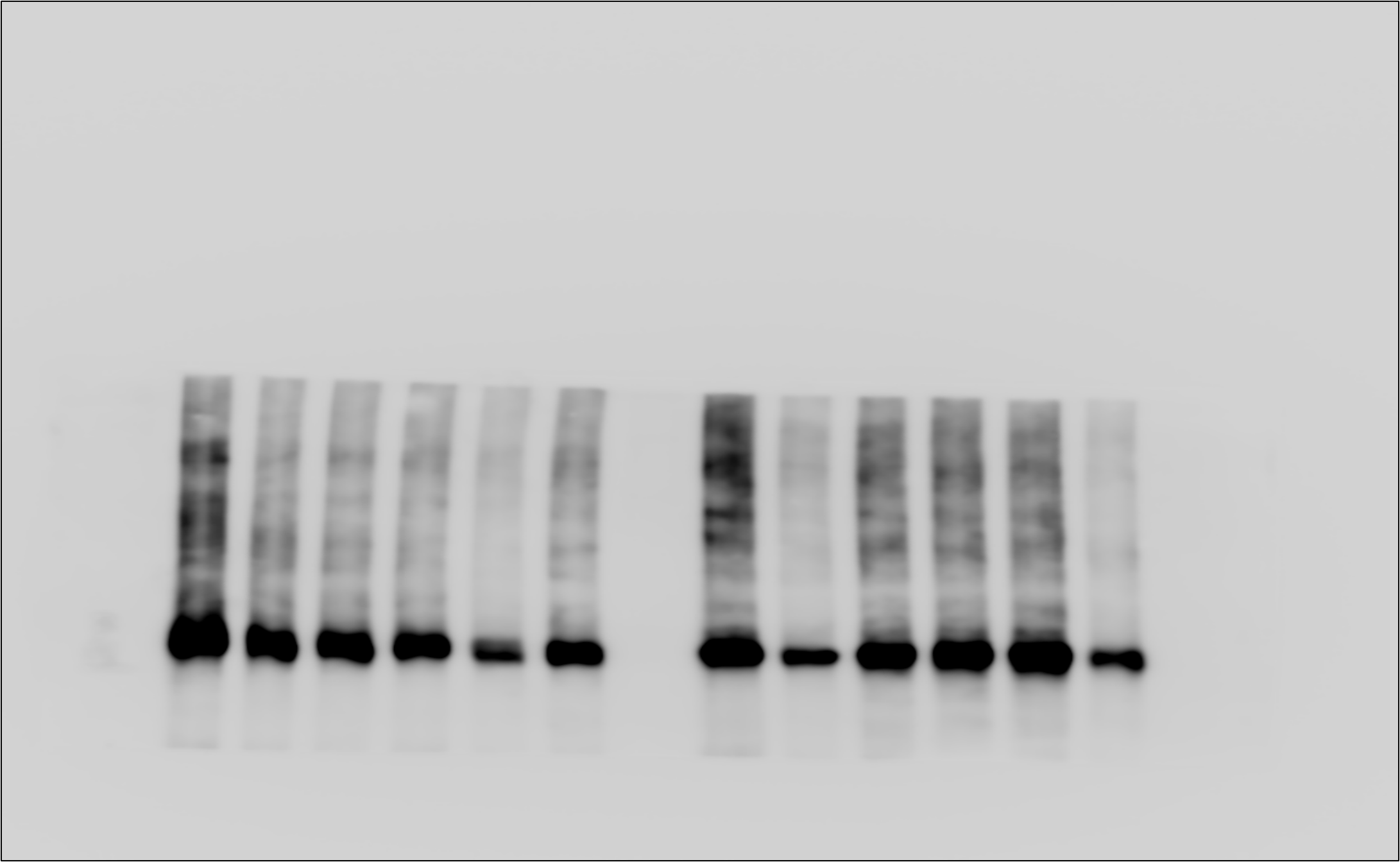

Supplement: Figure 8—source data 2. [file elife-108048-fig8-data2.zip › Figure 8/Figure 8 I-IP-HA.tif]

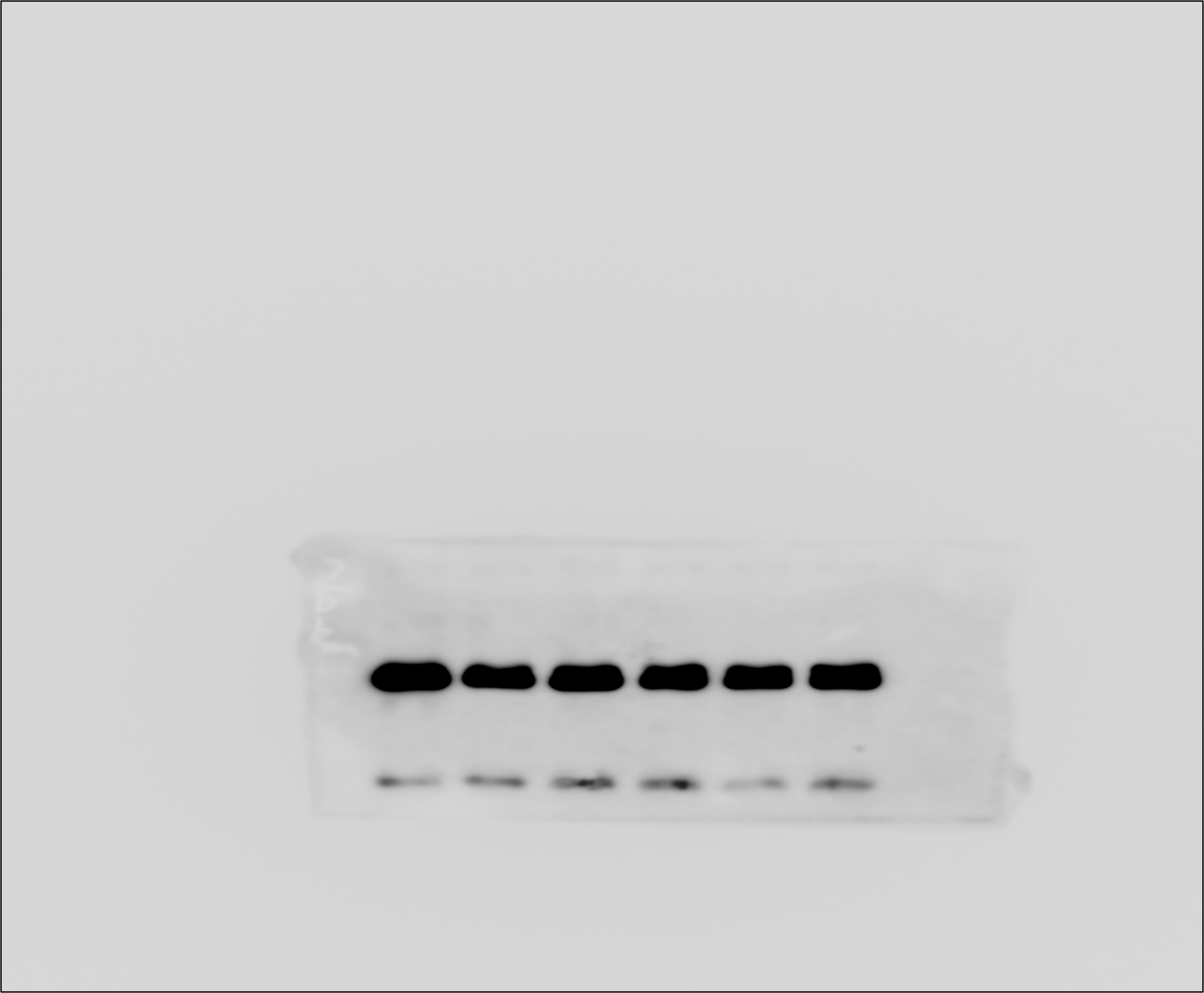

Supplement: Figure 8—source data 2. [file elife-108048-fig8-data2.zip › Figure 8/Figure 8 I-IP-Myc.tif]

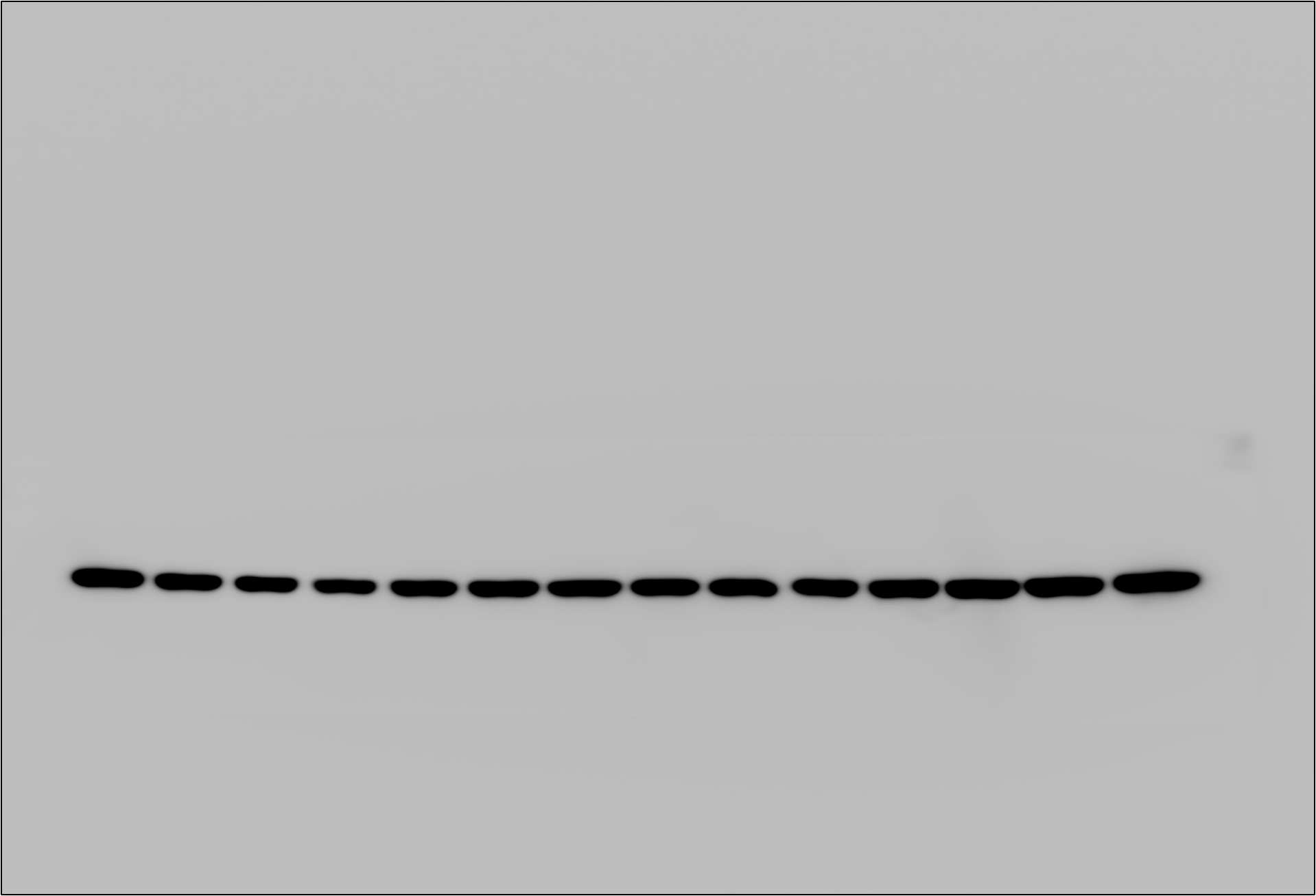

Supplement: Figure 8—source data 2. [file elife-108048-fig8-data2.zip › Figure 8/Figure 8 I-WCL-Actin.tif]

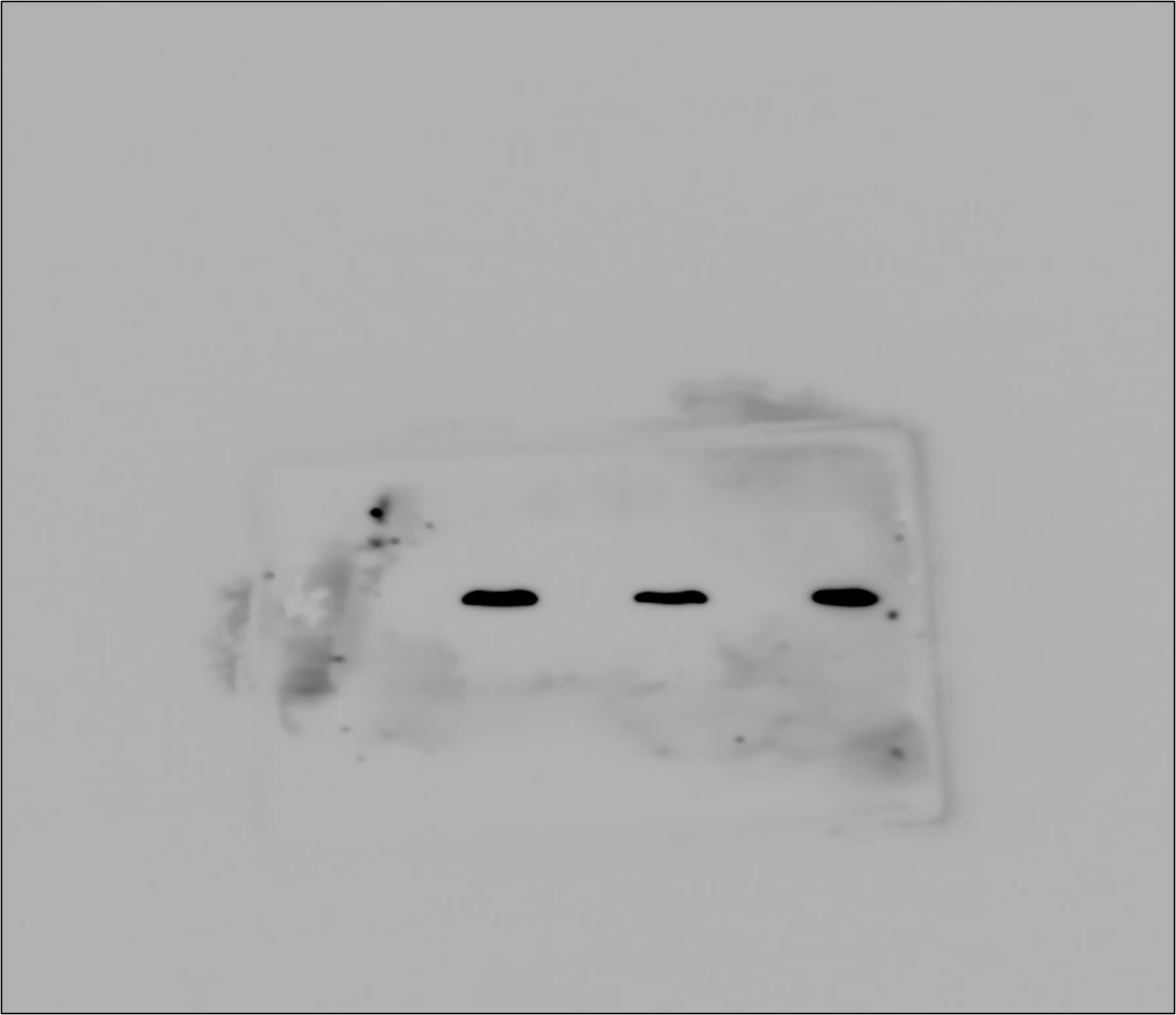

Supplement: Figure 8—source data 2. [file elife-108048-fig8-data2.zip › Figure 8/Figure 8 I-WCL-Flag.tif]

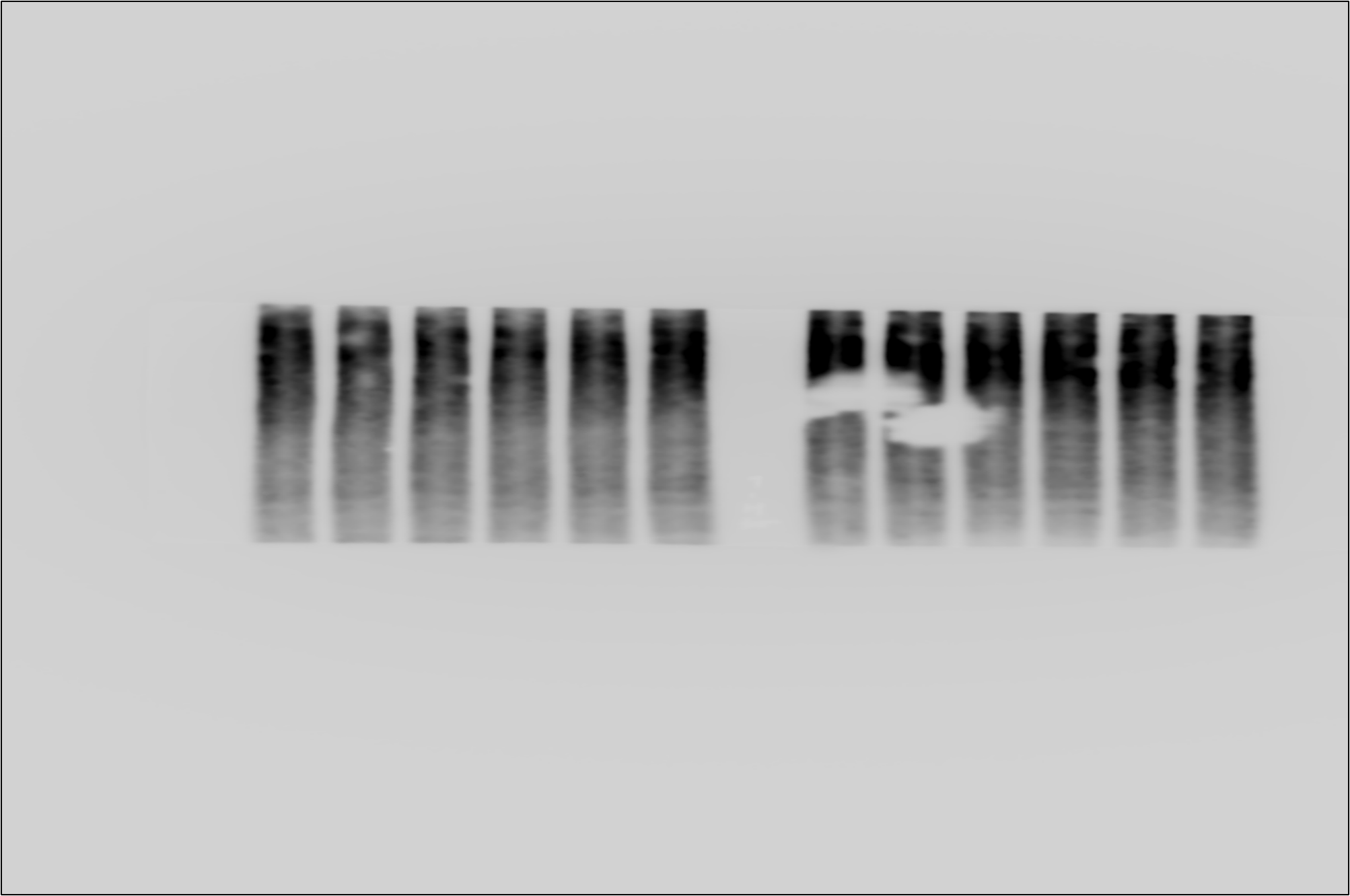

Supplement: Figure 8—source data 2. [file elife-108048-fig8-data2.zip › Figure 8/Figure 8 I-WCL-HA.tif]

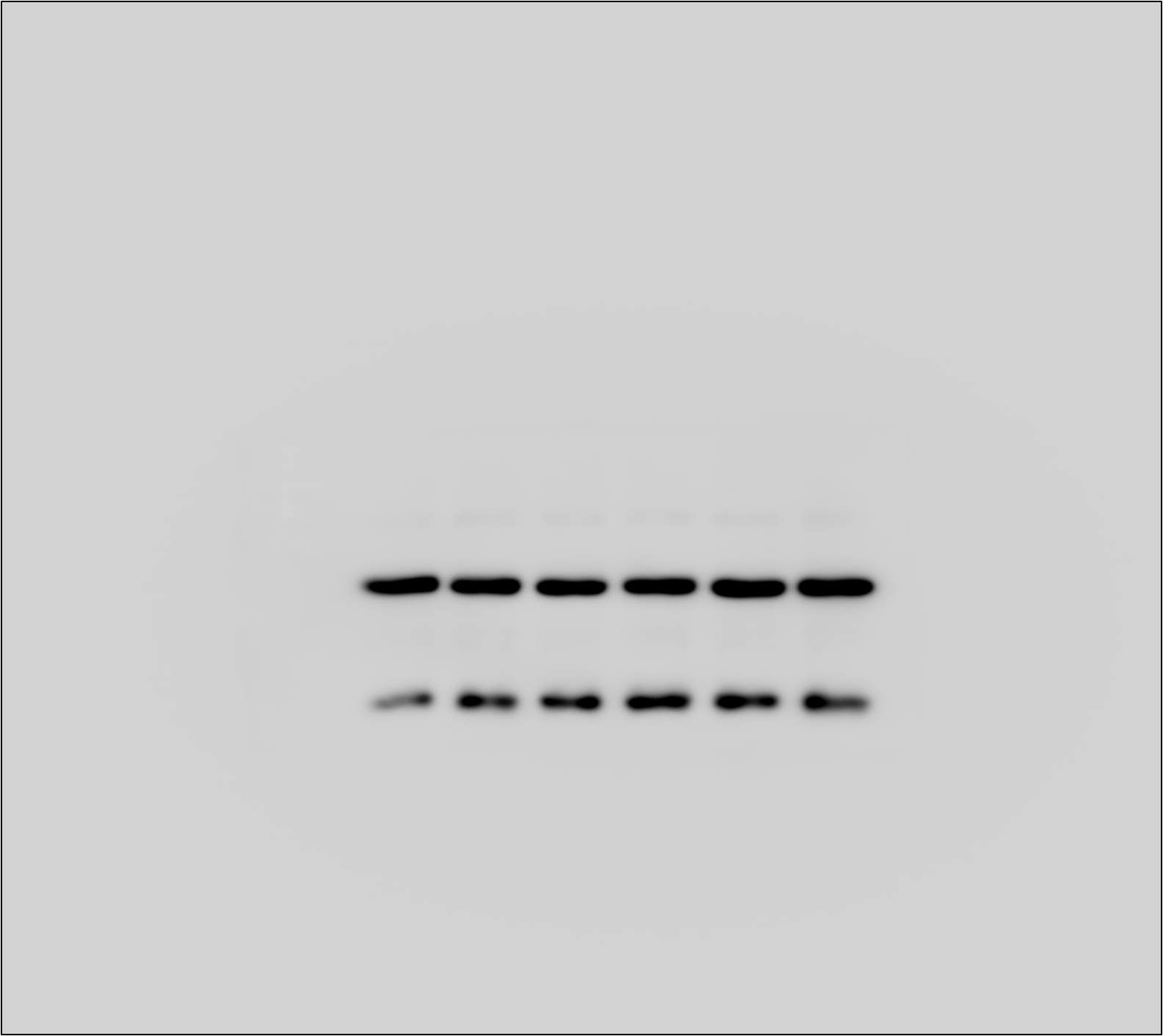

Supplement: Figure 8—source data 2. [file elife-108048-fig8-data2.zip › Figure 8/Figure 8 I-WCL-Myc.tif]

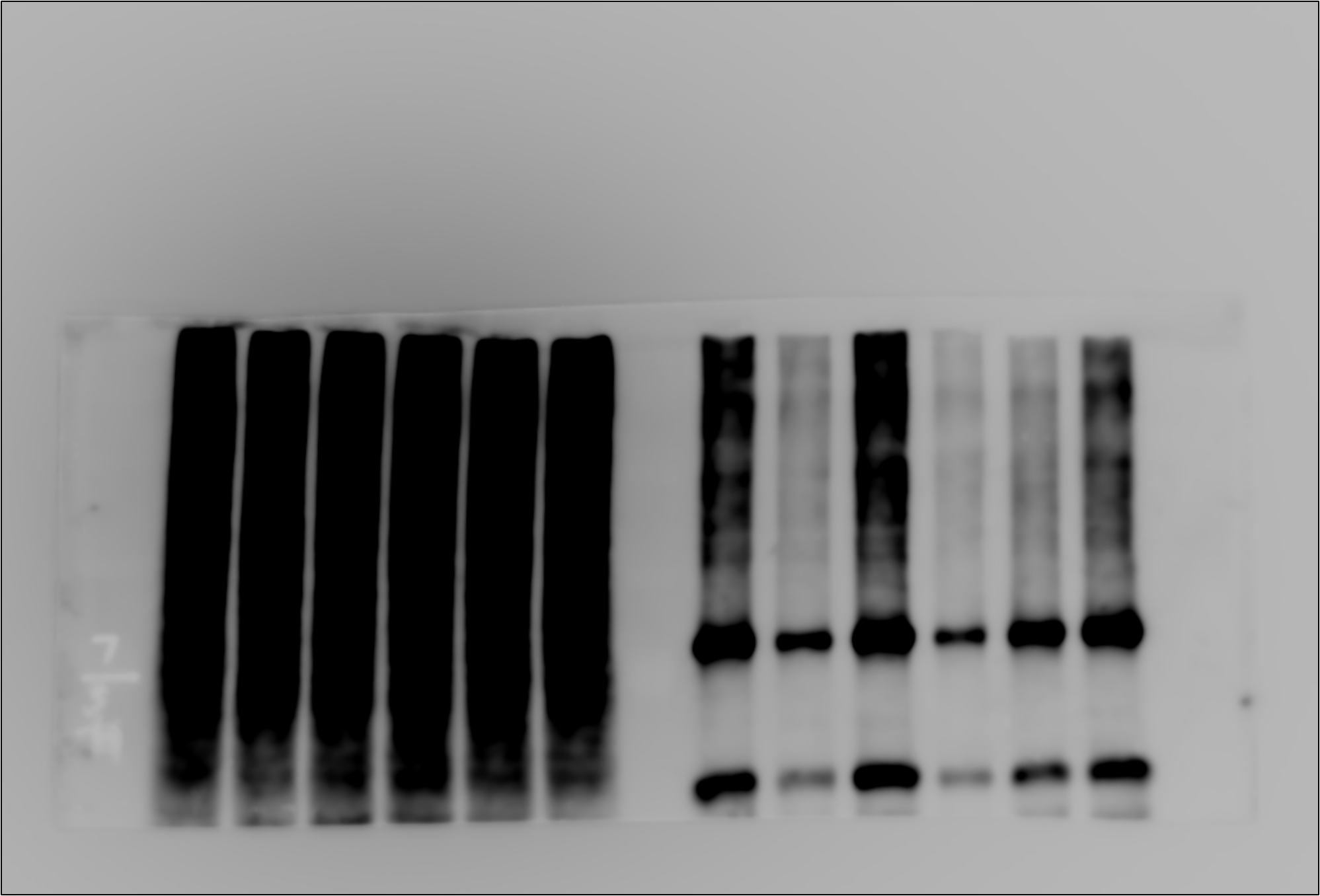

Supplement: Figure 8—source data 2. [file elife-108048-fig8-data2.zip › Figure 8/Figure 8 J-IP-HA.tif]

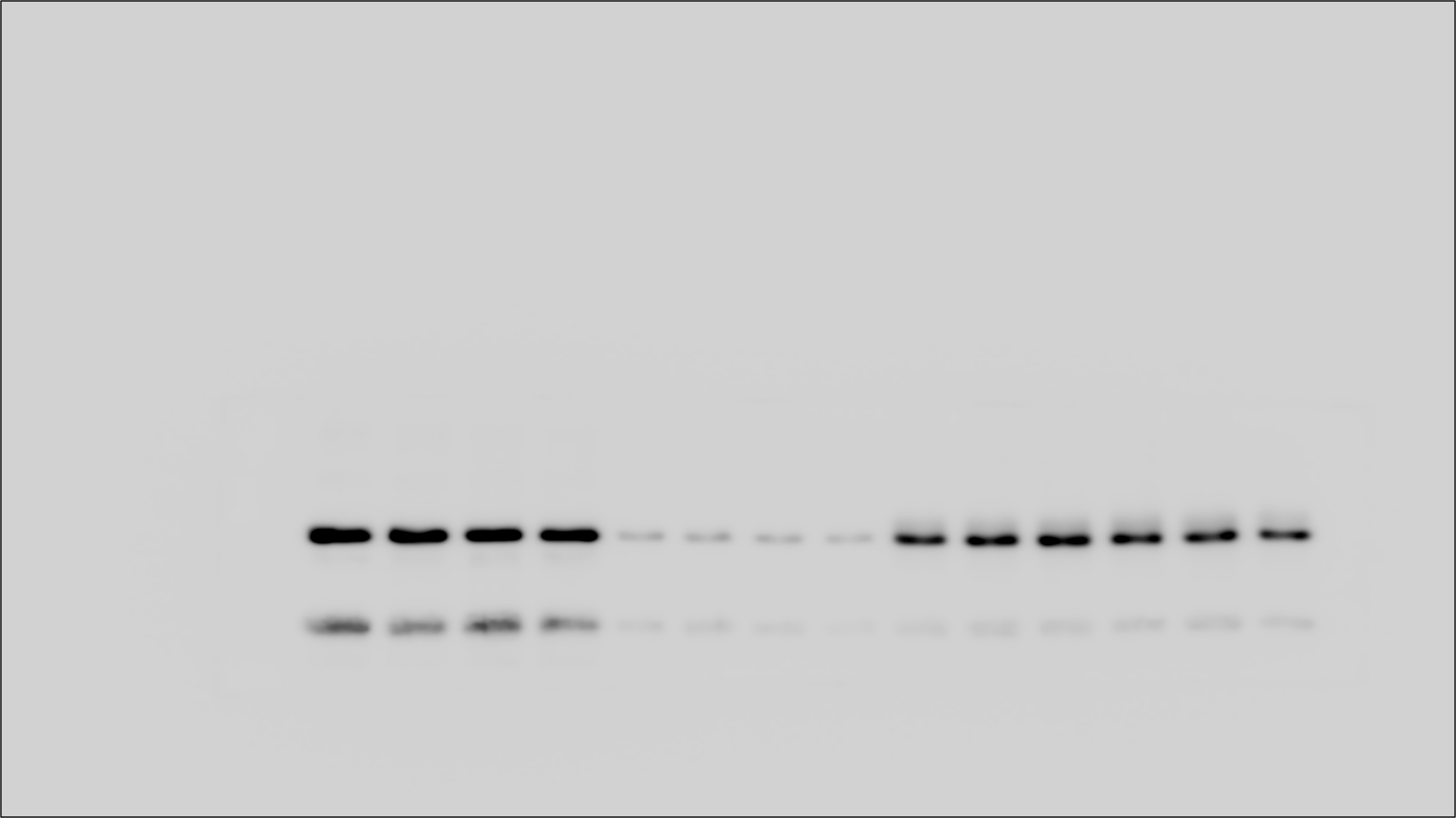

Supplement: Figure 8—source data 2. [file elife-108048-fig8-data2.zip › Figure 8/Figure 8 J-IP-Myc.tif]

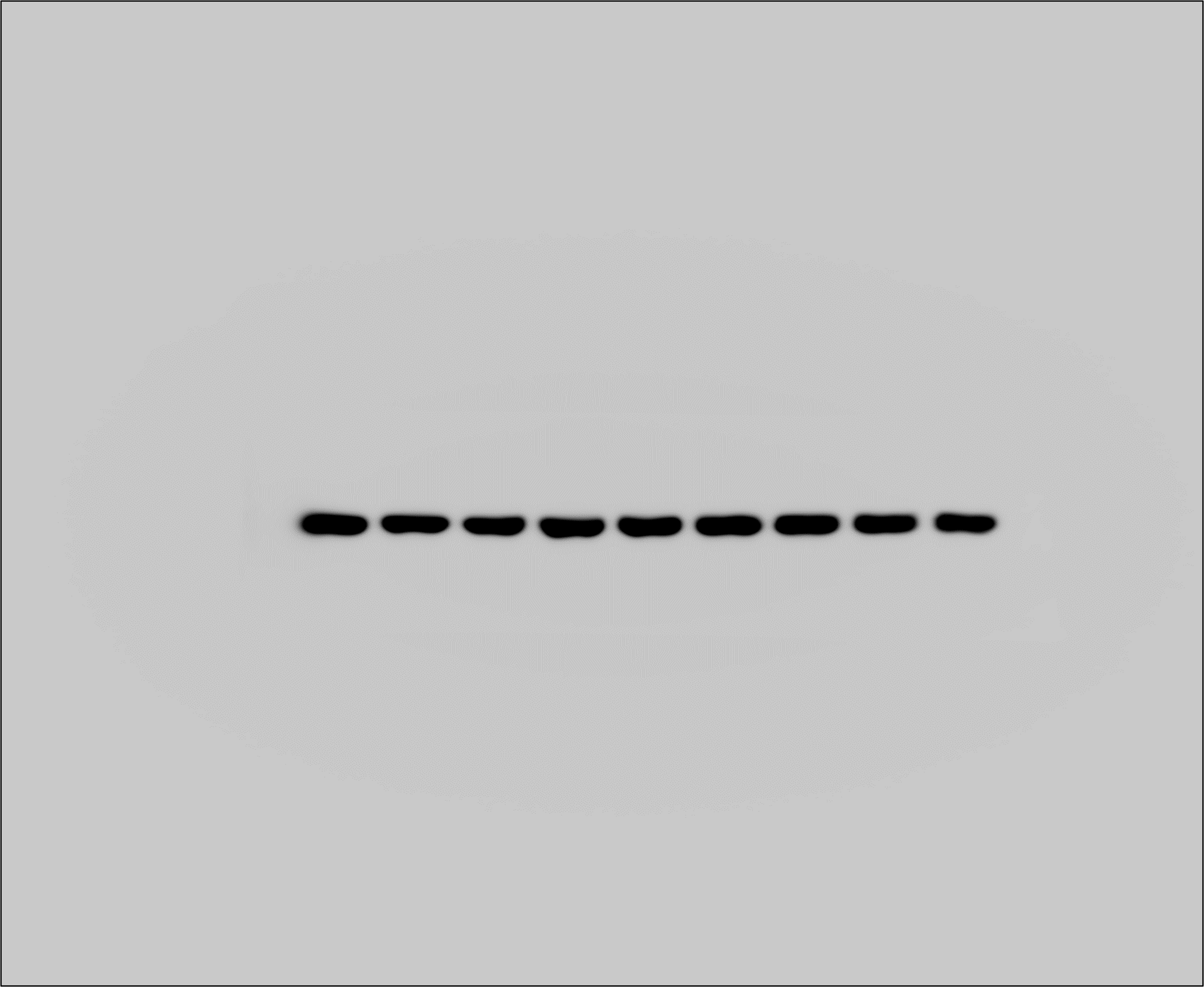

Supplement: Figure 8—source data 2. [file elife-108048-fig8-data2.zip › Figure 8/Figure 8 J-WCL-Actin.tif]

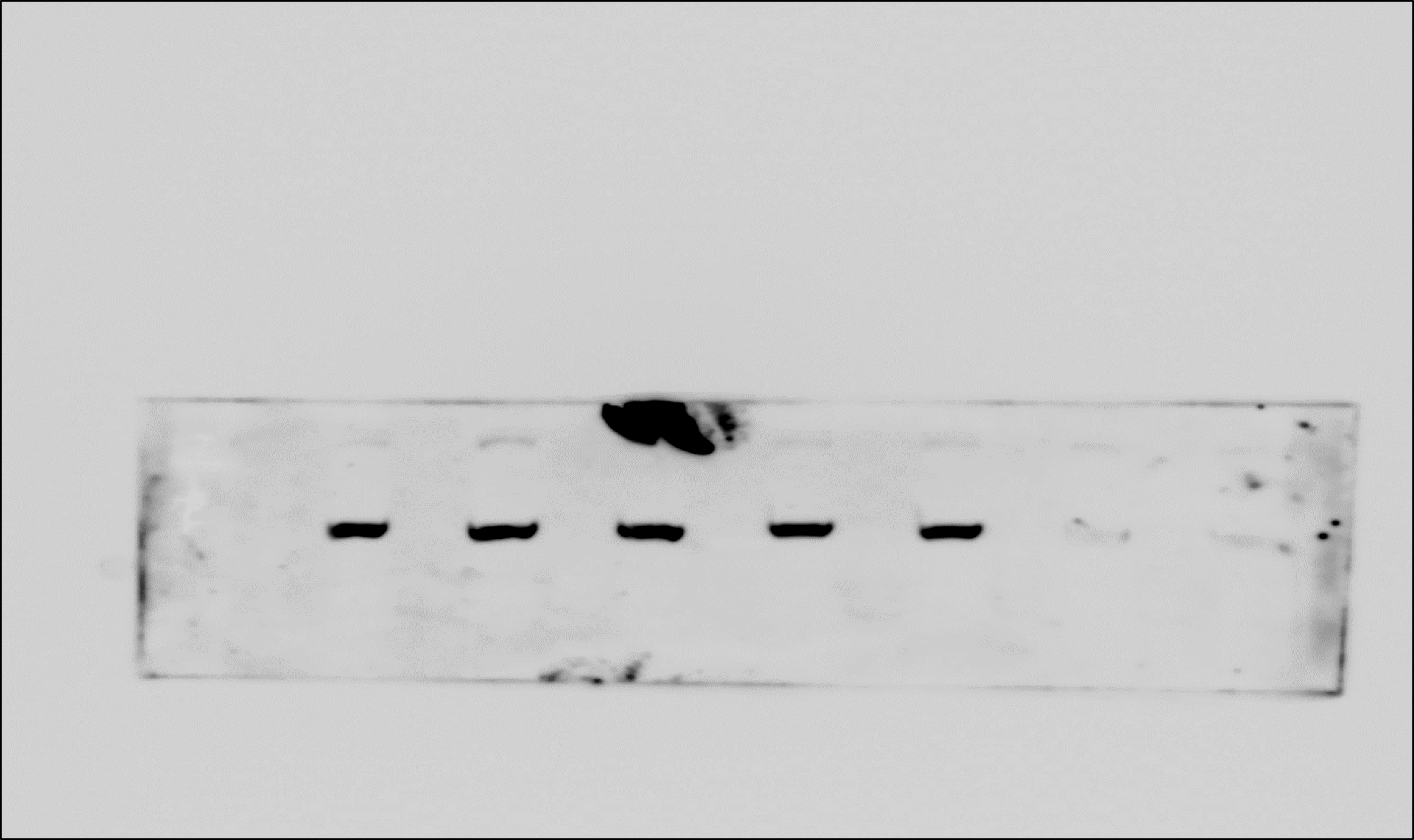

Supplement: Figure 8—source data 2. [file elife-108048-fig8-data2.zip › Figure 8/Figure 8 J-WCL-Flag.tif]

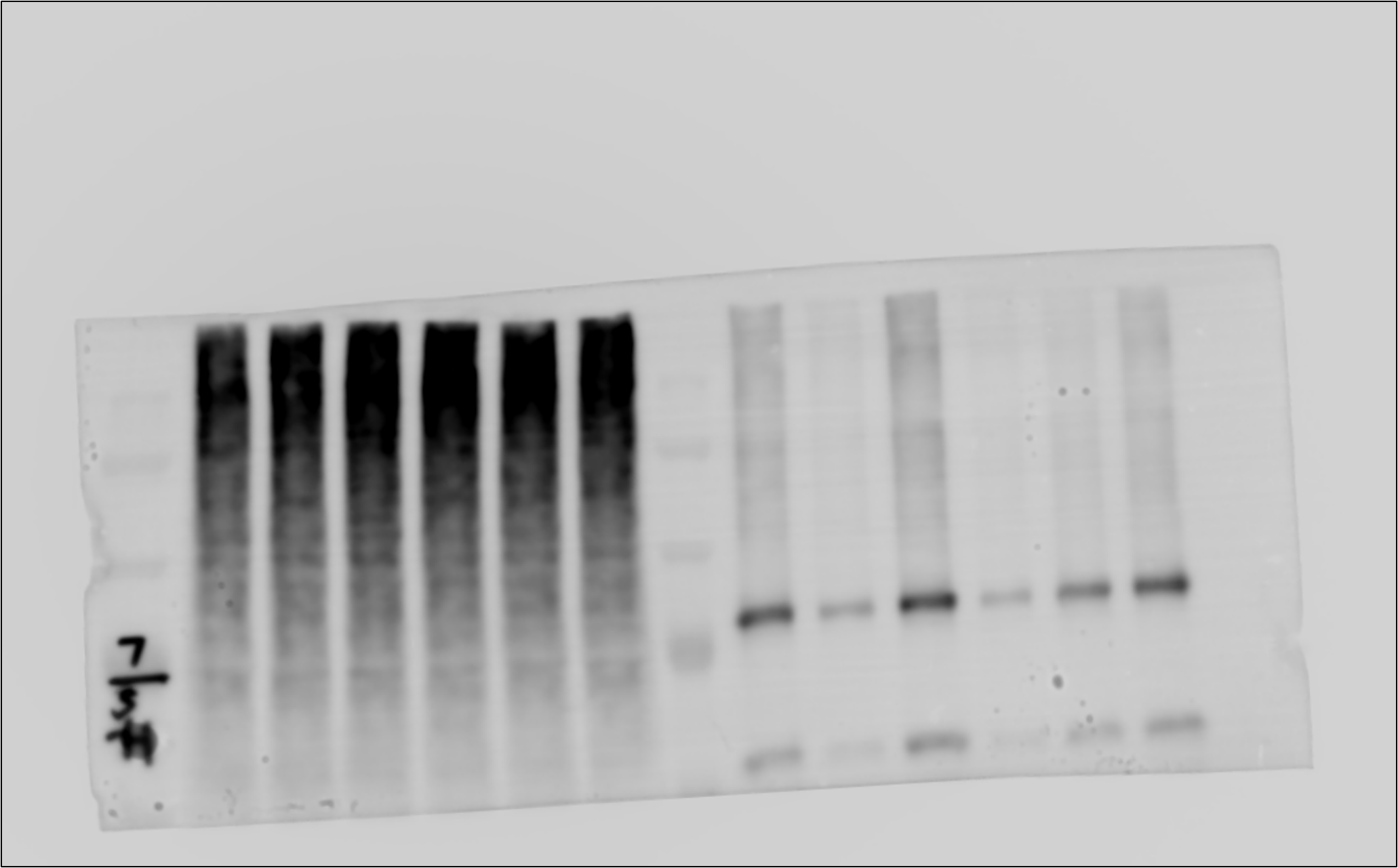

Supplement: Figure 8—source data 2. [file elife-108048-fig8-data2.zip › Figure 8/Figure 8 J-WCL-HA.tif]

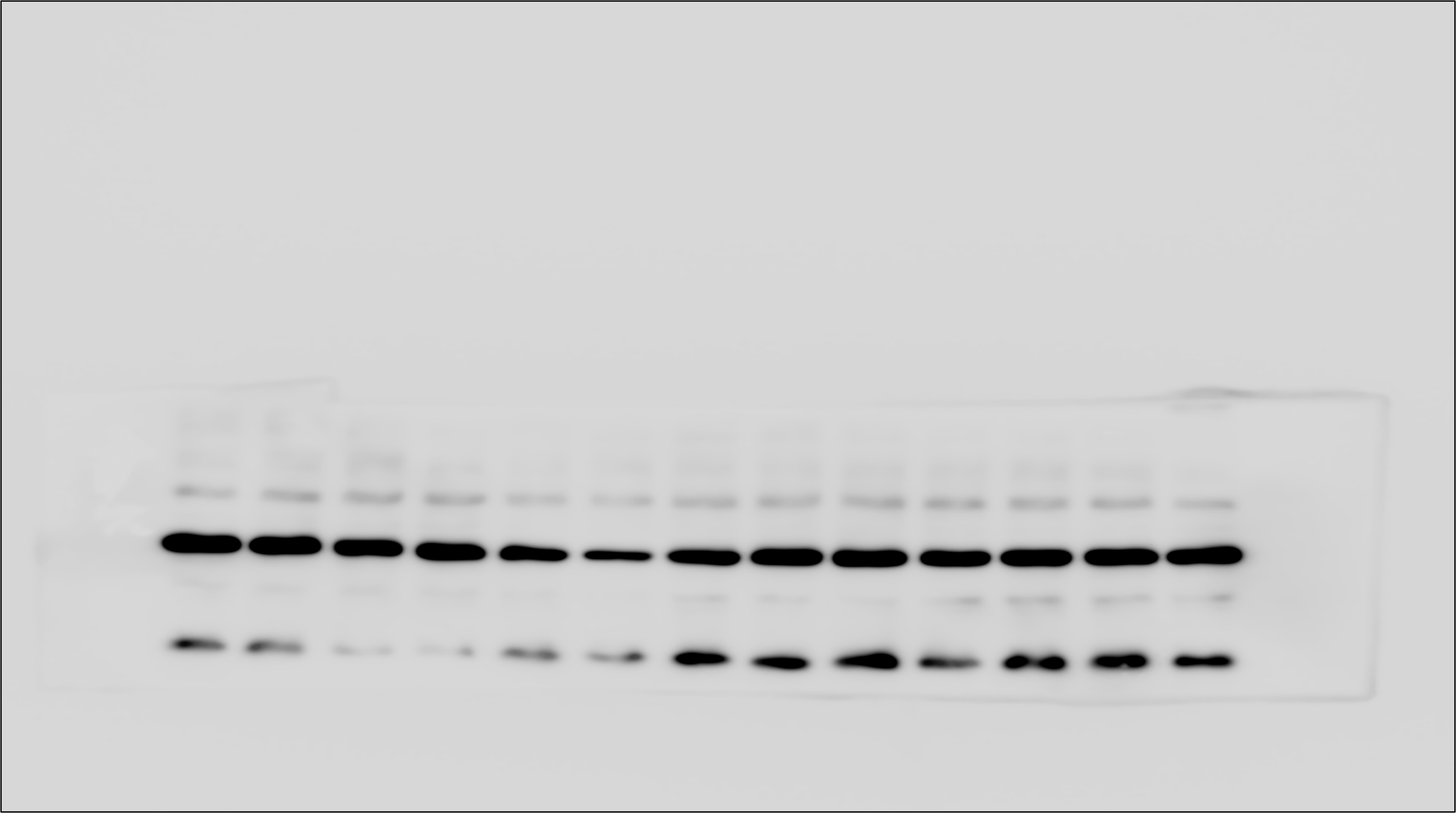

Supplement: Figure 8—source data 2. [file elife-108048-fig8-data2.zip › Figure 8/Figure 8 J-WCL-Myc.tif]

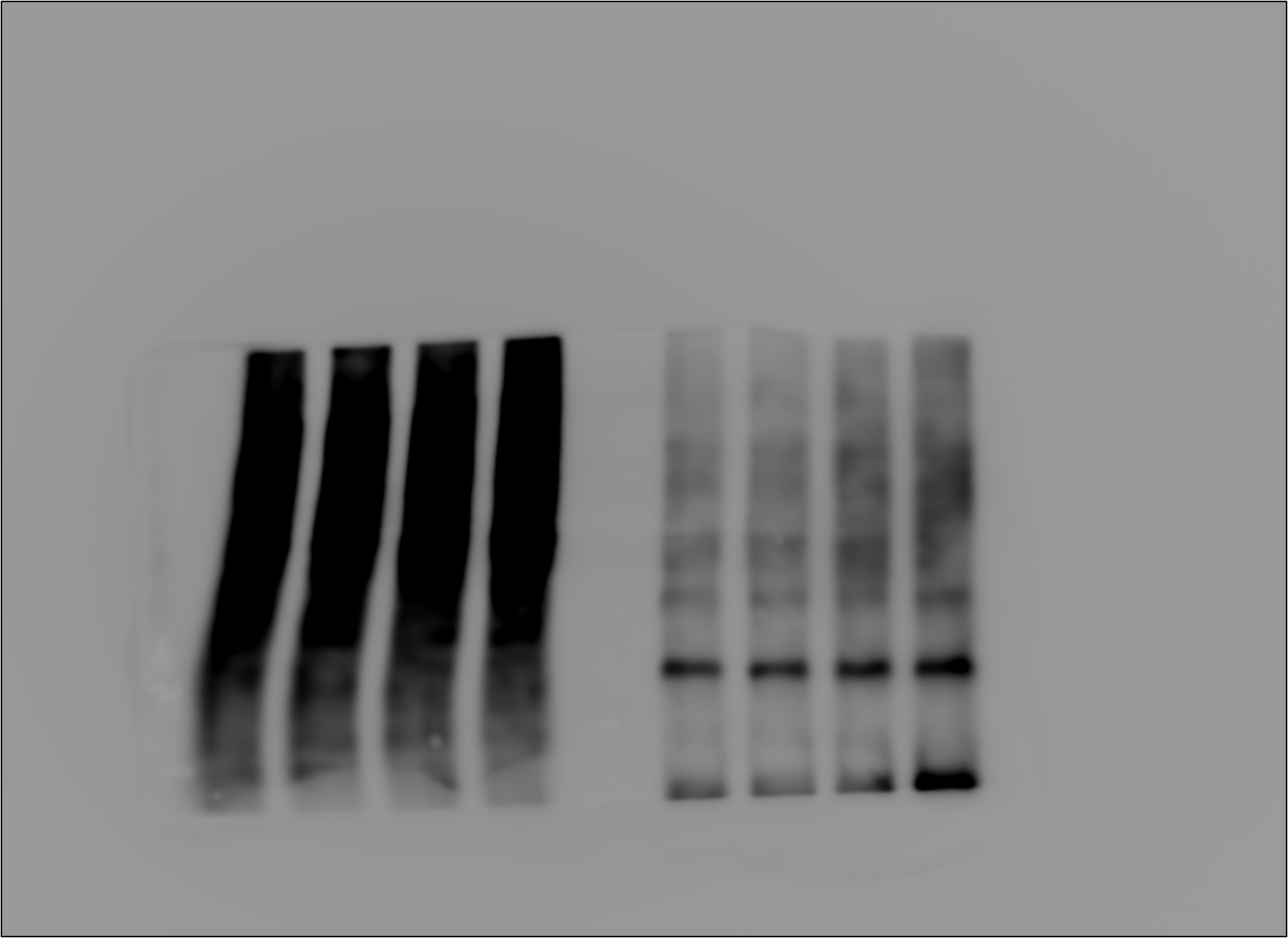

Supplement: Figure 8—source data 2. [file elife-108048-fig8-data2.zip › Figure 8/Figure 8 K-IP-HA.tif]

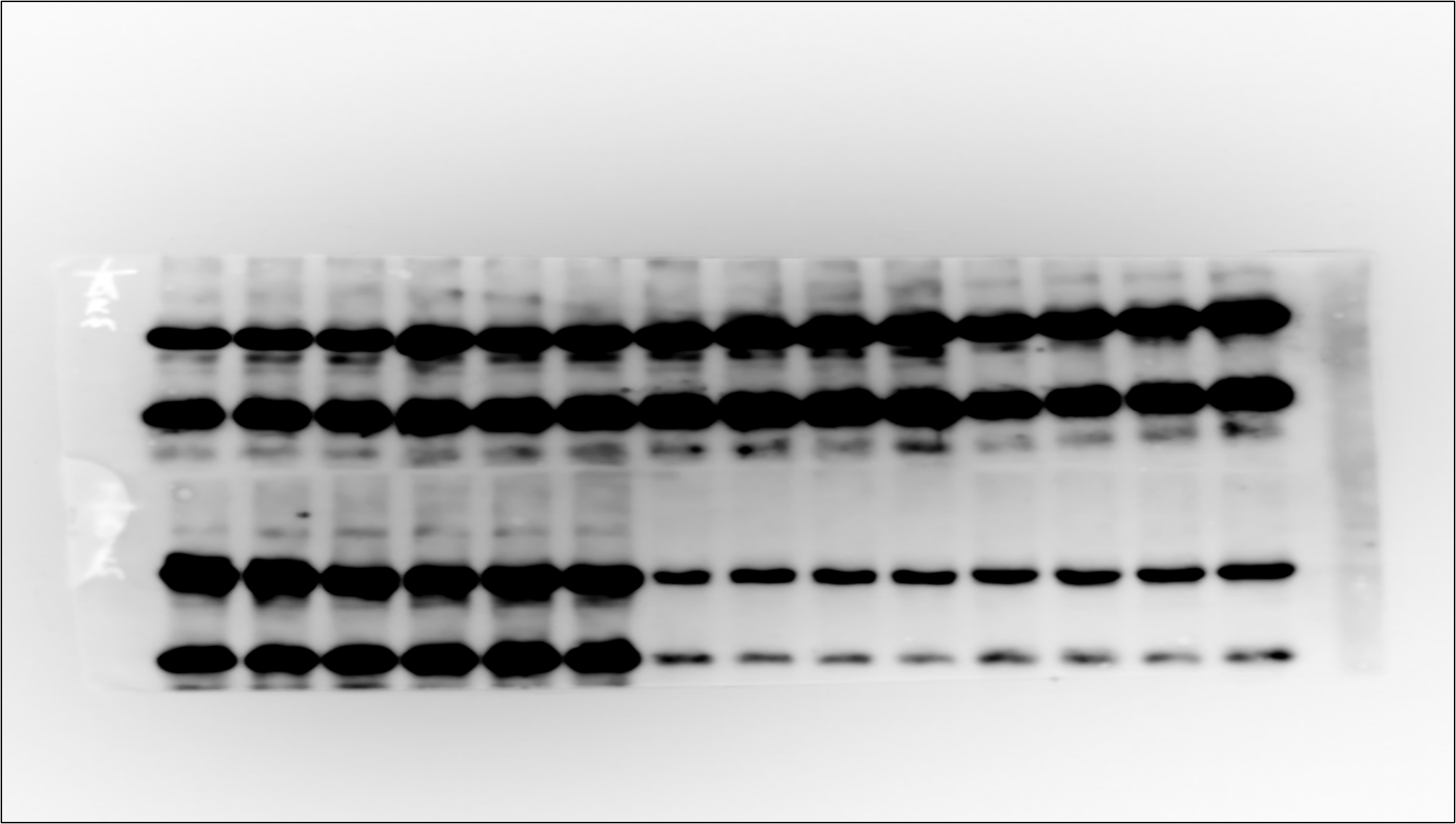

Supplement: Figure 8—source data 2. [file elife-108048-fig8-data2.zip › Figure 8/Figure 8 K-IP-Myc.tif]

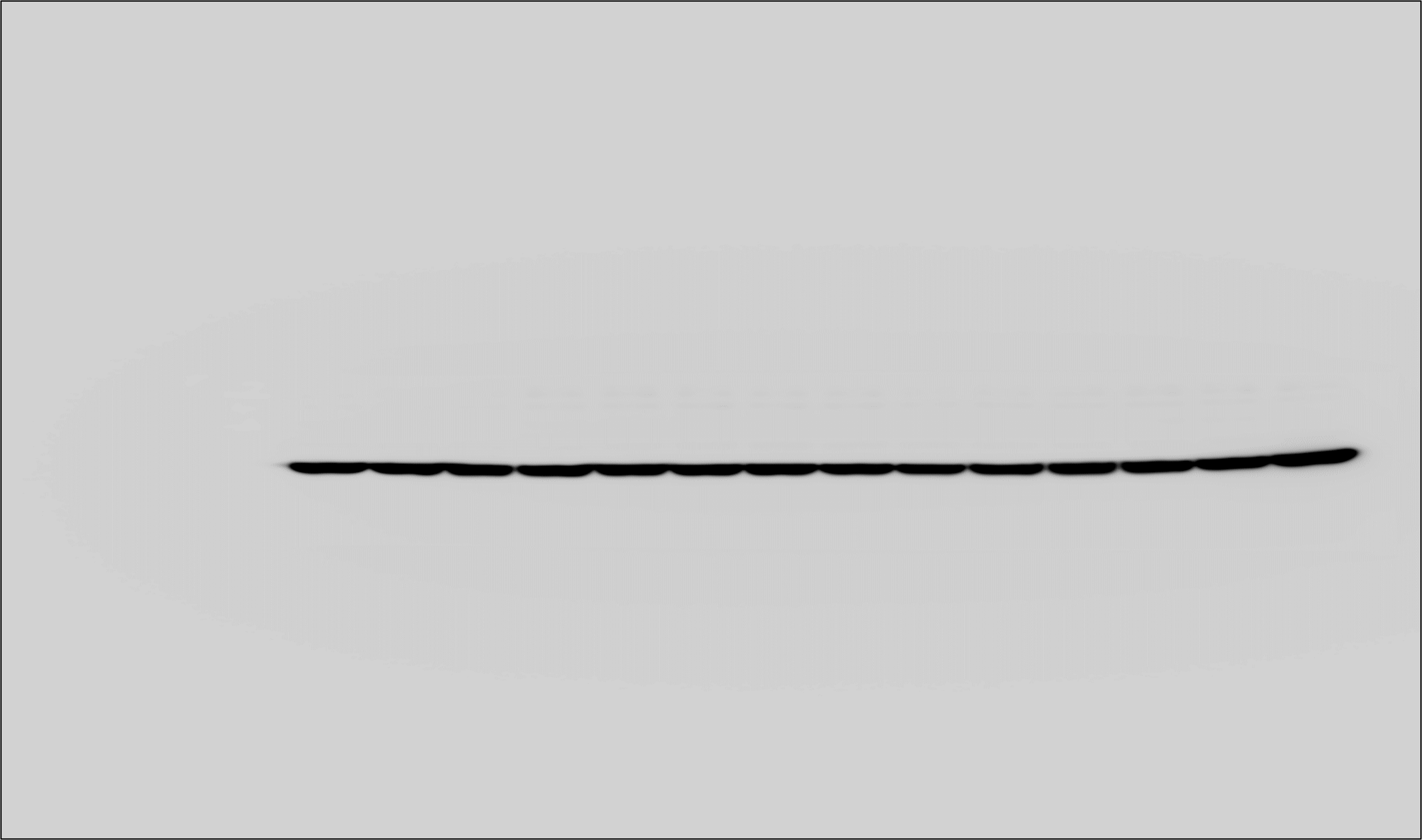

Supplement: Figure 8—source data 2. [file elife-108048-fig8-data2.zip › Figure 8/Figure 8 K-WCL-Actin.tif]

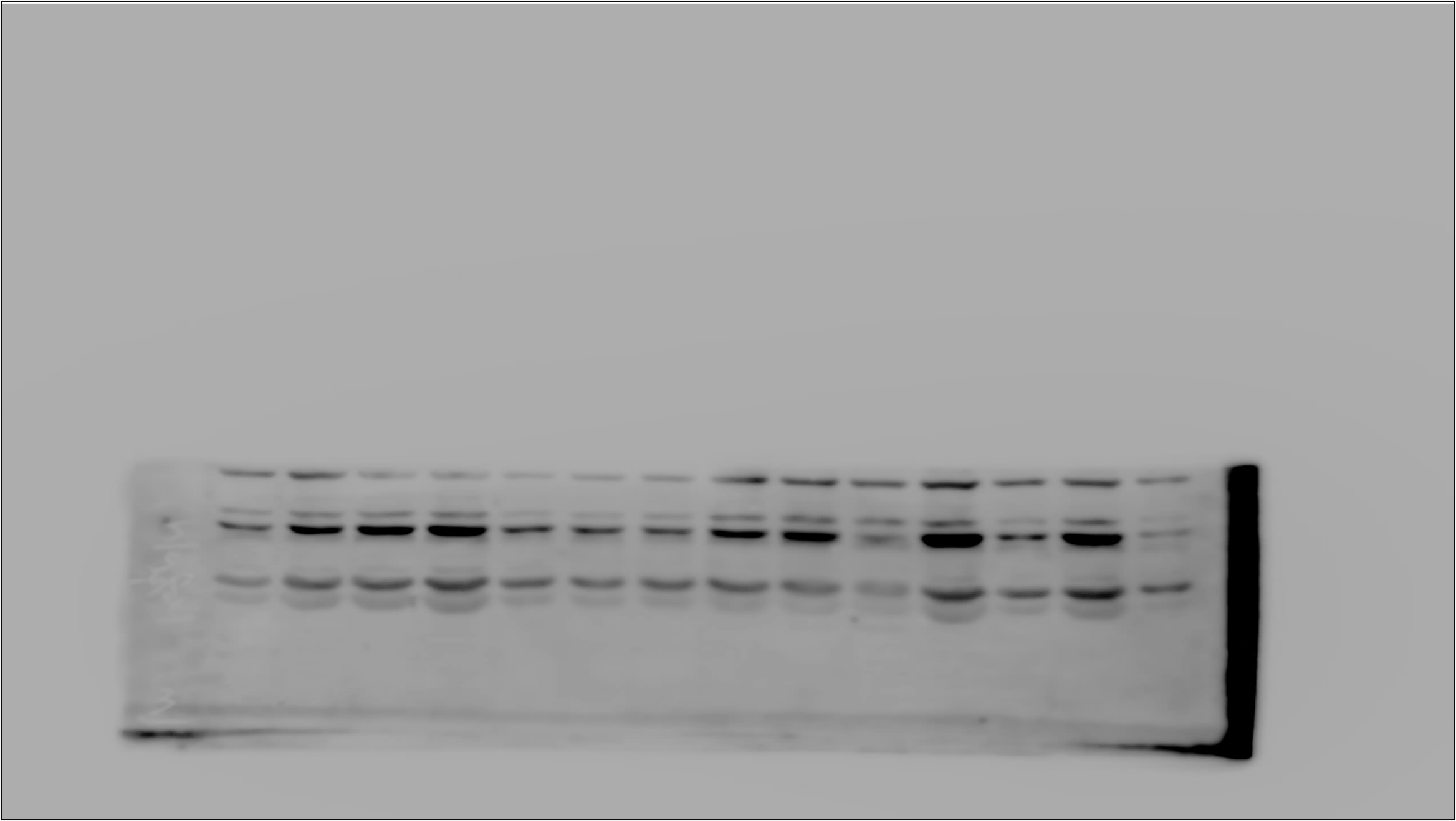

Supplement: Figure 8—source data 2. [file elife-108048-fig8-data2.zip › Figure 8/Figure 8 K-WCL-cyp17a2.tif]

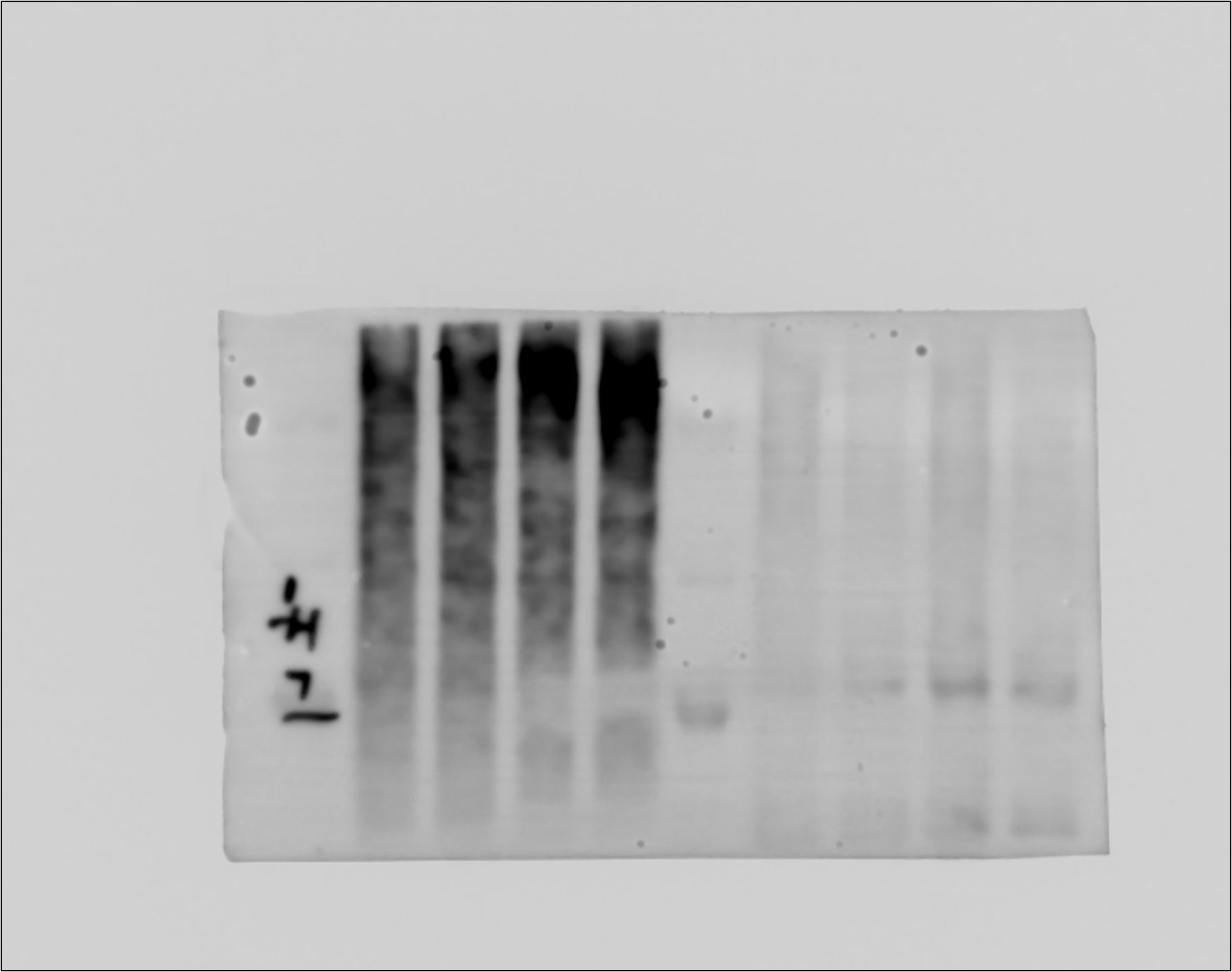

Supplement: Figure 8—source data 2. [file elife-108048-fig8-data2.zip › Figure 8/Figure 8 K-WCL-HA.tif]

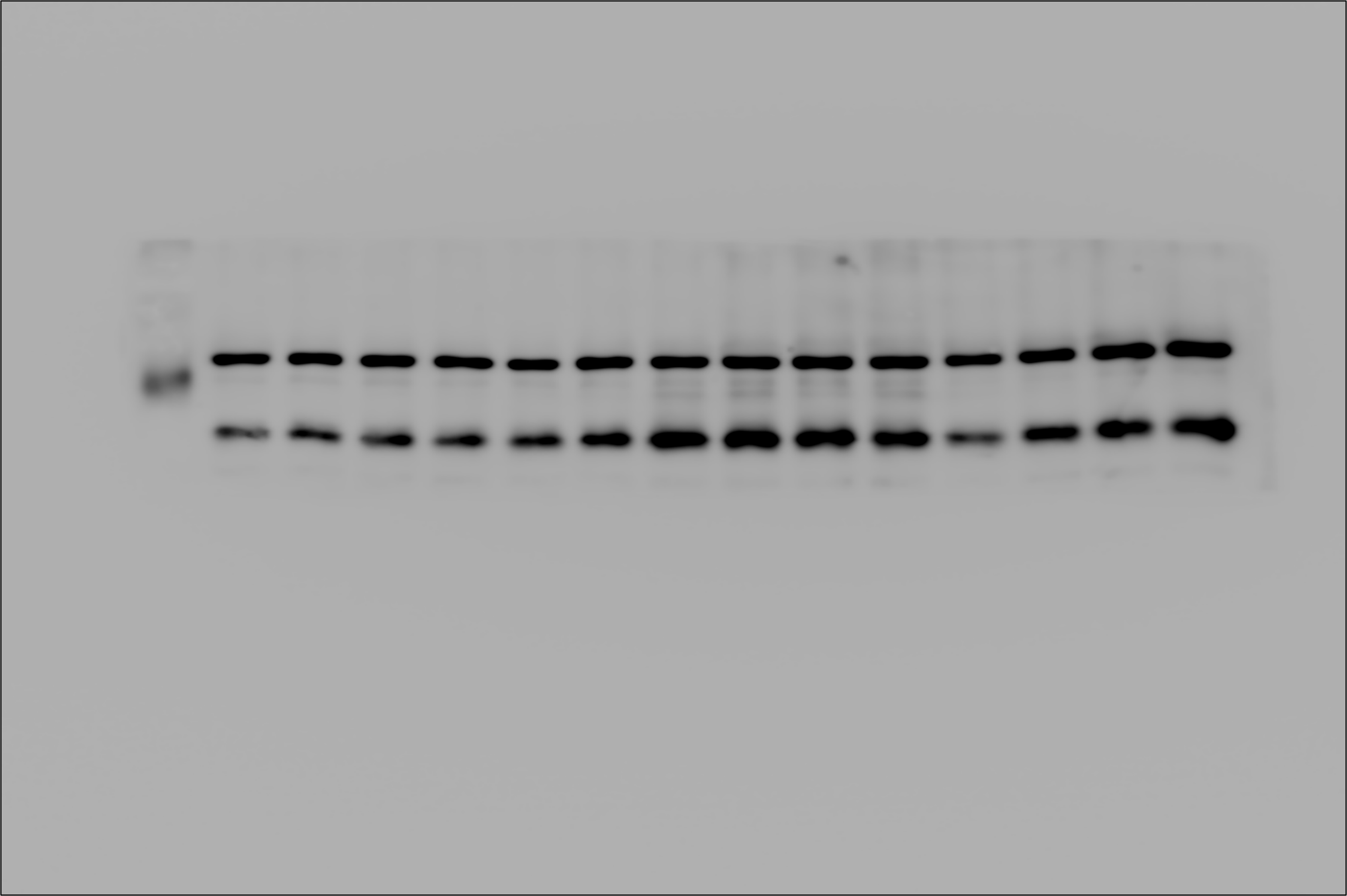

Supplement: Figure 8—source data 2. [file elife-108048-fig8-data2.zip › Figure 8/Figure 8 K-WCL-Myc.tif]

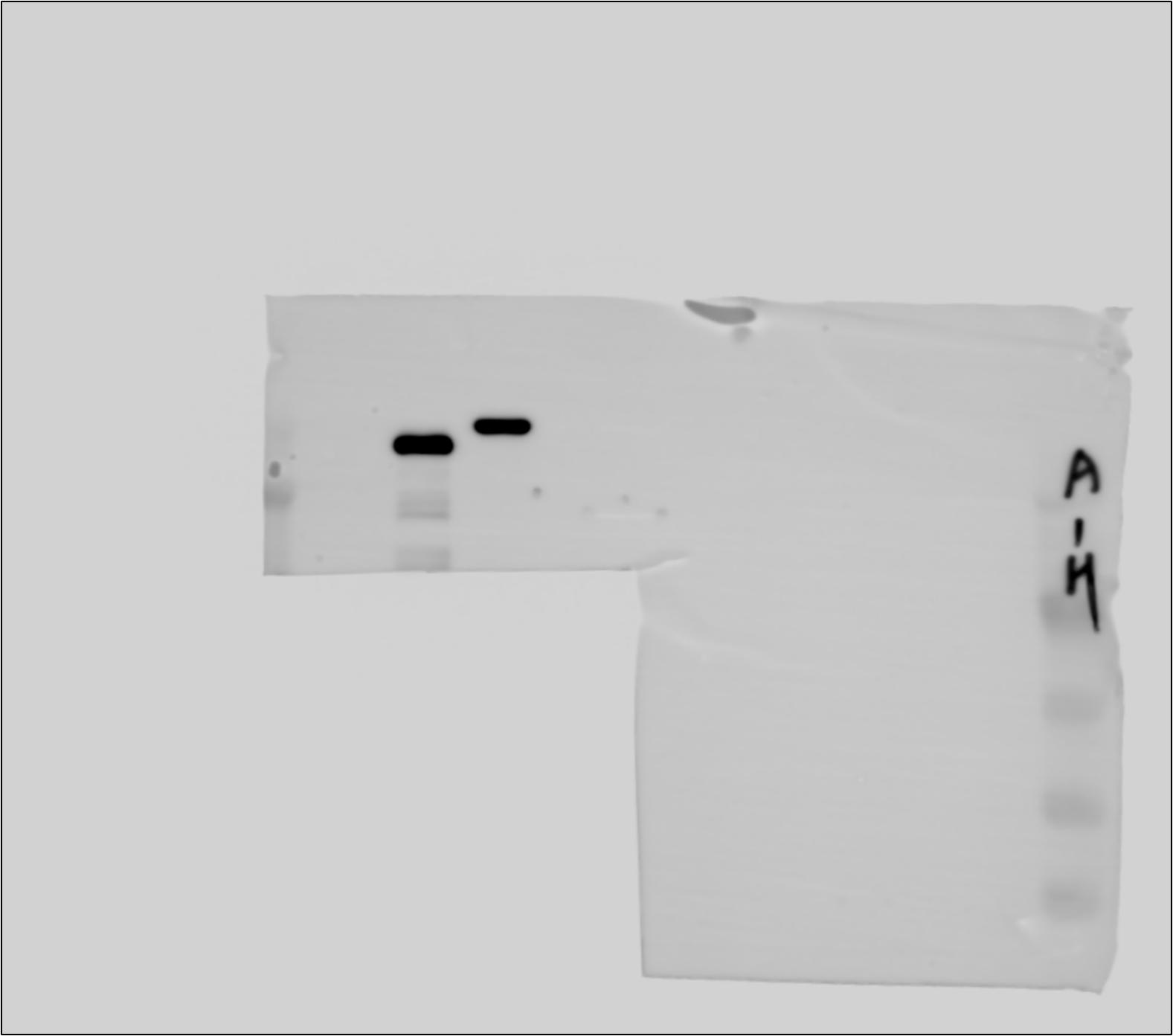

Supplement: Figure 9—source data 2. [file elife-108048-fig9-data2.zip › Figure 9/Figure 9 B-IP-HA.tif]

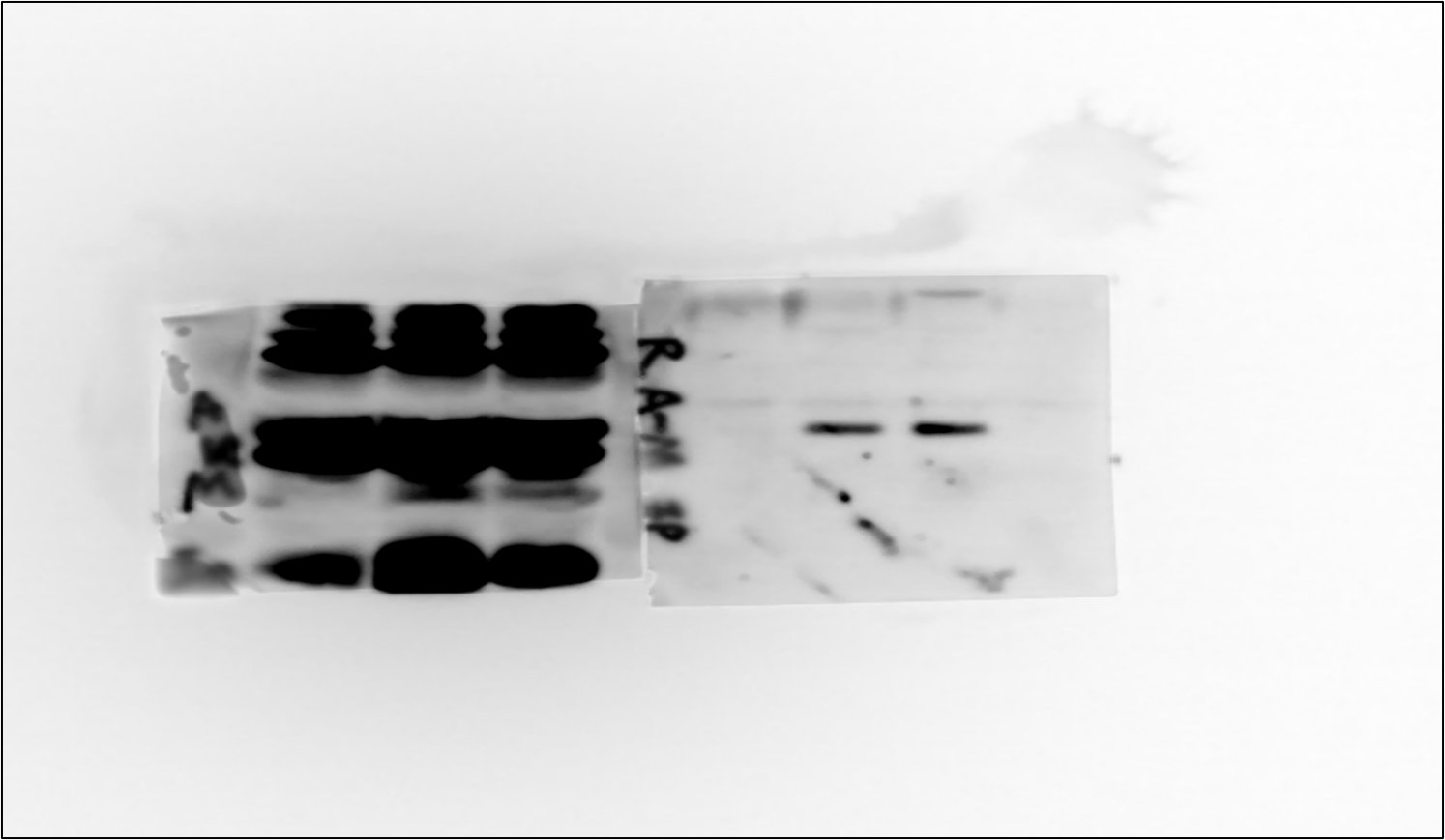

Supplement: Figure 9—source data 2. [file elife-108048-fig9-data2.zip › Figure 9/Figure 9 B-IP-Myc.tif]

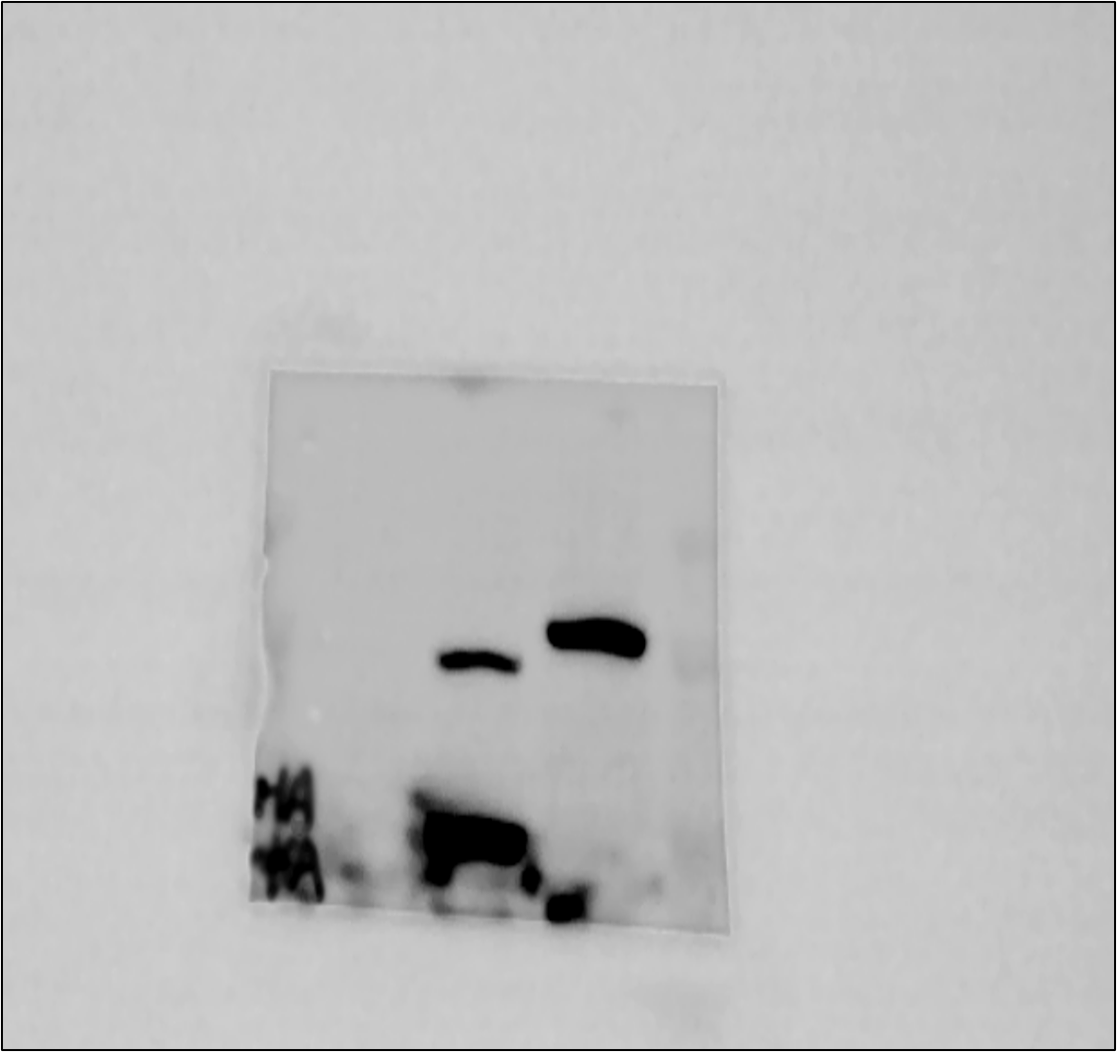

Supplement: Figure 9—source data 2. [file elife-108048-fig9-data2.zip › Figure 9/Figure 9 B-WCL-HA.tif]

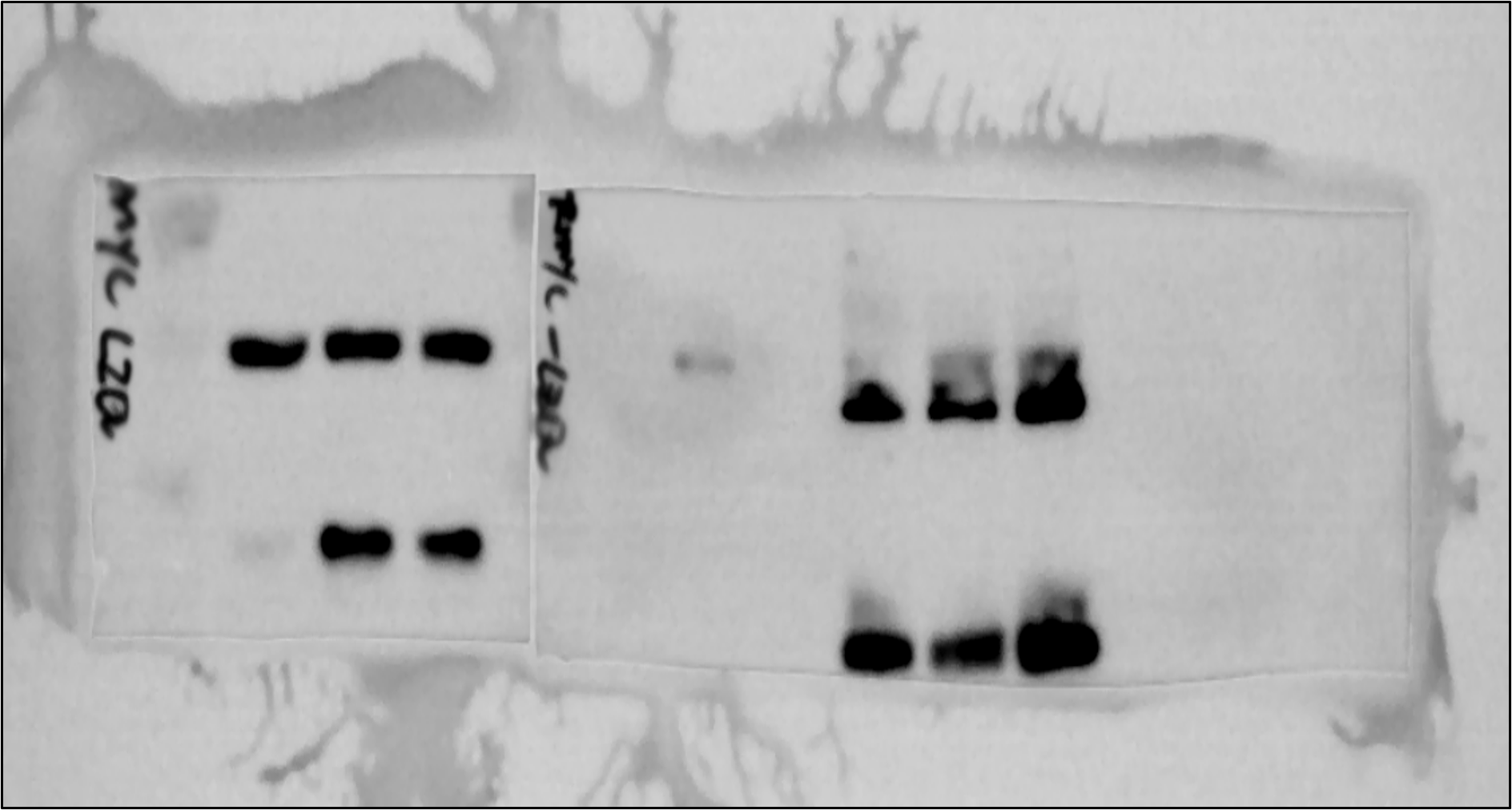

Supplement: Figure 9—source data 2. [file elife-108048-fig9-data2.zip › Figure 9/Figure 9 B-WCL-Myc.tif]

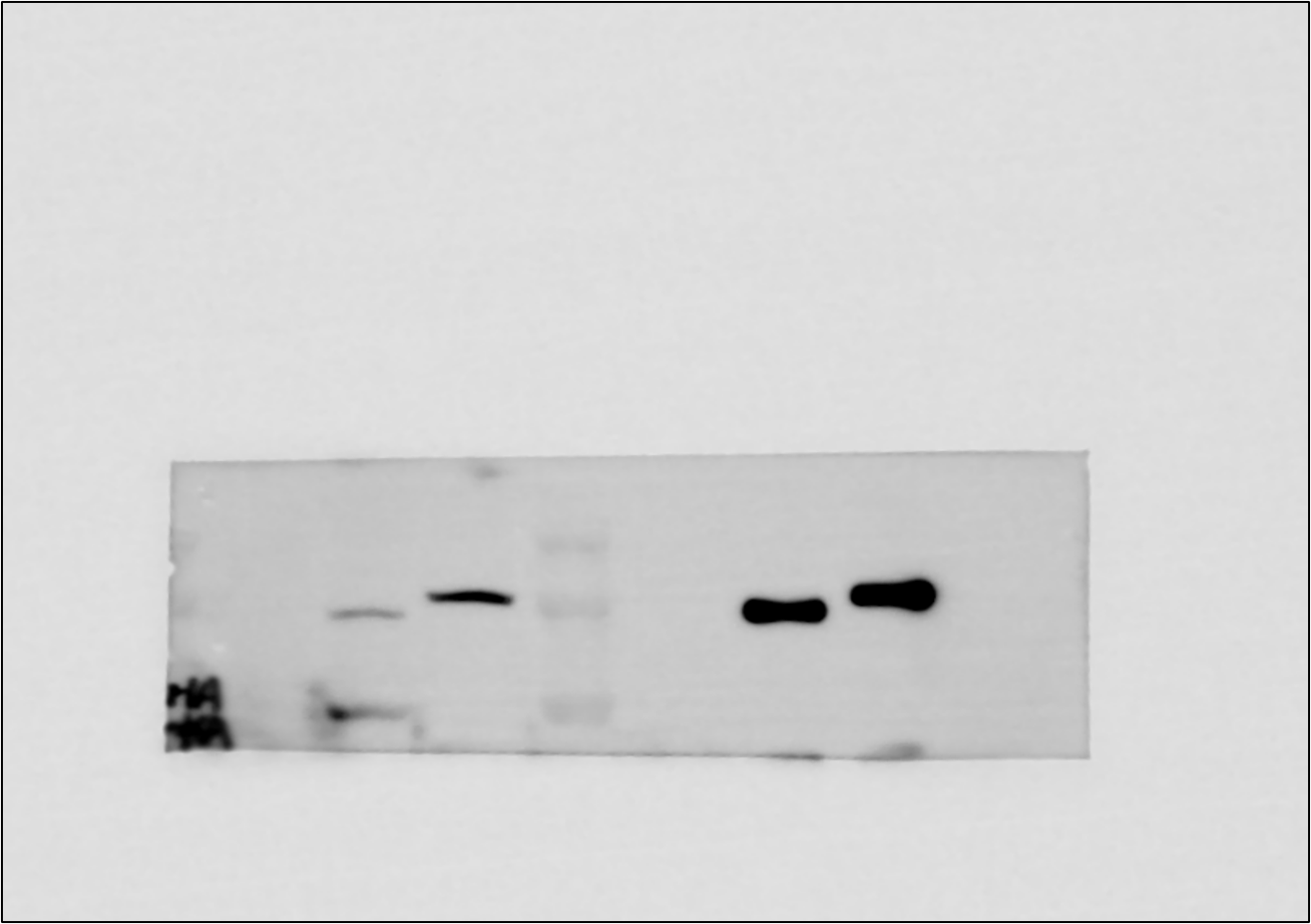

Supplement: Figure 9—source data 2. [file elife-108048-fig9-data2.zip › Figure 9/Figure 9 C-IP-HA.tif]

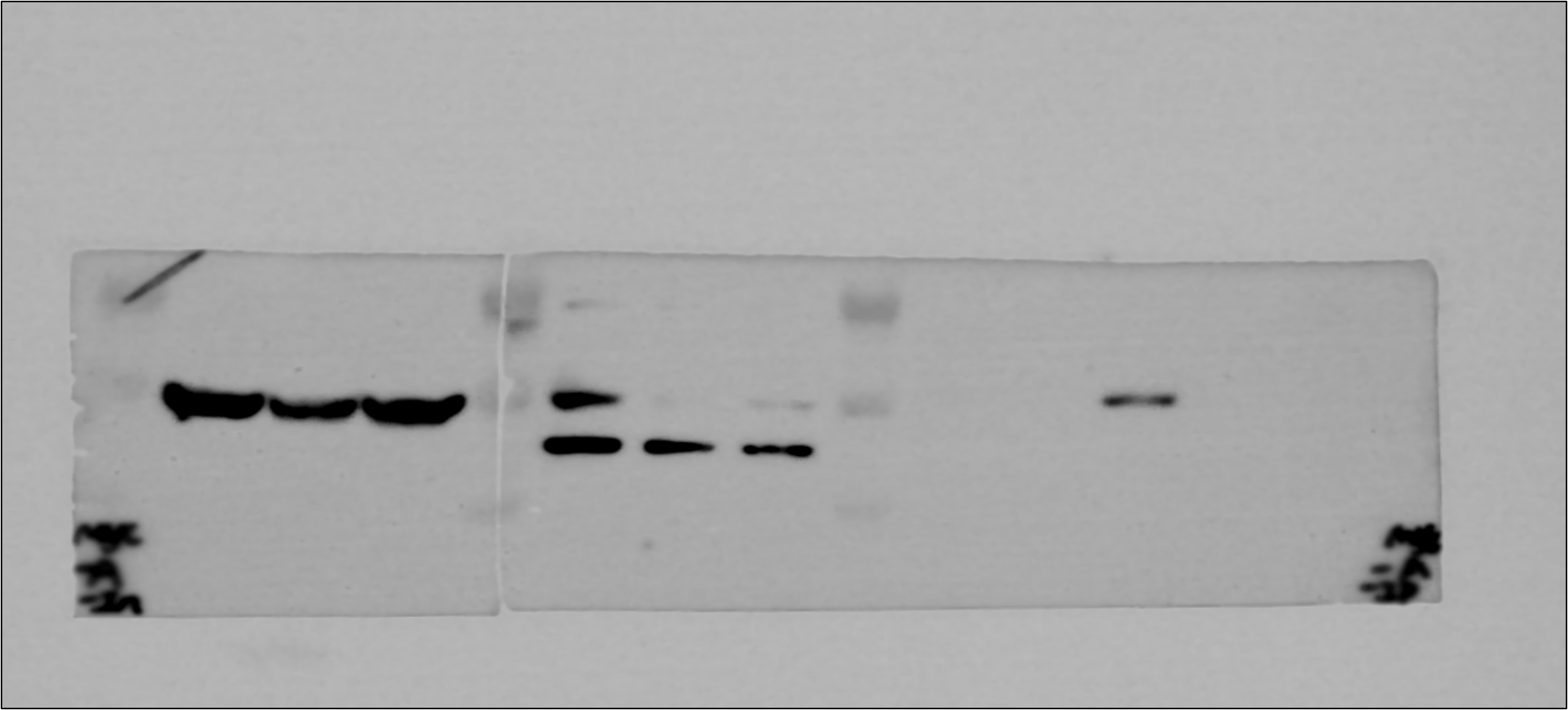

Supplement: Figure 9—source data 2. [file elife-108048-fig9-data2.zip › Figure 9/Figure 9 C-IP-Myc.tif]

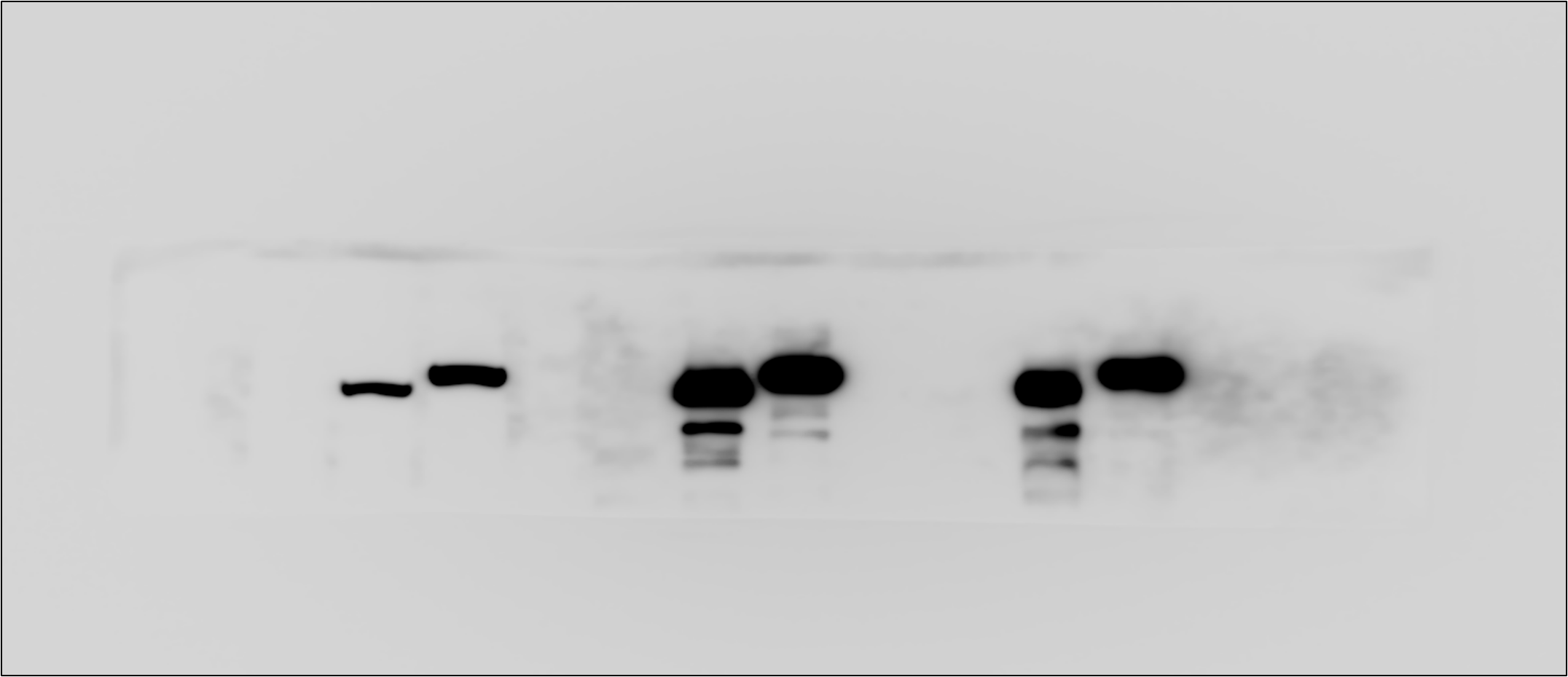

Supplement: Figure 9—source data 2. [file elife-108048-fig9-data2.zip › Figure 9/Figure 9 C-WCL-HA.tif]

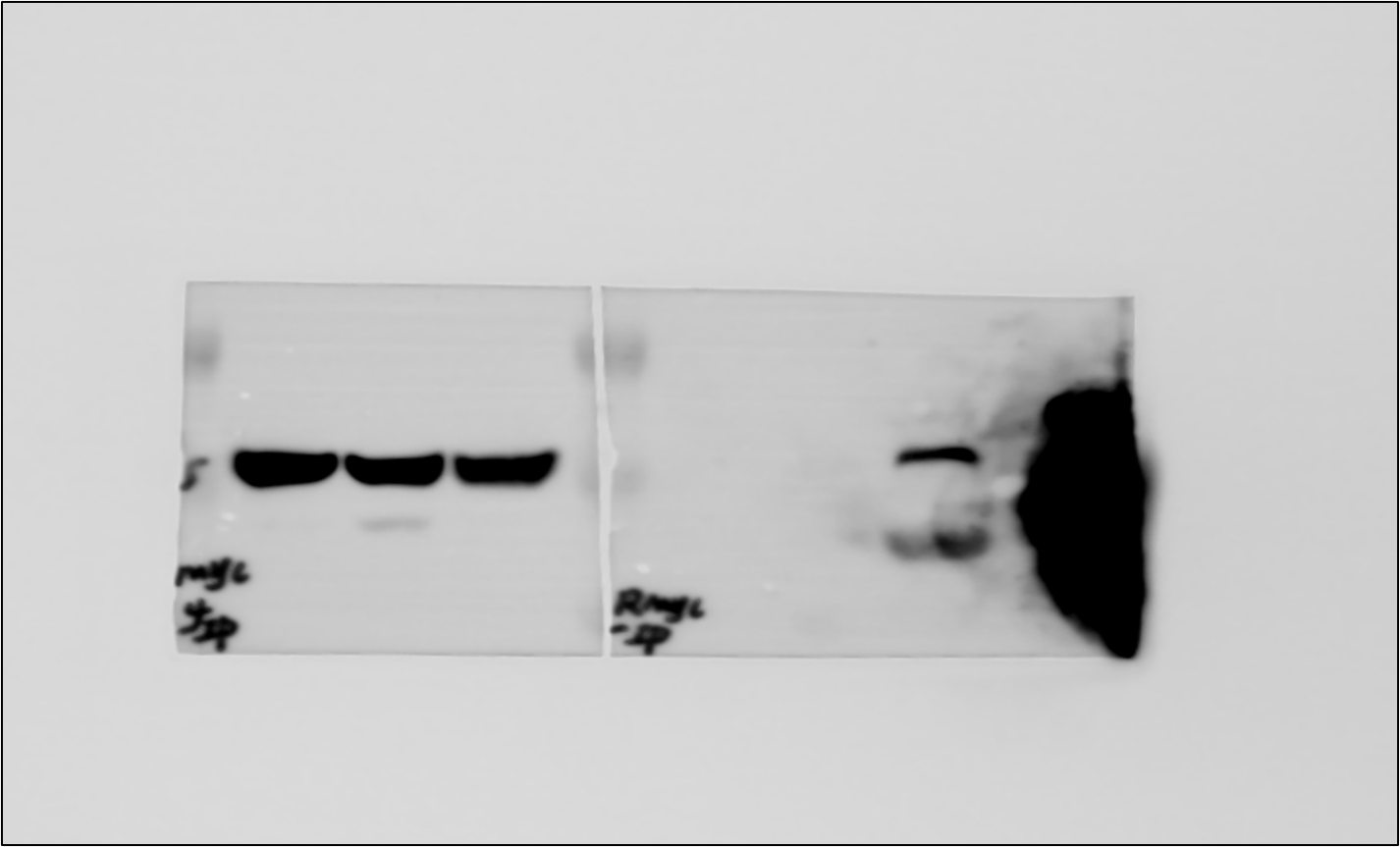

Supplement: Figure 9—source data 2. [file elife-108048-fig9-data2.zip › Figure 9/Figure 9 C-WCL-Myc.tif]
