# Supplementary figures and images for: Male-biased Cyp17a2 orchestrates antiviral sexual dimorphism in fish via STING stabilization and viral protein degradation (part 4 of 5)
Source: eLife. 2026 Feb 18;14:RP108048. doi: 10.7554/eLife.108048 (PMC12916102; doi:10.7554/eLife.108048)

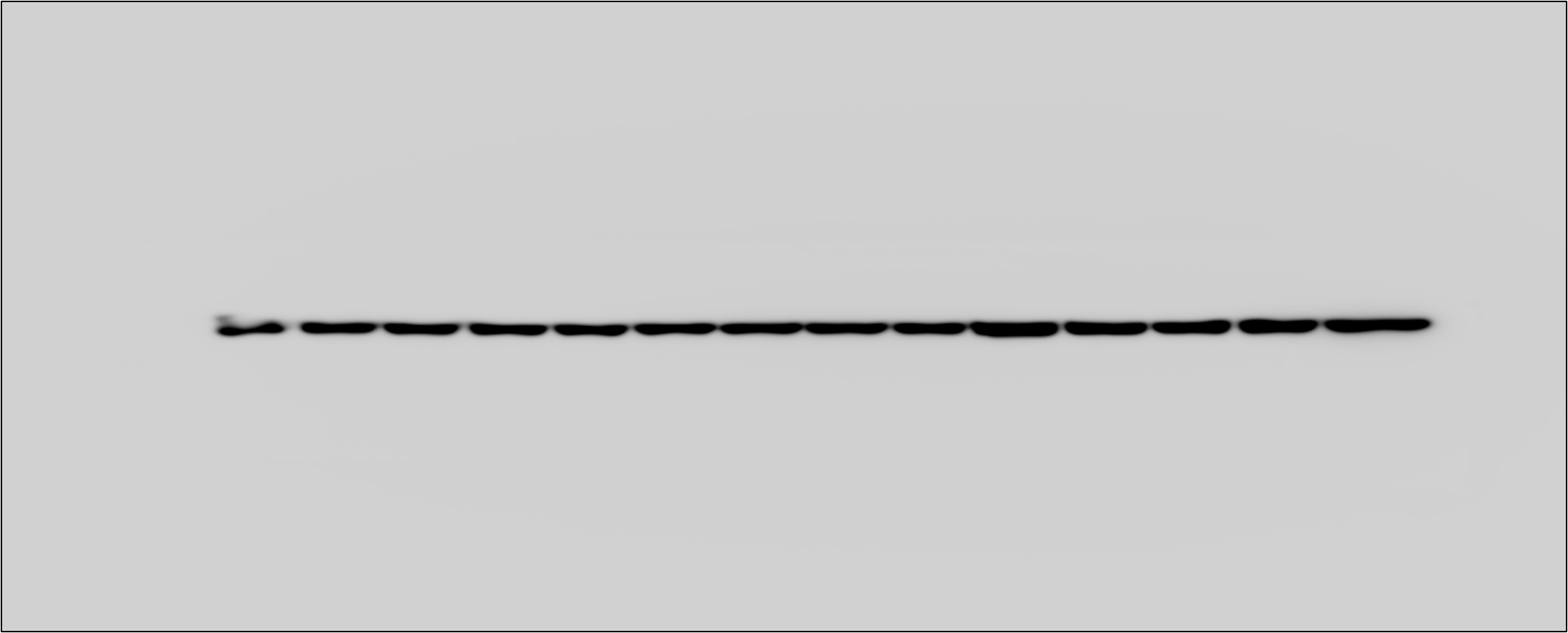

Supplement: Figure 9—source data 2. [file elife-108048-fig9-data2.zip › Figure 9/Figure 9 E-Actin.tif]

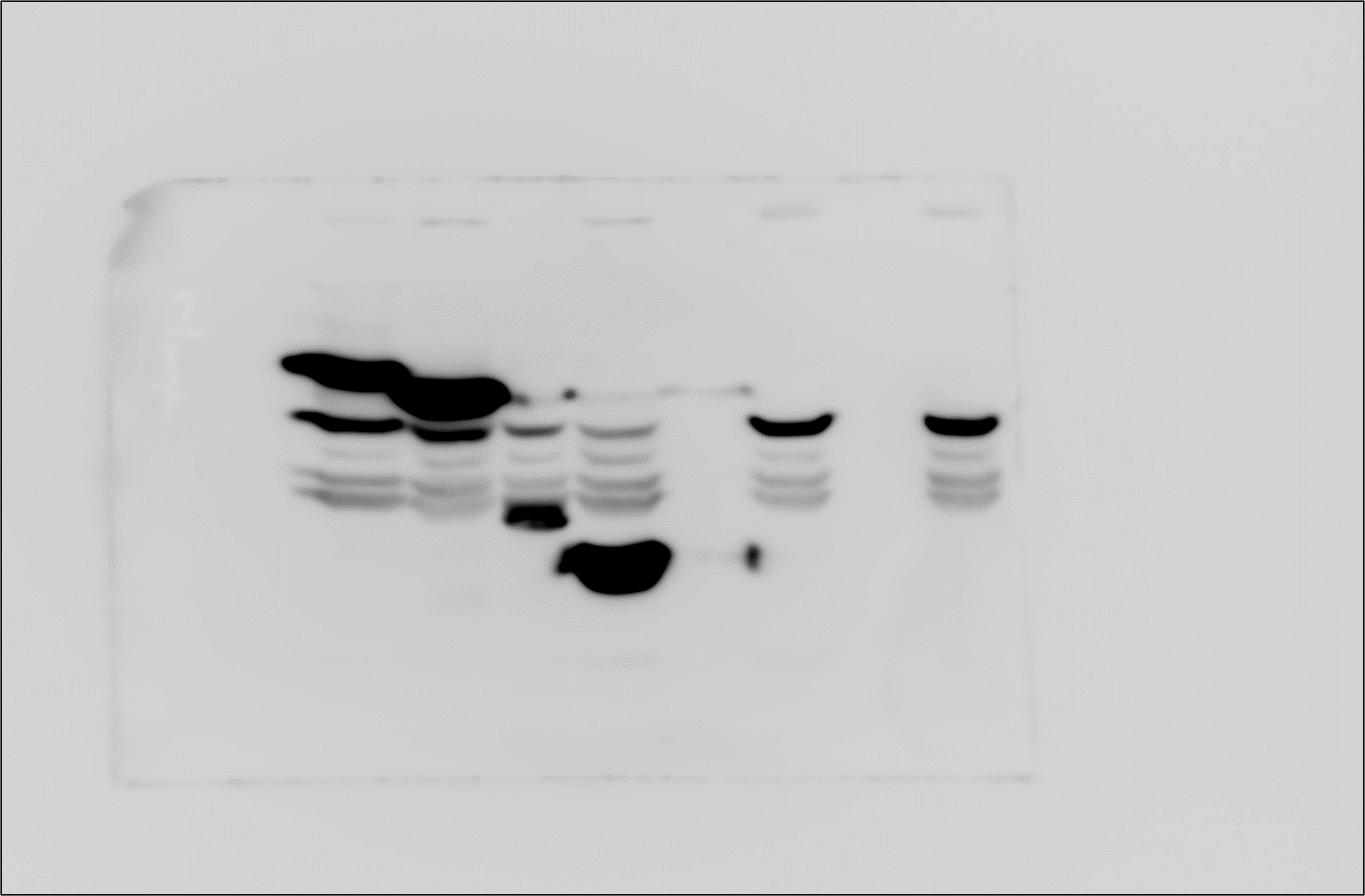

Supplement: Figure 9—source data 2. [file elife-108048-fig9-data2.zip › Figure 9/Figure 9 E-Flag.tif]

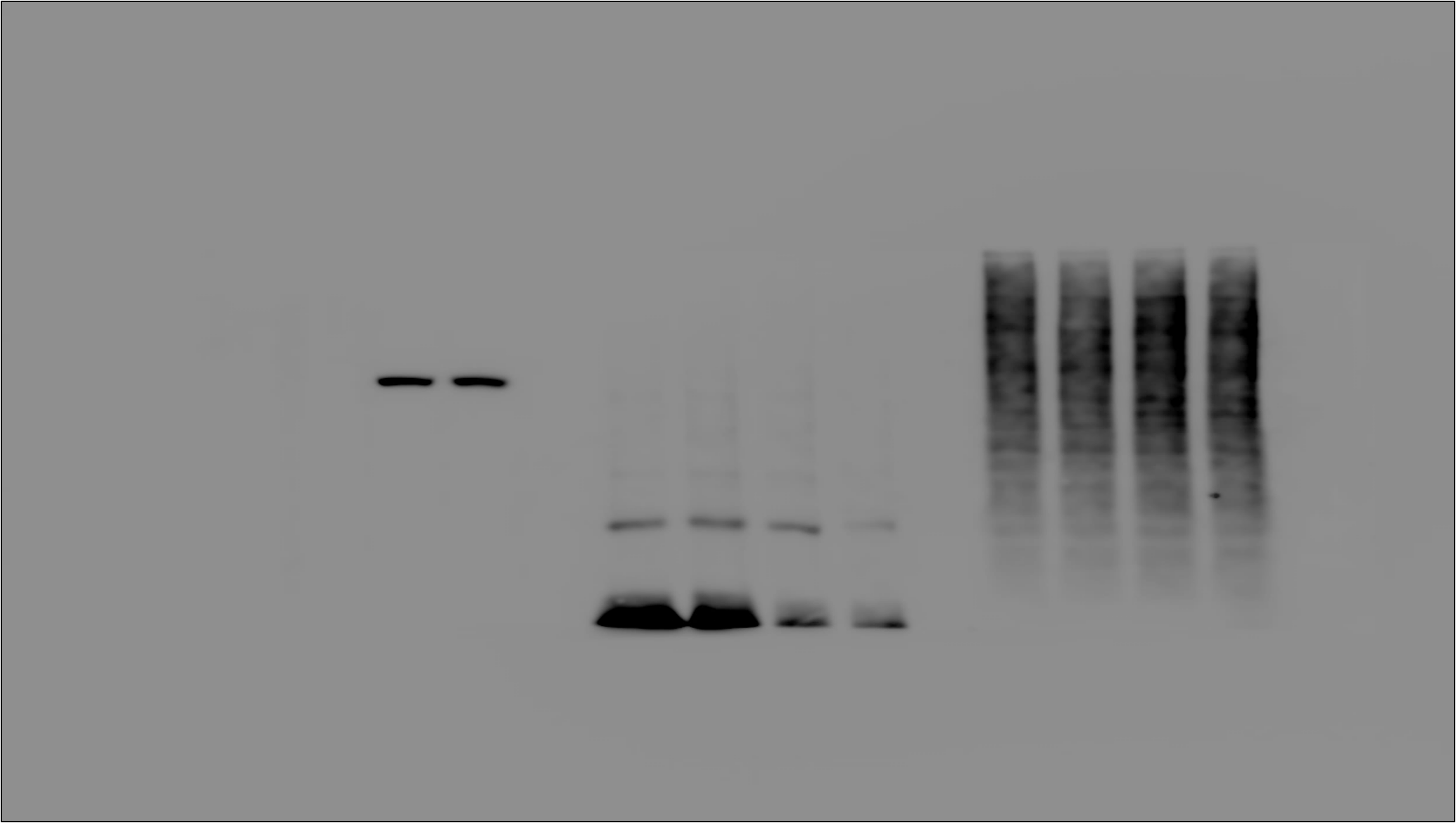

Supplement: Figure 9—source data 2. [file elife-108048-fig9-data2.zip › Figure 9/Figure 9 E-HA.tif]

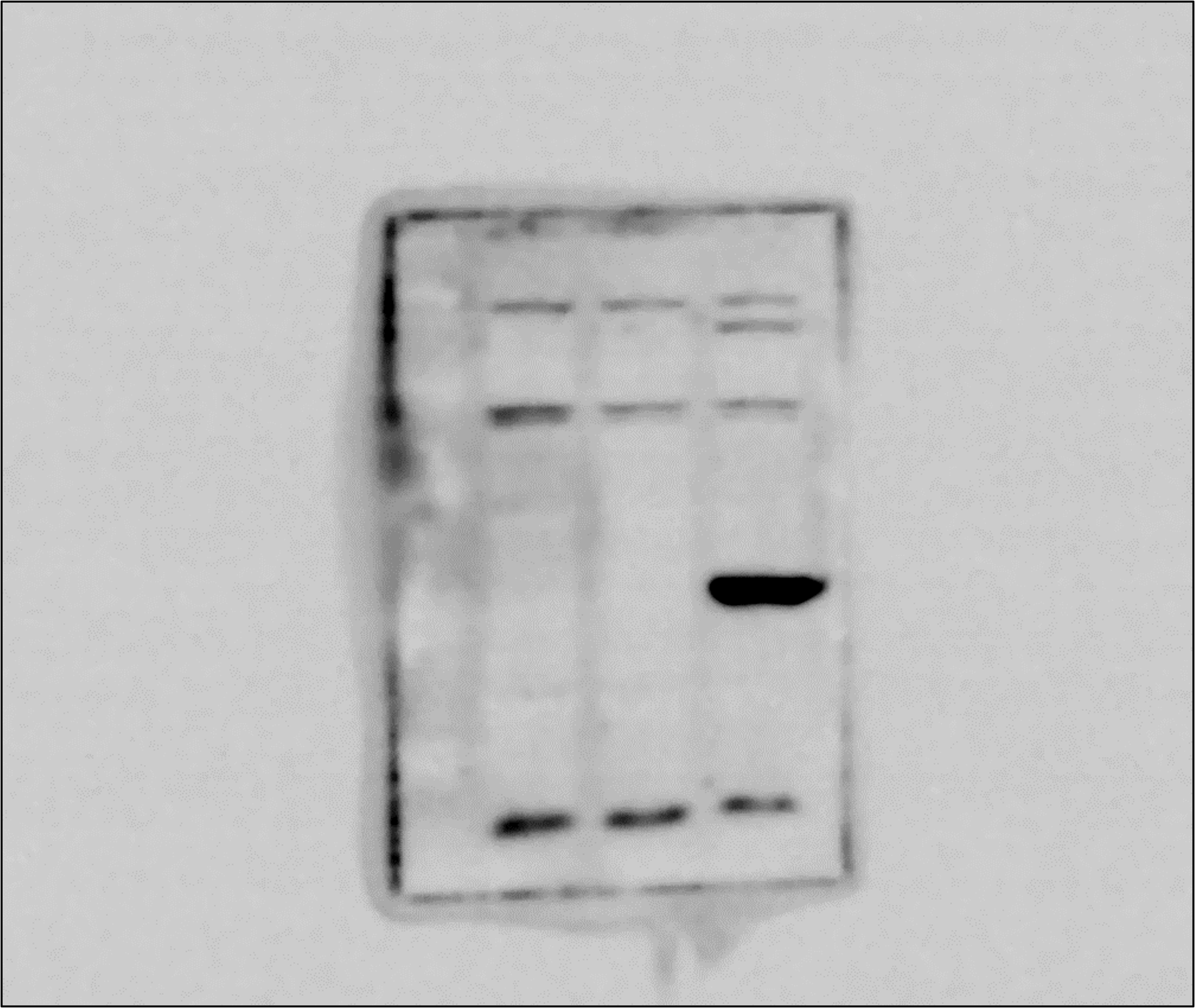

Supplement: Figure 9—source data 2. [file elife-108048-fig9-data2.zip › Figure 9/Figure 9 E-IP-Flag.tif]

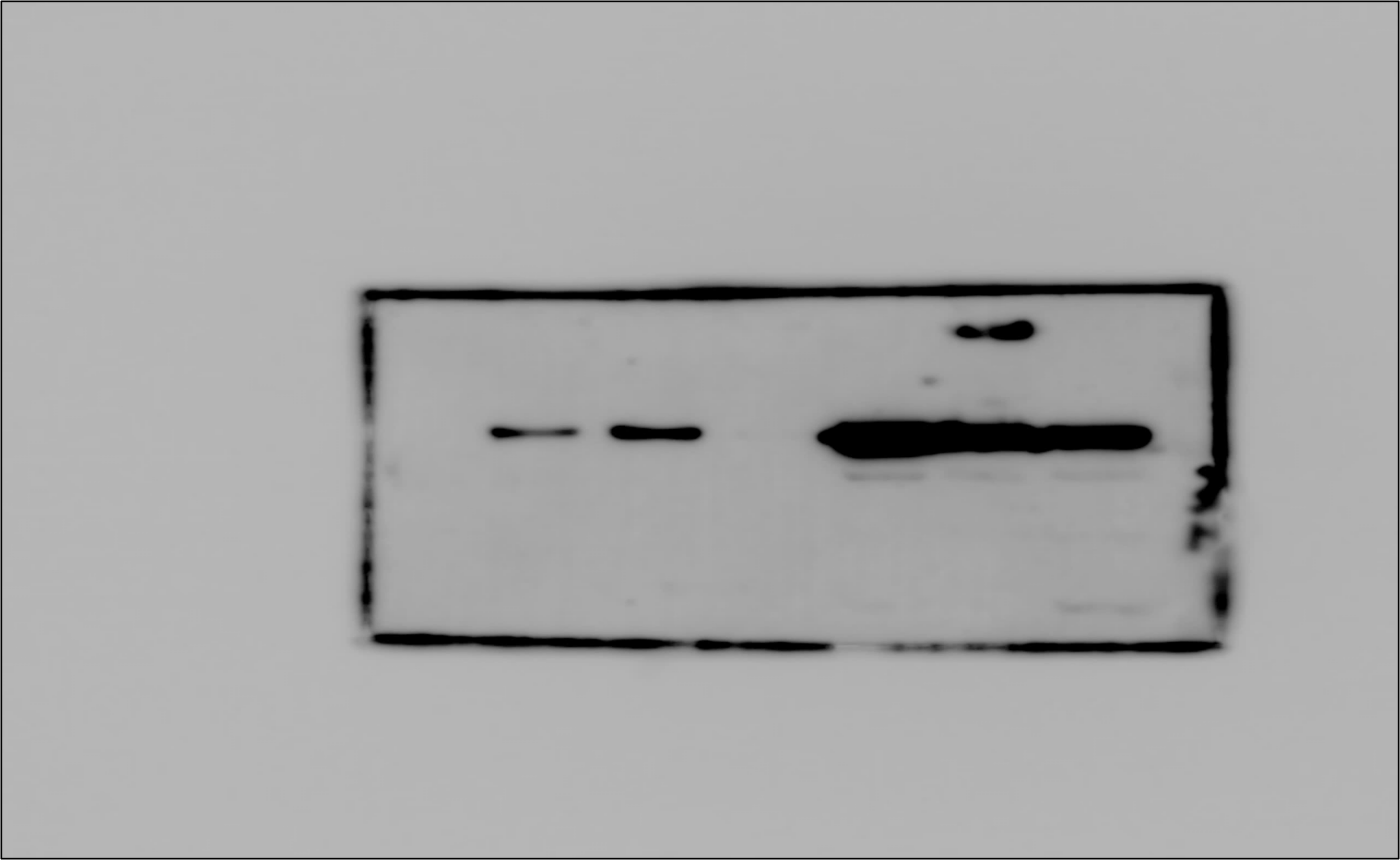

Supplement: Figure 9—source data 2. [file elife-108048-fig9-data2.zip › Figure 9/Figure 9 E-IP-HA.tif]

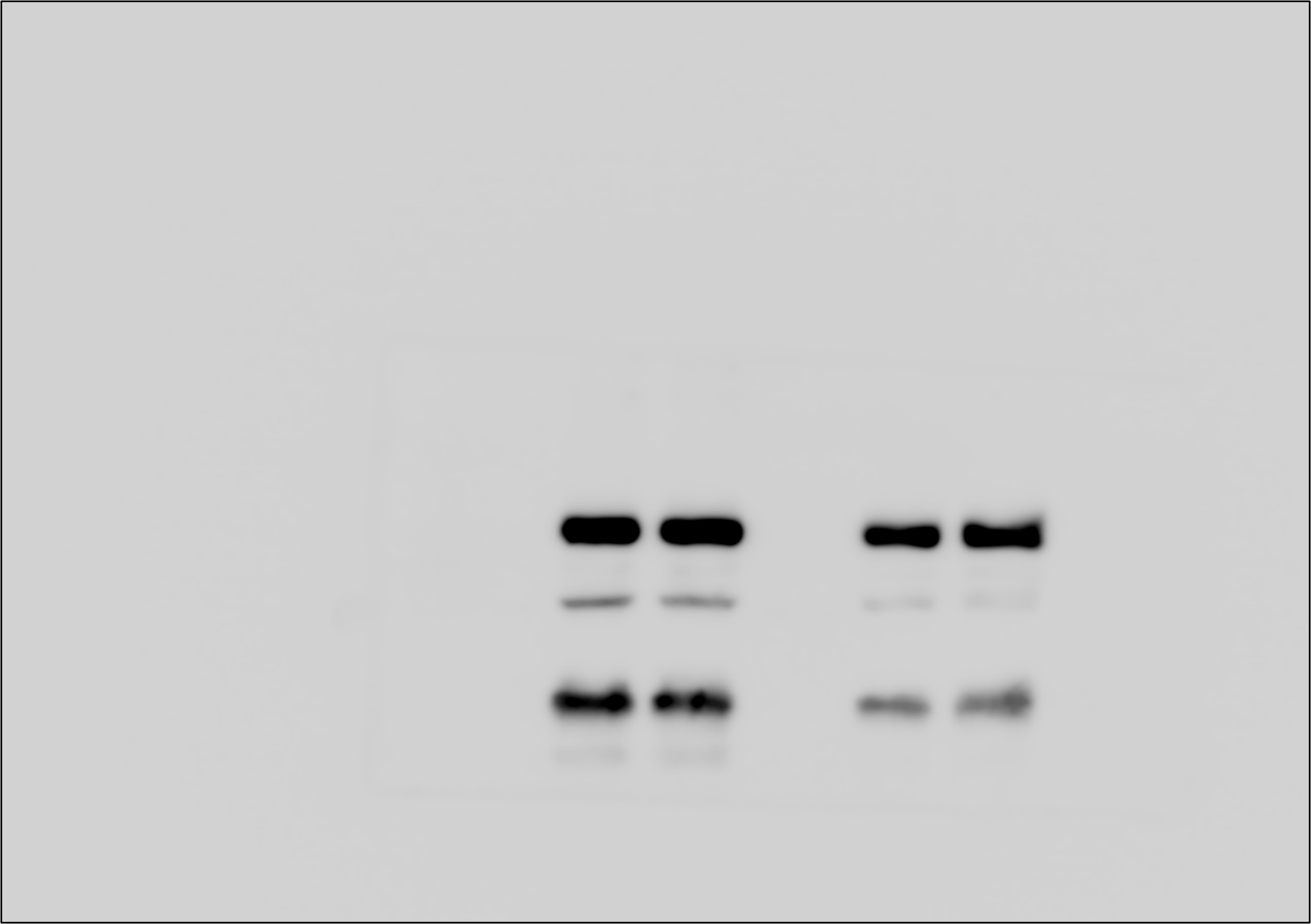

Supplement: Figure 9—source data 2. [file elife-108048-fig9-data2.zip › Figure 9/Figure 9 E-IP-Myc.tif]

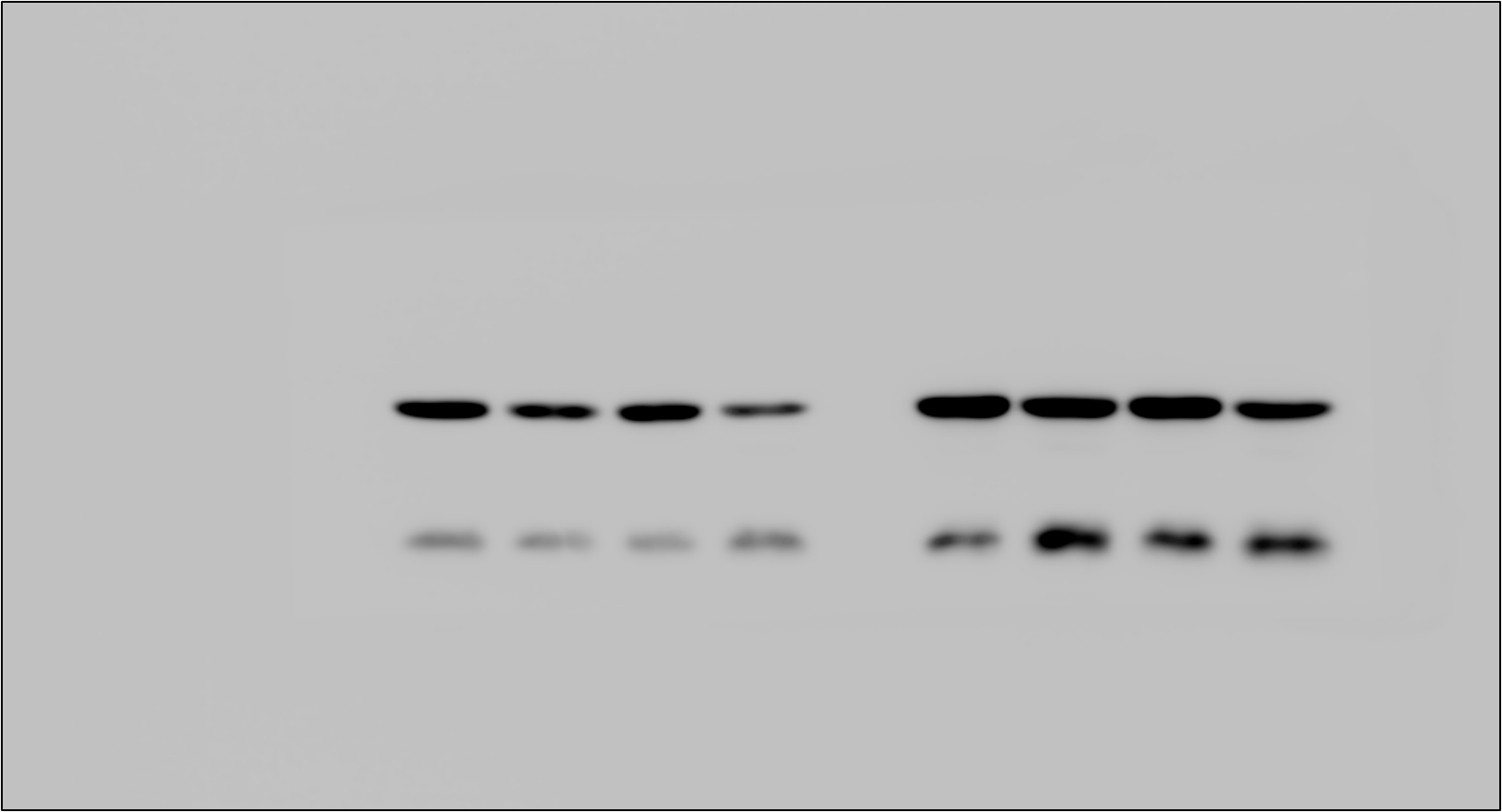

Supplement: Figure 9—source data 2. [file elife-108048-fig9-data2.zip › Figure 9/Figure 9 E-Myc.tif]

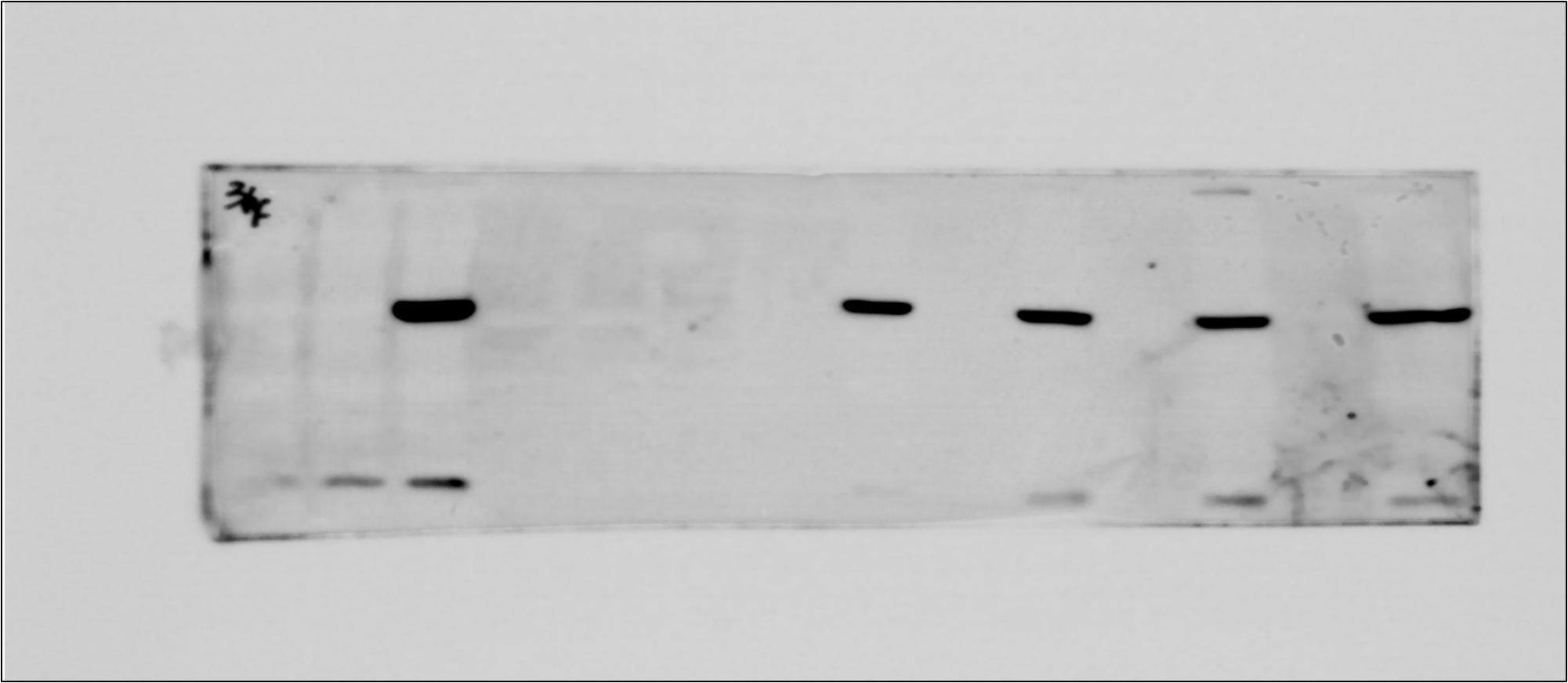

Supplement: Figure 9—source data 2. [file elife-108048-fig9-data2.zip › Figure 9/Figure 9 E-WCL-Flag.tif]

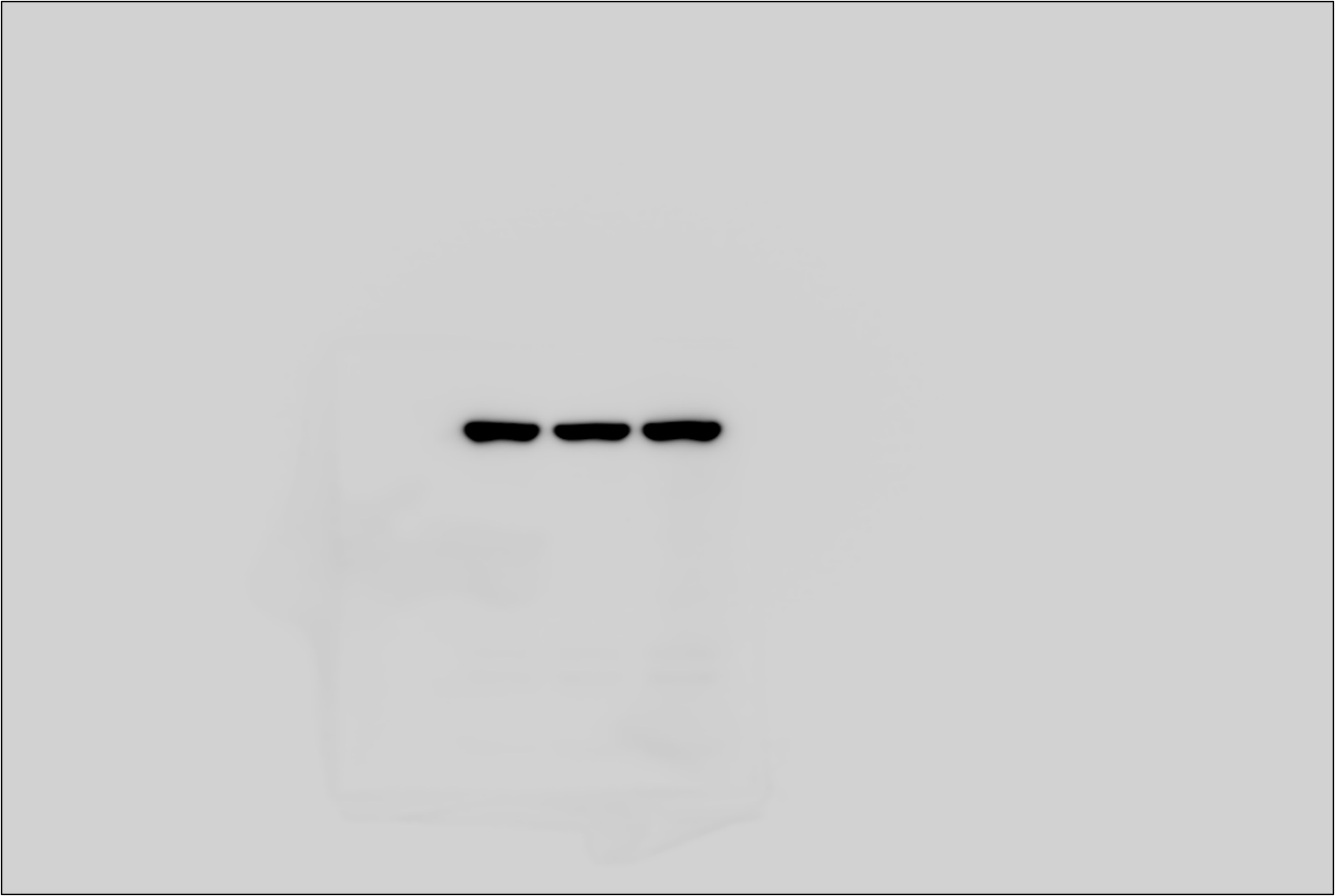

Supplement: Figure 9—source data 2. [file elife-108048-fig9-data2.zip › Figure 9/Figure 9 E-WCL-HA.tif]

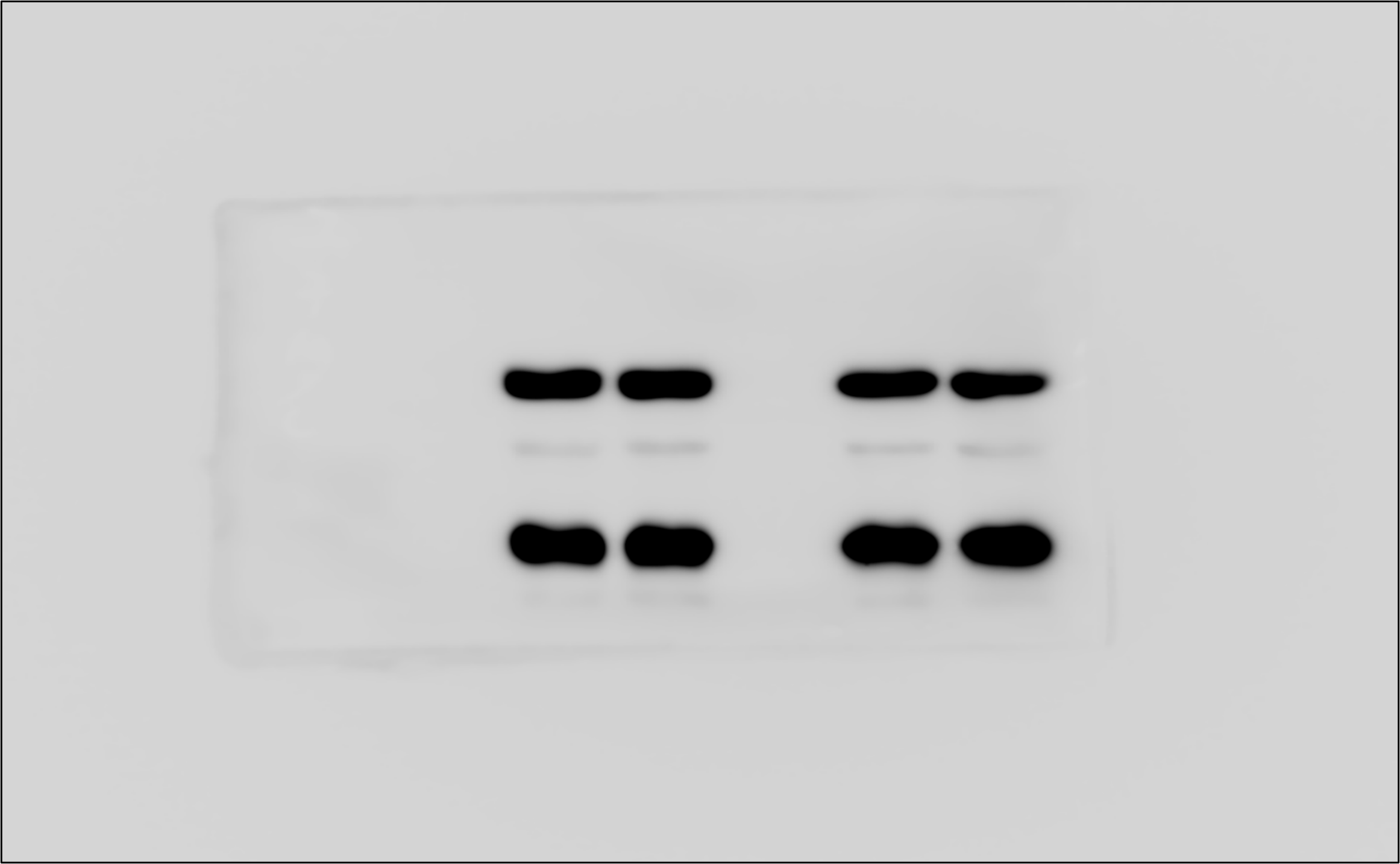

Supplement: Figure 9—source data 2. [file elife-108048-fig9-data2.zip › Figure 9/Figure 9 E-WCL-myc.tif]

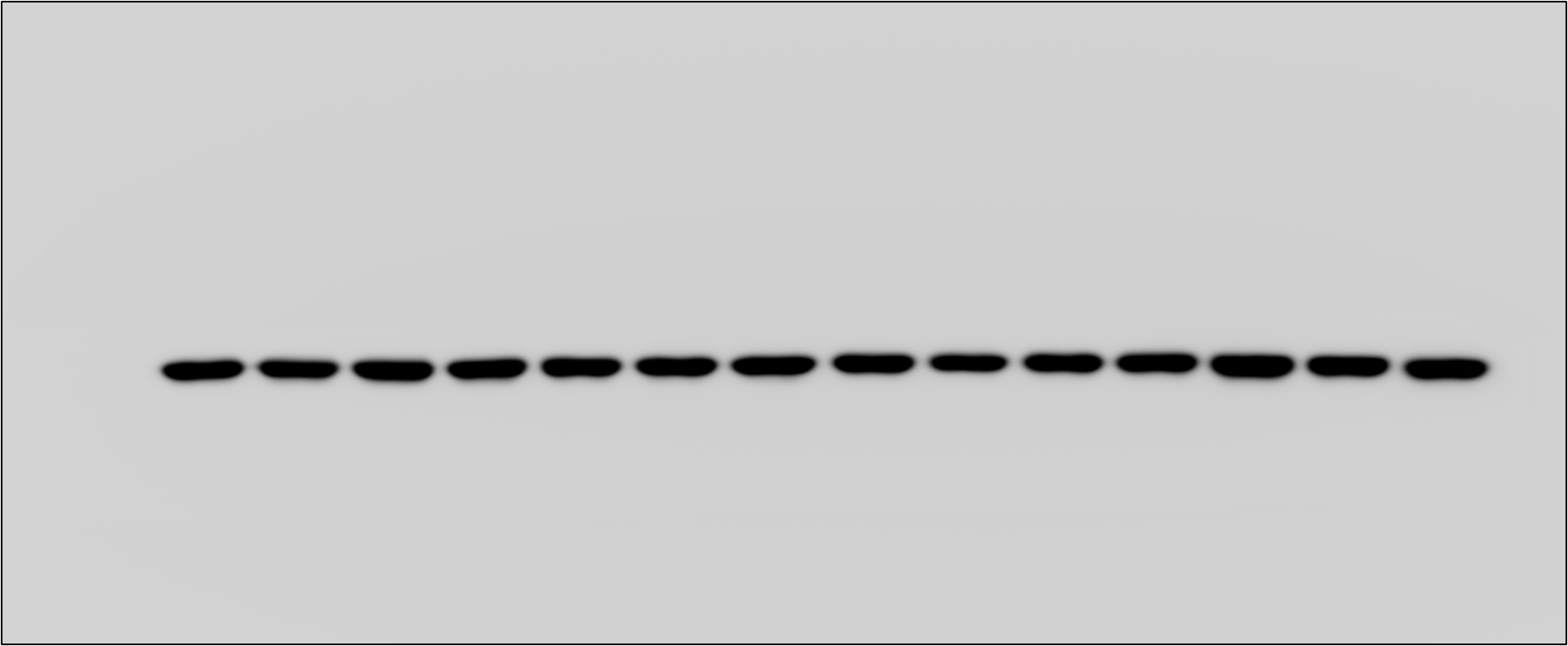

Supplement: Figure 9—source data 2. [file elife-108048-fig9-data2.zip › Figure 9/Figure 9 G-Actin.tif]

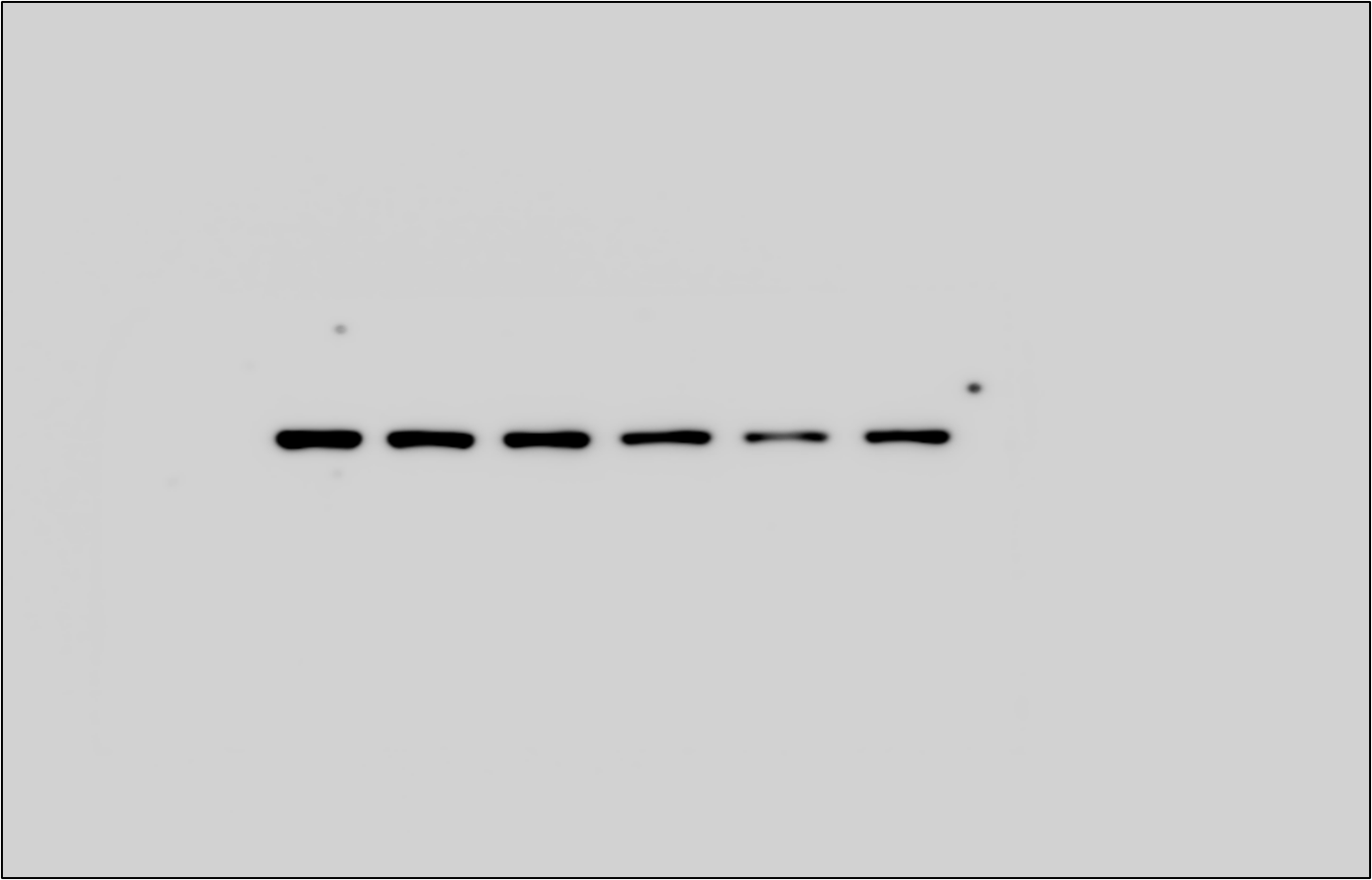

Supplement: Figure 9—source data 2. [file elife-108048-fig9-data2.zip › Figure 9/Figure 9 G-Flag.tif]

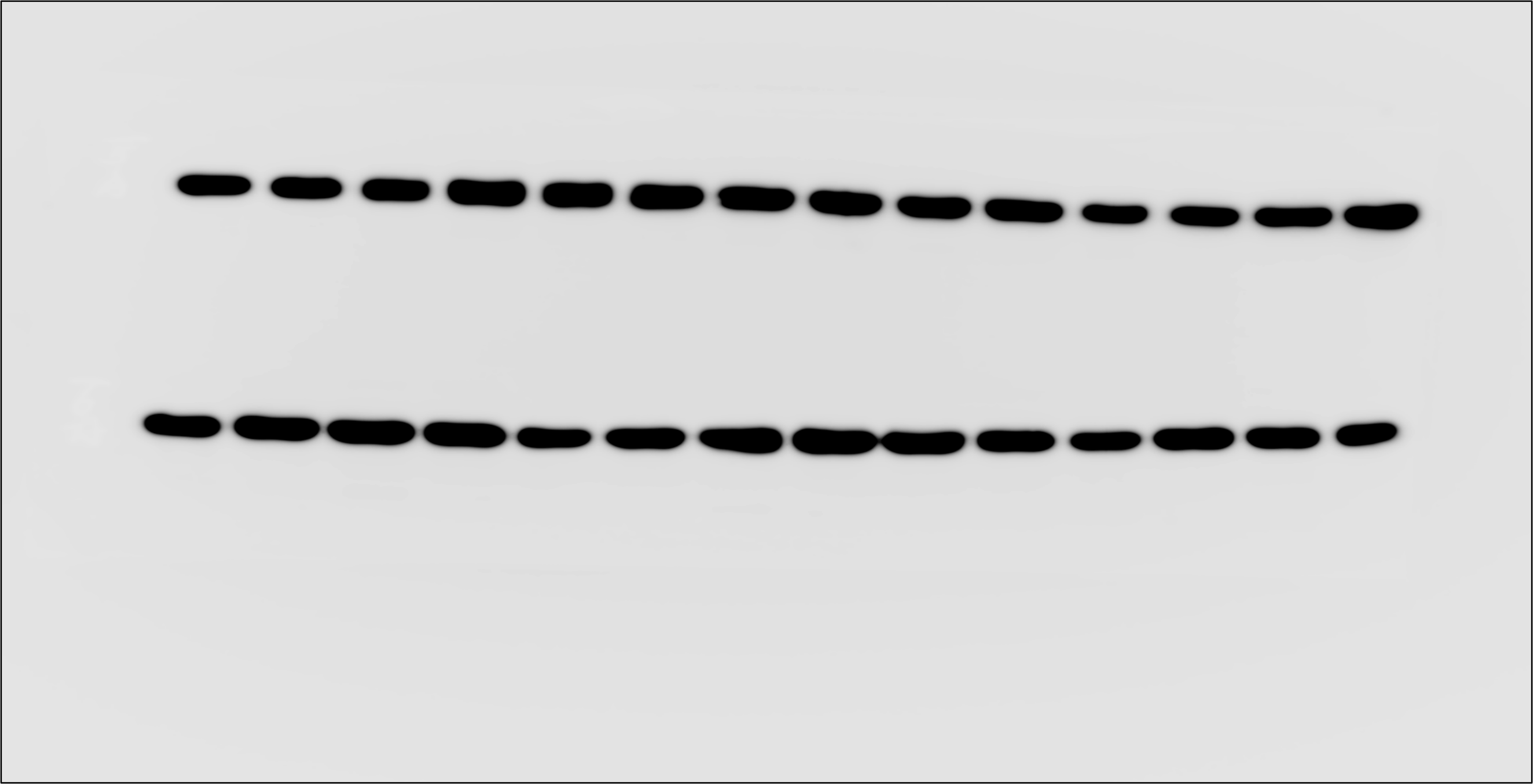

Supplement: Figure 9—source data 2. [file elife-108048-fig9-data2.zip › Figure 9/Figure 9 G-USP8-Actin.tif]

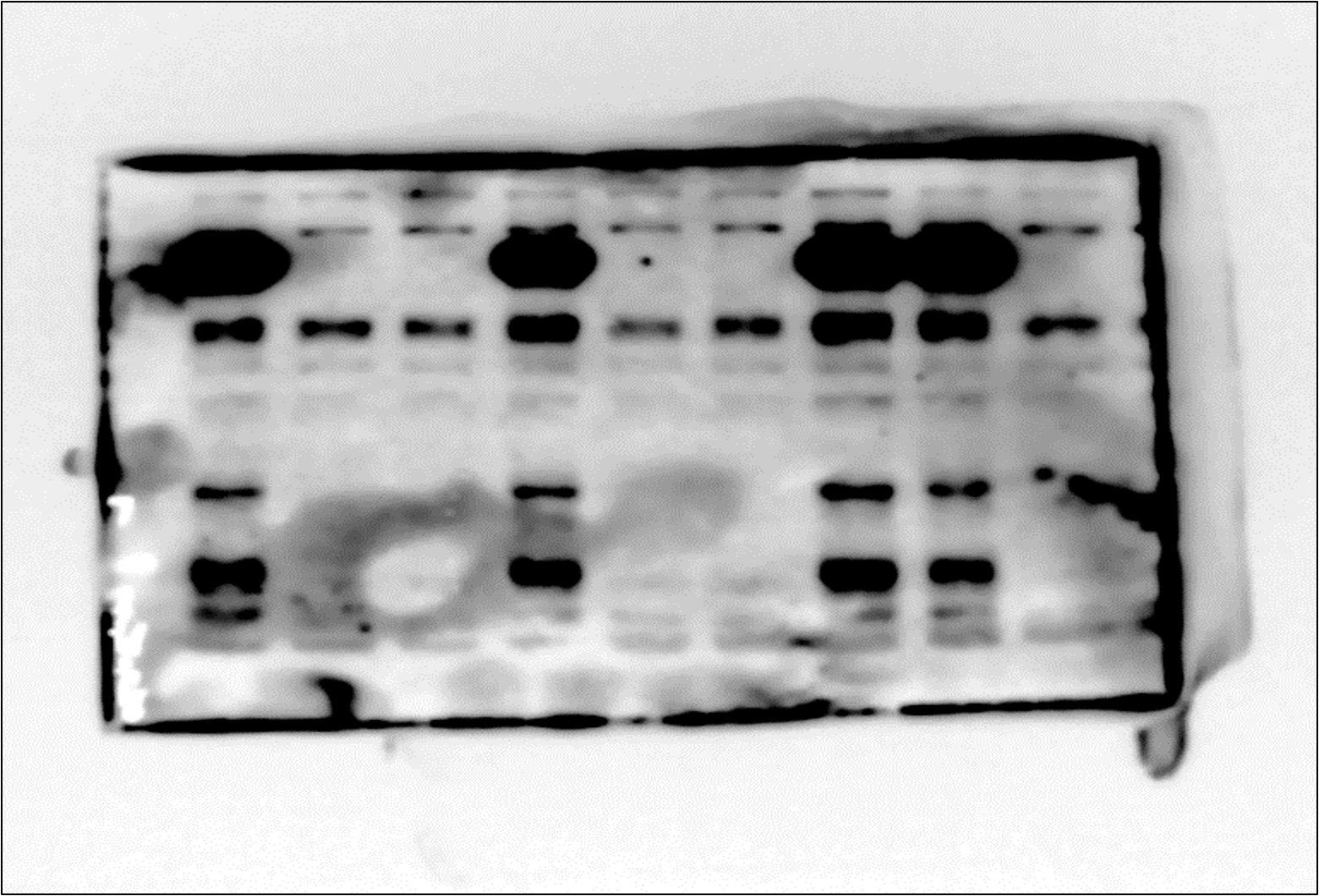

Supplement: Figure 9—source data 2. [file elife-108048-fig9-data2.zip › Figure 9/Figure 9 G-USP8.tif]

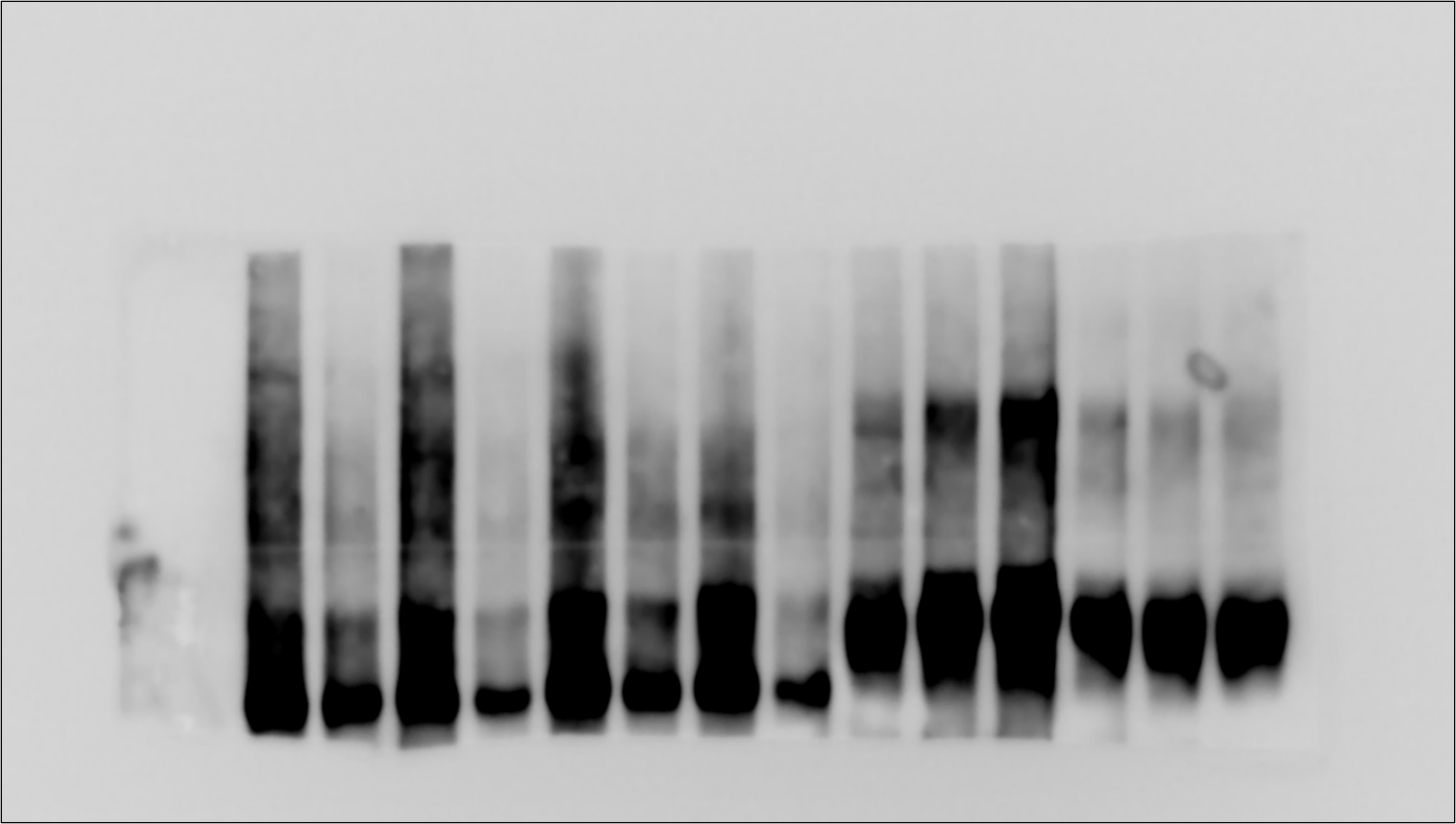

Supplement: Figure 9—source data 2. [file elife-108048-fig9-data2.zip › Figure 9/Figure 9 H-IP-HA.tif]

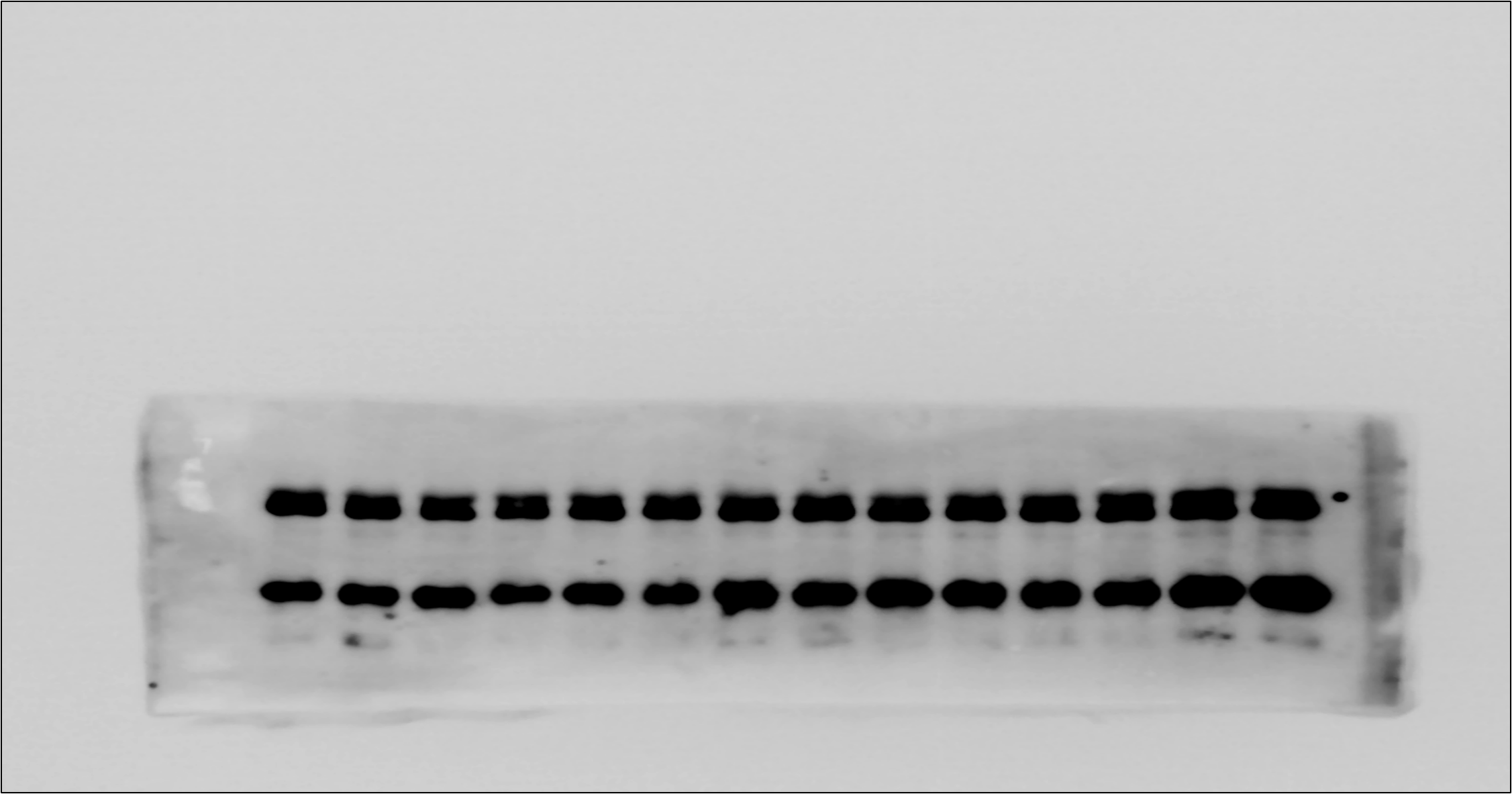

Supplement: Figure 9—source data 2. [file elife-108048-fig9-data2.zip › Figure 9/Figure 9 H-IP-Myc.tif]

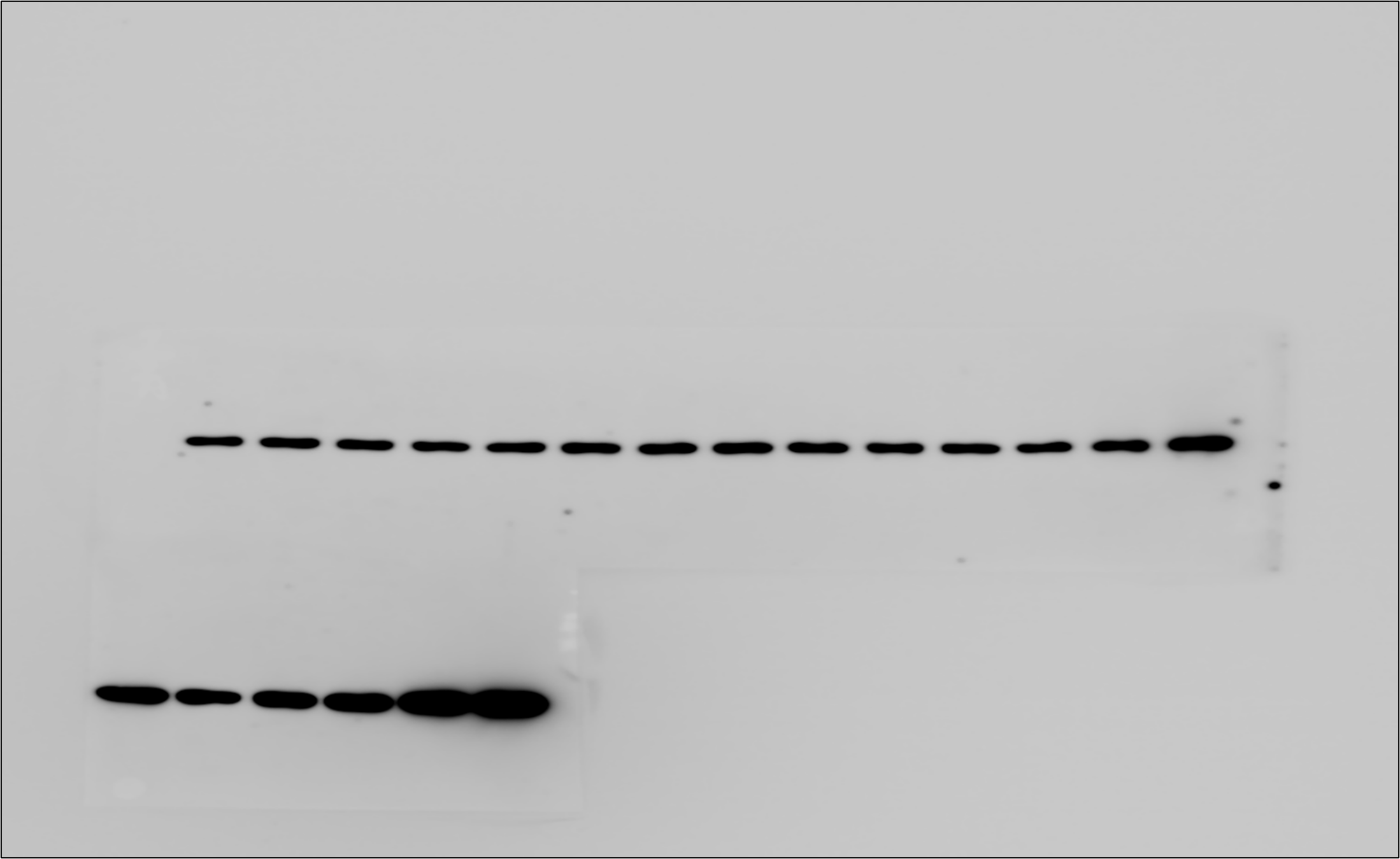

Supplement: Figure 9—source data 2. [file elife-108048-fig9-data2.zip › Figure 9/Figure 9 H-WCL-Actin.tif]

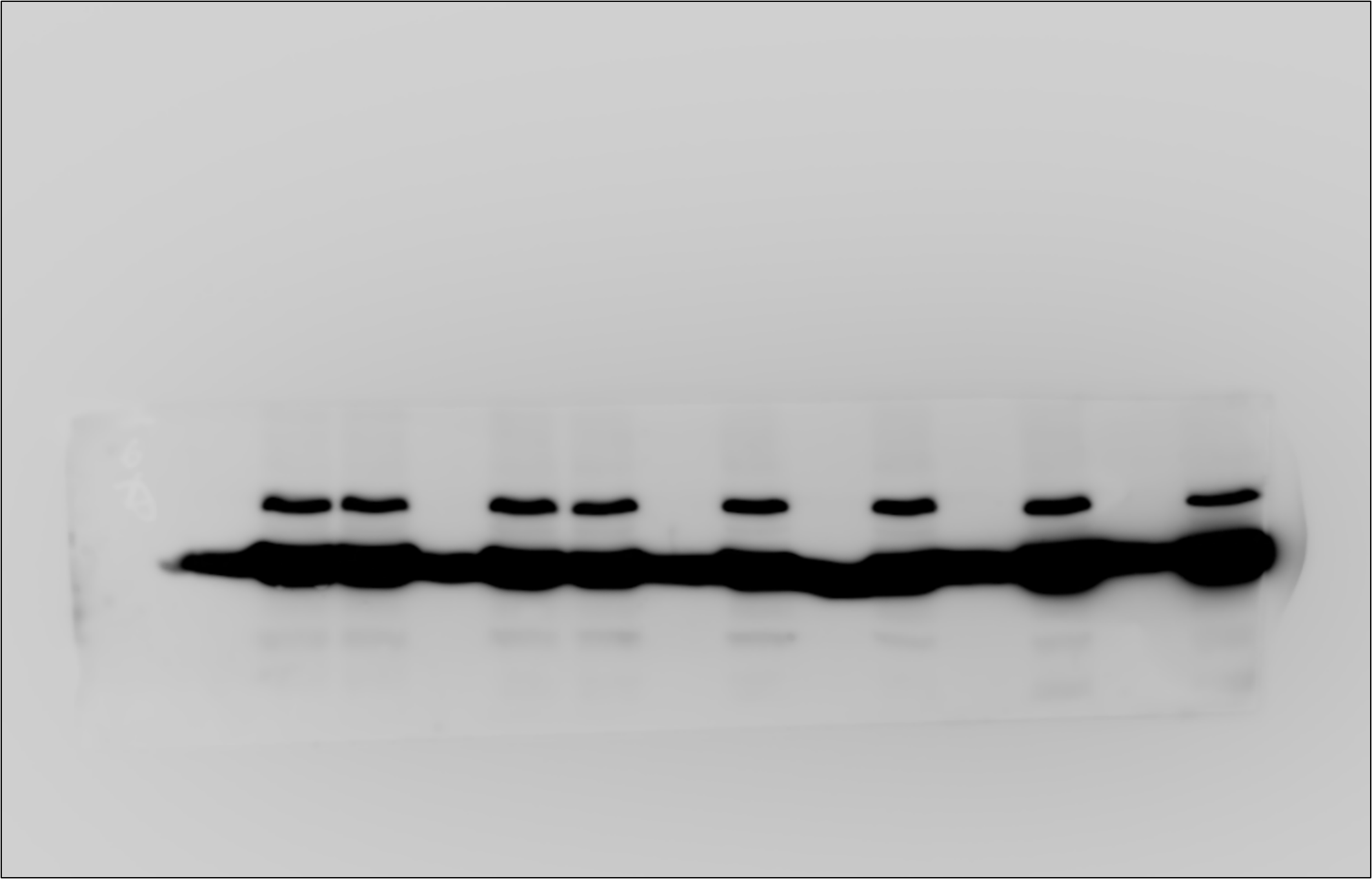

Supplement: Figure 9—source data 2. [file elife-108048-fig9-data2.zip › Figure 9/Figure 9 H-WCL-Flag.tif]

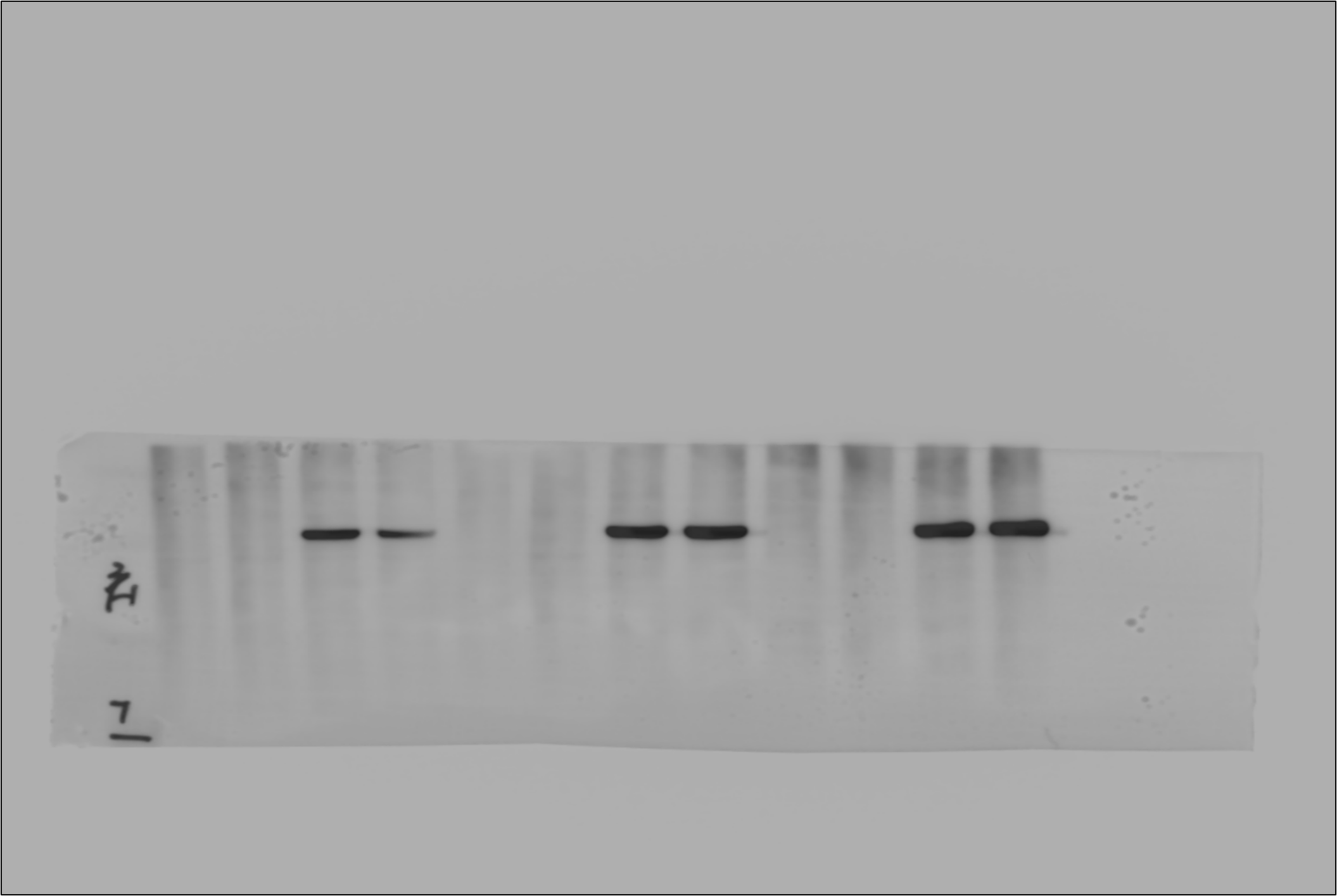

Supplement: Figure 9—source data 2. [file elife-108048-fig9-data2.zip › Figure 9/Figure 9 H-WCL-HA-USP8.tif]

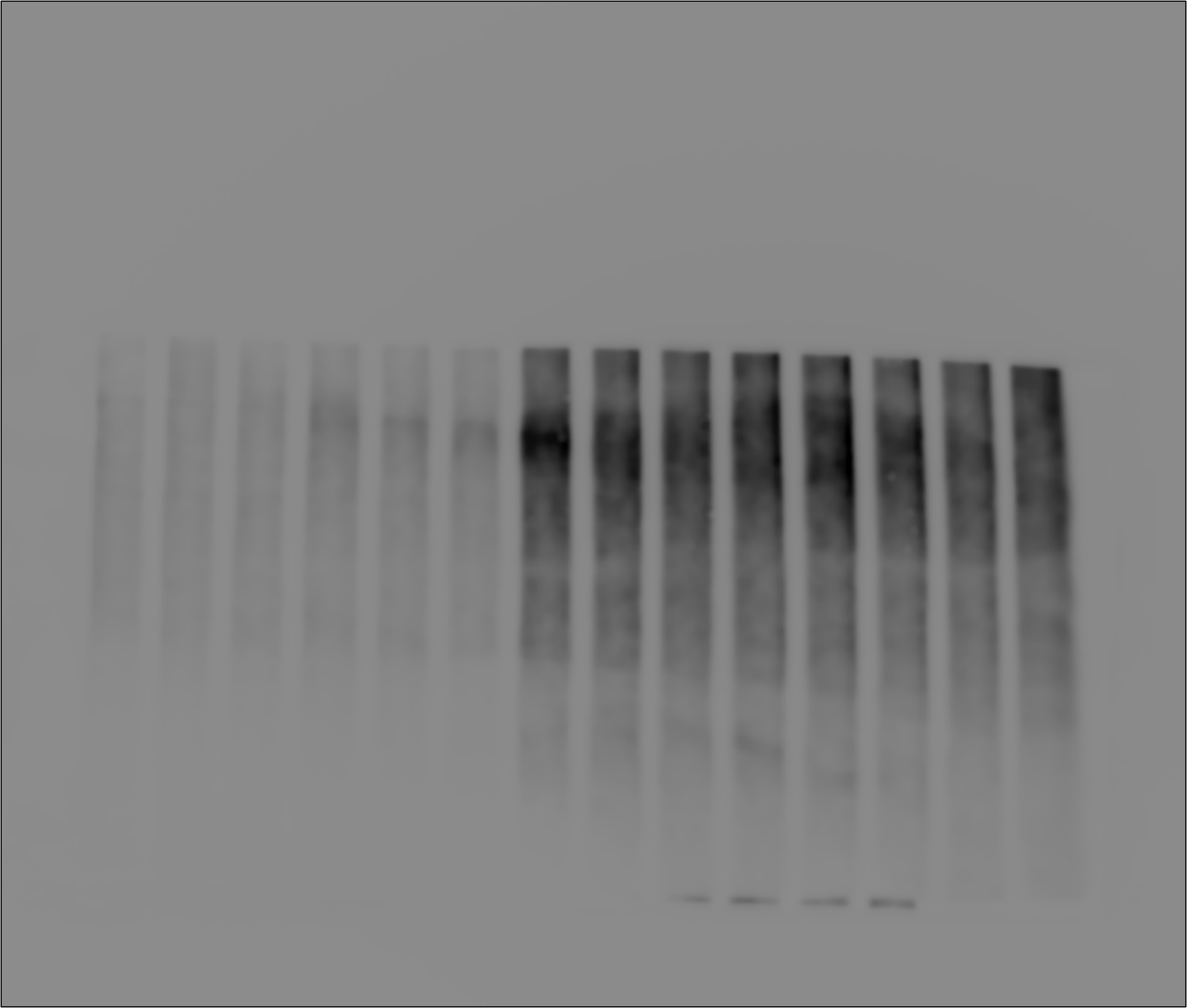

Supplement: Figure 9—source data 2. [file elife-108048-fig9-data2.zip › Figure 9/Figure 9 H-WCL-HA.tif]

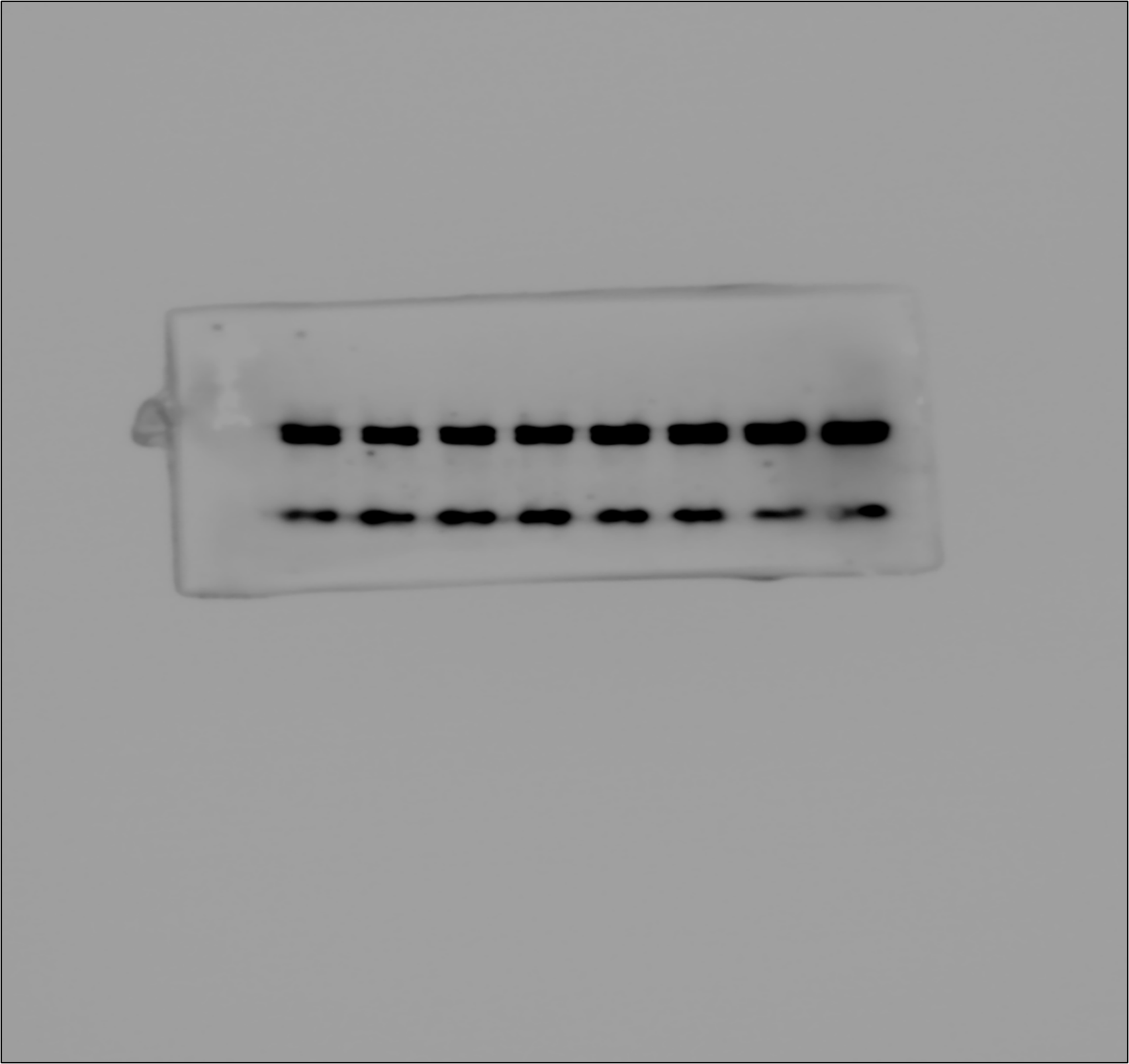

Supplement: Figure 9—source data 2. [file elife-108048-fig9-data2.zip › Figure 9/Figure 9 H-WCL-Myc.tif]

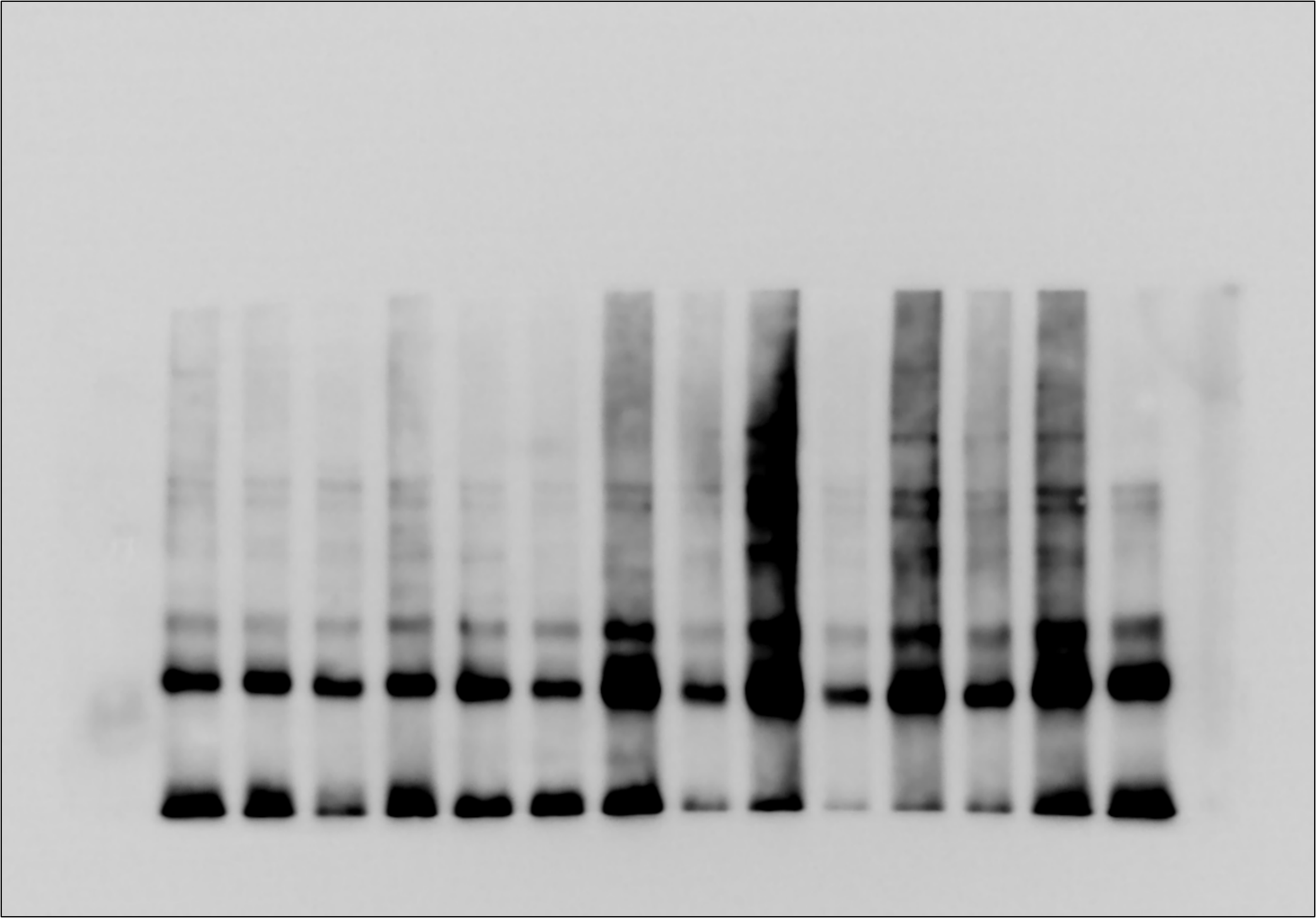

Supplement: Figure 9—source data 2. [file elife-108048-fig9-data2.zip › Figure 9/Figure 9 I-IP-HA.tif]

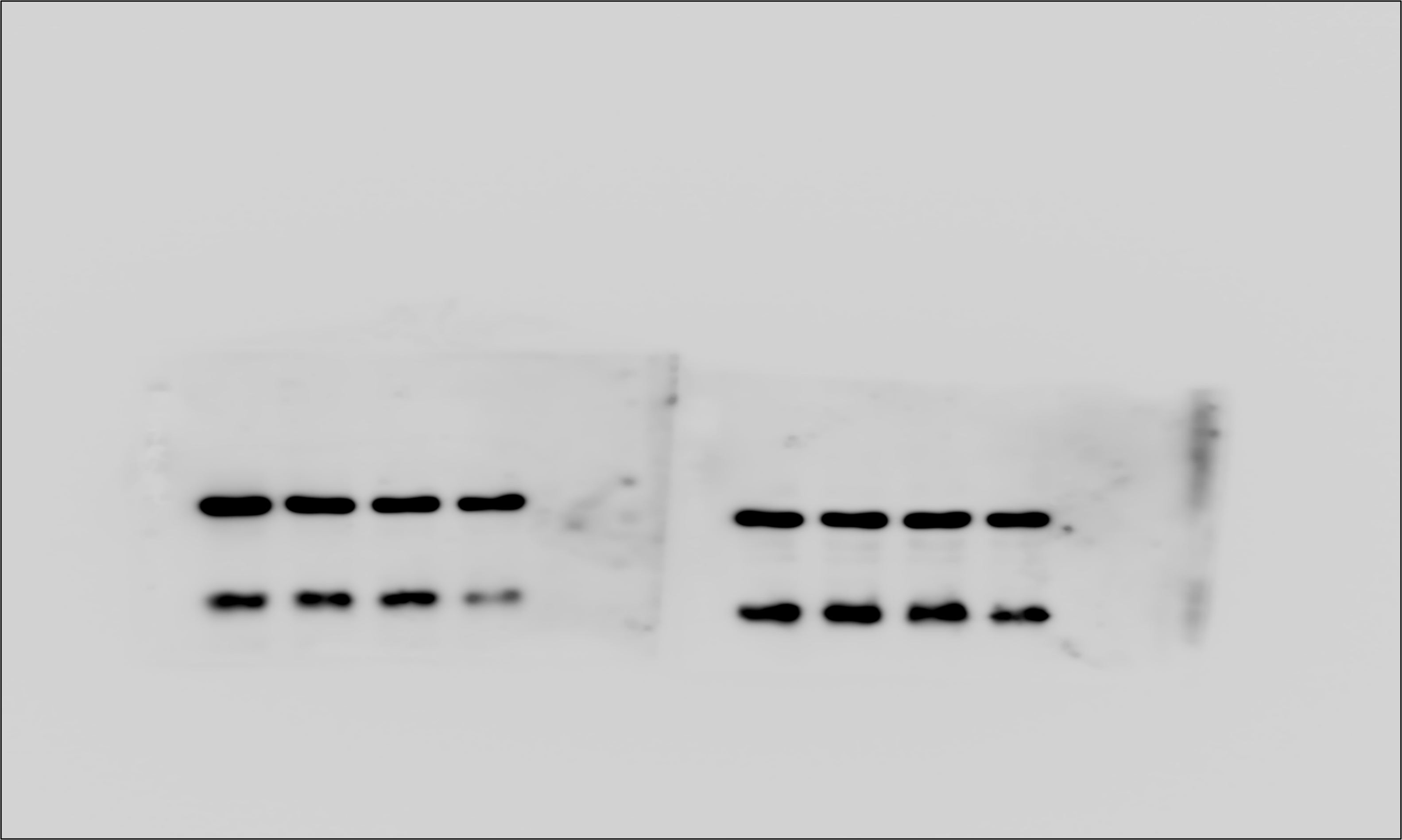

Supplement: Figure 9—source data 2. [file elife-108048-fig9-data2.zip › Figure 9/Figure 9 I-IP-Myc.tif]

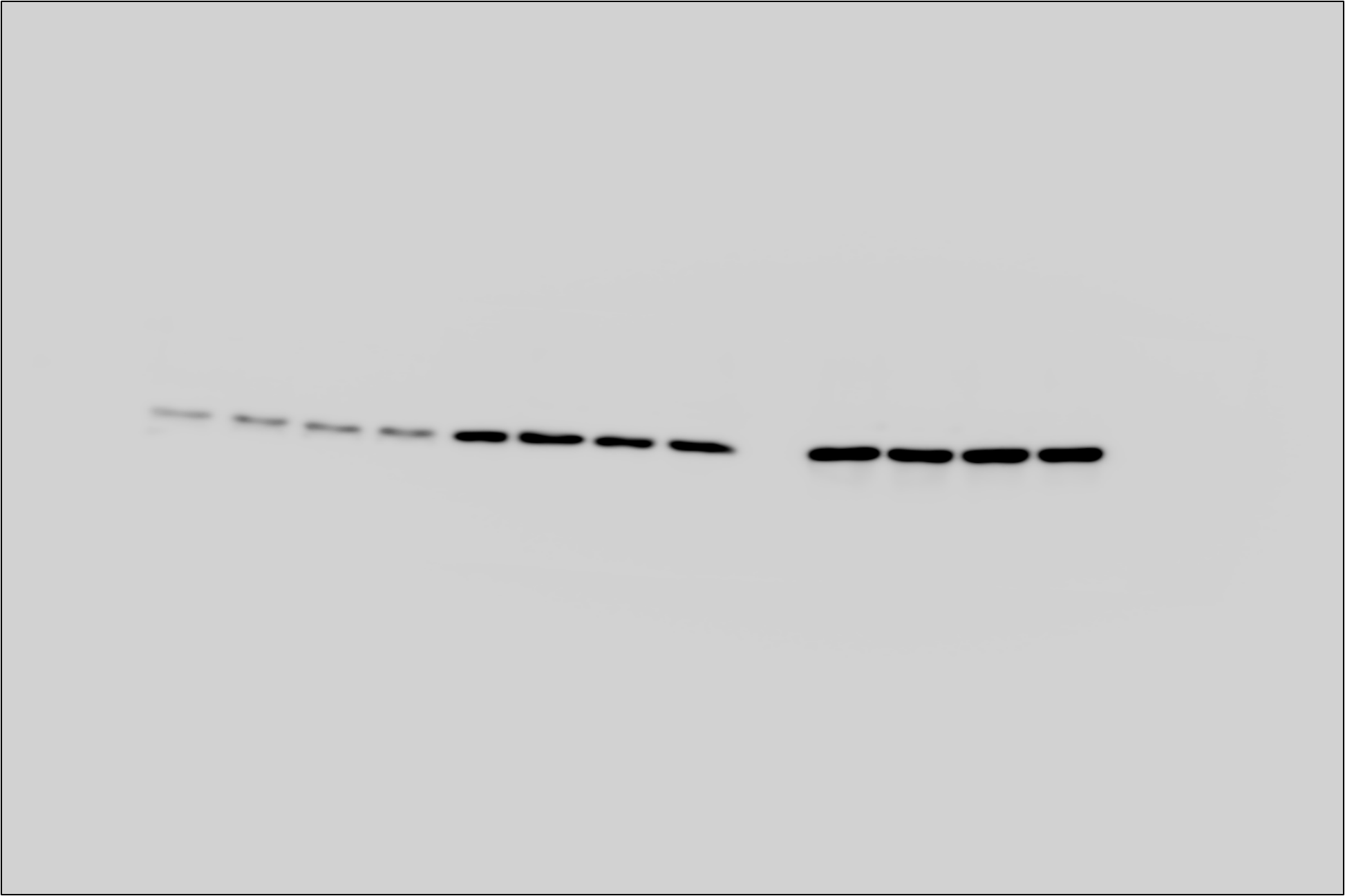

Supplement: Figure 9—source data 2. [file elife-108048-fig9-data2.zip › Figure 9/Figure 9 IP-WCL-Actin.tif]

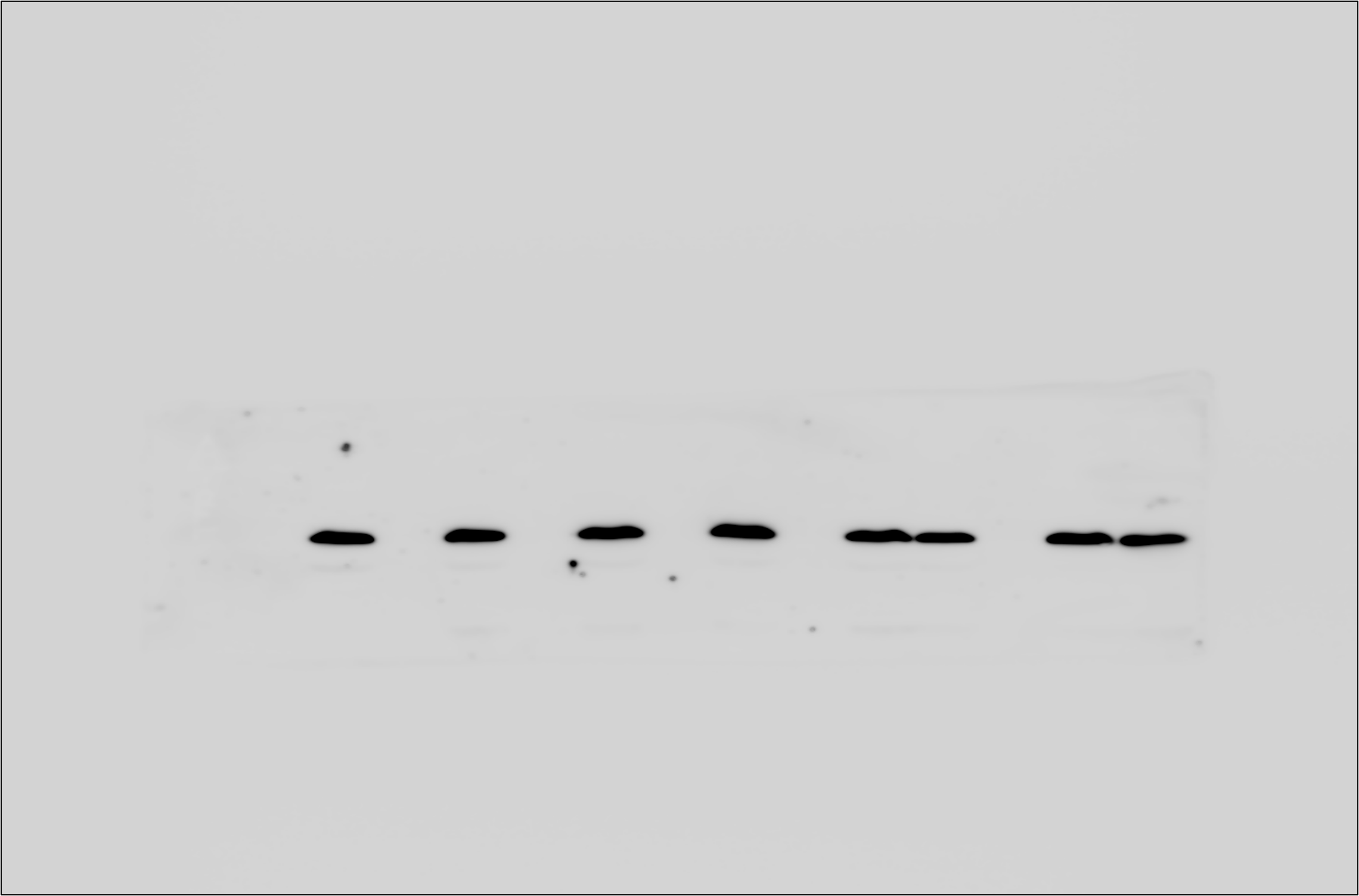

Supplement: Figure 9—source data 2. [file elife-108048-fig9-data2.zip › Figure 9/Figure 9 IP-WCL-Flag.tif]

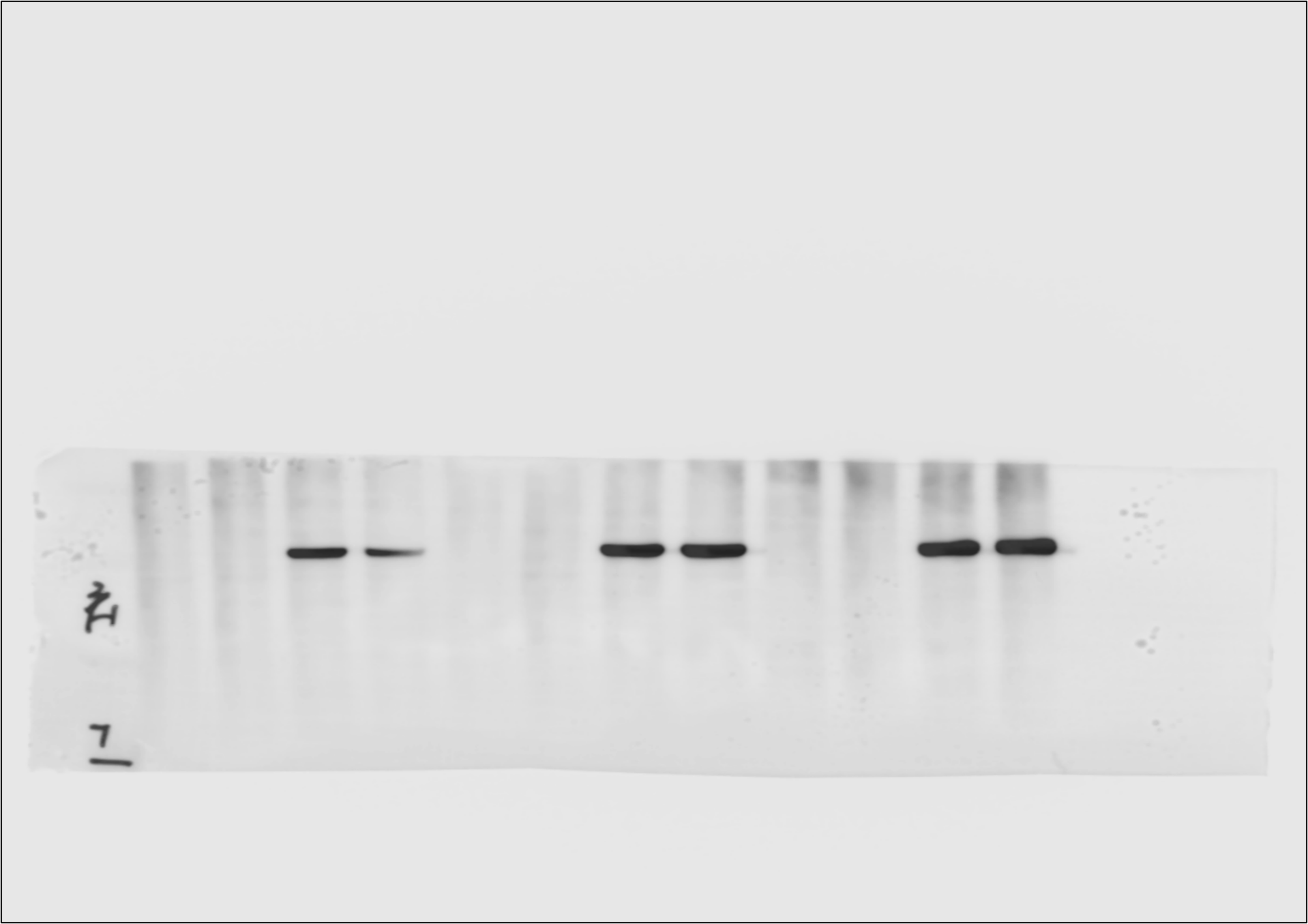

Supplement: Figure 9—source data 2. [file elife-108048-fig9-data2.zip › Figure 9/Figure 9 IP-WCL-HA-USP8.tif]

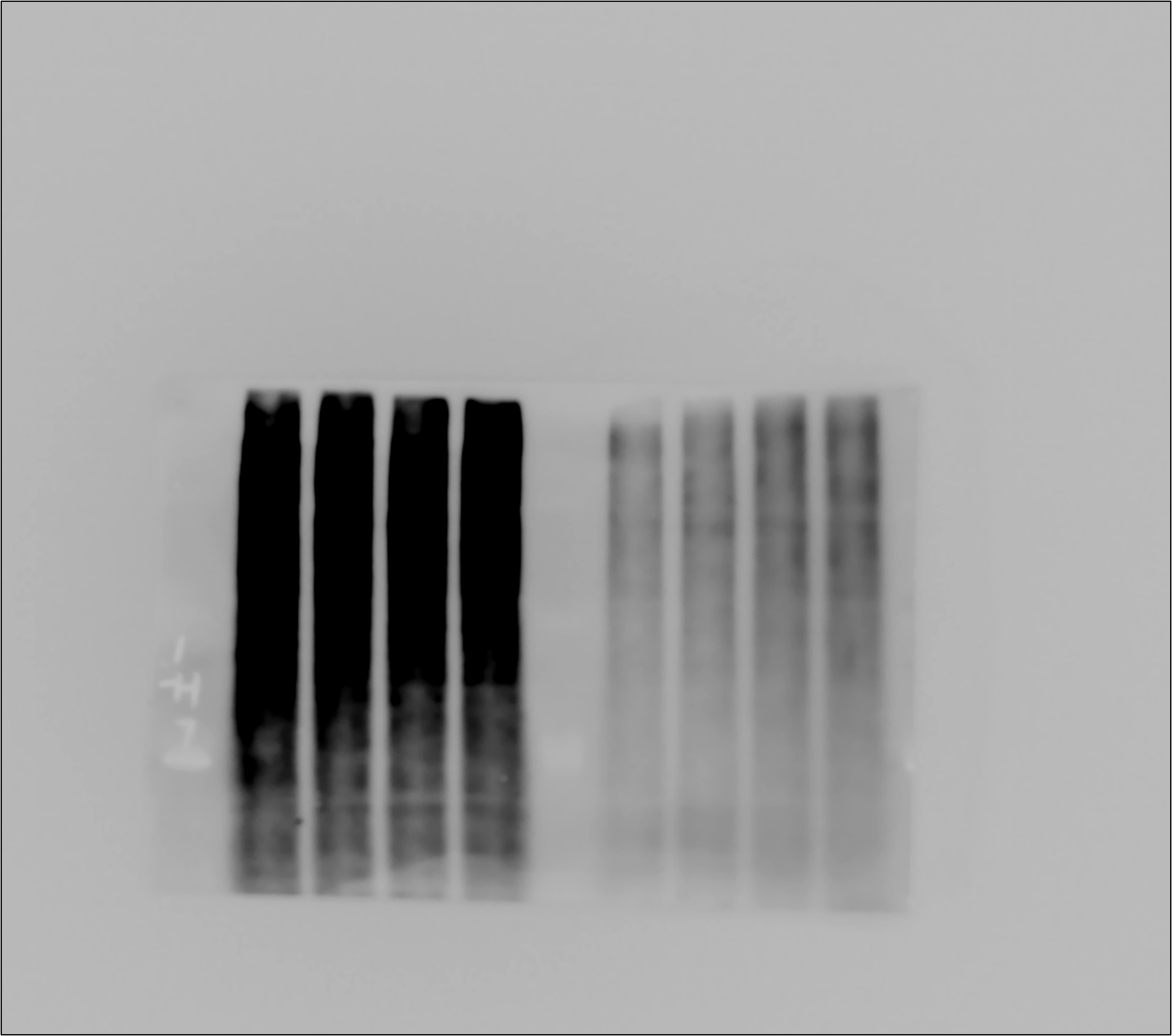

Supplement: Figure 9—source data 2. [file elife-108048-fig9-data2.zip › Figure 9/Figure 9 IP-WCL-HA.tif]

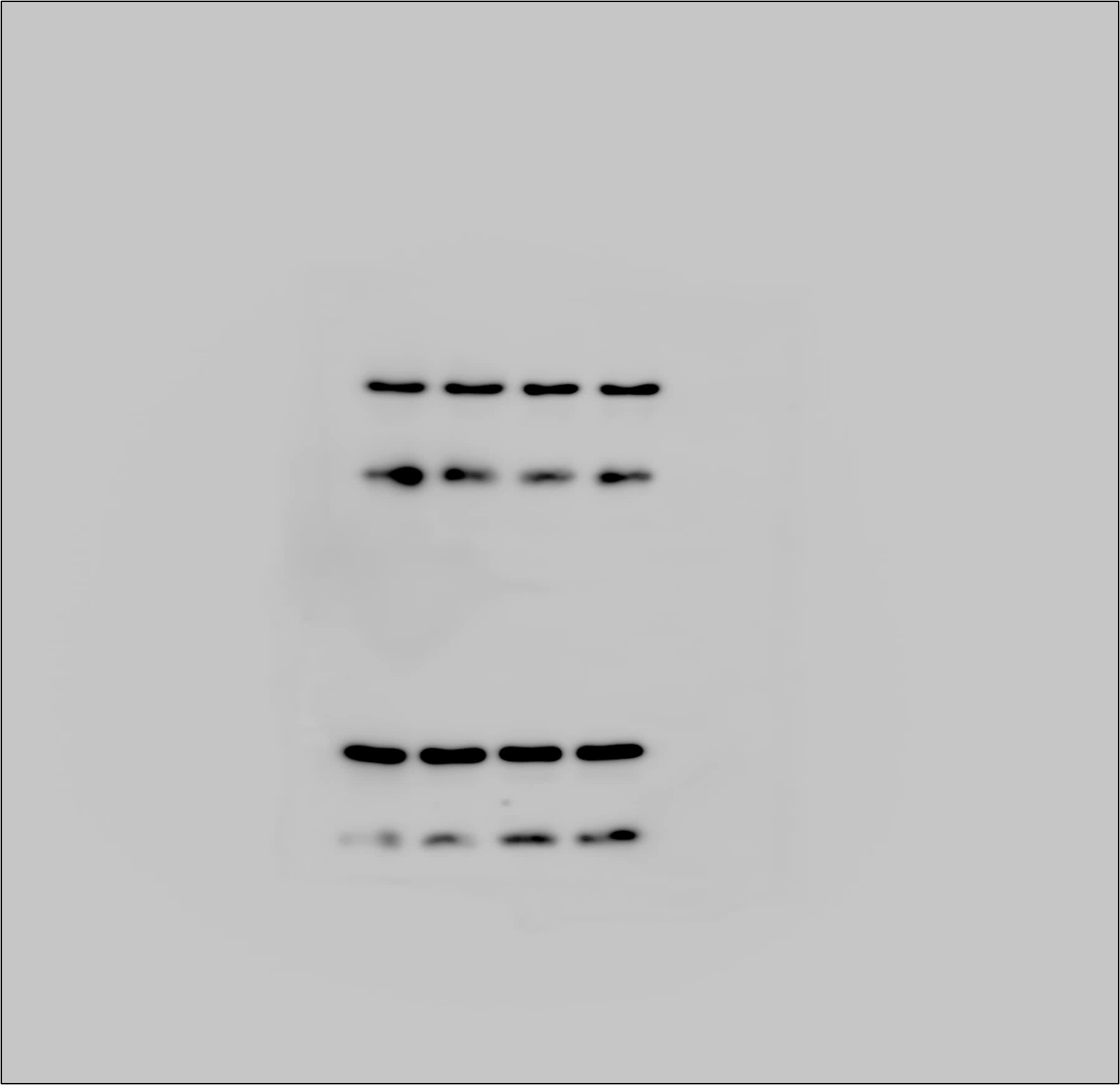

Supplement: Figure 9—source data 2. [file elife-108048-fig9-data2.zip › Figure 9/Figure 9 IP-WCL-Myc.tif]

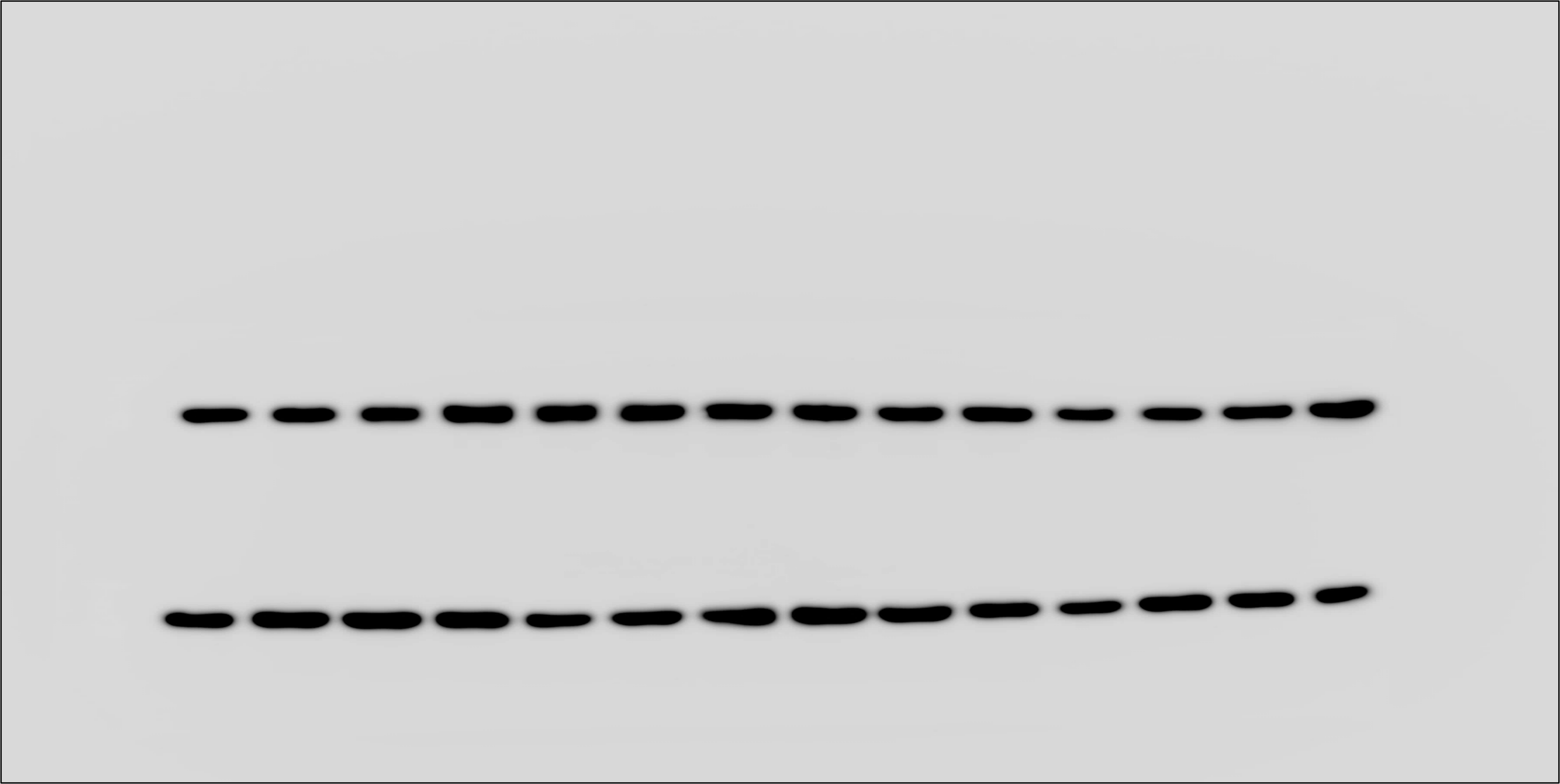

Supplement: Figure 9—source data 2. [file elife-108048-fig9-data2.zip › Figure 9/Figure 9 K-Actin.tif]

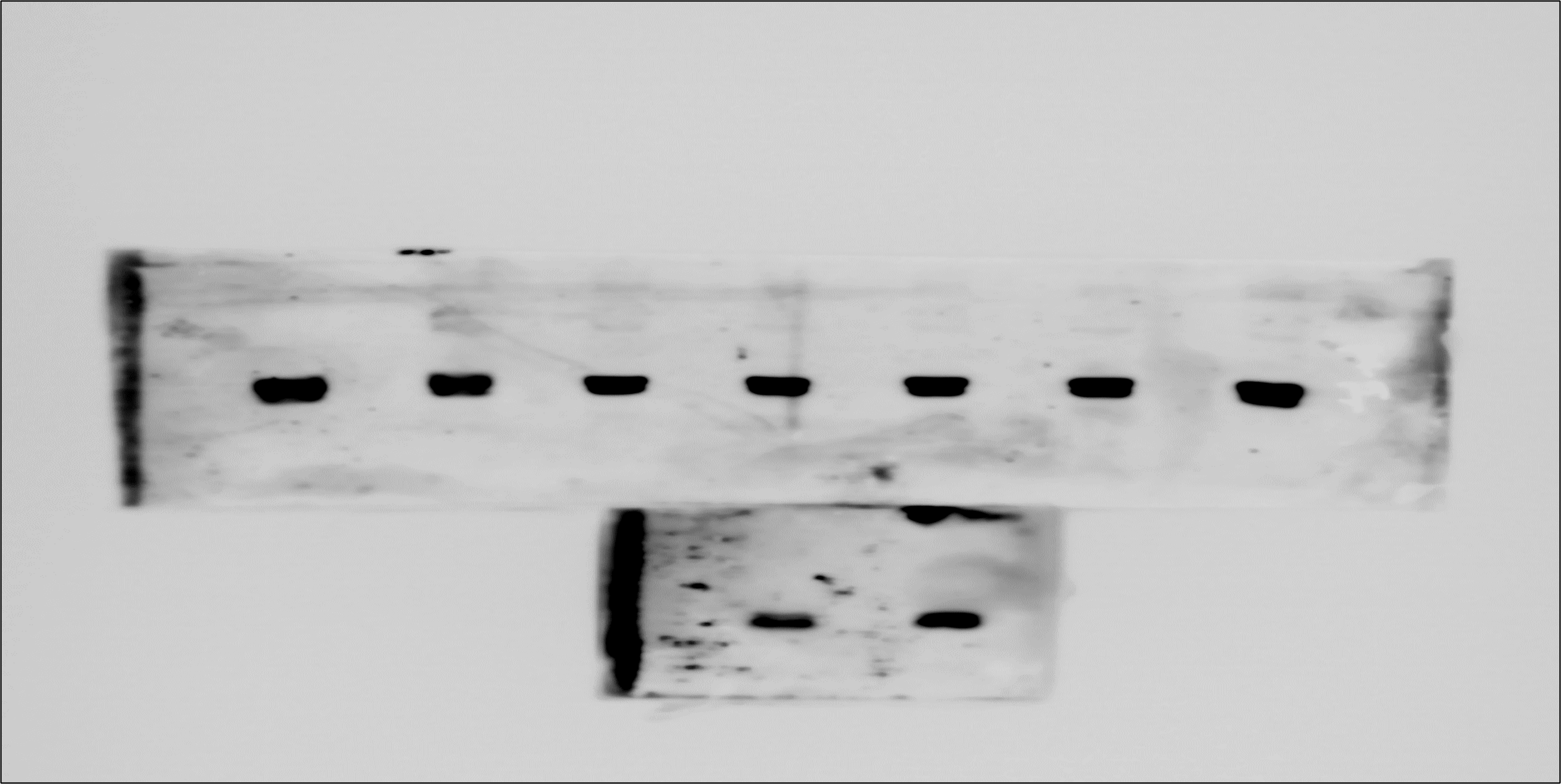

Supplement: Figure 9—source data 2. [file elife-108048-fig9-data2.zip › Figure 9/Figure 9 K-Flag.tif]

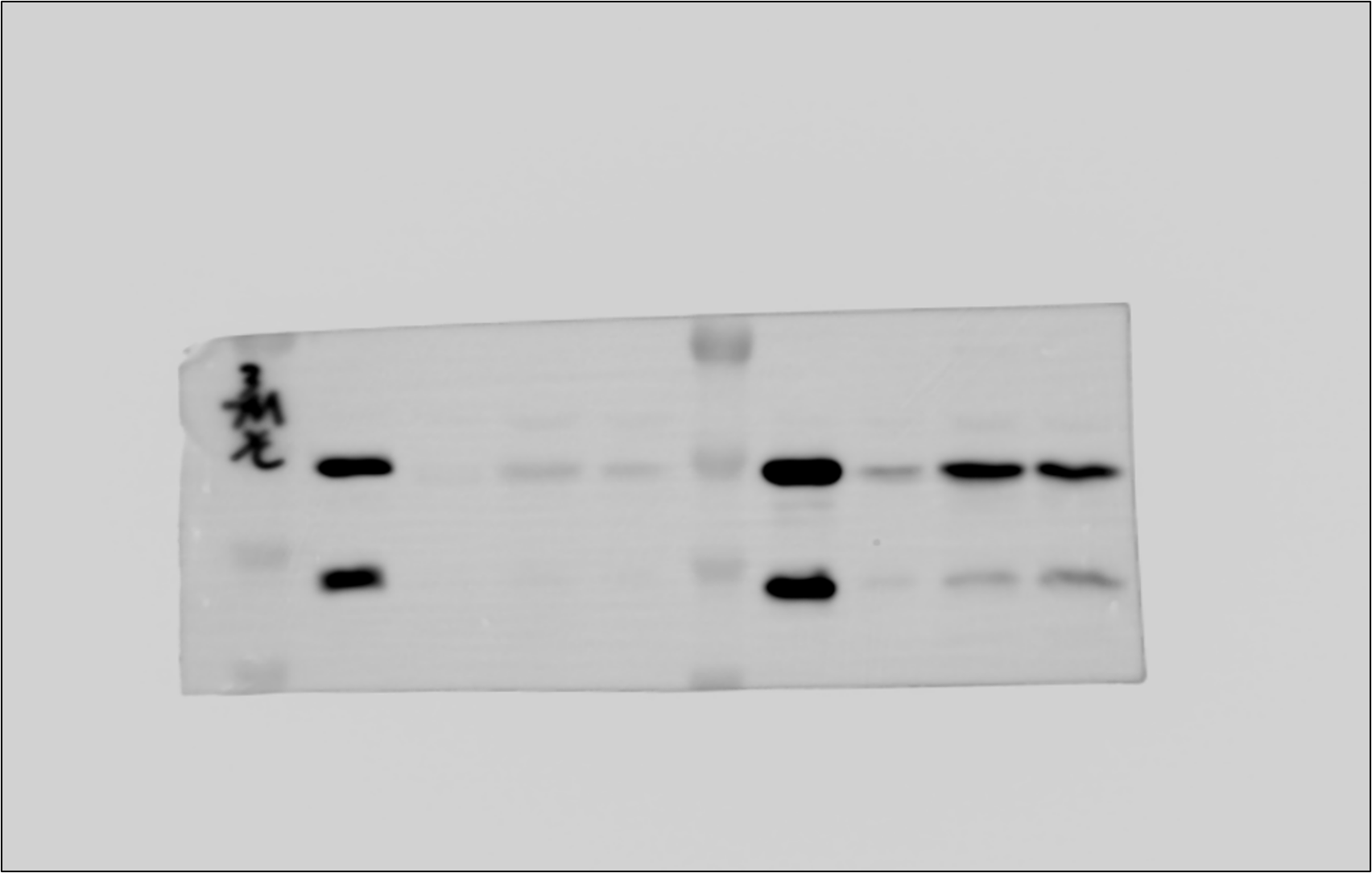

Supplement: Figure 9—source data 2. [file elife-108048-fig9-data2.zip › Figure 9/Figure 9 K-Myc.tif]

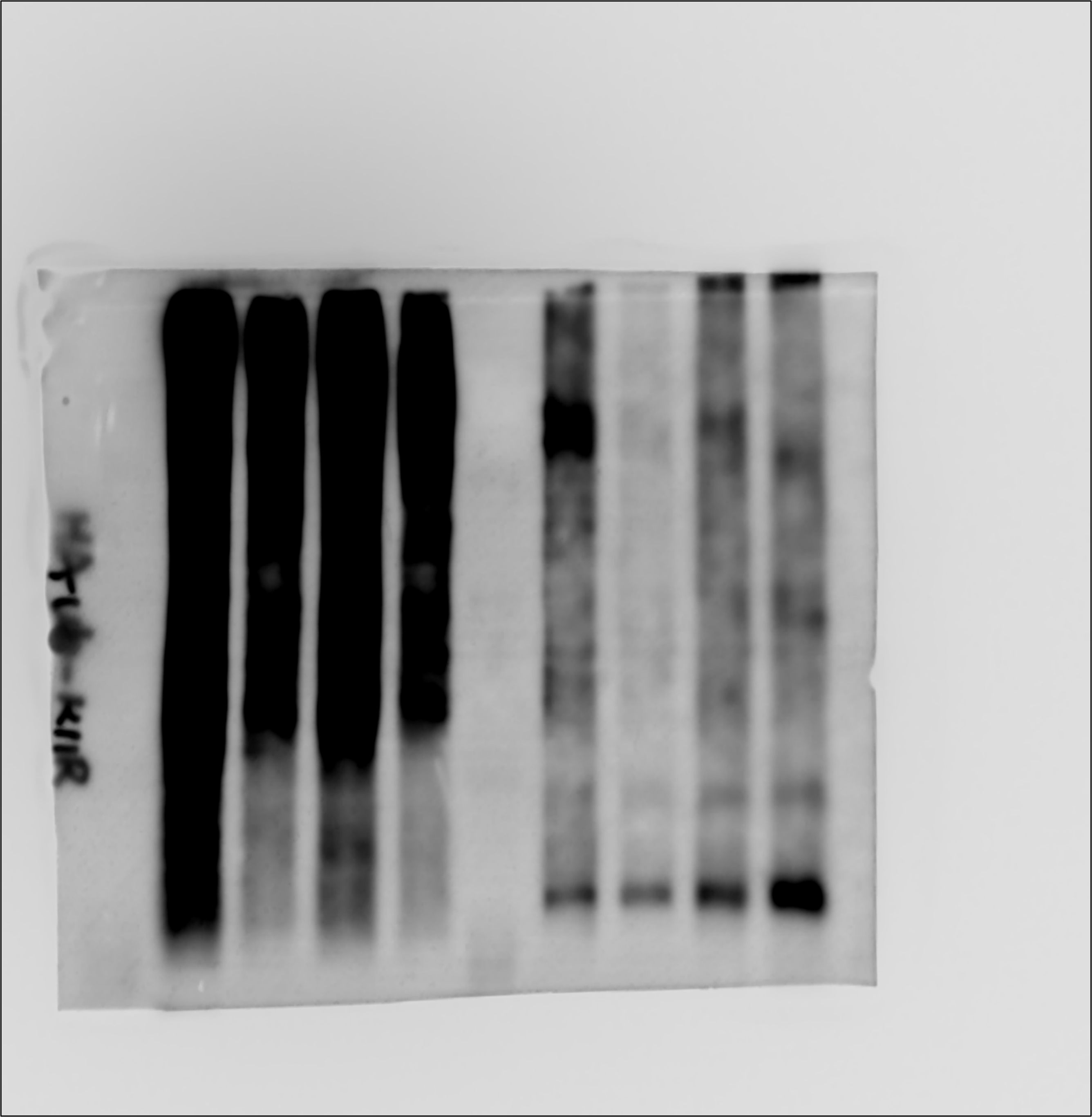

Supplement: Figure 9—source data 2. [file elife-108048-fig9-data2.zip › Figure 9/Figure 9 L-IP-HA.tif]

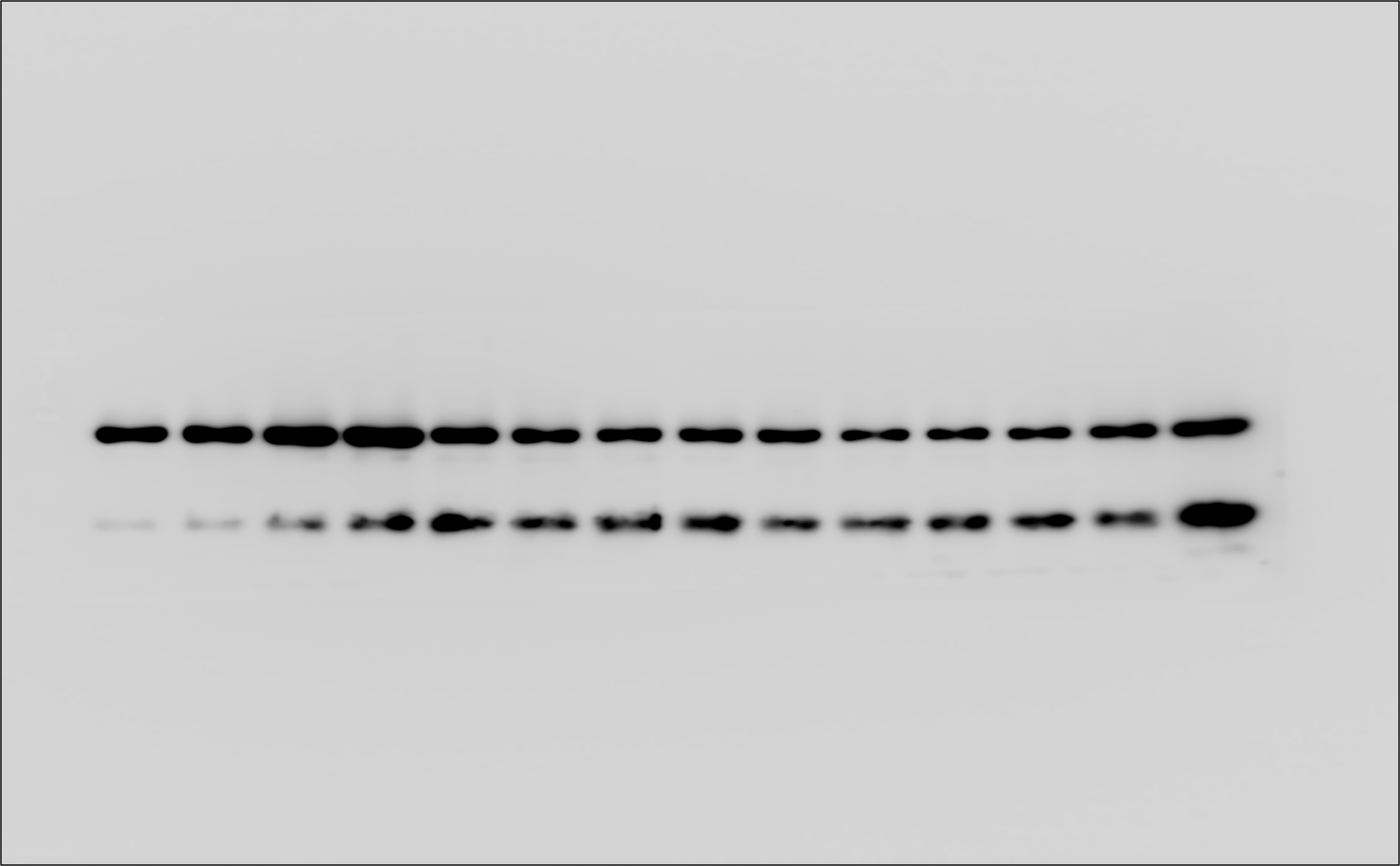

Supplement: Figure 9—source data 2. [file elife-108048-fig9-data2.zip › Figure 9/Figure 9 L-IP-Myc.tif]

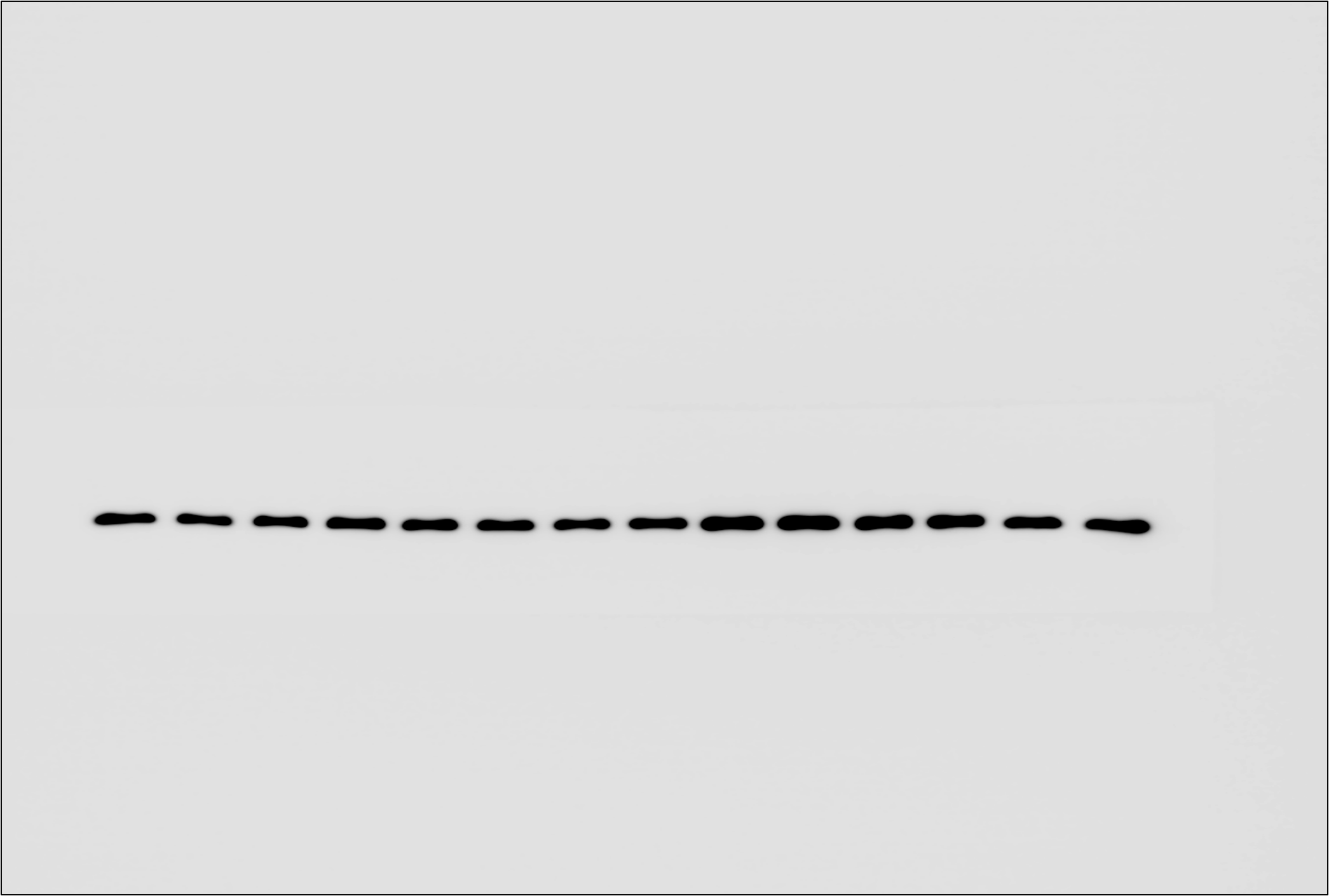

Supplement: Figure 9—source data 2. [file elife-108048-fig9-data2.zip › Figure 9/Figure 9 L-WCL-Actin.tif]

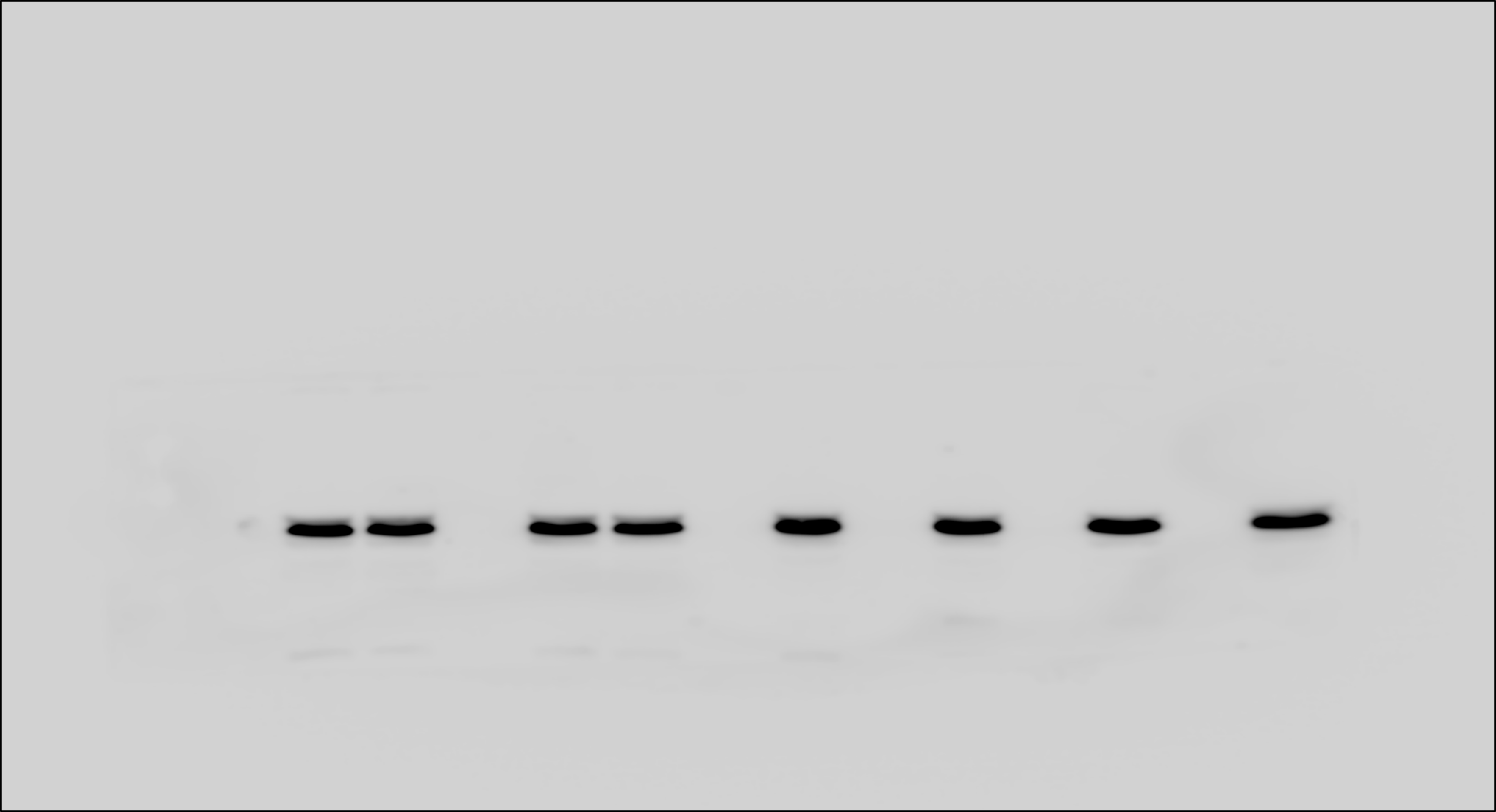

Supplement: Figure 9—source data 2. [file elife-108048-fig9-data2.zip › Figure 9/Figure 9 L-WCL-Flag.tif]

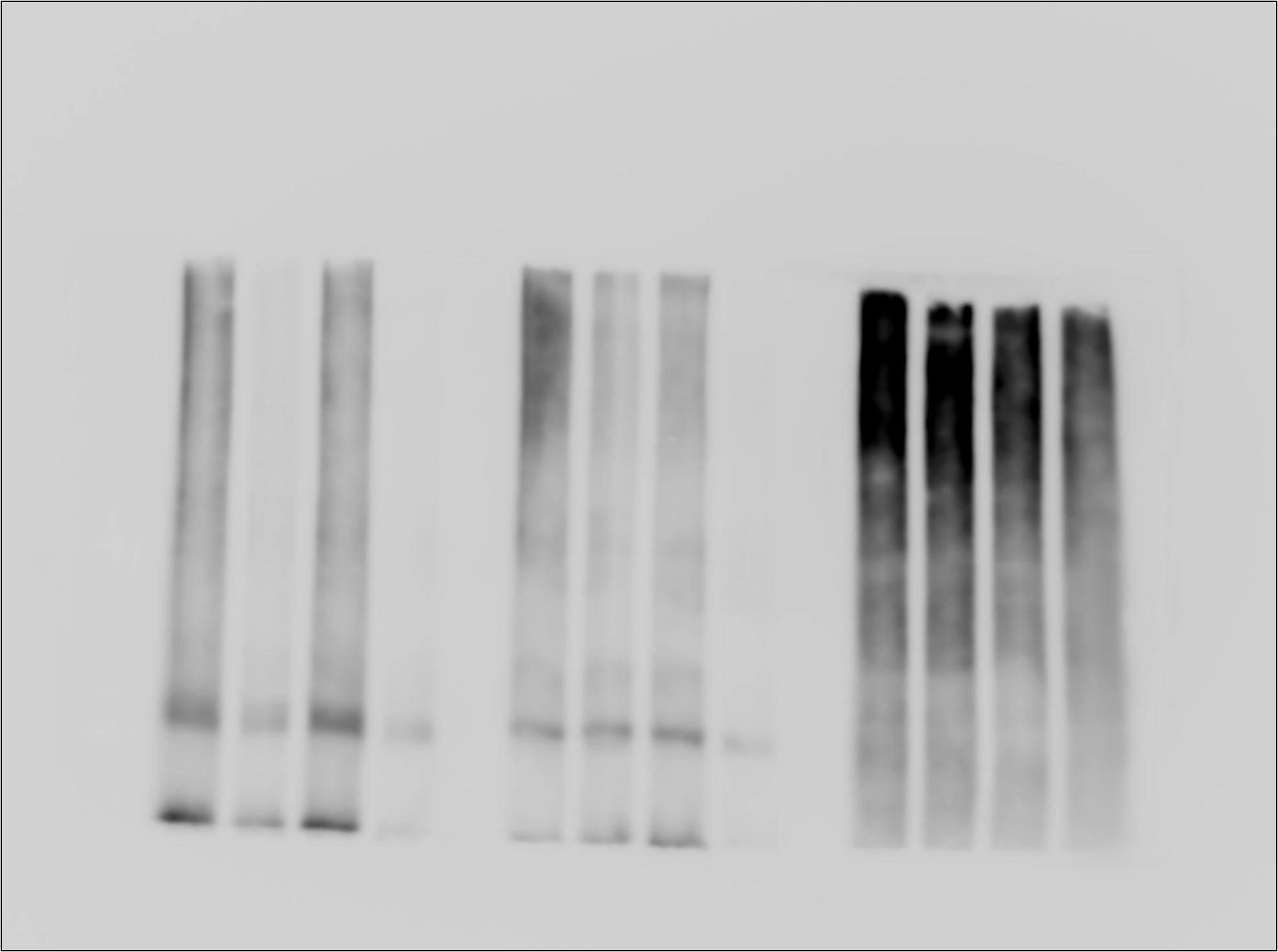

Supplement: Figure 9—source data 2. [file elife-108048-fig9-data2.zip › Figure 9/Figure 9 L-WCL-HA.tif]

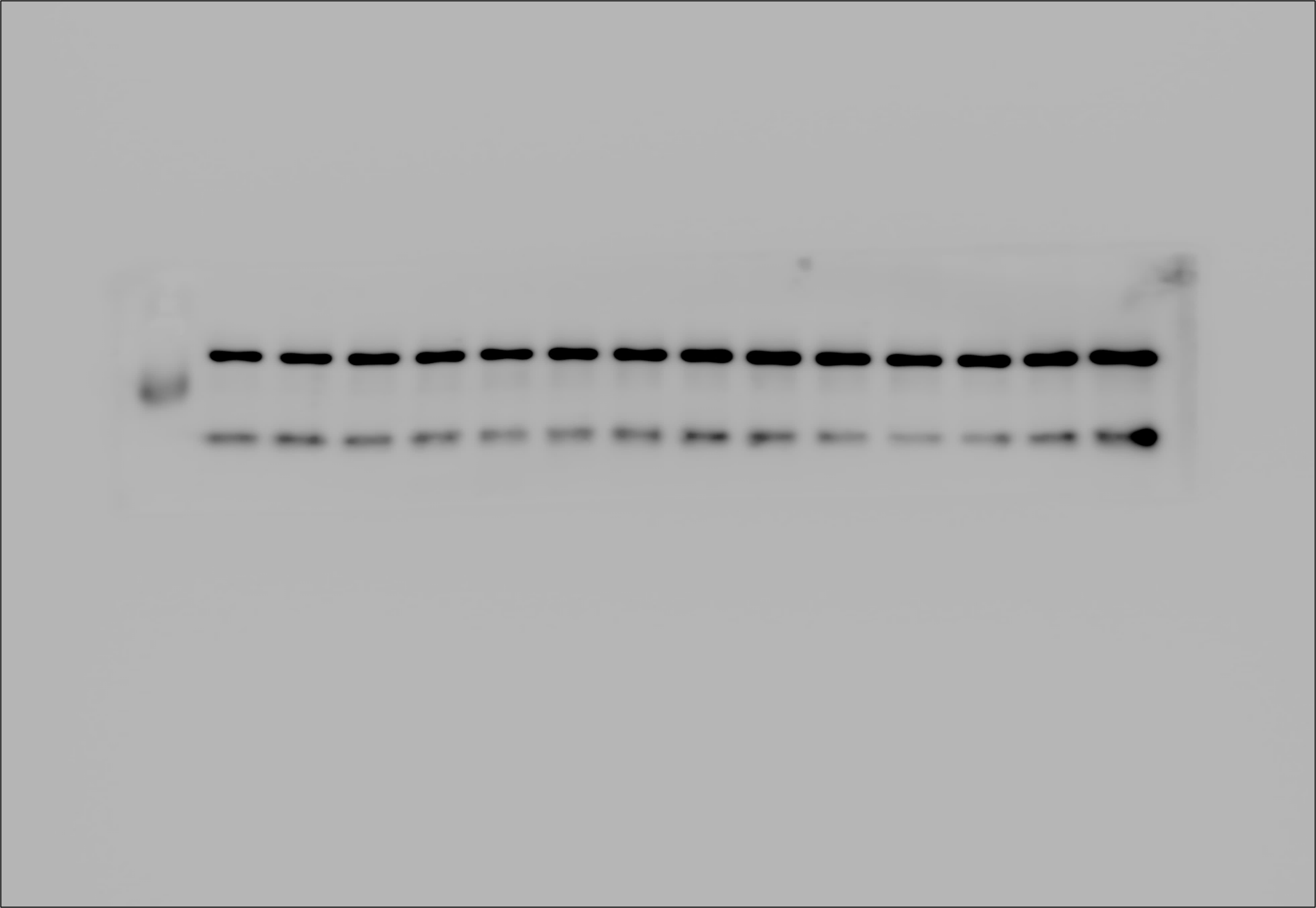

Supplement: Figure 9—source data 2. [file elife-108048-fig9-data2.zip › Figure 9/Figure 9 L-WCL-Myc.tif]

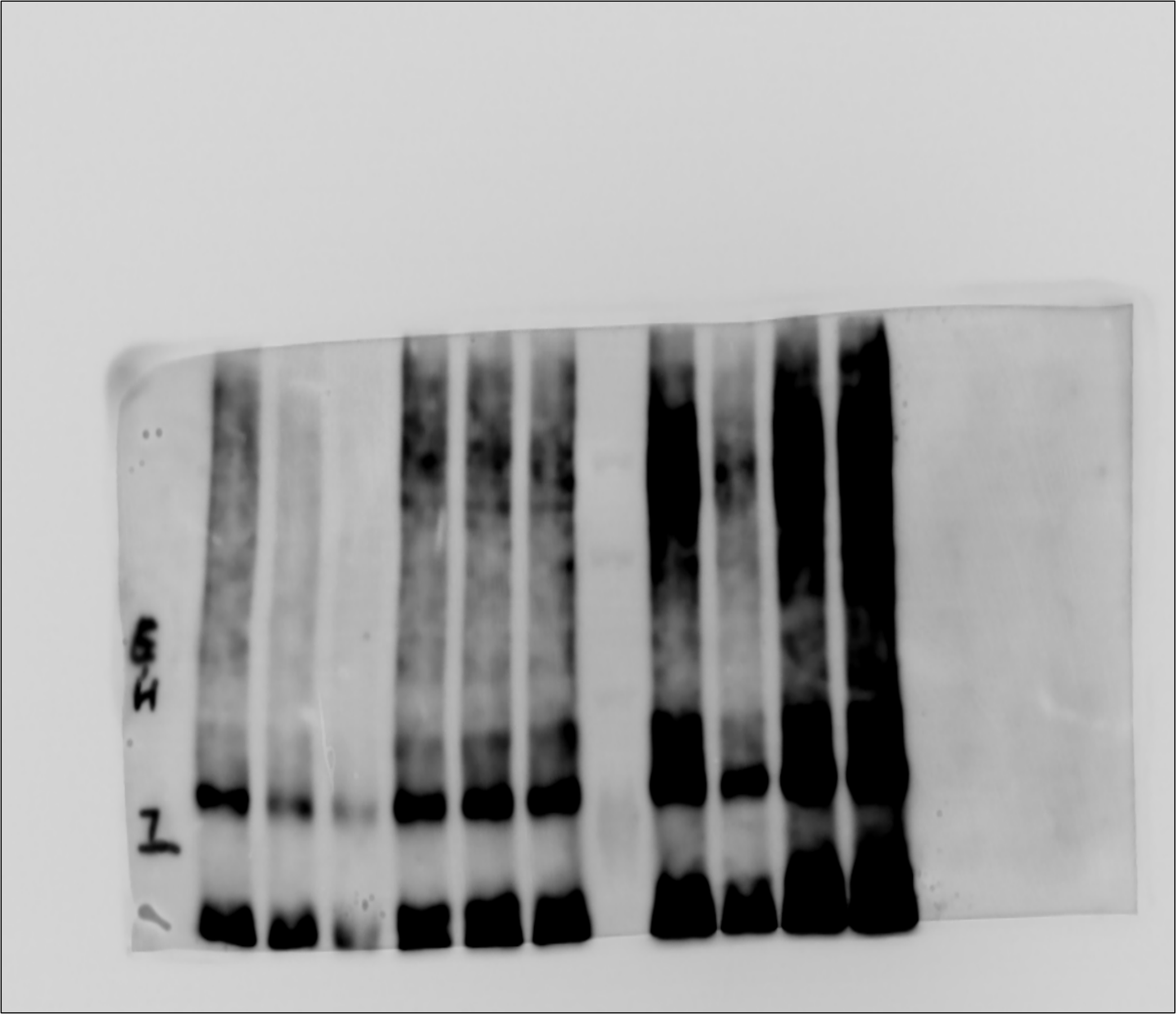

Supplement: Figure 9—source data 2. [file elife-108048-fig9-data2.zip › Figure 9/Figure 9 M-IP-HA.tif]

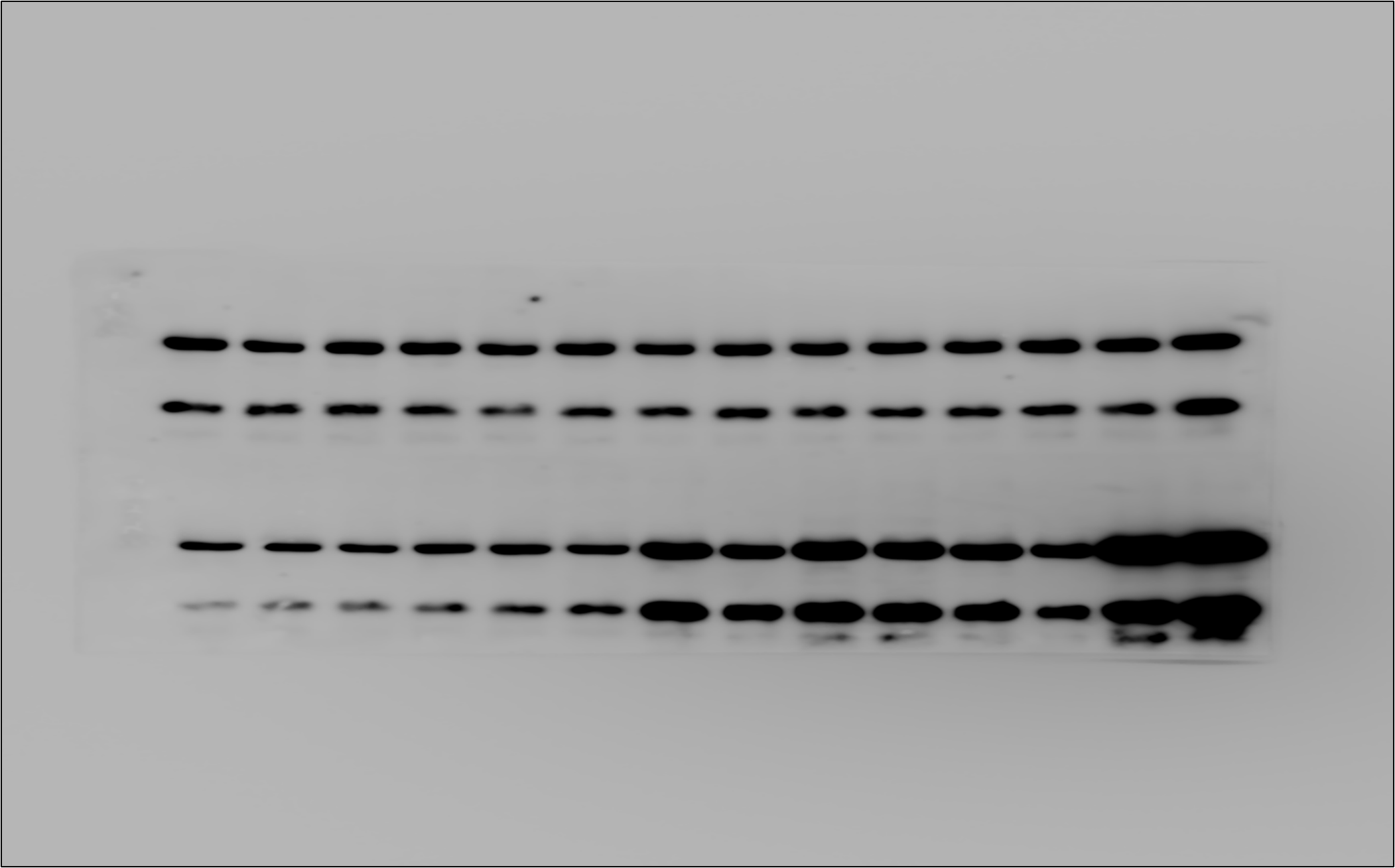

Supplement: Figure 9—source data 2. [file elife-108048-fig9-data2.zip › Figure 9/Figure 9 M-IP-Myc.tif]

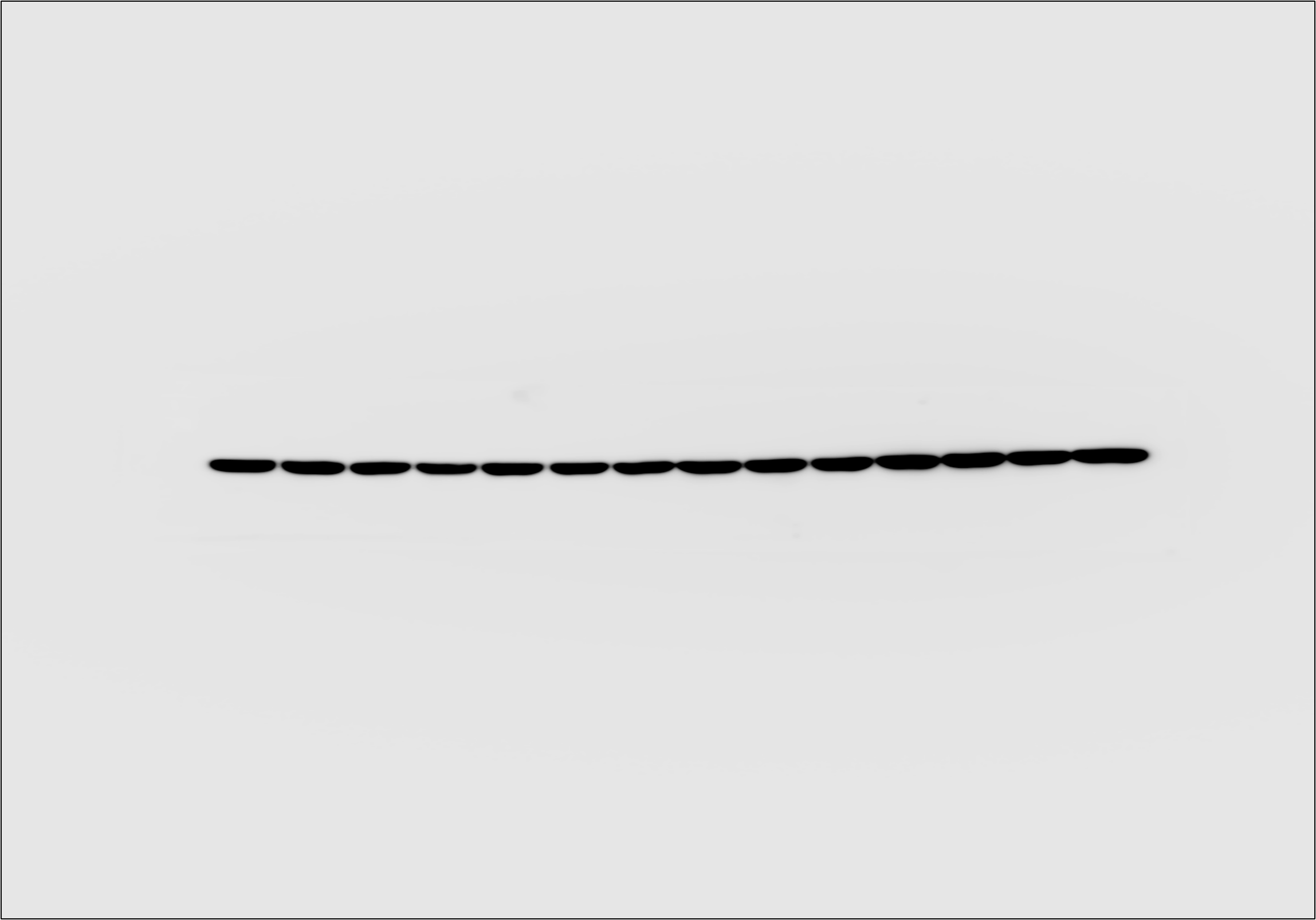

Supplement: Figure 9—source data 2. [file elife-108048-fig9-data2.zip › Figure 9/Figure 9 M-WCL-Actin.tif]

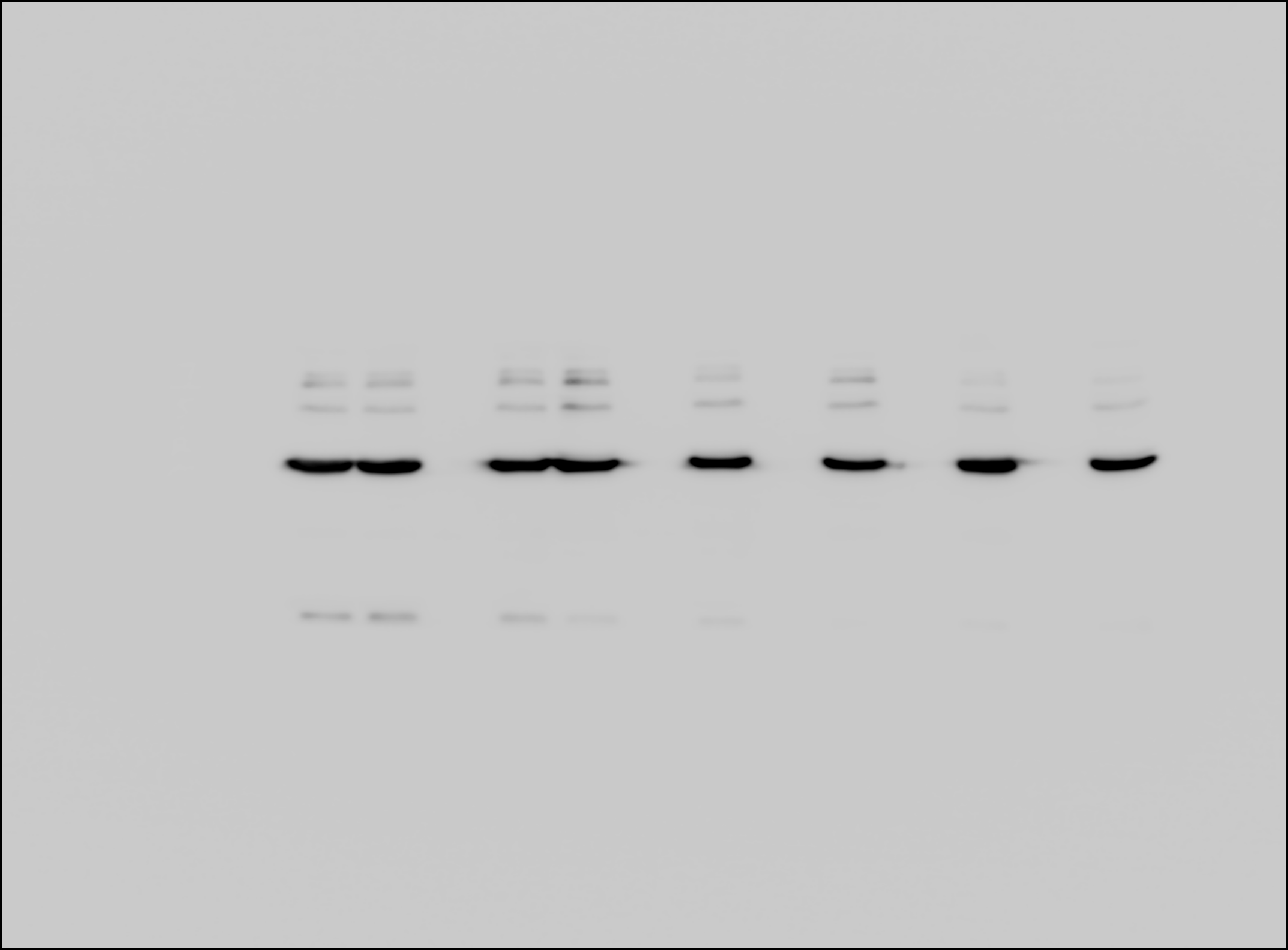

Supplement: Figure 9—source data 2. [file elife-108048-fig9-data2.zip › Figure 9/Figure 9 M-WCL-Flag.tif]

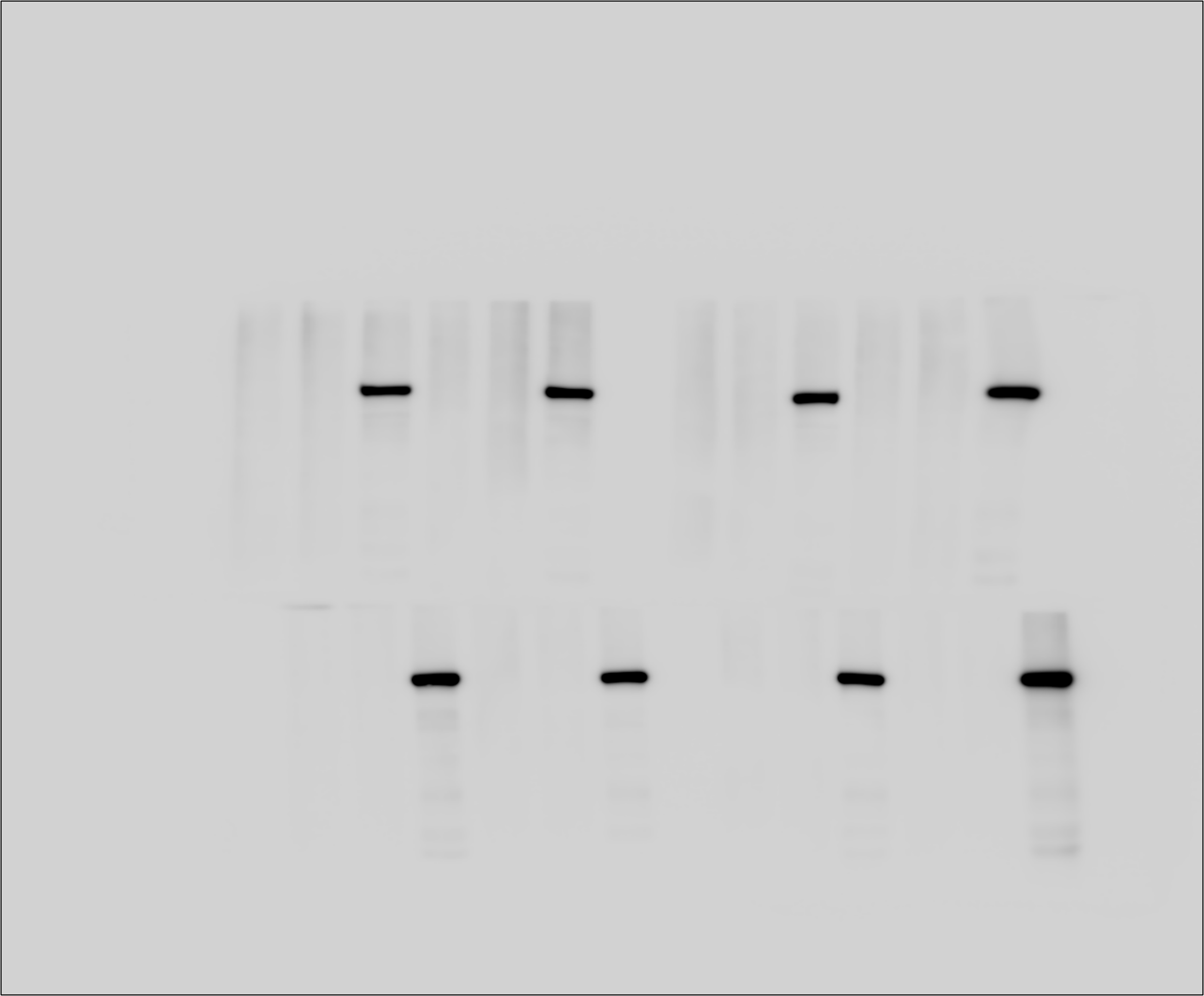

Supplement: Figure 9—source data 2. [file elife-108048-fig9-data2.zip › Figure 9/Figure 9 M-WCL-HA-USP8.tif]

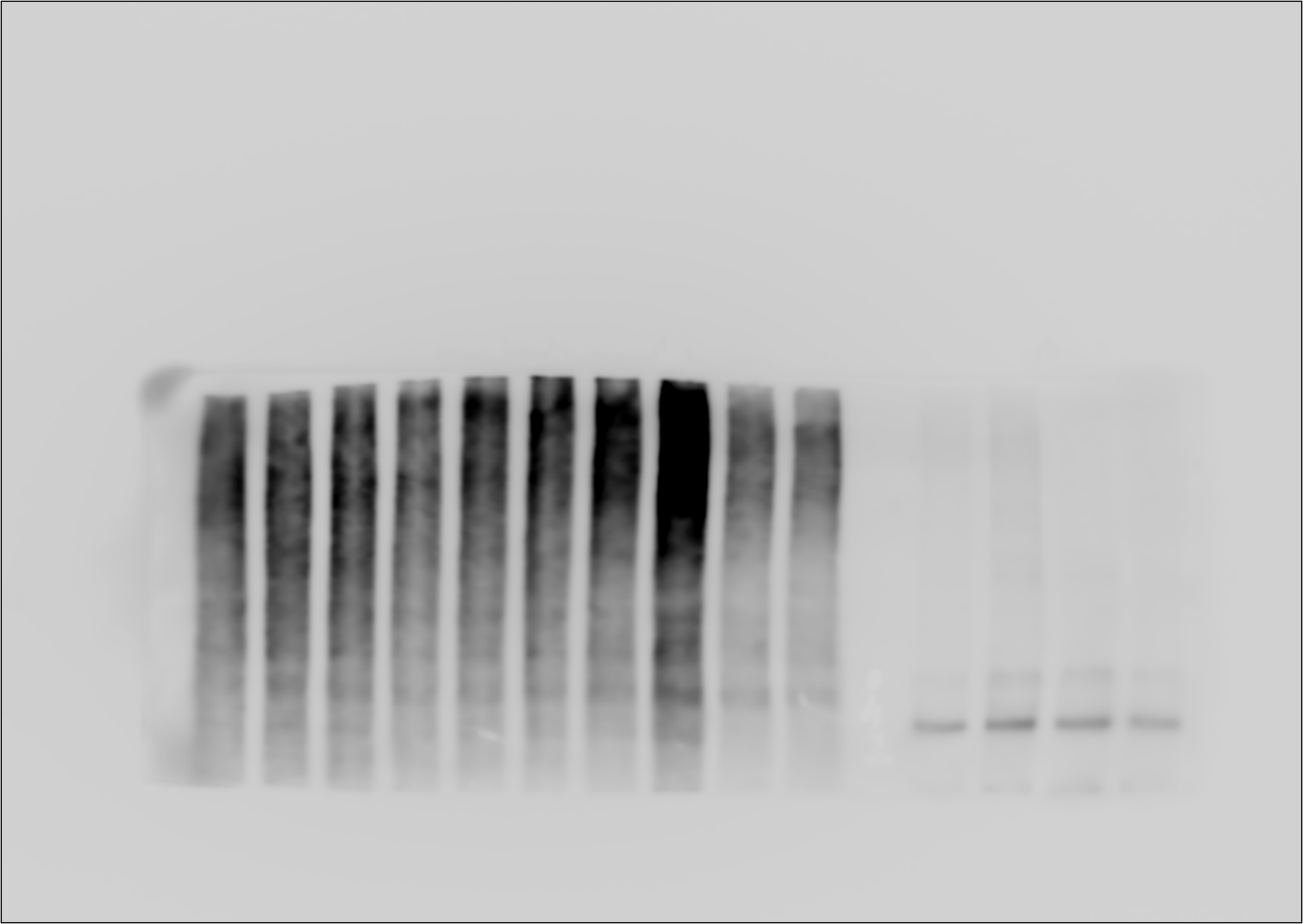

Supplement: Figure 9—source data 2. [file elife-108048-fig9-data2.zip › Figure 9/Figure 9 M-WCL-HA.tif]

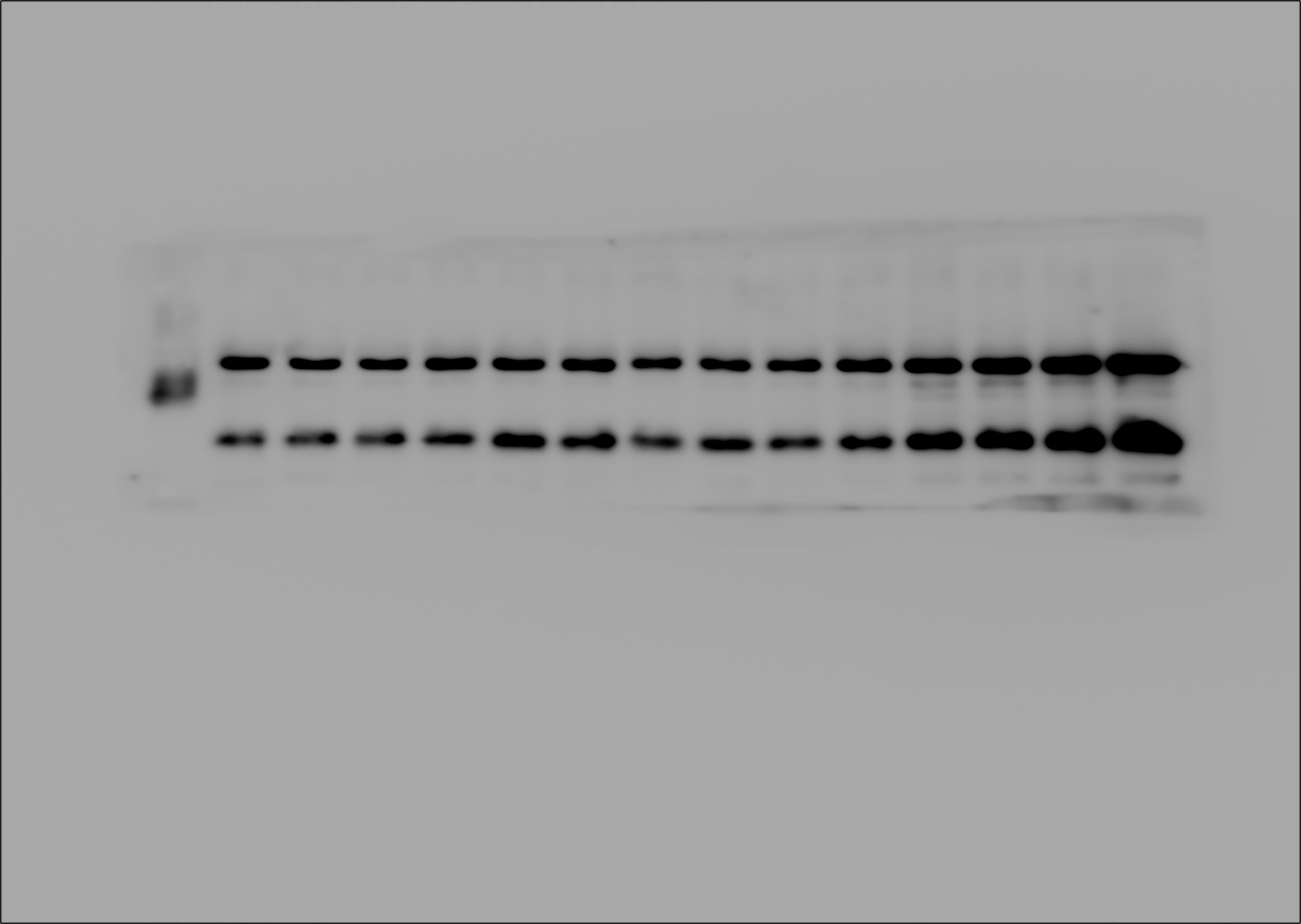

Supplement: Figure 9—source data 2. [file elife-108048-fig9-data2.zip › Figure 9/Figure 9 M-WCL-Myc.tif]

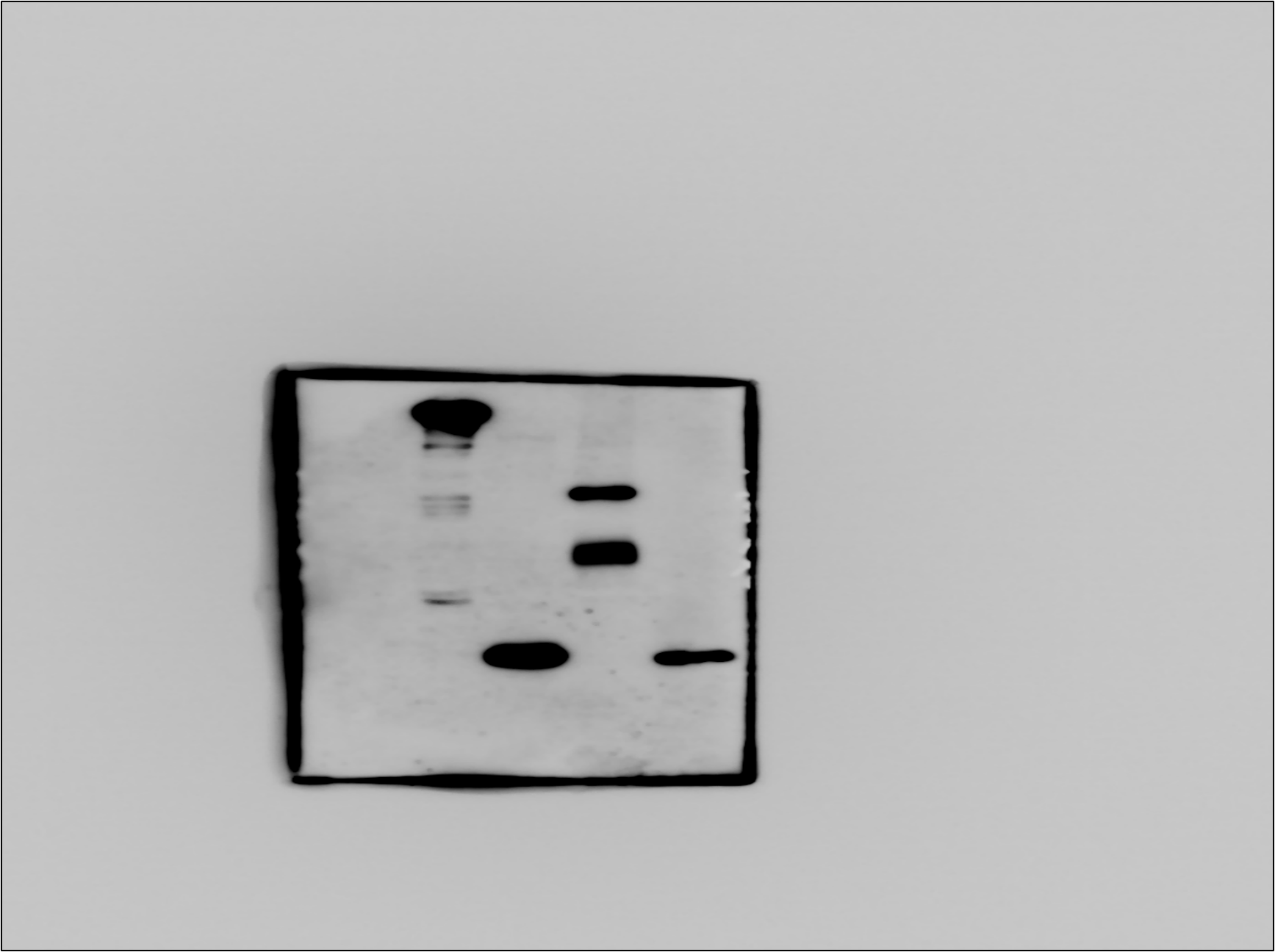

Supplement: Figure 9—source data 2. [file elife-108048-fig9-data2.zip › Figure 9/Figure 9 O-IP-HA-1.tif]

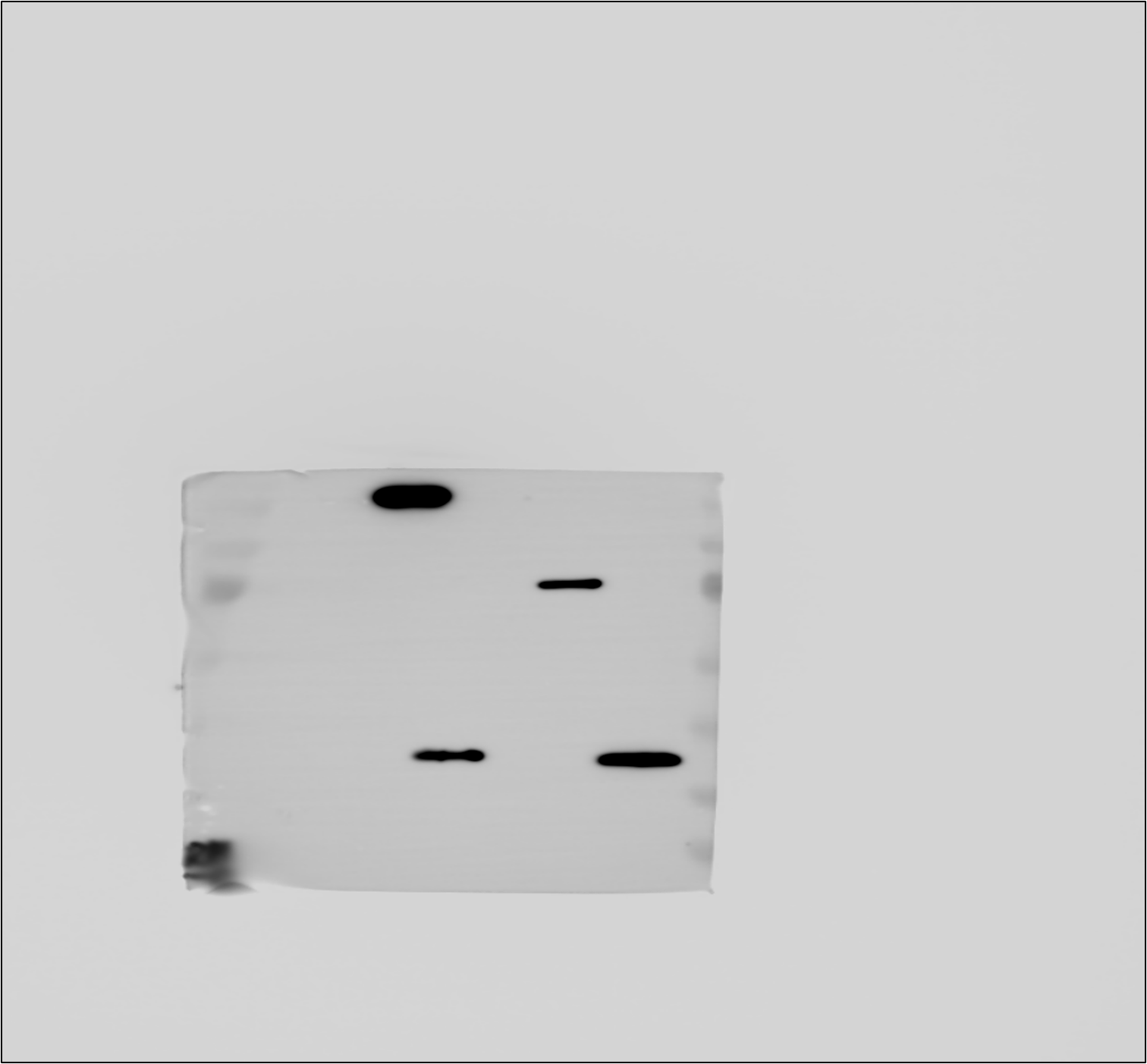

Supplement: Figure 9—source data 2. [file elife-108048-fig9-data2.zip › Figure 9/Figure 9 O-IP-HA-2.tif]

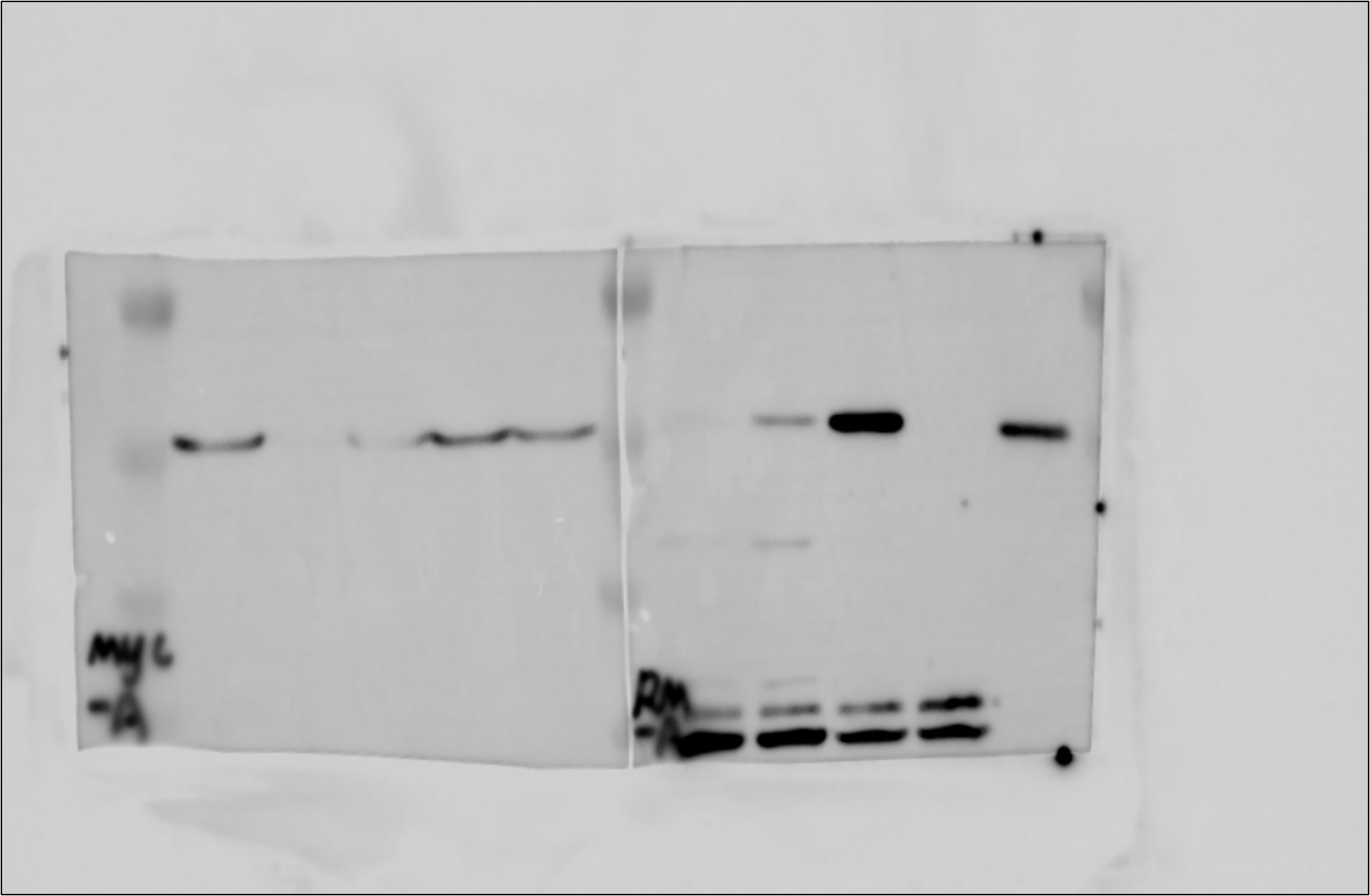

Supplement: Figure 9—source data 2. [file elife-108048-fig9-data2.zip › Figure 9/Figure 9 O-IP-Myc-cyp17a2.tif]

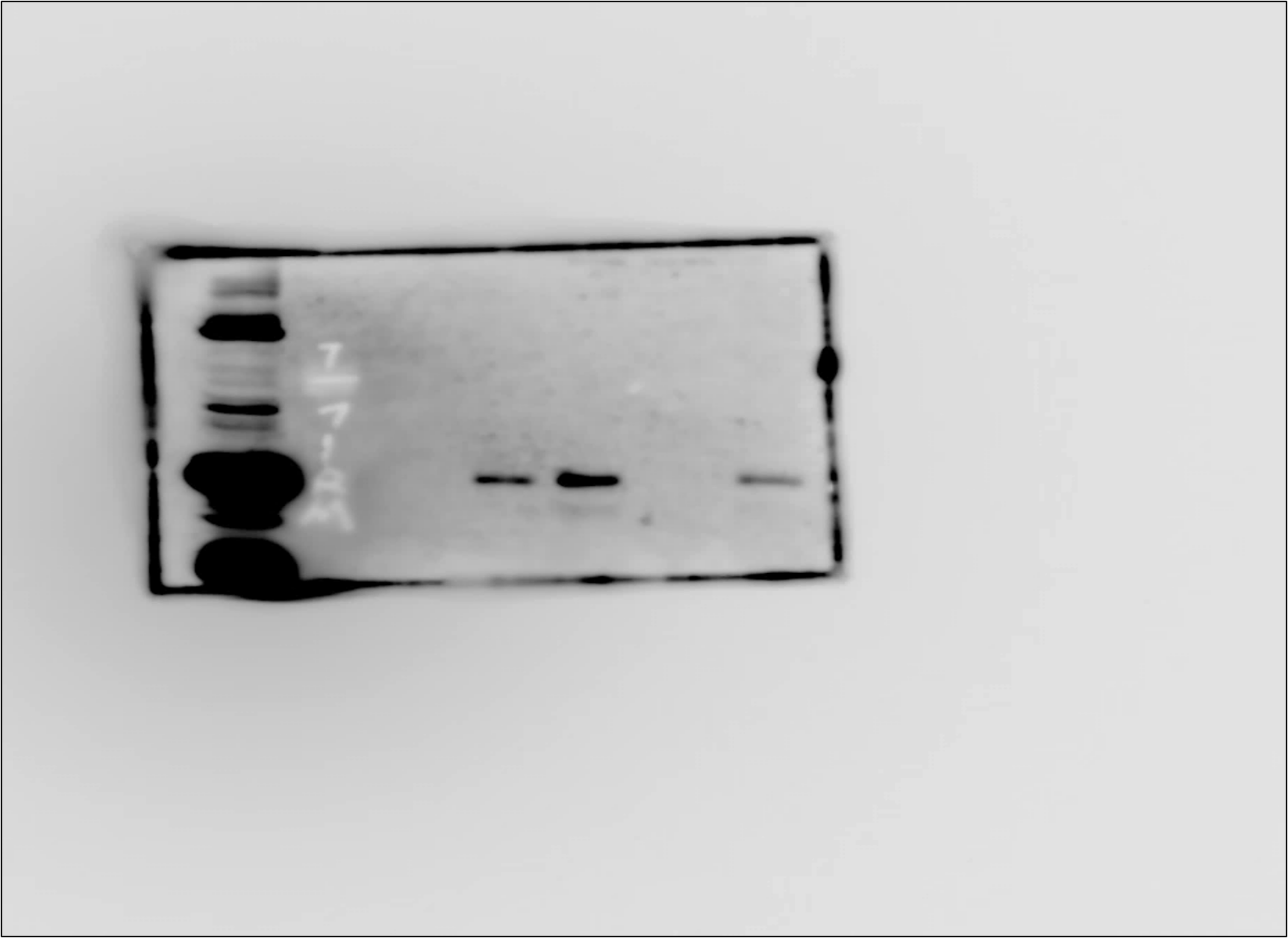

Supplement: Figure 9—source data 2. [file elife-108048-fig9-data2.zip › Figure 9/Figure 9 O-IP-Myc-P.tif]

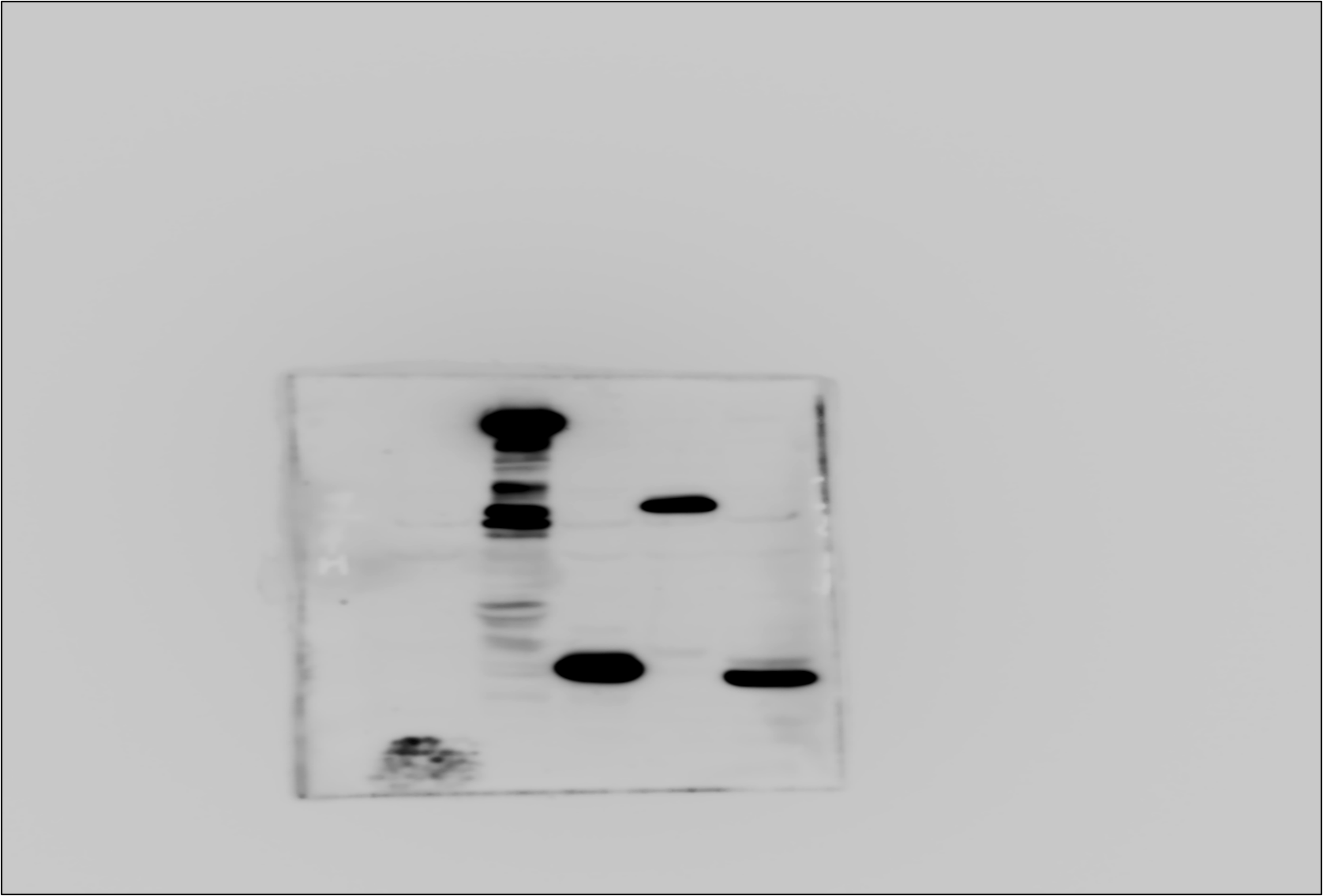

Supplement: Figure 9—source data 2. [file elife-108048-fig9-data2.zip › Figure 9/Figure 9 O-WCL-HA-1.tif]

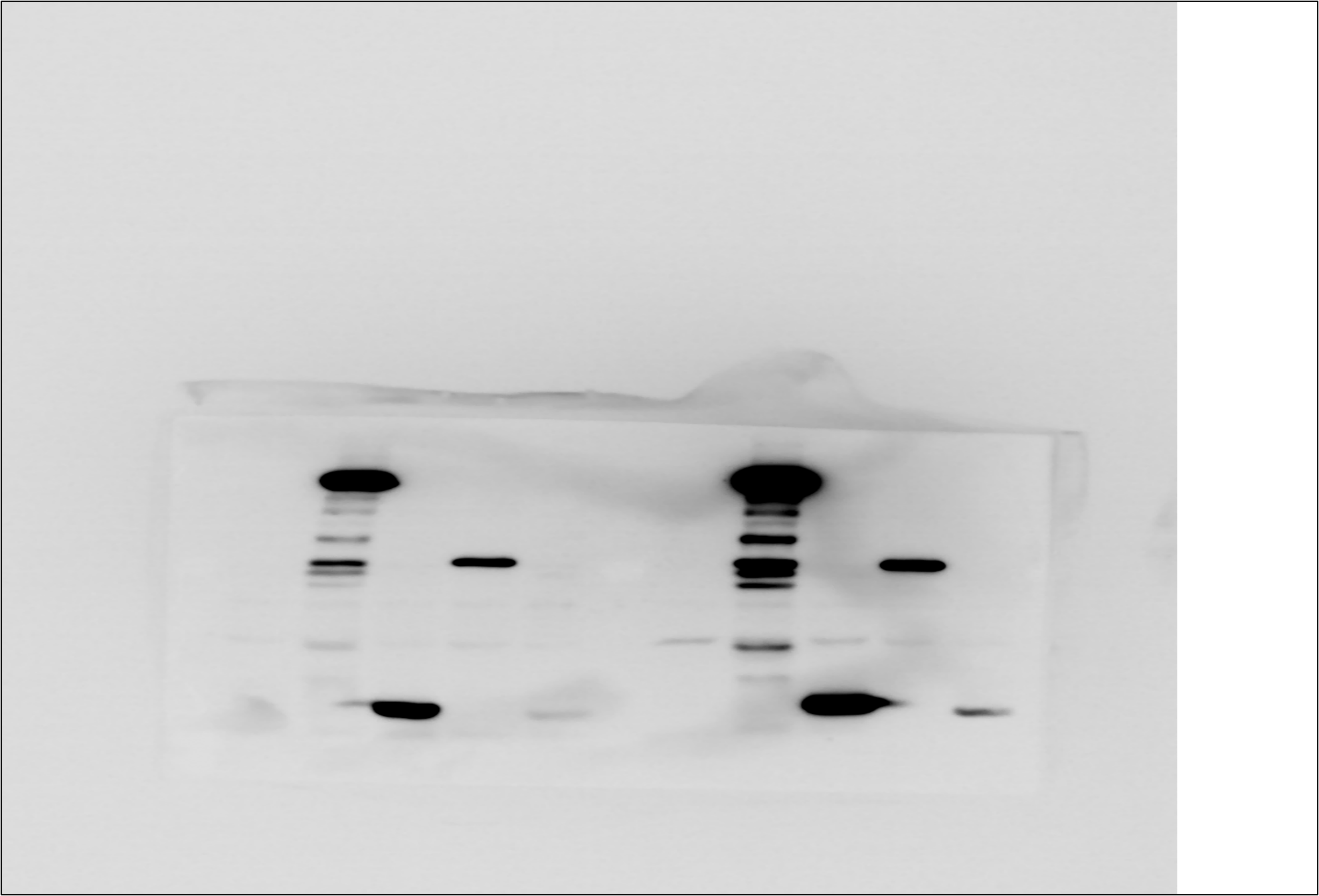

Supplement: Figure 9—source data 2. [file elife-108048-fig9-data2.zip › Figure 9/Figure 9 O-WCL-HA-2.tif]

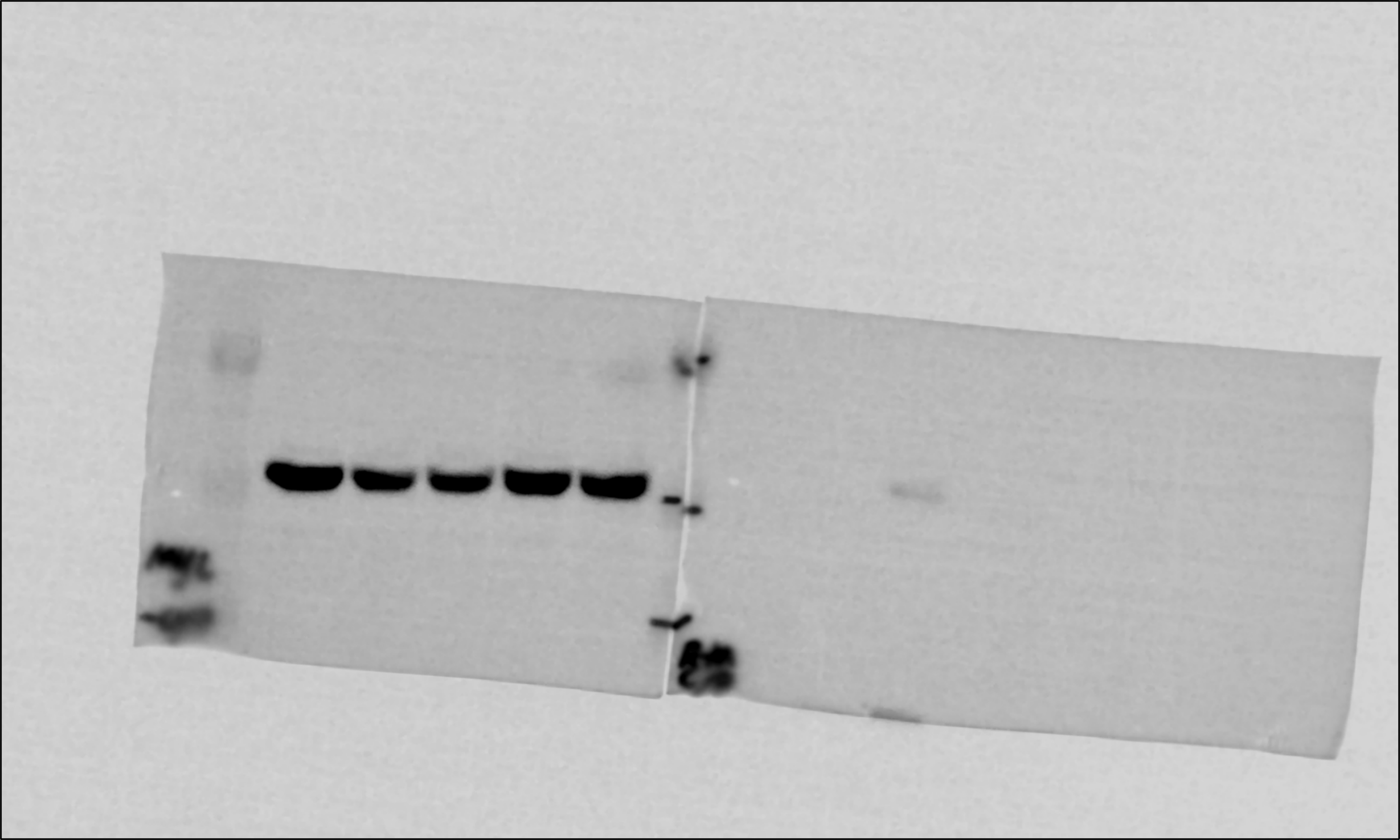

Supplement: Figure 9—source data 2. [file elife-108048-fig9-data2.zip › Figure 9/Figure 9 O-WCL-Myc-cyp17a2.tif]

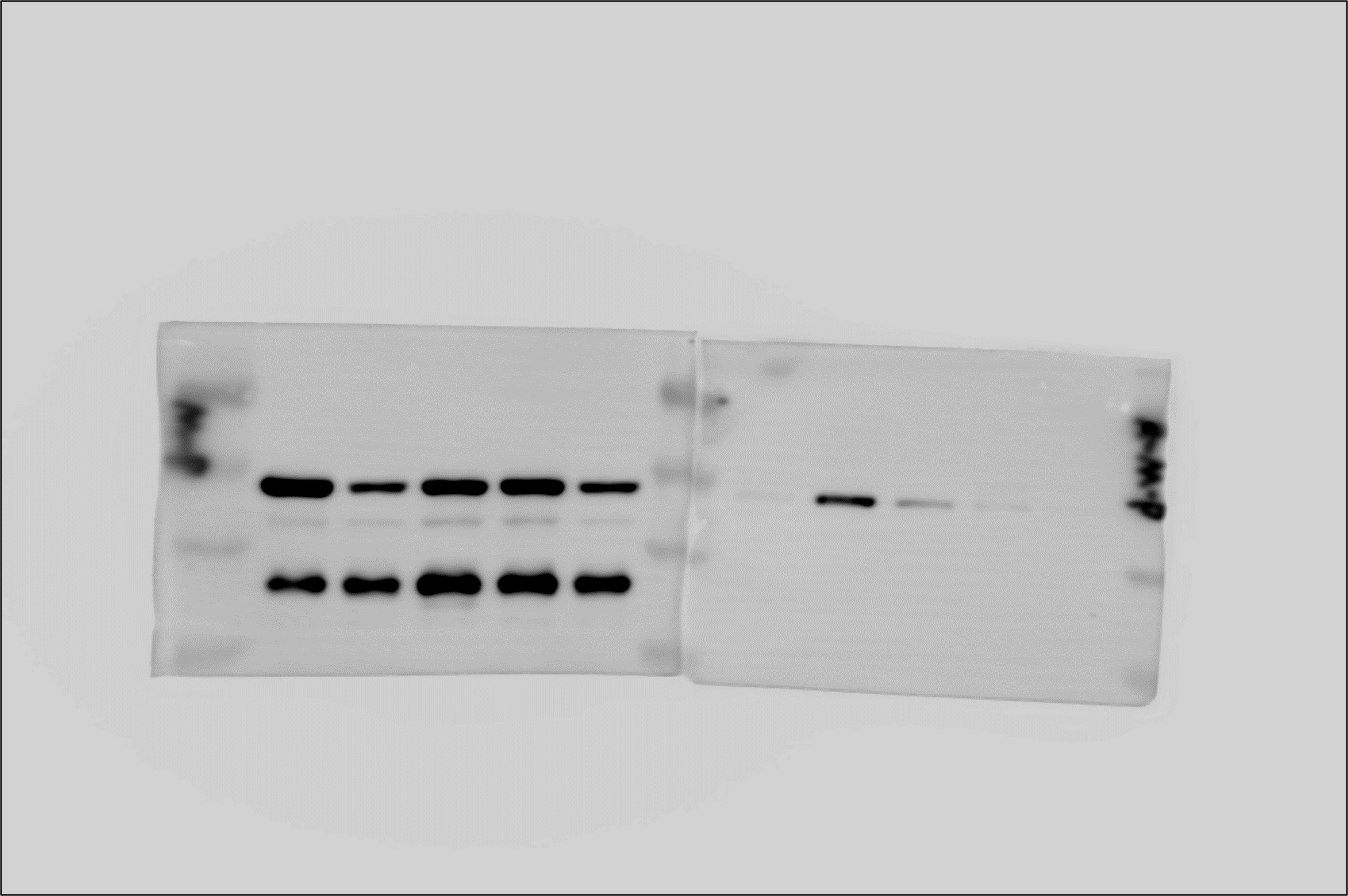

Supplement: Figure 9—source data 2. [file elife-108048-fig9-data2.zip › Figure 9/Figure 9 O-WCL-Myc-P.tif]

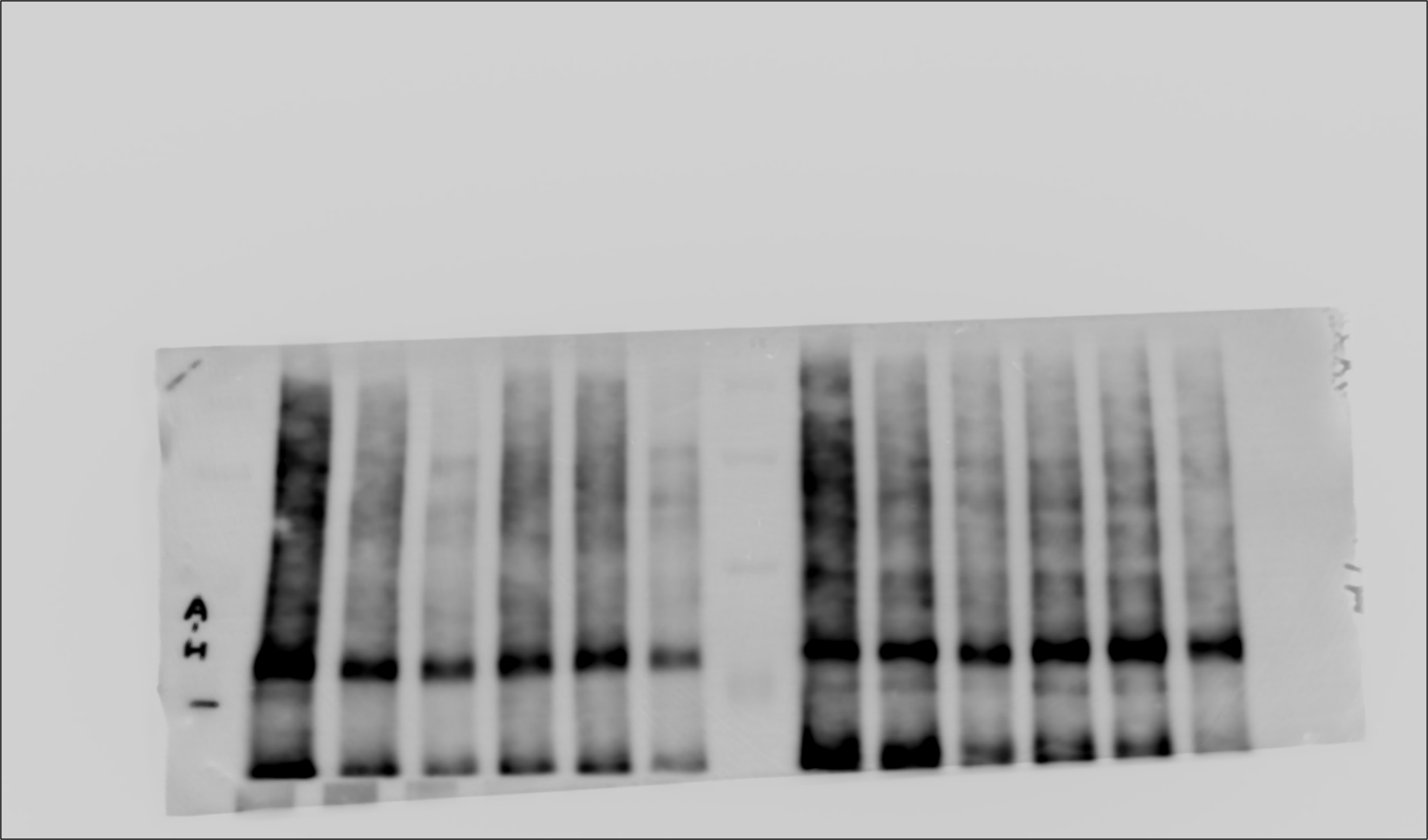

Supplement: Figure 9—source data 2. [file elife-108048-fig9-data2.zip › Figure 9/Figure 9 P-IP-HA-.tif]

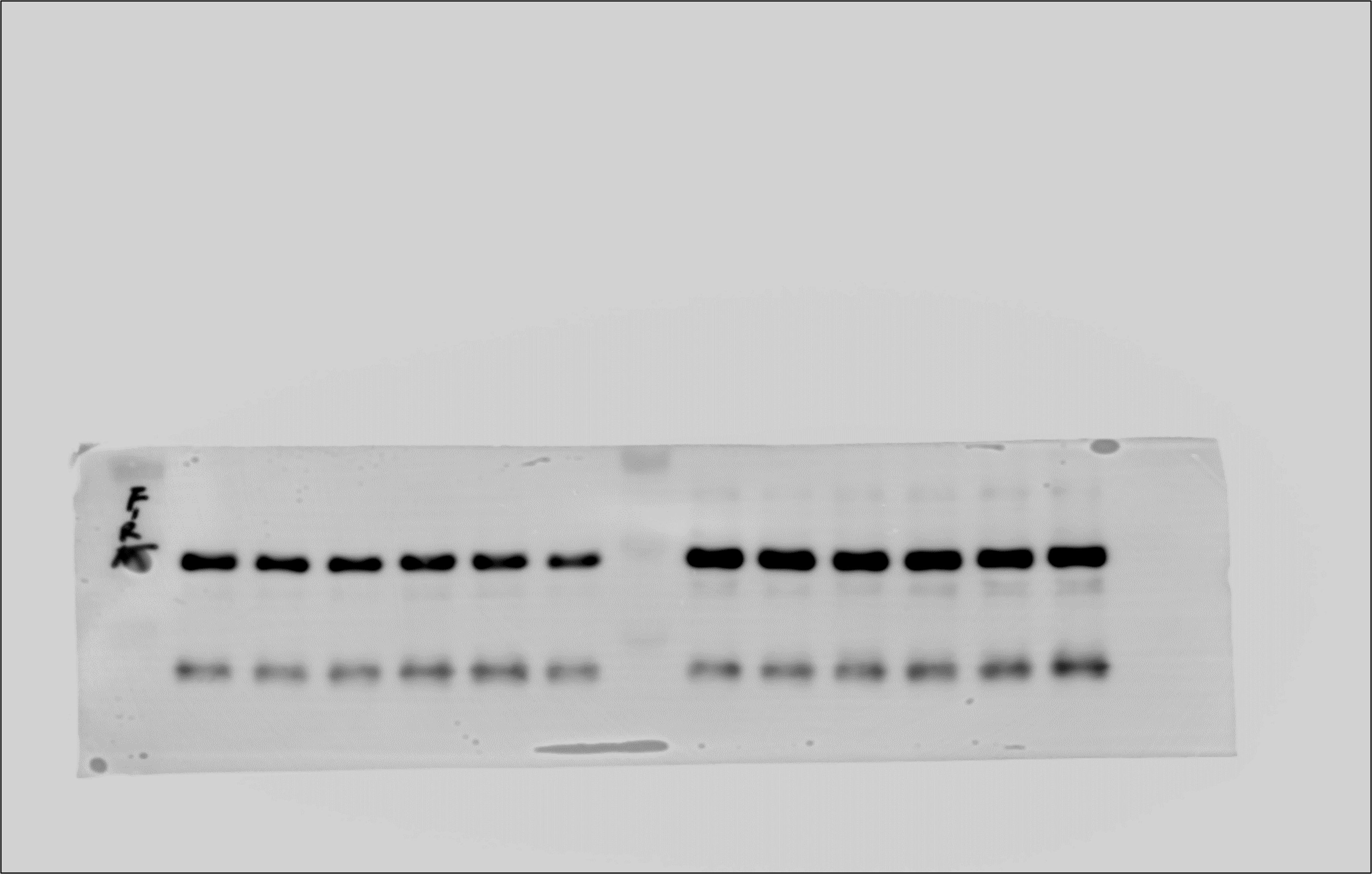

Supplement: Figure 9—source data 2. [file elife-108048-fig9-data2.zip › Figure 9/Figure 9 P-IP-Myc.tif]

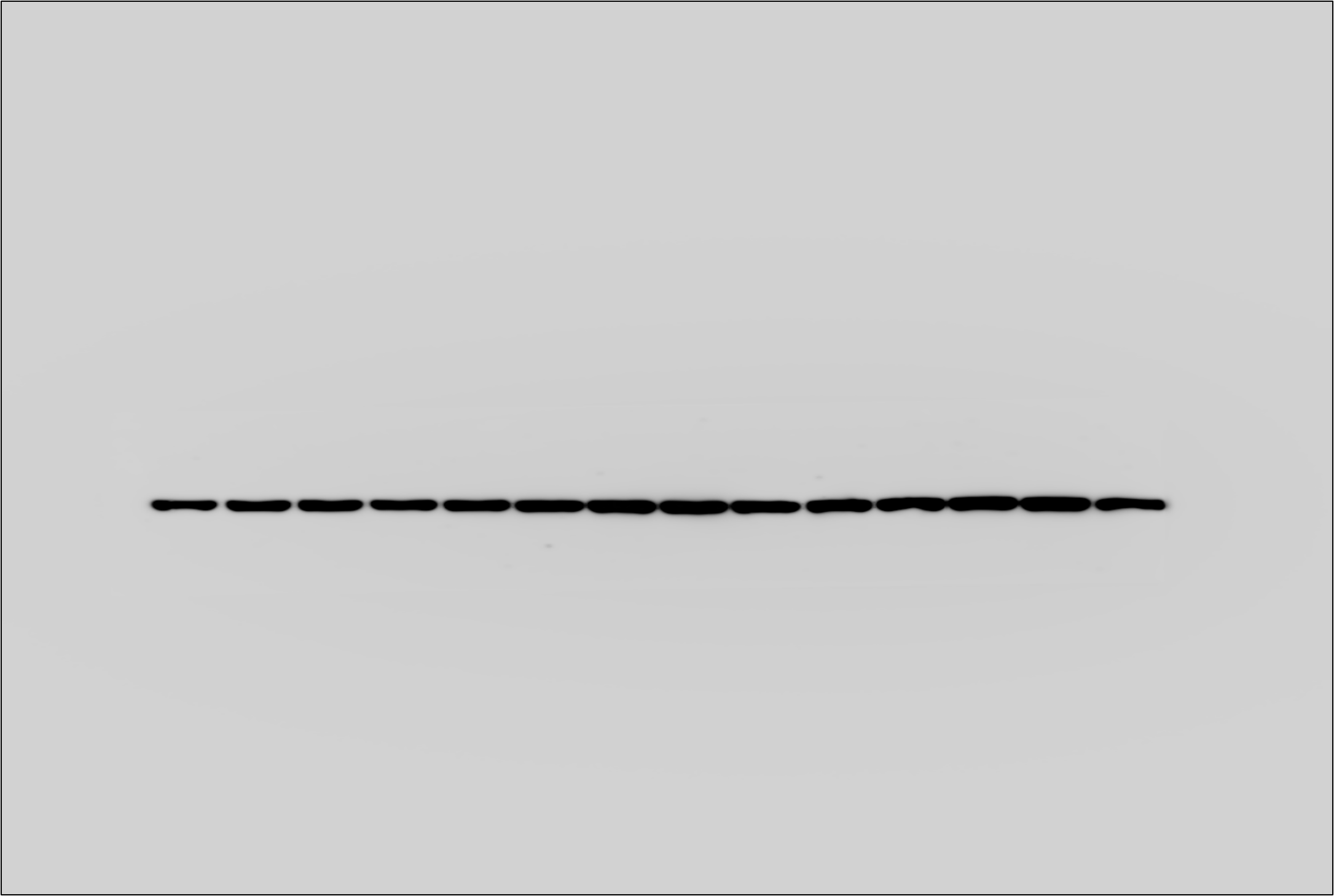

Supplement: Figure 9—source data 2. [file elife-108048-fig9-data2.zip › Figure 9/Figure 9 P-WCL-Actin.tif]

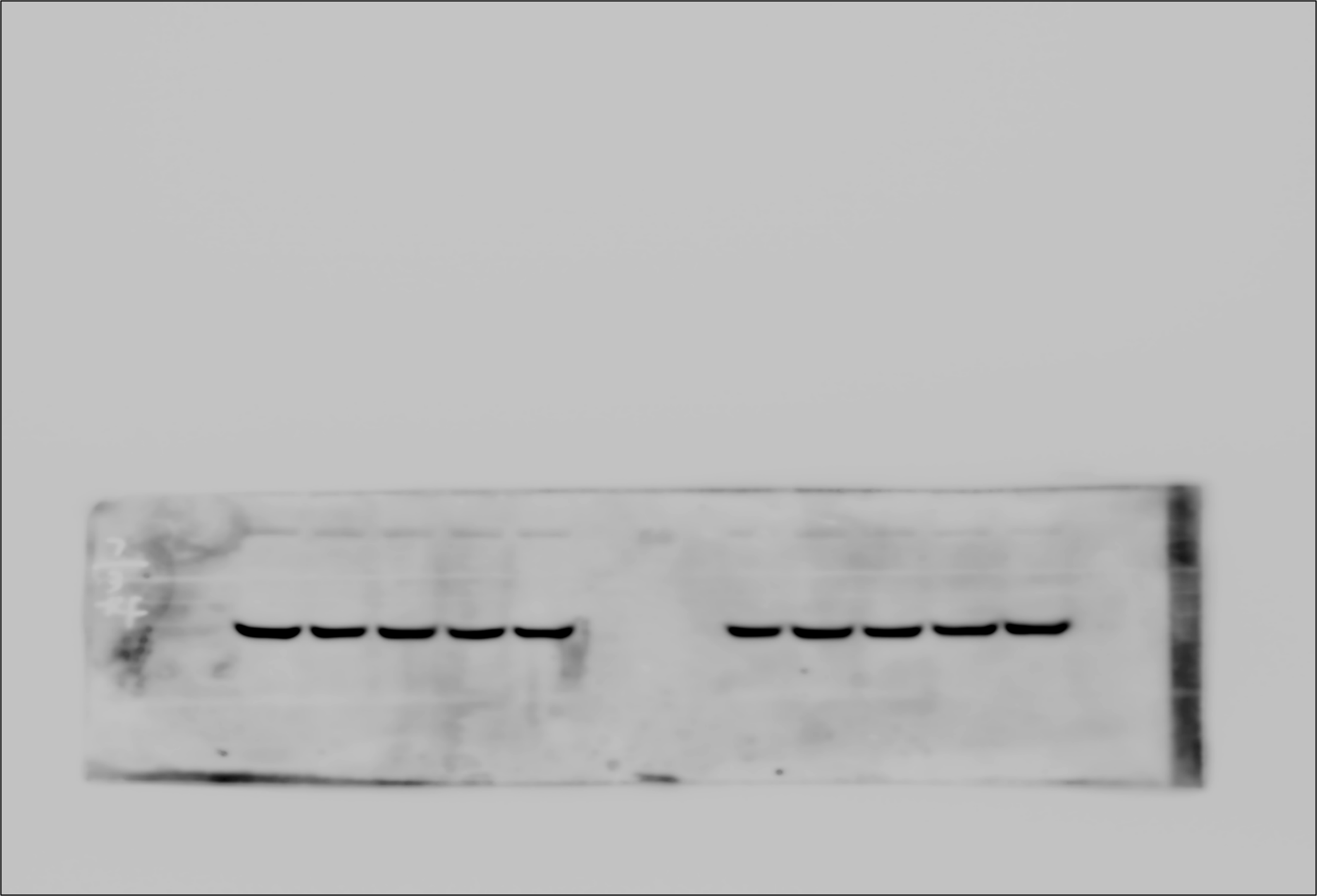

Supplement: Figure 9—source data 2. [file elife-108048-fig9-data2.zip › Figure 9/Figure 9 P-WCL-Flag.tif]

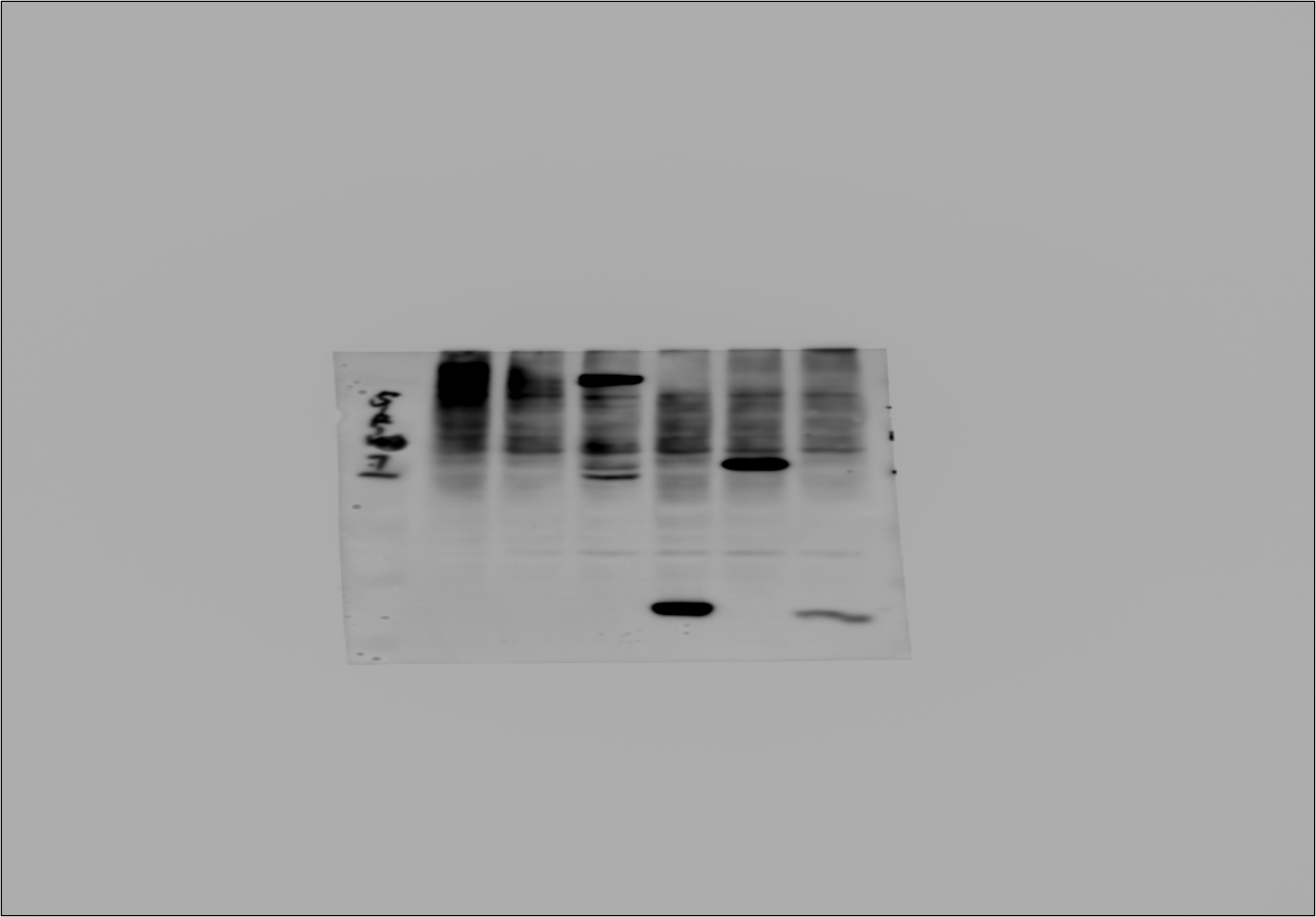

Supplement: Figure 9—source data 2. [file elife-108048-fig9-data2.zip › Figure 9/Figure 9 P-WCL-HA.tif]

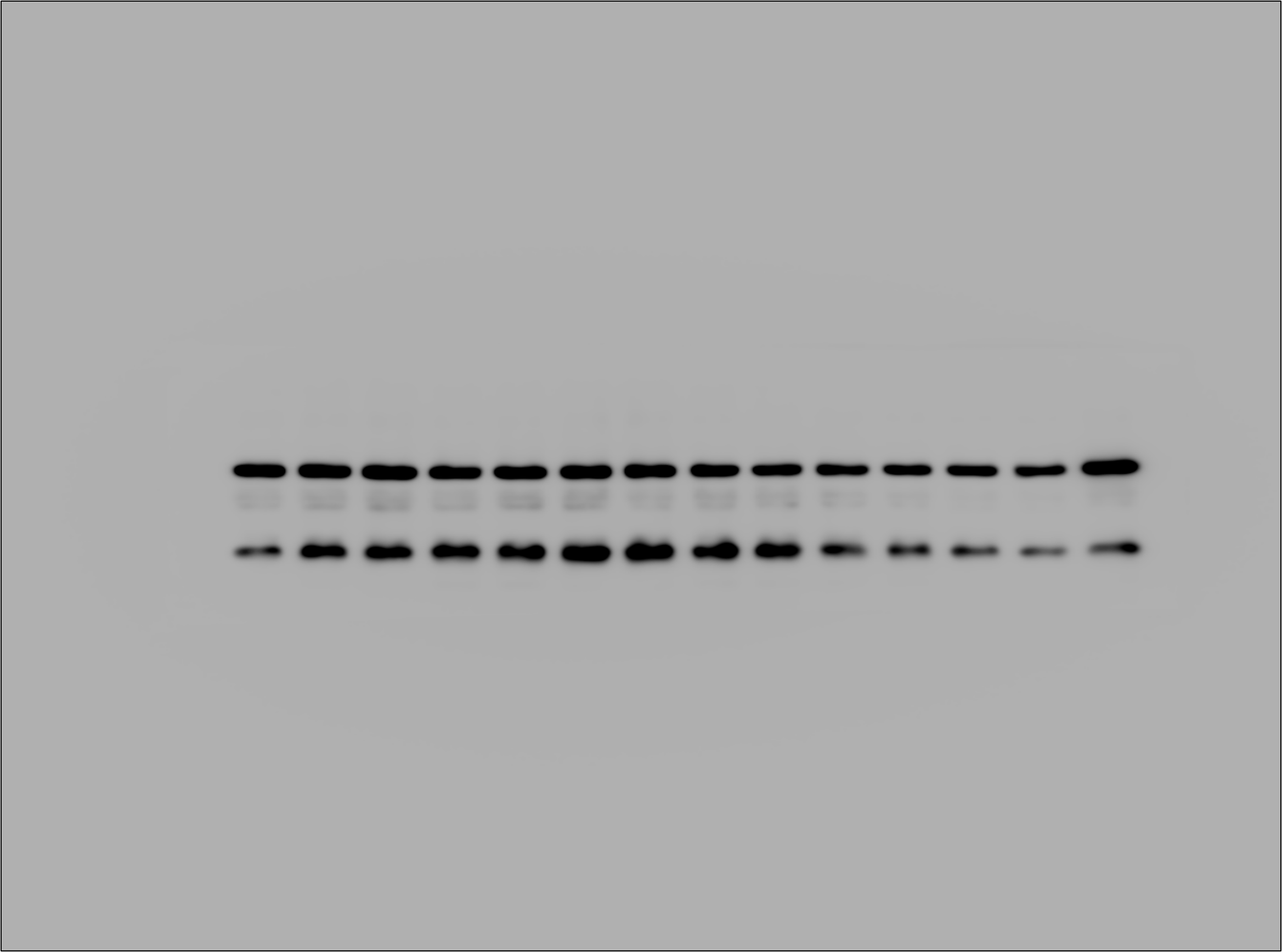

Supplement: Figure 9—source data 2. [file elife-108048-fig9-data2.zip › Figure 9/Figure 9 P-WCL-Myc.tif]

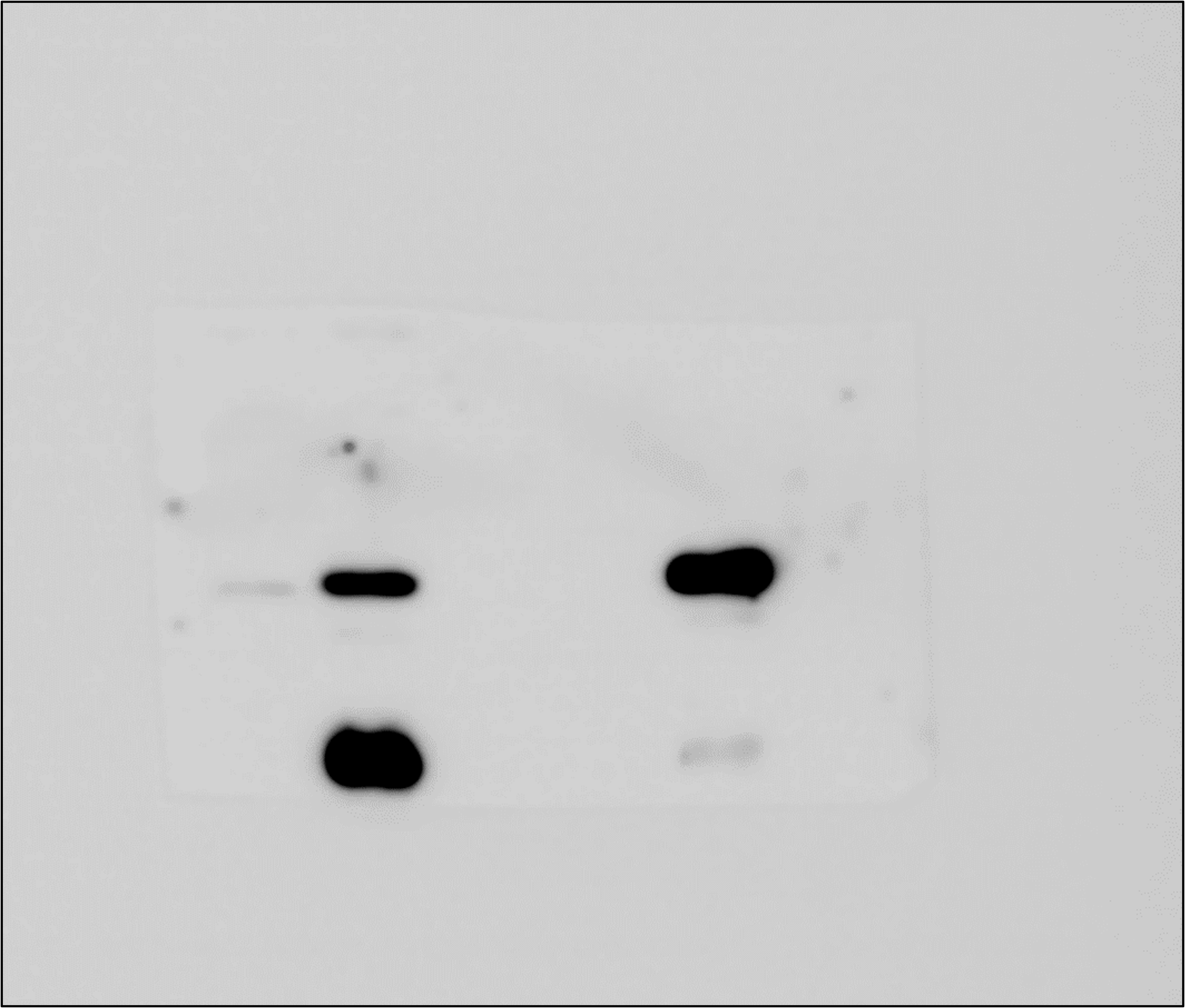

Supplement: Figure 9—figure supplement 1—source data 2. [file elife-108048-fig9-figsupp1-data2.zip › Figure 9-figure supplement 1/Figure S8 A-IP-Flag-2.tif]

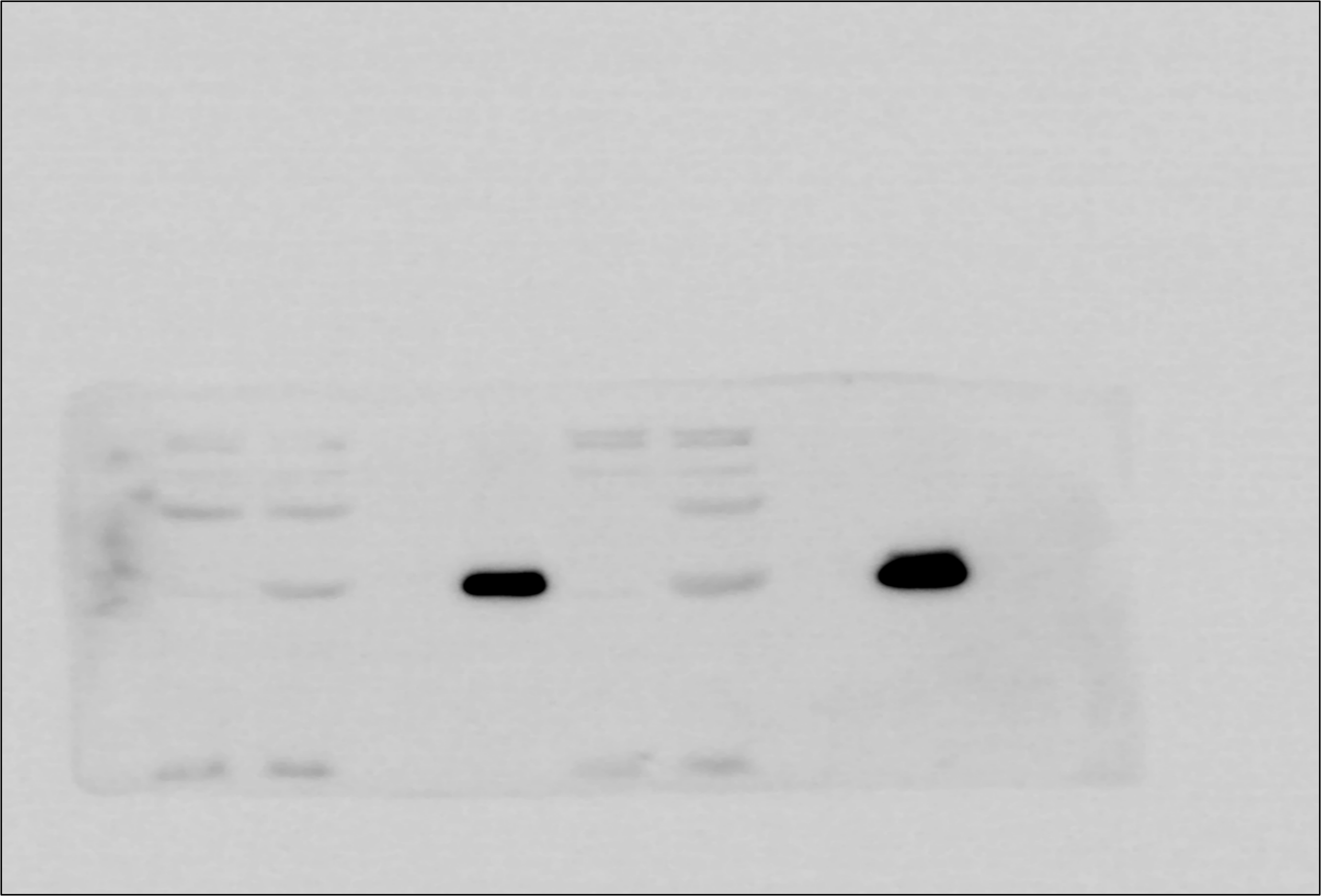

Supplement: Figure 9—figure supplement 1—source data 2. [file elife-108048-fig9-figsupp1-data2.zip › Figure 9-figure supplement 1/Figure S8 A-IP-Flag.tif]

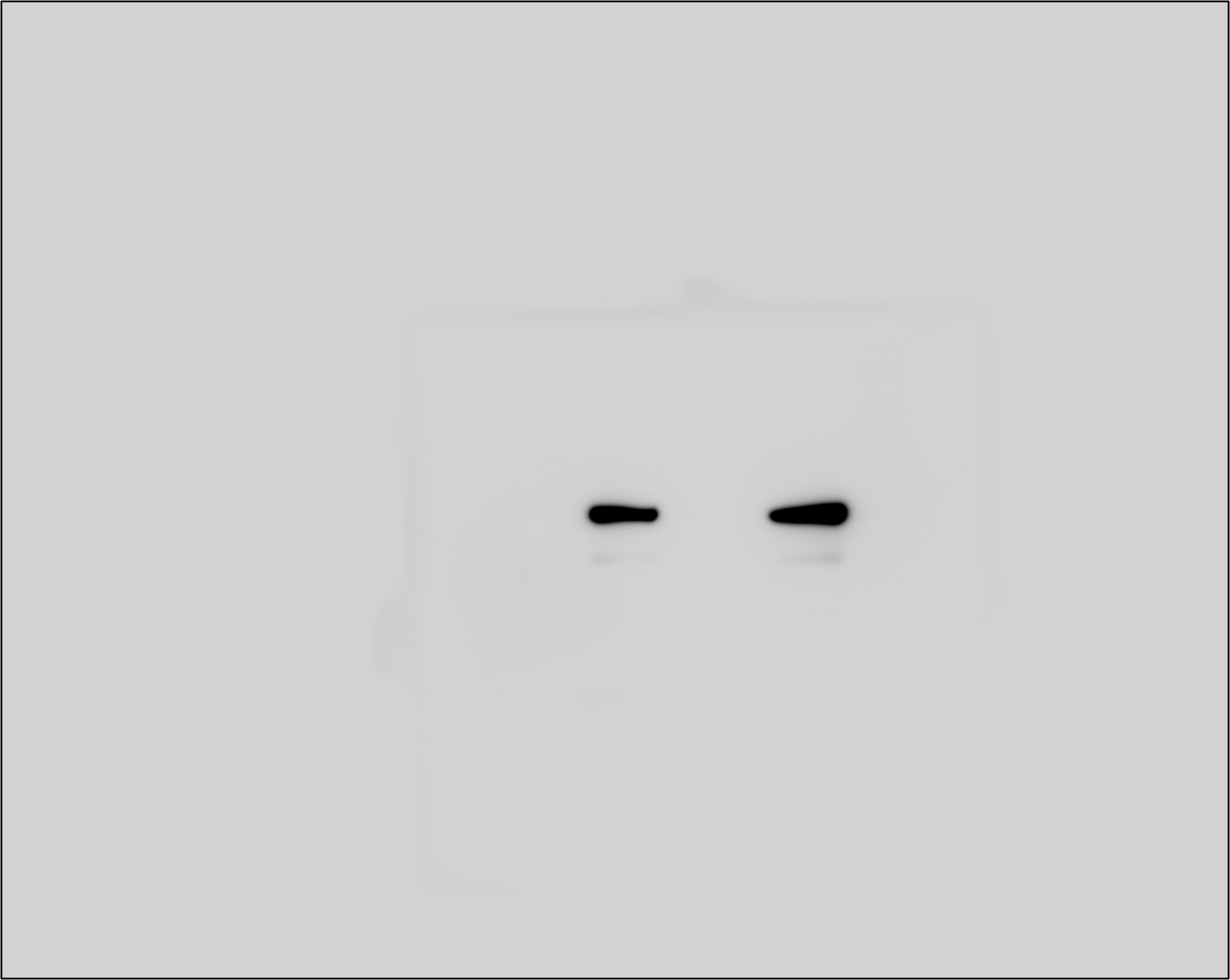

Supplement: Figure 9—figure supplement 1—source data 2. [file elife-108048-fig9-figsupp1-data2.zip › Figure 9-figure supplement 1/Figure S8 A-IP-HA.tif]

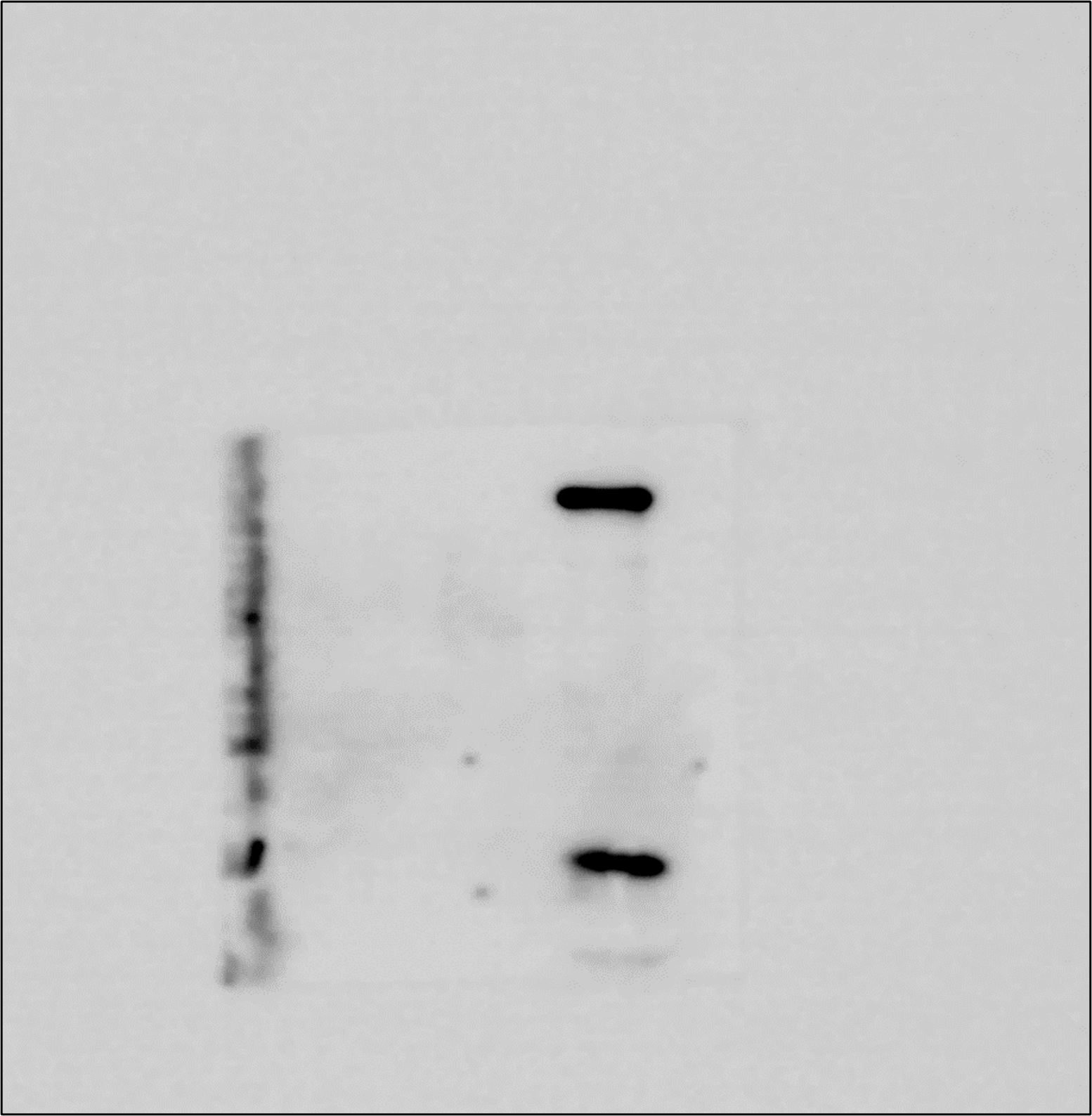

Supplement: Figure 9—figure supplement 1—source data 2. [file elife-108048-fig9-figsupp1-data2.zip › Figure 9-figure supplement 1/Figure S8 A-IP-Myc.tif]

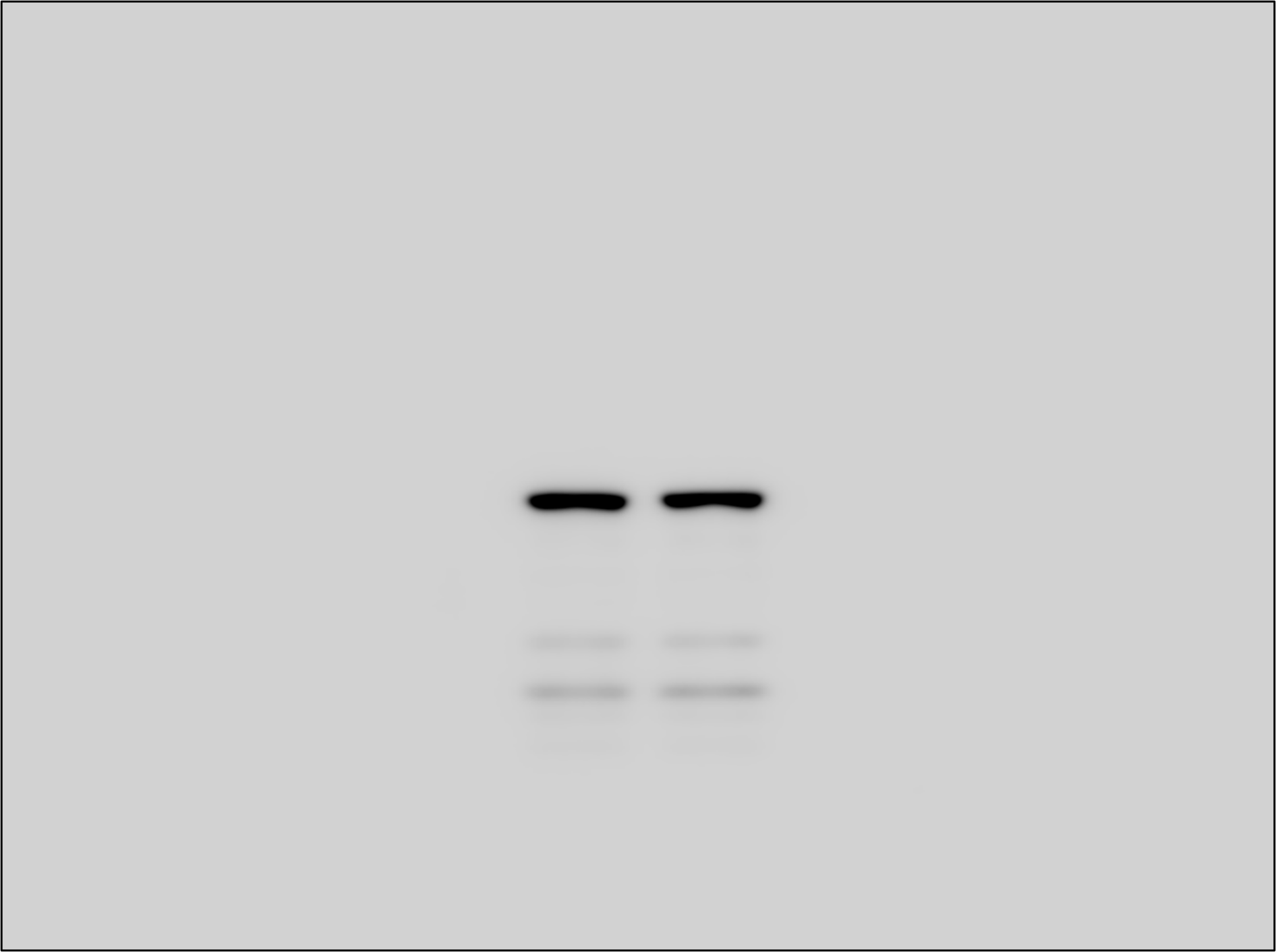

Supplement: Figure 9—figure supplement 1—source data 2. [file elife-108048-fig9-figsupp1-data2.zip › Figure 9-figure supplement 1/Figure S8 A-WCL-Flag-2.tif]

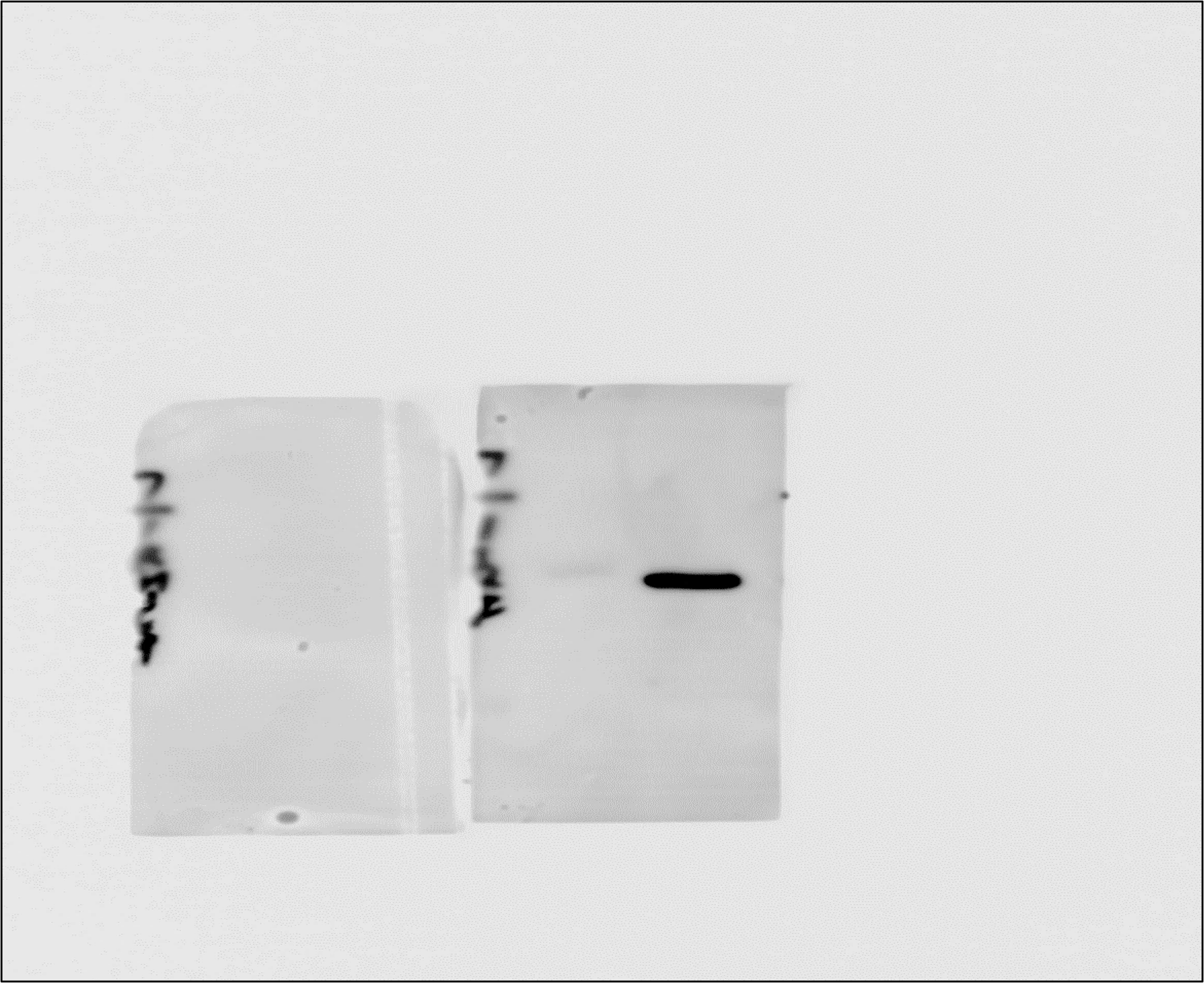

Supplement: Figure 9—figure supplement 1—source data 2. [file elife-108048-fig9-figsupp1-data2.zip › Figure 9-figure supplement 1/Figure S8 A-WCL-Flag.tif]

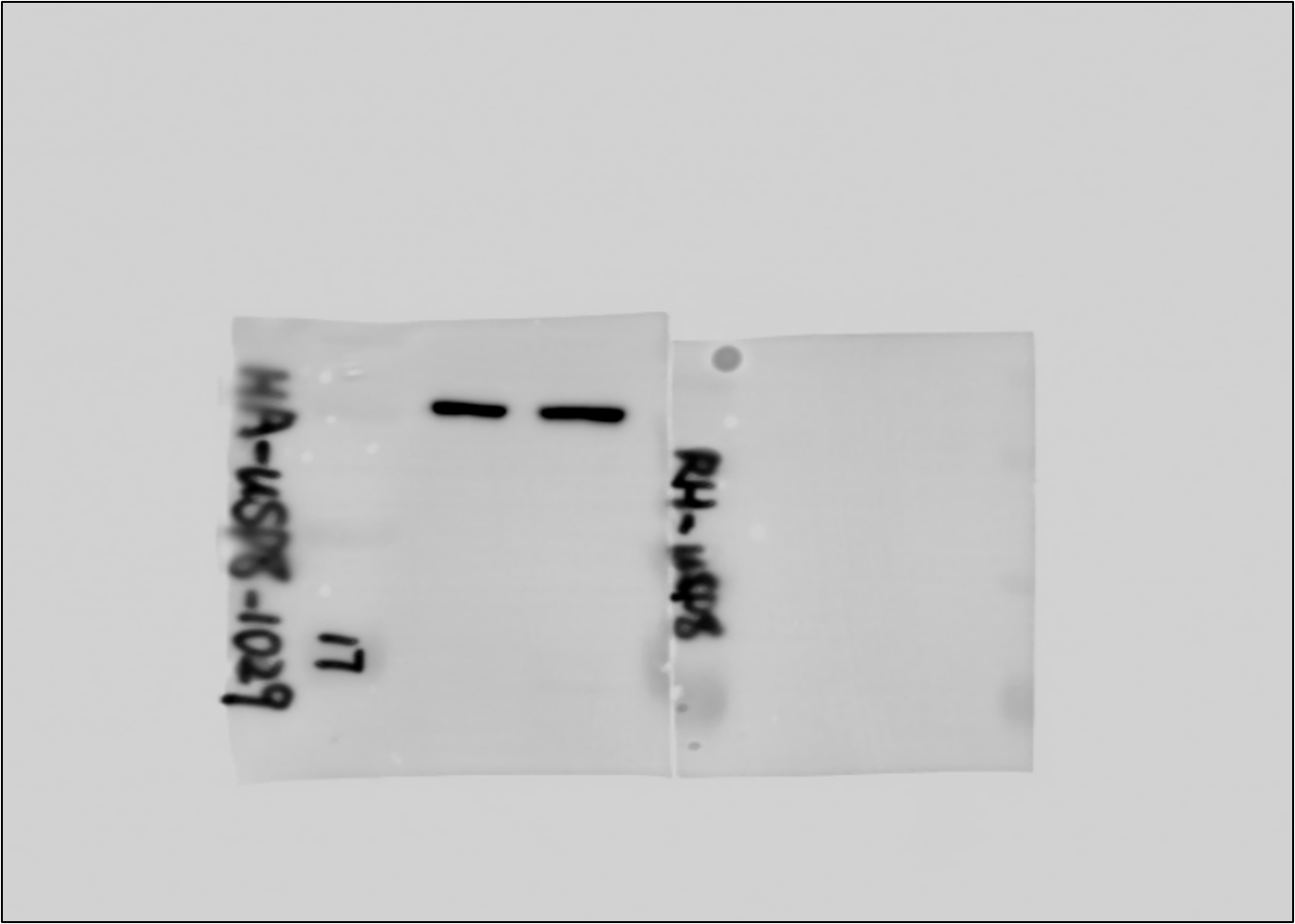

Supplement: Figure 9—figure supplement 1—source data 2. [file elife-108048-fig9-figsupp1-data2.zip › Figure 9-figure supplement 1/Figure S8 A-WCL-HA.tif]

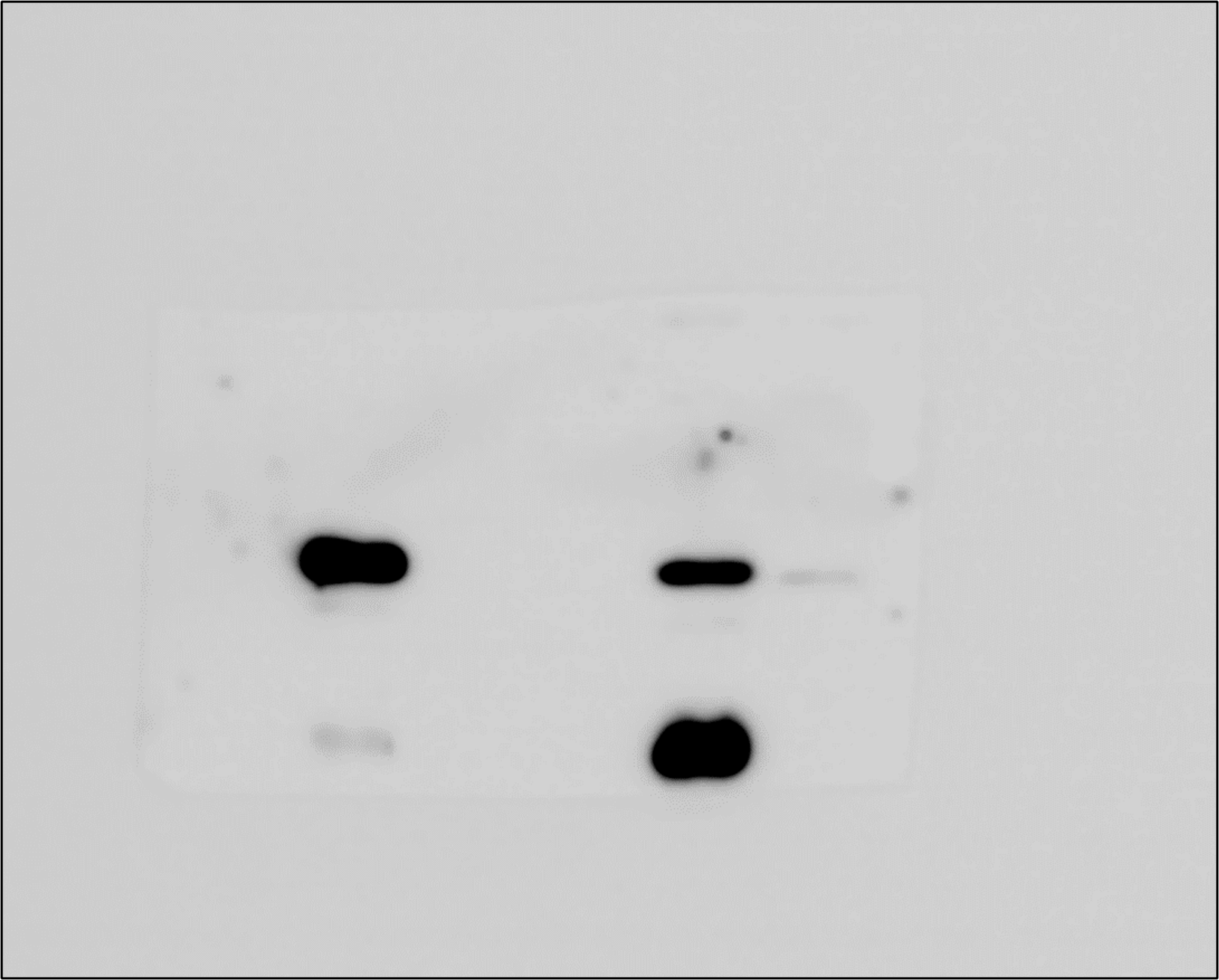

Supplement: Figure 9—figure supplement 1—source data 2. [file elife-108048-fig9-figsupp1-data2.zip › Figure 9-figure supplement 1/Figure S8 A-WCL-Myc.tif]

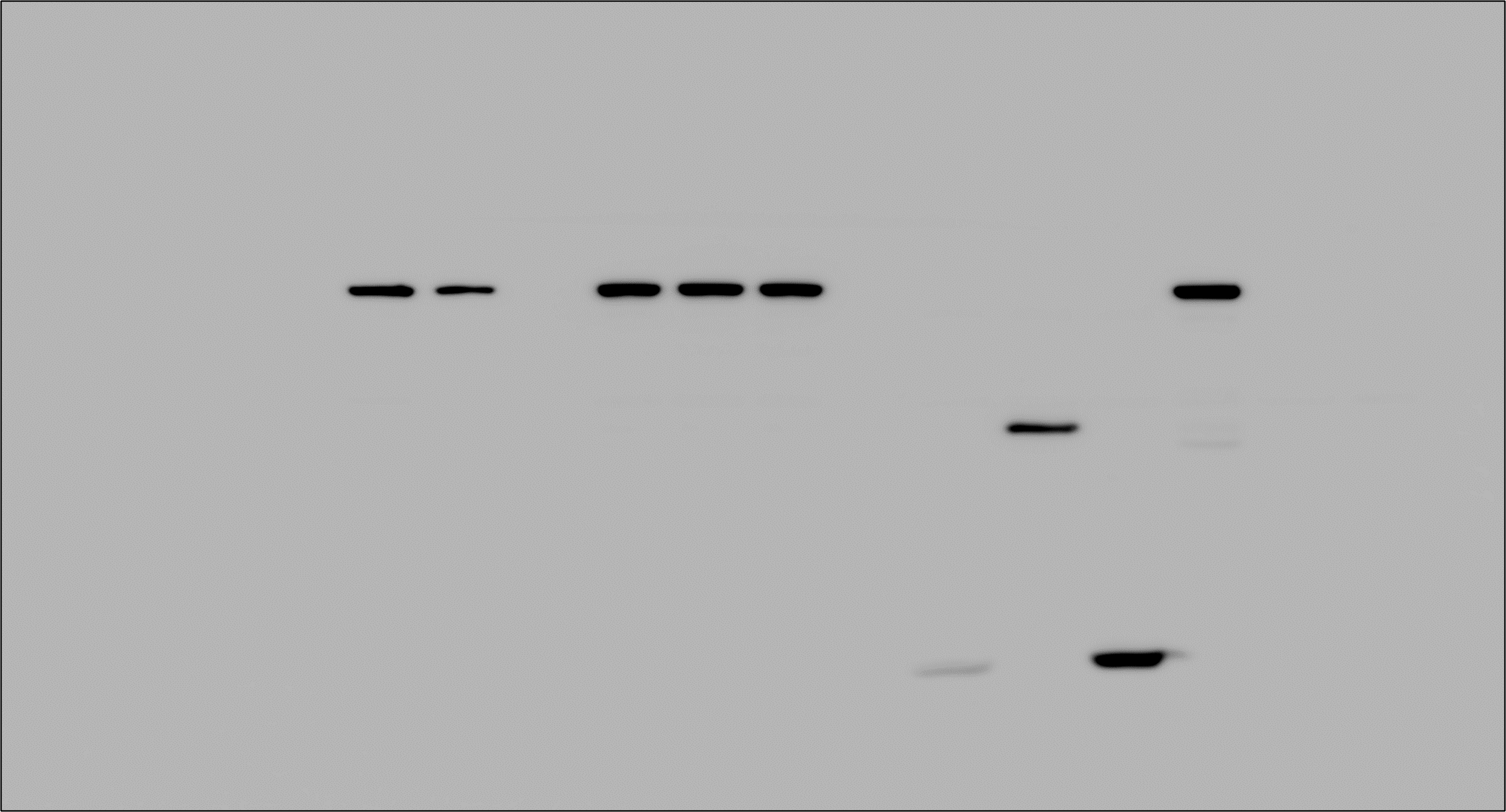

Supplement: Figure 9—figure supplement 1—source data 2. [file elife-108048-fig9-figsupp1-data2.zip › Figure 9-figure supplement 1/Figure S8 B-IP-HA.tif]

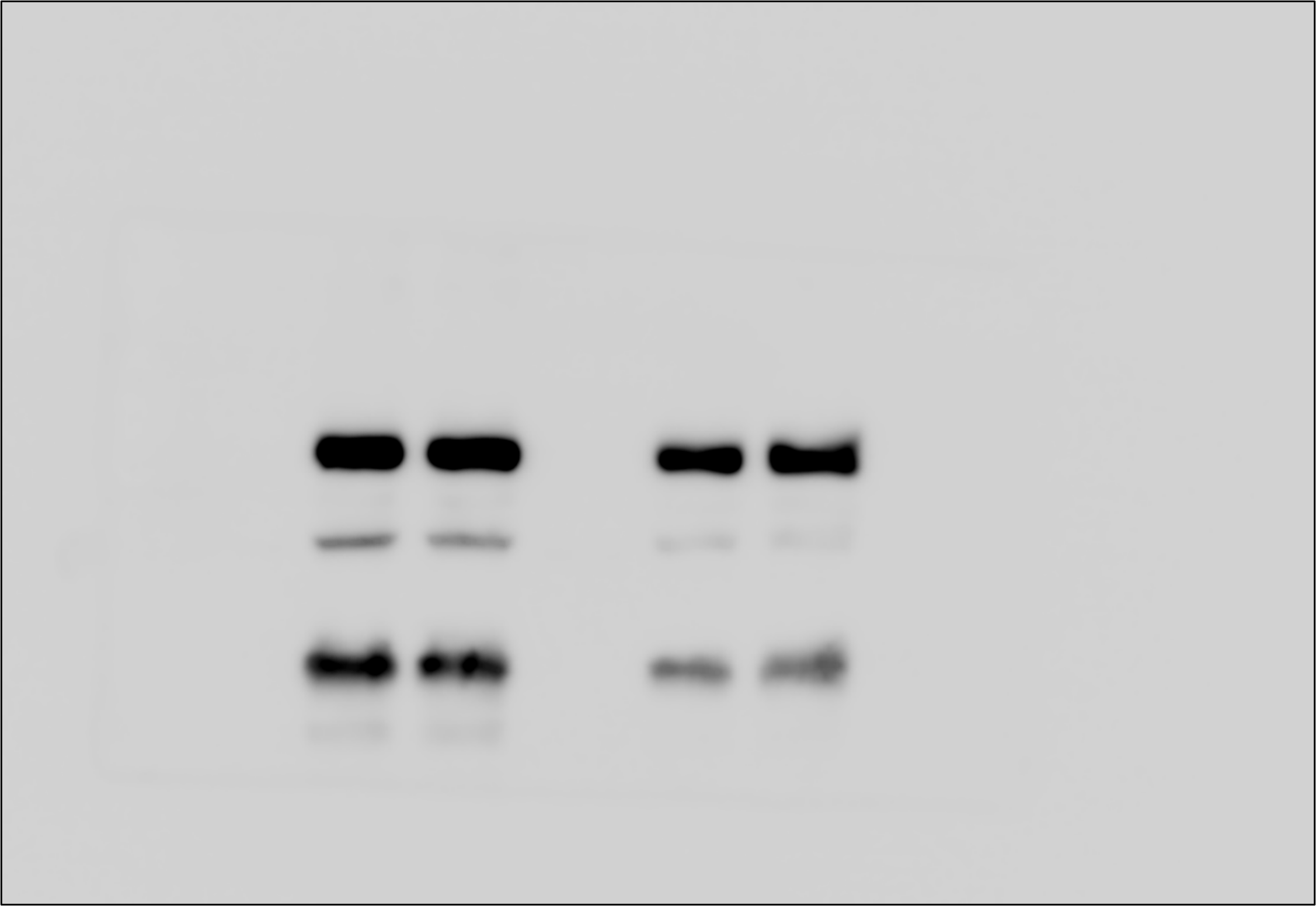

Supplement: Figure 9—figure supplement 1—source data 2. [file elife-108048-fig9-figsupp1-data2.zip › Figure 9-figure supplement 1/Figure S8 B-IP-Myc.tif]

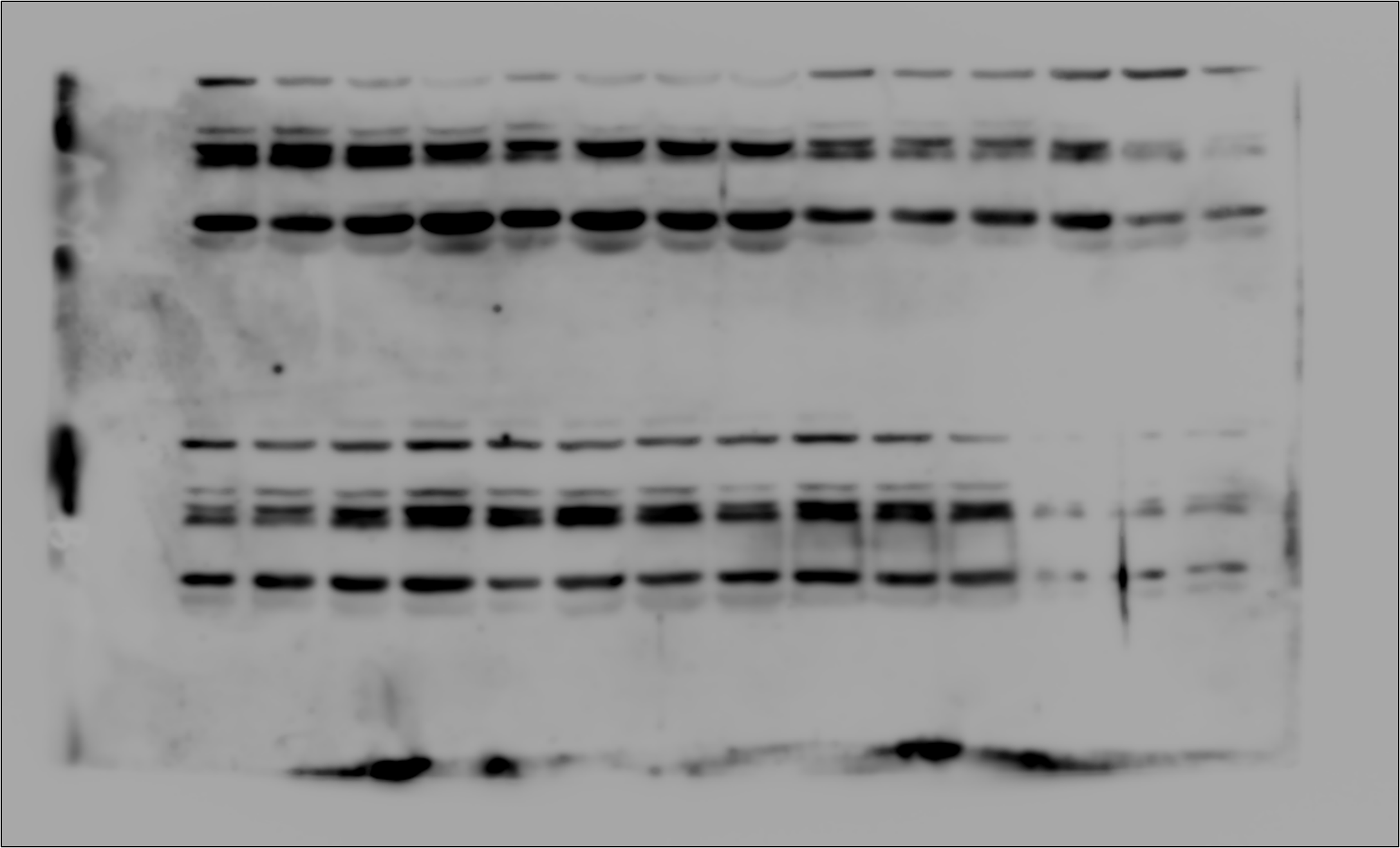

Supplement: Figure 9—figure supplement 1—source data 2. [file elife-108048-fig9-figsupp1-data2.zip › Figure 9-figure supplement 1/Figure S8 B-WCL-cyp17a2.tif]

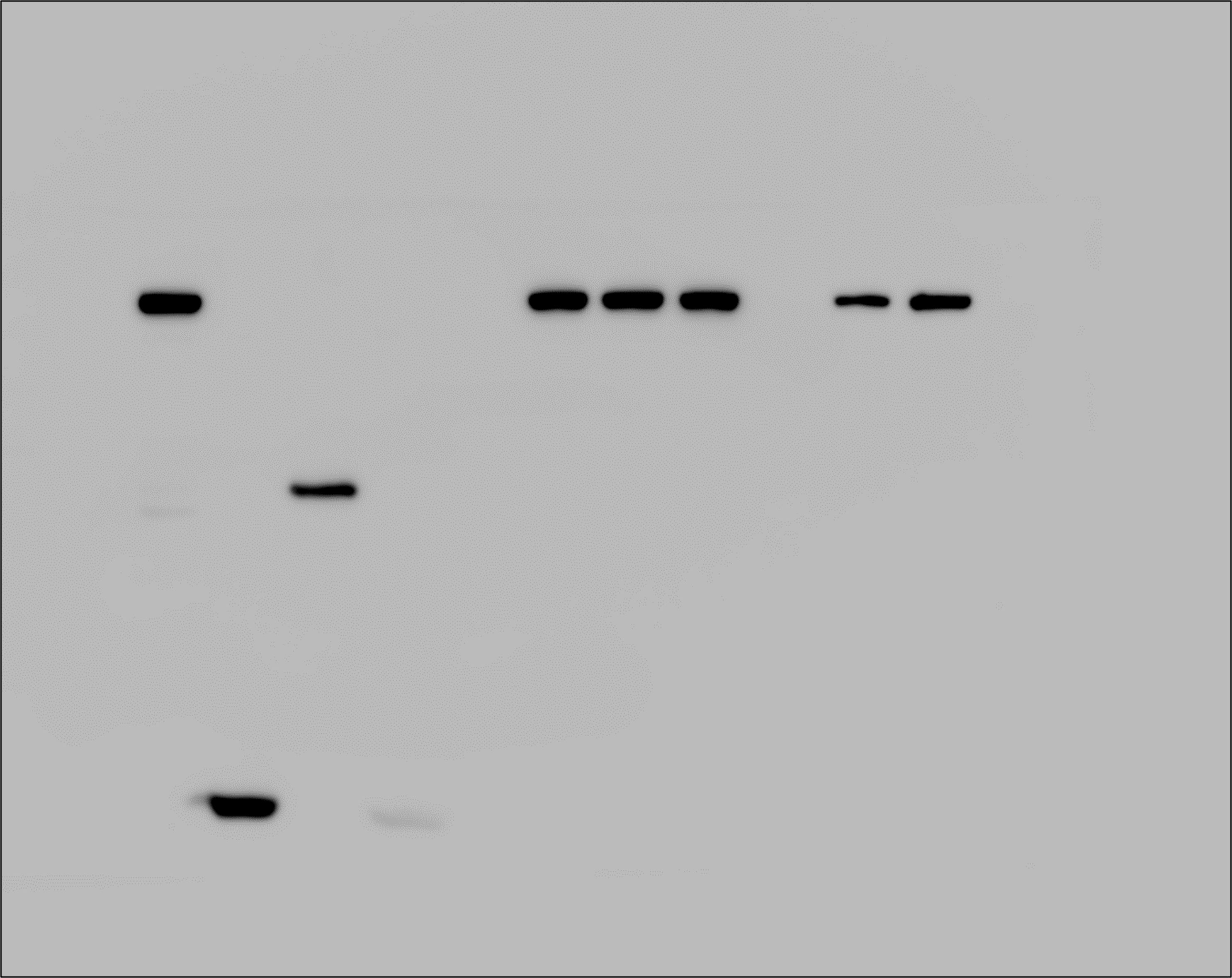

Supplement: Figure 9—figure supplement 1—source data 2. [file elife-108048-fig9-figsupp1-data2.zip › Figure 9-figure supplement 1/Figure S8 B-WCL-HA.tif]

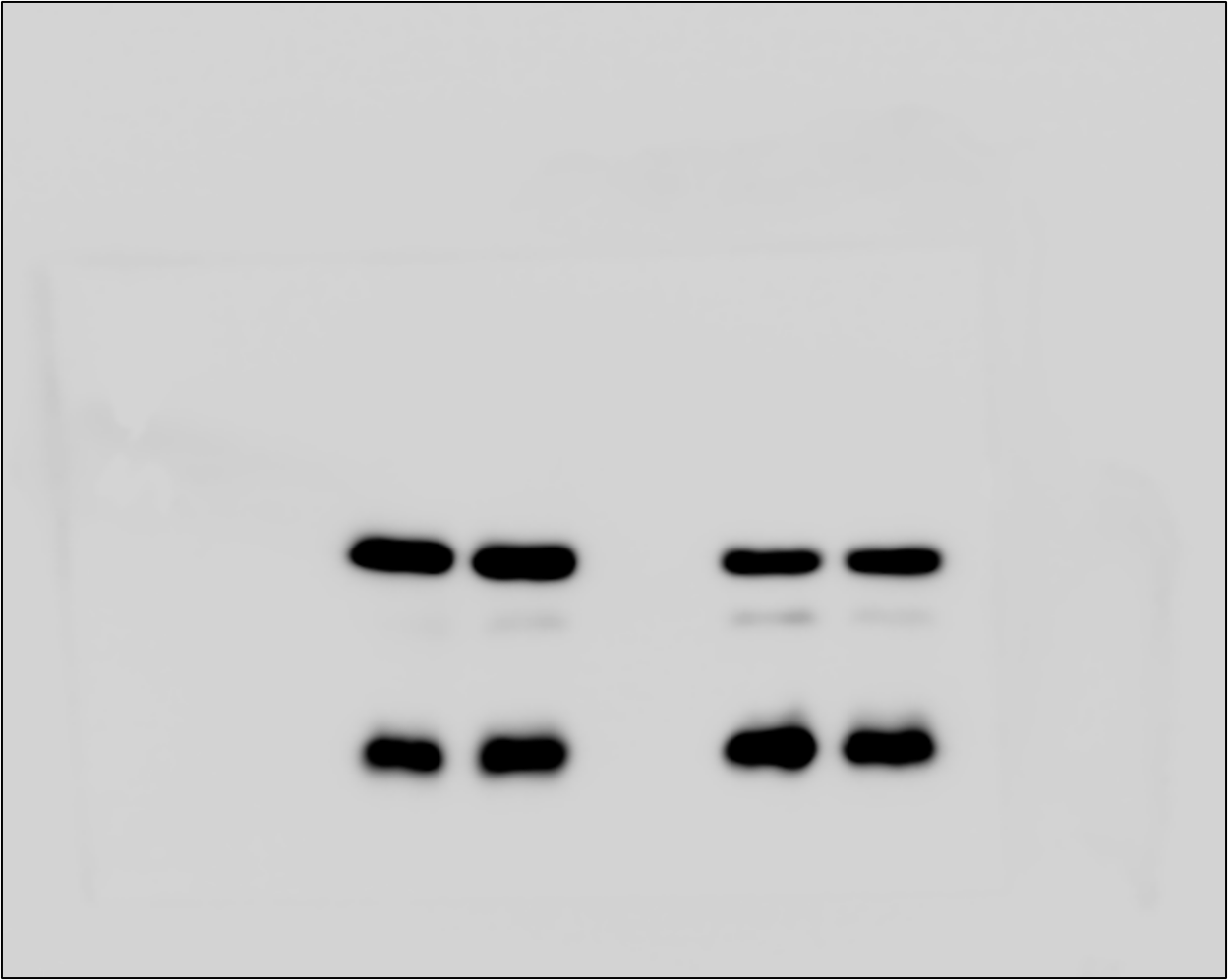

Supplement: Figure 9—figure supplement 1—source data 2. [file elife-108048-fig9-figsupp1-data2.zip › Figure 9-figure supplement 1/Figure S8 B-WCL-Myc.tif]

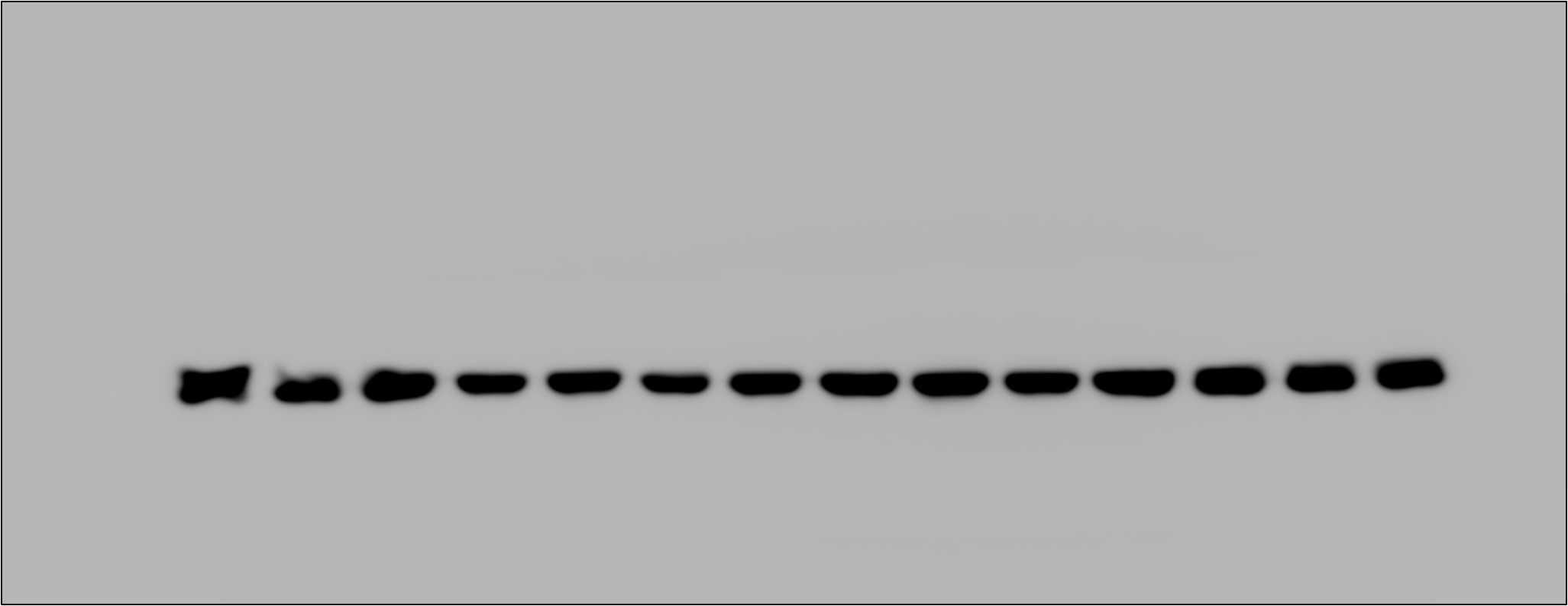

Supplement: Figure 9—figure supplement 1—source data 2. [file elife-108048-fig9-figsupp1-data2.zip › Figure 9-figure supplement 1/Figure S8 D-Actin.tif]

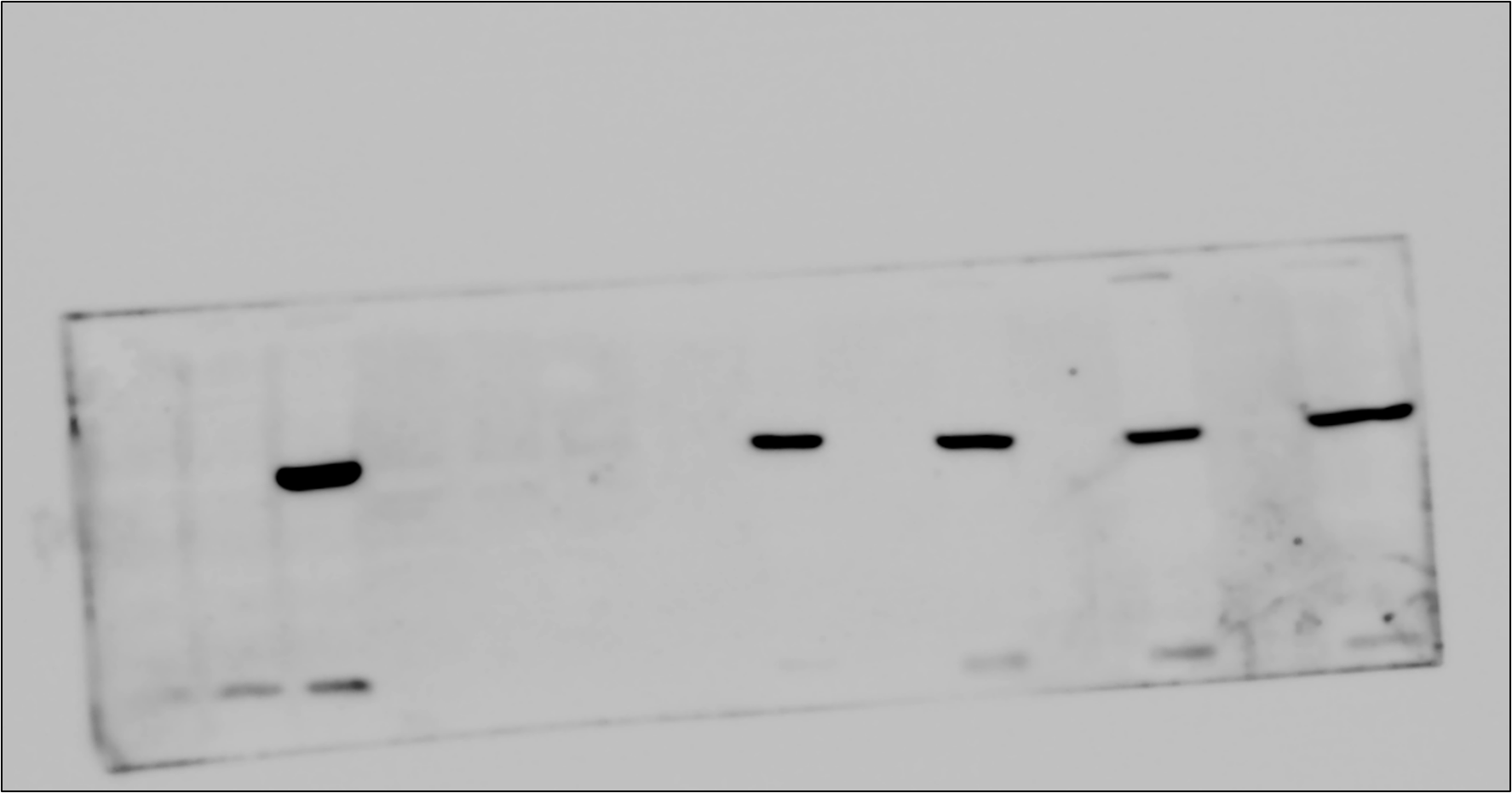

Supplement: Figure 9—figure supplement 1—source data 2. [file elife-108048-fig9-figsupp1-data2.zip › Figure 9-figure supplement 1/Figure S8 D-Flag.tif]

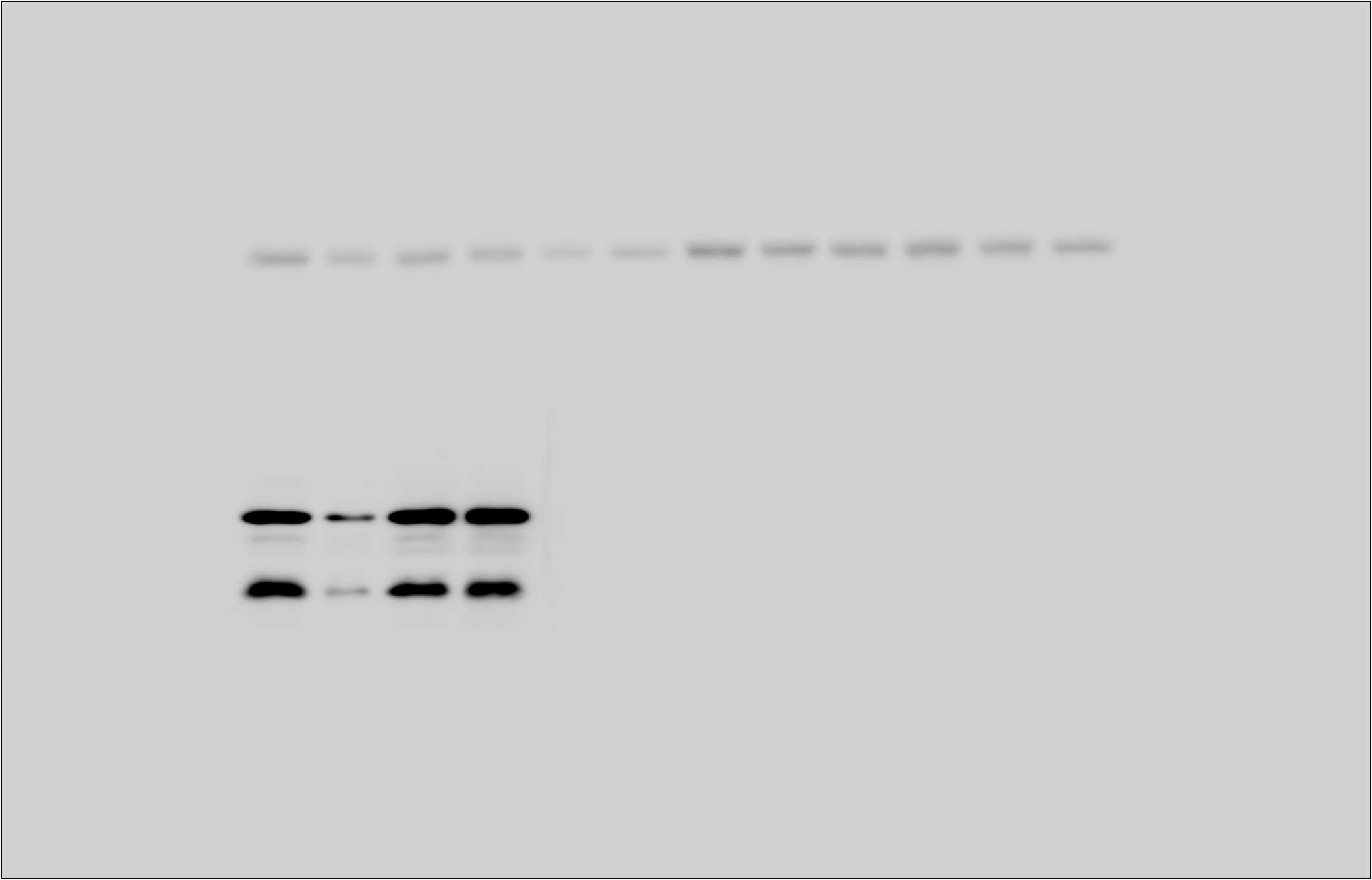

Supplement: Figure 9—figure supplement 1—source data 2. [file elife-108048-fig9-figsupp1-data2.zip › Figure 9-figure supplement 1/Figure S8 D-Myc.tif]

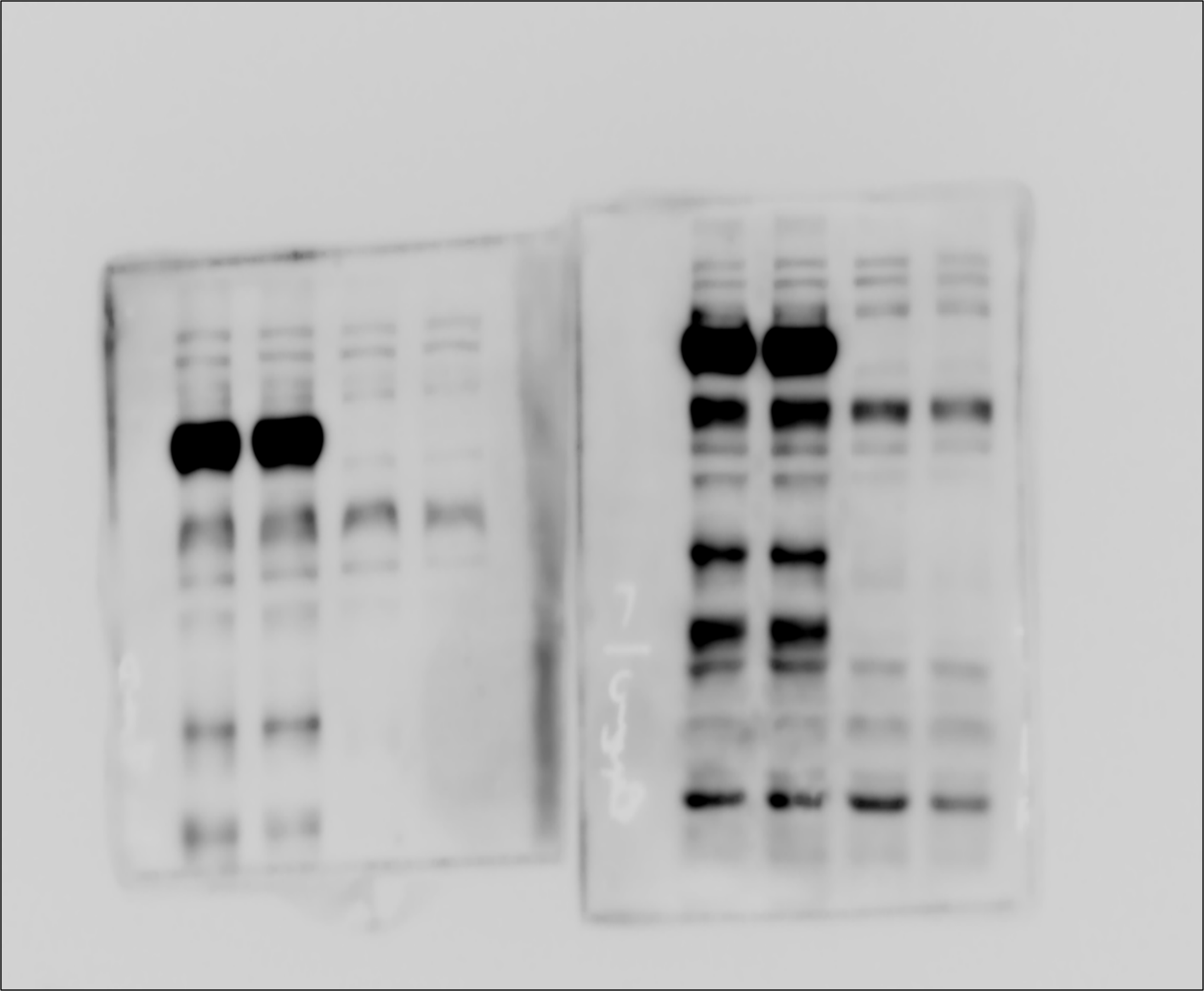

Supplement: Figure 9—figure supplement 1—source data 2. [file elife-108048-fig9-figsupp1-data2.zip › Figure 9-figure supplement 1/Figure S8 D-USP8.tif]

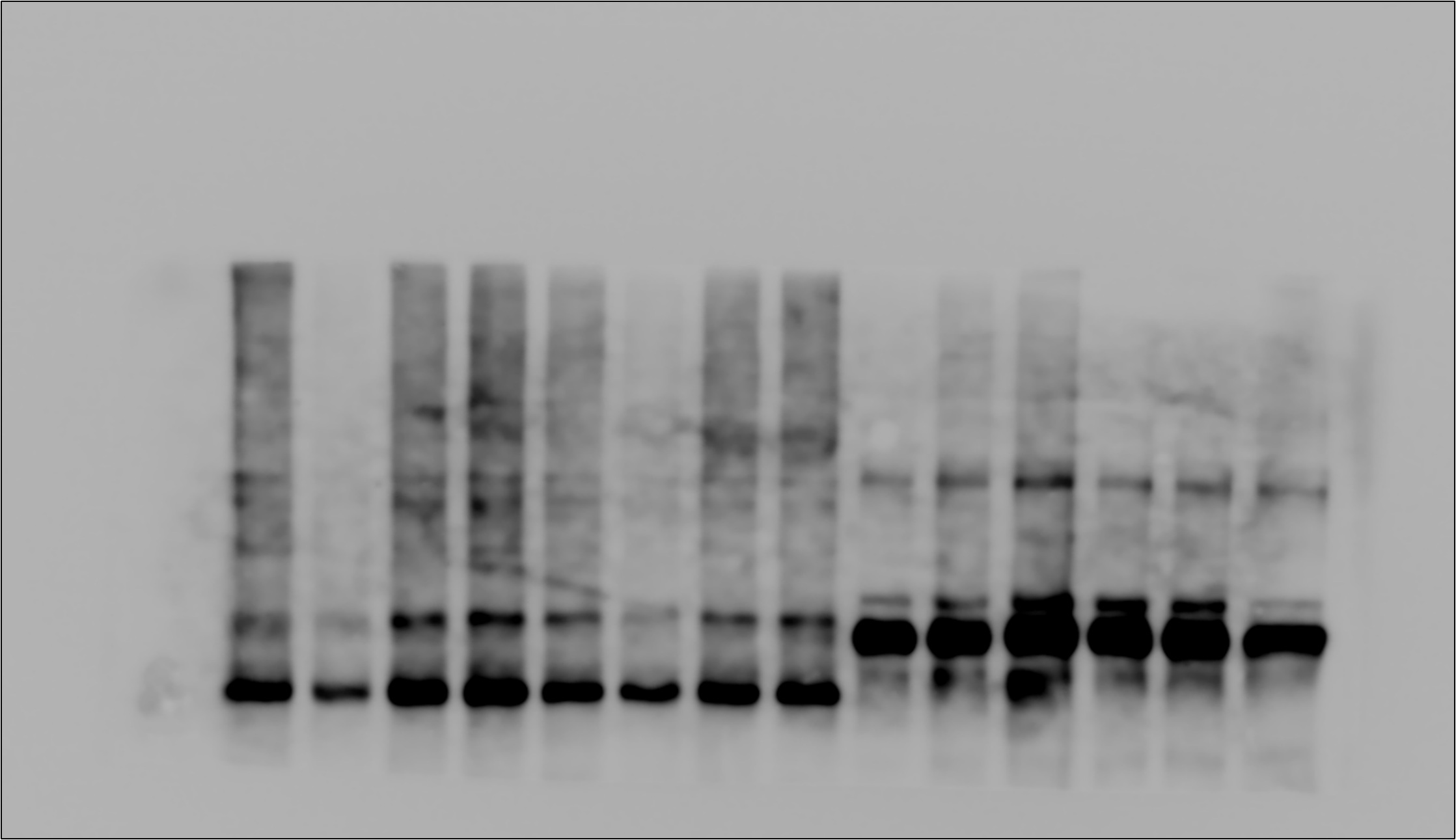

Supplement: Figure 9—figure supplement 1—source data 2. [file elife-108048-fig9-figsupp1-data2.zip › Figure 9-figure supplement 1/Figure S8 E-IP-HA.tif]

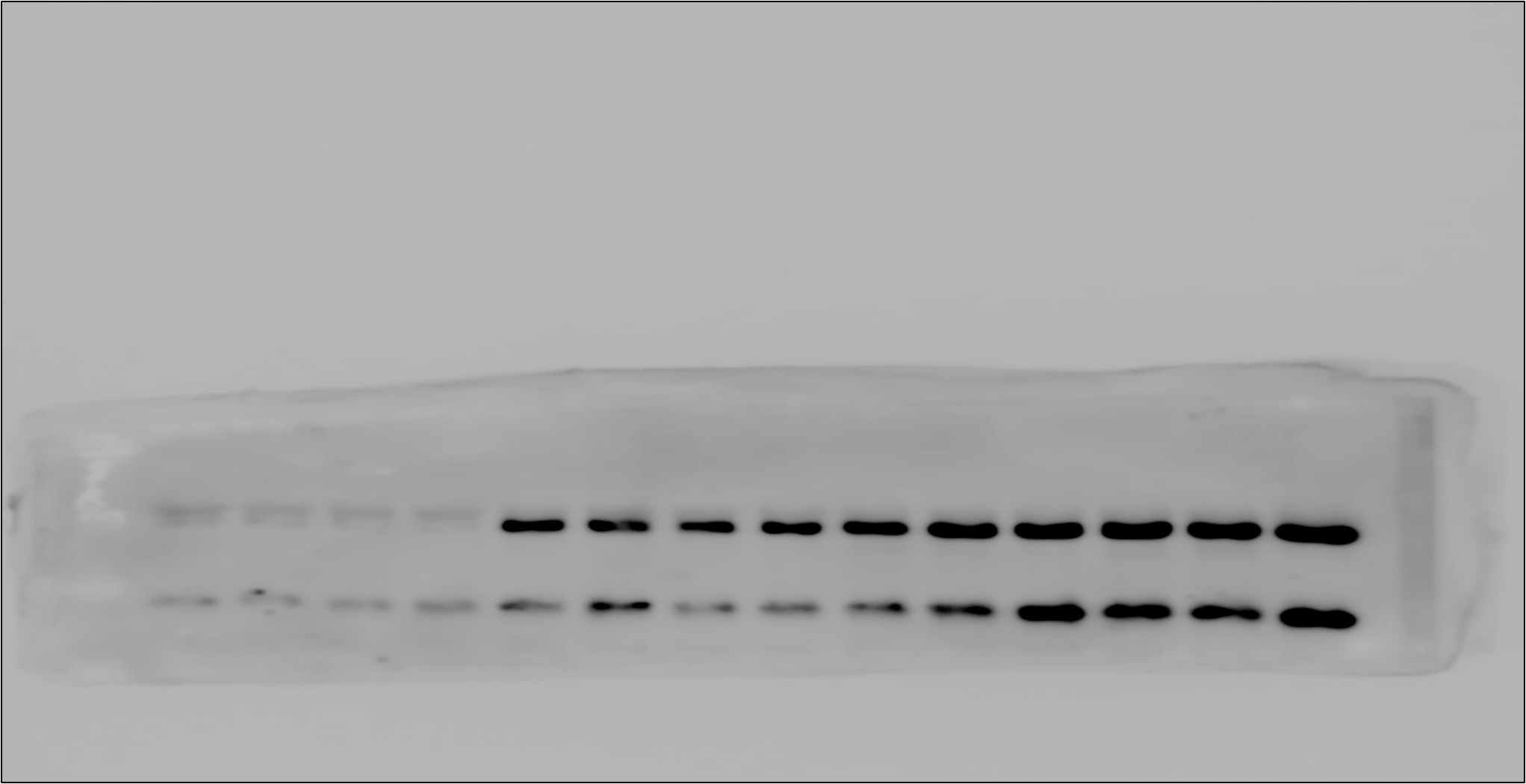

Supplement: Figure 9—figure supplement 1—source data 2. [file elife-108048-fig9-figsupp1-data2.zip › Figure 9-figure supplement 1/Figure S8 E-IP-Myc.tif]

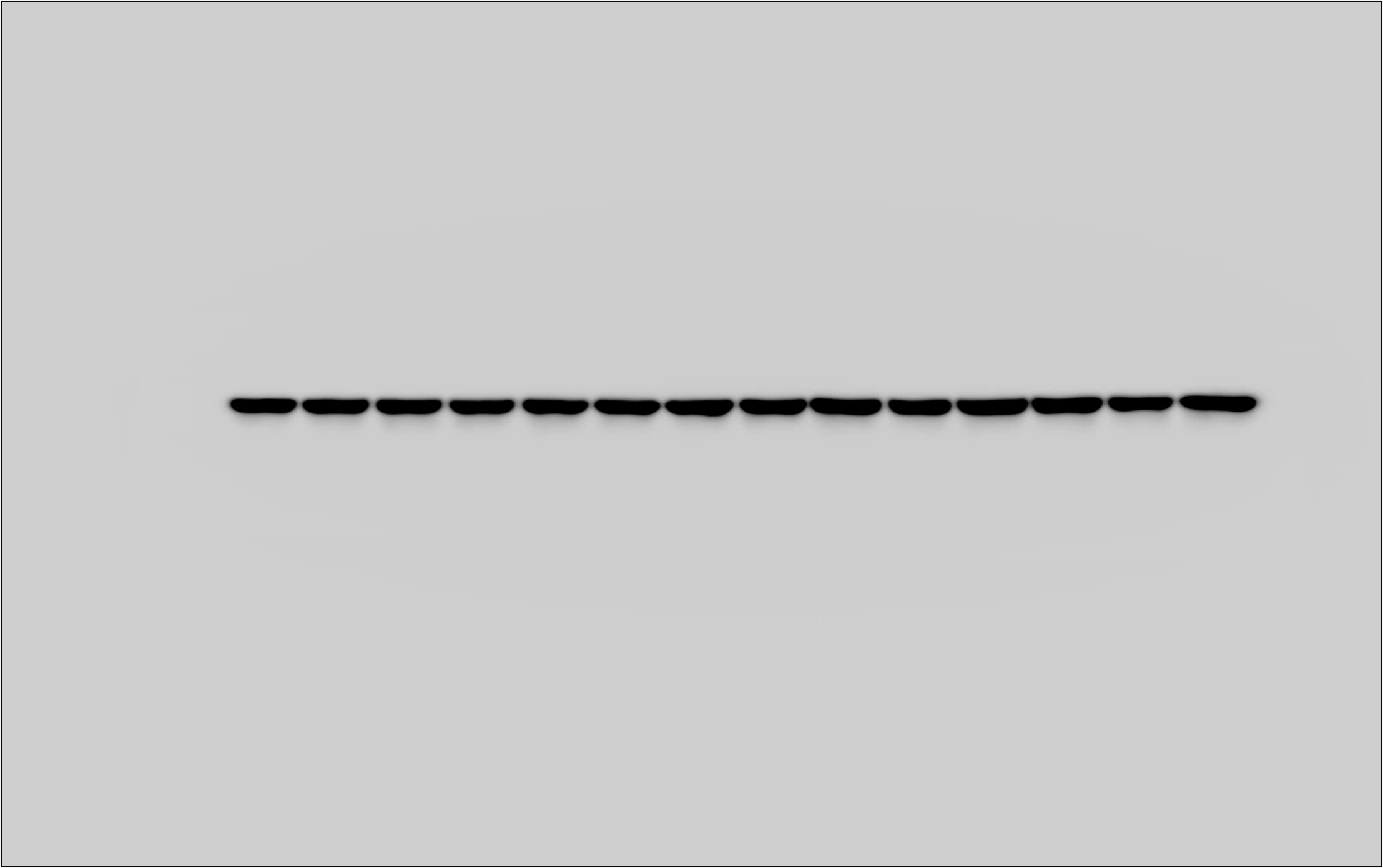

Supplement: Figure 9—figure supplement 1—source data 2. [file elife-108048-fig9-figsupp1-data2.zip › Figure 9-figure supplement 1/Figure S8 E-WCL-Actin.tif]

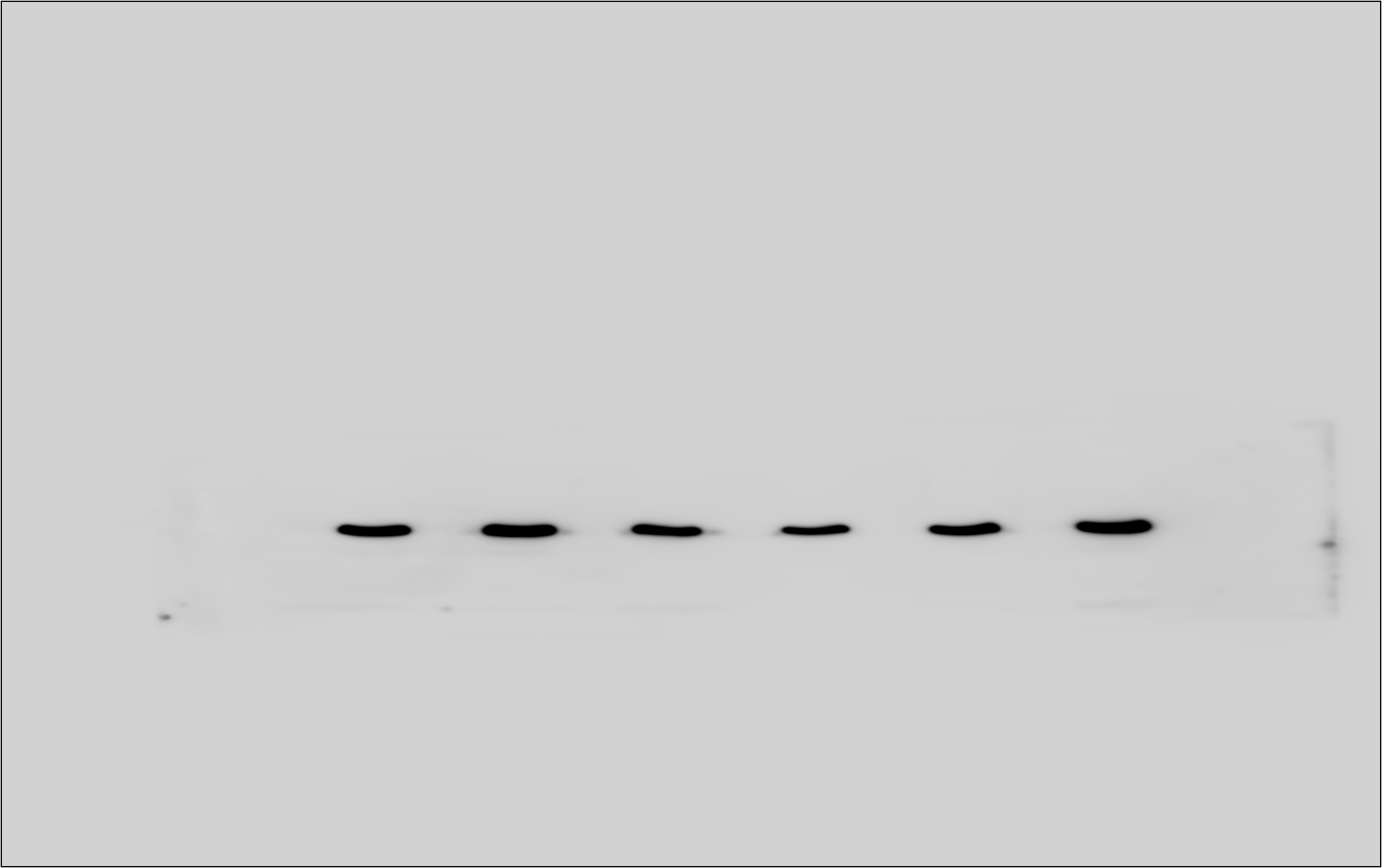

Supplement: Figure 9—figure supplement 1—source data 2. [file elife-108048-fig9-figsupp1-data2.zip › Figure 9-figure supplement 1/Figure S8 E-WCL-Flag.tif]

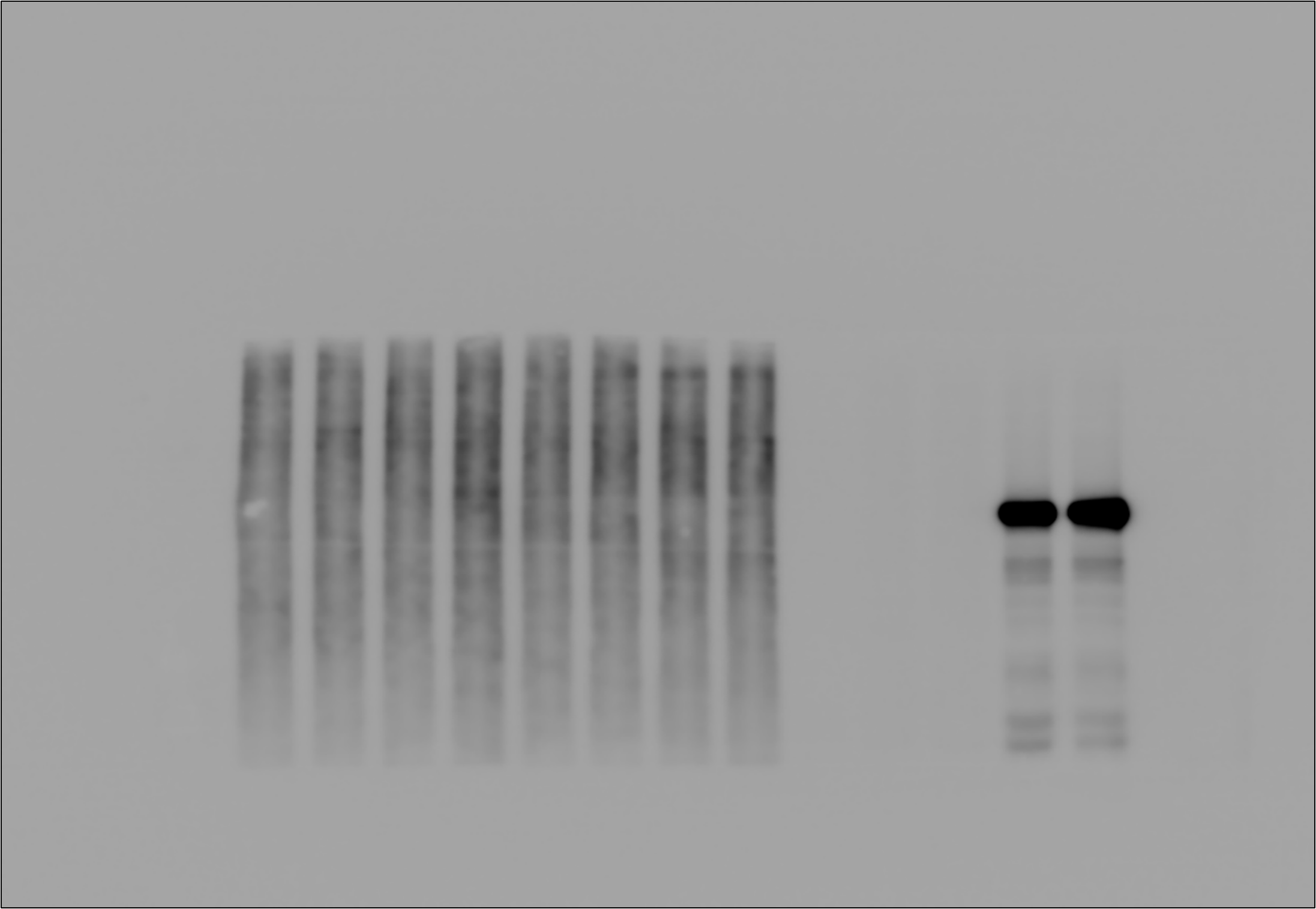

Supplement: Figure 9—figure supplement 1—source data 2. [file elife-108048-fig9-figsupp1-data2.zip › Figure 9-figure supplement 1/Figure S8 E-WCL-HA.tif]

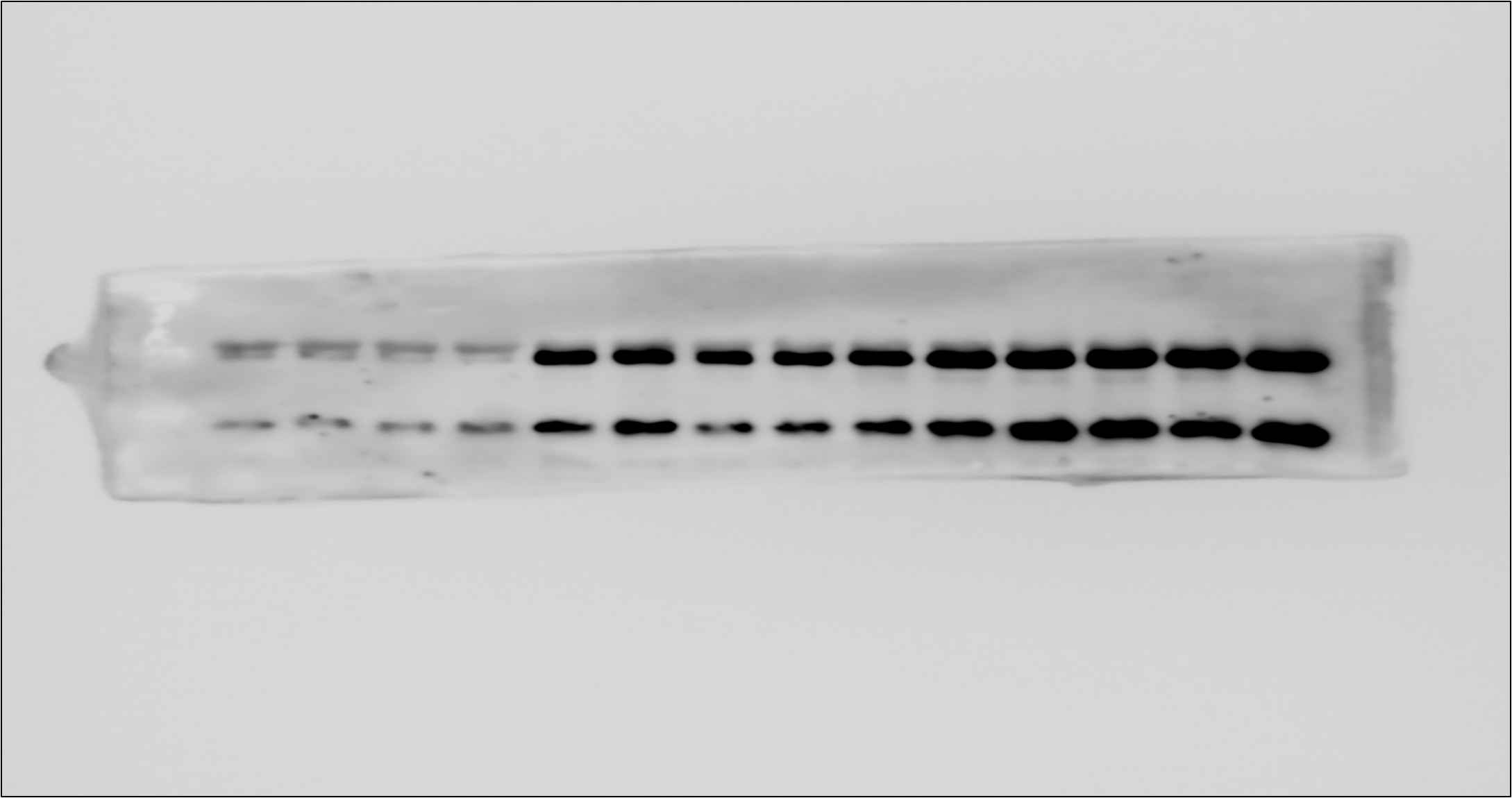

Supplement: Figure 9—figure supplement 1—source data 2. [file elife-108048-fig9-figsupp1-data2.zip › Figure 9-figure supplement 1/Figure S8 E-WCL-Myc.tif]

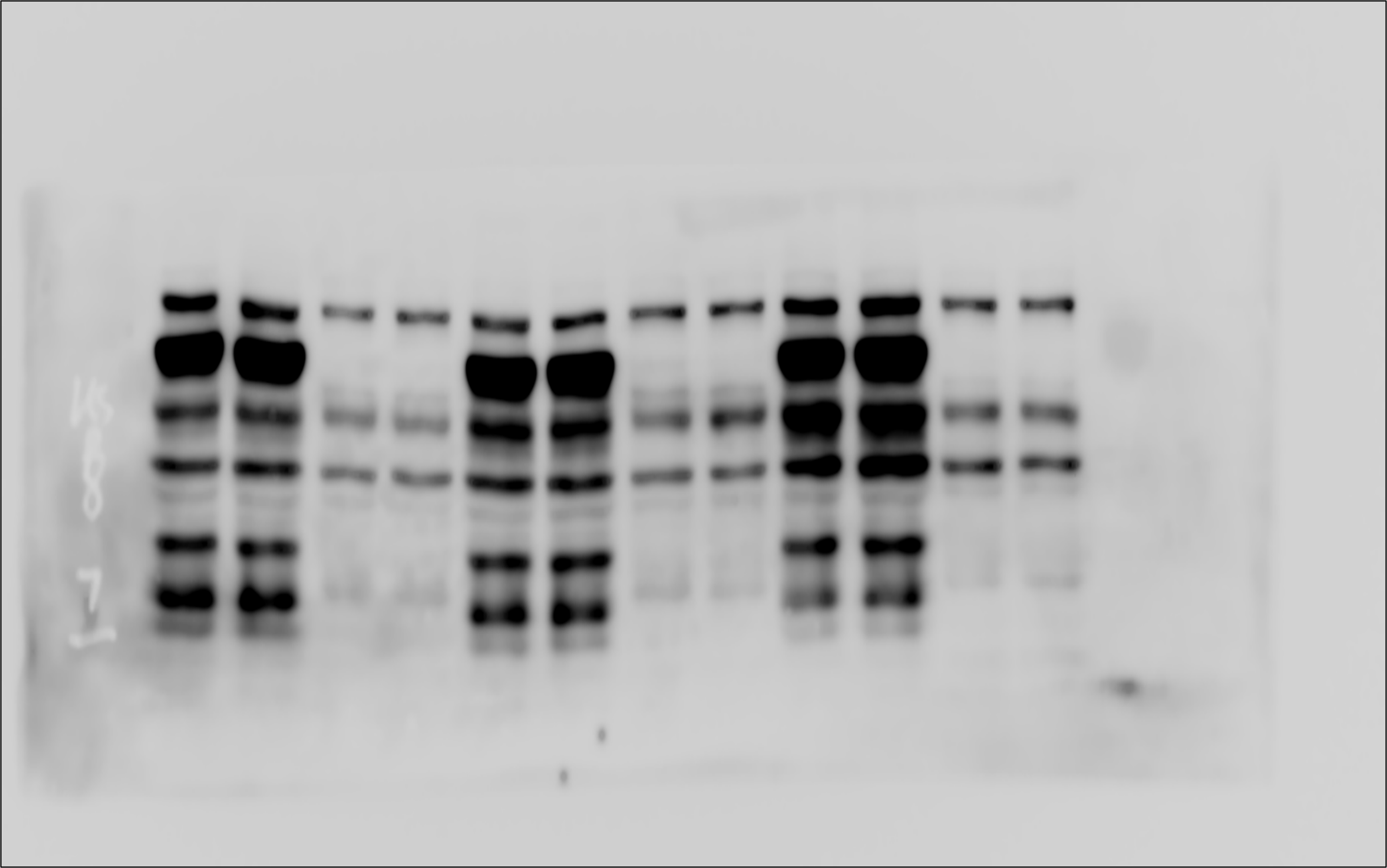

Supplement: Figure 9—figure supplement 1—source data 2. [file elife-108048-fig9-figsupp1-data2.zip › Figure 9-figure supplement 1/Figure S8 E-WCL-USP8.tif]

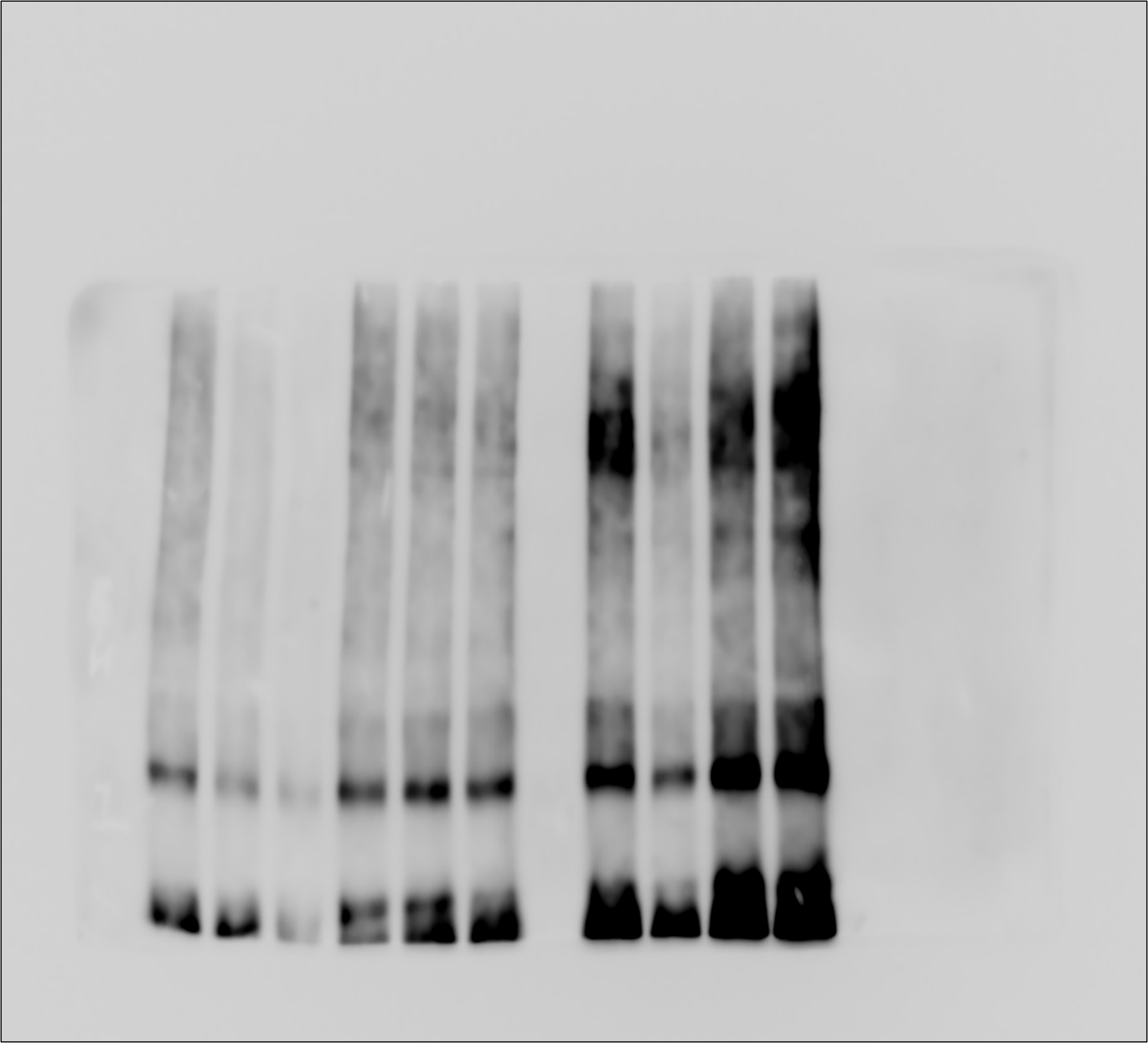

Supplement: Figure 9—figure supplement 1—source data 2. [file elife-108048-fig9-figsupp1-data2.zip › Figure 9-figure supplement 1/Figure S8 F-IP-HA.tif]

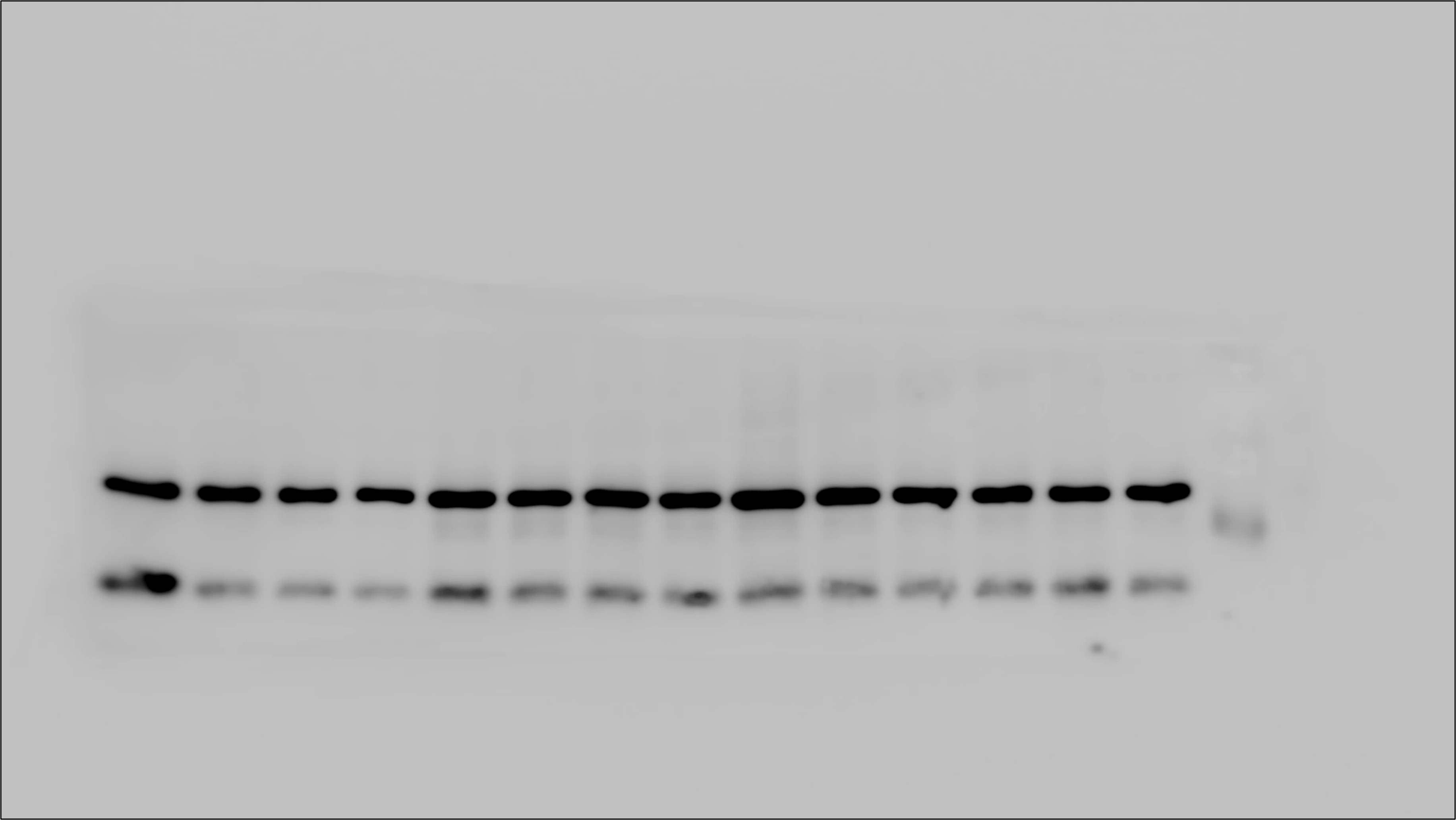

Supplement: Figure 9—figure supplement 1—source data 2. [file elife-108048-fig9-figsupp1-data2.zip › Figure 9-figure supplement 1/Figure S8 F-IP-Myc.tif]

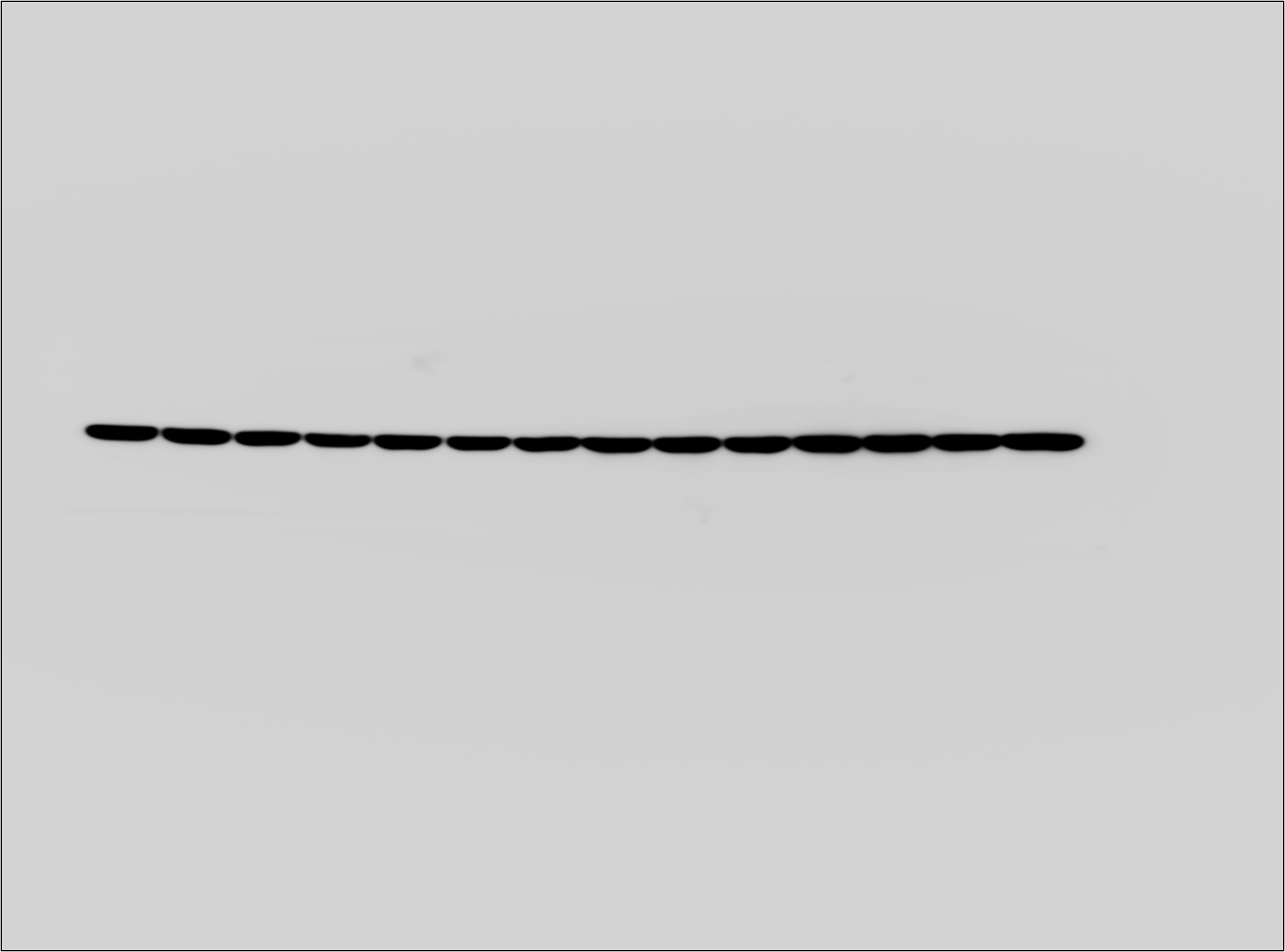

Supplement: Figure 9—figure supplement 1—source data 2. [file elife-108048-fig9-figsupp1-data2.zip › Figure 9-figure supplement 1/Figure S8 F-WCL-Actin.tif]

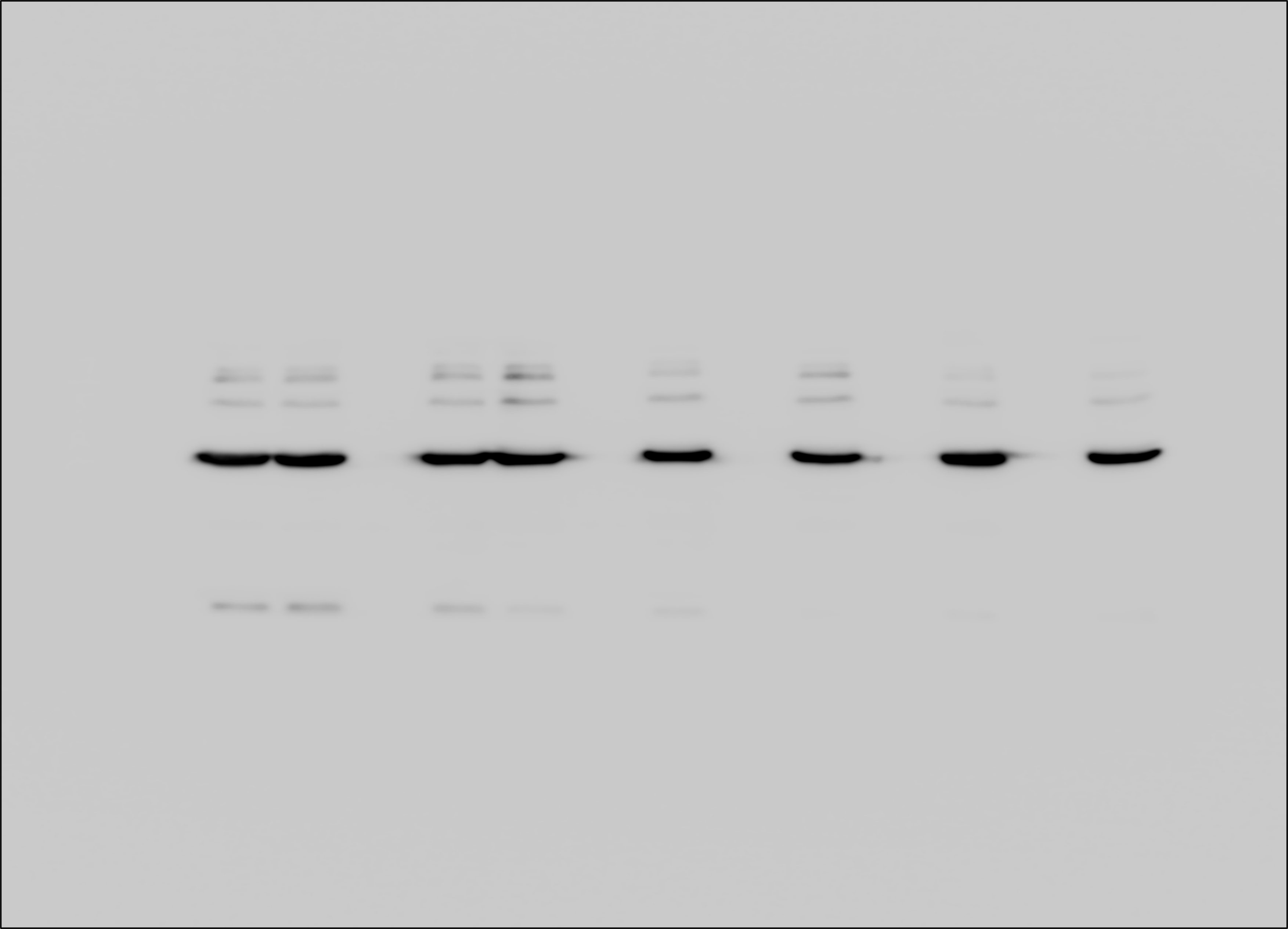

Supplement: Figure 9—figure supplement 1—source data 2. [file elife-108048-fig9-figsupp1-data2.zip › Figure 9-figure supplement 1/Figure S8 F-WCL-Flag.tif]

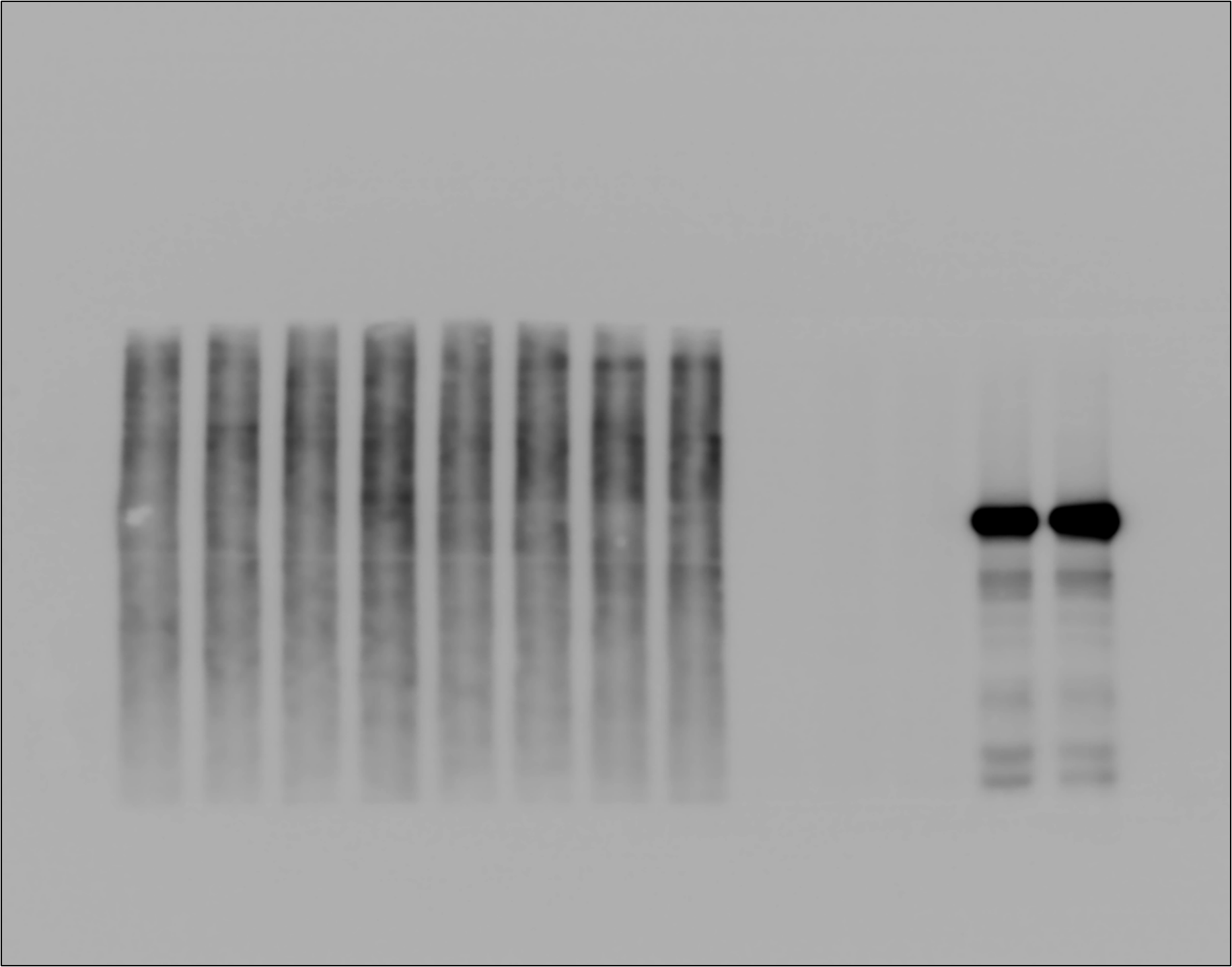

Supplement: Figure 9—figure supplement 1—source data 2. [file elife-108048-fig9-figsupp1-data2.zip › Figure 9-figure supplement 1/Figure S8 F-WCL-HA.tif]

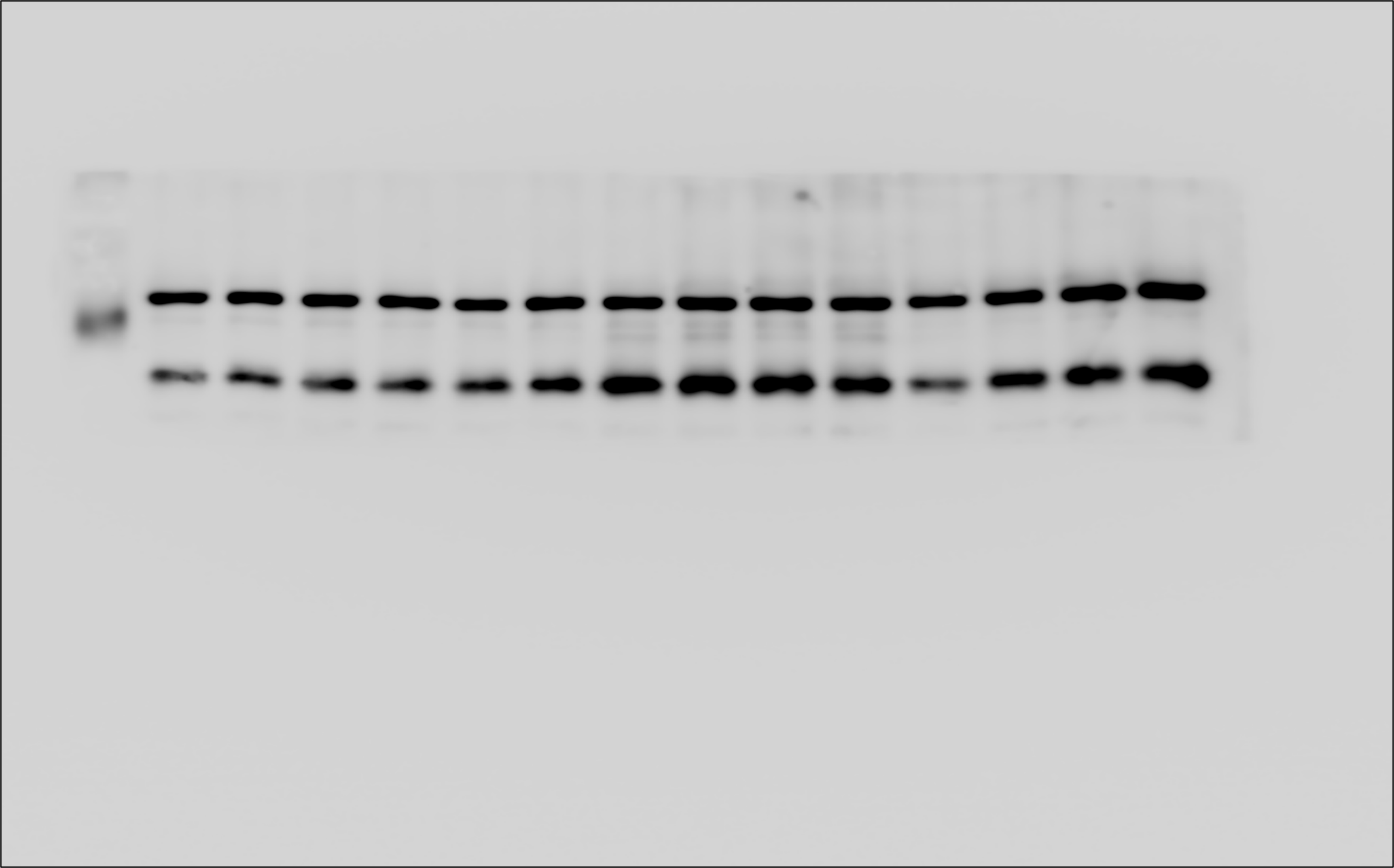

Supplement: Figure 9—figure supplement 1—source data 2. [file elife-108048-fig9-figsupp1-data2.zip › Figure 9-figure supplement 1/Figure S8 F-WCL-Myc.tif]

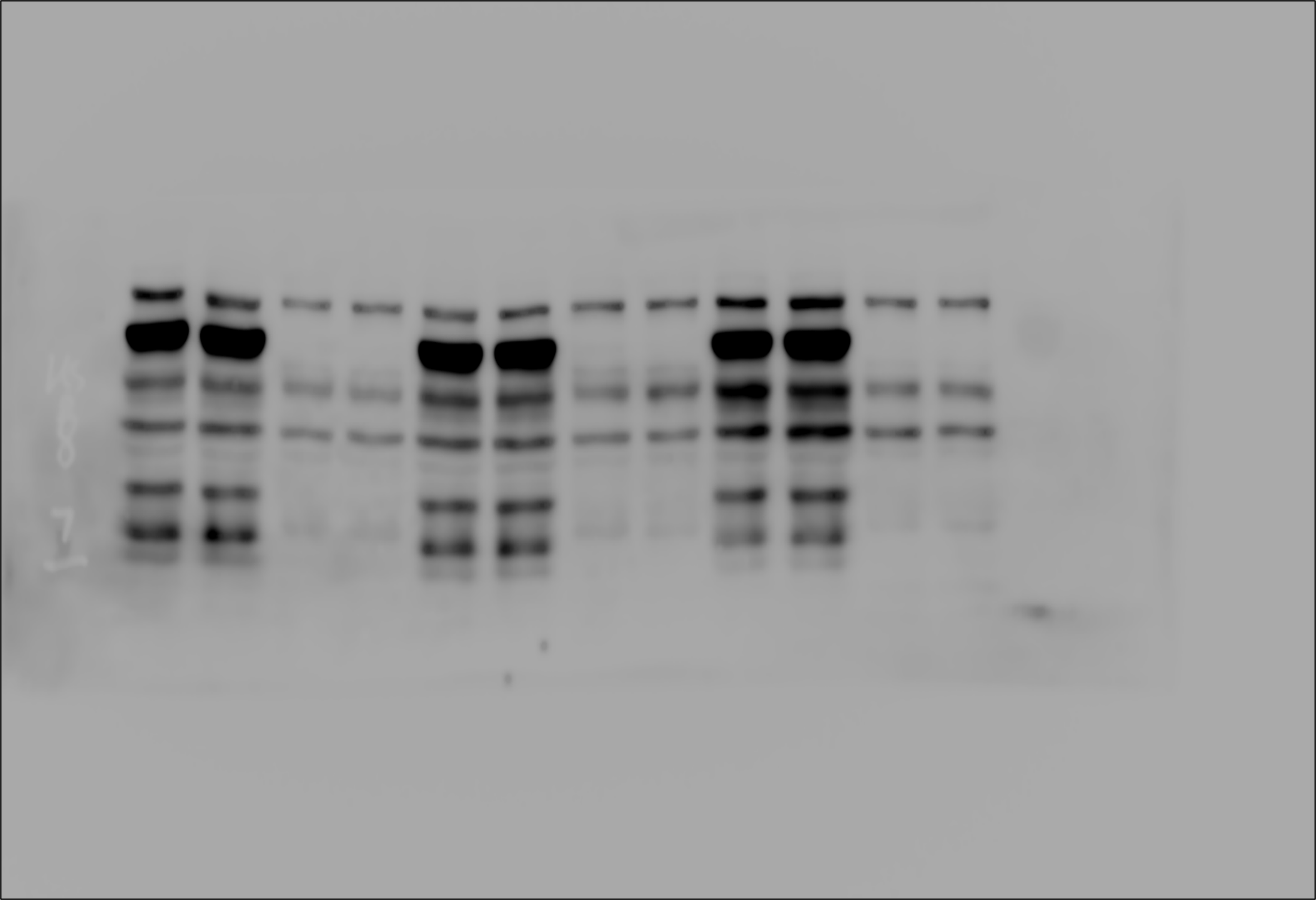

Supplement: Figure 9—figure supplement 1—source data 2. [file elife-108048-fig9-figsupp1-data2.zip › Figure 9-figure supplement 1/Figure S8 F-WCL-USP8.tif]

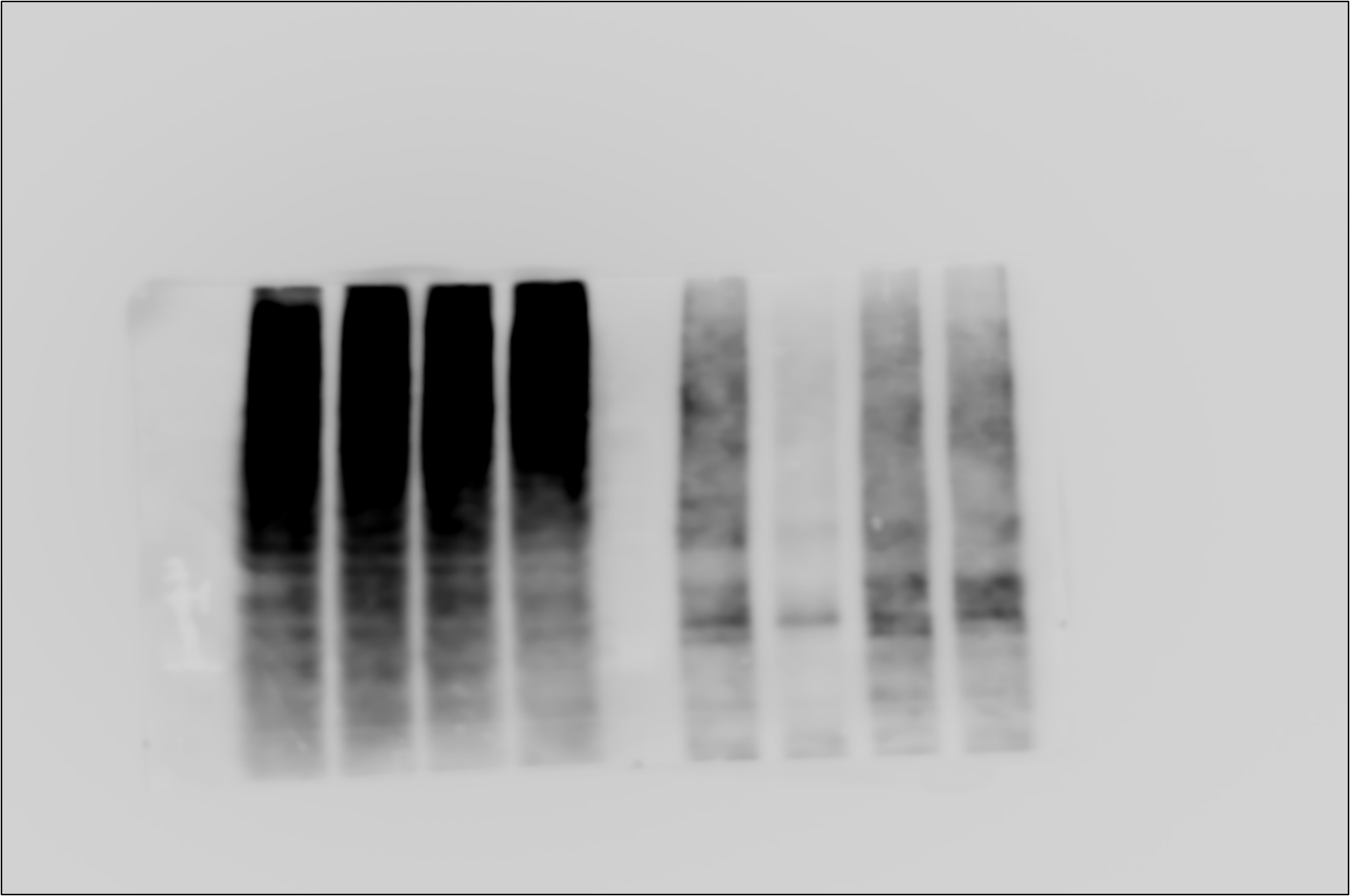

Supplement: Figure 9—figure supplement 1—source data 2. [file elife-108048-fig9-figsupp1-data2.zip › Figure 9-figure supplement 1/Figure S8 G-IP-HA.tif]

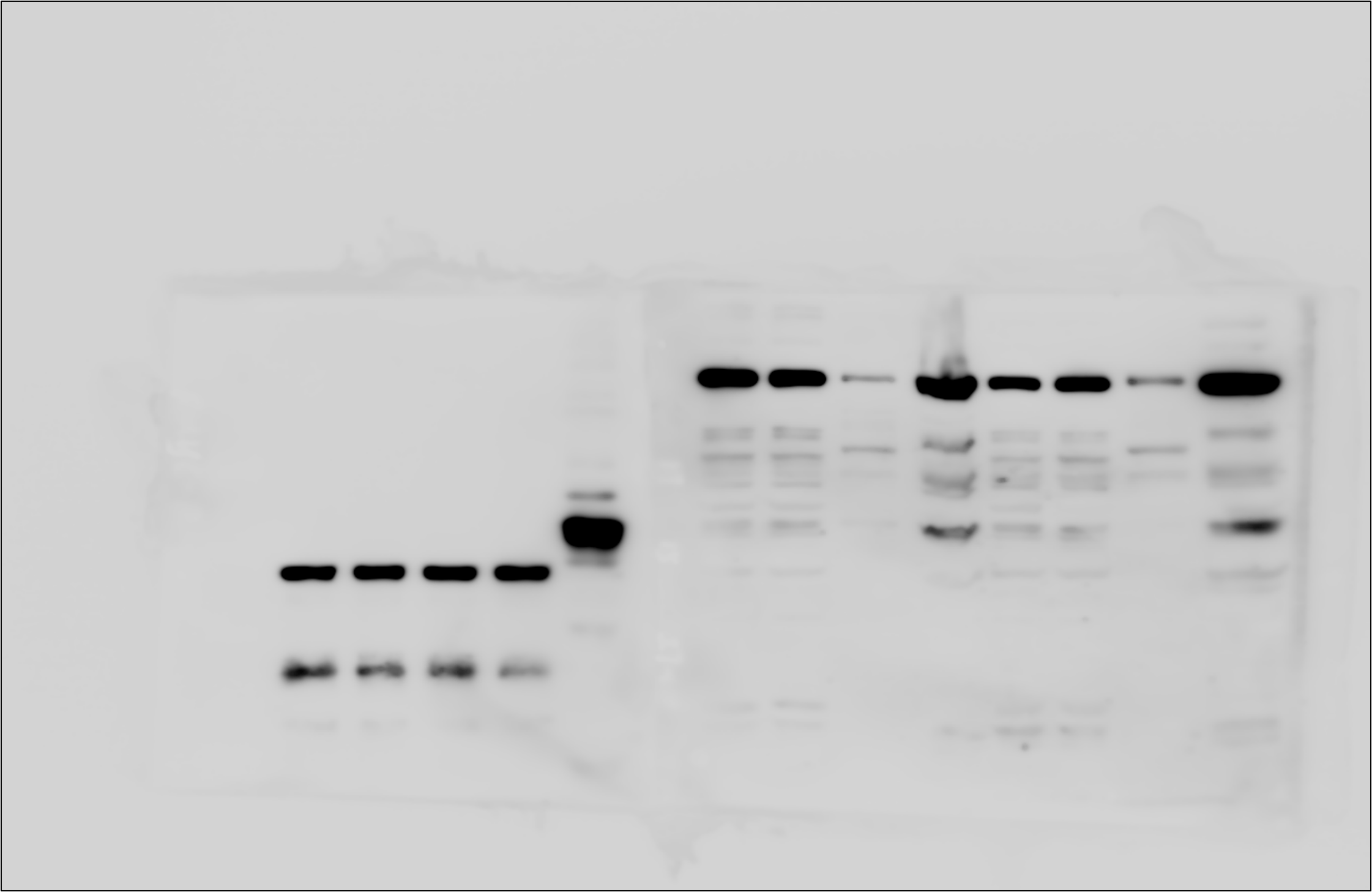

Supplement: Figure 9—figure supplement 1—source data 2. [file elife-108048-fig9-figsupp1-data2.zip › Figure 9-figure supplement 1/Figure S8 G-IP-Myc.tif]

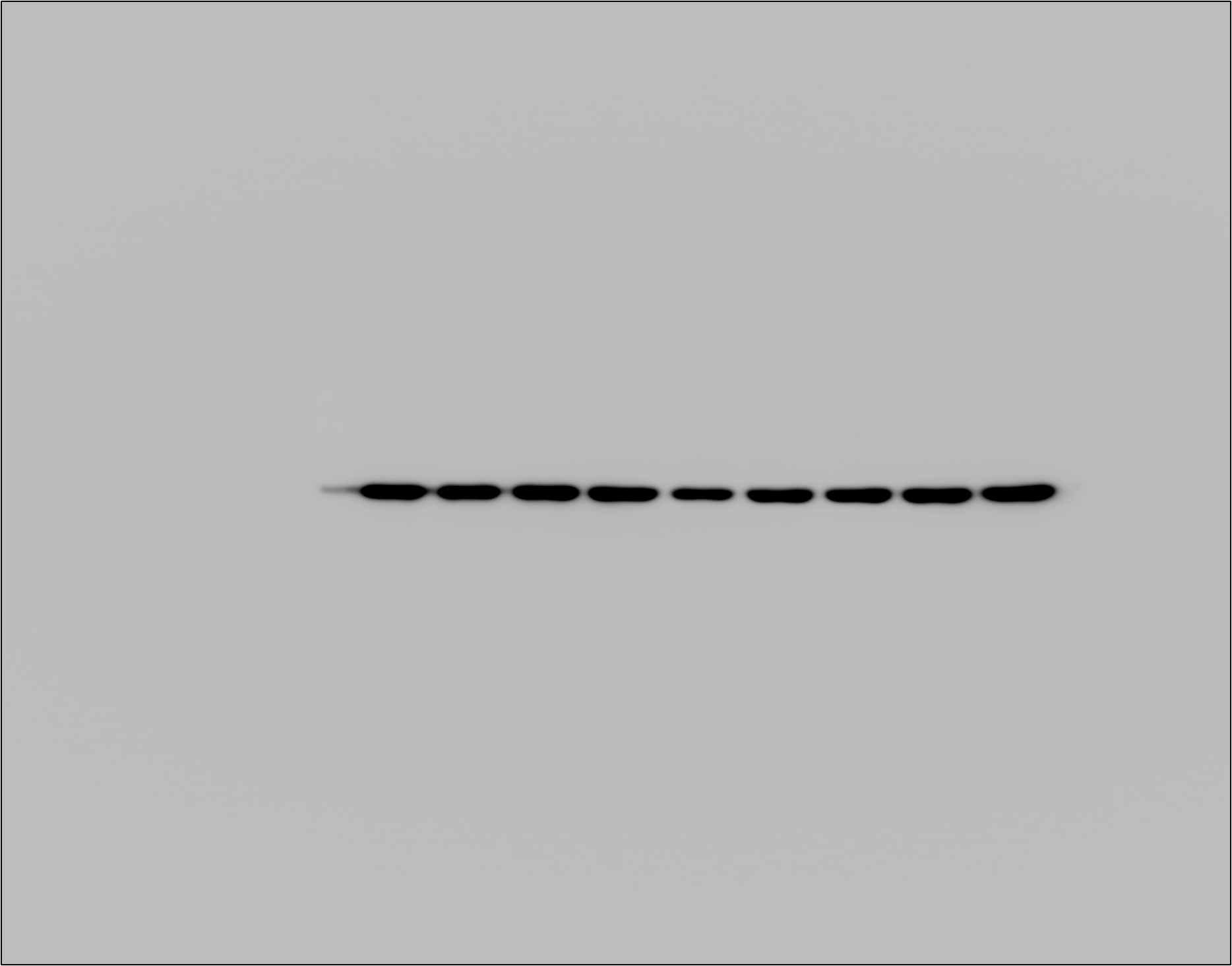

Supplement: Figure 9—figure supplement 1—source data 2. [file elife-108048-fig9-figsupp1-data2.zip › Figure 9-figure supplement 1/Figure S8 G-WCL-Actin.tif]

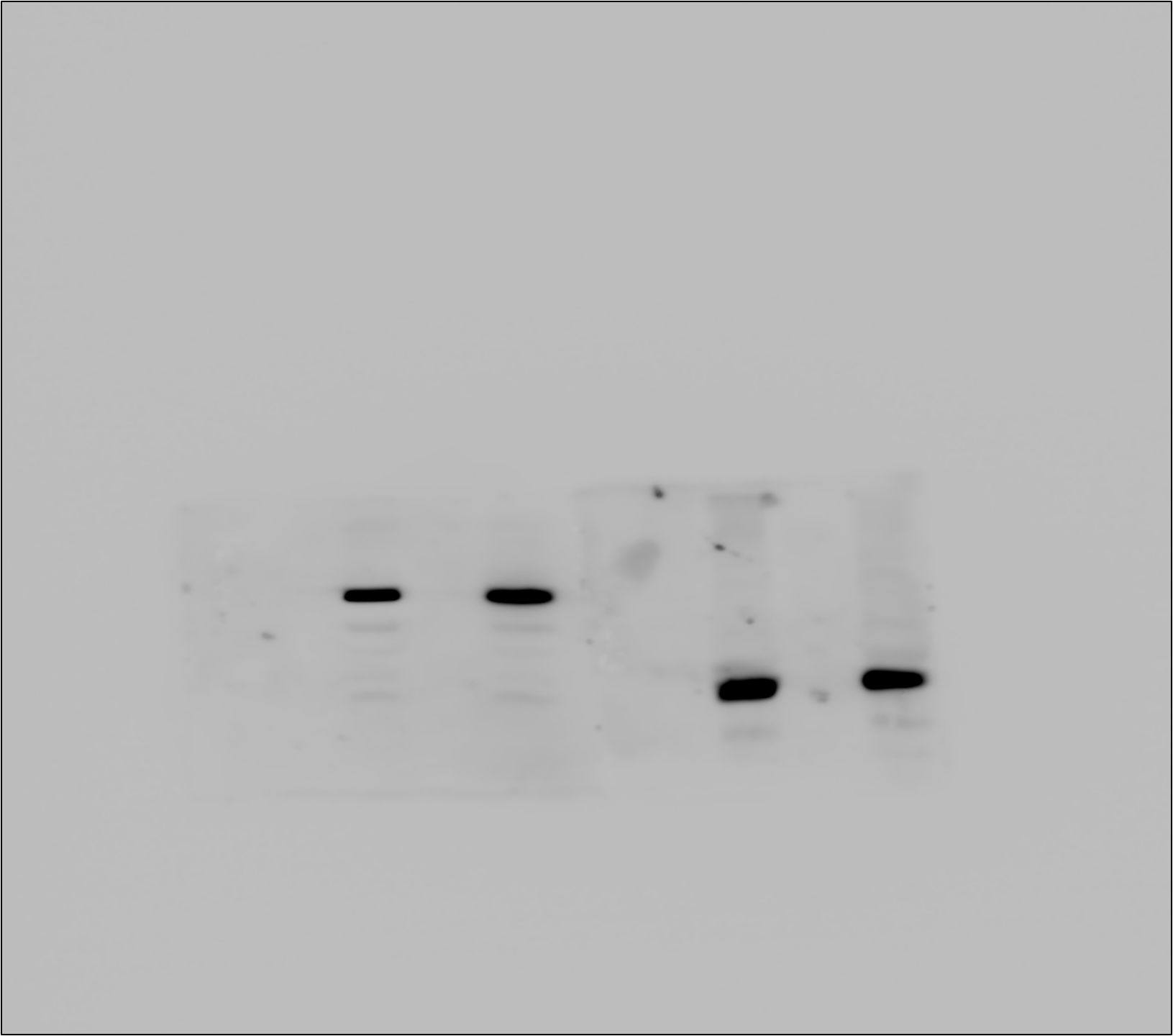

Supplement: Figure 9—figure supplement 1—source data 2. [file elife-108048-fig9-figsupp1-data2.zip › Figure 9-figure supplement 1/Figure S8 G-WCL-Flag.tif]

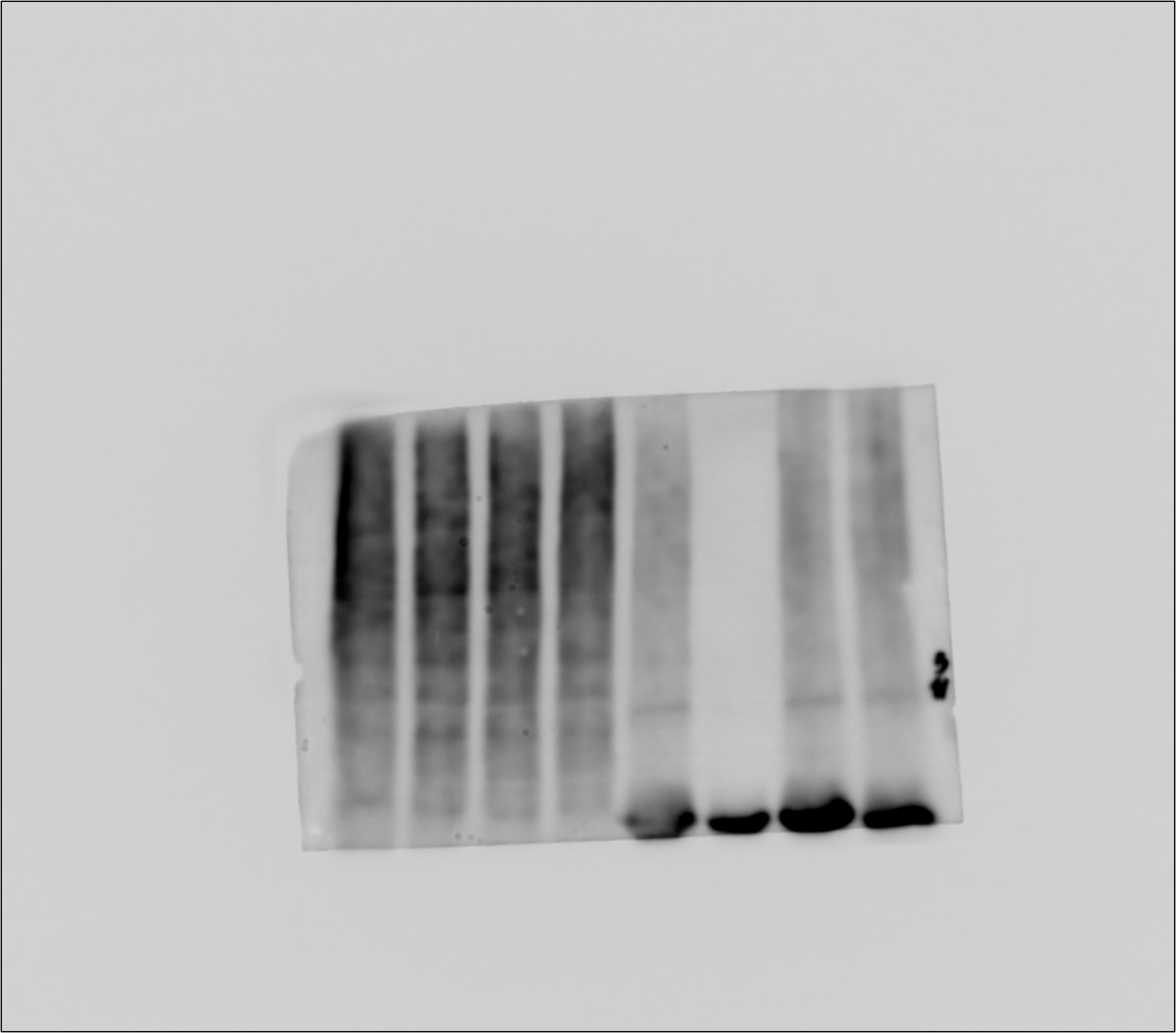

Supplement: Figure 9—figure supplement 1—source data 2. [file elife-108048-fig9-figsupp1-data2.zip › Figure 9-figure supplement 1/Figure S8 G-WCL-HA.tif]

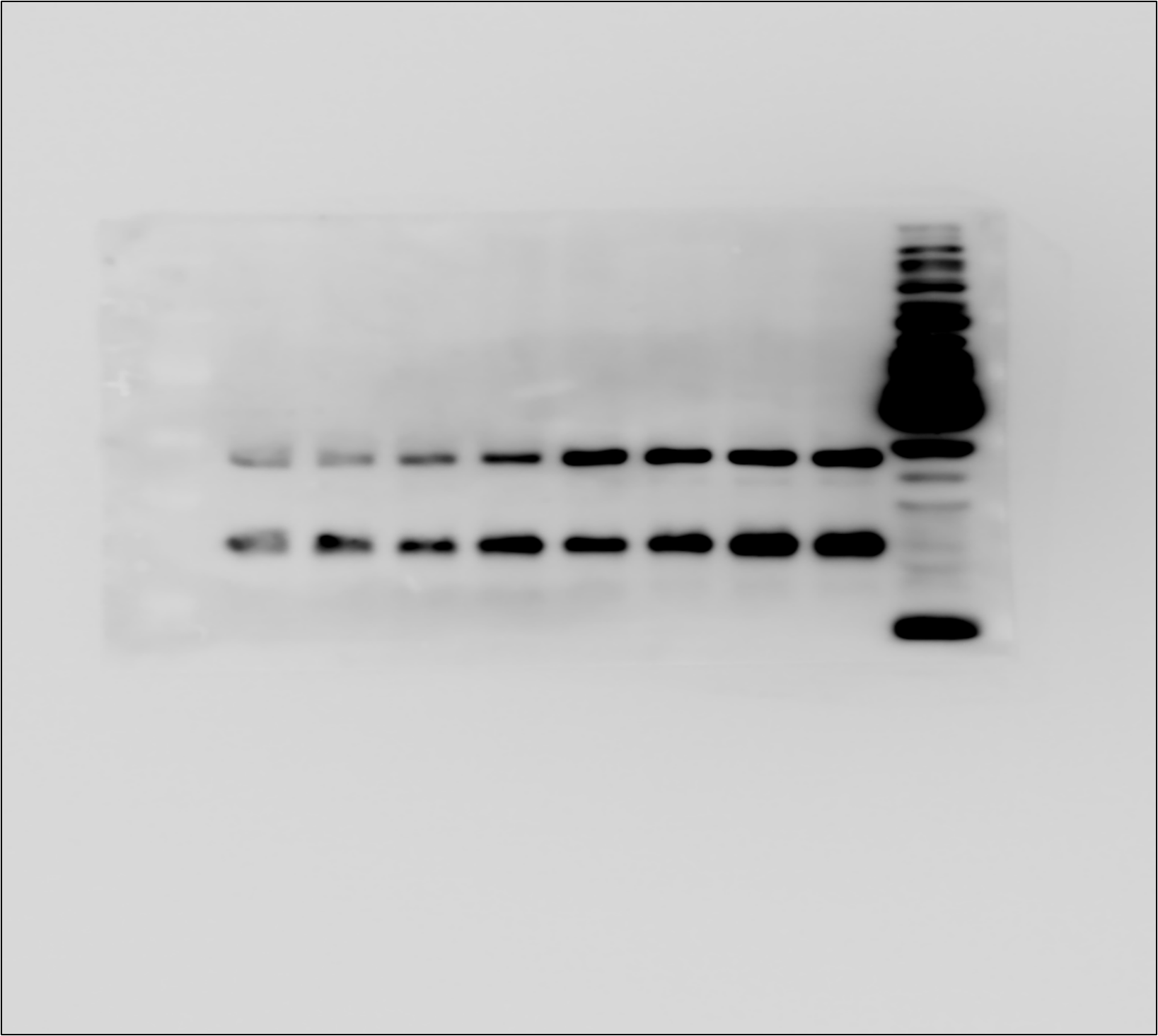

Supplement: Figure 9—figure supplement 1—source data 2. [file elife-108048-fig9-figsupp1-data2.zip › Figure 9-figure supplement 1/Figure S8 G-WCL-Myc.tif]

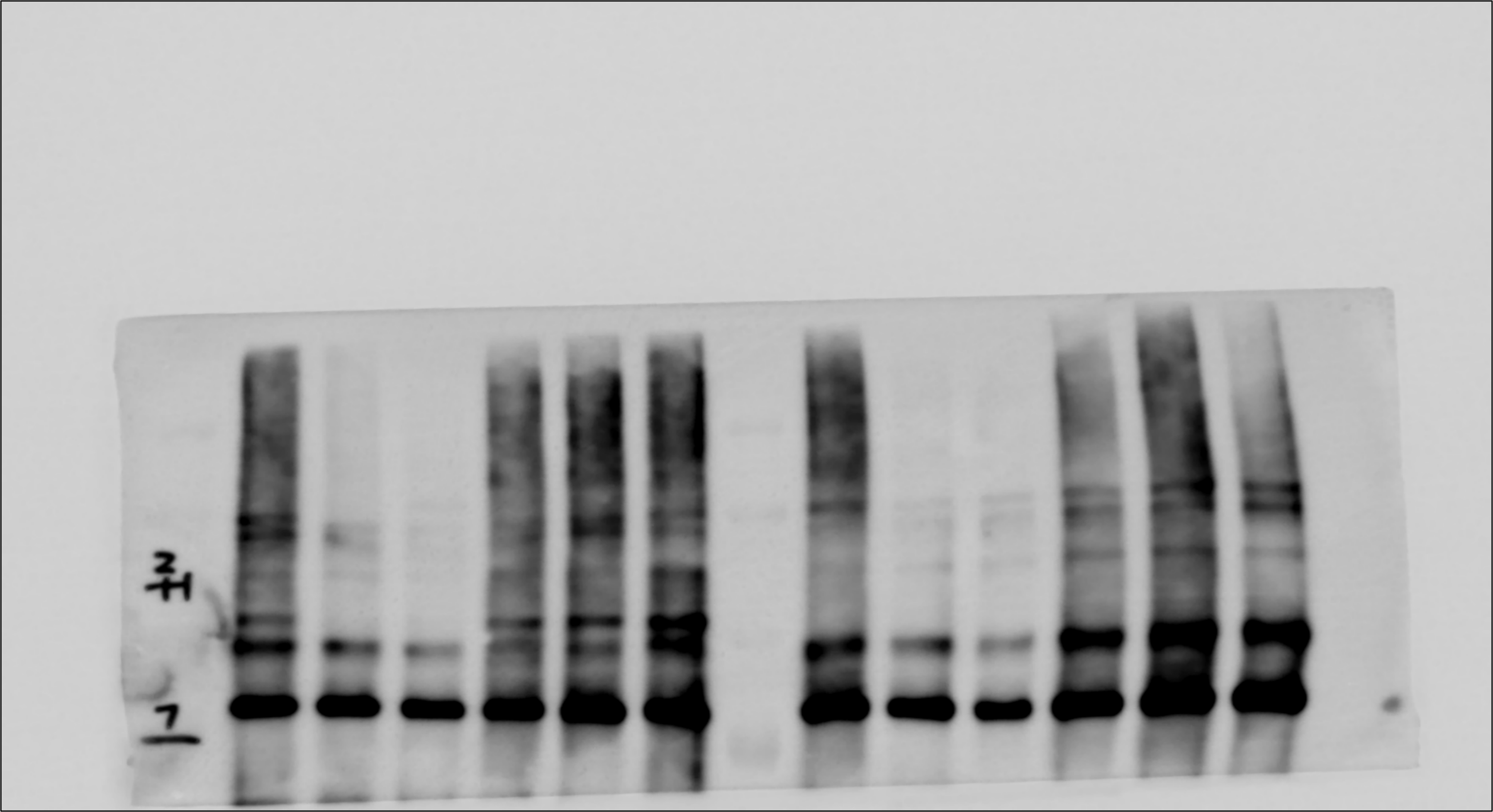

Supplement: Figure 9—figure supplement 1—source data 2. [file elife-108048-fig9-figsupp1-data2.zip › Figure 9-figure supplement 1/Figure S8 H-IP-HA.tif]

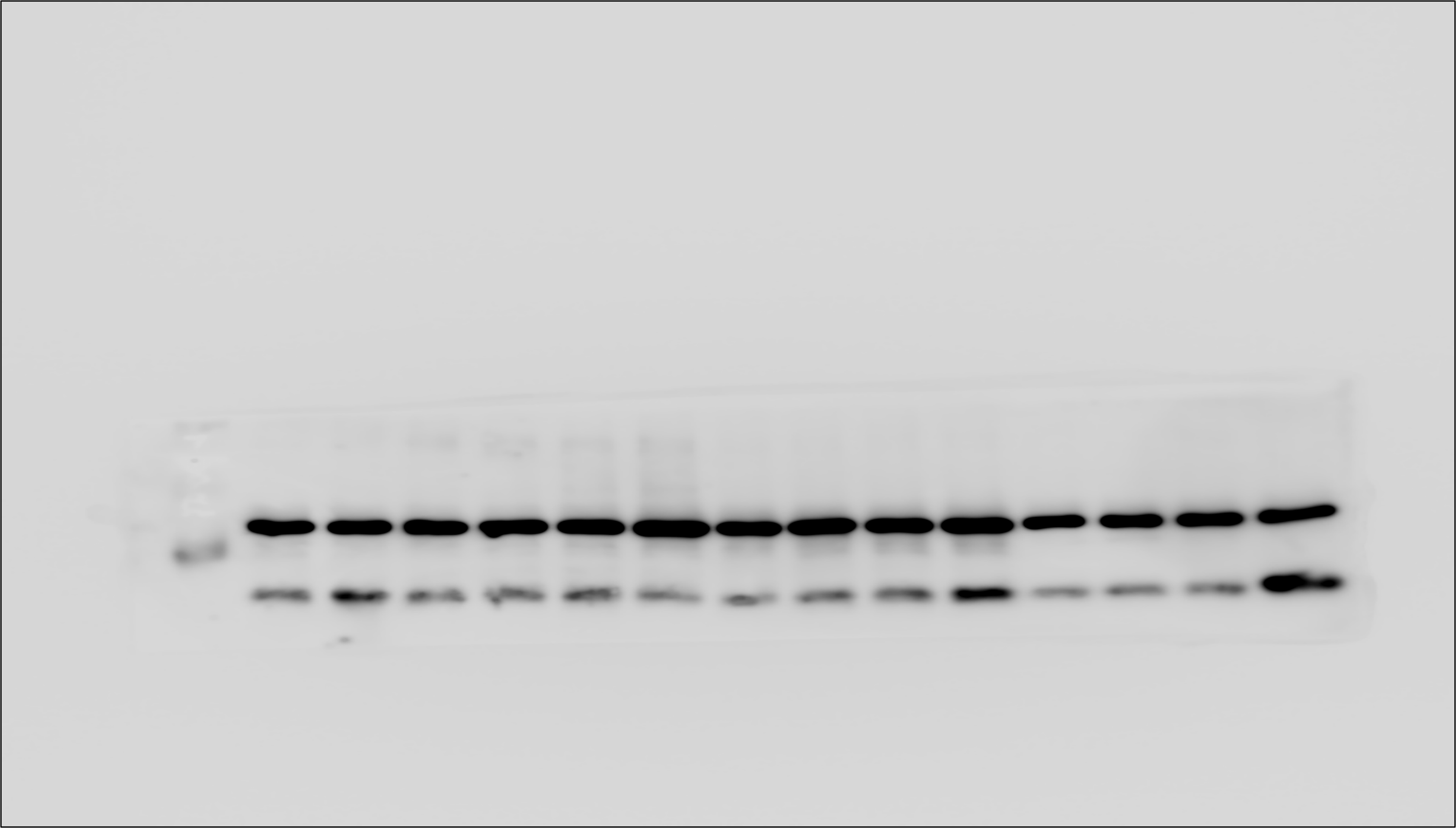

Supplement: Figure 9—figure supplement 1—source data 2. [file elife-108048-fig9-figsupp1-data2.zip › Figure 9-figure supplement 1/Figure S8 H-IP-Myc.tif]

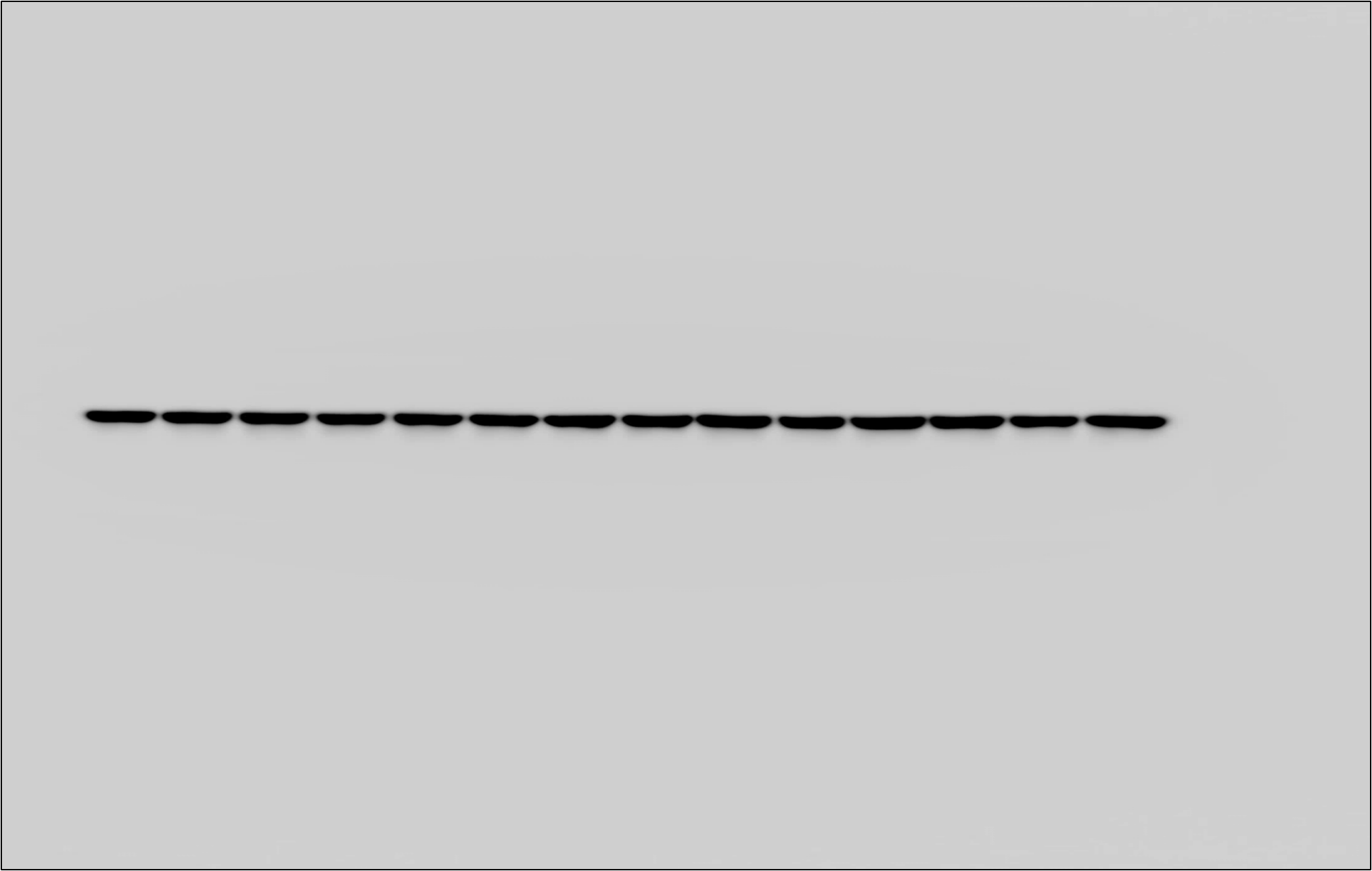

Supplement: Figure 9—figure supplement 1—source data 2. [file elife-108048-fig9-figsupp1-data2.zip › Figure 9-figure supplement 1/Figure S8 H-WCL-Actin.tif]

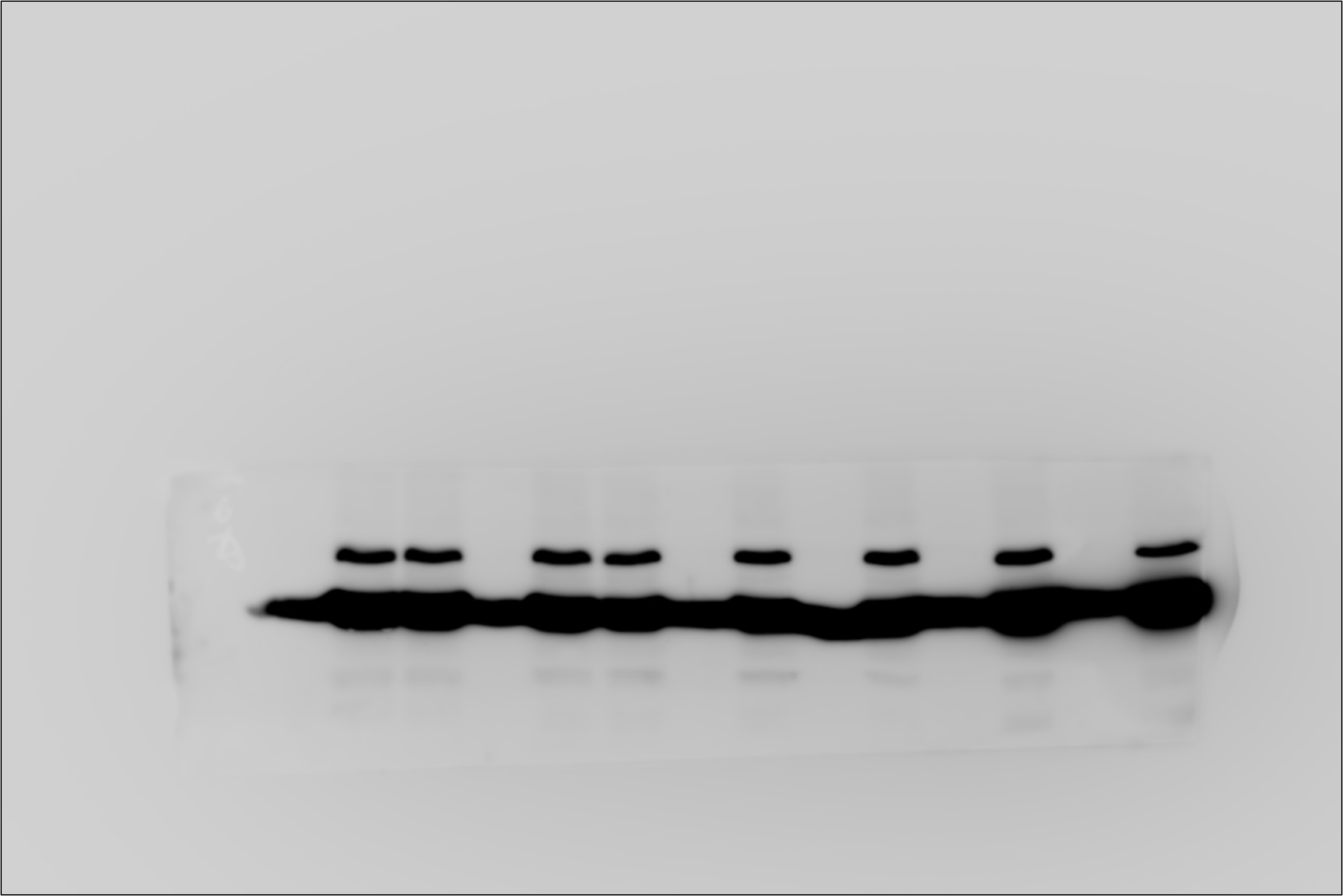

Supplement: Figure 9—figure supplement 1—source data 2. [file elife-108048-fig9-figsupp1-data2.zip › Figure 9-figure supplement 1/Figure S8 H-WCL-Flag.tif]

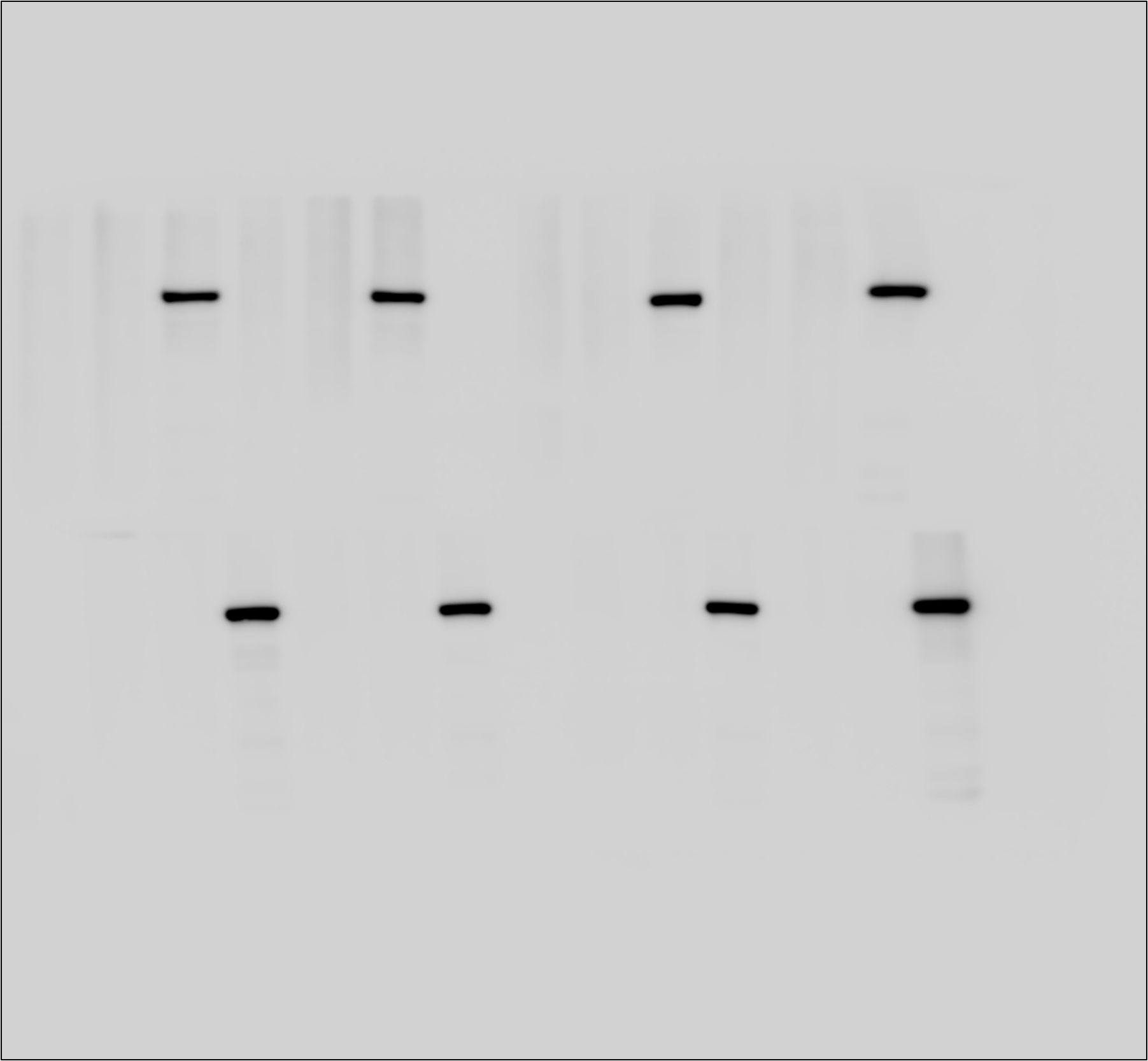

Supplement: Figure 9—figure supplement 1—source data 2. [file elife-108048-fig9-figsupp1-data2.zip › Figure 9-figure supplement 1/Figure S8 H-WCL-HA-USP8.tif]
