# Supplementary figures and images for: Male-biased Cyp17a2 orchestrates antiviral sexual dimorphism in fish via STING stabilization and viral protein degradation (part 5 of 5)
Source: eLife. 2026 Feb 18;14:RP108048. doi: 10.7554/eLife.108048 (PMC12916102; doi:10.7554/eLife.108048)

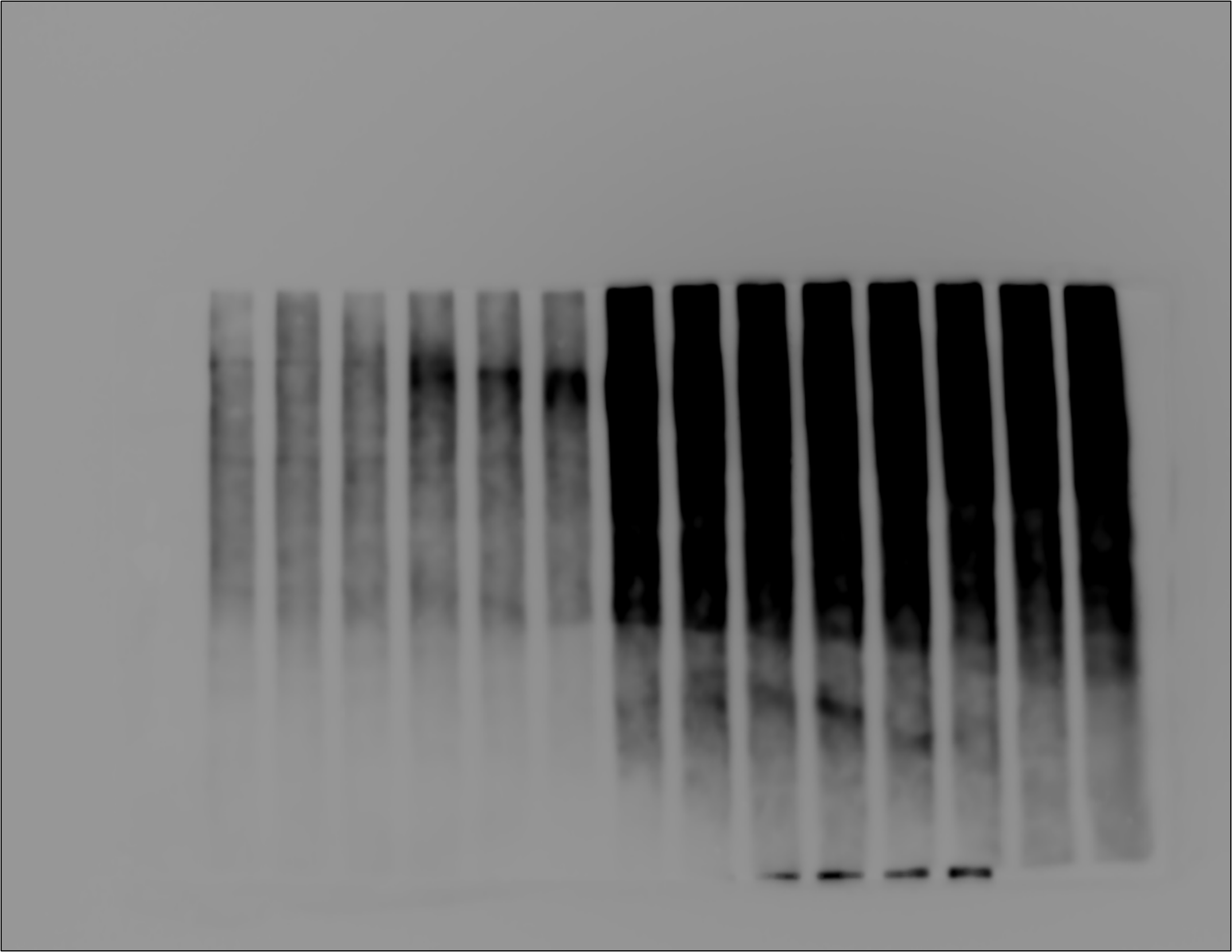

Supplement: Figure 9—figure supplement 1—source data 2. [file elife-108048-fig9-figsupp1-data2.zip › Figure 9-figure supplement 1/Figure S8 H-WCL-HA.tif]

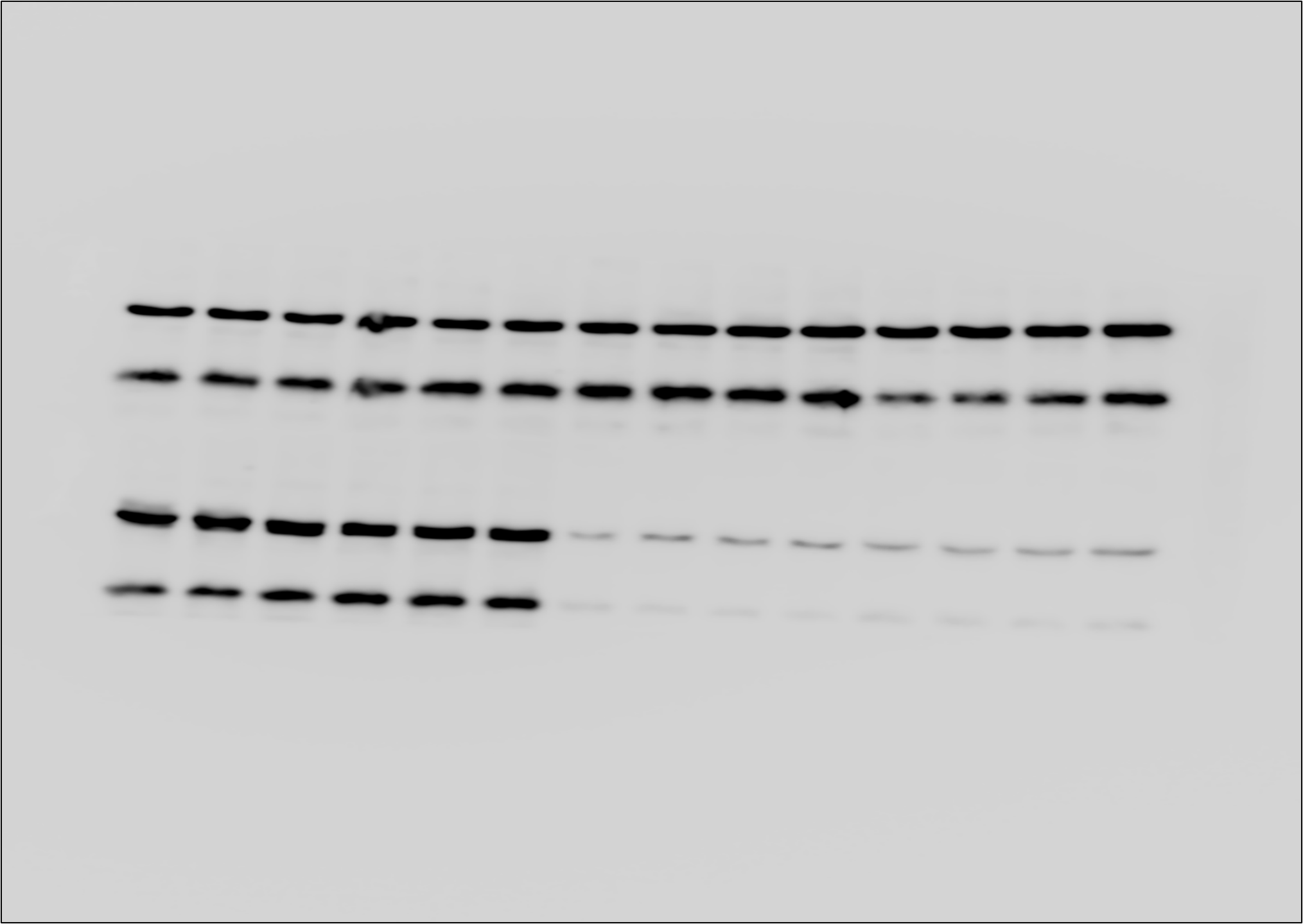

Supplement: Figure 9—figure supplement 1—source data 2. [file elife-108048-fig9-figsupp1-data2.zip › Figure 9-figure supplement 1/Figure S8 H-WCL-Myc.tif]

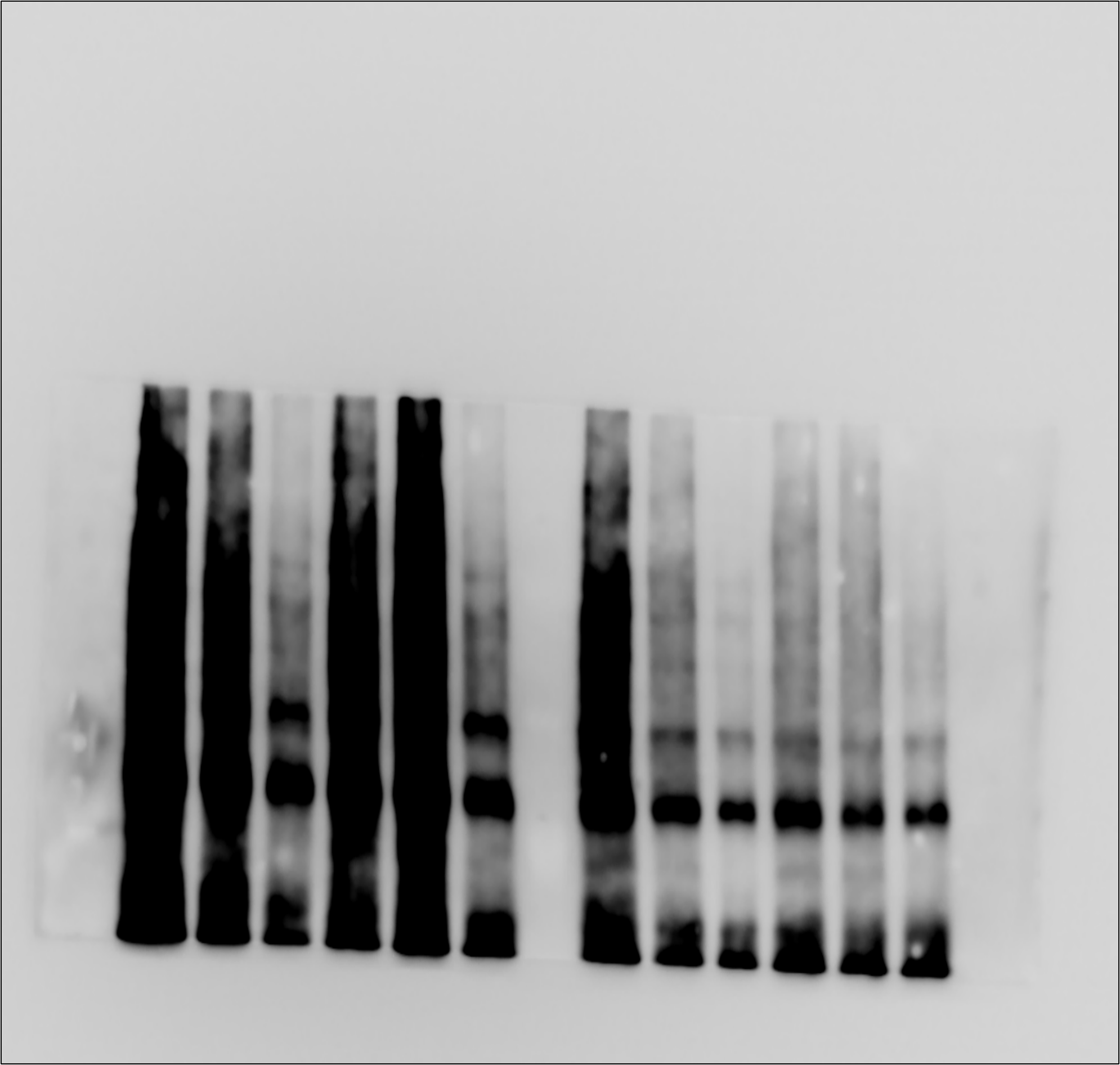

Supplement: Figure 9—figure supplement 1—source data 2. [file elife-108048-fig9-figsupp1-data2.zip › Figure 9-figure supplement 1/Figure S8 I-IP-HA.tif]

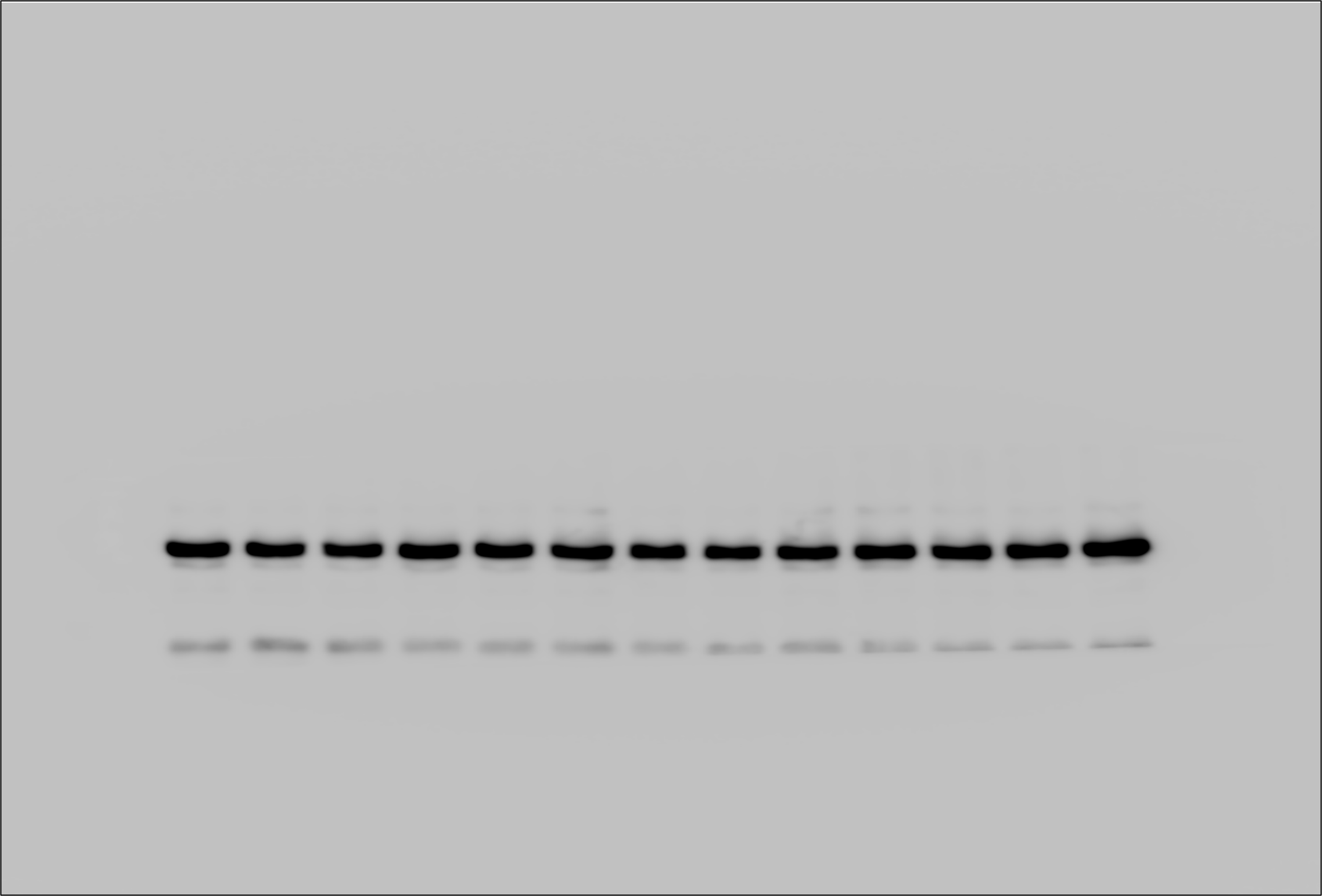

Supplement: Figure 9—figure supplement 1—source data 2. [file elife-108048-fig9-figsupp1-data2.zip › Figure 9-figure supplement 1/Figure S8 I-IP-Myc.tif]

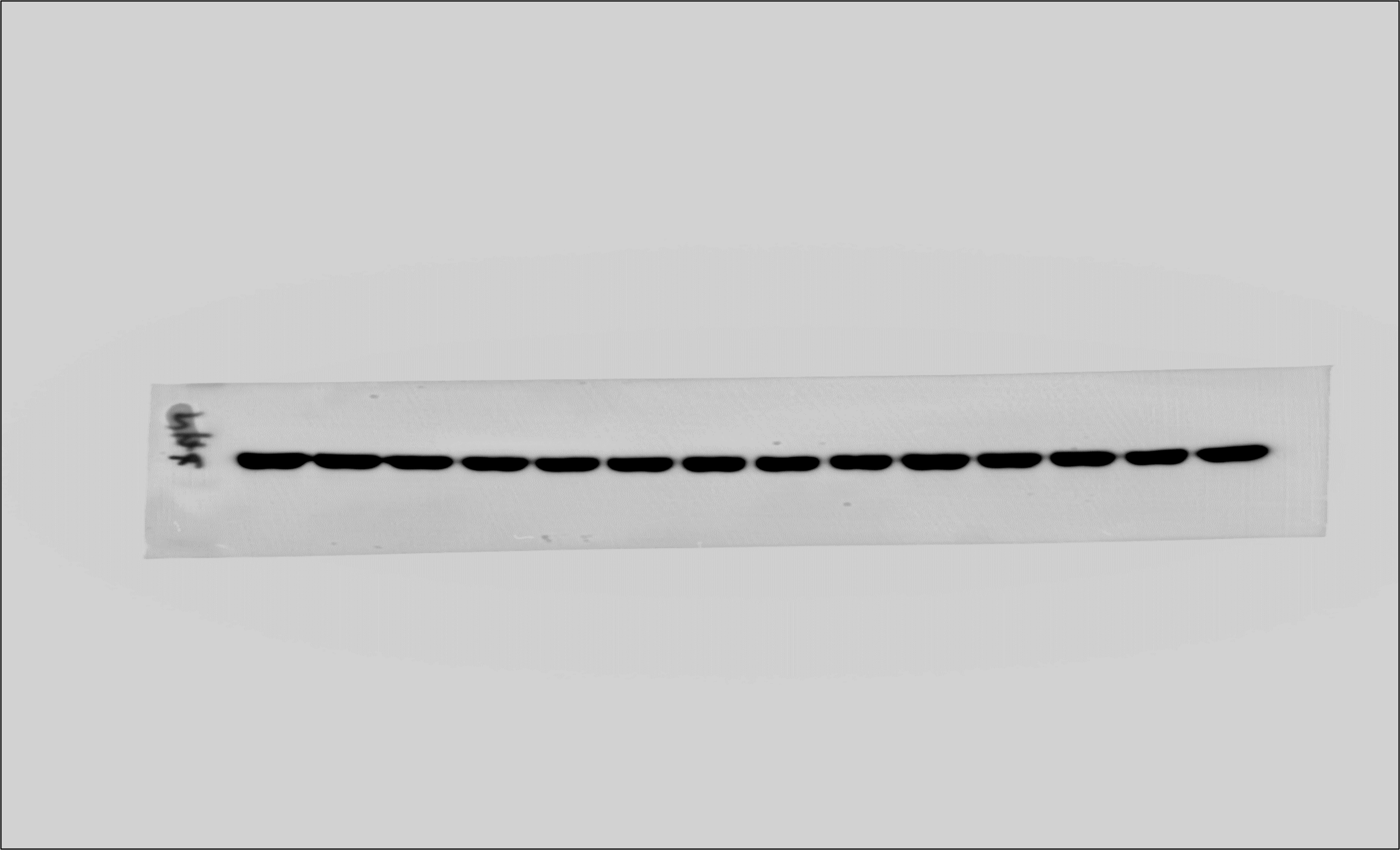

Supplement: Figure 9—figure supplement 1—source data 2. [file elife-108048-fig9-figsupp1-data2.zip › Figure 9-figure supplement 1/Figure S8 I-WCL-Actin.tif]

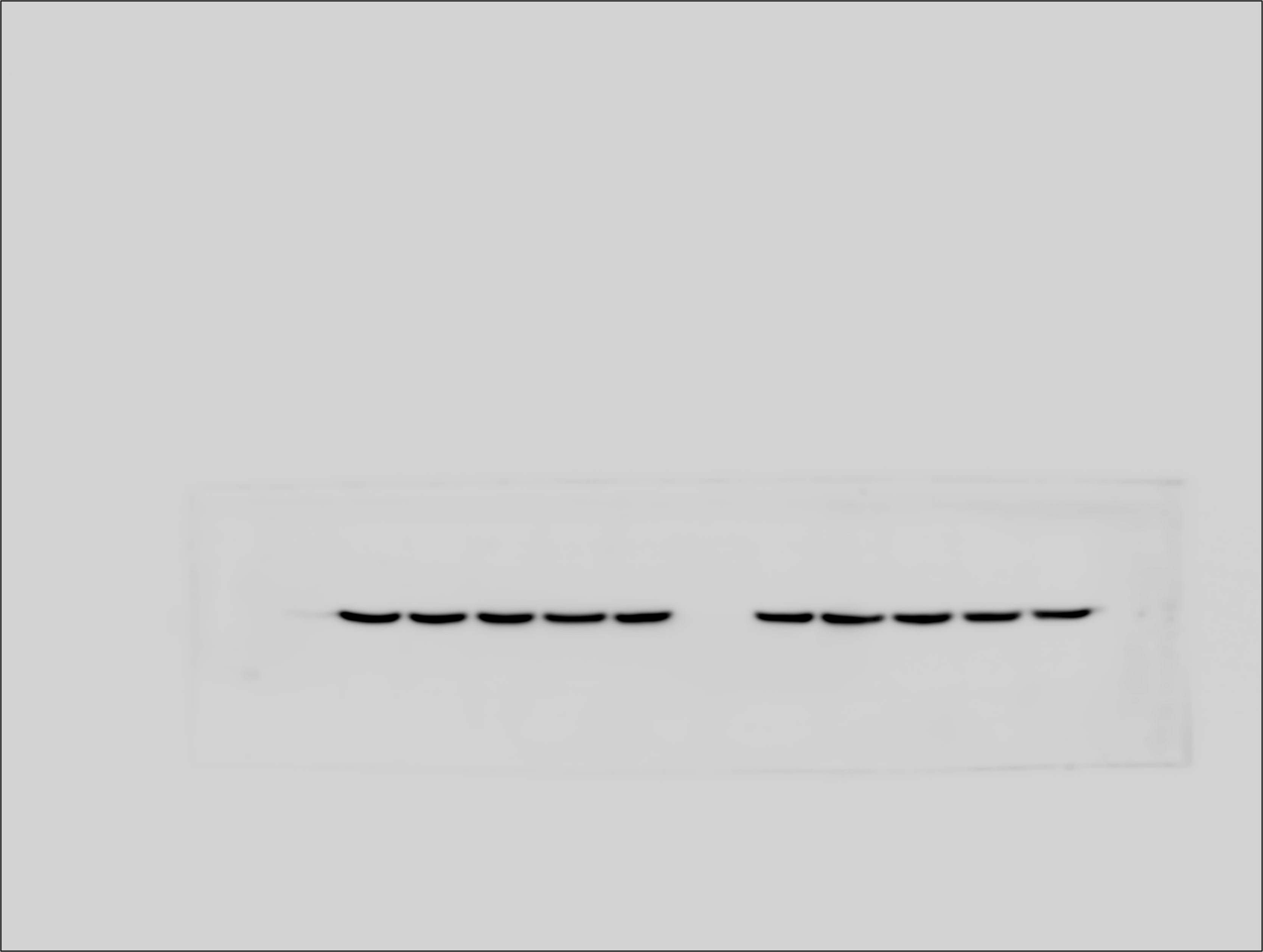

Supplement: Figure 9—figure supplement 1—source data 2. [file elife-108048-fig9-figsupp1-data2.zip › Figure 9-figure supplement 1/Figure S8 I-WCL-Flag.tif]

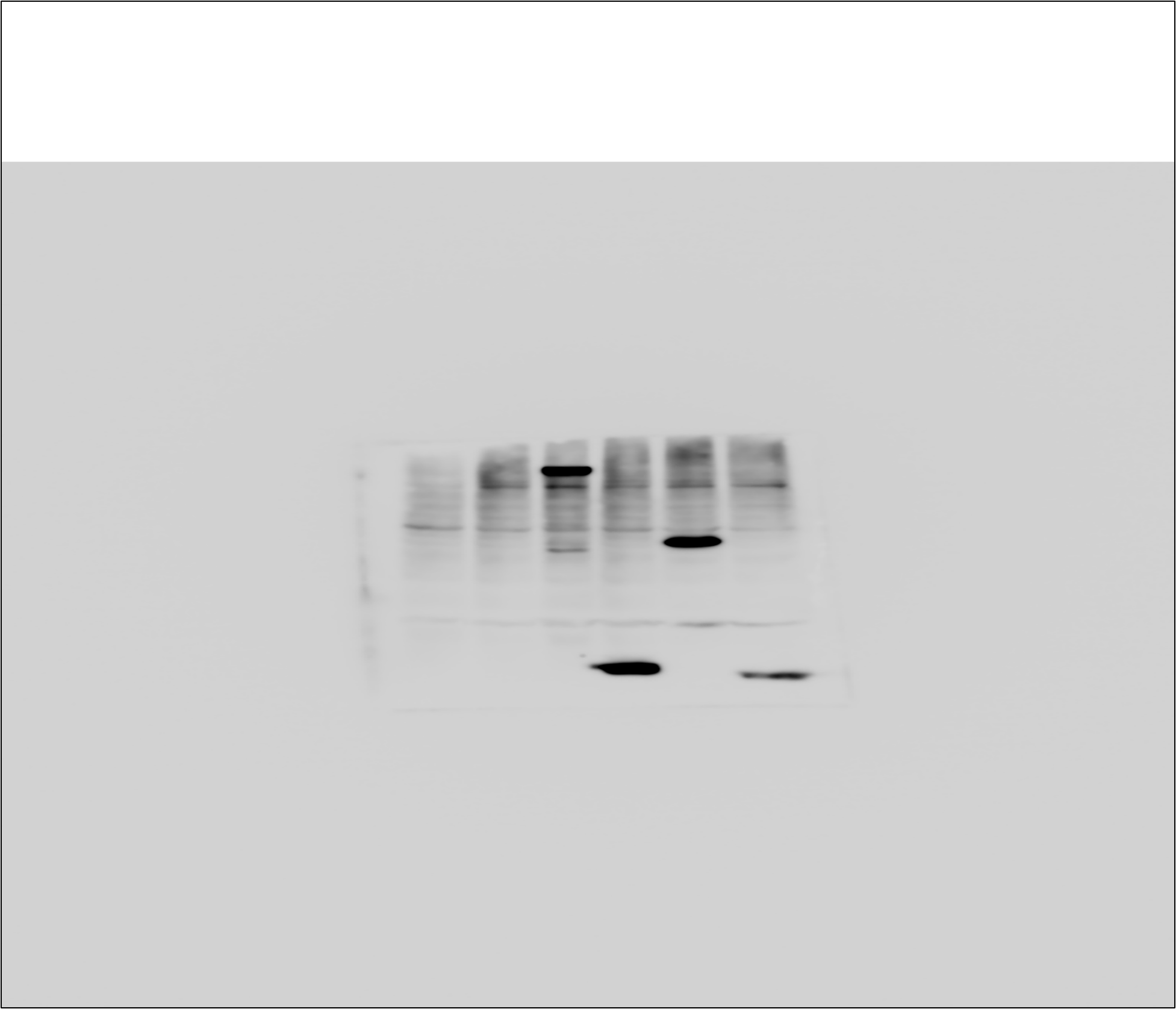

Supplement: Figure 9—figure supplement 1—source data 2. [file elife-108048-fig9-figsupp1-data2.zip › Figure 9-figure supplement 1/Figure S8 I-WCL-HA.tif]

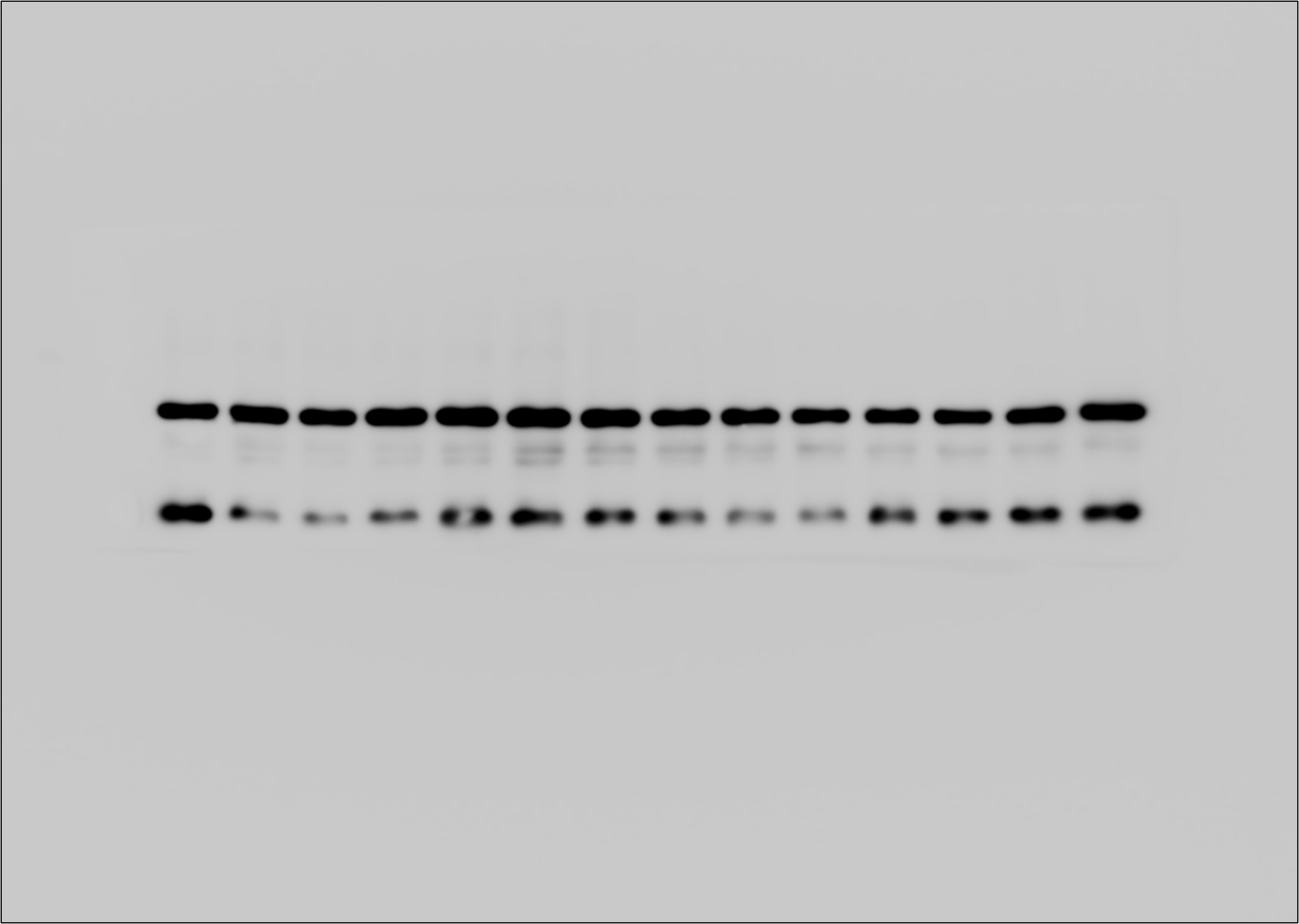

Supplement: Figure 9—figure supplement 1—source data 2. [file elife-108048-fig9-figsupp1-data2.zip › Figure 9-figure supplement 1/Figure S8 I-WCL-Myc.tif]
